# Endometriosis-Associated Intestinal Tumor: A Case Report and Literature Review with Comprehensive Proteomic Data

**DOI:** 10.70352/scrj.cr.25-0761

**Published:** 2026-03-11

**Authors:** Naoyuki Toyota, Yuki Yoshimatsu, Chihiro Kamozawa, Ryo Nakanishi, Shin Fujita, Makoto Abe, Ryo Konno, Yusuke Kawashima, Tadashi Kondo

**Affiliations:** 1 Department of Colorectal Surgery, Tochigi Cancer Center, Utsunomiya, Tochigi, Japan; 2 Department of Patient-Derived Cancer Model, Tochigi Cancer Center Research Institute, Utsunomiya, Tochigi, Japan; 3 Department of Gynaecology, Tochigi Cancer Center, Utsunomiya, Tochigi, Japan; 4 Department of Pathology, Tochigi Cancer Center, Utsunomiya, Tochigi, Japan; 5 Department of Applied Genomics, Kazusa DNA Research Institute, Kisarazu, Chiba, Japan; 6 Department of Cancer Proteogenomics, Tochigi Cancer Center Research Institute, Utsunomiya, Tochigi, Japan; 7 Division of Rare Cancer Research, National Cancer Center Research Institute, Chuo-ku, Tokyo, Japan

**Keywords:** endometriosis, intestinal tumor, endometrioid adenocarcinoma, EAIT, proteomics, pelvic exenteration

## Abstract

**INTRODUCTION:**

Endometriosis-associated intestinal tumors (EAITs) are rare malignancies that arise from ectopic endometrial tissue, and their clinical and molecular characteristics remain poorly defined.

**CASE PRESENTATION:**

We report a 53-year-old woman who presented with rectal bleeding. Imaging revealed a rectal mass invading the uterus. An initial endoscopic biopsy was inconclusive; however, a subsequent CT-guided biopsy confirmed adenocarcinoma. Immunohistochemical analysis suggested a gynecological origin. The patient underwent posterior total pelvic exenteration with bilateral lymphadenectomy. Histopathological evaluation confirmed endometriosis-associated endometrioid adenocarcinoma with ovarian metastasis. Postoperative chemotherapy was administered, and the patient has remained disease-free for 3.5 years.

**CONCLUSIONS:**

This case highlights the diagnostic challenges and clinical complexity of EAITs. Tumor proteomic profiling provided additional biological insights and may serve as a reference for future comparative and translational research.

## Abbreviations

EAIT
: endometriosis-associated intestinal tumors
^18^F-FDG-PET
: fluorine-18 fluorodeoxyglucose-PET

## INTRODUCTION

Endometriosis is a benign, non-neoplastic condition characterized by the ectopic proliferation of endometrial tissue. Approximately 80% of cases involve the ovaries, whereas about 12% affect the intestines, with the rectum and sigmoid colon being the most frequently involved sites.^1)^ Malignant transformation of intestinal endometriosis is exceedingly rare. The first case of endometriosis-associated adenocarcinoma was reported by Sampson in 1925.^2)^ Since then, only a few comprehensive reports have been published, including a notable series of 23 cases by Slavin et al. in 2000.^3)^

Despite these efforts, the clinicopathological and molecular features of EAITs remain poorly understood. In this report, we describe a rare case of rectal EAIT and provide an integrated clinicopathological analysis in the context of previously published cases, including 15 reported in the past literature. Additionally, we conducted a comprehensive proteomic analysis of the tumor tissue using mass spectrometry (**Supplementary Table 1**). The resulting global protein expression profile was deposited in the publicly accessible Japan ProteOme STandard (jPOST) repository, providing a reference resource that may support future comparative analyses as more cases are accumulated.

## CASE PRESENTATION

A 53-year-old woman presented with rectal bleeding and was referred to a secondary hospital for further evaluation.

### History of present illness

She reported persistent dull pain in the lower abdomen but denied other gastrointestinal symptoms such as weight loss, vomiting, nausea, or diarrhea.

### Past medical and family history

The patient had no notable past medical history except for a prior anal fistula, and no relevant family history.

### Physical examination

The patient appeared well, with a body mass index of 21.8 kg/m^2^. Abdominal examination revealed no tenderness or palpable masses, and there was no enlargement of the spleen, liver, or lymph nodes. However, gynecological examination revealed a palpable mass in the pouch of Douglas with mild tenderness. Digital rectal examination identified a solid mass on the anterior wall of the rectum.

### Laboratory findings

Routine hematological and biochemical tests were within normal limits, including liver and renal function tests. Tumor markers, including carcinoembryonic antigen and carbohydrate antigen 19-9, were within normal ranges. However, CA125 was elevated at 148.0 U/mL (normal range: 0–35 U/mL), suggesting a potential gynecologic malignancy.

### Imaging studies

Initial colonoscopy at the referring hospital revealed an 8-cm segmental stenosis in the rectum (Rb–Rs), suspected to result from extramural compression. Endoscopic biopsy was inconclusive, prompting referral for further investigation.

**Fig. 1:**
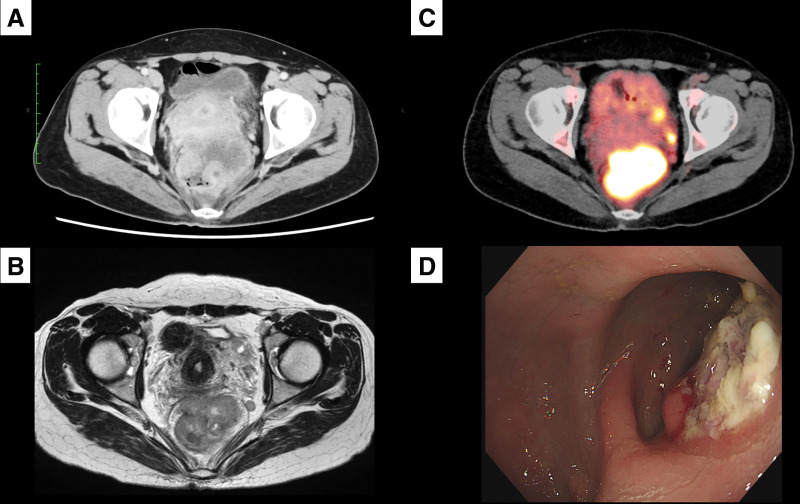
Preoperative multimodal imaging studies. (**A**) Contrast-enhanced abdominal CT demonstrating a highly invasive, bulky tumor occupying more than half of the pelvic cavity and involving both the rectum and the uterus. (**B**) Pelvic MRI showing circumferential rectal wall thickening with heterogeneous enhancement, suggestive of invasion into the uterovaginal junction. (**C**) Fluorine-18 fluorodeoxyglucose-PET revealing marked tracer uptake in the rectal tumor as well as in the bilateral lateral pelvic lymph nodes. (**D**) Repeat colonoscopy identifying the lower tumor margin approximately 6 cm from the anal verge. Extramural compression was again observed, accompanied by partial mucosal ulceration with a villous tumor-like appearance.

Contrast-enhanced CT demonstrated a poorly defined 7-cm mass on the anterior rectal wall, with suspected extensive invasion into the uterus and prominent pelvic and lateral lymphadenopathy (**Fig. 1A**). Pelvic MRI showed circumferential wall thickening of the rectum with heterogeneous enhancement, raising suspicion for invasion into the uterovaginal junction (**Fig. 1B**). PET with ^18^F-FDG-PET revealed significant tracer uptake in the rectal lesion and bilateral lateral pelvic lymph nodes (**Fig. 1C**).

A second colonoscopy identified the tumor’s lower edge approximately 6 cm from the anal verge. Extramural compression was again observed, with partial mucosal ulceration resembling a villous tumor (**Fig. 1D**). However, biopsy specimens demonstrated only necrotic tissue and no evidence of malignancy.

**Fig. 2:**
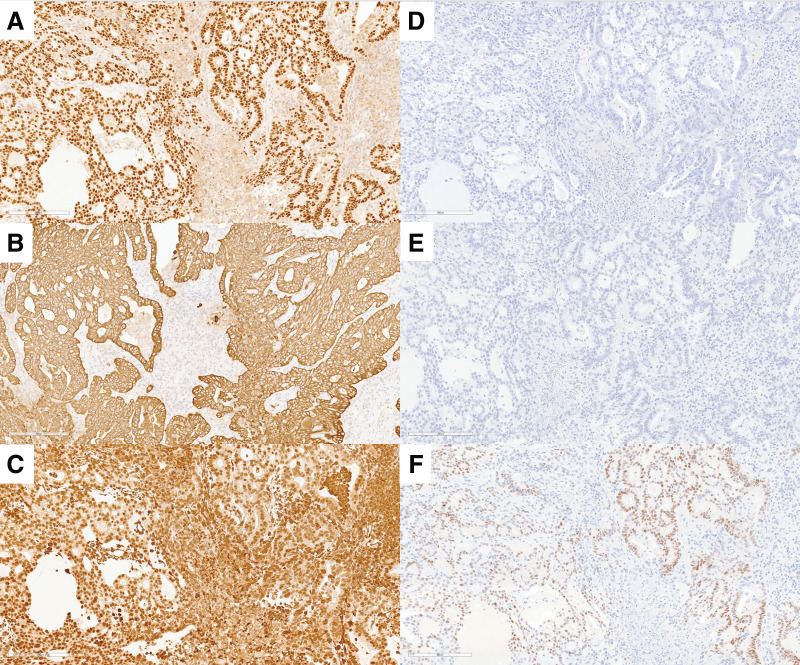
Immunohistochemical (IHC) staining of tumor cells for six markers (original magnification ×200). (**A**) PAX8 (positive). (**B**) CK7 (positive). (**C**) Vimentin (positive). (**D**) CK20 (negative). (**E**) CDX2 (negative). (**F**) Estrogen receptor (ER) (positive).

To establish a definitive diagnosis, a CT-guided needle biopsy was performed. Histopathological analysis confirmed adenocarcinoma. Immunohistochemical stains showed the tumor cells to be positive for PAX8, CK7, estrogen receptor (ER), and vimentin, and negative for CK20 and CDX2, findings consistent with a gynecologic origin (**Fig. 2A**–**2F**).

### Final diagnosis

Based on the clinicopathological findings, the diagnosis of EAIT was established.

### Treatment

Given the aggressive clinical presentation and precedent from previous case reports, the patient underwent posterior pelvic exenteration. The procedure included low anterior resection, total hysterectomy, bilateral salpingo-oophorectomy, vaginal cuff resection, and bilateral lateral pelvic lymph node dissection, in collaboration with the gynecology team. No macroscopic intraperitoneal metastasis was identified intraoperatively. Owing to bilateral tumor extension, resection of both pelvic plexus nerves was performed.

### Pathological findings

**Fig. 3:**
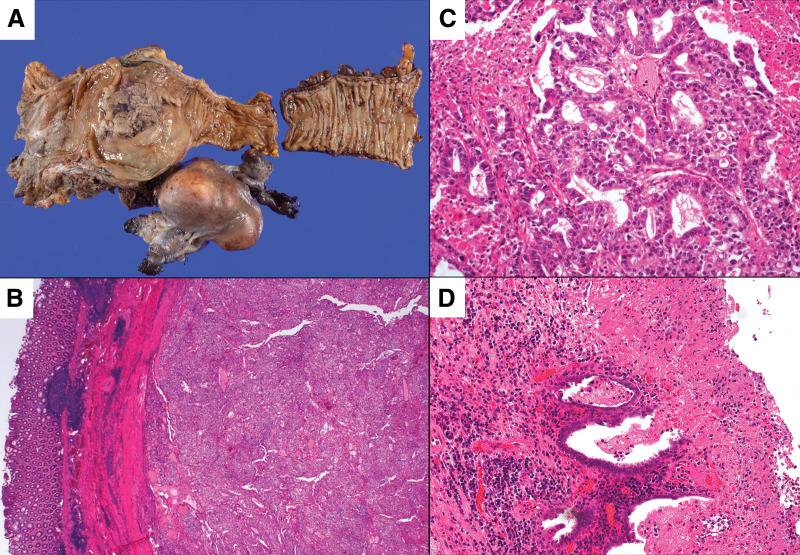
Histopathological features of the resected specimen. (**A**) Macroscopic examination of the resected specimen showing a tumor located predominantly on the anterior rectal wall with mucosal ulceration. (**B**) Low-power magnification (original magnification ×40) showing tumor invasion into the rectal muscularis propria. (**C**) High-power magnification (original magnification ×200) demonstrating neoplastic glands composed of columnar cells with clear to eosinophilic cytoplasm, consistent with endometrioid carcinoma. (**D**) Foci of endometriosis identified on the rectal serosa adjacent to the tumor (original magnification ×200).

Gross examination revealed a 70 × 67 × 48 mm tumor extending from the rectosigmoid junction to the upper rectum, located predominantly on the anterior rectal wall with mucosal ulceration (**Fig. 3A**), and invading the uterine cervix. Microscopically, the lesion displayed glandular and solid architecture, composed of columnar cells with eosinophilic and clear cytoplasm, mimicking endometrial-type glands (**Fig. 3B** and **3C**). Foci of adjacent endometriosis were identified (**Fig. 3D**). Based on these findings, the tumor was diagnosed as endometriosis-associated endometrioid adenocarcinoma. Surgical margins were negative. No lymph node metastasis was identified, but ovarian metastasis was confirmed.

### Postoperative course and follow-up

The postoperative course was uneventful, except for the development of a neurogenic bladder. Because of the ovarian metastasis, six cycles of adjuvant chemotherapy with paclitaxel and carboplatin (TC regimen) were administered. The patient has remained disease-free for 3.5 years.

### Proteomic analysis

Tumor tissue was obtained from the Tochigi Cancer Biobank. Samples were collected intraoperatively under the supervision of a certified pathologist and preserved in vapor-phase liquid nitrogen. Informed consent was obtained from the patient. Ethical approval was granted by the Tochigi Cancer Center Ethics Committee (Approval No. 21-A037). Proteomic profiling was performed using mass spectrometry, as described in the Supplementary Methods. A total of 9294 proteins were identified (**Supplementary Table 1**), and the data have been deposited in the jPOST database (Accession Nos.: JPST004032 and PXD067723). The dataset is publicly available through the ProteomeXchange Consortium via the jPOST partner repository.

## DISCUSSION

Endometriosis is characterized by the presence of endometrial glands and stroma outside the uterine cavity. Although its etiology remains unclear, Sampson’s theory of retrograde menstruation is the most widely accepted hypothesis. According to this theory, endometrial fragments reflux through the fallopian tubes into the peritoneal cavity during menstruation, where they adhere to and infiltrate the pelvic organs. Endometriosis affects approximately 10%–15% of women of reproductive age. Based on anatomical distribution, it is classified into common sites (e.g., ovaries, uterosacral ligaments, pouch of Douglas, peritoneum), less common sites (e.g., intestines, ureters, bladder), and rare sites (e.g., lungs, pleura, soft tissues).^4)^

Among these, intestinal endometriosis represents the most frequent form of extrapelvic disease, occurring in 12%–37% of all endometriosis cases. The rectum and sigmoid colon are the most commonly involved intestinal sites (84%), followed by the colon (7%), cecum (5%), and appendix (3%).^5)^ While intestinal endometriosis is often managed conservatively, its malignant transformation—referred to as EAITs—is of major clinical concern. Approximately 0.7%–1.0% of all endometriosis cases undergo malignant transformation, with 78.7% of these occurring in the ovaries and the remaining 21.3% in extragonadal sites.^6)^ In a Japanese nationwide survey by Mandai et al., 7 out of 11 cases of malignant transformation in rare-site endometriosis involved the intestines.^4)^

EAITs are exceedingly rare, and no consensus exists regarding their optimal management. This study provides two main contributions. First, it represents the first attempt to investigate EAITs using proteomic analysis. Second, given that fewer than ten cases of EAIT have been reported in the Japanese population, presenting a Japanese EAIT case with proteomic analysis results is particularly noteworthy. In this study, proteomic analysis was prioritized over genomic or transcriptomic approaches to address the specific clinical and pathological questions raised by this rare case. While genomic and transcriptomic profiling are powerful tools for identifying recurrent molecular alterations across cohorts, their interpretability is inherently limited in single-case studies, particularly when the goal is to understand functional tumor biology. Proteomics captures the integrated downstream consequences of genetic, epigenetic, and transcriptional regulation, thereby providing a more direct representation of the tumor’s active molecular state. This feature makes proteomic profiling especially suitable for exploratory analyses aimed at linking molecular signatures with histopathological features and clinical behavior in rare or atypical presentations.

To date, we have identified 56 previously reported cases published in the English literature, which are summarized in **Table 1**.^3,7–37)^ The median age of onset ranged from 36 to 83 years, with a peak incidence in the 60s, followed by the 50s and 40s. About half of the patients presented with abdominal pain or gastrointestinal symptoms such as rectal bleeding, melaena, or hematochezia. Acute presentations, including bowel obstruction or peritonitis, were rare. Consistent with previous studies, the rectum was the most frequent site of EAITs.

**Table 1:** Summary of the literature on endometriosis-associated intestinal tumors

Case no.	Author	Year	Age	Site	Prodromal symptoms	Diagnosis	Treatment	Lymph nodes metastasis	Postoperative therapy	Outcome	Follow-up (month)
1	Nylander^7)^	1938	36	Rectum	Abdominal pain	Spindle cell sarcoma	Surgery	(Not available)	None	AWOD	(Not available)
2	Marx^8)^	1949	48	Sigmoid	Lower abdominal pain	Squamous cell carcinoma	Surgery	(Not available)	None	(Not available)	(Not available)
3	Ferraro^9)^	1956	44	Ileum	Abdominal pain, LLQ mass, Melena	Endometrial sarcoma	Sigmoidectomy with R2 resection	(Not available)	Radiation	DOD	4
4	Weinrod^10)^	1956	59	Sigmoid	Abdominal discomfort, Melena	Adenocanthoma	Small bowel resection	Negative	None	(Not available)	(Not available)
5	Scully^11)^	1966	64	Sigmoid	Frequent bowel movements, Melena	Endometrial stromal sarcoma	Sigmoidectomy	(Not available)	None	DOC	216
6	Reintoft^12)^	1974	36	Rectum	Not given	Endometrioid carcinoma	Hysterectomy, left salpingo-oophorectomy, and rectal resection	Positive	Radiation	DOD	4
7	Weisz-Carrington^13)^	1977	77	Cecum	Melena, Distension	Carcinosarcoma	Ileocolectomy	Positive	None	DOD	1
8	Lott^14)^	1978	52	Rectum	Hematochezia	Endometrioid carcinoma	Anterior resection	(Not available)	None	AWOD	(Not available)
9	Grimes^15)^	1980	44	Cecum and terminal ileum	Abdominal pain	Adenosquamous carcinoma	Right colectomy	Positive (9/20)	None	DOD	10
10	Amano^16)^	1981	44	Sigmoid	Abdominal distress, Melena	Endometrioid carcinoma	Sigmoidectomy	Negative	None	AWOD	(Not available)
11	Lankerani^17)^	1982	49	Sigmoid	Abdominal discomfort	Mixed germ cell tumor	Hysterectomy, Bilateral salpingo-oophorectomy, and rectal resection	(Not available)	None	DOD	12
12	Chumas^18)^	1986	67	Rectosigmoid	Frequent bowel movements, Melena	Carcinosarcoma	Hartmann procedure with distal right ureter resection	Positive (1/20)	Chemotherapy	DOD	24
13	Baiocchi^19)^	1990	38	Terminal ileum	Abdominal pain	Endometrial stromal sarcoma	Partial ileal resection; transverse and ascending colectomy with R2 resection	(Not available)	Chemotherapy	AWOD	(Not available)
14	Hitti^20)^	1990	39	Ascending colon	Lower abdominal pain	Clear cell carcinoma	Sigmoid colectomy with total hysterectomy and bilateral salpingo-oophorectomy	Negative	Chemotherapy, Radiation	AWOD	9
15	Yasui^21)^	1992	44	Sigmoid	Ileus	Endometrioid carcinoma	Low anterior resection	(Not available)	None	(Not available)	(Not available)
16	Duun^22)^	1993	62	Rectum	Pelvic mass	Endometrioid carcinoma	Anterior resection of the rectosigmoid colon	(Not available)	Radiation	DOD	12
17	Jiang^23)^	1993	42	Sigmoid	Abdominal mass	Endometrioid carcinoma	Surgery	(Not available)	Chemotherapy	AWOD	6
18	Slavin^3)^	2000	62	Rectum	Perirectal mass	Endometrioid carcinoma	Segmental rectal resection with peritoneal mass excision, sigmoid colectomy, and TAH-BSO	Negative	None	AWOD	24
19	Slavin^3)^	2000	38	Rectum	Abdominal pain, Hematochezia	Endometrioid carcinoma	Sigmoidectomy, TAH + BSO, and omentectomy	Negative	None	AWOD	24
20	Slavin^3)^	2000	54	Sigmoid	Vaginal bleeding	Endometrioid carcinoma	Extirpation of pelvic mass with partial vaginectomy and additional sigmoid colectomy	Negative	None	AWOD	12
21	Slavin^3)^	2000	49	Sigmoid	Abdominal pain in LLQ	Adenofibroma	Sigmoidectomy	Negative	None	AWOD	36
22	Slavin^3)^	2000	47	Sigmoid	Small bowel obstruction	Adenosarcoma	Segmental distal ileal resection with right anterior pelvic sidewall resection	Negative	Chemotherapy (adriamycin, ifosfamide, etoposide)	AWOD	72
23	Slavin^3)^	2000	50	Sigmoid	Melena, pelvic mass	Carcinosarcoma	Sigmoidectomy with right pelvic sidewall resection	Positive	Chemotherapy (cisplatin, megestrol acetate)	DOD	5
24	Yantiss^24)^	2000	52	Sigmoid	Obstruction	Endometrioid carcinoma	Segmental colectomy	Negative	None	AWOD	1
25	Yantiss^24)^	2000	66	Rectum (rectovaginal septum)	Pelvic mass, pelvic pain	Endometrioid carcinoma	Segmental colectomy with R2 resection	Negative	Radiation	AWOD	38
26	Yantiss^24)^	2000	74	Sigmoid	Obstruction	Endometrioid carcinoma	Segmental colectomy	Positive	None	DOD	12
27	Yantiss^24)^	2000	47	Rectum (rectovaginal septum)	Pelvic pain, pelvic mass	Endometrioid carcinoma	Segmental colectomy	Negative	Radiation	AWOD	9
28	Yantiss^24)^	2000	67	Rectum (rectovaginal septum)	Vaginal bleeding	Endometrioid carcinoma	Segmental colectomy	Negative	None	AWOD	156
29	Yantiss^24)^	2000	48	Rectum (rectovaginal septum)	Acute abdomen, Fecal peritonitis	Endometrioid carcinoma	Segmental colectomy	(Not available)	(Not available)	(Not available)	(Not available)
30	Yantiss^24)^	2000	60	Colon	(Not available)	Endometrioid carcinoma	Segmental colectomy	Positive	Chemotherapy	AWD	24
31	Yantiss^24)^	2000	62	Sigmoid	(Not available)	Endometrioid carcinoma	Segmental colectomy	(Not available)	(Not available)	(Not available)	(Not available)
32	Yantiss^24)^	2000	65	Small bowel	Chronic abdominal pain, Hypermenorrhea	Endometrioid adenofibroma, adenocarcinoma *in situ*	Segmental small bowel resection	Negative	None	AWOD	3
33	Yantiss^24)^	2000	36	Sigmoid	Abdominal pain	Endometrioid carcinoma	Segmental colectomy	Negative	None	AWOD	36
34	Yantiss^24)^	2000	50	Colon	(Not available)	Mullerian adenosarcoma	Segmental colectomy	(Not available)	(Not available)	AWOD	24
35	Yantiss^24)^	2000	83	Small bowel	Abdominal mass, Obstruction	Mullerian adenosarcoma	Segmental small bowel resection	(Not available)	(Not available)	(Not available)	(Not available)
36	Yantiss^24)^	2000	43	Small bowel	(Not available)	Mullerian adenosarcoma	Segmental colectomy with R2 resection	(Not available)	(Not available)	(Not available)	(Not available)
37	Yantiss^24)^	2000	63	Rectum	(Not available)	Endometrioid stromal sarcoma	Segmental colectomy	Negative	Radiotherapy	AWD	36
38	Yantiss^24)^	2000	57	Sigmoid	Obstruction, BRBPR	Mild atypical hyperplasia	Segmental colectomy	Negative	None	AWOD	1
39	Yantiss^24)^	2000	63	Rectum	Progressive change bowel habits	Moderate atypical hyperplasia, endometriosis	Segmental colectomy	(Not available)	(Not available)	AWOD	60
40	Yantiss^24)^	2000	59	Sigmoid	Obstruction	Adenocarcinoma in situ	Segmental colectomy	Negative	None	AWOD	192
41	Magtibay^25)^	2001	76	Rectum	Diarrhea, rectal bleeding	Endometrioid adenocarcinoma	Low anterior resection with colonic J-pouch anal anastomosis and diverting right transverse colostomy	Negative	Radiation	AWOD	21
42	Jones^26)^	2002	52	Sigmoid	Rectal bleeding	Endometrioid adenocarcinoma	Anterior resection of the rectosigmoid colon	Negative	None	AWOD	9
43	Chen^27)^	2002	80	Rectum	Constipation, Hematochezia	Endometrioid adenocarcinoma	Rectosigmoid segmental resection	Positive (5/10)	(Not available)	(Not available)	(Not available)
44	Petersen^28)^	2002	61	Sigmoid	Diarrhea	Endometrioid adenocarcinoma	Anterior resection with formation of a loop ileostomy	Negative	(Not available)	(Not available)	(Not available)
45	Petersen^28)^	2002	47	Rectum	Large bowel obstruction	Endometrioid adenocarcinoma	Sigmoidectomy, TAH + BSO	Positive (3/18)	(Not available)	(Not available)	(Not available)
46	Petersen^28)^	2002	57	Rectum	Lower abdominal pain and mass lesion	Endometrioid adenocarcinoma	Anterior resection	Negative	(Not available)	(Not available)	(Not available)
47	Debus^29)^	2001	50	Rectum	Abdominal mass	Endometrioid adenocarcinoma	Rectosigmoid colectomy with diverting ileostomy, omentectomy, and appendectomy	Negative	Chemotherapy (TC, 6 cycles)	AWOD	(Not available)
48	Hoang^30)^	2005	60	Rectum	Hematochezia	Endometrioid adenocarcinoma	En bloc resection of the upper rectum and distal ureter	Negative	(Not available)	(Not available)	(Not available)
49	Kawate^31)^	2005	62	Sigmoid	Abdominal tumor	Endometrioid adenocarcinoma	Sigmoidectomy with lymph node dissection	Positive (5/10)	Chemotherapy (Cyclophosphamide, Pirarubicin, Hydrochloride, Carboplatin)	AWOD	28
50	Efethymou^32)^	2009	59	Rectum	Constipation, Weight loss	Endometrioid adenocarcinoma	Anterior resection with diverting loop ileostomy	Negative	(Not available)	AWOD	(Not available)
51	Kobayashi^33)^	2010	45	Rectum	Periodic hematochezia with lower abdominal pain during her menstrual period	Endometrioid adenocarcinoma	Anterior resection	Positive (6/8)	Chemotherapy (TC)	AWOD	(Not available)
52	Palla^34)^	2017	75	Sigmoid	Abdominal pain, Enterorrhagia	Endometrioid adenocarcinoma	Sigmoidectomy	(Not available)	None	(Not available)	(Not available)
53	Hemedez^35)^	2018	58	Rectum	Recto-vaginal pain, Severe constipation	Endometrioid adenocarcinoma	Low Anterior resection	Negative	Chemotherapy	AWOD	(Not available)
54	Li^36)^	2018	55	Rectum	Abdominal pain, Enterorrhagia	Endometrioid adenocarcinoma	Anterior resection, hysterectomy and bilateral salpingo-oophorectomy	Positive (8/30)	Chemotherapy (TC, 8 cycles)	AWD	23
55	Yang^37)^	2019	57	Rectum	Left lower abdominal pain	Endometrioid adenocarcinoma	Radical hysterectomy; bilateral adnexectomy; pelvic peritonectomy; pelvic lymphadenectomy; omentectomy; partial rectal resection with diverting ileostomy; appendectomy	(Not available)	Chemotherapy	AWOD	12
56	Current case	2025	53	Rectum	Lower abdominal pain	Endometrioid adenocarcinoma	Low anterior resection, total hysterectomy, bilateral salpingo-oophorectomy, vaginal cuff resection, and bilateral lateral pelvic lymph node dissection	Negative	Chemotherapy (TC, 6 cycles)	AWOD	36

AWD, alive with disease; AWOD, alive without disease; BRBPR, bright red blood per rectum; BSO, bilateral salpingo-oophorectomy; DOC, died of other causes; DOD, died of disease; LLQ, left lower quadrant; TAH, total abdominal hysterectomy

Histologically, endometrioid adenocarcinoma was the predominant tumor type (n = 34), followed by carcinosarcoma (n = 3), Müllerian adenosarcoma (n = 3), endometrial stromal sarcoma (n = 2), spindle cell carcinoma (n = 1), squamous cell carcinoma (n = 1), adenoacanthoma (n = 1), adenosquamous carcinoma (n = 1), and clear cell carcinoma (n = 1). This histological diversity underscores the heterogeneous nature of EAITs.

Preoperative diagnosis remains challenging, largely owing to the submucosal or extrinsic growth pattern of these tumors. Conventional diagnostic modalities such as ultrasound, CT, and MRI have shown limited accuracy, with correct preoperative diagnosis reported in only 9%–22% of cases.^38,39)^ More recently, endoscopic ultrasound with fine-needle aspiration has emerged as a valuable tool for tissue diagnosis and should be considered early in the diagnostic work-up.^40,41)^

Immunohistochemistry also plays a critical role in differentiating EAITs from primary colorectal adenocarcinomas. While CK20 is commonly expressed in intestinal-type and Merkel cell carcinomas, CK7 positivity (in the absence of CK20) is typically seen in gynecological, breast, and lung adenocarcinomas.^42)^ Additionally, PAX8 and CDX2 staining provide further diagnostic guidance. PAX8 is a marker for Müllerian, renal, and thyroid tumors,^43)^ whereas CDX2 is a highly sensitive and specific marker for intestinal differentiation, particularly colorectal origin. Furthermore, vimentin and ER positivity supports an endometrioid phenotype. Therefore, the use of a panel of immunomarkers is recommended to distinguish EAITs from primary colorectal malignancies.

Surgical resection remains the mainstay of treatment. Most reported cases have undergone en bloc resection with concurrent or non-concurrent hysterectomy and oophorectomy, although no formal guidelines recommend this approach. Given the common dissemination pattern of endometriosis through the uterus and adnexa, combined gynecological resection is generally favored.^42)^

According to **Table 1**, 51 of 55 cases achieved R0/1 resection, and 94% of patients who survived for more than 1 year had undergone R0/1 surgery. In addition, lymph node metastasis appears to have a substantial impact on prognosis. Lymph node metastasis was identified in 12 of 38 cases for which lymph node status was available; recurrence occurred in 8 of these 12 patients in the lymph node-positive group, compared with 1 of 26 patients in the lymph node-negative group, indicating a markedly higher recurrence rate.

This finding is consistent with the report by Tanaka et al., who analyzed Japanese case reports and demonstrated that the 5-year overall survival rate was 90.9% in the lymph node-negative group and 41.7% in the lymph node-positive group.^44)^ Taken together, these findings suggest that achieving complete resection is a key determinant of favorable outcomes, whereas lymph node metastasis is associated with poor prognosis and a high risk of recurrence.

On this basis, we elected to perform posterior pelvic exenteration with bilateral pelvic lymph node dissection, including lateral pelvic nodes, with the aim of maximizing oncological clearance. Although this approach entails substantial surgical invasiveness, the patient was deemed fit for radical surgery. Furthermore, preoperative imaging demonstrated significant enlargement of the lateral pelvic lymph nodes, supporting the need for aggressive surgical clearance. In this context, the potential oncological benefit of complete tumor eradication was considered to outweigh the risks associated with the extensive procedure.

The role of adjuvant chemotherapy or radiation therapy in EAIT remains unclear. Among patients with lymph node-positive disease listed in **Table 1**, 7 of 12 received additional treatment, such as chemotherapy or radiation therapy. In most reported cases, chemotherapy regimens based on ovarian cancer protocols—most commonly paclitaxel and carboplatin—have been used empirically. Kawate et al. reported a favorable outcome using a regimen comprising cyclophosphamide, pirarubicin, and carboplatin in a patient with lymph node-positive disease;^31)^ however, the evidence remains anecdotal. Similarly, the role of radiation therapy is poorly defined, with no established evidence regarding optimal dose or irradiation field. Therefore, further investigation is warranted to establish optimal adjuvant treatment strategies and standardized protocols through collaborative efforts.

Finally, we conducted proteomic analysis of the tumor using mass spectrometry. We acknowledge the inherent limitations of single-case studies, including the inability to draw generalized conclusions or establish causality. Accordingly, the present analysis should be regarded as exploratory in nature. Rather than serving as definitive evidence, the proteomic dataset generated in this study is intended to function as a reference molecular profile for this rare disease context. By providing a detailed protein-level characterization, this work offers a foundation for future comparative analyses, enabling subsequent studies to assess reproducibility, identify shared molecular features, and refine biological hypotheses as additional cases become available. Integrating such datasets across multiple cases may enable the identification of diagnostic biomarkers or novel therapeutic targets in EAIT.

## CONCLUSIONS

We presented an extremely rare case of an EAIT. Although EAITs generally have a poor prognosis, our case suggests that aggressive surgical resection, including lymphadenectomy, followed by adjuvant chemotherapy may improve clinical outcomes. Further research is required to determine the optimal surgical approach, define the role of adjuvant therapies, and identify reliable prognostic indicators. Moreover, the proteomic data generated from this case, which are publicly available through a proteomic repository, provide a valuable resource for future comparative studies when integrated with additional cases.

## SUPPLEMENTARY MATERIALS

Supplementary Table 1 Results of comprehensive proteomic analysis of the tumor tissue using mass spectrometry.

Supplementary Methods Detailed methods are described in the Supplementary Appendix.

## DECLARATIONS

### Availability of data and materials

All data supporting the findings of this study are included in the article and its supplementary materials. The dataset is also publicly accessible in the jPOST repository under the Accession Nos. JPST004032 and PXD067723.
