# Supplementary material for: Endometriosis-Associated Intestinal Tumor: A Case Report and Literature Review with Comprehensive Proteomic Data
Source: Surg Case Rep. 2026 Mar 11;12(1):25-0761. doi: 10.70352/scrj.cr.25-0761 (PMC12991934; doi:10.70352/scrj.cr.25-0761)
Supplement: Supplementary Table 1 — Results of comprehensive proteomic analysis of the tumor tissue using mass spectrometry. [file scr-12-01-25-0761-s001.pdf]

Supplementary Table 1. Results of comprehensive proteomic analysis of the tumor tissue using mass spectrometry

Number	# of Grouped proteins	Accession number	Master accession number	Master protein name	Master Gene Symbol	Species	MW(kDa)	Peptides	Unique peptides	N_Cjo228_032	T_Cjo228_031
1	1	Q15149	Q15149	Plectin	PLEC	Homo sapiens	531.798	250	242	458959199	123132746
2	1	Q09666	Q09666	Neuroblast differentiation-associated protein AHNAK	AHNAK	Homo sapiens	629.112	198	198	1009683471	60571070
3	1	Q8NF91	Q8NF91	Nesprin-1	SYNE1	Homo sapiens	1011.107	195	193	16988918	30252402
4	1	Q9UPN3	Q9UPN3	Microtubule-actin cross-linking factor 1, isoforms 1/2/3/4/5	MACF1	Homo sapiens	838.323	194	189	37759533	15199722
5	1	Q14204	Q14204	Cytoplasmic dynein 1 heavy chain 1	DYNC1H1	Homo sapiens	532.418	187	187	213501456	78461509
6	1	P04114	P04114	Apolipoprotein B-100	APOB	Homo sapiens	515.615	171	171	165673017	248150820
7	1	Q13813	Q13813	Spectrin alpha chain, non-erythrocytic 1	SPTAN1	Homo sapiens	284.544	141	141	240554437	58965053
8	1	P98160	P98160	Basement membrane-specific heparan sulfate proteoglycan core protein	HSPG2	Homo sapiens	468.836	135	135	445386238	53961407
9	1	P12111	P12111	Collagen alpha-3(VI) chain	COL6A3	Homo sapiens	343.671	134	134	2272509238	299541516
10	1	Q8WXH0	Q8WXH0	Nesprin-2	SYNE2	Homo sapiens	796.458	134	134	15878440	10636368
11	1	P21333	P21333	Filamin-A	FLNA	Homo sapiens	280.742	140	130	8201166458	289364614
12	1	P11532	P11532	Dystrophin	DMD	Homo sapiens	426.757	127	125	61813750	0
13	1	Q03001	Q03001	Dystonin	DST	Homo sapiens	860.678	123	123	19873458	153081
14	1	P78527	P78527	DNA-dependent protein kinase catalytic subunit	PRKDC	Homo sapiens	469.099	121	121	53357188	42382920
15	1	Q14315	Q14315	Filamin-C	FLNC	Homo sapiens	291.025	125	121	1394684568	16801145
16	1	P15924	P15924	Desmoplakin	DSP	Homo sapiens	331.78	113	113	95181514	5099946
17	1	P35749	P35749	Myosin-11	MYH11	Homo sapiens	227.342	120	113	7612896131	3398814
18	1	Q99715	Q99715	Collagen alpha-1(XII) chain	COL12A1	Homo sapiens	333.151	112	112	115840514	109485728
19	1	Q01082	Q01082	Spectrin beta chain, non-erythrocytic 1	SPTBN1	Homo sapiens	274.614	122	111	210575259	47468593
20	1	Q8IVF2	Q8IVF2	Protein AHNAK2	AHNAK2	Homo sapiens	616.639	109	109	53377418	7313451
21	1	Q63HN8	Q63HN8	E3 ubiquitin-protein ligase RNF213	RNF213	Homo sapiens	591.42	104	104	10302386	19872661
22	1	P35579	P35579	Myosin-9	MYH9	Homo sapiens	226.536	126	103	399632256	565634900
23	1	O75369	O75369	Filamin-B	FLNB	Homo sapiens	278.167	99	99	148510704	42462058
24	1	P22105	P22105	Tenascin-X	TNXB	Homo sapiens	458.395	112	99	77858871	157200
25	1	Q14789	Q14789	Golgin subfamily B member 1	GOLGB1	Homo sapiens	376.027	98	98	16098022	4058526
26	1	Q9Y490	Q9Y490	Talin-1	TLN1	Homo sapiens	269.773	111	97	584933227	99635335
27	1	Q14980	Q14980	Nuclear mitotic apparatus protein 1	NUMA1	Homo sapiens	238.263	95	95	44398796	11167194
28	1	P02549	P02549	Spectrin alpha chain, erythrocytic 1	SPTA1	Homo sapiens	280.019	93	93	23065043	49693994
29	1	P46939	P46939	Utrophin	UTRN	Homo sapiens	394.474	92	92	27504713	3101054
30	1	Q7Z406	Q7Z406	Myosin-14	MYH14	Homo sapiens	227.875	94	92	77068573	9492330
31	1	O95425	O95425	Supervillin	SVIL	Homo sapiens	247.75	87	87	75376699	6090184
32	1	P01024	P01024	Complement C3	C3	Homo sapiens	187.152	91	85	503969106	1008022062
33	1	P58107	P58107	Epiplakin	EPPK1	Homo sapiens	555.667	85	85	45470045	13686512
34	1	P11277	P11277	Spectrin beta chain, erythrocytic	SPTB	Homo sapiens	246.47	84	84	20182120	42031734
35	1	O15230	O15230	Laminin subunit alpha-5	LAMA5	Homo sapiens	399.744	84	83	83652999	6308251
36	1	Q5T4S7	Q5T4S7	E3 ubiquitin-protein ligase UBR4	UBR4	Homo sapiens	573.853	82	82	16666636	14019450
37	1	P35555	P35555	Fibrillin-1	FBN1	Homo sapiens	312.307	83	81	146789946	43117629
38	1	Q7Z6Z7	Q7Z6Z7	E3 ubiquitin-protein ligase HUWE1	HUWE1	Homo sapiens	481.899	80	80	24247031	6814051
39	1	Q9NZM1	Q9NZM1	Myoferlin	MYOF	Homo sapiens	234.713	78	77	52579963	29860908
40	1	Q07954	Q07954	Prolow-density lipoprotein receptor-related protein 1	LRP1	Homo sapiens	504.617	75	75	28065451	6216337
41	1	Q709C8	Q709C8	Intermembrane lipid transfer protein VPS13C	VPS13C	Homo sapiens	422.398	73	73	21428331	11557866
42	1	O15061	O15061	Synemin	SYNM	Homo sapiens	172.868	71	71	532861903	911047
43	1	Q92616	Q92616	eIF-2-alpha kinase activator GCN1	GCN1	Homo sapiens	292.713	71	71	23756235	15689931
44	1	P49327	P49327	Fatty acid synthase	FASN	Homo sapiens	273.431	70	70	37386624	21828839
45	1	Q01484	Q01484	Ankyrin-2	ANK2	Homo sapiens	433.723	77	69	17891735	0

46	1	Q99996	Q99996	A-kinase anchor protein 9	AKAP9	Homo sapiens	452.996	69	69	7982167	777919
47	1	Q9Y6R7	Q9Y6R7	IgGFc-binding protein	FCGBP	Homo sapiens	572.028	68	68	341754477	4803738
48	1	P18206	P18206	Vinculin	VCL	Homo sapiens	123.801	67	67	1104142235	91140747
49	1	Q9Y4G6	Q9Y4G6	Talin-2	TLN2	Homo sapiens	271.62	67	67	26514986	938476
50	1	P24043	P24043	Laminin subunit alpha-2	LAMA2	Homo sapiens	343.911	65	65	15595183	0
51	1	Q92614	Q92614	Unconventional myosin-XVIIIa	MYO18A	Homo sapiens	233.116	65	65	10343627	12414300
52	1	Q9HBL0	Q9HBL0	Tensin-1	TNS1	Homo sapiens	197.024	67	65	213404656	2571546
53	1	P12270	P12270	Nucleoprotein TPR	TPR	Homo sapiens	267.298	63	63	17410474	7770680
54	1	P35580	P35580	Myosin-10	MYH10	Homo sapiens	229.002	63	63	21655604	12128757
55	1	O75165	O75165	DnaJ homolog subfamily C member 13	DNAJC13	Homo sapiens	254.422	62	62	11539220	23075743
56	1	O60437	O60437	Periplakin	PPL	Homo sapiens	204.749	61	61	10102226	4076220
57	1	Q8NDA2	Q8NDA2	Hemicentin-2	HMCN2	Homo sapiens	541.985	60	60	11438317	0
58	1	P55268	P55268	Laminin subunit beta-2	LAMB2	Homo sapiens	195.985	60	59	103442217	4386069
59	1	O75643	O75643	U5 small nuclear ribonucleoprotein 200 kDa helicase	SNRNP200	Homo sapiens	244.511	58	58	22680184	9553555
60	1	Q02817	Q02817	Mucin-2	MUC2	Homo sapiens	550.866	58	58	355819807	1556664
61	1	O75923	O75923	Dysferlin	DYSF	Homo sapiens	237.298	57	57	14570384	51913030
62	1	Q13439	Q13439	Golgin subfamily A member 4	GOLGA4	Homo sapiens	261.144	57	57	7057983	2743844
63	1	Q15075	Q15075	Early endosome antigen 1	EEA1	Homo sapiens	162.468	57	57	26885661	9118316
64	1	Q6P2Q9	Q6P2Q9	Pre-mRNA-processing-splicing factor 8	PRPF8	Homo sapiens	273.605	56	56	22706406	9936138
65	1	O75592	O75592	E3 ubiquitin-protein ligase MYCBP2	MYCBP2	Homo sapiens	513.646	55	55	4153773	3240570
66	1	P24821	P24821	Tenascin	TNC	Homo sapiens	240.857	55	55	229766640	57783780
67	1	P46940	P46940	Ras GTPase-activating-like protein IQGAP1	IQGAP1	Homo sapiens	189.254	57	55	149974624	93908978
68	1	Q6UVK1	Q6UVK1	Chondroitin sulfate proteoglycan 4	CSPG4	Homo sapiens	250.54	55	55	45689107	3953244
69	1	P02545	P02545	Prelamin-A/C	LMNA	Homo sapiens	74.141	54	54	584600055	55847712
70	1	P16157	P16157	Ankyrin-1	ANK1	Homo sapiens	206.27	56	54	15310532	68555313
71	1	P50851	P50851	Lipopolysaccharide-responsive and beige-like anchor protein	LRBA	Homo sapiens	319.112	59	54	14635155	4605005
72	1	Q05707	Q05707	Collagen alpha-1(XIV) chain	COL14A1	Homo sapiens	193.519	54	54	406086726	10936140
73	1	Q00610	Q00610	Clathrin heavy chain 1	CLTC	Homo sapiens	191.617	69	53	148457751	88664253
74	1	Q02952	Q02952	A-kinase anchor protein 12	AKAP12	Homo sapiens	191.487	53	53	58948984	633885
75	1	Q14008	Q14008	Cytoskeleton-associated protein 5	CKAP5	Homo sapiens	225.499	53	53	15581028	6281991
76	1	Q86UP2	Q86UP2	Kinectin	KTN1	Homo sapiens	156.279	53	53	29255672	6797814
77	1	P04275	P04275	von Willebrand factor	VWF	Homo sapiens	309.27	52	52	19638259	14051258
78	1	Q14683	Q14683	Structural maintenance of chromosomes protein 1A	SMC1A	Homo sapiens	143.236	52	52	15979605	10571164
79	1	Q16787	Q16787	Laminin subunit alpha-3	LAMA3	Homo sapiens	366.629	52	52	2250984	26803731
80	1	P35573	P35573	Glycogen debranching enzyme	AGL	Homo sapiens	174.768	51	51	18688928	11093215
81	1	A3KMH1	A3KMH1	von Willebrand factor A domain-containing protein 8	VWA8	Homo sapiens	214.825	50	50	9221221	1301200
82	1	A5YKK6	A5YKK6	CCR4-NOT transcription complex subunit 1	CNOT1	Homo sapiens	266.945	50	50	10394151	4692362
83	1	P16144	P16144	Integrin beta-4	ITGB4	Homo sapiens	202.17	50	50	28485541	5219484
84	1	Q14203	Q14203	Dynactin subunit 1	DCTN1	Homo sapiens	141.696	50	50	28880575	11445281
85	1	P01023	P01023	Alpha-2-macroglobulin	A2M	Homo sapiens	163.292	56	49	151242154	308462028
86	1	P46821	P46821	Microtubule-associated protein 1B	MAP1B	Homo sapiens	270.639	50	49	21234894	5077704
87	1	P53621	P53621	Coatomer subunit alpha	COPA	Homo sapiens	138.349	49	49	57633738	39821819
88	1	Q9UQE7	Q9UQE7	Structural maintenance of chromosomes protein 3	SMC3	Homo sapiens	141.543	49	49	13164482	9668159
89	1	P07814	P07814	Bifunctional glutamate/proline--tRNA ligase	EPRS1	Homo sapiens	170.594	48	48	51041619	20726820
90	1	P55072	P55072	Transitional endoplasmic reticulum ATPase	VCP	Homo sapiens	89.322	48	48	91441973	52225664
91	1	Q07157	Q07157	Tight junction protein ZO-1	TJP1	Homo sapiens	195.461	48	48	19583282	3962411
92	1	Q13576	Q13576	Ras GTPase-activating-like protein IQGAP2	IQGAP2	Homo sapiens	180.581	48	48	28200571	18744398
93	1	Q16363	Q16363	Laminin subunit alpha-4	LAMA4	Homo sapiens	202.525	48	48	89188128	11012416

94	1	Q9C0C2	Q9C0C2	182 kDa tankyrase-1-binding protein	TNKS1BP1	Homo sapiens	181.799	48	48	19485173	2643828
95	1	P02788	P02788	Lactotransferrin	LTF	Homo sapiens	78.183	49	47	29272530	1588777400
96	1	P55196	P55196	Afadin	AFDN	Homo sapiens	206.808	47	47	11659604	734040
97	1	Q13464	Q13464	Rho-associated protein kinase 1	ROCK1	Homo sapiens	158.178	54	47	13844544	13840916
98	1	Q5S007	Q5S007	Leucine-rich repeat serine/threonine-protein kinase 2	LRRK2	Homo sapiens	286.109	47	47	70875	11861364
99	1	Q5T5P2	Q5T5P2	Sickle tail protein homolog	KIAA1217	Homo sapiens	214.12	47	47	8725801	354854
100	1	O43149	O43149	Zinc finger ZZ-type and EF-hand domain-containing protein 1	ZZEF1	Homo sapiens	331.083	46	46	6591605	2373041
101	1	O94832	O94832	Unconventional myosin-Id	MYO1D	Homo sapiens	116.205	48	46	50787649	1071075
102	1	P11047	P11047	Laminin subunit gamma-1	LAMC1	Homo sapiens	177.608	47	46	121435139	21308095
103	1	P42704	P42704	Leucine-rich PPR motif-containing protein, mitochondrial	LRPPRC	Homo sapiens	157.907	46	46	65973653	11553172
104	1	P78559	P78559	Microtubule-associated protein 1A	MAP1A	Homo sapiens	305.49	46	46	15884593	1257678
105	1	Q08378	Q08378	Golgin subfamily A member 3	GOLGA3	Homo sapiens	167.355	46	46	10029013	3842845
106	1	Q14152	Q14152	Eukaryotic translation initiation factor 3 subunit A	EIF3A	Homo sapiens	166.569	46	46	27109781	9669870
107	1	Q92608	Q92608	Dedicator of cytokinesis protein 2	DOCK2	Homo sapiens	211.95	46	46	4235397	22947067
108	1	O95359	O95359	Transforming acidic coiled-coil-containing protein 2	TACC2	Homo sapiens	309.432	46	45	7594637	0
109	1	P27708	P27708	CAD protein	CAD	Homo sapiens	242.985	46	45	11135656	10891226
110	1	Q15746	Q15746	Myosin light chain kinase, smooth muscle	MYLK	Homo sapiens	210.717	45	45	590266503	5064619
111	1	Q8WUY3	Q8WUY3	Protein prune homolog 2	PRUNE2	Homo sapiens	340.642	45	45	31348563	110180
112	1	Q9NQT8	Q9NQT8	Kinesin-like protein KIF13B	KIF13B	Homo sapiens	202.792	50	45	13264660	5329655
113	1	Q9P2E9	Q9P2E9	Ribosome-binding protein 1	RRBP1	Homo sapiens	152.457	46	45	79438565	22787942
114	1	P48681	P48681	Nestin	NES	Homo sapiens	177.44	44	44	28205851	17901677
115	1	Q14112	Q14112	Nidogen-2	NID2	Homo sapiens	151.256	45	44	45280800	17147778
116	1	Q96Q06	Q96Q06	Perilipin-4	PLIN4	Homo sapiens	135.894	44	44	74013066	0
117	1	Q9BX66	Q9BX66	Sorbin and SH3 domain-containing protein 1	SORBS1	Homo sapiens	142.514	45	44	229684854	594332
118	1	Q9P2M7	Q9P2M7	Cingulin	CGN	Homo sapiens	137.056	44	44	15681151	147251
119	1	A6NC98	A6NC98	Coiled-coil domain-containing protein 88B	CCDC88B	Homo sapiens	164.81	44	43	178181	10493028
120	1	P08603	P08603	Complement factor H	CFH	Homo sapiens	139.097	49	43	48161930	131620106
121	1	P14543	P14543	Nidogen-1	NID1	Homo sapiens	136.379	43	43	116370226	9358345
122	1	P49792	P49792	E3 SUMO-protein ligase RanBP2	RANBP2	Homo sapiens	358.206	67	43	9999478	8927711
123	1	Q14764	Q14764	Major vault protein	MVP	Homo sapiens	99.328	43	43	101194201	66123043
124	1	Q15643	Q15643	Thyroid receptor-interacting protein 11	TRIP11	Homo sapiens	227.591	43	43	3912150	2013844
125	1	O00159	O00159	Unconventional myosin-Ic	MYO1C	Homo sapiens	121.682	42	42	121469285	5196627
126	1	O43491	O43491	Band 4.1-like protein 2	EPB41L2	Homo sapiens	112.588	45	42	56106875	3377538
127	1	O75116	O75116	Rho-associated protein kinase 2	ROCK2	Homo sapiens	160.903	42	42	21051456	5822916
128	1	P01031	P01031	Complement C5	C5	Homo sapiens	188.308	42	42	17642542	117458538
129	1	P02768	P02768	Albumin	ALB	Homo sapiens	69.365	46	42	8496965701	11801061704
130	1	P08133	P08133	Annexin A6	ANXA6	Homo sapiens	75.875	42	42	336267508	172349958
131	1	P30622	P30622	CAP-Gly domain-containing linker protein 1	CLIP1	Homo sapiens	162.249	45	42	21530235	5847287
132	1	Q13459	Q13459	Unconventional myosin-IXb	MYO9B	Homo sapiens	243.405	43	42	4193015	7047015
133	1	Q96RL7	Q96RL7	Intermembrane lipid transfer protein VPS13A	VPS13A	Homo sapiens	360.285	42	42	4388815	2185225
134	1	O75533	O75533	Splicing factor 3B subunit 1	SF3B1	Homo sapiens	145.831	41	41	16419724	5907333
135	1	P02787	P02787	Serotransferrin	TF	Homo sapiens	77.05	41	41	423718834	694174042
136	1	P17661	P17661	Desmin	DES	Homo sapiens	53.536	41	41	7011175616	6241670
137	1	Q05682	Q05682	Caldesmon	CALD1	Homo sapiens	93.231	41	41	900513398	18430030
138	1	O15027	O15027	Protein transport protein Sec16A	SEC16A	Homo sapiens	251.897	40	40	7444702	3851306
139	1	P00450	P00450	Ceruloplasmin	CP	Homo sapiens	122.207	40	40	151209045	446008720
140	1	P02671	P02671	Fibrinogen alpha chain	FGA	Homo sapiens	94.975	41	40	542255751	29053968219
141	1	P42345	P42345	Serine/threonine-protein kinase mTOR	MTOR	Homo sapiens	288.896	40	40	6075980	2821539

142	1	Q00341	Q00341	Vigilin	HDLBP	Homo sapiens	141.457	40	40	26413813	11723115
143	1	Q14185	Q14185	Dedicator of cytokinesis protein 1	DOCK1	Homo sapiens	215.349	40	40	5922091	116987
144	1	Q5VYK3	Q5VYK3	Proteasome adapter and scaffold protein ECM29	ECPAS	Homo sapiens	204.294	40	40	12482235	6155103
145	1	Q6PIF6	Q6PIF6	Unconventional myosin-VIIb	MYO7B	Homo sapiens	241.601	42	40	8519444	665086
146	1	Q9Y4A5	Q9Y4A5	Transformation/transcription domain-associated protein	TRRAP	Homo sapiens	437.61	40	40	2751467	1052527
147	1	O43707	O43707	Alpha-actinin-4	ACTN4	Homo sapiens	104.855	61	39	181375013	55988582
148	1	O60716	O60716	Catenin delta-1	CTNND1	Homo sapiens	108.171	39	39	40084171	4868112
149	1	P07942	P07942	Laminin subunit beta-1	LAMB1	Homo sapiens	198.042	39	39	54350632	11011153
150	1	P11215	P11215	Integrin alpha-M	ITGAM	Homo sapiens	127.182	41	39	4002274	160596452
151	1	P42858	P42858	Huntingtin	HTT	Homo sapiens	347.607	39	39	3666794	1231096
152	1	Q08211	Q08211	ATP-dependent RNA helicase A	DHX9	Homo sapiens	140.961	39	39	32239077	15028934
153	1	Q13315	Q13315	Serine-protein kinase ATM	ATM	Homo sapiens	350.694	39	39	2428392	2891408
154	1	Q9BQS8	Q9BQS8	FYVE and coiled-coil domain-containing protein 1	FYCO1	Homo sapiens	166.984	39	39	9007927	891452
155	1	Q9H7D0	Q9H7D0	Dedicator of cytokinesis protein 5	DOCK5	Homo sapiens	215.312	40	39	3374553	4638203
156	1	A6NHR9	A6NHR9	Structural maintenance of chromosomes flexible hinge domain-containing protein 1	SMCHD1	Homo sapiens	226.377	38	38	4783384	10386562
157	1	P00747	P00747	Plasminogen	PLG	Homo sapiens	90.569	46	38	29431272	569099792
158	1	P10586	P10586	Receptor-type tyrosine-protein phosphatase F	PTPRF	Homo sapiens	212.882	43	38	7273114	188755
159	1	P12259	P12259	Coagulation factor V	F5	Homo sapiens	251.709	48	38	1064249	41975685
160	1	P46013	P46013	Proliferation marker protein Ki-67	MKI67	Homo sapiens	358.699	38	38	3446202	525291
161	1	P53618	P53618	Coatomer subunit beta	COPB1	Homo sapiens	107.143	38	38	27551559	16136373
162	1	Q14643	Q14643	Inositol 1,4,5-trisphosphate receptor type 1	ITPR1	Homo sapiens	313.933	44	38	7123330	718953
163	1	Q8TCU6	Q8TCU6	Phosphatidylinositol 3,4,5-trisphosphate-dependent Rac exchanger 1 protein	PREX1	Homo sapiens	186.204	38	38	1410726	27615199
164	1	Q8WX93	Q8WX93	Palladin	PALLD	Homo sapiens	150.567	39	38	220707557	5662696
165	1	Q92673	Q92673	Sortilin-related receptor	SORL1	Homo sapiens	248.432	38	38	3986788	9166144
166	1	Q9Y5S2	Q9Y5S2	Serine/threonine-protein kinase MRCK beta	CDC42BPB	Homo sapiens	194.317	42	38	7020213	1905974
167	1	Q9Y6C2	Q9Y6C2	EMILIN-1	EMILIN1	Homo sapiens	106.696	38	38	112677098	8448397
168	1	O43451	O43451	Maltase-glucoamylase	MGAM	Homo sapiens	312.027	40	37	6085	25347948
169	1	P08575	P08575	Receptor-type tyrosine-protein phosphatase C	PTPRC	Homo sapiens	147.488	37	37	8658911	70303512
170	1	P08670	P08670	Vimentin	VIM	Homo sapiens	53.652	45	37	1175138955	497613118
171	1	P13639	P13639	Elongation factor 2	EEF2	Homo sapiens	95.342	37	37	170473843	98457195
172	1	P16885	P16885	1-phosphatidylinositol 4,5-bisphosphate phosphodiesterase gamma-2	PLCG2	Homo sapiens	147.873	37	37	3725630	12920023
173	1	P22897	P22897	Macrophage mannose receptor 1	MRC1	Homo sapiens	166.013	37	37	14705715	5944701
174	1	P41252	P41252	Isoleucine--tRNA ligase, cytoplasmic	IARS1	Homo sapiens	144.498	37	37	16055813	13722132
175	1	P53814	P53814	Smoothelin	SMTN	Homo sapiens	99.059	37	37	193127585	184829
176	1	Q02880	Q02880	DNA topoisomerase 2-beta	TOP2B	Homo sapiens	183.272	45	37	9664326	4968463
177	1	Q03252	Q03252	Lamin-B2	LMNB2	Homo sapiens	69.949	37	37	41875894	6606009
178	1	Q13085	Q13085	Acetyl-CoA carboxylase 1	ACACA	Homo sapiens	265.558	47	37	2992392	3002682
179	1	Q14573	Q14573	Inositol 1,4,5-trisphosphate receptor type 3	ITPR3	Homo sapiens	304.115	38	37	5359775	3210901
180	1	Q14697	Q14697	Neutral alpha-glucosidase AB	GANAB	Homo sapiens	106.874	37	37	68941695	34048954
181	1	Q6ZNJ1	Q6ZNJ1	Neurobeachin-like protein 2	NBEAL2	Homo sapiens	302.521	37	37	2484028	19222370
182	1	Q7KZF4	Q7KZF4	Staphylococcal nuclease domain-containing protein 1	SND1	Homo sapiens	101.999	37	37	50774327	33653265
183	1	Q8IWJ2	Q8IWJ2	GRIP and coiled-coil domain-containing protein 2	GCC2	Homo sapiens	195.912	37	37	4214130	1375585
184	1	Q9UMS6	Q9UMS6	Synaptopodin-2	SYNPO2	Homo sapiens	117.516	37	37	342416576	64744
185	1	O94915	O94915	Protein furry homolog-like	FRYL	Homo sapiens	339.603	38	36	4349320	1863332
186	1	P11717	P11717	Cation-independent mannose-6-phosphate receptor	IGF2R	Homo sapiens	274.381	42	36	4429278	5742370
187	1	P12109	P12109	Collagen alpha-1(VI) chain	COL6A1	Homo sapiens	108.529	36	36	569232164	85051713

188	1	P20700	P20700	Lamin-B1	LMNB1	Homo sapiens	66.409	38	36	57873668	60167823
189	1	P29144	P29144	Tripeptidyl-peptidase 2	TPP2	Homo sapiens	138.352	36	36	14324471	9035816
190	1	Q0VD83	Q0VD83	Apolipoprotein B receptor	APOBR	Homo sapiens	115.634	36	36	14240263	11862097
191	1	Q14839	Q14839	Chromodomain-helicase-DNA-binding protein 4	CHD4	Homo sapiens	218.008	47	36	7565194	3097435
192	1	Q460N5	Q460N5	Protein mono-ADP-ribosyltransferase PARP14	PARP14	Homo sapiens	202.803	36	36	3608429	7010066
193	1	Q8N3D4	Q8N3D4	EH domain-binding protein 1-like protein 1	EHBP1L1	Homo sapiens	161.858	37	36	12948226	3715904
194	1	Q9H3S7	Q9H3S7	Tyrosine-protein phosphatase non-receptor type 23	PTPN23	Homo sapiens	178.974	36	36	7402742	2658538
195	1	Q9NY15	Q9NY15	Stabilin-1	STAB1	Homo sapiens	275.487	36	36	8571610	434835
196	1	Q9NYU2	Q9NYU2	UDP-glucose:glycoprotein glucosyltransferase 1	UGGT1	Homo sapiens	177.192	37	36	22689509	16501589
197	1	Q9NZB2	Q9NZB2	Constitutive coactivator of PPAR-gamma-like protein 1	FAM120A	Homo sapiens	121.888	39	36	20985064	5785440
198	1	Q9UM54	Q9UM54	Unconventional myosin-VI	MYO6	Homo sapiens	149.694	36	36	11086734	5420100
199	1	P05164	P05164	Myeloperoxidase	MPO	Homo sapiens	83.872	41	35	24358176	927507538
200	1	P11216	P11216	Glycogen phosphorylase, brain form	PYGB	Homo sapiens	96.699	43	35	66896371	5864574
201	1	Q13751	Q13751	Laminin subunit beta-3	LAMB3	Homo sapiens	129.572	35	35	1371547	29466263
202	1	Q13753	Q13753	Laminin subunit gamma-2	LAMC2	Homo sapiens	130.977	35	35	1317123	29785850
203	1	Q8NF50	Q8NF50	Dedicator of cytokinesis protein 8	DOCK8	Homo sapiens	238.534	36	35	1428040	11617781
204	1	Q92900	Q92900	Regulator of nonsense transcripts 1	UPF1	Homo sapiens	124.346	35	35	18183162	8699495
205	1	Q9BZF9	Q9BZF9	Uveal autoantigen with coiled-coil domains and ankyrin repeats	UACA	Homo sapiens	162.509	35	35	4832873	195437
206	1	Q9H2G2	Q9H2G2	STE20-like serine/threonine-protein kinase	SLK	Homo sapiens	142.697	35	35	14089137	7008206
207	1	Q9NR99	Q9NR99	Matrix-remodeling-associated protein 5	MXRA5	Homo sapiens	312.157	35	35	3190060	5374175
208	1	Q9P2J5	Q9P2J5	Leucine--tRNA ligase, cytoplasmic	LARS1	Homo sapiens	134.468	35	35	16756515	10928736
209	1	Q9Y3R5	Q9Y3R5	Protein dopey-2	DOP1B	Homo sapiens	258.235	36	35	3704083	1658221
210	1	Q9Y3Z3	Q9Y3Z3	Deoxynucleoside triphosphate triphosphohydrolase SAMHD1	SAMHD1	Homo sapiens	72.203	35	35	35489947	22333931
211	1	B0I1T2	B0I1T2	Unconventional myosin-Ig	MYO1G	Homo sapiens	116.444	34	34	2897500	20107477
212	1	O60313	O60313	Dynamin-like 120 kDa protein, mitochondrial	OPA1	Homo sapiens	111.631	34	34	16979272	4235537
213	1	P02751	P02751	Fibronectin	FN1	Homo sapiens	272.323	75	34	158618137	289928259
214	1	P05107	P05107	Integrin beta-2	ITGB2	Homo sapiens	84.785	34	34	4896484	152865429
215	1	P05165	P05165	Propionyl-CoA carboxylase alpha chain, mitochondrial	PCCA	Homo sapiens	80.06	34	34	30656875	2262776
216	1	P09874	P09874	Poly [ADP-ribose] polymerase 1	PARP1	Homo sapiens	113.086	34	34	38963787	7985585
217	1	P11586	P11586	C-1-tetrahydrofolate synthase, cytoplasmic	MTHFD1	Homo sapiens	101.531	35	34	48769577	35788094
218	1	P13667	P13667	Protein disulfide-isomerase A4	PDIA4	Homo sapiens	72.934	35	34	126467378	67559138
219	1	P52948	P52948	Nuclear pore complex protein Nup98-Nup96	NUP98	Homo sapiens	197.583	34	34	7159647	3717170
220	1	P53396	P53396	ATP-citrate synthase	ACLY	Homo sapiens	120.838	34	34	16267396	17437163
221	1	Q15393	Q15393	Splicing factor 3B subunit 3	SF3B3	Homo sapiens	135.578	34	34	20055640	12226100
222	1	Q16531	Q16531	DNA damage-binding protein 1	DDB1	Homo sapiens	126.97	34	34	21893628	9244159
223	1	Q5JRA6	Q5JRA6	Transport and Golgi organization protein 1 homolog	MIA3	Homo sapiens	213.707	34	34	6981380	2709342
224	1	Q86VP6	Q86VP6	Cullin-associated NEDD8-dissociated protein 1	CAND1	Homo sapiens	136.377	35	34	40533965	13378670
225	1	Q9H2D6	Q9H2D6	TRIO and F-actin-binding protein	TRIOBP	Homo sapiens	261.381	35	34	19069336	1594586
226	1	A0AVT1	A0AVT1	Ubiquitin-like modifier-activating enzyme 6	UBA6	Homo sapiens	117.972	33	33	11400155	14139798
227	1	A2RRP1	A2RRP1	NBAS subunit of NRZ tethering complex	NBAS	Homo sapiens	268.575	33	33	5203783	1199043
228	1	O14980	O14980	Exportin-1	XPO1	Homo sapiens	123.387	33	33	14111463	6079294
229	1	O75339	O75339	Cartilage intermediate layer protein 1	CILP	Homo sapiens	132.566	33	33	15305918	58531
230	1	P12814	P12814	Alpha-actinin-1	ACTN1	Homo sapiens	103.062	37	33	391903349	72920945
231	1	Q02218	Q02218	2-oxoglutarate dehydrogenase complex component E1	OGDH	Homo sapiens	115.937	42	33	36448040	5381680
232	1	Q13200	Q13200	26S proteasome non-ATPase regulatory subunit 2	PSMD2	Homo sapiens	100.201	33	33	23383081	17744333
233	1	Q14BN4	Q14BN4	Sarcolemmal membrane-associated protein	SLMAP	Homo sapiens	95.199	33	33	116160362	2416669
234	1	Q5VZ89	Q5VZ89	DENN domain-containing protein 4C	DENND4C	Homo sapiens	212.714	33	33	5815442	1101054
235	1	Q63ZY3	Q63ZY3	KN motif and ankyrin repeat domain-containing protein 2	KANK2	Homo sapiens	91.175	34	33	58567949	241821

236	1	Q68DQ2	Q68DQ2	Very large A-kinase anchor protein	CRYBG3	Homo sapiens	330.641	33	33	2989263	0
237	1	Q7KZ85	Q7KZ85	Transcription elongation factor SPT6	SUPT6H	Homo sapiens	199.076	33	33	3595989	1218973
238	1	Q8WWI1	Q8WWI1	LIM domain only protein 7	LMO7	Homo sapiens	192.702	33	33	6033780	174830
239	1	Q93008	Q93008	Probable ubiquitin carboxyl-terminal hydrolase FAF-X	USP9X	Homo sapiens	290.467	55	33	11289088	7371360
240	1	Q96N67	Q96N67	Dedicator of cytokinesis protein 7	DOCK7	Homo sapiens	242.565	35	33	4413611	300556
241	1	Q9P0K7	Q9P0K7	Ankycorbin	RAI14	Homo sapiens	110.041	33	33	11654828	2472965
242	1	Q9UBC5	Q9UBC5	Unconventional myosin-Ia	MYO1A	Homo sapiens	118.402	33	33	10568326	51513
243	1	Q9ULV0	Q9ULV0	Unconventional myosin-Vb	MYO5B	Homo sapiens	213.675	37	33	9118420	435812
244	1	Q9Y4I1	Q9Y4I1	Unconventional myosin-Va	MYO5A	Homo sapiens	215.409	33	33	7863511	3104142
245	1	P22102	P22102	Trifunctional purine biosynthetic protein adenosine-3	GART	Homo sapiens	107.766	32	32	13391933	9857628
246	1	P26640	P26640	Valine--tRNA ligase	VARS1	Homo sapiens	140.479	32	32	22847485	10757139
247	1	P27816	P27816	Microtubule-associated protein 4	MAP4	Homo sapiens	121.007	32	32	106135476	9625931
248	1	P33176	P33176	Kinesin-1 heavy chain	KIF5B	Homo sapiens	109.688	43	32	38217378	10087684
249	1	P34932	P34932	Heat shock 70 kDa protein 4	HSPA4	Homo sapiens	94.333	34	32	30618747	20106543
250	1	P54136	P54136	Arginine--tRNA ligase, cytoplasmic	RARS1	Homo sapiens	75.378	32	32	20317284	11823489
251	1	Q12802	Q12802	A-kinase anchor protein 13	AKAP13	Homo sapiens	307.555	32	32	3448776	3213445
252	1	Q14690	Q14690	Protein RRP5 homolog	PDCD11	Homo sapiens	208.704	32	32	3381173	982643
253	1	Q15124	Q15124	Phosphoglucomutase-like protein 5	PGM5	Homo sapiens	62.225	32	32	231418450	0
254	1	Q15772	Q15772	Striated muscle preferentially expressed protein kinase	SPEG	Homo sapiens	354.295	32	32	5113246	0
255	1	Q16891	Q16891	MICOS complex subunit MIC60	IMMT	Homo sapiens	83.679	32	32	43789623	13234394
256	1	Q6ZS81	Q6ZS81	WD repeat- and FYVE domain-containing protein 4	WDFY4	Homo sapiens	353.616	32	32	507863	2992556
257	1	Q8IZQ1	Q8IZQ1	WD repeat and FYVE domain-containing protein 3	WDFY3	Homo sapiens	395.267	32	32	1236144	3031367
258	1	Q92626	Q92626	Peroxidasin homolog	PXDN	Homo sapiens	165.278	32	32	4545214	9118391
259	1	Q9HC84	Q9HC84	Mucin-5B	MUC5B	Homo sapiens	596.355	34	32	7119012	0
260	1	Q9NZ08	Q9NZ08	Endoplasmic reticulum aminopeptidase 1	ERAP1	Homo sapiens	107.238	32	32	25018357	11543705
261	1	Q9Y4E8	Q9Y4E8	Ubiquitin carboxyl-terminal hydrolase 15	USP15	Homo sapiens	112.422	34	32	6249537	5056971
262	1	O14974	O14974	Protein phosphatase 1 regulatory subunit 12A	PPP1R12A	Homo sapiens	115.284	31	31	56794971	8300885
263	1	O60610	O60610	Protein diaphanous homolog 1	DIAPH1	Homo sapiens	141.35	31	31	7714316	9763847
264	1	P02675	P02675	Fibrinogen beta chain	FGB	Homo sapiens	55.931	35	31	223342316	14295763505
265	1	P04843	P04843	Dolichyl-diphosphooligosaccharide--protein glycosyltransferase subunit 1	RPN1	Homo sapiens	68.57	31	31	59268116	48421551
266	1	P09327	P09327	Villin-1	VIL1	Homo sapiens	92.695	32	31	76720864	1302664
267	1	P11021	P11021	Endoplasmic reticulum chaperone BiP	HSPA5	Homo sapiens	72.331	32	31	223737676	174445253
268	1	P12956	P12956	X-ray repair cross-complementing protein 6	XRCC6	Homo sapiens	69.844	31	31	63101120	36521887
269	1	P14618	P14618	Pyruvate kinase PKM	PKM	Homo sapiens	57.938	32	31	209643201	350380470
270	1	P22314	P22314	Ubiquitin-like modifier-activating enzyme 1	UBA1	Homo sapiens	117.849	31	31	68834920	47387234
271	1	P41219	P41219	Peripherin	PRPH	Homo sapiens	53.653	31	31	79573740	0
272	1	Q12955	Q12955	Ankyrin-3	ANK3	Homo sapiens	480.418	31	31	12253468	0
273	1	Q13045	Q13045	Protein flightless-1 homolog	FLII	Homo sapiens	144.753	31	31	14730053	16589082
274	1	Q13618	Q13618	Cullin-3	CUL3	Homo sapiens	88.931	31	31	10541064	4070172
275	1	Q14126	Q14126	Desmoglein-2	DSG2	Homo sapiens	122.295	31	31	19154333	2490576
276	1	Q14669	Q14669	E3 ubiquitin-protein ligase TRIP12	TRIP12	Homo sapiens	220.438	31	31	2922794	2089373
277	1	Q5JRX3	Q5JRX3	Presequence protease, mitochondrial	PITRM1	Homo sapiens	117.415	31	31	9157348	2801510
278	1	Q5JSL3	Q5JSL3	Dedicator of cytokinesis protein 11	DOCK11	Homo sapiens	237.675	33	31	3306955	5618266
279	1	Q6PGP7	Q6PGP7	SKI3 subunit of superkiller complex protein	SKIC3	Homo sapiens	175.491	31	31	11058857	3537145
280	1	Q86UU1	Q86UU1	Pleckstrin homology-like domain family B member 1	PHLDB1	Homo sapiens	151.165	31	31	7537165	301940
281	1	Q8IY21	Q8IY21	Probable ATP-dependent RNA helicase DDX60	DDX60	Homo sapiens	197.857	33	31	7623107	2145061
282	1	Q8N1F7	Q8N1F7	Nuclear pore complex protein Nup93	NUP93	Homo sapiens	93.489	31	31	11656111	9636526

283	1	Q8TDJ6	Q8TDJ6	DmX-like protein 2	DMXL2	Homo sapiens	339.646	31	31	2160239	1682805
284	1	Q8WYP5	Q8WYP5	Protein ELYS	AHCTF1	Homo sapiens	252.503	31	31	3696612	1146929
285	1	Q92538	Q92538	Golgi-specific brefeldin A-resistance guanine nucleotide exchange factor 1	GBF1	Homo sapiens	206.45	31	31	5007339	1488120
286	1	Q93009	Q93009	Ubiquitin carboxyl-terminal hydrolase 7	USP7	Homo sapiens	128.303	31	31	14461273	6792837
287	1	Q99798	Q99798	Aconitate hydratase, mitochondrial	ACO2	Homo sapiens	85.426	31	31	152680733	28429844
288	1	Q9BSJ8	Q9BSJ8	Extended synaptotagmin-1	ESYT1	Homo sapiens	122.857	31	31	26224927	6308674
289	1	Q9NR09	Q9NR09	Baculoviral IAP repeat-containing protein 6	BIRC6	Homo sapiens	530.279	31	31	4022680	1112078
290	1	Q9P265	Q9P265	Disco-interacting protein 2 homolog B	DIP2B	Homo sapiens	171.492	32	31	4469263	1837637
291	1	Q9UKK3	Q9UKK3	Protein mono-ADP-ribosyltransferase PARP4	PARP4	Homo sapiens	192.598	31	31	11212953	7387595
292	1	Q9Y4L1	Q9Y4L1	Hypoxia up-regulated protein 1	HYOU1	Homo sapiens	111.34	31	31	29433420	22874681
293	1	O00468	O00468	Agrin	AGRN	Homo sapiens	217.321	30	30	10706695	201018904
294	1	O60271	O60271	C-Jun-amino-terminal kinase-interacting protein 4	SPAG9	Homo sapiens	146.207	34	30	10264430	2296067
295	1	O60841	O60841	Eukaryotic translation initiation factor 5B	EIF5B	Homo sapiens	138.829	30	30	10493909	4498856
296	1	P12110	P12110	Collagen alpha-2(VI) chain	COL6A2	Homo sapiens	108.582	30	30	495486587	71225559
297	1	P28331	P28331	NADH-ubiquinone oxidoreductase 75 kDa subunit, mitochondrial	NDUFS1	Homo sapiens	79.469	30	30	42442204	7094386
298	1	P47897	P47897	Glutamine--tRNA ligase	QARS1	Homo sapiens	87.799	30	30	22070083	10488587
299	1	P49588	P49588	Alanine--tRNA ligase, cytoplasmic	AARS1	Homo sapiens	106.813	30	30	23008214	13137942
300	1	P49589	P49589	Cysteine--tRNA ligase, cytoplasmic	CARS1	Homo sapiens	85.474	30	30	15316268	5917572
301	1	P50990	P50990	T-complex protein 1 subunit theta	CCT8	Homo sapiens	59.619	30	30	78089262	51467078
302	1	P98171	P98171	Rho GTPase-activating protein 4	ARHGAP4	Homo sapiens	105.025	30	30	1508057	9629367
303	1	Q07065	Q07065	Cytoskeleton-associated protein 4	CKAP4	Homo sapiens	66.021	30	30	42098645	30988150
304	1	Q08426	Q08426	Peroxisomal bifunctional enzyme	EHHADH	Homo sapiens	79.493	30	30	8586411	2739992
305	1	Q15276	Q15276	Rab GTPase-binding effector protein 1	RABEP1	Homo sapiens	99.291	30	30	3605226	1617923
306	1	Q5JSH3	Q5JSH3	WD repeat-containing protein 44	WDR44	Homo sapiens	101.369	30	30	7873813	4486120
307	1	Q7L2E3	Q7L2E3	ATP-dependent RNA helicase DHX30	DHX30	Homo sapiens	133.939	30	30	3959927	354431
308	1	Q7L576	Q7L576	Cytoplasmic FMR1-interacting protein 1	CYFIP1	Homo sapiens	145.185	41	30	20797397	4893454
309	1	Q7RTP6	Q7RTP6	[F-actin]-monooxygenase MICAL3	MICAL3	Homo sapiens	224.298	32	30	4337148	42466
310	1	Q7Z3J2	Q7Z3J2	VPS35 endosomal protein-sorting factor-like	VPS35L	Homo sapiens	109.566	30	30	5204651	2877361
311	1	Q86UX2	Q86UX2	Inter-alpha-trypsin inhibitor heavy chain H5	ITIH5	Homo sapiens	104.578	30	30	19819046	29629
312	1	Q86V48	Q86V48	Leucine zipper protein 1	LUZP1	Homo sapiens	120.276	30	30	3983476	561202
313	1	Q92878	Q92878	DNA repair protein RAD50	RAD50	Homo sapiens	153.893	30	30	7093742	5942970
314	1	Q92888	Q92888	Rho guanine nucleotide exchange factor 1	ARHGEF1	Homo sapiens	102.435	30	30	6427895	16455469
315	1	Q96JE9	Q96JE9	Microtubule-associated protein 6	MAP6	Homo sapiens	86.507	30	30	7503243	0
316	1	Q96PE2	Q96PE2	Rho guanine nucleotide exchange factor 17	ARHGEF17	Homo sapiens	221.675	30	30	5013768	535933
317	1	Q9C0C9	Q9C0C9	(E3-independent) E2 ubiquitin-conjugating enzyme	UBE2O	Homo sapiens	141.296	30	30	2973547	2027735
318	1	Q9UPU5	Q9UPU5	Ubiquitin carboxyl-terminal hydrolase 24	USP24	Homo sapiens	294.369	30	30	4751496	1140650
319	1	Q9Y5B9	Q9Y5B9	FACT complex subunit SPT16	SUPT16H	Homo sapiens	119.916	30	30	8317303	3182448
320	1	O00160	O00160	Unconventional myosin-If	MYO1F	Homo sapiens	124.846	32	29	2546775	37740624
321	1	O60763	O60763	General vesicular transport factor p115	USO1	Homo sapiens	107.895	29	29	15477339	9971961
322	1	O75976	O75976	Carboxypeptidase D	CPD	Homo sapiens	152.935	29	29	4447487	8993092
323	1	P13611	P13611	Versican core protein	VCAN	Homo sapiens	372.826	29	29	34543828	13618904
324	1	P15144	P15144	Aminopeptidase N	ANPEP	Homo sapiens	109.54	29	29	7656669	22883535
325	1	P35658	P35658	Nuclear pore complex protein Nup214	NUP214	Homo sapiens	213.623	29	29	8425928	6884331
326	1	P38646	P38646	Stress-70 protein, mitochondrial	HSPA9	Homo sapiens	73.682	29	29	87416909	44356925
327	1	P39880	P39880	Homeobox protein cut-like 1	CUX1	Homo sapiens	164.19	29	29	3463896	992427
328	1	P42356	P42356	Phosphatidylinositol 4-kinase alpha	PI4KA	Homo sapiens	236.834	36	29	3811327	1260110
329	1	P46459	P46459	Vesicle-fusing ATPase	NSF	Homo sapiens	82.597	29	29	32414127	30740020

330	1	Q14289	Q14289	Protein-tyrosine kinase 2-beta	PTK2B	Homo sapiens	115.875	29	29	1956271	7116704
331	1	Q29RF7	Q29RF7	Sister chromatid cohesion protein PDS5 homolog A	PDS5A	Homo sapiens	150.829	29	29	4739008	2089663
332	1	Q70J99	Q70J99	Protein unc-13 homolog D	UNC13D	Homo sapiens	123.284	29	29	890632	17519523
333	1	Q7Z460	Q7Z460	CLIP-associating protein 1	CLASP1	Homo sapiens	169.453	34	29	3921440	1043201
334	1	Q96K76	Q96K76	Ubiquitin carboxyl-terminal hydrolase 47	USP47	Homo sapiens	157.314	29	29	4598543	2453791
335	1	Q99698	Q99698	Lysosomal-trafficking regulator	LYST	Homo sapiens	429.148	29	29	435624	2588479
336	1	Q9H3U1	Q9H3U1	Protein unc-45 homolog A	UNC45A	Homo sapiens	103.077	29	29	13621878	3805674
337	1	Q9NSE4	Q9NSE4	Isoleucine--tRNA ligase, mitochondrial	IARS2	Homo sapiens	113.793	29	29	21238952	6557020
338	1	Q9UHB6	Q9UHB6	LIM domain and actin-binding protein 1	LIMA1	Homo sapiens	85.228	29	29	46064190	4765502
339	1	Q9UHD8	Q9UHD8	Septin-9	SEPTIN9	Homo sapiens	65.402	29	29	43431395	9659162
340	1	Q9UQ35	Q9UQ35	Serine/arginine repetitive matrix protein 2	SRRM2	Homo sapiens	299.619	29	29	13069493	3286421
341	1	Q9Y678	Q9Y678	Coatomer subunit gamma-1	COPG1	Homo sapiens	97.719	31	29	19286337	11228864
342	1	O95466	O95466	Formin-like protein 1	FMNL1	Homo sapiens	121.856	30	28	2445267	18913341
343	1	P02679	P02679	Fibrinogen gamma chain	FGG	Homo sapiens	51.511	30	28	411893102	18289303938
344	1	P07384	P07384	Calpain-1 catalytic subunit	CAPN1	Homo sapiens	81.892	28	28	51603053	24800821
345	1	P10643	P10643	Complement component C7	C7	Homo sapiens	93.519	31	28	9262202	42950749
346	1	P11498	P11498	Pyruvate carboxylase, mitochondrial	PC	Homo sapiens	129.633	28	28	6361513	2295377
347	1	P35442	P35442	Thrombospondin-2	THBS2	Homo sapiens	129.993	28	28	63498	7826690
348	1	P35606	P35606	Coatomer subunit beta'	COPB2	Homo sapiens	102.488	28	28	34253051	20077001
349	1	P43243	P43243	Matrin-3	MATR3	Homo sapiens	94.624	28	28	15220594	6235663
350	1	P43304	P43304	Glycerol-3-phosphate dehydrogenase, mitochondrial	GPD2	Homo sapiens	80.852	28	28	13521889	11462669
351	1	P45974	P45974	Ubiquitin carboxyl-terminal hydrolase 5	USP5	Homo sapiens	95.788	30	28	20214010	11433629
352	1	P78344	P78344	Eukaryotic translation initiation factor 4 gamma 2	EIF4G2	Homo sapiens	102.363	28	28	19501331	6485801
353	1	P78371	P78371	T-complex protein 1 subunit beta	CCT2	Homo sapiens	57.488	28	28	50002259	23965918
354	1	Q04637	Q04637	Eukaryotic translation initiation factor 4 gamma 1	EIF4G1	Homo sapiens	175.493	31	28	18313959	5768433
355	1	Q12768	Q12768	WASH complex subunit 5	WASHC5	Homo sapiens	134.289	28	28	4767915	3370098
356	1	Q12882	Q12882	Dihydropyrimidine dehydrogenase [NADP(+)]	DPYD	Homo sapiens	111.401	28	28	9382770	7422008
357	1	Q12888	Q12888	TP53-binding protein 1	TP53BP1	Homo sapiens	213.579	28	28	6564512	1429618
358	1	Q12913	Q12913	Receptor-type tyrosine-protein phosphatase eta	PTPRJ	Homo sapiens	145.944	28	28	1330563	15729636
359	1	Q13228	Q13228	Methanethiol oxidase	SELENBP1	Homo sapiens	52.39	28	28	256346001	9804047
360	1	Q14160	Q14160	Protein scribble homolog	SCRIB	Homo sapiens	174.916	30	28	7221397	1135456
361	1	Q14571	Q14571	Inositol 1,4,5-trisphosphate receptor type 2	ITPR2	Homo sapiens	308.07	28	28	2605105	2276137
362	1	Q5VT25	Q5VT25	Serine/threonine-protein kinase MRCK alpha	CDC42BPA	Homo sapiens	197.312	28	28	3608259	32869
363	1	Q6WCQ1	Q6WCQ1	Myosin phosphatase Rho-interacting protein	MPRIP	Homo sapiens	116.533	28	28	3434672	1116575
364	1	Q86UX7	Q86UX7	Fermitin family homolog 3	FERMT3	Homo sapiens	75.953	28	28	10293618	66203092
365	1	Q92598	Q92598	Heat shock protein 105 kDa	HSPH1	Homo sapiens	96.865	29	28	9800038	6765998
366	1	Q96QK1	Q96QK1	Vacuolar protein sorting-associated protein 35	VPS35	Homo sapiens	91.707	28	28	19579558	11645861
367	1	Q99460	Q99460	26S proteasome non-ATPase regulatory subunit 1	PSMD1	Homo sapiens	105.837	28	28	13020622	9349837
368	1	Q99959	Q99959	Plakophilin-2	PKP2	Homo sapiens	97.419	28	28	22149137	365210
369	1	Q9HC35	Q9HC35	Echinoderm microtubule-associated protein-like 4	EML4	Homo sapiens	108.916	28	28	15007158	4916747
370	1	Q9UDY2	Q9UDY2	Tight junction protein ZO-2	TJP2	Homo sapiens	133.958	28	28	9476655	2325048
371	1	Q9ULT8	Q9ULT8	E3 ubiquitin-protein ligase HECTD1	HECTD1	Homo sapiens	289.375	28	28	5627025	1230891
372	1	Q9Y520	Q9Y520	Protein PRRC2C	PRRC2C	Homo sapiens	316.918	29	28	4720699	2032794
373	1	A0MZ66	A0MZ66	Shootin-1	SHTN1	Homo sapiens	71.642	27	27	7205165	2891247
374	1	O14497	O14497	AT-rich interactive domain-containing protein 1A	ARID1A	Homo sapiens	242.048	29	27	5344665	572408
375	1	O14841	O14841	5-oxoprolinase	OPLAH	Homo sapiens	137.457	27	27	6133655	2970887
376	1	O15031	O15031	Plexin-B2	PLXNB2	Homo sapiens	205.13	27	27	8026656	1464482
377	1	O60701	O60701	UDP-glucose 6-dehydrogenase	UGDH	Homo sapiens	55.026	27	27	66699042	20721891

378	1	O94875	O94875	Sorbin and SH3 domain-containing protein 2	SORBS2	Homo sapiens	124.107	27	27	92105845	0
379	1	O95248	O95248	Myotubularin-related protein 5	SBF1	Homo sapiens	208.446	28	27	2212435	686605
380	1	O95786	O95786	Antiviral innate immune response receptor RIG-I	RIGI	Homo sapiens	106.599	27	27	3001180	3635714
381	1	P01833	P01833	Polymeric immunoglobulin receptor	PIGR	Homo sapiens	83.284	27	27	25953599	26931498
382	1	P06756	P06756	Integrin alpha-V	ITGAV	Homo sapiens	116.038	27	27	18757440	4401103
383	1	P09960	P09960	Leukotriene A-4 hydrolase	LTA4H	Homo sapiens	69.285	27	27	32686691	65229717
384	1	P13671	P13671	Complement component C6	C6	Homo sapiens	104.789	27	27	6966596	41616919
385	1	P14625	P14625	Endoplasmin	HSP90B1	Homo sapiens	92.472	32	27	274334907	142396722
386	1	P19367	P19367	Hexokinase-1	HK1	Homo sapiens	102.489	31	27	29653985	14829200
387	1	P19827	P19827	Inter-alpha-trypsin inhibitor heavy chain H1	ITIH1	Homo sapiens	101.39	29	27	19656489	25486598
388	1	P29475	P29475	Nitric oxide synthase, brain	NOS1	Homo sapiens	160.973	27	27	4154724	0
389	1	P36871	P36871	Phosphoglucomutase-1	PGM1	Homo sapiens	61.45	27	27	32682260	15293487
390	1	P42224	P42224	Signal transducer and activator of transcription 1-alpha/beta	STAT1	Homo sapiens	87.333	28	27	20271203	36866082
391	1	P43121	P43121	Cell surface glycoprotein MUC18	MCAM	Homo sapiens	71.611	27	27	55181560	3092626
392	1	P49750	P49750	YLP motif-containing protein 1	YLPM1	Homo sapiens	241.651	27	27	4213878	1378337
393	1	Q12965	Q12965	Unconventional myosin-Ie	MYO1E	Homo sapiens	127.063	27	27	7638547	2153821
394	1	Q13617	Q13617	Cullin-2	CUL2	Homo sapiens	86.982	27	27	8363816	5236449
395	1	Q14624	Q14624	Inter-alpha-trypsin inhibitor heavy chain H4	ITIH4	Homo sapiens	103.358	28	27	47515357	92825864
396	1	Q5T1M5	Q5T1M5	FK506-binding protein 15	FKBP15	Homo sapiens	133.63	27	27	3444933	5717276
397	1	Q63HR2	Q63HR2	Tensin-2	TNS2	Homo sapiens	152.581	27	27	10583444	0
398	1	Q66K14	Q66K14	TBC1 domain family member 9B	TBC1D9B	Homo sapiens	140.526	31	27	5435175	3671486
399	1	Q7Z7B0	Q7Z7B0	Filamin-A-interacting protein 1	FILIP1	Homo sapiens	138.109	27	27	4593100	0
400	1	Q8TDZ2	Q8TDZ2	[F-actin]-monooxygenase MICAL1	MICAL1	Homo sapiens	117.875	28	27	3367878	12558832
401	1	Q8WUM4	Q8WUM4	Programmed cell death 6-interacting protein	PDCD6IP	Homo sapiens	96.023	27	27	30571177	17764924
402	1	Q92499	Q92499	ATP-dependent RNA helicase DDX1	DDX1	Homo sapiens	82.434	27	27	19958695	8238350
403	1	Q96P48	Q96P48	Arf-GAP with Rho-GAP domain, ANK repeat and PH domain-containing protein 1	ARAP1	Homo sapiens	162.193	27	27	2451920	10241184
404	1	Q99973	Q99973	Telomerase protein component 1	TEP1	Homo sapiens	290.494	27	27	3010820	682523
405	1	Q9BZQ8	Q9BZQ8	Protein Niban 1	NIBAN1	Homo sapiens	103.136	27	27	47569086	19269796
406	1	Q9NTI5	Q9NTI5	Sister chromatid cohesion protein PDS5 homolog B	PDS5B	Homo sapiens	164.67	28	27	6098458	4697170
407	1	Q9P107	Q9P107	GEM-interacting protein	GMIP	Homo sapiens	106.683	27	27	1151280	6712201
408	1	Q9Y6F6	Q9Y6F6	Inositol 1,4,5-triphosphate receptor associated 1	IRAG1	Homo sapiens	97.959	27	27	12030037	16799
409	1	A0FGR8	A0FGR8	Extended synaptotagmin-2	ESYT2	Homo sapiens	102.358	26	26	14580919	6375822
410	1	O14936	O14936	Peripheral plasma membrane protein CASK	CASK	Homo sapiens	105.126	26	26	8984644	836300
411	1	O43242	O43242	26S proteasome non-ATPase regulatory subunit 3	PSMD3	Homo sapiens	60.978	26	26	16519910	13327091
412	1	O43795	O43795	Unconventional myosin-Ib	MYO1B	Homo sapiens	131.986	28	26	10391438	6328806
413	1	O75955	O75955	Flotillin-1	FLOT1	Homo sapiens	47.356	26	26	12127124	25345306
414	1	O94874	O94874	E3 UFM1-protein ligase 1	UFL1	Homo sapiens	89.597	26	26	13828457	5238605
415	1	P02647	P02647	Apolipoprotein A-I	APOA1	Homo sapiens	30.777	27	26	643412163	1139728551
416	1	P10809	P10809	60 kDa heat shock protein, mitochondrial	HSPD1	Homo sapiens	61.054	26	26	114902611	47938365
417	1	P13796	P13796	Plastin-2	LCP1	Homo sapiens	70.291	32	26	31265911	202678831
418	1	P14735	P14735	Insulin-degrading enzyme	IDE	Homo sapiens	117.97	26	26	8684415	4220795
419	1	P14868	P14868	Aspartate--tRNA ligase, cytoplasmic	DARS1	Homo sapiens	57.136	26	26	26133058	20926083
420	1	P21359	P21359	Neurofibromin	NF1	Homo sapiens	319.377	26	26	3539112	347099
421	1	P25705	P25705	ATP synthase subunit alpha, mitochondrial	ATP5F1A	Homo sapiens	59.75	26	26	305660052	68280607
422	1	P28838	P28838	Cytosol aminopeptidase	LAP3	Homo sapiens	56.168	26	26	41319452	37133112
423	1	P30101	P30101	Protein disulfide-isomerase A3	PDIA3	Homo sapiens	56.783	26	26	240383560	175563601
424	1	P31948	P31948	Stress-induced-phosphoprotein 1	STIP1	Homo sapiens	62.639	26	26	47604377	20905691

425	1	P40939	P40939	Trifunctional enzyme subunit alpha, mitochondrial	HADHA	Homo sapiens	82.999	26	26	107154274	30205105
426	1	P42566	P42566	Epidermal growth factor receptor substrate 15	EPS15	Homo sapiens	98.659	26	26	6896898	2819581
427	1	P54289	P54289	Voltage-dependent calcium channel subunit alpha-2/delta-1	CACNA2D1	Homo sapiens	124.57	26	26	30905496	1921323
428	1	P55060	P55060	Exportin-2	CSE1L	Homo sapiens	110.419	26	26	16174054	7681215
429	1	P55265	P55265	Double-stranded RNA-specific adenosine deaminase	ADAR	Homo sapiens	136.067	26	26	7230557	4982899
430	1	P82094	P82094	TATA element modulatory factor	TMF1	Homo sapiens	122.842	26	26	2533205	1777958
431	1	Q08379	Q08379	Golgin subfamily A member 2	GOLGA2	Homo sapiens	113.089	26	26	4466382	2610552
432	1	Q08J23	Q08J23	RNA cytosine C(5)-methyltransferase NSUN2	NSUN2	Homo sapiens	86.473	26	26	6540008	4352824
433	1	Q12906	Q12906	Interleukin enhancer-binding factor 3	ILF3	Homo sapiens	95.341	30	26	37138100	14416035
434	1	Q13435	Q13435	Splicing factor 3B subunit 2	SF3B2	Homo sapiens	100.23	26	26	17239402	5204369
435	1	Q15042	Q15042	Rab3 GTPase-activating protein catalytic subunit	RAB3GAP1	Homo sapiens	110.526	26	26	4388933	3103898
436	1	Q1KMD3	Q1KMD3	Heterogeneous nuclear ribonucleoprotein U-like protein 2	HNRNPUL2	Homo sapiens	85.105	26	26	31434761	21344284
437	1	Q7L014	Q7L014	Probable ATP-dependent RNA helicase DDX46	DDX46	Homo sapiens	117.363	26	26	7876929	5800829
438	1	Q86YV0	Q86YV0	RAS protein activator like-3	RASAL3	Homo sapiens	111.899	26	26	637445	4898947
439	1	Q8IUD2	Q8IUD2	ELKS/Rab6-interacting/CAST family member 1	ERC1	Homo sapiens	128.089	31	26	5674059	1457573
440	1	Q8N3C0	Q8N3C0	Activating signal cointegrator 1 complex subunit 3	ASCC3	Homo sapiens	251.464	26	26	2479193	770844
441	1	Q96A65	Q96A65	Exocyst complex component 4	EXOC4	Homo sapiens	110.499	26	26	8650121	3136819
442	1	Q9BZ29	Q9BZ29	Dedicator of cytokinesis protein 9	DOCK9	Homo sapiens	236.448	27	26	3000831	885518
443	1	Q9H2M9	Q9H2M9	Rab3 GTPase-activating protein non-catalytic subunit	RAB3GAP2	Homo sapiens	155.984	26	26	5404295	3951730
444	1	Q9NPP4	Q9NPP4	NLR family CARD domain-containing protein 4	NLRC4	Homo sapiens	116.159	26	26	34860	8107443
445	1	Q9UIA9	Q9UIA9	Exportin-7	XPO7	Homo sapiens	123.908	26	26	7284298	4831949
446	1	Q9UIG0	Q9UIG0	Tyrosine-protein kinase BAZ1B	BAZ1B	Homo sapiens	170.906	26	26	3120038	1271402
447	1	Q9Y2J2	Q9Y2J2	Band 4.1-like protein 3	EPB41L3	Homo sapiens	120.678	28	26	15718032	1643305
448	1	Q9Y2L5	Q9Y2L5	Trafficking protein particle complex subunit 8	TRAPPC8	Homo sapiens	160.998	26	26	2708062	2428494
449	1	Q9Y2Q0	Q9Y2Q0	Phospholipid-transporting ATPase IA	ATP8A1	Homo sapiens	131.369	27	26	2639175	11599217
450	1	O00410	O00410	Importin-5	IPO5	Homo sapiens	123.633	26	25	10668047	3548476
451	1	O14727	O14727	Apoptotic protease-activating factor 1	APAF1	Homo sapiens	141.843	25	25	2002545	11072316
452	1	O43432	O43432	Eukaryotic translation initiation factor 4 gamma 3	EIF4G3	Homo sapiens	176.656	25	25	3931803	1484210
453	1	O75376	O75376	Nuclear receptor corepressor 1	NCOR1	Homo sapiens	270.216	25	25	2711672	399187
454	1	O76074	O76074	cGMP-specific 3',5'-cyclic phosphodiesterase	PDE5A	Homo sapiens	99.986	25	25	7512129	52780
455	1	O94906	O94906	Pre-mRNA-processing factor 6	PRPF6	Homo sapiens	106.929	25	25	7032922	4206967
456	1	P00488	P00488	Coagulation factor XIII A chain	F13A1	Homo sapiens	83.268	25	25	32308367	15570969
457	1	P04003	P04003	C4b-binding protein alpha chain	C4BPA	Homo sapiens	67.034	26	25	33187291	440052668
458	1	P04040	P04040	Catalase	CAT	Homo sapiens	59.757	25	25	42096762	67060404
459	1	P08648	P08648	Integrin alpha-5	ITGA5	Homo sapiens	114.538	25	25	98783107	4793988
460	1	P11413	P11413	Glucose-6-phosphate 1-dehydrogenase	G6PD	Homo sapiens	59.259	25	25	8718197	36524131
461	1	P21980	P21980	Protein-glutamine gamma-glutamyltransferase 2	TGM2	Homo sapiens	77.328	26	25	44226108	47467400
462	1	P24928	P24928	DNA-directed RNA polymerase II subunit RPB1	POLR2A	Homo sapiens	217.18	25	25	4576799	2117770
463	1	P27487	P27487	Dipeptidyl peptidase 4	DPP4	Homo sapiens	88.279	25	25	5282686	26631125
464	1	P31939	P31939	Bifunctional purine biosynthesis protein ATIC	ATIC	Homo sapiens	64.616	25	25	34465399	18812853
465	1	P33993	P33993	DNA replication licensing factor MCM7	MCM7	Homo sapiens	81.31	25	25	5776870	11051947
466	1	P48147	P48147	Prolyl endopeptidase	PREP	Homo sapiens	80.699	25	25	21328237	5790202
467	1	P49368	P49368	T-complex protein 1 subunit gamma	CCT3	Homo sapiens	60.533	25	25	38982227	24240036
468	1	P49748	P49748	Very long-chain specific acyl-CoA dehydrogenase, mitochondrial	ACADVL	Homo sapiens	70.392	25	25	51788480	17121535
469	1	P49915	P49915	GMP synthase [glutamine-hydrolyzing]	GMPS	Homo sapiens	76.718	25	25	7431576	6360413
470	1	P52789	P52789	Hexokinase-2	HK2	Homo sapiens	102.383	26	25	7091703	5914873
471	1	P98095	P98095	Fibulin-2	FBLN2	Homo sapiens	126.574	25	25	5777596	862220
472	1	Q05086	Q05086	Ubiquitin-protein ligase E3A	UBE3A	Homo sapiens	100.691	25	25	4998752	1590557

473	1	Q12931	Q12931	Heat shock protein 75 kDa, mitochondrial	TRAP1	Homo sapiens	80.111	25	25	15690147	46394713
474	1	Q14195	Q14195	Dihydropyrimidinase-related protein 3	DPYSL3	Homo sapiens	61.962	26	25	224758019	3089565
475	1	Q14258	Q14258	E3 ubiquitin/ISG15 ligase TRIM25	TRIM25	Homo sapiens	70.975	25	25	11663665	6797680
476	1	Q15029	Q15029	116 kDa U5 small nuclear ribonucleoprotein component	EFTUD2	Homo sapiens	109.439	25	25	13667035	5191580
477	1	Q2PPJ7	Q2PPJ7	Ral GTPase-activating protein subunit alpha-2	RALGAPA2	Homo sapiens	210.774	26	25	1555046	3240275
478	1	Q4L180	Q4L180	Filamin A-interacting protein 1-like	FILIP1L	Homo sapiens	130.386	25	25	6522984	706546
479	1	Q5VT52	Q5VT52	Regulation of nuclear pre-mRNA domain-containing protein 2	RPRD2	Homo sapiens	156.02	25	25	17837603	407816
480	1	Q6P1M3	Q6P1M3	LLGL scribble cell polarity complex component 2	LLGL2	Homo sapiens	113.449	25	25	11230275	3416057
481	1	Q7Z2W4	Q7Z2W4	Zinc finger CCCH-type antiviral protein 1	ZC3HAV1	Homo sapiens	101.433	25	25	7437218	7768004
482	1	Q7Z4S6	Q7Z4S6	Kinesin-like protein KIF21A	KIF21A	Homo sapiens	187.18	28	25	4654180	426661
483	1	Q86TI0	Q86TI0	TBC1 domain family member 1	TBC1D1	Homo sapiens	133.087	25	25	4477331	2091869
484	1	Q86U86	Q86U86	Protein polybromo-1	PBRM1	Homo sapiens	192.951	25	25	3241638	1056441
485	1	Q8N2S1	Q8N2S1	Latent-transforming growth factor beta-binding protein 4	LTBP4	Homo sapiens	173.439	25	25	8334030	18385
486	1	Q8NE71	Q8NE71	ATP-binding cassette sub-family F member 1	ABCF1	Homo sapiens	95.927	25	25	9853203	4446158
487	1	Q8TF72	Q8TF72	Protein Shroom3	SHROOM3	Homo sapiens	216.86	26	25	11404932	0
488	1	Q92620	Q92620	Pre-mRNA-splicing factor ATP-dependent RNA helicase PRP16	DHX38	Homo sapiens	140.508	25	25	33929441	868465
489	1	Q92621	Q92621	Nuclear pore complex protein Nup205	NUP205	Homo sapiens	227.922	25	25	4929660	4253452
490	1	Q92835	Q92835	Phosphatidylinositol 3,4,5-trisphosphate 5-phosphatase 1	INPP5D	Homo sapiens	133.294	25	25	1609502	9194545
491	1	Q96AC1	Q96AC1	Fermitin family homolog 2	FERMT2	Homo sapiens	77.86	26	25	104279990	2615746
492	1	Q96JH7	Q96JH7	Deubiquitinating protein VCPIP1	VCPIP1	Homo sapiens	134.324	25	25	3037486	1863416
493	1	Q9C0D5	Q9C0D5	Protein TANC1	TANC1	Homo sapiens	202.222	25	25	2126502	0
494	1	Q9NQ66	Q9NQ66	1-phosphatidylinositol 4,5-bisphosphate phosphodiesterase beta-1	PLCB1	Homo sapiens	138.568	25	25	3583595	0
495	1	Q9NZM3	Q9NZM3	Intersectin-2	ITSN2	Homo sapiens	193.463	28	25	2493182	2125145
496	1	Q9UGP8	Q9UGP8	Translocation protein SEC63 homolog	SEC63	Homo sapiens	87.998	25	25	6545223	7576720
497	1	Q9UPT5	Q9UPT5	Exocyst complex component 7	EXOC7	Homo sapiens	83.382	25	25	7871494	3678277
498	1	Q9Y2A7	Q9Y2A7	Nck-associated protein 1	NCKAP1	Homo sapiens	128.79	25	25	15405329	1269016
499	1	Q9Y485	Q9Y485	DmX-like protein 1	DMXL1	Homo sapiens	337.845	25	25	1815885	755640
500	1	O00429	O00429	Dynamin-1-like protein	DNM1L	Homo sapiens	81.877	24	24	10972488	6552917
501	1	O14617	O14617	AP-3 complex subunit delta-1	AP3D1	Homo sapiens	130.159	24	24	10041517	4702943
502	1	O15195	O15195	Villin-like protein	VILL	Homo sapiens	95.907	24	24	7471787	47599
503	1	O75534	O75534	Cold shock domain-containing protein E1	CSDE1	Homo sapiens	88.885	24	24	15929214	5988137
504	1	O95202	O95202	Mitochondrial proton/calcium exchanger protein	LETM1	Homo sapiens	83.357	24	24	13755938	7275469
505	1	P00751	P00751	Complement factor B	CFB	Homo sapiens	85.534	24	24	78861733	140031667
506	1	P05556	P05556	Integrin beta-1	ITGB1	Homo sapiens	88.417	24	24	131103824	19360604
507	1	P06576	P06576	ATP synthase subunit beta, mitochondrial	ATP5F1B	Homo sapiens	56.559	24	24	345456751	104600499
508	1	P06737	P06737	Glycogen phosphorylase, liver form	PYGL	Homo sapiens	97.151	26	24	10578526	32790876
509	1	P12429	P12429	Annexin A3	ANXA3	Homo sapiens	36.376	24	24	15832961	332865398
510	1	P17927	P17927	Complement receptor type 1	CR1	Homo sapiens	223.668	27	24	653978	6403824
511	1	P29350	P29350	Tyrosine-protein phosphatase non-receptor type 6	PTPN6	Homo sapiens	67.561	24	24	8393959	37252194
512	1	P29590	P29590	Protein PML	PML	Homo sapiens	97.551	24	24	6901254	8054658
513	1	P32004	P32004	Neural cell adhesion molecule L1	L1CAM	Homo sapiens	140.006	24	24	16251067	0
514	1	P33992	P33992	DNA replication licensing factor MCM5	MCM5	Homo sapiens	82.284	24	24	6112516	2480501
515	1	P36776	P36776	Lon protease homolog, mitochondrial	LONP1	Homo sapiens	106.492	24	24	12539076	10308694
516	1	P40763	P40763	Signal transducer and activator of transcription 3	STAT3	Homo sapiens	88.068	24	24	21679511	16029005
517	1	P48444	P48444	Coatomer subunit delta	ARCN1	Homo sapiens	57.212	24	24	37653817	21356047
518	1	P51610	P51610	Host cell factor 1	HCFC1	Homo sapiens	208.734	24	24	8994585	4137365
519	1	P52272	P52272	Heterogeneous nuclear ribonucleoprotein M	HNRNPM	Homo sapiens	77.518	24	24	63139212	44547436
520	1	P52790	P52790	Hexokinase-3	HK3	Homo sapiens	99.028	24	24	1720123	50450362

521	1	P54886	P54886	Delta-1-pyrroline-5-carboxylate synthase	ALDH18A1	Homo sapiens	87.304	24	24	9596769	4944838
522	1	P55786	P55786	Puromycin-sensitive aminopeptidase	NPEPPS	Homo sapiens	103.277	35	24	38679324	11456128
523	1	Q02809	Q02809	Procollagen-lysine,2-oxoglutarate 5-dioxygenase 1	PLOD1	Homo sapiens	83.553	24	24	4423359	9135782
524	1	Q02818	Q02818	Nucleobindin-1	NUCB1	Homo sapiens	53.879	24	24	14690951	5446879
525	1	Q06210	Q06210	Glutamine--fructose-6-phosphate aminotransferase [isomerizing] 1	GFPT1	Homo sapiens	78.806	27	24	34573546	13841847
526	1	Q12791	Q12791	Calcium-activated potassium channel subunit alpha-1	KCNMA1	Homo sapiens	137.56	24	24	4339880	0
527	1	Q13423	Q13423	NAD(P) transhydrogenase, mitochondrial	NNT	Homo sapiens	113.895	24	24	49035184	15258564
528	1	Q14974	Q14974	Importin subunit beta-1	KPNB1	Homo sapiens	97.172	24	24	28184400	14582321
529	1	Q16706	Q16706	Alpha-mannosidase 2	MAN2A1	Homo sapiens	131.144	25	24	6135729	1619150
530	1	Q16851	Q16851	UTP--glucose-1-phosphate uridylyltransferase	UGP2	Homo sapiens	56.942	24	24	50781131	33184060
531	1	Q5K651	Q5K651	Sterile alpha motif domain-containing protein 9	SAMD9	Homo sapiens	184.282	25	24	1441408	2413339
532	1	Q69YQ0	Q69YQ0	Cytospin-A	SPECC1L	Homo sapiens	124.543	24	24	3507649	286061
533	1	Q7LBC6	Q7LBC6	Lysine-specific demethylase 3B	KDM3B	Homo sapiens	191.584	25	24	2398545	301695
534	1	Q7Z478	Q7Z478	ATP-dependent RNA helicase DHX29	DHX29	Homo sapiens	155.239	25	24	3107808	954987
535	1	Q8N573	Q8N573	Oxidation resistance protein 1	OXR1	Homo sapiens	97.973	24	24	6843861	1241436
536	1	Q8NDA8	Q8NDA8	Maestro heat-like repeat-containing protein family member 1	MROH1	Homo sapiens	181.252	24	24	1819929	1854023
537	1	Q8NFC6	Q8NFC6	Biorientation of chromosomes in cell division protein 1-like 1	BOD1L1	Homo sapiens	330.471	24	24	1238587	195472
538	1	Q8TEM1	Q8TEM1	Nuclear pore membrane glycoprotein 210	NUP210	Homo sapiens	205.115	24	24	4415176	5382749
539	1	Q8TEW0	Q8TEW0	Partitioning defective 3 homolog	PARD3	Homo sapiens	151.426	24	24	3302035	187533
540	1	Q92945	Q92945	Far upstream element-binding protein 2	KHSRP	Homo sapiens	73.117	24	24	21532481	5797241
541	1	Q96B97	Q96B97	SH3 domain-containing kinase-binding protein 1	SH3KBP1	Homo sapiens	73.126	24	24	5668599	8033605
542	1	Q96JQ2	Q96JQ2	Calmin	CLMN	Homo sapiens	111.652	24	24	4879786	10376
543	1	Q9BY89	Q9BY89	Uncharacterized protein KIAA1671	KIAA1671	Homo sapiens	196.714	24	24	2234586	234591
544	1	Q9H4A4	Q9H4A4	Aminopeptidase B	RNPEP	Homo sapiens	72.598	24	24	22356572	13744427
545	1	Q9NQX4	Q9NQX4	Unconventional myosin-Vc	MYO5C	Homo sapiens	202.814	24	24	2996387	0
546	1	Q9NRY4	Q9NRY4	Rho GTPase-activating protein 35	ARHGAP35	Homo sapiens	170.516	24	24	2225985	538988
547	1	Q9NZN5	Q9NZN5	Rho guanine nucleotide exchange factor 12	ARHGEF12	Homo sapiens	173.237	24	24	3698147	482981
548	1	Q9P2D3	Q9P2D3	HEAT repeat-containing protein 5B	HEATR5B	Homo sapiens	224.306	24	24	3462603	622091
549	1	Q9UBC2	Q9UBC2	Epidermal growth factor receptor substrate 15-like 1	EPS15L1	Homo sapiens	94.257	24	24	10530436	4397027
550	1	Q9UDT6	Q9UDT6	CAP-Gly domain-containing linker protein 2	CLIP2	Homo sapiens	115.837	24	24	3871842	592055
551	1	Q9UH99	Q9UH99	SUN domain-containing protein 2	SUN2	Homo sapiens	80.314	25	24	6176320	4120971
552	1	Q9UKV3	Q9UKV3	Apoptotic chromatin condensation inducer in the nucleus	ACIN1	Homo sapiens	151.864	24	24	8221901	2465582
553	1	Q9Y230	Q9Y230	RuvB-like 2	RUVBL2	Homo sapiens	51.156	24	24	20268656	13751840
554	1	Q9Y3P9	Q9Y3P9	Rab GTPase-activating protein 1	RABGAP1	Homo sapiens	121.737	24	24	6882693	843563
555	1	O00423	O00423	Echinoderm microtubule-associated protein-like 1	EML1	Homo sapiens	89.865	23	23	23763904	79502
556	1	O14672	O14672	Disintegrin and metalloproteinase domain-containing protein 10	ADAM10	Homo sapiens	84.14	23	23	11253946	6868089
557	1	O43143	O43143	ATP-dependent RNA helicase DHX15	DHX15	Homo sapiens	90.934	24	23	10080446	6908484
558	1	O43520	O43520	Phospholipid-transporting ATPase IC	ATP8B1	Homo sapiens	143.697	25	23	7906724	227008
559	1	O75122	O75122	CLIP-associating protein 2	CLASP2	Homo sapiens	141.065	23	23	5077376	911747
560	1	O75694	O75694	Nuclear pore complex protein Nup155	NUP155	Homo sapiens	155.203	23	23	5447639	3831905
561	1	O75962	O75962	Triple functional domain protein	TRIO	Homo sapiens	346.905	27	23	1510545	113391
562	1	O94979	O94979	Protein transport protein Sec31A	SEC31A	Homo sapiens	133.018	23	23	17188851	9781365
563	1	O95479	O95479	GDH/6PGL endoplasmic bifunctional protein	H6PD	Homo sapiens	88.896	23	23	5044518	7451440
564	1	P05023	P05023	Sodium/potassium-transporting ATPase subunit alpha-1	ATP1A1	Homo sapiens	112.897	37	23	127794143	27430109
565	1	P06681	P06681	Complement C2	C2	Homo sapiens	83.269	23	23	8449595	14568241
566	1	P14923	P14923	Junction plakoglobin	JUP	Homo sapiens	81.745	23	23	34779326	2380952
567	1	P17980	P17980	26S proteasome regulatory subunit 6A	PSMC3	Homo sapiens	49.204	23	23	16970220	12008771
568	1	P18583	P18583	Protein SON	SON	Homo sapiens	263.835	23	23	5772824	2348411

569	1	P21399	P21399	Cytoplasmic aconitate hydratase	ACO1	Homo sapiens	98.403	23	23	27329792	5183415
570	1	P22626	P22626	Heterogeneous nuclear ribonucleoproteins A2/B1	HNRNPA2B1	Homo sapiens	37.428	24	23	159234142	86605114
571	1	P23229	P23229	Integrin alpha-6	ITGA6	Homo sapiens	126.604	23	23	11435073	2906940
572	1	P23458	P23458	Tyrosine-protein kinase JAK1	JAK1	Homo sapiens	133.279	23	23	2591573	2224246
573	1	P35221	P35221	Catenin alpha-1	CTNNA1	Homo sapiens	100.073	32	23	43942630	3906674
574	1	P35222	P35222	Catenin beta-1	CTNNB1	Homo sapiens	85.496	26	23	25265620	2577658
575	1	P42331	P42331	Rho GTPase-activating protein 25	ARHGAP25	Homo sapiens	73.435	23	23	1205985	11577074
576	1	P43490	P43490	Nicotinamide phosphoribosyltransferase	NAMPT	Homo sapiens	55.523	23	23	13074948	146698758
577	1	P48634	P48634	Protein PRRC2A	PRRC2A	Homo sapiens	228.866	23	23	3101810	410888
578	1	P49591	P49591	Serine--tRNA ligase, cytoplasmic	SARS1	Homo sapiens	58.778	23	23	13123052	6767164
579	1	P49736	P49736	DNA replication licensing factor MCM2	MCM2	Homo sapiens	101.897	23	23	4553604	2006357
580	1	P55011	P55011	Solute carrier family 12 member 2	SLC12A2	Homo sapiens	131.448	24	23	15287242	487549
581	1	Q01813	Q01813	ATP-dependent 6-phosphofructokinase, platelet type	PFKP	Homo sapiens	85.598	23	23	22659349	16080365
582	1	Q01970	Q01970	1-phosphatidylinositol 4,5-bisphosphate phosphodiesterase beta-3	PLCB3	Homo sapiens	138.802	23	23	6184189	664855
583	1	Q14966	Q14966	Zinc finger protein 638	ZNF638	Homo sapiens	220.628	23	23	3685528	1629957
584	1	Q15418	Q15418	Ribosomal protein S6 kinase alpha-1	RPS6KA1	Homo sapiens	82.725	31	23	7871225	10538663
585	1	Q27J81	Q27J81	Inverted formin-2	INF2	Homo sapiens	135.625	23	23	8503775	926423
586	1	Q3V6T2	Q3V6T2	Girdin	CCDC88A	Homo sapiens	216.049	23	23	1410048	838472
587	1	Q567U6	Q567U6	Coiled-coil domain-containing protein 93	CCDC93	Homo sapiens	73.196	23	23	6808850	5238346
588	1	Q5TZA2	Q5TZA2	Rootletin	CROCC	Homo sapiens	228.427	24	23	2164878	20366
589	1	Q6P1N0	Q6P1N0	Coiled-coil and C2 domain-containing protein 1A	CC2D1A	Homo sapiens	104.061	23	23	6154230	1842347
590	1	Q86W92	Q86W92	Liprin-beta-1	PPFIBP1	Homo sapiens	114.025	25	23	4870572	1340193
591	1	Q8NFP9	Q8NFP9	Neurobeachin	NBEA	Homo sapiens	327.825	23	23	1811404	0
592	1	Q8TD43	Q8TD43	Transient receptor potential cation channel subfamily M member 4	TRPM4	Homo sapiens	134.301	23	23	9531590	18368
593	1	Q8TEQ6	Q8TEQ6	Gem-associated protein 5	GEMIN5	Homo sapiens	168.592	23	23	2938553	677109
594	1	Q96FS4	Q96FS4	Signal-induced proliferation-associated protein 1	SIPA1	Homo sapiens	112.149	23	23	1355290	4585244
595	1	Q96KP4	Q96KP4	Cytosolic non-specific dipeptidase	CNDP2	Homo sapiens	52.877	23	23	42834869	29443227
596	1	Q96Q15	Q96Q15	Serine/threonine-protein kinase SMG1	SMG1	Homo sapiens	410.507	24	23	1150005	418509
597	1	Q96QH2	Q96QH2	PML-RARA-regulated adapter molecule 1	PRAM1	Homo sapiens	73.969	23	23	39521	8601445
598	1	Q96T51	Q96T51	RUN and FYVE domain-containing protein 1	RUFY1	Homo sapiens	79.819	24	23	5393476	3494817
599	1	Q9BQG0	Q9BQG0	Myb-binding protein 1A	MYBBP1A	Homo sapiens	148.856	23	23	2309208	1004747
600	1	Q9BTC0	Q9BTC0	Death-inducer obliterator 1	DIDO1	Homo sapiens	243.876	23	23	3814798	833403
601	1	Q9BZL4	Q9BZL4	Protein phosphatase 1 regulatory subunit 12C	PPP1R12C	Homo sapiens	84.883	23	23	10192541	415633
602	1	Q9H270	Q9H270	Vacuolar protein sorting-associated protein 11 homolog	VPS11	Homo sapiens	107.839	23	23	4069000	4892479
603	1	Q9HD20	Q9HD20	Endoplasmic reticulum transmembrane helix translocase	ATP13A1	Homo sapiens	132.957	23	23	5977902	5170899
604	1	Q9NVE7	Q9NVE7	4'-phosphopantetheine phosphatase	PANK4	Homo sapiens	85.992	23	23	6922613	1375132
605	1	Q9NZN4	Q9NZN4	EH domain-containing protein 2	EHD2	Homo sapiens	61.16	24	23	168850329	2300585
606	1	Q9UIQ6	Q9UIQ6	Leucyl-cystinyl aminopeptidase	LNPEP	Homo sapiens	117.35	23	23	10008760	4122271
607	1	Q9ULH1	Q9ULH1	Arf-GAP with SH3 domain, ANK repeat and PH domain-containing protein 1	ASAP1	Homo sapiens	125.499	24	23	1568992	7102222
608	1	Q9UPQ0	Q9UPQ0	LIM and calponin homology domains-containing protein 1	LIMCH1	Homo sapiens	121.871	23	23	3543637	0
609	1	Q9Y263	Q9Y263	Phospholipase A-2-activating protein	PLAA	Homo sapiens	87.158	23	23	7984645	5722197
610	1	Q9Y446	Q9Y446	Plakophilin-3	PKP3	Homo sapiens	87.08	23	23	7535365	1193456
611	1	O00232	O00232	26S proteasome non-ATPase regulatory subunit 12	PSMD12	Homo sapiens	52.906	22	22	11079625	8433803
612	1	O00267	O00267	Transcription elongation factor SPT5	SUPT5H	Homo sapiens	121.002	22	22	4126780	2603807
613	1	O14976	O14976	Cyclin-G-associated kinase	GAK	Homo sapiens	143.193	22	22	5085364	3155845
614	1	O60306	O60306	RNA helicase aquarius	AQR	Homo sapiens	171.299	22	22	2638151	2110680

615	1	O60568	O60568	Multifunctional procollagen lysine hydroxylase and glycosyltransferase LH3	PLOD3	Homo sapiens	84.787	22	22	7094339	5039241
616	1	O60645	O60645	Exocyst complex component 3	EXOC3	Homo sapiens	85.567	22	22	4562984	1482613
617	1	O60826	O60826	Coiled-coil domain-containing protein 22	CCDC22	Homo sapiens	70.754	22	22	4436011	2938479
618	1	O75083	O75083	WD repeat-containing protein 1	WDR1	Homo sapiens	66.194	22	22	174432631	81689455
619	1	O76094	O76094	Signal recognition particle subunit SRP72	SRP72	Homo sapiens	74.609	22	22	10416112	5015806
620	1	O94973	O94973	AP-2 complex subunit alpha-2	AP2A2	Homo sapiens	103.961	29	22	18359697	4512000
621	1	O95163	O95163	Elongator complex protein 1	ELP1	Homo sapiens	150.254	22	22	4777494	1379439
622	1	O95782	O95782	AP-2 complex subunit alpha-1	AP2A1	Homo sapiens	107.547	22	22	13811459	6084240
623	1	P05166	P05166	Propionyl-CoA carboxylase beta chain, mitochondrial	PCCB	Homo sapiens	58.215	22	22	20720000	1657262
624	1	P12268	P12268	Inosine-5'-monophosphate dehydrogenase 2	IMPDH2	Homo sapiens	55.806	23	22	13519879	13947999
625	1	P14780	P14780	Matrix metalloproteinase-9	MMP9	Homo sapiens	78.458	22	22	2211967	61818413
626	1	P16435	P16435	NADPH--cytochrome P450 reductase	POR	Homo sapiens	76.69	22	22	7650353	11325020
627	1	P17480	P17480	Nucleolar transcription factor 1	UBTF	Homo sapiens	89.408	22	22	6478871	983220
628	1	P23634	P23634	Plasma membrane calcium-transporting ATPase 4	ATP2B4	Homo sapiens	137.923	34	22	61348853	7069519
629	1	P26639	P26639	Threonine--tRNA ligase 1, cytoplasmic	TARS1	Homo sapiens	83.436	23	22	16449776	10075849
630	1	P41250	P41250	Glycine--tRNA ligase	GARS1	Homo sapiens	83.167	22	22	8203192	7755460
631	1	P42166	P42166	Lamina-associated polypeptide 2, isoform alpha	TMPO	Homo sapiens	75.492	22	22	18021356	8726667
632	1	P43405	P43405	Tyrosine-protein kinase SYK	SYK	Homo sapiens	72.068	22	22	3086018	17375248
633	1	P47989	P47989	Xanthine dehydrogenase/oxidase	XDH	Homo sapiens	146.427	23	22	627166	10664152
634	1	P49419	P49419	Alpha-aminoadipic semialdehyde dehydrogenase	ALDH7A1	Homo sapiens	58.487	22	22	21549078	4124386
635	1	P49790	P49790	Nuclear pore complex protein Nup153	NUP153	Homo sapiens	153.941	22	22	5388988	1630310
636	1	P50416	P50416	Carnitine O-palmitoyltransferase 1, liver isoform	CPT1A	Homo sapiens	88.367	23	22	32259356	6845907
637	1	P50895	P50895	Basal cell adhesion molecule	BCAM	Homo sapiens	67.407	22	22	15578863	1543922
638	1	P54577	P54577	Tyrosine--tRNA ligase, cytoplasmic	YARS1	Homo sapiens	59.144	22	22	14854136	19931222
639	1	P55884	P55884	Eukaryotic translation initiation factor 3 subunit B	EIF3B	Homo sapiens	92.482	22	22	17903839	10551551
640	1	P56192	P56192	Methionine--tRNA ligase, cytoplasmic	MARS1	Homo sapiens	101.116	22	22	10451299	8041119
641	1	Q01118	Q01118	Sodium channel protein type 7 subunit alpha	SCN7A	Homo sapiens	193.497	22	22	5645011	0
642	1	Q01995	Q01995	Transgelin	TAGLN	Homo sapiens	22.61	22	22	4267665275	44340139
643	1	Q06124	Q06124	Tyrosine-protein phosphatase non-receptor type 11	PTPN11	Homo sapiens	68.012	22	22	14848083	6024388
644	1	Q0ZGT2	Q0ZGT2	Nexilin	NEXN	Homo sapiens	80.661	22	22	26430359	791543
645	1	Q12929	Q12929	Epidermal growth factor receptor kinase substrate 8	EPS8	Homo sapiens	91.884	22	22	6638459	2267668
646	1	Q13428	Q13428	Treacle protein	TCOF1	Homo sapiens	152.11	22	22	9406365	2373813
647	1	Q14118	Q14118	Dystroglycan 1	DAG1	Homo sapiens	97.443	22	22	11255269	870547
648	1	Q14247	Q14247	Src substrate cortactin	CTTN	Homo sapiens	61.585	22	22	18748367	4175361
649	1	Q14C86	Q14C86	GTPase-activating protein and VPS9 domain-containing protein 1	GAPVD1	Homo sapiens	164.979	22	22	3247237	3833171
650	1	Q15020	Q15020	Squamous cell carcinoma antigen recognized by T-cells 3	SART3	Homo sapiens	109.935	22	22	6322501	2610924
651	1	Q15386	Q15386	Ubiquitin-protein ligase E3C	UBE3C	Homo sapiens	123.924	22	22	5808464	1783789
652	1	Q15477	Q15477	SKI2 subunit of superkiller complex protein	SKIC2	Homo sapiens	137.757	22	22	3618535	1341464
653	1	Q15811	Q15811	Intersectin-1	ITSN1	Homo sapiens	195.424	22	22	4993105	188574
654	1	Q16822	Q16822	Phosphoenolpyruvate carboxykinase [GTP], mitochondrial	PCK2	Homo sapiens	70.701	25	22	18643723	2525087
655	1	Q3SY69	Q3SY69	Mitochondrial 10-formyltetrahydrofolate dehydrogenase	ALDH1L2	Homo sapiens	101.747	23	22	5177510	3879710
656	1	Q4V328	Q4V328	GRIP1-associated protein 1	GRIPAP1	Homo sapiens	96.006	22	22	4701481	3502172
657	1	Q5JTV8	Q5JTV8	Torsin-1A-interacting protein 1	TOR1AIP1	Homo sapiens	66.246	23	22	12429579	21726037
658	1	Q5SRE5	Q5SRE5	Nucleoporin NUP188	NUP188	Homo sapiens	196.046	22	22	2274236	856100
659	1	Q5T447	Q5T447	E3 ubiquitin-protein ligase HECTD3	HECTD3	Homo sapiens	97.114	22	22	5091864	2113354
660	1	Q5VZK9	Q5VZK9	F-actin-uncapping protein LRRC16A	CARMIL1	Homo sapiens	151.557	22	22	3652894	666590
661	1	Q6KC79	Q6KC79	Nipped-B-like protein	NIPBL	Homo sapiens	316.058	22	22	1357124	660566

662	1	Q7Z6P3	Q7Z6P3	Ras-related protein Rab-44	RAB44	Homo sapiens	110.85	22	22	0	7080780
663	1	Q8IZH2	Q8IZH2	5'-3' exoribonuclease 1	XRN1	Homo sapiens	194.109	22	22	2195418	1605004
664	1	Q8N766	Q8N766	ER membrane protein complex subunit 1	EMC1	Homo sapiens	111.761	22	22	7038718	3987187
665	1	Q8NCN5	Q8NCN5	Pyruvate dehydrogenase phosphatase regulatory subunit, mitochondrial	PDPR	Homo sapiens	99.364	22	22	6916581	1192159
666	1	Q8TDB6	Q8TDB6	E3 ubiquitin-protein ligase DTX3L	DTX3L	Homo sapiens	83.556	22	22	3138898	2468189
667	1	Q8WUM0	Q8WUM0	Nuclear pore complex protein Nup133	NUP133	Homo sapiens	128.981	22	22	4999735	2461957
668	1	Q8WVV4	Q8WVV4	Protein POF1B	POF1B	Homo sapiens	68.064	22	22	16341545	0
669	1	Q92797	Q92797	Symplekin	SYMPK	Homo sapiens	141.151	22	22	2794618	900831
670	1	Q92841	Q92841	Probable ATP-dependent RNA helicase DDX17	DDX17	Homo sapiens	80.276	29	22	23711517	13059861
671	1	Q92896	Q92896	Golgi apparatus protein 1	GLG1	Homo sapiens	134.556	22	22	7368348	2739783
672	1	Q99832	Q99832	T-complex protein 1 subunit eta	CCT7	Homo sapiens	59.365	22	22	20939459	15913527
673	1	Q9BSJ2	Q9BSJ2	Gamma-tubulin complex component 2	TUBGCP2	Homo sapiens	102.536	22	22	3773384	1471898
674	1	Q9BZZ2	Q9BZZ2	Sialoadhesin	SIGLEC1	Homo sapiens	182.624	22	22	1855059	3519308
675	1	Q9H1H9	Q9H1H9	Kinesin-like protein KIF13A	KIF13A	Homo sapiens	202.313	22	22	4007215	836717
676	1	Q9HCC0	Q9HCC0	Methylcrotonoyl-CoA carboxylase beta chain, mitochondrial	MCCC2	Homo sapiens	61.332	22	22	12328250	4720686
677	1	Q9NR30	Q9NR30	Nucleolar RNA helicase 2	DDX21	Homo sapiens	87.344	24	22	5713536	3091256
678	1	Q9NSK0	Q9NSK0	Kinesin light chain 4	KLC4	Homo sapiens	68.641	25	22	8385001	272456
679	1	Q9NTJ3	Q9NTJ3	Structural maintenance of chromosomes protein 4	SMC4	Homo sapiens	147.183	22	22	4438210	2301279
680	1	Q9P2B2	Q9P2B2	Prostaglandin F2 receptor negative regulator	PTGFRN	Homo sapiens	98.558	22	22	24147464	564807
681	1	A8K7I4	A8K7I4	Calcium-activated chloride channel regulator 1	CLCA1	Homo sapiens	100.226	21	21	184340459	1463232
682	1	O00203	O00203	AP-3 complex subunit beta-1	AP3B1	Homo sapiens	121.321	28	21	10215152	9500239
683	1	O00567	O00567	Nucleolar protein 56	NOP56	Homo sapiens	66.051	21	21	7935863	2485564
684	1	O14776	O14776	Transcription elongation regulator 1	TCERG1	Homo sapiens	123.903	22	21	4385788	2667875
685	1	O15067	O15067	Phosphoribosylformylglycinamidine synthase	PFAS	Homo sapiens	144.738	21	21	5602601	3052187
686	1	O43390	O43390	Heterogeneous nuclear ribonucleoprotein R	HNRNPR	Homo sapiens	70.943	28	21	24145966	8013160
687	1	O75153	O75153	Clustered mitochondria protein homolog	CLUH	Homo sapiens	146.673	21	21	4943224	825840
688	1	O95071	O95071	E3 ubiquitin-protein ligase UBR5	UBR5	Homo sapiens	309.357	21	21	1701306	511949
689	1	O95219	O95219	Sorting nexin-4	SNX4	Homo sapiens	51.911	21	21	6313613	927630
690	1	O95340	O95340	Bifunctional 3'-phosphoadenosine 5'-phosphosulfate synthase 2	PAPSS2	Homo sapiens	69.498	22	21	18769508	4378626
691	1	P00734	P00734	Prothrombin	F2	Homo sapiens	70.037	28	21	18203814	205403004
692	1	P01009	P01009	Alpha-1-antitrypsin	SERPINA1	Homo sapiens	46.735	21	21	855616706	1156785857
693	1	P02786	P02786	Transferrin receptor protein 1	TFRC	Homo sapiens	84.872	21	21	10496978	7316121
694	1	P02790	P02790	Hemopexin	HPX	Homo sapiens	51.678	21	21	294988151	463879743
695	1	P06727	P06727	Apolipoprotein A-IV	APOA4	Homo sapiens	45.373	22	21	8037242	15206486
696	1	P07237	P07237	Protein disulfide-isomerase	P4HB	Homo sapiens	57.118	21	21	89436738	129371335
697	1	P07358	P07358	Complement component C8 beta chain	C8B	Homo sapiens	67.045	21	21	4811891	25343623
698	1	P08473	P08473	Neprilysin	MME	Homo sapiens	85.514	21	21	356191	7099805
699	1	P08514	P08514	Integrin alpha-IIb	ITGA2B	Homo sapiens	113.378	21	21	988416	16702601
700	1	P09525	P09525	Annexin A4	ANXA4	Homo sapiens	35.883	21	21	35818036	50148486
701	1	P10909	P10909	Clusterin	CLU	Homo sapiens	52.494	21	21	33087820	289377539
702	1	P11142	P11142	Heat shock cognate 71 kDa protein	HSPA8	Homo sapiens	70.899	28	21	217433817	132747923
703	1	P11217	P11217	Glycogen phosphorylase, muscle form	PYGM	Homo sapiens	97.091	21	21	11498837	0
704	1	P16284	P16284	Platelet endothelial cell adhesion molecule	PECAM1	Homo sapiens	82.524	21	21	7175262	10820487
705	1	P17858	P17858	ATP-dependent 6-phosphofructokinase, liver type	PFKL	Homo sapiens	85.018	24	21	25449629	18121725
706	1	P17987	P17987	T-complex protein 1 subunit alpha	TCP1	Homo sapiens	60.343	21	21	35523583	22503378
707	1	P20936	P20936	Ras GTPase-activating protein 1	RASA1	Homo sapiens	116.405	21	21	3004436	1897360
708	1	P22059	P22059	Oxysterol-binding protein 1	OSBP	Homo sapiens	89.422	22	21	11116754	4092372
709	1	P23526	P23526	Adenosylhomocysteinase	AHCY	Homo sapiens	47.715	21	21	58211880	23009237

710	1	P27694	P27694	Replication protein A 70 kDa DNA-binding subunit	RPA1	Homo sapiens	68.139	21	21	8790025	8319825
711	1	P27824	P27824	Calnexin	CANX	Homo sapiens	67.57	21	21	65882674	83708277
712	1	P30740	P30740	Leukocyte elastase inhibitor	SERPINB1	Homo sapiens	42.741	22	21	56458345	351220131
713	1	P30876	P30876	DNA-directed RNA polymerase II subunit RPB2	POLR2B	Homo sapiens	133.897	21	21	5205677	2517118
714	1	P33121	P33121	Long-chain-fatty-acid--CoA ligase 1	ACSL1	Homo sapiens	77.945	22	21	6243923	45036972
715	1	P33991	P33991	DNA replication licensing factor MCM4	MCM4	Homo sapiens	96.56	21	21	4915363	1184371
716	1	P35611	P35611	Alpha-adducin	ADD1	Homo sapiens	80.956	21	21	15400525	3103963
717	1	P35998	P35998	26S proteasome regulatory subunit 7	PSMC2	Homo sapiens	48.634	21	21	20607112	20251242
718	1	P42285	P42285	Exosome RNA helicase MTR4	MTREX	Homo sapiens	117.807	21	21	3784840	2523515
719	1	P46379	P46379	Large proline-rich protein BAG6	BAG6	Homo sapiens	119.41	21	21	17975168	13865015
720	1	P48449	P48449	Lanosterol synthase	LSS	Homo sapiens	83.308	21	21	7199692	1776798
721	1	P48506	P48506	Glutamate--cysteine ligase catalytic subunit	GCLC	Homo sapiens	72.768	21	21	9506646	2869924
722	1	P48643	P48643	T-complex protein 1 subunit epsilon	CCT5	Homo sapiens	59.672	21	21	33491561	20646480
723	1	P50395	P50395	Rab GDP dissociation inhibitor beta	GDI2	Homo sapiens	50.666	29	21	105944891	58566030
724	1	P50552	P50552	Vasodilator-stimulated phosphoprotein	VASP	Homo sapiens	39.83	21	21	35258856	51910074
725	1	P50991	P50991	T-complex protein 1 subunit delta	CCT4	Homo sapiens	57.924	21	21	19988291	10988118
726	1	P51659	P51659	Peroxisomal multifunctional enzyme type 2	HSD17B4	Homo sapiens	79.686	21	21	28644434	21066028
727	1	P61978	P61978	Heterogeneous nuclear ribonucleoprotein K	HNRNPK	Homo sapiens	50.979	21	21	59863499	30984455
728	1	P62191	P62191	26S proteasome regulatory subunit 4	PSMC1	Homo sapiens	49.185	21	21	14716606	7430930
729	1	P78347	P78347	General transcription factor II-I	GTF2I	Homo sapiens	112.418	23	21	7110870	1808245
730	1	Q01780	Q01780	Exosome component 10	EXOSC10	Homo sapiens	100.833	21	21	2900286	1026338
731	1	Q01968	Q01968	Inositol polyphosphate 5-phosphatase OCRL	OCRL	Homo sapiens	104.204	22	21	3940736	885146
732	1	Q12789	Q12789	General transcription factor 3C polypeptide 1	GTF3C1	Homo sapiens	238.879	21	21	2124775	365474
733	1	Q12797	Q12797	Aspartyl/asparaginyl beta-hydroxylase	ASPH	Homo sapiens	85.863	21	21	16106899	9476654
734	1	Q13616	Q13616	Cullin-1	CUL1	Homo sapiens	89.679	21	21	6319563	2356354
735	1	Q14766	Q14766	Latent-transforming growth factor beta-binding protein 1	LTBP1	Homo sapiens	186.797	21	21	2371282	1977580
736	1	Q15046	Q15046	Lysine--tRNA ligase	KARS1	Homo sapiens	68.048	21	21	12334572	8017561
737	1	Q15459	Q15459	Splicing factor 3A subunit 1	SF3A1	Homo sapiens	88.887	21	21	8594862	3217483
738	1	Q15833	Q15833	Syntaxin-binding protein 2	STXBP2	Homo sapiens	66.453	21	21	3389789	14991244
739	1	Q5H9R7	Q5H9R7	Serine/threonine-protein phosphatase 6 regulatory subunit 3	PPP6R3	Homo sapiens	97.671	21	21	5185377	1425506
740	1	Q68CZ2	Q68CZ2	Tensin-3	TNS3	Homo sapiens	155.268	21	21	5016889	2865275
741	1	Q6P179	Q6P179	Endoplasmic reticulum aminopeptidase 2	ERAP2	Homo sapiens	110.462	21	21	4920831	2412296
742	1	Q6ZMZ3	Q6ZMZ3	Nesprin-3	SYNE3	Homo sapiens	112.219	21	21	2261426	887908
743	1	Q86SQ0	Q86SQ0	Pleckstrin homology-like domain family B member 2	PHLDB2	Homo sapiens	142.161	21	21	6185573	88787
744	1	Q86XP3	Q86XP3	ATP-dependent RNA helicase DDX42	DDX42	Homo sapiens	102.978	21	21	6972543	2952186
745	1	Q8IVT2	Q8IVT2	Mitotic interactor and substrate of PLK1	MISP	Homo sapiens	75.358	21	21	7970417	50138
746	1	Q8IY17	Q8IY17	Patatin-like phospholipase domain-containing protein 6	PNPLA6	Homo sapiens	150.958	21	21	6096429	1683487
747	1	Q8NI08	Q8NI08	Nuclear receptor coactivator 7	NCOA7	Homo sapiens	106.165	21	21	2138866	1033710
748	1	Q93034	Q93034	Cullin-5	CUL5	Homo sapiens	90.956	21	21	9772634	2603056
749	1	Q96CV9	Q96CV9	Optineurin	OPTN	Homo sapiens	65.922	21	21	6771713	1592437
750	1	Q96HP0	Q96HP0	Dedicator of cytokinesis protein 6	DOCK6	Homo sapiens	229.562	21	21	1607566	120457
751	1	Q96Q05	Q96Q05	Trafficking protein particle complex subunit 9	TRAPPC9	Homo sapiens	128.532	21	21	2165975	2289288
752	1	Q96TA1	Q96TA1	Protein Niban 2	NIBAN2	Homo sapiens	84.137	21	21	18790006	6165723
753	1	Q99459	Q99459	Cell division cycle 5-like protein	CDC5L	Homo sapiens	92.252	21	21	14060075	1203363
754	1	Q9BQS7	Q9BQS7	Hephaestin	HEPH	Homo sapiens	130.451	21	21	6639049	0
755	1	Q9H2K8	Q9H2K8	Serine/threonine-protein kinase TAO3	TAOK3	Homo sapiens	105.405	24	21	2094542	3676756
756	1	Q9H4G0	Q9H4G0	Band 4.1-like protein 1	EPB41L1	Homo sapiens	98.504	21	21	6935312	109940
757	1	Q9HCH5	Q9HCH5	Synaptotagmin-like protein 2	SYTL2	Homo sapiens	104.93	21	21	3789779	0

758	1	Q9NTJ5	Q9NTJ5	Phosphatidylinositol-3-phosphatase SAC1	SACM1L	Homo sapiens	66.968	21	21	8421526	7965129
759	1	Q9P2R3	Q9P2R3	Rabankyrin-5	ANKFY1	Homo sapiens	128.399	21	21	4921699	2842954
760	1	Q9UPY3	Q9UPY3	Endoribonuclease Dicer	DICER1	Homo sapiens	218.686	21	21	2041486	1686094
761	1	Q9Y2H5	Q9Y2H5	Pleckstrin homology domain-containing family A member 6	PLEKHA6	Homo sapiens	117.13	21	21	5665281	0
762	1	Q9Y2W1	Q9Y2W1	Thyroid hormone receptor-associated protein 3	THRAP3	Homo sapiens	108.667	22	21	11733148	4693056
763	1	Q9Y305	Q9Y305	Acyl-coenzyme A thioesterase 9, mitochondrial	ACOT9	Homo sapiens	49.902	21	21	15831863	4767830
764	1	O00231	O00231	26S proteasome non-ATPase regulatory subunit 11	PSMD11	Homo sapiens	47.464	20	20	11964552	9682422
765	1	O14639	O14639	Actin-binding LIM protein 1	ABLIM1	Homo sapiens	87.689	20	20	4517964	0
766	1	O15294	O15294	UDP-N-acetylglucosamine--peptide N-acetylglucosaminyltransferase 110 kDa subunit	OGT	Homo sapiens	116.925	20	20	3610292	1933268
767	1	O15438	O15438	ATP-binding cassette sub-family C member 3	ABCC3	Homo sapiens	169.344	20	20	4243020	662497
768	1	O43815	O43815	Striatin	STRN	Homo sapiens	86.134	20	20	4913632	2510041
769	1	O60231	O60231	Pre-mRNA-splicing factor ATP-dependent RNA helicase DHX16	DHX16	Homo sapiens	119.264	20	20	2258313	444978
770	1	O60462	O60462	Neuropilin-2	NRP2	Homo sapiens	104.834	20	20	6227468	2334394
771	1	O60488	O60488	Long-chain-fatty-acid--CoA ligase 4	ACSL4	Homo sapiens	79.189	20	20	2398865	5829887
772	1	O95347	O95347	Structural maintenance of chromosomes protein 2	SMC2	Homo sapiens	135.661	20	20	1885674	2375332
773	1	O95793	O95793	Double-stranded RNA-binding protein Staufen homolog 1	STAU1	Homo sapiens	63.183	20	20	6793536	1287884
774	1	P00352	P00352	Aldehyde dehydrogenase 1A1	ALDH1A1	Homo sapiens	54.862	21	20	170535442	6901383
775	1	P02748	P02748	Complement component C9	C9	Homo sapiens	63.175	23	20	36332328	187825855
776	1	P04424	P04424	Argininosuccinate lyase	ASL	Homo sapiens	51.66	20	20	14467746	5307252
777	1	P05106	P05106	Integrin beta-3	ITGB3	Homo sapiens	87.058	20	20	8271855	10924051
778	1	P13010	P13010	X-ray repair cross-complementing protein 5	XRCC5	Homo sapiens	82.705	20	20	36381907	22915833
779	1	P16615	P16615	Sarcoplasmic/endoplasmic reticulum calcium ATPase 2	ATP2A2	Homo sapiens	114.757	31	20	19503685	13554522
780	1	P19838	P19838	Nuclear factor NF-kappa-B p105 subunit	NFKB1	Homo sapiens	105.359	20	20	3913092	5993239
781	1	P19878	P19878	Neutrophil cytosol factor 2	NCF2	Homo sapiens	59.761	20	20	3163779	95497501
782	1	P22033	P22033	Methylmalonyl-CoA mutase, mitochondrial	MMUT	Homo sapiens	83.135	20	20	8214762	1204765
783	1	P23246	P23246	Splicing factor, proline- and glutamine-rich	SFPQ	Homo sapiens	76.151	20	20	31345642	45280517
784	1	P24557	P24557	Thromboxane-A synthase	TBXAS1	Homo sapiens	60.519	20	20	3068755	5472807
785	1	P25205	P25205	DNA replication licensing factor MCM3	MCM3	Homo sapiens	90.981	20	20	2555937	1448792
786	1	P33527	P33527	Multidrug resistance-associated protein 1	ABCC1	Homo sapiens	171.593	21	20	3150529	1838050
787	1	P35556	P35556	Fibrillin-2	FBN2	Homo sapiens	314.781	20	20	3736289	456235
788	1	P38606	P38606	V-type proton ATPase catalytic subunit A	ATP6V1A	Homo sapiens	68.301	20	20	12525022	12250773
789	1	P46019	P46019	Phosphorylase b kinase regulatory subunit alpha, liver isoform	PHKA2	Homo sapiens	138.409	20	20	1423928	3423412
790	1	P46100	P46100	Transcriptional regulator ATRX	ATRX	Homo sapiens	282.591	20	20	1812392	298996
791	1	P48735	P48735	Isocitrate dehydrogenase [NADP], mitochondrial	IDH2	Homo sapiens	50.911	20	20	75684648	24896015
792	1	P49411	P49411	Elongation factor Tu, mitochondrial	TUFM	Homo sapiens	49.876	20	20	54942309	18170424
793	1	P49756	P49756	RNA-binding protein 25	RBM25	Homo sapiens	100.186	20	20	6169189	3721975
794	1	P49959	P49959	Double-strand break repair protein MRE11	MRE11	Homo sapiens	80.594	20	20	4528932	2074502
795	1	P50454	P50454	Serpin H1	SERPINH1	Homo sapiens	46.441	20	20	31119147	38415207
796	1	P50570	P50570	Dynamin-2	DNM2	Homo sapiens	98.067	30	20	12377823	12765921
797	1	P60228	P60228	Eukaryotic translation initiation factor 3 subunit E	EIF3E	Homo sapiens	52.223	20	20	16032260	6850726
798	1	P62195	P62195	26S proteasome regulatory subunit 8	PSMC5	Homo sapiens	45.624	21	20	9076112	7530462
799	1	Q00722	Q00722	1-phosphatidylinositol 4,5-bisphosphate phosphodiesterase beta-2	PLCB2	Homo sapiens	134.026	20	20	553074	3300284
800	1	Q02108	Q02108	Guanylate cyclase soluble subunit alpha-1	GUCY1A1	Homo sapiens	77.452	20	20	4927506	94973
801	1	Q02153	Q02153	Guanylate cyclase soluble subunit beta-1	GUCY1B1	Homo sapiens	70.514	20	20	4182663	179016
802	1	Q02252	Q02252	Methylmalonate-semialdehyde dehydrogenase [acylating], mitochondrial	ALDH6A1	Homo sapiens	57.841	20	20	20606562	3076830
803	1	Q12769	Q12769	Nuclear pore complex protein Nup160	NUP160	Homo sapiens	162.123	20	20	2945938	2069792
804	1	Q13017	Q13017	Rho GTPase-activating protein 5	ARHGAP5	Homo sapiens	172.461	20	20	2155369	0

805	1	Q13451	Q13451	Peptidyl-prolyl cis-trans isomerase FKBP5	FKBP5	Homo sapiens	51.214	20	20	8672738	11580961
806	1	Q13496	Q13496	Myotubularin	MTM1	Homo sapiens	69.931	20	20	5492174	999641
807	1	Q13586	Q13586	Stromal interaction molecule 1	STIM1	Homo sapiens	77.423	20	20	5493152	4564826
808	1	Q13596	Q13596	Sorting nexin-1	SNX1	Homo sapiens	59.069	20	20	15833572	7267228
809	1	Q14166	Q14166	Tubulin--tyrosine ligase-like protein 12	TTLL12	Homo sapiens	74.404	20	20	8381487	10042889
810	1	Q15642	Q15642	Cdc42-interacting protein 4	TRIP10	Homo sapiens	68.351	20	20	5770113	1789739
811	1	Q16555	Q16555	Dihydropyrimidinase-related protein 2	DPYSL2	Homo sapiens	62.294	23	20	87135148	14255430
812	1	Q16666	Q16666	Gamma-interferon-inducible protein 16	IFI16	Homo sapiens	88.258	22	20	7127059	9978197
813	1	Q2M2I8	Q2M2I8	AP2-associated protein kinase 1	AAK1	Homo sapiens	103.886	21	20	4210456	2117480
814	1	Q32MZ4	Q32MZ4	Leucine-rich repeat flightless-interacting protein 1	LRRFIP1	Homo sapiens	89.253	21	20	10341532	7993684
815	1	Q4KMQ2	Q4KMQ2	Anoctamin-6	ANO6	Homo sapiens	106.167	20	20	3685359	1122373
816	1	Q66K74	Q66K74	Microtubule-associated protein 1S	MAP1S	Homo sapiens	112.213	20	20	5846905	4535550
817	1	Q6P2E9	Q6P2E9	Enhancer of mRNA-decapping protein 4	EDC4	Homo sapiens	151.664	20	20	5477674	2186766
818	1	Q6ZRV2	Q6ZRV2	Protein FAM83H	FAM83H	Homo sapiens	127.123	20	20	2809165	396209
819	1	Q6ZS30	Q6ZS30	Neurobeachin-like protein 1	NBEAL1	Homo sapiens	307.243	20	20	1766224	231568
820	1	Q86SF2	Q86SF2	N-acetylgalactosaminyltransferase 7	GALNT7	Homo sapiens	75.391	20	20	5690355	2112118
821	1	Q86YP4	Q86YP4	Transcriptional repressor p66-alpha	GATAD2A	Homo sapiens	68.063	22	20	2874467	457407
822	1	Q8IWV7	Q8IWV7	E3 ubiquitin-protein ligase UBR1	UBR1	Homo sapiens	200.213	20	20	2681446	298041
823	1	Q8N1G4	Q8N1G4	Leucine-rich repeat-containing protein 47	LRRC47	Homo sapiens	63.473	20	20	11829194	3295527
824	1	Q8N3P4	Q8N3P4	Vacuolar protein sorting-associated protein 8 homolog	VPS8	Homo sapiens	161.754	20	20	2481345	1711494
825	1	Q8NDI1	Q8NDI1	EH domain-binding protein 1	EHBP1	Homo sapiens	140.019	20	20	2377946	189071
826	1	Q8TD19	Q8TD19	Serine/threonine-protein kinase Nek9	NEK9	Homo sapiens	107.169	20	20	6360510	4085081
827	1	Q8TER5	Q8TER5	Rho guanine nucleotide exchange factor 40	ARHGEF40	Homo sapiens	164.66	20	20	1884889	188168
828	1	Q8WTW3	Q8WTW3	Conserved oligomeric Golgi complex subunit 1	COG1	Homo sapiens	108.982	20	20	6379552	1040354
829	1	Q92625	Q92625	Ankyrin repeat and SAM domain-containing protein 1A	ANKS1A	Homo sapiens	123.11	20	20	3557919	2336973
830	1	Q93084	Q93084	Sarcoplasmic/endoplasmic reticulum calcium ATPase 3	ATP2A3	Homo sapiens	109.257	22	20	9505851	15792261
831	1	Q96F07	Q96F07	Cytoplasmic FMR1-interacting protein 2	CYFIP2	Homo sapiens	148.402	20	20	785267	6020721
832	1	Q96M96	Q96M96	FYVE, RhoGEF and PH domain-containing protein 4	FGD4	Homo sapiens	86.63	20	20	2623930	1638074
833	1	Q99707	Q99707	Methionine synthase	MTR	Homo sapiens	140.529	20	20	2014389	0
834	1	Q9BZH6	Q9BZH6	WD repeat-containing protein 11	WDR11	Homo sapiens	136.687	20	20	2765615	2009070
835	1	Q9H223	Q9H223	EH domain-containing protein 4	EHD4	Homo sapiens	61.177	21	20	20930049	8503348
836	1	Q9H6S3	Q9H6S3	Epidermal growth factor receptor kinase substrate 8-like protein 2	EPS8L2	Homo sapiens	80.621	20	20	4157631	824731
837	1	Q9H9A6	Q9H9A6	Leucine-rich repeat-containing protein 40	LRRC40	Homo sapiens	68.25	20	20	6650311	2240698
838	1	Q9P260	Q9P260	RAB11-binding protein RELCH	RELCH	Homo sapiens	134.633	20	20	1640803	142324
839	1	Q9UEY8	Q9UEY8	Gamma-adducin	ADD3	Homo sapiens	79.157	20	20	16534731	3727478
840	1	Q9ULC5	Q9ULC5	Long-chain-fatty-acid--CoA ligase 5	ACSL5	Homo sapiens	75.991	21	20	11711335	2108529
841	1	Q9Y217	Q9Y217	Myotubularin-related protein 6	MTMR6	Homo sapiens	71.968	20	20	3325375	1266023
842	1	Q9Y2D5	Q9Y2D5	A-kinase anchor protein 2	AKAP2	Homo sapiens	94.662	20	20	5997540	1159141
843	1	Q9Y2E4	Q9Y2E4	Disco-interacting protein 2 homolog C	DIP2C	Homo sapiens	170.769	20	20	3070830	0
844	1	Q9Y2Z0	Q9Y2Z0	Protein SGT1 homolog	SUGT1	Homo sapiens	41.025	20	20	13571062	5392180
845	1	Q9Y4D8	Q9Y4D8	Probable E3 ubiquitin-protein ligase HECTD4	HECTD4	Homo sapiens	439.353	20	20	1241962	29873
846	1	Q9Y4F1	Q9Y4F1	FERM, ARHGEF and pleckstrin domain-containing protein 1	FARP1	Homo sapiens	118.635	20	20	9445465	437500
847	1	Q9Y4P3	Q9Y4P3	Transducin beta-like protein 2	TBL2	Homo sapiens	49.8	20	20	4374319	2275040
848	1	Q9Y5K6	Q9Y5K6	CD2-associated protein	CD2AP	Homo sapiens	71.452	20	20	10366827	2927821
849	1	Q9Y6D6	Q9Y6D6	Brefeldin A-inhibited guanine nucleotide-exchange protein 1	ARFGEF1	Homo sapiens	208.77	31	20	2736100	1931033
850	1	Q9Y6Y8	Q9Y6Y8	SEC23-interacting protein	SEC23IP	Homo sapiens	111.077	20	20	8430396	5305070
851	1	E9PAV3	E9PAV3	Nascent polypeptide-associated complex subunit alpha, muscle-specific form	NACA	Homo sapiens	205.426	20	19	22104458	6276908

852	1	O00339	O00339	Matrilin-2	MATN2	Homo sapiens	106.839	19	19	14626773	0
853	1	O00461	O00461	Golgi integral membrane protein 4	GOLIM4	Homo sapiens	81.881	19	19	4721508	715052
854	1	O15042	O15042	U2 snRNP-associated SURP motif-containing protein	U2SURP	Homo sapiens	118.29	19	19	4288268	1633936
855	1	O15085	O15085	Rho guanine nucleotide exchange factor 11	ARHGEF11	Homo sapiens	167.706	19	19	16213049	2364136
856	1	O60504	O60504	Vinexin	SORBS3	Homo sapiens	75.341	19	19	10208539	316275
857	1	O94822	O94822	E3 ubiquitin-protein ligase listerin	LTN1	Homo sapiens	200.553	19	19	1421381	1757464
858	1	O95573	O95573	Fatty acid CoA ligase Acsl3	ACSL3	Homo sapiens	80.422	19	19	6925182	4508438
859	1	O95757	O95757	Heat shock 70 kDa protein 4L	HSPA4L	Homo sapiens	94.515	19	19	2724744	2272402
860	1	O95817	O95817	BAG family molecular chaperone regulator 3	BAG3	Homo sapiens	61.595	19	19	22992405	793409
861	1	P04217	P04217	Alpha-1B-glycoprotein	A1BG	Homo sapiens	54.252	19	19	19201746	39401064
862	1	P09917	P09917	Polyunsaturated fatty acid 5-lipoxygenase	ALOX5	Homo sapiens	77.982	19	19	861563	14168897
863	1	P11387	P11387	DNA topoisomerase 1	TOP1	Homo sapiens	90.726	22	19	9306141	4487657
864	1	P15311	P15311	Ezrin	EZR	Homo sapiens	69.414	20	19	22277229	15952261
865	1	P19823	P19823	Inter-alpha-trypsin inhibitor heavy chain H2	ITIH2	Homo sapiens	106.465	19	19	31070867	78235714
866	1	P20042	P20042	Eukaryotic translation initiation factor 2 subunit 2	EIF2S2	Homo sapiens	38.389	19	19	15565922	7153217
867	1	P20742	P20742	Pregnancy zone protein	PZP	Homo sapiens	163.866	19	19	5343775	6728922
868	1	P23141	P23141	Liver carboxylesterase 1	CES1	Homo sapiens	62.523	20	19	59349887	7046997
869	1	P23469	P23469	Receptor-type tyrosine-protein phosphatase epsilon	PTPRE	Homo sapiens	80.641	19	19	891472	5620518
870	1	P26006	P26006	Integrin alpha-3	ITGA3	Homo sapiens	116.612	19	19	8184598	4393853
871	1	P26196	P26196	Probable ATP-dependent RNA helicase DDX6	DDX6	Homo sapiens	54.418	19	19	15775768	3729502
872	1	P26358	P26358	DNA (cytosine-5)-methyltransferase 1	DNMT1	Homo sapiens	183.167	19	19	1647067	684754
873	1	P27797	P27797	Calreticulin	CALR	Homo sapiens	48.141	19	19	106868220	96142175
874	1	P49916	P49916	DNA ligase 3	LIG3	Homo sapiens	112.907	19	19	2447045	1873092
875	1	P50995	P50995	Annexin A11	ANXA11	Homo sapiens	54.39	20	19	28071880	34329797
876	1	P52701	P52701	DNA mismatch repair protein Msh6	MSH6	Homo sapiens	152.789	19	19	1913014	103551
877	1	P56199	P56199	Integrin alpha-1	ITGA1	Homo sapiens	130.849	19	19	41348828	2894163
878	1	Q02790	Q02790	Peptidyl-prolyl cis-trans isomerase FKBP4	FKBP4	Homo sapiens	51.805	19	19	21331857	13687708
879	1	Q05209	Q05209	Tyrosine-protein phosphatase non-receptor type 12	PTPN12	Homo sapiens	88.109	19	19	2060424	5161739
880	1	Q05397	Q05397	Focal adhesion kinase 1	PTK2	Homo sapiens	119.234	19	19	4917439	473807
881	1	Q07866	Q07866	Kinesin light chain 1	KLC1	Homo sapiens	65.31	27	19	9433890	4159047
882	1	Q10570	Q10570	Cleavage and polyadenylation specificity factor subunit 1	CPSF1	Homo sapiens	160.886	19	19	3106724	1583824
883	1	Q12864	Q12864	Cadherin-17	CDH17	Homo sapiens	92.22	19	19	87420290	451288
884	1	Q12959	Q12959	Disks large homolog 1	DLG1	Homo sapiens	100.456	23	19	6743202	1137689
885	1	Q13136	Q13136	Liprin-alpha-1	PPFIA1	Homo sapiens	135.779	23	19	2909407	1141279
886	1	Q13201	Q13201	Multimerin-1	MMRN1	Homo sapiens	138.114	19	19	917689	2155739
887	1	Q13393	Q13393	Phospholipase D1	PLD1	Homo sapiens	124.187	19	19	748762	3510797
888	1	Q13546	Q13546	Receptor-interacting serine/threonine-protein kinase 1	RIPK1	Homo sapiens	75.933	19	19	4125749	2714458
889	1	Q13564	Q13564	NEDD8-activating enzyme E1 regulatory subunit	NAE1	Homo sapiens	60.247	19	19	8665060	3527603
890	1	Q13976	Q13976	cGMP-dependent protein kinase 1	PRKG1	Homo sapiens	76.365	19	19	9173432	464771
891	1	Q14254	Q14254	Flotillin-2	FLOT2	Homo sapiens	47.064	19	19	11083548	28980771
892	1	Q14517	Q14517	Protocadherin Fat 1	FAT1	Homo sapiens	506.28	19	19	1565543	284539
893	1	Q15019	Q15019	Septin-2	SEPTIN2	Homo sapiens	41.488	19	19	22603759	3961828
894	1	Q15233	Q15233	Non-POU domain-containing octamer-binding protein	NONO	Homo sapiens	54.23	19	19	19092009	8612000
895	1	Q15262	Q15262	Receptor-type tyrosine-protein phosphatase kappa	PTPRK	Homo sapiens	162.105	21	19	2285872	95953
896	1	Q15436	Q15436	Protein transport protein Sec23A	SEC23A	Homo sapiens	86.16	22	19	9849582	5508618
897	1	Q16134	Q16134	Electron transfer flavoprotein-ubiquinone oxidoreductase, mitochondrial	ETFDH	Homo sapiens	68.494	19	19	11738078	2861529
898	1	Q16513	Q16513	Serine/threonine-protein kinase N2	PKN2	Homo sapiens	112.034	19	19	4367972	841107
899	1	Q16610	Q16610	Extracellular matrix protein 1	ECM1	Homo sapiens	60.675	19	19	5751073	27870717

900	1	Q562E7	Q562E7	WD repeat-containing protein 81	WDR81	Homo sapiens	211.7	19	19	2442650	1762347
901	1	Q5GLZ8	Q5GLZ8	Probable E3 ubiquitin-protein ligase HERC4	HERC4	Homo sapiens	118.563	19	19	5451789	2799472
902	1	Q5JTZ9	Q5JTZ9	Alanine--tRNA ligase, mitochondrial	AARS2	Homo sapiens	107.341	19	19	5230032	499074
903	1	Q6WKZ4	Q6WKZ4	Rab11 family-interacting protein 1	RAB11FIP1	Homo sapiens	137.168	21	19	990073	3817936
904	1	Q6XZF7	Q6XZF7	Dynamin-binding protein	DNMBP	Homo sapiens	177.349	19	19	1608340	244237
905	1	Q6ZMI0	Q6ZMI0	Protein phosphatase 1 regulatory subunit 21	PPP1R21	Homo sapiens	88.315	19	19	3341114	1039458
906	1	Q7KZI7	Q7KZI7	Serine/threonine-protein kinase MARK2	MARK2	Homo sapiens	87.911	24	19	2552113	2867823
907	1	Q86VB7	Q86VB7	Scavenger receptor cysteine-rich type 1 protein M130	CD163	Homo sapiens	125.452	19	19	5189361	8841447
908	1	Q86VN1	Q86VN1	Vacuolar protein-sorting-associated protein 36	VPS36	Homo sapiens	43.816	19	19	6020998	3052914
909	1	Q86WG5	Q86WG5	Myotubularin-related protein 13	SBF2	Homo sapiens	208.467	19	19	2076037	107075
910	1	Q8IX12	Q8IX12	Cell division cycle and apoptosis regulator protein 1	CCAR1	Homo sapiens	132.823	19	19	4726933	1965391
911	1	Q8N122	Q8N122	Regulatory-associated protein of mTOR	RPTOR	Homo sapiens	149.038	19	19	1804242	284950
912	1	Q8N8A2	Q8N8A2	Serine/threonine-protein phosphatase 6 regulatory ankyrin repeat subunit B	ANKRD44	Homo sapiens	107.605	20	19	1702875	4107326
913	1	Q8NBF2	Q8NBF2	NHL repeat-containing protein 2	NHLRC2	Homo sapiens	79.446	19	19	12503901	3779508
914	1	Q8NEU8	Q8NEU8	DCC-interacting protein 13-beta	APPL2	Homo sapiens	74.493	19	19	6489402	2193123
915	1	Q8TBA6	Q8TBA6	Golgin subfamily A member 5	GOLGA5	Homo sapiens	83.025	19	19	2545748	563136
916	1	Q8WWQ0	Q8WWQ0	PH-interacting protein	PHIP	Homo sapiens	206.693	20	19	2327260	325158
917	1	Q92609	Q92609	TBC1 domain family member 5	TBC1D5	Homo sapiens	89.004	19	19	2144532	1537035
918	1	Q92619	Q92619	Rho GTPase-activating protein 45	ARHGAP45	Homo sapiens	124.616	19	19	1368326	10730126
919	1	Q92696	Q92696	Geranylgeranyl transferase type-2 subunit alpha	RABGGTA	Homo sapiens	65.075	19	19	4028298	3386871
920	1	Q92805	Q92805	Golgin subfamily A member 1	GOLGA1	Homo sapiens	88.185	19	19	2129425	234824
921	1	Q93052	Q93052	Lipoma-preferred partner	LPP	Homo sapiens	65.746	19	19	201197196	2835778
922	1	Q96AQ6	Q96AQ6	Pre-B-cell leukemia transcription factor-interacting protein 1	PBXIP1	Homo sapiens	80.642	19	19	7694054	3234745
923	1	Q96EK7	Q96EK7	Constitutive coactivator of peroxisome proliferator-activated receptor gamma	FAM120B	Homo sapiens	103.785	19	19	3001652	653708
924	1	Q96G03	Q96G03	Phosphopentomutase	PGM2	Homo sapiens	68.283	19	19	23235680	12807811
925	1	Q96HC4	Q96HC4	PDZ and LIM domain protein 5	PDLIM5	Homo sapiens	63.946	19	19	19307242	3995353
926	1	Q96JC1	Q96JC1	Vam6/Vps39-like protein	VPS39	Homo sapiens	101.812	19	19	2050894	1930913
927	1	Q96JI7	Q96JI7	Spatacsin	SPG11	Homo sapiens	278.872	19	19	714391	1000727
928	1	Q96L91	Q96L91	E1A-binding protein p400	EP400	Homo sapiens	343.493	19	19	1448288	94931
929	1	Q96RP9	Q96RP9	Elongation factor G, mitochondrial	GFM1	Homo sapiens	83.474	19	19	9805284	1805466
930	1	Q96ST3	Q96ST3	Paired amphipathic helix protein Sin3a	SIN3A	Homo sapiens	145.177	19	19	2338569	710234
931	1	Q9BUJ2	Q9BUJ2	Heterogeneous nuclear ribonucleoprotein U-like protein 1	HNRNPUL1	Homo sapiens	95.742	19	19	9544429	6386720
932	1	Q9BXJ9	Q9BXJ9	N-alpha-acetyltransferase 15, NatA auxiliary subunit	NAA15	Homo sapiens	101.275	23	19	6227983	3223852
933	1	Q9BXX0	Q9BXX0	EMILIN-2	EMILIN2	Homo sapiens	115.686	19	19	3507764	1935401
934	1	Q9H2P0	Q9H2P0	Activity-dependent neuroprotector homeobox protein	ADNP	Homo sapiens	123.565	19	19	3464821	368765
935	1	Q9H307	Q9H307	Pinin	PNN	Homo sapiens	81.629	19	19	4753590	1496906
936	1	Q9H583	Q9H583	HEAT repeat-containing protein 1	HEATR1	Homo sapiens	242.375	19	19	1467402	210048
937	1	Q9HAU0	Q9HAU0	Pleckstrin homology domain-containing family A member 5	PLEKHA5	Homo sapiens	127.465	19	19	3219877	70051
938	1	Q9NR12	Q9NR12	PDZ and LIM domain protein 7	PDLIM7	Homo sapiens	49.847	19	19	287595071	6402654
939	1	Q9NV70	Q9NV70	Exocyst complex component 1	EXOC1	Homo sapiens	101.984	19	19	3655181	2105739
940	1	Q9NXF1	Q9NXF1	Testis-expressed protein 10	TEX10	Homo sapiens	105.674	19	19	1762164	1087187
941	1	Q9NXV6	Q9NXV6	CDKN2A-interacting protein	CDKN2AIP	Homo sapiens	61.126	19	19	2747640	2176388
942	1	Q9P266	Q9P266	Junctional cadherin 5-associated protein	JCAD	Homo sapiens	148.354	19	19	2998123	45052
943	1	Q9UBG0	Q9UBG0	C-type mannose receptor 2	MRC2	Homo sapiens	166.676	19	19	6923288	1555387
944	1	Q9UKV8	Q9UKV8	Protein argonaute-2	AGO2	Homo sapiens	97.209	26	19	5130020	991600
945	1	Q9UPU7	Q9UPU7	TBC1 domain family member 2B	TBC1D2B	Homo sapiens	109.883	19	19	2235887	2081284

946	1	Q9Y223	Q9Y223	Bifunctional UDP-N-acetylglucosamine 2-epimerase/N-acetylmannosamine kinase	GNE	Homo sapiens	79.275	19	19	7897543	1191440
947	1	Q9Y450	Q9Y450	HBS1-like protein	HBS1L	Homo sapiens	75.475	19	19	3731770	2027513
948	1	Q9Y4F5	Q9Y4F5	Centrosomal protein of 170 kDa protein B	CEP170B	Homo sapiens	171.689	19	19	2050360	13575
949	1	Q9Y4W6	Q9Y4W6	AFG3-like protein 2	AFG3L2	Homo sapiens	88.584	19	19	14230122	8019313
950	1	Q9Y6D5	Q9Y6D5	Brefeldin A-inhibited guanine nucleotide-exchange protein 2	ARFGEF2	Homo sapiens	202.043	19	19	2785290	824344
951	1	Q9Y6N5	Q9Y6N5	Sulfide:quinone oxidoreductase, mitochondrial	SQOR	Homo sapiens	49.96	19	19	71846247	86455357
952	1	A1L390	A1L390	Pleckstrin homology domain-containing family G member 3	PLEKHG3	Homo sapiens	134.415	18	18	1736963	291876
953	1	O00116	O00116	Alkyldihydroxyacetonephosphate synthase, peroxisomal	AGPS	Homo sapiens	72.909	18	18	5217973	6332447
954	1	O00471	O00471	Exocyst complex component 5	EXOC5	Homo sapiens	81.85	18	18	3815426	2100819
955	1	O00534	O00534	von Willebrand factor A domain-containing protein 5A	VWA5A	Homo sapiens	86.492	18	18	13107918	2122238
956	1	O15144	O15144	Actin-related protein 2/3 complex subunit 2	ARPC2	Homo sapiens	34.332	18	18	45214589	48529588
957	1	O15394	O15394	Neural cell adhesion molecule 2	NCAM2	Homo sapiens	93.049	18	18	3286457	0
958	1	O15439	O15439	ATP-binding cassette sub-family C member 4	ABCC4	Homo sapiens	149.528	18	18	4250155	844813
959	1	O15460	O15460	Prolyl 4-hydroxylase subunit alpha-2	P4HA2	Homo sapiens	60.901	18	18	3677375	6279126
960	1	O43157	O43157	Plexin-B1	PLXNB1	Homo sapiens	232.302	19	18	2297115	0
961	1	O43426	O43426	Synaptojanin-1	SYNJ1	Homo sapiens	173.105	18	18	2275318	2277494
962	1	O43615	O43615	Mitochondrial import inner membrane translocase subunit TIM44	TIMM44	Homo sapiens	51.356	18	18	7433681	2046498
963	1	O60264	O60264	SWI/SNF-related matrix-associated actin-dependent regulator of chromatin subfamily A member 5	SMARCA5	Homo sapiens	121.907	25	18	4957847	2991502
964	1	O60749	O60749	Sorting nexin-2	SNX2	Homo sapiens	58.471	20	18	28256213	10689415
965	1	O75146	O75146	Huntingtin-interacting protein 1-related protein	HIP1R	Homo sapiens	119.39	18	18	2338994	242980
966	1	O75150	O75150	E3 ubiquitin-protein ligase BRE1B	RNF40	Homo sapiens	113.682	22	18	13907081	8154854
967	1	O94804	O94804	Serine/threonine-protein kinase 10	STK10	Homo sapiens	112.136	18	18	1705668	7147250
968	1	P02730	P02730	Band 3 anion transport protein	SLC4A1	Homo sapiens	101.795	18	18	29365044	60332596
969	1	P05091	P05091	Aldehyde dehydrogenase, mitochondrial	ALDH2	Homo sapiens	56.381	18	18	81910769	44331284
970	1	P08237	P08237	ATP-dependent 6-phosphofructokinase, muscle type	PFKM	Homo sapiens	85.184	18	18	14084789	2418201
971	1	P08519	P08519	Apolipoprotein(a)	LPA	Homo sapiens	226.55	18	18	3404439	9559704
972	1	P08631	P08631	Tyrosine-protein kinase HCK	HCK	Homo sapiens	59.601	18	18	809111	16047009
973	1	P08758	P08758	Annexin A5	ANXA5	Homo sapiens	35.937	18	18	173045460	46399941
974	2	P0DMV8;P0DMV9	P0DMV8	Heat shock 70 kDa protein 1A	HSPA1A	Homo sapiens	70.056	21	18	143849182	55615023
975	1	P10645	P10645	Chromogranin-A	CHGA	Homo sapiens	50.689	18	18	36942455	143739
976	1	P11171	P11171	Protein 4.1	EPB41	Homo sapiens	97.018	18	18	9183792	9113415
977	1	P13797	P13797	Plastin-3	PLS3	Homo sapiens	70.813	20	18	9618344	8371607
978	1	P17301	P17301	Integrin alpha-2	ITGA2	Homo sapiens	129.295	18	18	6132484	2708030
979	1	P17655	P17655	Calpain-2 catalytic subunit	CAPN2	Homo sapiens	79.996	18	18	18033972	3859152
980	1	P17812	P17812	CTP synthase 1	CTPS1	Homo sapiens	66.691	20	18	6850127	4171884
981	1	P18031	P18031	Tyrosine-protein phosphatase non-receptor type 1	PTPN1	Homo sapiens	49.968	18	18	5038722	8230916
982	1	P20810	P20810	Calpastatin	CAST	Homo sapiens	76.573	18	18	28582449	8345697
983	1	P23743	P23743	Diacylglycerol kinase alpha	DGKA	Homo sapiens	82.632	18	18	2873162	4884442
984	1	P26038	P26038	Moesin	MSN	Homo sapiens	67.82	27	18	101463638	69958983
985	1	P29401	P29401	Transketolase	TKT	Homo sapiens	67.878	18	18	32004154	60297147
986	1	P30041	P30041	Peroxiredoxin-6	PRDX6	Homo sapiens	25.034	18	18	95630484	28913249
987	1	P31040	P31040	Succinate dehydrogenase [ubiquinone] flavoprotein subunit, mitochondrial	SDHA	Homo sapiens	72.69	18	18	27196263	5126595
988	1	P31327	P31327	Carbamoyl-phosphate synthase [ammonia], mitochondrial	CPS1	Homo sapiens	164.943	18	18	523376	2695885
989	1	P39060	P39060	Collagen alpha-1(XVIII) chain	COL18A1	Homo sapiens	178.191	18	18	31689803	15758893
990	1	P41226	P41226	Ubiquitin-like modifier-activating enzyme 7	UBA7	Homo sapiens	111.695	18	18	3451736	4463479

991	1	P46063	P46063	ATP-dependent DNA helicase Q1	RECQL	Homo sapiens	73.462	18	18	11423239	5847450
992	1	P51991	P51991	Heterogeneous nuclear ribonucleoprotein A3	HNRNPA3	Homo sapiens	39.595	18	18	31127994	11535695
993	1	P57737	P57737	Coronin-7	CORO7	Homo sapiens	100.607	18	18	6051978	15279540
994	1	P57740	P57740	Nuclear pore complex protein Nup107	NUP107	Homo sapiens	106.375	18	18	12465168	3349558
995	1	P61764	P61764	Syntaxin-binding protein 1	STXBP1	Homo sapiens	67.569	18	18	8438094	290874
996	1	P80303	P80303	Nucleobindin-2	NUCB2	Homo sapiens	50.222	18	18	7710421	3632626
997	1	Q07955	Q07955	Serine/arginine-rich splicing factor 1	SRSF1	Homo sapiens	27.743	18	18	18993211	6085686
998	1	Q12830	Q12830	Nucleosome-remodeling factor subunit BPTF	BPTF	Homo sapiens	338.27	18	18	1116637	223975
999	1	Q13263	Q13263	Transcription intermediary factor 1-beta	TRIM28	Homo sapiens	88.553	18	18	15043166	5291340
1000	1	Q13418	Q13418	Integrin-linked protein kinase	ILK	Homo sapiens	51.419	18	18	76598313	7495098
1001	1	Q13683	Q13683	Integrin alpha-7	ITGA7	Homo sapiens	128.948	18	18	14075167	150594
1002	1	Q13724	Q13724	Mannosyl-oligosaccharide glucosidase	MOGS	Homo sapiens	91.917	18	18	11181824	8103042
1003	1	Q14139	Q14139	Ubiquitin conjugation factor E4 A	UBE4A	Homo sapiens	122.563	18	18	4252990	1281355
1004	1	Q14157	Q14157	Ubiquitin-associated protein 2-like	UBAP2L	Homo sapiens	114.536	18	18	3389189	953067
1005	1	Q14566	Q14566	DNA replication licensing factor MCM6	MCM6	Homo sapiens	92.889	18	18	3519246	1059191
1006	1	Q14689	Q14689	Disco-interacting protein 2 homolog A	DIP2A	Homo sapiens	170.37	23	18	1893233	251517
1007	1	Q2M389	Q2M389	WASH complex subunit 4	WASHC4	Homo sapiens	136.404	18	18	3972972	2804326
1008	1	Q5SW79	Q5SW79	Centrosomal protein of 170 kDa	CEP170	Homo sapiens	175.295	25	18	1460521	1714444
1009	1	Q5T2T1	Q5T2T1	MAGUK p55 subfamily member 7	MPP7	Homo sapiens	65.522	18	18	3086549	848096
1010	1	Q5UIP0	Q5UIP0	Telomere-associated protein RIF1	RIF1	Homo sapiens	274.472	18	18	735273	639277
1011	1	Q5VTR2	Q5VTR2	E3 ubiquitin-protein ligase BRE1A	RNF20	Homo sapiens	113.663	18	18	2238275	948050
1012	1	Q5VW36	Q5VW36	Focadhesin	FOCAD	Homo sapiens	200.06	18	18	2012481	549055
1013	1	Q6IQ23	Q6IQ23	Pleckstrin homology domain-containing family A member 7	PLEKHA7	Homo sapiens	127.137	18	18	1676497	0
1014	1	Q6PKG0	Q6PKG0	La-related protein 1	LARP1	Homo sapiens	123.512	22	18	5512291	4517843
1015	1	Q6UWE0	Q6UWE0	E3 ubiquitin-protein ligase LRSAM1	LRSAM1	Homo sapiens	83.593	18	18	7286148	768091
1016	1	Q6YHK3	Q6YHK3	CD109 antigen	CD109	Homo sapiens	161.689	18	18	6646646	115878
1017	1	Q7Z3D6	Q7Z3D6	D-glutamate cyclase, mitochondrial	DGLUCY	Homo sapiens	66.436	18	18	18785499	1437924
1018	1	Q86UE4	Q86UE4	Protein LYRIC	MTDH	Homo sapiens	63.837	18	18	14392873	5660562
1019	1	Q86YS7	Q86YS7	C2 domain-containing protein 5	C2CD5	Homo sapiens	110.448	18	18	2924007	1016517
1020	1	Q8IYB7	Q8IYB7	DIS3-like exonuclease 2	DIS3L2	Homo sapiens	99.279	18	18	2167347	442908
1021	1	Q8N3F8	Q8N3F8	MICAL-like protein 1	MICALL1	Homo sapiens	93.442	18	18	5814636	399615
1022	1	Q8N8S7	Q8N8S7	Protein enabled homolog	ENAH	Homo sapiens	66.51	18	18	15437456	1489397
1023	1	Q8NI27	Q8NI27	THO complex subunit 2	THOC2	Homo sapiens	182.778	18	18	1726536	1144263
1024	1	Q92859	Q92859	Neogenin	NEO1	Homo sapiens	160.018	18	18	3182336	45252
1025	1	Q96AE4	Q96AE4	Far upstream element-binding protein 1	FUBP1	Homo sapiens	67.561	18	18	22121586	11396158
1026	1	Q96JG6	Q96JG6	Syndetin	VPS50	Homo sapiens	111.175	18	18	3031590	473923
1027	1	Q96KR1	Q96KR1	Zinc finger RNA-binding protein	ZFR	Homo sapiens	117.013	18	18	3617531	424023
1028	1	Q96PK6	Q96PK6	RNA-binding protein 14	RBM14	Homo sapiens	69.491	18	18	11821659	4375008
1029	1	Q96RT1	Q96RT1	Erbin	ERBIN	Homo sapiens	158.299	18	18	2194646	779625
1030	1	Q99570	Q99570	Phosphoinositide 3-kinase regulatory subunit 4	PIK3R4	Homo sapiens	153.106	18	18	2168197	1396064
1031	1	Q9BY44	Q9BY44	Eukaryotic translation initiation factor 2A	EIF2A	Homo sapiens	64.99	18	18	6757957	3184318
1032	1	Q9H1A4	Q9H1A4	Anaphase-promoting complex subunit 1	ANAPC1	Homo sapiens	216.5	18	18	1220200	570639
1033	1	Q9H4M9	Q9H4M9	EH domain-containing protein 1	EHD1	Homo sapiens	60.627	29	18	23399972	25246156
1034	1	Q9HCE1	Q9HCE1	Helicase MOV-10	MOV10	Homo sapiens	113.672	18	18	3173737	328528
1035	1	Q9NQW7	Q9NQW7	Xaa-Pro aminopeptidase 1	XPNPEP1	Homo sapiens	69.92	18	18	9746618	5623065
1036	1	Q9NSD9	Q9NSD9	Phenylalanine--tRNA ligase beta subunit	FARSB	Homo sapiens	66.117	18	18	15660052	6768079
1037	1	Q9NUB1	Q9NUB1	Acetyl-coenzyme A synthetase 2-like, mitochondrial	ACSS1	Homo sapiens	74.857	18	18	6392735	1216825
1038	1	Q9NWH9	Q9NWH9	SAFB-like transcription modulator	SLTM	Homo sapiens	117.151	18	18	3693556	3151089

1039	1	Q9NYF8	Q9NYF8	Bcl-2-associated transcription factor 1	BCLAF1	Homo sapiens	106.124	18	18	5375398	1726771
1040	1	Q9UHB9	Q9UHB9	Signal recognition particle subunit SRP68	SRP68	Homo sapiens	70.73	18	18	9050679	5113971
1041	1	Q9UHG3	Q9UHG3	Prenylcysteine oxidase 1	PCYOX1	Homo sapiens	56.64	18	18	19084695	3424396
1042	1	Q9UHN6	Q9UHN6	Cell surface hyaluronidase	CEMIP2	Homo sapiens	154.376	18	18	1899198	3003117
1043	1	Q9UKG1	Q9UKG1	DCC-interacting protein 13-alpha	APPL1	Homo sapiens	79.664	18	18	11229843	6222547
1044	1	Q9Y2H6	Q9Y2H6	Fibronectin type-III domain-containing protein 3A	FNDC3A	Homo sapiens	131.854	18	18	2476526	989551
1045	1	Q9Y2J8	Q9Y2J8	Protein-arginine deiminase type-2	PADI2	Homo sapiens	75.563	18	18	21463215	15682499
1046	1	Q9Y3S1	Q9Y3S1	Serine/threonine-protein kinase WNK2	WNK2	Homo sapiens	242.68	19	18	2439989	0
1047	1	Q9Y4E6	Q9Y4E6	WD repeat-containing protein 7	WDR7	Homo sapiens	163.812	18	18	3304191	1720921
1048	1	Q9Y4K1	Q9Y4K1	Beta/gamma crystallin domain-containing protein 1	CRYBG1	Homo sapiens	188.679	18	18	2269621	2094276
1049	1	O00291	O00291	Huntingtin-interacting protein 1	HIP1	Homo sapiens	116.223	18	17	1748500	1925385
1050	1	O00469	O00469	Procollagen-lysine,2-oxoglutarate 5-dioxygenase 2	PLOD2	Homo sapiens	84.686	17	17	1607634	5891715
1051	1	O00533	O00533	Neural cell adhesion molecule L1-like protein	CHL1	Homo sapiens	135.071	17	17	5563363	30551
1052	1	O14745	O14745	Na(+)/H(+) exchange regulatory cofactor NHE-RF1	NHERF1	Homo sapiens	38.867	17	17	14043466	9165457
1053	1	O14920	O14920	Inhibitor of nuclear factor kappa-B kinase subunit beta	IKBKB	Homo sapiens	86.565	17	17	1899952	2398521
1054	1	O15056	O15056	Synaptojanin-2	SYNJ2	Homo sapiens	165.541	17	17	2094920	57175
1055	1	O15484	O15484	Calpain-5	CAPN5	Homo sapiens	73.171	17	17	8507709	0
1056	1	O43150	O43150	Arf-GAP with SH3 domain, ANK repeat and PH domain-containing protein 2	ASAP2	Homo sapiens	111.65	17	17	2385248	325800
1057	1	O43237	O43237	Cytoplasmic dynein 1 light intermediate chain 2	DYNC1LI2	Homo sapiens	54.101	17	17	20166626	3473101
1058	1	O43301	O43301	Heat shock 70 kDa protein 12A	HSPA12A	Homo sapiens	74.978	18	17	7010628	99798
1059	1	O43592	O43592	Exportin-T	XPOT	Homo sapiens	109.967	17	17	3467134	3173281
1060	1	O43719	O43719	HIV Tat-specific factor 1	HTATSF1	Homo sapiens	85.854	17	17	2688877	830843
1061	1	O60341	O60341	Lysine-specific histone demethylase 1A	KDM1A	Homo sapiens	92.903	17	17	2155654	691110
1062	1	O60343	O60343	TBC1 domain family member 4	TBC1D4	Homo sapiens	146.567	17	17	2295176	111463
1063	1	O60934	O60934	Nibrin	NBN	Homo sapiens	84.962	17	17	3174074	929711
1064	1	O75410	O75410	Transforming acidic coiled-coil-containing protein 1	TACC1	Homo sapiens	87.795	17	17	5103066	691954
1065	1	O75439	O75439	Mitochondrial-processing peptidase subunit beta	PMPCB	Homo sapiens	54.367	17	17	6541555	3087891
1066	1	O75746	O75746	Electrogenic aspartate/glutamate antiporter SLC25A12, mitochondrial	SLC25A12	Homo sapiens	74.762	17	17	13880229	1688428
1067	1	O75874	O75874	Isocitrate dehydrogenase [NADP] cytoplasmic	IDH1	Homo sapiens	46.659	17	17	61919838	29897068
1068	1	O94776	O94776	Metastasis-associated protein MTA2	MTA2	Homo sapiens	75.024	19	17	6415782	3143946
1069	1	O94901	O94901	SUN domain-containing protein 1	SUN1	Homo sapiens	87.111	17	17	4332790	426003
1070	1	O95352	O95352	Ubiquitin-like modifier-activating enzyme ATG7	ATG7	Homo sapiens	77.96	17	17	4165767	7778738
1071	1	P00390	P00390	Glutathione reductase, mitochondrial	GSR	Homo sapiens	56.257	17	17	40843959	17872765
1072	1	P01042	P01042	Kininogen-1	KNG1	Homo sapiens	71.959	17	17	41295593	87724158
1073	1	P04083	P04083	Annexin A1	ANXA1	Homo sapiens	38.714	17	17	144676355	322716200
1074	1	P07099	P07099	Epoxide hydrolase 1	EPHX1	Homo sapiens	52.949	17	17	28722891	11285881
1075	1	P07686	P07686	Beta-hexosaminidase subunit beta	HEXB	Homo sapiens	63.138	17	17	19142796	8605167
1076	1	P07900	P07900	Heat shock protein HSP 90-alpha	HSP90AA1	Homo sapiens	84.662	35	17	69459324	49395557
1077	1	P07948	P07948	Tyrosine-protein kinase Lyn	LYN	Homo sapiens	58.575	19	17	2383231	15918622
1078	1	P08572	P08572	Collagen alpha-2(IV) chain	COL4A2	Homo sapiens	167.555	17	17	86159256	24296371
1079	1	P09769	P09769	Tyrosine-protein kinase Fgr	FGR	Homo sapiens	59.478	17	17	265592	14716903
1080	1	P12277	P12277	Creatine kinase B-type	CKB	Homo sapiens	42.646	17	17	716173725	5275523
1081	1	P12694	P12694	2-oxoisovalerate dehydrogenase subunit alpha, mitochondrial	BCKDHA	Homo sapiens	50.472	17	17	4772706	702472
1082	1	P13489	P13489	Ribonuclease inhibitor	RNH1	Homo sapiens	49.976	17	17	40194000	22233960
1083	1	P13674	P13674	Prolyl 4-hydroxylase subunit alpha-1	P4HA1	Homo sapiens	61.052	17	17	4006075	9070861
1084	1	P14410	P14410	Sucrase-isomaltase, intestinal	SI	Homo sapiens	209.457	17	17	1322712	0
1085	1	P15498	P15498	Proto-oncogene vav	VAV1	Homo sapiens	98.315	18	17	1518336	8072673

1086	1	P16452	P16452	Protein 4.2	EPB42	Homo sapiens	77.008	17	17	3535808	7914041
1087	1	P19971	P19971	Thymidine phosphorylase	TYMP	Homo sapiens	49.954	17	17	12377381	35952627
1088	1	P21397	P21397	Amine oxidase [flavin-containing] A	MAOA	Homo sapiens	59.681	18	17	64780097	3748663
1089	1	P22695	P22695	Cytochrome b-c1 complex subunit 2, mitochondrial	UQCRC2	Homo sapiens	48.445	17	17	47413137	8112188
1090	1	P23368	P23368	NAD-dependent malic enzyme, mitochondrial	ME2	Homo sapiens	65.442	17	17	13402511	5952714
1091	1	P27987	P27987	Inositol-trisphosphate 3-kinase B	ITPKB	Homo sapiens	102.377	18	17	4704675	82843
1092	1	P31150	P31150	Rab GDP dissociation inhibitor alpha	GDI1	Homo sapiens	50.585	17	17	24283502	5355686
1093	1	P36578	P36578	60S ribosomal protein L4	RPL4	Homo sapiens	47.696	17	17	72200847	27152007
1094	1	P40818	P40818	Ubiquitin carboxyl-terminal hydrolase 8	USP8	Homo sapiens	127.523	17	17	2839224	1553389
1095	1	P48553	P48553	Trafficking protein particle complex subunit 10	TRAPPC10	Homo sapiens	142.191	17	17	1131274	1072962
1096	1	P49321	P49321	Nuclear autoantigenic sperm protein	NASP	Homo sapiens	85.237	17	17	9936916	4663928
1097	1	P49815	P49815	Tuberin	TSC2	Homo sapiens	200.613	17	17	1433957	533339
1098	1	P51812	P51812	Ribosomal protein S6 kinase alpha-3	RPS6KA3	Homo sapiens	83.737	20	17	7240906	4483250
1099	1	P51911	P51911	Calponin-1	CNN1	Homo sapiens	33.17	17	17	1327817166	2594514
1100	1	P52888	P52888	Thimet oligopeptidase	THOP1	Homo sapiens	78.842	17	17	2617798	2097813
1101	1	P54652	P54652	Heat shock-related 70 kDa protein 2	HSPA2	Homo sapiens	70.02	18	17	70608698	2449191
1102	1	P55010	P55010	Eukaryotic translation initiation factor 5	EIF5	Homo sapiens	49.224	17	17	10522859	3847942
1103	1	P55160	P55160	Nck-associated protein 1-like	NCKAP1L	Homo sapiens	128.156	17	17	3045615	8240043
1104	1	Q00013	Q00013	55 kDa erythrocyte membrane protein	MPP1	Homo sapiens	52.295	17	17	4637313	7311975
1105	1	Q01433	Q01433	AMP deaminase 2	AMPD2	Homo sapiens	100.69	17	17	4589034	3379481
1106	1	Q15027	Q15027	Arf-GAP with coiled-coil, ANK repeat and PH domain-containing protein 1	ACAP1	Homo sapiens	81.537	17	17	139122	5637472
1107	1	Q15080	Q15080	Neutrophil cytosol factor 4	NCF4	Homo sapiens	39.031	17	17	1778098	43514653
1108	1	Q15154	Q15154	Pericentriolar material 1 protein	PCM1	Homo sapiens	228.546	17	17	1753815	102765
1109	1	Q15437	Q15437	Protein transport protein Sec23B	SEC23B	Homo sapiens	86.481	17	17	5009314	2234160
1110	1	Q16181	Q16181	Septin-7	SEPTIN7	Homo sapiens	50.682	17	17	32119497	5179821
1111	1	Q16204	Q16204	Coiled-coil domain-containing protein 6	CCDC6	Homo sapiens	53.291	17	17	11279258	2847560
1112	1	Q16795	Q16795	NADH dehydrogenase [ubiquinone] 1 alpha subcomplex subunit 9, mitochondrial	NDUFA9	Homo sapiens	42.509	17	17	24628417	3937682
1113	1	Q53SF7	Q53SF7	Cordon-bleu protein-like 1	COBLL1	Homo sapiens	123.869	17	17	1774302	61164
1114	1	Q6NUK1	Q6NUK1	Calcium-binding mitochondrial carrier protein SCaMC-1	SLC25A24	Homo sapiens	53.355	17	17	15841518	5490250
1115	1	Q6P3W7	Q6P3W7	SCY1-like protein 2	SCYL2	Homo sapiens	103.711	17	17	4445512	2629029
1116	1	Q6P996	Q6P996	Pyridoxal-dependent decarboxylase domain-containing protein 1	PDXDC1	Homo sapiens	86.707	24	17	15414913	2027640
1117	1	Q6PJT7	Q6PJT7	Zinc finger CCCH domain-containing protein 14	ZC3H14	Homo sapiens	82.878	17	17	2685650	489660
1118	1	Q6VY07	Q6VY07	Phosphofurin acidic cluster sorting protein 1	PACS1	Homo sapiens	104.9	17	17	4280037	3867363
1119	1	Q7Z2Z2	Q7Z2Z2	Elongation factor-like GTPase 1	EFL1	Homo sapiens	125.43	17	17	2410691	1598774
1120	1	Q7Z3U7	Q7Z3U7	Protein MON2 homolog	MON2	Homo sapiens	190.362	17	17	3001899	810479
1121	1	Q7Z417	Q7Z417	FMR1-interacting protein NUFIP2	NUFIP2	Homo sapiens	76.122	17	17	1838179	1599660
1122	1	Q86VS8	Q86VS8	Protein Hook homolog 3	HOOK3	Homo sapiens	83.129	17	17	3807760	2697830
1123	1	Q8IYB8	Q8IYB8	ATP-dependent RNA helicase SUPV3L1, mitochondrial	SUPV3L1	Homo sapiens	87.992	17	17	3433270	479342
1124	1	Q8N201	Q8N201	Integrator complex subunit 1	INTS1	Homo sapiens	244.302	17	17	1162398	758168
1125	1	Q8NBJ4	Q8NBJ4	Golgi membrane protein 1	GOLM1	Homo sapiens	45.332	17	17	6633969	1707261
1126	1	Q8NEB9	Q8NEB9	Phosphatidylinositol 3-kinase catalytic subunit type 3	PIK3C3	Homo sapiens	101.552	17	17	2708176	1501750
1127	1	Q8TAT6	Q8TAT6	Nuclear protein localization protein 4 homolog	NPLOC4	Homo sapiens	68.121	17	17	3497612	4007288
1128	1	Q8TCS8	Q8TCS8	Polyribonucleotide nucleotidyltransferase 1, mitochondrial	PNPT1	Homo sapiens	85.956	17	17	5669073	1784065
1129	1	Q8WVM8	Q8WVM8	Sec1 family domain-containing protein 1	SCFD1	Homo sapiens	72.381	17	17	6489019	2333681
1130	1	Q8WVV9	Q8WVV9	Heterogeneous nuclear ribonucleoprotein L-like	HNRNPLL	Homo sapiens	60.083	17	17	2775458	2889397
1131	1	Q92667	Q92667	A-kinase anchor protein 1, mitochondrial	AKAP1	Homo sapiens	97.342	17	17	29428114	303190
1132	1	Q92973	Q92973	Transportin-1	TNPO1	Homo sapiens	102.357	19	17	10770451	5503075

1133	1	Q92974	Q92974	Rho guanine nucleotide exchange factor 2	ARHGEF2	Homo sapiens	111.544	17	17	1890845	6100970
1134	1	Q93100	Q93100	Phosphorylase b kinase regulatory subunit beta	PHKB	Homo sapiens	124.885	17	17	1768073	3371142
1135	1	Q96HE7	Q96HE7	ERO1-like protein alpha	ERO1A	Homo sapiens	54.392	18	17	180417028	22214049
1136	1	Q96JB2	Q96JB2	Conserved oligomeric Golgi complex subunit 3	COG3	Homo sapiens	94.098	17	17	3545521	799312
1137	1	Q96JB5	Q96JB5	CDK5 regulatory subunit-associated protein 3	CDK5RAP3	Homo sapiens	56.919	17	17	5056635	1462489
1138	1	Q96JQ0	Q96JQ0	Protocadherin-16	DCHS1	Homo sapiens	346.184	17	17	1978339	0
1139	1	Q96L92	Q96L92	Sorting nexin-27	SNX27	Homo sapiens	61.264	17	17	8256729	8609884
1140	1	Q96T37	Q96T37	RNA-binding protein 15	RBM15	Homo sapiens	107.191	17	17	3964283	1578047
1141	1	Q99569	Q99569	Plakophilin-4	PKP4	Homo sapiens	131.868	18	17	2030710	58702
1142	1	Q9BT78	Q9BT78	COP9 signalosome complex subunit 4	COPS4	Homo sapiens	46.271	17	17	12471695	3774536
1143	1	Q9BTW9	Q9BTW9	Tubulin-specific chaperone D	TBCD	Homo sapiens	132.601	17	17	2818595	347972
1144	1	Q9C0J8	Q9C0J8	pre-mRNA 3' end processing protein WDR33	WDR33	Homo sapiens	145.891	17	17	2256942	756388
1145	1	Q9GZM7	Q9GZM7	Tubulointerstitial nephritis antigen-like	TINAGL1	Homo sapiens	52.387	17	17	12379157	2621035
1146	1	Q9H0A0	Q9H0A0	RNA cytidine acetyltransferase	NAT10	Homo sapiens	115.732	17	17	6571105	1202831
1147	1	Q9H0D6	Q9H0D6	5'-3' exoribonuclease 2	XRN2	Homo sapiens	108.582	17	17	4182106	3130676
1148	1	Q9H267	Q9H267	Vacuolar protein sorting-associated protein 33B	VPS33B	Homo sapiens	70.588	17	17	2605824	1114112
1149	1	Q9H845	Q9H845	Complex I assembly factor ACAD9, mitochondrial	ACAD9	Homo sapiens	68.761	17	17	6144587	3845663
1150	1	Q9HAU5	Q9HAU5	Regulator of nonsense transcripts 2	UPF2	Homo sapiens	147.81	17	17	3232688	1120352
1151	1	Q9HCB6	Q9HCB6	Spondin-1	SPON1	Homo sapiens	90.977	17	17	12486530	1470221
1152	1	Q9HDC9	Q9HDC9	Adipocyte plasma membrane-associated protein	APMAP	Homo sapiens	46.48	17	17	14648563	74911116
1153	1	Q9NRF8	Q9NRF8	CTP synthase 2	CTPS2	Homo sapiens	65.679	17	17	4478650	2217225
1154	1	Q9NRY5	Q9NRY5	Protein FAM114A2	FAM114A2	Homo sapiens	55.467	17	17	3181910	2208025
1155	1	Q9NU22	Q9NU22	Midasin	MDN1	Homo sapiens	632.83	17	17	848370	59812
1156	1	Q9NVP1	Q9NVP1	ATP-dependent RNA helicase DDX18	DDX18	Homo sapiens	75.408	17	17	4030119	962401
1157	1	Q9P253	Q9P253	Vacuolar protein sorting-associated protein 18 homolog	VPS18	Homo sapiens	110.186	17	17	2386549	2345804
1158	1	Q9UBT2	Q9UBT2	SUMO-activating enzyme subunit 2	UBA2	Homo sapiens	71.226	17	17	9188212	5288538
1159	1	Q9UG63	Q9UG63	ATP-binding cassette sub-family F member 2	ABCF2	Homo sapiens	71.292	17	17	4199953	1630350
1160	1	Q9UGI8	Q9UGI8	Testin	TES	Homo sapiens	47.998	17	17	98134493	8146040
1161	1	Q9UJU6	Q9UJU6	Drebrin-like protein	DBNL	Homo sapiens	48.207	17	17	14422526	12925887
1162	1	Q9UMZ2	Q9UMZ2	Synergin gamma	SYNRG	Homo sapiens	140.657	17	17	1707753	475659
1163	1	Q9UNZ2	Q9UNZ2	NSFL1 cofactor p47	NSFL1C	Homo sapiens	40.571	17	17	13832223	4955597
1164	1	Q9UPW5	Q9UPW5	Cytosolic carboxypeptidase 1	AGTPBP1	Homo sapiens	138.45	18	17	177956	2544069
1165	1	Q9Y265	Q9Y265	RuvB-like 1	RUVBL1	Homo sapiens	50.226	17	17	17286435	11288607
1166	1	Q9Y2X3	Q9Y2X3	Nucleolar protein 58	NOP58	Homo sapiens	59.581	17	17	12552686	3628305
1167	1	Q9Y613	Q9Y613	FH1/FH2 domain-containing protein 1	FHOD1	Homo sapiens	126.554	17	17	1991037	1129776
1168	1	Q9Y6W3	Q9Y6W3	Calpain-7	CAPN7	Homo sapiens	92.654	17	17	2293035	1647669
1169	1	O00186	O00186	Syntaxin-binding protein 3	STXBP3	Homo sapiens	67.766	16	16	8952182	3297100
1170	1	O00391	O00391	Sulfhydryl oxidase 1	QSOX1	Homo sapiens	82.576	16	16	9710703	10043116
1171	1	O00443	O00443	Phosphatidylinositol 4-phosphate 3-kinase C2 domain-containing subunit alpha	PIK3C2A	Homo sapiens	190.684	16	16	1795017	420964
1172	1	O00515	O00515	Ladinin-1	LAD1	Homo sapiens	57.132	16	16	6926621	631304
1173	1	O14981	O14981	TATA-binding protein-associated factor 172	BTAF1	Homo sapiens	206.89	16	16	1899495	325369
1174	1	O43172	O43172	U4/U6 small nuclear ribonucleoprotein Prp4	PRPF4	Homo sapiens	58.45	16	16	3574961	1814837
1175	1	O43252	O43252	Bifunctional 3'-phosphoadenosine 5'-phosphosulfate synthase 1	PAPSS1	Homo sapiens	70.832	16	16	3309589	3416285
1176	1	O43294	O43294	Transforming growth factor beta-1-induced transcript 1 protein	TGFB1I1	Homo sapiens	49.814	16	16	30022802	696961
1177	1	O43314	O43314	Inositol hexakisphosphate and diphosphoinositol-pentakisphosphate kinase 2	PPIP5K2	Homo sapiens	140.408	21	16	1366562	2366559
1178	1	O60486	O60486	Plexin-C1	PLXNC1	Homo sapiens	175.744	16	16	134272	2032054

1179	1	O60832	O60832	H/ACA ribonucleoprotein complex subunit DKC1	DKC1	Homo sapiens	57.673	16	16	4762743	1639074
1180	1	O75112	O75112	LIM domain-binding protein 3	LDB3	Homo sapiens	77.133	16	16	17380558	0
1181	1	O75351	O75351	Vacuolar protein sorting-associated protein 4B	VPS4B	Homo sapiens	49.303	18	16	11766789	19906252
1182	1	O75489	O75489	NADH dehydrogenase [ubiquinone] iron-sulfur protein 3, mitochondrial	NDUFS3	Homo sapiens	30.239	16	16	19786247	3451848
1183	1	O75882	O75882	Attractin	ATRN	Homo sapiens	158.542	16	16	1438056	1821535
1184	1	O94819	O94819	Kelch repeat and BTB domain-containing protein 11	KBTBD11	Homo sapiens	65.72	16	16	2596534	1534980
1185	1	O94887	O94887	FERM, ARHGEF and pleckstrin domain-containing protein 2	FARP2	Homo sapiens	119.889	16	16	1376825	57205
1186	1	O94911	O94911	ABC-type organic anion transporter ABCA8	ABCA8	Homo sapiens	183.679	18	16	4936543	0
1187	1	O94966	O94966	Ubiquitin carboxyl-terminal hydrolase 19	USP19	Homo sapiens	145.65	16	16	1340072	600618
1188	1	O95140	O95140	Mitofusin-2	MFN2	Homo sapiens	86.403	16	16	3164828	1156063
1189	1	O95294	O95294	RasGAP-activating-like protein 1	RASAL1	Homo sapiens	90.016	16	16	11137	1737520
1190	1	O95373	O95373	Importin-7	IPO7	Homo sapiens	119.517	17	16	12044435	4031562
1191	1	O95831	O95831	Apoptosis-inducing factor 1, mitochondrial	AIFM1	Homo sapiens	66.9	16	16	29192953	7592390
1192	1	P00491	P00491	Purine nucleoside phosphorylase	PNP	Homo sapiens	32.118	16	16	17269990	21604444
1193	1	P01008	P01008	Antithrombin-III	SERPINC1	Homo sapiens	52.604	17	16	40113491	202698811
1194	1	P01011	P01011	Alpha-1-antichymotrypsin	SERPINA3	Homo sapiens	47.65	16	16	187546998	678973023
1195	1	P03952	P03952	Plasma kallikrein	KLKB1	Homo sapiens	71.368	17	16	7102622	17835414
1196	1	P05067	P05067	Amyloid-beta precursor protein	APP	Homo sapiens	86.944	16	16	1683198	734032
1197	1	P05455	P05455	Lupus La protein	SSB	Homo sapiens	46.835	16	16	27005183	12827001
1198	1	P07332	P07332	Tyrosine-protein kinase Fes/Fps	FES	Homo sapiens	93.498	17	16	392324	5764798
1199	1	P09543	P09543	2',3'-cyclic-nucleotide 3'-phosphodiesterase	CNP	Homo sapiens	47.58	16	16	21400219	9260757
1200	1	P09972	P09972	Fructose-bisphosphate aldolase C	ALDOC	Homo sapiens	39.458	16	16	16937598	10676335
1201	1	P10636	P10636	Microtubule-associated protein tau	MAPT	Homo sapiens	78.928	16	16	6422333	0
1202	1	P11274	P11274	Breakpoint cluster region protein	BCR	Homo sapiens	142.823	16	16	1536601	1152256
1203	1	P16278	P16278	Beta-galactosidase	GLB1	Homo sapiens	76.074	17	16	9641030	5163663
1204	1	P17174	P17174	Aspartate aminotransferase, cytoplasmic	GOT1	Homo sapiens	46.247	16	16	29910929	6903068
1205	1	P19801	P19801	Amiloride-sensitive amine oxidase [copper-containing]	AOC1	Homo sapiens	85.379	16	16	7264734	0
1206	1	P20073	P20073	Annexin A7	ANXA7	Homo sapiens	52.739	16	16	25523012	13840978
1207	1	P20592	P20592	Interferon-induced GTP-binding protein Mx2	MX2	Homo sapiens	82.088	18	16	1355339	4889712
1208	1	P20839	P20839	Inosine-5'-monophosphate dehydrogenase 1	IMPDH1	Homo sapiens	55.406	16	16	1110131	5230197
1209	1	P23381	P23381	Tryptophan--tRNA ligase, cytoplasmic	WARS1	Homo sapiens	53.168	16	16	26620478	96212382
1210	1	P27338	P27338	Amine oxidase [flavin-containing] B	MAOB	Homo sapiens	58.763	16	16	19489892	558726
1211	1	P28340	P28340	DNA polymerase delta catalytic subunit	POLD1	Homo sapiens	123.63	16	16	1600727	614071
1212	1	P35052	P35052	Glypican-1	GPC1	Homo sapiens	61.681	16	16	4579282	246740
1213	1	P35232	P35232	Prohibitin 1	PHB1	Homo sapiens	29.801	16	16	60462131	21738406
1214	1	P35558	P35558	Phosphoenolpyruvate carboxykinase, cytosolic [GTP]	PCK1	Homo sapiens	69.195	16	16	6450221	37362
1215	1	P40926	P40926	Malate dehydrogenase, mitochondrial	MDH2	Homo sapiens	35.502	16	16	154986077	33527747
1216	1	P41218	P41218	Myeloid cell nuclear differentiation antigen	MNDA	Homo sapiens	45.837	16	16	5197430	148090808
1217	1	P43034	P43034	Platelet-activating factor acetylhydrolase IB subunit beta	PAFAH1B1	Homo sapiens	46.639	16	16	17982921	7906140
1218	1	P43686	P43686	26S proteasome regulatory subunit 6B	PSMC4	Homo sapiens	47.367	16	16	5999373	4094784
1219	1	P46087	P46087	Probable 28S rRNA (cytosine(4447)-C(5))-methyltransferase	NOP2	Homo sapiens	89.303	16	16	5196961	5505325
1220	1	P46977	P46977	Dolichyl-diphosphooligosaccharide--protein glycosyltransferase subunit STT3A	STT3A	Homo sapiens	80.53	16	16	19317857	14613099
1221	1	P47712	P47712	Cytosolic phospholipase A2	PLA2G4A	Homo sapiens	85.241	16	16	2891367	1924176
1222	1	P50453	P50453	Serpin B9	SERPINB9	Homo sapiens	42.403	17	16	8188280	14418218
1223	1	P50479	P50479	PDZ and LIM domain protein 4	PDLIM4	Homo sapiens	35.396	16	16	25676687	1123741
1224	1	P51608	P51608	Methyl-CpG-binding protein 2	MECP2	Homo sapiens	52.441	16	16	14618377	1427337
1225	1	P51888	P51888	Prolargin	PRELP	Homo sapiens	43.812	16	16	223233090	1415508

1226	1	P52630	P52630	Signal transducer and activator of transcription 2	STAT2	Homo sapiens	97.918	16	16	2173276	1404760
1227	1	P61158	P61158	Actin-related protein 3	ACTR3	Homo sapiens	47.371	18	16	42456337	50594906
1228	1	P62333	P62333	26S proteasome regulatory subunit 10B	PSMC6	Homo sapiens	44.172	16	16	9013596	7545758
1229	1	P63244	P63244	Receptor of activated protein C kinase 1	RACK1	Homo sapiens	35.075	16	16	58320879	25057049
1230	1	Q00653	Q00653	Nuclear factor NF-kappa-B p100 subunit	NFKB2	Homo sapiens	96.749	16	16	1663141	2160519
1231	1	Q00839	Q00839	Heterogeneous nuclear ribonucleoprotein U	HNRNPU	Homo sapiens	90.588	16	16	73111491	40959414
1232	1	Q03164	Q03164	Histone-lysine N-methyltransferase 2A	KMT2A	Homo sapiens	431.775	16	16	1872795	0
1233	1	Q04446	Q04446	1,4-alpha-glucan-branching enzyme	GBE1	Homo sapiens	80.474	16	16	6736709	14734882
1234	1	Q10567	Q10567	AP-1 complex subunit beta-1	AP1B1	Homo sapiens	104.607	33	16	15543383	10354015
1235	1	Q12907	Q12907	Vesicular integral-membrane protein VIP36	LMAN2	Homo sapiens	40.23	16	16	21217989	23681523
1236	1	Q13308	Q13308	Inactive tyrosine-protein kinase 7	PTK7	Homo sapiens	118.391	16	16	3295847	1848656
1237	1	Q13488	Q13488	V-type proton ATPase 116 kDa subunit a 3	TCIRG1	Homo sapiens	92.969	16	16	903745	9036162
1238	1	Q14156	Q14156	Protein EFR3 homolog A	EFR3A	Homo sapiens	92.925	17	16	2103470	883458
1239	1	Q14515	Q14515	SPARC-like protein 1	SPARCL1	Homo sapiens	75.208	16	16	2834088	690001
1240	1	Q14554	Q14554	Protein disulfide-isomerase A5	PDIA5	Homo sapiens	59.595	16	16	8329407	4218877
1241	1	Q14651	Q14651	Plastin-1	PLS1	Homo sapiens	70.254	16	16	30392821	2133373
1242	1	Q14677	Q14677	Clathrin interactor 1	CLINT1	Homo sapiens	68.26	16	16	12454506	6176935
1243	1	Q14746	Q14746	Conserved oligomeric Golgi complex subunit 2	COG2	Homo sapiens	83.209	16	16	2003968	554334
1244	1	Q15008	Q15008	26S proteasome non-ATPase regulatory subunit 6	PSMD6	Homo sapiens	45.533	16	16	9514725	8663224
1245	1	Q15052	Q15052	Rho guanine nucleotide exchange factor 6	ARHGEF6	Homo sapiens	87.5	20	16	2348222	5302848
1246	1	Q15057	Q15057	Arf-GAP with coiled-coil, ANK repeat and PH domain-containing protein 2	ACAP2	Homo sapiens	88.03	17	16	2477780	7217127
1247	1	Q15334	Q15334	Lethal(2) giant larvae protein homolog 1	LLGL1	Homo sapiens	115.419	16	16	2813324	63629
1248	1	Q16643	Q16643	Drebrin	DBN1	Homo sapiens	71.43	16	16	10562222	3378640
1249	1	Q32P28	Q32P28	Prolyl 3-hydroxylase 1	P3H1	Homo sapiens	83.395	16	16	1965175	2773477
1250	1	Q5HYK7	Q5HYK7	SH3 domain-containing protein 19	SH3D19	Homo sapiens	86.527	16	16	3578318	0
1251	1	Q5T5C0	Q5T5C0	Syntaxin-binding protein 5	STXBP5	Homo sapiens	127.575	17	16	1766996	4064762
1252	1	Q69YN4	Q69YN4	Protein virilizer homolog	VIRMA	Homo sapiens	202.027	16	16	3192901	220894
1253	1	Q6SZW1	Q6SZW1	NAD(+) hydrolase SARM1	SARM1	Homo sapiens	79.387	16	16	1409791	15294
1254	1	Q7L7X3	Q7L7X3	Serine/threonine-protein kinase TAO1	TAOK1	Homo sapiens	116.072	17	16	1357333	483891
1255	1	Q7Z3B4	Q7Z3B4	Nucleoporin p54	NUP54	Homo sapiens	55.434	16	16	2186194	1809049
1256	1	Q7Z3T8	Q7Z3T8	Zinc finger FYVE domain-containing protein 16	ZFYVE16	Homo sapiens	168.905	16	16	1901704	1947199
1257	1	Q7Z6E9	Q7Z6E9	E3 ubiquitin-protein ligase RBBP6	RBBP6	Homo sapiens	201.569	16	16	995309	232859
1258	1	Q7Z7G0	Q7Z7G0	Target of Nesh-SH3	ABI3BP	Homo sapiens	117.898	16	16	20851865	291797
1259	1	Q86UV5	Q86UV5	Ubiquitin carboxyl-terminal hydrolase 48	USP48	Homo sapiens	119.033	16	16	1035711	336394
1260	1	Q86X10	Q86X10	Ral GTPase-activating protein subunit beta	RALGAPB	Homo sapiens	166.804	16	16	2074993	758551
1261	1	Q8IUR7	Q8IUR7	Armadillo repeat-containing protein 8	ARMC8	Homo sapiens	75.511	16	16	2728246	940269
1262	1	Q8IVG5	Q8IVG5	Sterile alpha motif domain-containing protein 9-like	SAMD9L	Homo sapiens	184.535	16	16	278509	1287039
1263	1	Q8IZ83	Q8IZ83	Aldehyde dehydrogenase family 16 member A1	ALDH16A1	Homo sapiens	85.127	16	16	6117687	3888945
1264	1	Q8N1B4	Q8N1B4	Vacuolar protein sorting-associated protein 52 homolog	VPS52	Homo sapiens	82.223	16	16	2964530	1340466
1265	1	Q8NC51	Q8NC51	Plasminogen activator inhibitor 1 RNA-binding protein	SERBP1	Homo sapiens	44.967	16	16	20268662	6352526
1266	1	Q8ND30	Q8ND30	Liprin-beta-2	PPFIBP2	Homo sapiens	98.547	16	16	1992615	201707
1267	1	Q8TB22	Q8TB22	Spermatogenesis-associated protein 20	SPATA20	Homo sapiens	87.9	16	16	3020880	148536
1268	1	Q8TEU7	Q8TEU7	Rap guanine nucleotide exchange factor 6	RAPGEF6	Homo sapiens	179.426	19	16	263818	2044799
1269	1	Q8WWM7	Q8WWM7	Ataxin-2-like protein	ATXN2L	Homo sapiens	113.376	16	16	5463799	1756473
1270	1	Q8WXE0	Q8WXE0	Caskin-2	CASKIN2	Homo sapiens	126.782	17	16	2064389	0
1271	1	Q92508	Q92508	Piezo-type mechanosensitive ion channel component 1	PIEZO1	Homo sapiens	286.794	16	16	2062994	1034090
1272	1	Q93050	Q93050	V-type proton ATPase 116 kDa subunit a 1	ATP6V0A1	Homo sapiens	96.415	17	16	6345318	7537521
1273	1	Q96AY3	Q96AY3	Peptidyl-prolyl cis-trans isomerase FKBP10	FKBP10	Homo sapiens	64.245	16	16	5447413	11951194

1274	1 Q96CM8	Q96CM8	Medium-chain acyl-CoA ligase ACSF2, mitochondrial	ACSF2	Homo sapiens	68.126	16	16	5930057	1564978
1275	1 Q96CW5	Q96CW5	Gamma-tubulin complex component 3	TUBGCP3	Homo sapiens	103.571	16	16	2213512	584475
1276	1 Q96FV9	Q96FV9	THO complex subunit 1	THOC1	Homo sapiens	75.669	16	16	3129119	865390
1277	1 Q96GQ7	Q96GQ7	Probable ATP-dependent RNA helicase DDX27	DDX27	Homo sapiens	89.834	16	16	2944844	504390
1278	1 Q96RQ3	Q96RQ3	Methylcrotonoyl-CoA carboxylase subunit alpha, mitochondrial	MCCC1	Homo sapiens	80.472	16	16	10642505	2108129
1279	1 Q9BS26	Q9BS26	Endoplasmic reticulum resident protein 44	ERP44	Homo sapiens	46.974	16	16	16564513	22396500
1280	1 Q9BTU6	Q9BTU6	Phosphatidylinositol 4-kinase type 2-alpha	PI4K2A	Homo sapiens	54.021	16	16	1379581	1941273
1281	1 Q9BZE4	Q9BZE4	GTP-binding protein 4	GTPBP4	Homo sapiens	73.965	16	16	2725893	1135725
1282	1 Q9BZJ0	Q9BZJ0	Crooked neck-like protein 1	CRNKL1	Homo sapiens	100.455	16	16	1989223	928807
1283	1 Q9H2U1	Q9H2U1	ATP-dependent DNA/RNA helicase DHX36	DHX36	Homo sapiens	114.762	16	16	1738203	540869
1284	1 Q9H425	Q9H425	Uncharacterized protein C1orf198	C1orf198	Homo sapiens	36.346	16	16	4527608	30854
1285	1 Q9H4A3	Q9H4A3	Serine/threonine-protein kinase WNK1	WNK1	Homo sapiens	250.797	21	16	2246348	1334386
1286	1 Q9HCG7	Q9HCG7	Non-lysosomal glucosylceramidase	GBA2	Homo sapiens	104.648	16	16	2456321	0
1287	1 Q9NP81	Q9NP81	Serine--tRNA ligase, mitochondrial	SARS2	Homo sapiens	58.284	16	16	5525608	1219639
1288	1 Q9NQC3	Q9NQC3	Reticulon-4	RTN4	Homo sapiens	129.933	16	16	16479731	7173112
1289	1 Q9NQX3	Q9NQX3	Gephyrin	GPHN	Homo sapiens	79.749	16	16	5589164	919799
1290	1 Q9NRW7	Q9NRW7	Vacuolar protein sorting-associated protein 45	VPS45	Homo sapiens	65.075	16	16	5515087	2152837
1291	1 Q9P2B4	Q9P2B4	CTTNBP2 N-terminal-like protein	CTTNBP2NL	Homo sapiens	70.157	16	16	1559240	352659
1292	1 Q9UBF2	Q9UBF2	Coatomer subunit gamma-2	COPG2	Homo sapiens	97.623	16	16	3215414	2815457
1293	1 Q9UBU9	Q9UBU9	Nuclear RNA export factor 1	NXF1	Homo sapiens	70.183	16	16	2353217	1347686
1294	1 Q9UDR5	Q9UDR5	Alpha-aminoadipic semialdehyde synthase, mitochondrial	AASS	Homo sapiens	102.131	16	16	1936654	0
1295	1 Q9UGU0	Q9UGU0	Transcription factor 20	TCF20	Homo sapiens	211.775	16	16	801933	29514
1296	1 Q9UH65	Q9UH65	Switch-associated protein 70	SWAP70	Homo sapiens	68.998	16	16	3726876	3231350
1297	1 Q9UI12	Q9UI12	V-type proton ATPase subunit H	ATP6V1H	Homo sapiens	55.885	16	16	5276659	3377618
1298	1 Q9UKN1	Q9UKN1	Mucin-12	MUC12	Homo sapiens	558.175	16	16	3265784	0
1299	1 Q9UL18	Q9UL18	Protein argonaute-1	AGO1	Homo sapiens	97.216	25	16	2755178	269833
1300	1 Q9UNN5	Q9UNN5	FAS-associated factor 1	FAF1	Homo sapiens	73.957	16	16	3940619	859499
1301	1 Q9UPQ9	Q9UPQ9	Trinucleotide repeat-containing gene 6B protein	TNRC6B	Homo sapiens	194.004	16	16	1342308	88355
1302	1 Q9UPT8	Q9UPT8	Zinc finger CCCH domain-containing protein 4	ZC3H4	Homo sapiens	140.26	16	16	2238808	740512
1303	1 Q9Y2G3	Q9Y2G3	Phospholipid-transporting ATPase IF	ATP11B	Homo sapiens	134.189	16	16	635633	4776293
1304	1 Q9Y3I0	Q9Y3I0	RNA-splicing ligase RtcB homolog	RTCB	Homo sapiens	55.209	16	16	27747894	10107744
1305	1 Q9Y696	Q9Y696	Chloride intracellular channel protein 4	CLIC4	Homo sapiens	28.771	16	16	40093057	7354322
1306	1 Q9Y6D9	Q9Y6D9	Mitotic spindle assembly checkpoint protein MAD1	MAD1L1	Homo sapiens	83.067	16	16	5241464	988648
1307	1 Q9Y6G9	Q9Y6G9	Cytoplasmic dynein 1 light intermediate chain 1	DYNC1LI1	Homo sapiens	56.579	17	16	7409119	4905406
1308	1 A1X283	A1X283	SH3 and PX domain-containing protein 2B	SH3PXD2B	Homo sapiens	101.58	16	15	5672841	2025923
1309	1 O00170	O00170	AH receptor-interacting protein	AIP	Homo sapiens	37.665	15	15	5753198	2772271
1310	1 O00299	O00299	Chloride intracellular channel protein 1	CLIC1	Homo sapiens	26.922	15	15	55788220	58807557
1311	1 O00562	O00562	Membrane-associated phosphatidylinositol transfer protein 1	PITPNM1	Homo sapiens	134.848	15	15	359736	1289201
1312	1 O00748	O00748	Cocaine esterase	CES2	Homo sapiens	61.807	15	15	48783069	927036
1313	1 O15075	O15075	Serine/threonine-protein kinase DCLK1	DCLK1	Homo sapiens	82.223	16	15	3213700	14078
1314	1 O15357	O15357	Phosphatidylinositol 3,4,5-trisphosphate 5-phosphatase 2	INPPL1	Homo sapiens	138.6	15	15	1657745	727032
1315	1 O15371	O15371	Eukaryotic translation initiation factor 3 subunit D	EIF3D	Homo sapiens	63.974	15	15	12248186	6062525
1316	1 O43175	O43175	D-3-phosphoglycerate dehydrogenase	PHGDH	Homo sapiens	56.652	15	15	12541376	4562956
1317	1 O43182	O43182	Rho GTPase-activating protein 6	ARHGAP6	Homo sapiens	105.948	15	15	1911272	0
1318	1 O43278	O43278	Kunitz-type protease inhibitor 1	SPINT1	Homo sapiens	58.4	15	15	3921191	4522659
1319	1 O43290	O43290	U4/U6.U5 tri-snRNP-associated protein 1	SART1	Homo sapiens	90.255	15	15	2040081	508156
1320	1 O43310	O43310	CBP80/20-dependent translation initiation factor	CTIF	Homo sapiens	67.588	15	15	1955220	0
1321	1 O43396	O43396	Thioredoxin-like protein 1	TXNL1	Homo sapiens	32.251	15	15	23852634	6034545

1322	1	O43747	O43747	AP-1 complex subunit gamma-1	AP1G1	Homo sapiens	91.353	15	15	5316393	7442465
1323	1	O43776	O43776	Asparagine--tRNA ligase, cytoplasmic	NARS1	Homo sapiens	62.943	15	15	20581320	8516273
1324	1	O43852	O43852	Calumenin	CALU	Homo sapiens	37.104	15	15	36894162	16640429
1325	1	O60449	O60449	Lymphocyte antigen 75	LY75	Homo sapiens	198.315	15	15	2045573	2388343
1326	1	O60502	O60502	Protein O-GlcNAcase	OGA	Homo sapiens	102.919	15	15	2163977	1284984
1327	1	O60506	O60506	Heterogeneous nuclear ribonucleoprotein Q	SYNCRIP	Homo sapiens	69.605	15	15	25085192	10327779
1328	1	O60825	O60825	6-phosphofructo-2-kinase/fructose-2,6-bisphosphatase 2	PFKFB2	Homo sapiens	58.477	20	15	4131870	2729977
1329	1	O60879	O60879	Protein diaphanous homolog 2	DIAPH2	Homo sapiens	125.571	15	15	2766501	1705212
1330	1	O75131	O75131	Copine-3	CPNE3	Homo sapiens	60.129	15	15	17482131	36472404
1331	1	O75891	O75891	Cytosolic 10-formyltetrahydrofolate dehydrogenase	ALDH1L1	Homo sapiens	98.831	15	15	1574802	747585
1332	1	O94760	O94760	N(G),N(G)-dimethylarginine dimethylaminohydrolase 1	DDAH1	Homo sapiens	31.121	15	15	36888559	3695421
1333	1	O94826	O94826	Mitochondrial import receptor subunit TOM70	TOMM70	Homo sapiens	67.454	15	15	9823520	1319235
1334	1	O94851	O94851	[F-actin]-monooxygenase MICAL2	MICAL2	Homo sapiens	219.064	15	15	4281893	456208
1335	1	O95486	O95486	Protein transport protein Sec24A	SEC24A	Homo sapiens	119.75	15	15	3579104	2413325
1336	1	O95487	O95487	Protein transport protein Sec24B	SEC24B	Homo sapiens	137.417	15	15	3051557	1204235
1337	1	O95602	O95602	DNA-directed RNA polymerase I subunit RPA1	POLR1A	Homo sapiens	194.815	15	15	980865	55895
1338	1	O95613	O95613	Pericentrin	PCNT	Homo sapiens	378.042	15	15	808485	0
1339	1	O95834	O95834	Echinoderm microtubule-associated protein-like 2	EML2	Homo sapiens	70.68	17	15	11274514	7983199
1340	1	P00736	P00736	Complement C1r subcomponent	C1R	Homo sapiens	80.119	15	15	4099356	7937952
1341	1	P02649	P02649	Apolipoprotein E	APOE	Homo sapiens	36.153	17	15	5590536	21025775
1342	1	P02774	P02774	Vitamin D-binding protein	GC	Homo sapiens	52.918	18	15	32064982	91619088
1343	1	P05155	P05155	Plasma protease C1 inhibitor	SERPING1	Homo sapiens	55.154	15	15	56825695	98354288
1344	1	P05156	P05156	Complement factor I	CFI	Homo sapiens	65.75	16	15	9385008	14736691
1345	1	P08240	P08240	Signal recognition particle receptor subunit alpha	SRPRA	Homo sapiens	69.812	15	15	4415392	2756030
1346	1	P10644	P10644	cAMP-dependent protein kinase type I-alpha regulatory subunit	PRKAR1A	Homo sapiens	42.981	17	15	18605776	15464147
1347	1	P11137	P11137	Microtubule-associated protein 2	MAP2	Homo sapiens	199.529	15	15	2241118	0
1348	1	P11678	P11678	Eosinophil peroxidase	EPX	Homo sapiens	81.042	15	15	6138653	854057
1349	1	P13716	P13716	Delta-aminolevulinic acid dehydratase	ALAD	Homo sapiens	36.294	15	15	8291970	3660608
1350	1	P13798	P13798	Acylamino-acid-releasing enzyme	APEH	Homo sapiens	81.229	15	15	22588790	10823681
1351	1	P13861	P13861	cAMP-dependent protein kinase type II-alpha regulatory subunit	PRKAR2A	Homo sapiens	45.52	17	15	12271323	4612847
1352	1	P14866	P14866	Heterogeneous nuclear ribonucleoprotein L	HNRNPL	Homo sapiens	64.135	15	15	30419910	18472739
1353	1	P16333	P16333	Cytoplasmic protein NCK1	NCK1	Homo sapiens	42.865	15	15	4439051	2892778
1354	1	P18124	P18124	60S ribosomal protein L7	RPL7	Homo sapiens	29.225	15	15	69663951	27962779
1355	1	P20020	P20020	Plasma membrane calcium-transporting ATPase 1	ATP2B1	Homo sapiens	134.687	16	15	7891932	1791054
1356	1	P21283	P21283	V-type proton ATPase subunit C 1	ATP6V1C1	Homo sapiens	43.943	15	15	3325156	3671555
1357	1	P22307	P22307	Sterol carrier protein 2	SCP2	Homo sapiens	58.995	15	15	23322497	9331052
1358	1	P22681	P22681	E3 ubiquitin-protein ligase CBL	CBL	Homo sapiens	99.634	16	15	1391029	2542348
1359	1	P23396	P23396	40S ribosomal protein S3	RPS3	Homo sapiens	26.684	15	15	68862415	23622335
1360	1	P23786	P23786	Carnitine O-palmitoyltransferase 2, mitochondrial	CPT2	Homo sapiens	73.779	15	15	10254910	2197568
1361	1	P28290	P28290	Protein ITPRID2	ITPRID2	Homo sapiens	138.387	15	15	2055260	1179103
1362	1	P29692	P29692	Elongation factor 1-delta	EEF1D	Homo sapiens	31.122	16	15	29500567	13196118
1363	1	P30153	P30153	Serine/threonine-protein phosphatase 2A 65 kDa regulatory subunit A alpha isoform	PPP2R1A	Homo sapiens	65.31	20	15	28274925	11164440
1364	1	P35241	P35241	Radixin	RDX	Homo sapiens	68.564	15	15	8596860	2193967
1365	1	P35858	P35858	Insulin-like growth factor-binding protein complex acid labile subunit	IGFALS	Homo sapiens	66.033	15	15	3328922	7127033
1366	1	P38919	P38919	Eukaryotic initiation factor 4A-III	EIF4A3	Homo sapiens	46.871	15	15	14098628	8135771
1367	1	P43155	P43155	Carnitine O-acetyltransferase	CRAT	Homo sapiens	70.859	15	15	6355168	2835376
1368	1	P46060	P46060	Ran GTPase-activating protein 1	RANGAP1	Homo sapiens	63.543	15	15	3891738	10828737

1369	1	P46934	P46934	E3 ubiquitin-protein ligase NEDD4	NEDD4	Homo sapiens	149.118	19	15	39652346	95026967
1370	1	P47756	P47756	F-actin-capping protein subunit beta	CAPZB	Homo sapiens	30.629	15	15	85167940	53966691
1371	1	P49189	P49189	4-trimethylaminobutyraldehyde dehydrogenase	ALDH9A1	Homo sapiens	53.801	15	15	40071560	10424546
1372	1	P49754	P49754	Vacuolar protein sorting-associated protein 41 homolog	VPS41	Homo sapiens	98.567	15	15	1172099	2163935
1373	1	P50579	P50579	Methionine aminopeptidase 2	METAP2	Homo sapiens	52.895	15	15	4653261	2007322
1374	1	P51003	P51003	Poly(A) polymerase alpha	PAPOLA	Homo sapiens	82.843	20	15	1369261	566341
1375	1	P51116	P51116	RNA-binding protein FXR2	FXR2	Homo sapiens	74.223	17	15	1410326	995470
1376	1	P53992	P53992	Protein transport protein Sec24C	SEC24C	Homo sapiens	118.325	17	15	12731138	4762285
1377	1	P54578	P54578	Ubiquitin carboxyl-terminal hydrolase 14	USP14	Homo sapiens	56.069	15	15	16308265	9655227
1378	1	P54819	P54819	Adenylate kinase 2, mitochondrial	AK2	Homo sapiens	26.478	15	15	22498967	11367656
1379	1	P61011	P61011	Signal recognition particle subunit SRP54	SRP54	Homo sapiens	55.703	15	15	5671792	1975362
1380	1	P61106	P61106	Ras-related protein Rab-14	RAB14	Homo sapiens	23.898	17	15	22285744	13032721
1381	1	P61221	P61221	ATP-binding cassette sub-family E member 1	ABCE1	Homo sapiens	67.315	15	15	7107603	2816993
1382	1	P83436	P83436	Conserved oligomeric Golgi complex subunit 7	COG7	Homo sapiens	86.345	15	15	3402660	1088108
1383	1	Q03169	Q03169	Tumor necrosis factor alpha-induced protein 2	TNFAIP2	Homo sapiens	72.663	15	15	2177666	4279581
1384	1	Q05193	Q05193	Dynamin-1	DNM1	Homo sapiens	97.409	16	15	3484252	129502
1385	1	Q06033	Q06033	Inter-alpha-trypsin inhibitor heavy chain H3	ITIH3	Homo sapiens	99.849	17	15	11526682	30295479
1386	1	Q06187	Q06187	Tyrosine-protein kinase BTK	BTK	Homo sapiens	76.28	15	15	722780	3070137
1387	1	Q07889	Q07889	Son of sevenless homolog 1	SOS1	Homo sapiens	152.466	20	15	1576564	456246
1388	1	Q08945	Q08945	FACT complex subunit SSRP1	SSRP1	Homo sapiens	81.075	15	15	6247297	2767606
1389	1	Q12805	Q12805	EGF-containing fibulin-like extracellular matrix protein 1	EFEMP1	Homo sapiens	54.641	15	15	12348722	13857153
1390	1	Q13098	Q13098	COP9 signalosome complex subunit 1	GPS1	Homo sapiens	55.537	15	15	7217155	2224510
1391	1	Q13107	Q13107	Ubiquitin carboxyl-terminal hydrolase 4	USP4	Homo sapiens	108.566	16	15	967391	1155945
1392	1	Q13231	Q13231	Chitotriosidase-1	CHIT1	Homo sapiens	51.681	15	15	220090	10509042
1393	1	Q13242	Q13242	Serine/arginine-rich splicing factor 9	SRSF9	Homo sapiens	25.544	15	15	8427913	5469009
1394	1	Q13561	Q13561	Dynactin subunit 2	DCTN2	Homo sapiens	44.23	15	15	21119596	4721081
1395	1	Q13620	Q13620	Cullin-4B	CUL4B	Homo sapiens	103.981	21	15	2374097	1195706
1396	1	Q13740	Q13740	CD166 antigen	ALCAM	Homo sapiens	65.102	15	15	5989939	382162
1397	1	Q14149	Q14149	MORC family CW-type zinc finger protein 3	MORC3	Homo sapiens	107.115	15	15	1855797	798145
1398	1	Q14644	Q14644	Ras GTPase-activating protein 3	RASA3	Homo sapiens	95.7	15	15	1181265	155175
1399	1	Q14CX7	Q14CX7	N-alpha-acetyltransferase 25, NatB auxiliary subunit	NAA25	Homo sapiens	112.293	15	15	1354148	555801
1400	1	Q15147	Q15147	1-phosphatidylinositol 4,5-bisphosphate phosphodiesterase beta-4	PLCB4	Homo sapiens	134.467	15	15	1662331	0
1401	1	Q15435	Q15435	Protein phosphatase 1 regulatory subunit 7	PPP1R7	Homo sapiens	41.564	15	15	14810460	5124366
1402	1	Q15813	Q15813	Tubulin-specific chaperone E	TBCE	Homo sapiens	59.348	15	15	1787403	577226
1403	1	Q16352	Q16352	Alpha-internexin	INA	Homo sapiens	55.392	16	15	7678490	19569
1404	1	Q16512	Q16512	Serine/threonine-protein kinase N1	PKN1	Homo sapiens	103.933	15	15	1832508	3750295
1405	1	Q32P44	Q32P44	Echinoderm microtubule-associated protein-like 3	EML3	Homo sapiens	95.2	15	15	1712620	1097934
1406	1	Q3LXA3	Q3LXA3	Triokinase/FMN cyclase	TKFC	Homo sapiens	58.946	15	15	4745006	4154287
1407	1	Q53FZ2	Q53FZ2	Acyl-coenzyme A synthetase ACSM3, mitochondrial	ACSM3	Homo sapiens	66.152	15	15	3386161	1486851
1408	1	Q5SSJ5	Q5SSJ5	Heterochromatin protein 1-binding protein 3	HP1BP3	Homo sapiens	61.209	15	15	43321985	18295146
1409	1	Q5T5U3	Q5T5U3	Rho GTPase-activating protein 21	ARHGAP21	Homo sapiens	217.468	15	15	803496	100323
1410	1	Q5THJ4	Q5THJ4	Intermembrane lipid transfer protein VPS13D	VPS13D	Homo sapiens	491.923	15	15	650492	103441
1411	1	Q5VV41	Q5VV41	Rho guanine nucleotide exchange factor 16	ARHGEF16	Homo sapiens	80.104	15	15	4595890	196640
1412	1	Q5VYS8	Q5VYS8	Terminal uridylyltransferase 7	TUT7	Homo sapiens	171.231	16	15	464933	1328972
1413	1	Q6IA69	Q6IA69	Glutamine-dependent NAD(+) synthetase	NADSYN1	Homo sapiens	79.284	15	15	3899473	2201506
1414	1	Q6IBS0	Q6IBS0	Twinfilin-2	TWF2	Homo sapiens	39.548	16	15	7411358	7285240
1415	1	Q6NZY4	Q6NZY4	Zinc finger CCHC domain-containing protein 8	ZCCHC8	Homo sapiens	78.578	15	15	3021100	455041
1416	1	Q6PCE3	Q6PCE3	Glucose 1,6-bisphosphate synthase	PGM2L1	Homo sapiens	70.441	15	15	5879315	1915644

1417	1 Q6PI48	Q6PI48	Aspartate--tRNA ligase, mitochondrial	DARS2	Homo sapiens	73.563	15	15	7080572	1666520
1418	1 Q6PJG2	Q6PJG2	Mitotic deacetylase-associated SANT domain protein	MIDEAS	Homo sapiens	114.991	15	15	1438108	19500
1419	1 Q7L0Y3	Q7L0Y3	tRNA methyltransferase 10 homolog C	TRMT10C	Homo sapiens	47.347	15	15	4444091	1211733
1420	1 Q7Z2K6	Q7Z2K6	Endoplasmic reticulum metallopeptidase 1	ERMP1	Homo sapiens	100.233	15	15	6415793	8487304
1421	1 Q7Z392	Q7Z392	Trafficking protein particle complex subunit 11	TRAPPC11	Homo sapiens	128.883	15	15	1746245	1658297
1422	1 Q86TU7	Q86TU7	Actin-histidine N-methyltransferase	SETD3	Homo sapiens	67.26	15	15	3477129	1099492
1423	1 Q86UY8	Q86UY8	5'-nucleotidase domain-containing protein 3	NT5DC3	Homo sapiens	63.419	15	15	5742578	574085
1424	1 Q8IW45	Q8IW45	ATP-dependent (S)-NAD(P)H-hydrate dehydratase	NAXD	Homo sapiens	36.575	15	15	4391700	135671
1425	1 Q8IWB7	Q8IWB7	WD repeat and FYVE domain-containing protein 1	WDFY1	Homo sapiens	46.323	15	15	2810884	3007637
1426	1 Q8IWE2	Q8IWE2	Protein NOXP20	FAM114A1	Homo sapiens	60.743	15	15	9710368	1446586
1427	1 Q8IYI6	Q8IYI6	Exocyst complex component 8	EXOC8	Homo sapiens	81.799	15	15	3203089	1714697
1428	1 Q8N163	Q8N163	Cell cycle and apoptosis regulator protein 2	CCAR2	Homo sapiens	102.905	15	15	8311174	3780381
1429	1 Q8N1I0	Q8N1I0	Dedicator of cytokinesis protein 4	DOCK4	Homo sapiens	225.21	15	15	894293	915816
1430	1 Q8N392	Q8N392	Rho GTPase-activating protein 18	ARHGAP18	Homo sapiens	74.977	15	15	1766437	1214532
1431	1 Q8N6H7	Q8N6H7	ADP-ribosylation factor GTPase-activating protein 2	ARFGAP2	Homo sapiens	56.72	16	15	5609428	2273561
1432	1 Q8NEN9	Q8NEN9	PDZ domain-containing protein 8	PDZD8	Homo sapiens	128.563	15	15	1025530	838865
1433	1 Q8TAF3	Q8TAF3	WD repeat-containing protein 48	WDR48	Homo sapiens	76.211	15	15	2437226	469228
1434	1 Q8TAQ2	Q8TAQ2	SWI/SNF complex subunit SMARCC2	SMARCC2	Homo sapiens	132.881	22	15	4977695	2011700
1435	1 Q8TC07	Q8TC07	TBC1 domain family member 15	TBC1D15	Homo sapiens	79.49	15	15	2964880	2470110
1436	1 Q8TEH3	Q8TEH3	DENN domain-containing protein 1A	DENND1A	Homo sapiens	110.58	15	15	1513382	435759
1437	1 Q92541	Q92541	RNA polymerase-associated protein RTF1 homolog	RTF1	Homo sapiens	80.315	15	15	2968455	1085597
1438	1 Q969V3	Q969V3	BOS complex subunit NCLN	NCLN	Homo sapiens	62.974	15	15	4509401	3213291
1439	1 Q96A33	Q96A33	PAT complex subunit CCDC47	CCDC47	Homo sapiens	55.874	15	15	6107972	2494821
1440	1 Q96AY4	Q96AY4	Tetratricopeptide repeat protein 28	TTC28	Homo sapiens	270.888	15	15	675670	9410
1441	1 Q96BY6	Q96BY6	Dedicator of cytokinesis protein 10	DOCK10	Homo sapiens	249.535	15	15	1371852	544564
1442	1 Q96I99	Q96I99	Succinate--CoA ligase [GDP-forming] subunit beta, mitochondrial	SUCLG2	Homo sapiens	46.51	15	15	52110266	5267950
1443	1 Q96J02	Q96J02	E3 ubiquitin-protein ligase Itchy homolog	ITCH	Homo sapiens	102.801	19	15	2288386	1455694
1444	1 Q96KP1	Q96KP1	Exocyst complex component 2	EXOC2	Homo sapiens	104.066	15	15	2633453	1636763
1445	1 Q96ME7	Q96ME7	Zinc finger protein 512	ZNF512	Homo sapiens	64.682	15	15	4026881	1250623
1446	1 Q96PE3	Q96PE3	Inositol polyphosphate-4-phosphatase type I A	INPP4A	Homo sapiens	109.956	15	15	2645582	481911
1447	1 Q96RW7	Q96RW7	Hemicentin-1	HMCN1	Homo sapiens	613.4	15	15	395894	84862
1448	1 Q96T58	Q96T58	Msx2-interacting protein	SPEN	Homo sapiens	402.254	15	15	683349	29293
1449	1 Q99567	Q99567	Nuclear pore complex protein Nup88	NUP88	Homo sapiens	83.54	15	15	1805715	1999400
1450	1 Q99590	Q99590	Protein SCAF11	SCAF11	Homo sapiens	164.654	15	15	1565874	404436
1451	1 Q99615	Q99615	DnaJ homolog subfamily C member 7	DNAJC7	Homo sapiens	56.439	15	15	2402266	1762194
1452	1 Q9BTV4	Q9BTV4	Transmembrane protein 43	TMEM43	Homo sapiens	44.876	15	15	26155836	8265130
1453	1 Q9BUQ8	Q9BUQ8	Probable ATP-dependent RNA helicase DDX23	DDX23	Homo sapiens	95.582	15	15	4432014	2071700
1454	1 Q9BZZ5	Q9BZZ5	Apoptosis inhibitor 5	API5	Homo sapiens	59.005	15	15	12679886	3771342
1455	1 Q9C040	Q9C040	Tripartite motif-containing protein 2	TRIM2	Homo sapiens	81.532	16	15	3210259	178859
1456	1 Q9C0E2	Q9C0E2	Exportin-4	XPO4	Homo sapiens	130.139	15	15	1913787	788493
1457	1 Q9GZR7	Q9GZR7	ATP-dependent RNA helicase DDX24	DDX24	Homo sapiens	96.333	15	15	1588837	477194
1458	1 Q9H792	Q9H792	Inactive tyrosine-protein kinase PEAK1	PEAK1	Homo sapiens	193.108	15	15	1031502	226143
1459	1 Q9HBR0	Q9HBR0	Putative sodium-coupled neutral amino acid transporter 10	SLC38A10	Homo sapiens	119.764	15	15	556185	1272946
1460	1 Q9HCE6	Q9HCE6	Rho guanine nucleotide exchange factor 10-like protein	ARHGEF10L	Homo sapiens	140.381	15	15	10571575	664085
1461	1 Q9NPQ8	Q9NPQ8	Synembryn-A	RIC8A	Homo sapiens	59.708	16	15	3555046	770533
1462	1 Q9NR45	Q9NR45	Sialic acid synthase	NANS	Homo sapiens	40.31	15	15	57176403	10069087
1463	1 Q9NTZ6	Q9NTZ6	RNA-binding protein 12	RBM12	Homo sapiens	97.397	15	15	6104519	2308174
1464	1 Q9NUQ8	Q9NUQ8	ATP-binding cassette sub-family F member 3	ABCF3	Homo sapiens	79.746	15	15	2146779	1166911

1465	1	Q9NXC5	Q9NXC5	GATOR complex protein MIOS	MIOS	Homo sapiens	98.584	15	15	1674090	360253
1466	1	Q9NY33	Q9NY33	Dipeptidyl peptidase 3	DPP3	Homo sapiens	82.589	15	15	10713310	7521141
1467	1	Q9NZU5	Q9NZU5	LIM and cysteine-rich domains protein 1	LMCD1	Homo sapiens	40.834	15	15	5041006	2059245
1468	1	Q9UBB9	Q9UBB9	Tuftelin-interacting protein 11	TFIP11	Homo sapiens	96.821	15	15	2266200	657521
1469	1	Q9UHD2	Q9UHD2	Serine/threonine-protein kinase TBK1	TBK1	Homo sapiens	83.644	15	15	2391871	3371281
1470	1	Q9ULA0	Q9ULA0	Aspartyl aminopeptidase	DNPEP	Homo sapiens	53.412	15	15	18018944	3241901
1471	1	Q9ULE6	Q9ULE6	Paladin	PALD1	Homo sapiens	96.754	15	15	1282721	29370
1472	1	Q9ULV4	Q9ULV4	Coronin-1C	CORO1C	Homo sapiens	53.248	17	15	50312796	6881991
1473	1	Q9UNM6	Q9UNM6	26S proteasome non-ATPase regulatory subunit 13	PSMD13	Homo sapiens	42.944	15	15	9796043	7876839
1474	1	Q9UP83	Q9UP83	Conserved oligomeric Golgi complex subunit 5	COG5	Homo sapiens	92.743	15	15	2971315	484401
1475	1	Q9UPW6	Q9UPW6	DNA-binding protein SATB2	SATB2	Homo sapiens	82.557	18	15	9312137	0
1476	1	Q9UQ80	Q9UQ80	Proliferation-associated protein 2G4	PA2G4	Homo sapiens	43.785	15	15	28367527	17574513
1477	1	Q9UQB8	Q9UQB8	Brain-specific angiogenesis inhibitor 1-associated protein 2	BAIAP2	Homo sapiens	60.868	15	15	3130409	270344
1478	1	Q9Y262	Q9Y262	Eukaryotic translation initiation factor 3 subunit L	EIF3L	Homo sapiens	66.726	15	15	8713985	4437169
1479	1	Q9Y266	Q9Y266	Nuclear migration protein nudC	NUDC	Homo sapiens	38.244	15	15	18542774	6701361
1480	1	Q9Y276	Q9Y276	Mitochondrial chaperone BCS1	BCS1L	Homo sapiens	47.537	15	15	3607361	606320
1481	1	Q9Y2L1	Q9Y2L1	Exosome complex exonuclease RRP44	DIS3	Homo sapiens	109.004	15	15	2667793	1309680
1482	1	Q9Y4K0	Q9Y4K0	Lysyl oxidase homolog 2	LOXL2	Homo sapiens	86.728	15	15	914515	4417896
1483	1	Q9Y5Y6	Q9Y5Y6	Suppressor of tumorigenicity 14 protein	ST14	Homo sapiens	94.771	15	15	5831793	587080
1484	1	Q9Y618	Q9Y618	Nuclear receptor corepressor 2	NCOR2	Homo sapiens	273.66	15	15	1223028	46787
1485	1	A7E2V4	A7E2V4	Zinc finger SWIM domain-containing protein 8	ZSWIM8	Homo sapiens	197.301	14	14	1322151	236738
1486	1	O00192	O00192	Splicing regulator ARVCF	ARVCF	Homo sapiens	104.641	14	14	697234	0
1487	1	O00754	O00754	Lysosomal alpha-mannosidase	MAN2B1	Homo sapiens	113.743	14	14	4367933	3878534
1488	1	O15013	O15013	Rho guanine nucleotide exchange factor 10	ARHGEF10	Homo sapiens	151.614	14	14	1847678	235397
1489	1	O15117	O15117	FYN-binding protein 1	FYB1	Homo sapiens	85.388	14	14	508487	4411694
1490	1	O15270	O15270	Serine palmitoyltransferase 2	SPTLC2	Homo sapiens	62.926	14	14	1835719	911695
1491	1	O15344	O15344	E3 ubiquitin-protein ligase Midline-1	MID1	Homo sapiens	75.252	16	14	2069573	0
1492	1	O43166	O43166	Signal-induced proliferation-associated 1-like protein 1	SIPA1L1	Homo sapiens	200.03	14	14	725574	677593
1493	1	O43264	O43264	Centromere/kinetochore protein zw10 homolog	ZW10	Homo sapiens	88.83	14	14	1660976	927865
1494	1	O43837	O43837	Isocitrate dehydrogenase [NAD] subunit beta, mitochondrial	IDH3B	Homo sapiens	42.184	14	14	9604919	1693127
1495	1	O43847	O43847	Nardilysin	NRDC	Homo sapiens	131.705	14	14	1465912	1687444
1496	1	O60664	O60664	Perilipin-3	PLIN3	Homo sapiens	47.075	14	14	20427547	15346788
1497	1	O75312	O75312	Zinc finger protein ZPR1	ZPR1	Homo sapiens	50.924	14	14	2794335	1007668
1498	1	O75427	O75427	Leucine-rich repeat and calponin homology domain-containing protein 4	LRCH4	Homo sapiens	73.451	14	14	485916	2943633
1499	1	O75582	O75582	Ribosomal protein S6 kinase alpha-5	RPS6KA5	Homo sapiens	89.865	14	14	3790764	563657
1500	1	O95299	O95299	NADH dehydrogenase [ubiquinone] 1 alpha subcomplex subunit 10, mitochondrial	NDUFA10	Homo sapiens	40.751	14	14	24632186	3923639
1501	1	O95394	O95394	Phosphoacetylglucosamine mutase	PGM3	Homo sapiens	59.851	14	14	1978146	1301972
1502	1	O95816	O95816	BAG family molecular chaperone regulator 2	BAG2	Homo sapiens	23.771	14	14	15986018	284688
1503	1	O95861	O95861	3'(2'),5'-bisphosphate nucleotidase 1	BPNT1	Homo sapiens	33.392	14	14	9028376	5173903
1504	1	P00338	P00338	L-lactate dehydrogenase A chain	LDHA	Homo sapiens	36.688	18	14	165869207	150159384
1505	1	P01871	P01871	Immunoglobulin heavy constant mu	IGHM	Homo sapiens	49.44	14	14	137318714	333986085
1506	1	P04004	P04004	Vitronectin	VTN	Homo sapiens	54.305	16	14	51309708	693867211
1507	1	P04406	P04406	Glyceraldehyde-3-phosphate dehydrogenase	GAPDH	Homo sapiens	36.054	14	14	1016096847	840114136
1508	1	P04844	P04844	Dolichyl-diphosphooligosaccharide--protein glycosyltransferase subunit 2	RPN2	Homo sapiens	69.284	14	14	21459248	11728590
1509	1	P05060	P05060	Secretogranin-1	CHGB	Homo sapiens	78.278	14	14	2134641	0
1510	1	P05198	P05198	Eukaryotic translation initiation factor 2 subunit 1	EIF2S1	Homo sapiens	36.114	14	14	12943729	6191356

1511	1	P08195	P08195	4F2 cell-surface antigen heavy chain	SLC3A2	Homo sapiens	67.995	14	14	3284148	6008513
1512	1	P08311	P08311	Cathepsin G	CTSG	Homo sapiens	28.836	14	14	30361725	492561030
1513	1	P09211	P09211	Glutathione S-transferase P	GSTP1	Homo sapiens	23.355	14	14	86013030	61039634
1514	1	P09619	P09619	Platelet-derived growth factor receptor beta	PDGFRB	Homo sapiens	123.97	14	14	2447641	304383
1515	1	P09871	P09871	Complement C1s subcomponent	C1S	Homo sapiens	76.687	14	14	9194248	31603026
1516	1	P10253	P10253	Lysosomal alpha-glucosidase	GAA	Homo sapiens	105.326	14	14	4009635	1501728
1517	1	P12830	P12830	Cadherin-1	CDH1	Homo sapiens	97.457	14	14	16878765	3331184
1518	1	P13497	P13497	Bone morphogenetic protein 1	BMP1	Homo sapiens	111.25	17	14	0	4946253
1519	1	P13807	P13807	Glycogen [starch] synthase, muscle	GYS1	Homo sapiens	83.788	15	14	3670619	10507494
1520	1	P14550	P14550	Aldo-keto reductase family 1 member A1	AKR1A1	Homo sapiens	36.573	14	14	32330682	11158256
1521	1	P18433	P18433	Receptor-type tyrosine-protein phosphatase alpha	PTPRA	Homo sapiens	90.721	14	14	2476696	360277
1522	1	P19320	P19320	Vascular cell adhesion protein 1	VCAM1	Homo sapiens	81.276	14	14	1417257	734808
1523	1	P23588	P23588	Eukaryotic translation initiation factor 4B	EIF4B	Homo sapiens	69.151	14	14	5170532	1945983
1524	1	P25311	P25311	Zinc-alpha-2-glycoprotein	AZGP1	Homo sapiens	34.26	14	14	20398174	30051592
1525	1	P28288	P28288	ATP-binding cassette sub-family D member 3	ABCD3	Homo sapiens	75.478	14	14	5653021	3194912
1526	1	P29536	P29536	Leiomodin-1	LMOD1	Homo sapiens	67.033	14	14	63235201	288935
1527	1	P30837	P30837	Aldehyde dehydrogenase X, mitochondrial	ALDH1B1	Homo sapiens	57.25	14	14	29393342	1021340
1528	1	P31146	P31146	Coronin-1A	CORO1A	Homo sapiens	51.029	14	14	15193052	145360216
1529	1	P31930	P31930	Cytochrome b-c1 complex subunit 1, mitochondrial	UQCRC1	Homo sapiens	52.649	14	14	38554882	10370683
1530	1	P34897	P34897	Serine hydroxymethyltransferase, mitochondrial	SHMT2	Homo sapiens	55.994	15	14	7885439	5317340
1531	1	P34913	P34913	Bifunctional epoxide hydrolase 2	EPHX2	Homo sapiens	62.616	14	14	15929338	510630
1532	1	P35354	P35354	Prostaglandin G/H synthase 2	PTGS2	Homo sapiens	68.996	14	14	55515	6398297
1533	1	P36222	P36222	Chitinase-3-like protein 1	CHI3L1	Homo sapiens	42.624	14	14	1338842	26619970
1534	1	P41240	P41240	Tyrosine-protein kinase CSK	CSK	Homo sapiens	50.707	14	14	5187972	8408579
1535	1	P46777	P46777	60S ribosomal protein L5	RPL5	Homo sapiens	34.361	14	14	30277448	12092961
1536	1	P51149	P51149	Ras-related protein Rab-7a	RAB7A	Homo sapiens	23.488	14	14	38894950	53122725
1537	1	P51178	P51178	1-phosphatidylinositol 4,5-bisphosphate phosphodiesterase delta-1	PLCD1	Homo sapiens	85.663	14	14	4209030	611503
1538	1	P51858	P51858	Hepatoma-derived growth factor	HDGF	Homo sapiens	26.789	14	14	21448151	16054565
1539	1	P52306	P52306	Rap1 GTPase-GDP dissociation stimulator 1	RAP1GDS1	Homo sapiens	66.316	14	14	5811557	1919452
1540	1	P54920	P54920	Alpha-soluble NSF attachment protein	NAPA	Homo sapiens	33.233	16	14	17139103	13222474
1541	1	P55084	P55084	Trifunctional enzyme subunit beta, mitochondrial	HADHB	Homo sapiens	51.296	14	14	82968983	22283299
1542	1	P60174	P60174	Triosephosphate isomerase	TPI1	Homo sapiens	26.669	14	14	324846128	140105271
1543	1	P61160	P61160	Actin-related protein 2	ACTR2	Homo sapiens	44.762	14	14	35375512	35360750
1544	1	P62495	P62495	Eukaryotic peptide chain release factor subunit 1	ETF1	Homo sapiens	49.03	14	14	10995032	7398385
1545	1	P63010	P63010	AP-2 complex subunit beta	AP2B1	Homo sapiens	104.551	14	14	12764467	3417137
1546	1	P98196	P98196	Phospholipid-transporting ATPase IH	ATP11A	Homo sapiens	129.758	14	14	101901	2664732
1547	1	Q01518	Q01518	Adenylyl cyclase-associated protein 1	CAP1	Homo sapiens	51.9	22	14	37778945	36640628
1548	1	Q07960	Q07960	Rho GTPase-activating protein 1	ARHGAP1	Homo sapiens	50.435	14	14	23805901	7066547
1549	1	Q08188	Q08188	Protein-glutamine gamma-glutamyltransferase E	TGM3	Homo sapiens	76.631	14	14	38662	4738963
1550	1	Q08380	Q08380	Galectin-3-binding protein	LGALS3BP	Homo sapiens	65.332	14	14	15468760	14267850
1551	1	Q08752	Q08752	Peptidyl-prolyl cis-trans isomerase D	PPID	Homo sapiens	40.761	14	14	4117446	22041160
1552	1	Q09161	Q09161	Nuclear cap-binding protein subunit 1	NCBP1	Homo sapiens	91.842	14	14	3289185	1960239
1553	1	Q12788	Q12788	Transducin beta-like protein 3	TBL3	Homo sapiens	89.035	14	14	1347262	343800
1554	1	Q12884	Q12884	Prolyl endopeptidase FAP	FAP	Homo sapiens	87.714	14	14	290048	1306166
1555	1	Q12979	Q12979	Active breakpoint cluster region-related protein	ABR	Homo sapiens	97.597	15	14	3469349	1646195
1556	1	Q12996	Q12996	Cleavage stimulation factor subunit 3	CSTF3	Homo sapiens	82.923	14	14	2221852	887048
1557	1	Q13131	Q13131	5'-AMP-activated protein kinase catalytic subunit alpha-1	PRKAA1	Homo sapiens	64.009	16	14	7582018	4869666
1558	1	Q13283	Q13283	Ras GTPase-activating protein-binding protein 1	G3BP1	Homo sapiens	52.164	14	14	7710811	3367671

1559	1	Q13425	Q13425	Beta-2-syntrophin	SNTB2	Homo sapiens	57.951	14	14	12524753	1202240
1560	1	Q13573	Q13573	SNW domain-containing protein 1	SNW1	Homo sapiens	61.494	14	14	2904313	597426
1561	1	Q13619	Q13619	Cullin-4A	CUL4A	Homo sapiens	87.68	14	14	2786314	841291
1562	1	Q14005	Q14005	Pro-interleukin-16	IL16	Homo sapiens	141.752	14	14	3648871	10245102
1563	1	Q14161	Q14161	ARF GTPase-activating protein GIT2	GIT2	Homo sapiens	84.545	15	14	3137180	3965092
1564	1	Q14344	Q14344	Guanine nucleotide-binding protein subunit alpha-13	GNA13	Homo sapiens	44.05	15	14	9819216	6301510
1565	1	Q14671	Q14671	Pumilio homolog 1	PUM1	Homo sapiens	126.475	16	14	2314920	292225
1566	1	Q15031	Q15031	Leucine--tRNA ligase, mitochondrial	LARS2	Homo sapiens	101.976	14	14	2729982	563618
1567	1	Q15084	Q15084	Protein disulfide-isomerase A6	PDIA6	Homo sapiens	48.121	14	14	61258087	35126959
1568	1	Q15172	Q15172	Serine/threonine-protein phosphatase 2A 56 kDa regulatory subunit alpha isoform	PPP2R5A	Homo sapiens	56.195	15	14	3666980	3727616
1569	1	Q15750	Q15750	TGF-beta-activated kinase 1 and MAP3K7-binding protein 1	TAB1	Homo sapiens	54.645	14	14	2984252	857333
1570	1	Q16401	Q16401	26S proteasome non-ATPase regulatory subunit 5	PSMD5	Homo sapiens	56.195	14	14	11292250	3737190
1571	1	Q2LD37	Q2LD37	Bridge-like lipid transfer protein family member 1	BLTP1	Homo sapiens	555.493	14	14	583754	19755
1572	1	Q2UY09	Q2UY09	Collagen alpha-1(XXVIII) chain	COL28A1	Homo sapiens	116.66	14	14	8803259	0
1573	1	Q2WGJ9	Q2WGJ9	Fer-1-like protein 6	FER1L6	Homo sapiens	209.311	14	14	1527121	81754
1574	1	Q3YEC7	Q3YEC7	Rab-like protein 6	RABL6	Homo sapiens	79.549	14	14	6484578	1688971
1575	1	Q4G0J3	Q4G0J3	La-related protein 7	LARP7	Homo sapiens	66.898	14	14	1605099	775022
1576	1	Q4ZHG4	Q4ZHG4	Fibronectin type III domain-containing protein 1	FNDC1	Homo sapiens	205.561	14	14	1137448	0
1577	1	Q53EP0	Q53EP0	Fibronectin type III domain-containing protein 3B	FNDC3B	Homo sapiens	132.89	14	14	701336	16241854
1578	1	Q5HYW2	Q5HYW2	NHS-like protein 2	NHSL2	Homo sapiens	133.288	14	14	742161	1034384
1579	1	Q5T0N5	Q5T0N5	Formin-binding protein 1-like	FNBP1L	Homo sapiens	70.066	14	14	2804112	505982
1580	1	Q68DK2	Q68DK2	Zinc finger FYVE domain-containing protein 26	ZFYVE26	Homo sapiens	284.58	14	14	534234	1191950
1581	1	Q68EM7	Q68EM7	Rho GTPase-activating protein 17	ARHGAP17	Homo sapiens	95.437	14	14	6418216	2179750
1582	1	Q6DN90	Q6DN90	IQ motif and SEC7 domain-containing protein 1	IQSEC1	Homo sapiens	108.317	15	14	1351960	874265
1583	1	Q6NUM9	Q6NUM9	All-trans-retinol 13,14-reductase	RETSAT	Homo sapiens	66.82	14	14	6467379	1273922
1584	1	Q6P1J9	Q6P1J9	Parafibromin	CDC73	Homo sapiens	60.577	14	14	2315157	1075549
1585	1	Q6P1M0	Q6P1M0	Long-chain fatty acid transport protein 4	SLC27A4	Homo sapiens	72.067	15	14	4759914	1660647
1586	1	Q6ZU52	Q6ZU52	Uncharacterized protein KIAA0408	KIAA0408	Homo sapiens	79.162	14	14	1725397	19405
1587	1	Q70E73	Q70E73	Ras-associated and pleckstrin homology domains-containing protein 1	RAPH1	Homo sapiens	135.257	14	14	1560275	418541
1588	1	Q7Z5L7	Q7Z5L7	Podocan	PODN	Homo sapiens	68.975	14	14	9928480	0
1589	1	Q86UT6	Q86UT6	NLR family member X1	NLRX1	Homo sapiens	107.615	14	14	2092955	1379476
1590	1	Q86W42	Q86W42	THO complex subunit 6 homolog	THOC6	Homo sapiens	37.534	14	14	1674882	652284
1591	1	Q86YT6	Q86YT6	E3 ubiquitin-protein ligase MIB1	MIB1	Homo sapiens	110.137	15	14	1150030	288457
1592	1	Q8IUX7	Q8IUX7	Adipocyte enhancer-binding protein 1	AEBP1	Homo sapiens	130.931	14	14	8586589	3251751
1593	1	Q8IWZ8	Q8IWZ8	SURP and G-patch domain-containing protein 1	SUGP1	Homo sapiens	72.472	14	14	2418824	355789
1594	1	Q8IXQ6	Q8IXQ6	Protein mono-ADP-ribosyltransferase PARP9	PARP9	Homo sapiens	96.345	14	14	2025883	6887238
1595	1	Q8N0W3	Q8N0W3	L-fucose kinase	FCSK	Homo sapiens	117.622	14	14	1320780	454529
1596	1	Q8N1G2	Q8N1G2	Cap-specific mRNA (nucleoside-2'-O-)-methyltransferase 1	CMTR1	Homo sapiens	95.321	14	14	1601794	723207
1597	1	Q8N4A0	Q8N4A0	Polypeptide N-acetylgalactosaminyltransferase 4	GALNT4	Homo sapiens	66.666	15	14	3571708	132762
1598	1	Q8NBJ5	Q8NBJ5	Procollagen galactosyltransferase 1	COLGALT1	Homo sapiens	71.637	14	14	5383269	6179926
1599	1	Q8NBS9	Q8NBS9	Thioredoxin domain-containing protein 5	TXNDC5	Homo sapiens	47.628	14	14	58682168	11397453
1600	1	Q8ND71	Q8ND71	GTPase IMAP family member 8	GIMAP8	Homo sapiens	74.891	14	14	977243	4505830
1601	1	Q8NDX1	Q8NDX1	PH and SEC7 domain-containing protein 4	PSD4	Homo sapiens	116.25	14	14	75989	1298743
1602	1	Q8NFW8	Q8NFW8	N-acylneuraminate cytidylyltransferase	CMAS	Homo sapiens	48.381	14	14	8801365	891777
1603	1	Q8NI36	Q8NI36	WD repeat-containing protein 36	WDR36	Homo sapiens	105.324	14	14	1955054	305482
1604	1	Q8TEX9	Q8TEX9	Importin-4	IPO4	Homo sapiens	118.717	14	14	1487134	559655
1605	1	Q8TF05	Q8TF05	Serine/threonine-protein phosphatase 4 regulatory subunit 1	PPP4R1	Homo sapiens	107.007	14	14	1350879	2218939

1606	1	Q92556	Q92556	Engulfment and cell motility protein 1	ELMO1	Homo sapiens	83.832	17	14	2311960	7520333
1607	1	Q92576	Q92576	PHD finger protein 3	PHF3	Homo sapiens	229.484	14	14	789424	309145
1608	1	Q96AX1	Q96AX1	Vacuolar protein sorting-associated protein 33A	VPS33A	Homo sapiens	67.613	14	14	2944390	1892793
1609	1	Q96CX2	Q96CX2	BTB/POZ domain-containing protein KCTD12	KCTD12	Homo sapiens	35.702	15	14	41538764	9019760
1610	1	Q96DI7	Q96DI7	U5 small nuclear ribonucleoprotein 40 kDa protein	SNRNP40	Homo sapiens	39.31	14	14	5097348	2242129
1611	1	Q96JM3	Q96JM3	Chromosome alignment-maintaining phosphoprotein 1	CHAMP1	Homo sapiens	89.102	14	14	2491761	989800
1612	1	Q96LD4	Q96LD4	E3 ubiquitin-protein ligase TRIM47	TRIM47	Homo sapiens	69.531	14	14	4125958	331083
1613	1	Q96MM6	Q96MM6	Heat shock 70 kDa protein 12B	HSPA12B	Homo sapiens	75.689	14	14	3423845	0
1614	1	Q96RQ9	Q96RQ9	L-amino-acid oxidase	IL4I1	Homo sapiens	62.882	14	14	1073891	3141481
1615	1	Q96RS6	Q96RS6	NudC domain-containing protein 1	NUDCD1	Homo sapiens	66.755	14	14	2862712	1952182
1616	1	Q96SB3	Q96SB3	Neurabin-2	PPP1R9B	Homo sapiens	89.336	15	14	1420998	1014140
1617	1	Q96T76	Q96T76	MMS19 nucleotide excision repair protein homolog	MMS19	Homo sapiens	113.292	14	14	1748665	628934
1618	1	Q99447	Q99447	Ethanolamine-phosphate cytidylyltransferase	PCYT2	Homo sapiens	43.833	14	14	4100121	1515125
1619	1	Q99541	Q99541	Perilipin-2	PLIN2	Homo sapiens	48.076	14	14	624688	7425438
1620	1	Q9BRR9	Q9BRR9	Rho GTPase-activating protein 9	ARHGAP9	Homo sapiens	83.26	16	14	36362	4159795
1621	1	Q9BSW2	Q9BSW2	EF-hand calcium-binding domain-containing protein 4B	CRACR2A	Homo sapiens	83.193	14	14	1174319	1531926
1622	1	Q9BV36	Q9BV36	Melanophilin	MLPH	Homo sapiens	65.949	14	14	1564136	12990
1623	1	Q9BXP5	Q9BXP5	Serrate RNA effector molecule homolog	SRRT	Homo sapiens	100.669	14	14	6589579	3896646
1624	1	Q9BYK8	Q9BYK8	Helicase with zinc finger domain 2	HELZ2	Homo sapiens	294.653	14	14	506229	714238
1625	1	Q9BZF1	Q9BZF1	Oxysterol-binding protein-related protein 8	OSBPL8	Homo sapiens	101.197	16	14	5493415	4787058
1626	1	Q9H269	Q9H269	Vacuolar protein sorting-associated protein 16 homolog	VPS16	Homo sapiens	94.691	14	14	4446755	5700514
1627	1	Q9H3P7	Q9H3P7	Golgi resident protein GCP60	ACBD3	Homo sapiens	60.594	14	14	34403293	6183406
1628	1	Q9H4E7	Q9H4E7	Differentially expressed in FDCP 6 homolog	DEF6	Homo sapiens	73.911	15	14	868891	6438913
1629	1	Q9H5N1	Q9H5N1	Rab GTPase-binding effector protein 2	RABEP2	Homo sapiens	63.542	14	14	2450826	743743
1630	1	Q9H6S0	Q9H6S0	3'-5' RNA helicase YTHDC2	YTHDC2	Homo sapiens	160.251	14	14	1396430	744229
1631	1	Q9H9C1	Q9H9C1	Spermatogenesis-defective protein 39 homolog	VIPAS39	Homo sapiens	57.006	14	14	2174589	880330
1632	1	Q9HCG8	Q9HCG8	Pre-mRNA-splicing factor CWC22 homolog	CWC22	Homo sapiens	105.468	14	14	688619	148769
1633	1	Q9NR50	Q9NR50	Translation initiation factor eIF-2B subunit gamma	EIF2B3	Homo sapiens	50.241	14	14	3608975	2426759
1634	1	Q9NTK5	Q9NTK5	Obg-like ATPase 1	OLA1	Homo sapiens	44.741	14	14	21095885	8478503
1635	1	Q9NVH1	Q9NVH1	DnaJ homolog subfamily C member 11	DNAJC11	Homo sapiens	63.276	14	14	4329213	662209
1636	1	Q9NWZ3	Q9NWZ3	Interleukin-1 receptor-associated kinase 4	IRAK4	Homo sapiens	51.529	14	14	1467913	2772845
1637	1	Q9NZL9	Q9NZL9	Methionine adenosyltransferase 2 subunit beta	MAT2B	Homo sapiens	37.551	14	14	8214502	4372222
1638	1	Q9P0J1	Q9P0J1	[Pyruvate dehydrogenase [acetyl-transferring]]-phosphatase 1, mitochondrial	PDP1	Homo sapiens	61.054	14	14	4681384	650571
1639	1	Q9P258	Q9P258	Protein RCC2	RCC2	Homo sapiens	56.085	14	14	5598520	4110452
1640	1	Q9P2I0	Q9P2I0	Cleavage and polyadenylation specificity factor subunit 2	CPSF2	Homo sapiens	88.487	14	14	1682087	772838
1641	1	Q9UBB6	Q9UBB6	Neurochondrin	NCDN	Homo sapiens	78.864	14	14	1344964	277816
1642	1	Q9UI08	Q9UI08	Ena/VASP-like protein	EVL	Homo sapiens	44.621	14	14	4686124	954408
1643	1	Q9UJS0	Q9UJS0	Electrogenic aspartate/glutamate antiporter SLC25A13, mitochondrial	SLC25A13	Homo sapiens	74.175	20	14	10067818	4926235
1644	1	Q9UJW0	Q9UJW0	Dynactin subunit 4	DCTN4	Homo sapiens	52.337	14	14	5293338	1503991
1645	1	Q9UK61	Q9UK61	Protein TASOR	TASOR	Homo sapiens	189.034	14	14	1502377	351759
1646	1	Q9UKE5	Q9UKE5	TRAF2 and NCK-interacting protein kinase	TNIK	Homo sapiens	154.947	14	14	1244014	70225
1647	1	Q9Y2K7	Q9Y2K7	Lysine-specific demethylase 2A	KDM2A	Homo sapiens	132.795	14	14	1058885	866394
1648	1	Q9Y2U8	Q9Y2U8	Inner nuclear membrane protein Man1	LEMD3	Homo sapiens	99.998	14	14	3498871	1402595
1649	1	Q9Y315	Q9Y315	Deoxyribose-phosphate aldolase	DERA	Homo sapiens	35.231	14	14	3129409	1985540
1650	1	Q9Y5B6	Q9Y5B6	PAX3- and PAX7-binding protein 1	PAXBP1	Homo sapiens	104.806	14	14	1374893	228143
1651	1	Q9Y5L0	Q9Y5L0	Transportin-3	TNPO3	Homo sapiens	104.205	14	14	1705378	601500
1652	1	Q9Y606	Q9Y606	Pseudouridylate synthase 1 homolog	PUS1	Homo sapiens	47.471	14	14	2626480	2771182

1653	1	Q9Y6K5	Q9Y6K5	2'-5'-oligoadenylate synthase 3	OAS3	Homo sapiens	121.172	14	14	2134559	3147539
1654	1	O00139	O00139	Kinesin-like protein KIF2A	KIF2A	Homo sapiens	79.955	15	13	1980918	2664318
1655	1	O00151	O00151	PDZ and LIM domain protein 1	PDLIM1	Homo sapiens	36.07	13	13	45038026	5095912
1656	1	O00462	O00462	Beta-mannosidase	MANBA	Homo sapiens	100.897	13	13	1621516	1307710
1657	1	O00764	O00764	Pyridoxal kinase	PDXK	Homo sapiens	35.102	13	13	6826397	12114180
1658	1	O14744	O14744	Protein arginine N-methyltransferase 5	PRMT5	Homo sapiens	72.684	13	13	3560317	1652174
1659	1	O14802	O14802	DNA-directed RNA polymerase III subunit RPC1	POLR3A	Homo sapiens	155.645	13	13	741515	287792
1660	1	O15264	O15264	Mitogen-activated protein kinase 13	MAPK13	Homo sapiens	42.089	13	13	5459187	465506
1661	1	O15327	O15327	Inositol polyphosphate 4-phosphatase type II	INPP4B	Homo sapiens	104.738	13	13	1682672	4846182
1662	1	O43490	O43490	Prominin-1	PROM1	Homo sapiens	97.203	13	13	45723	2416491
1663	1	O43660	O43660	Pleiotropic regulator 1	PLRG1	Homo sapiens	57.195	13	13	77312753	245187
1664	1	O43704	O43704	Sulfotransferase 1B1	SULT1B1	Homo sapiens	34.899	13	13	22661587	4949943
1665	1	O60216	O60216	Double-strand-break repair protein rad21 homolog	RAD21	Homo sapiens	71.693	15	13	2717330	1198556
1666	1	O60245	O60245	Protocadherin-7	PCDH7	Homo sapiens	116.071	13	13	1411811	0
1667	1	O60292	O60292	Signal-induced proliferation-associated 1-like protein 3	SIPA1L3	Homo sapiens	194.611	13	13	1125202	81429
1668	1	O60524	O60524	Ribosome quality control complex subunit NEMF	NEMF	Homo sapiens	122.955	13	13	3387987	1392071
1669	1	O60603	O60603	Toll-like receptor 2	TLR2	Homo sapiens	89.836	13	13	360958	3135967
1670	1	O75051	O75051	Plexin-A2	PLXNA2	Homo sapiens	211.106	13	13	642436	0
1671	1	O75110	O75110	Probable phospholipid-transporting ATPase IIA	ATP9A	Homo sapiens	118.583	14	13	3960896	1907455
1672	1	O75128	O75128	Protein cordon-bleu	COBL	Homo sapiens	135.618	13	13	2173952	0
1673	1	O75152	O75152	Zinc finger CCCH domain-containing protein 11A	ZC3H11A	Homo sapiens	89.131	19	13	1862554	1175095
1674	1	O75179	O75179	Ankyrin repeat domain-containing protein 17	ANKRD17	Homo sapiens	274.262	21	13	3937430	211893
1675	1	O75363	O75363	Breast carcinoma-amplified sequence 1	BCAS1	Homo sapiens	61.71	13	13	10040156	140614
1676	1	O75382	O75382	Tripartite motif-containing protein 3	TRIM3	Homo sapiens	80.828	13	13	4455323	89914
1677	1	O75400	O75400	Pre-mRNA-processing factor 40 homolog A	PRPF40A	Homo sapiens	108.806	14	13	2716336	2524489
1678	1	O75475	O75475	PC4 and SFRS1-interacting protein	PSIP1	Homo sapiens	60.104	13	13	7004850	803055
1679	1	O75563	O75563	Src kinase-associated phosphoprotein 2	SKAP2	Homo sapiens	41.218	13	13	426317	11267265
1680	1	O75691	O75691	Small subunit processome component 20 homolog	UTP20	Homo sapiens	318.389	13	13	941763	142005
1681	1	O76024	O76024	Wolframin	WFS1	Homo sapiens	100.293	13	13	2768406	22710
1682	1	O94813	O94813	Slit homolog 2 protein	SLIT2	Homo sapiens	169.874	14	13	866850	0
1683	1	O94855	O94855	Protein transport protein Sec24D	SEC24D	Homo sapiens	113.01	13	13	4137812	1060181
1684	1	O94856	O94856	Neurofascin	NFASC	Homo sapiens	150.031	13	13	2402482	0
1685	1	O94925	O94925	Glutaminase kidney isoform, mitochondrial	GLS	Homo sapiens	73.46	13	13	5294392	649885
1686	1	O95433	O95433	Activator of 90 kDa heat shock protein ATPase homolog 1	AHSA1	Homo sapiens	38.273	13	13	11091981	4929471
1687	1	O95819	O95819	Mitogen-activated protein kinase kinase kinase kinase 4	MAP4K4	Homo sapiens	142.103	20	13	1153775	1849617
1688	1	P00505	P00505	Aspartate aminotransferase, mitochondrial	GOT2	Homo sapiens	47.519	13	13	41932638	7113242
1689	1	P00533	P00533	Epidermal growth factor receptor	EGFR	Homo sapiens	134.277	13	13	2592142	64994
1690	1	P00558	P00558	Phosphoglycerate kinase 1	PGK1	Homo sapiens	44.616	15	13	180013930	169085280
1691	1	P00568	P00568	Adenylate kinase isoenzyme 1	AK1	Homo sapiens	21.633	14	13	24981298	6202017
1692	1	P00740	P00740	Coagulation factor IX	F9	Homo sapiens	51.78	14	13	4439764	32989835
1693	1	P02461	P02461	Collagen alpha-1(III) chain	COL3A1	Homo sapiens	138.566	14	13	22157782	7246177
1694	1	P04066	P04066	Tissue alpha-L-fucosidase	FUCA1	Homo sapiens	53.689	13	13	23041013	1610639
1695	1	P04196	P04196	Histidine-rich glycoprotein	HRG	Homo sapiens	59.578	14	13	6065621	18709231
1696	1	P05160	P05160	Coagulation factor XIII B chain	F13B	Homo sapiens	75.51	14	13	1482989	13740748
1697	1	P05186	P05186	Alkaline phosphatase, tissue-nonspecific isozyme	ALPL	Homo sapiens	57.305	13	13	226710	4415983
1698	1	P05362	P05362	Intercellular adhesion molecule 1	ICAM1	Homo sapiens	57.825	13	13	1688276	10958759
1699	1	P06213	P06213	Insulin receptor	INSR	Homo sapiens	156.336	15	13	1382538	266603
1700	1	P07339	P07339	Cathepsin D	CTSD	Homo sapiens	44.551	13	13	54844583	51212438

1701	1 P08243	P08243	Asparagine synthetase [glutamine-hydrolyzing]	ASNS	Homo sapiens	64.371	13	13	2513013	1204384
1702	1 P08582	P08582	Melanotransferrin	MELTF	Homo sapiens	80.215	13	13	425552	1026251
1703	1 P13521	P13521	Secretogranin-2	SCG2	Homo sapiens	70.941	13	13	3848491	0
1704	1 P13637	P13637	Sodium/potassium-transporting ATPase subunit alpha-3	ATP1A3	Homo sapiens	111.75	15	13	2396342	63750
1705	1 P14314	P14314	Glucosidase 2 subunit beta	PRKCSH	Homo sapiens	59.428	13	13	38503889	21081373
1706	1 P14317	P14317	Hematopoietic lineage cell-specific protein	HCLS1	Homo sapiens	54.014	13	13	5695735	8831690
1707	1 P15586	P15586	N-acetylglucosamine-6-sulfatase	GNS	Homo sapiens	62.083	13	13	3579359	7849286
1708	1 P17844	P17844	Probable ATP-dependent RNA helicase DDX5	DDX5	Homo sapiens	69.148	13	13	8143869	4707699
1709	1 P18084	P18084	Integrin beta-5	ITGB5	Homo sapiens	88.056	13	13	1651785	137184
1710	1 P19174	P19174	1-phosphatidylinositol 4,5-bisphosphate phosphodiesterase gamma-1	PLCG1	Homo sapiens	148.534	13	13	2055589	102110
1711	1 P19474	P19474	E3 ubiquitin-protein ligase TRIM21	TRIM21	Homo sapiens	54.171	13	13	3567540	4355535
1712	1 P21266	P21266	Glutathione S-transferase Mu 3	GSTM3	Homo sapiens	26.559	13	13	18439476	1073823
1713	1 P21589	P21589	5'-nucleotidase	NT5E	Homo sapiens	63.365	13	13	10103064	2917713
1714	1 P21796	P21796	Voltage-dependent anion-selective channel protein 1	VDAC1	Homo sapiens	30.773	13	13	32481369	20435423
1715	1 P22570	P22570	NADPH:adrenodoxin oxidoreductase, mitochondrial	FDXR	Homo sapiens	53.836	13	13	4048940	2361401
1716	1 P23142	P23142	Fibulin-1	FBLN1	Homo sapiens	77.216	21	13	23565087	9631417
1717	1 P23470	P23470	Receptor-type tyrosine-protein phosphatase gamma	PTPRG	Homo sapiens	162.006	13	13	2123804	114071
1718	1 P25054	P25054	Adenomatous polyposis coli protein	APC	Homo sapiens	311.649	13	13	465609	0
1719	1 P26641	P26641	Elongation factor 1-gamma	EEF1G	Homo sapiens	50.12	13	13	36305601	25294413
1720	1 P27105	P27105	Stomatin	STOM	Homo sapiens	31.73	13	13	23936195	125671671
1721	1 P27695	P27695	DNA-(apurinic or apyrimidinic site) endonuclease	APEX1	Homo sapiens	35.555	13	13	18187063	16783404
1722	1 P28289	P28289	Tropomodulin-1	TMOD1	Homo sapiens	40.57	14	13	8537402	1054056
1723	1 P29323	P29323	Ephrin type-B receptor 2	EPHB2	Homo sapiens	117.492	17	13	2045210	28523
1724	1 P30085	P30085	UMP-CMP kinase	CMPK1	Homo sapiens	22.222	13	13	14861471	10206237
1725	1 P30566	P30566	Adenylosuccinate lyase	ADSL	Homo sapiens	54.89	13	13	5083593	2741223
1726	1 P32456	P32456	Guanylate-binding protein 2	GBP2	Homo sapiens	67.209	17	13	8660878	3012279
1727	1 P35237	P35237	Serpin B6	SERPINB6	Homo sapiens	42.623	13	13	44259491	11609045
1728	1 P35251	P35251	Replication factor C subunit 1	RFC1	Homo sapiens	128.257	13	13	800039	67239
1729	1 P35270	P35270	Sepiapterin reductase	SPR	Homo sapiens	28.047	13	13	10984915	437784
1730	1 P39059	P39059	Collagen alpha-1(XV) chain	COL15A1	Homo sapiens	141.721	13	13	21762263	3656909
1731	1 P40123	P40123	Adenylyl cyclase-associated protein 2	CAP2	Homo sapiens	52.824	13	13	14221407	1037423
1732	1 P40222	P40222	Alpha-taxilin	TXLNA	Homo sapiens	61.891	13	13	2660087	1350356
1733	1 P40879	P40879	Chloride anion exchanger	SLC26A3	Homo sapiens	84.504	13	13	6376253	64747
1734	1 P43246	P43246	DNA mismatch repair protein Msh2	MSH2	Homo sapiens	104.745	13	13	1626271	746925
1735	1 P43378	P43378	Tyrosine-protein phosphatase non-receptor type 9	PTPN9	Homo sapiens	68.021	13	13	1497314	476961
1736	1 P43652	P43652	Afamin	AFM	Homo sapiens	69.072	13	13	9525411	17485964
1737	1 P46108	P46108	Adapter molecule crk	CRK	Homo sapiens	33.83	13	13	9128739	1546904
1738	1 P46109	P46109	Crk-like protein	CRKL	Homo sapiens	33.777	13	13	8352344	3625626
1739	1 P49257	P49257	Protein ERGIC-53	LMAN1	Homo sapiens	57.547	13	13	8086923	9183619
1740	1 P49770	P49770	Translation initiation factor eIF-2B subunit beta	EIF2B2	Homo sapiens	38.988	13	13	1930744	712946
1741	1 P49821	P49821	NADH dehydrogenase [ubiquinone] flavoprotein 1, mitochondrial	NDUFV1	Homo sapiens	50.818	13	13	16819534	2774503
1742	1 P50748	P50748	Kinetochore-associated protein 1	KNTC1	Homo sapiens	250.751	13	13	236441	606026
1743	1 P50993	P50993	Sodium/potassium-transporting ATPase subunit alpha-2	ATP1A2	Homo sapiens	112.265	13	13	3816805	0
1744	1 P51665	P51665	26S proteasome non-ATPase regulatory subunit 7	PSMD7	Homo sapiens	37.025	13	13	6637585	4420127
1745	1 P51688	P51688	N-sulphoglucosamine sulphohydrolase	SGSH	Homo sapiens	56.697	13	13	5350717	8130093
1746	1 P52209	P52209	6-phosphogluconate dehydrogenase, decarboxylating	PGD	Homo sapiens	53.139	13	13	31138747	146786709
1747	1 P54619	P54619	5'-AMP-activated protein kinase subunit gamma-1	PRKAG1	Homo sapiens	37.579	14	13	8489767	3651561
1748	1 P55157	P55157	Microsomal triglyceride transfer protein large subunit	MTTP	Homo sapiens	99.353	13	13	1963197	115484

1749	1	P57772	P57772	Selenocysteine-specific elongation factor	EEFSEC	Homo sapiens	65.306	13	13	3724849	1519939
1750	1	P60842	P60842	Eukaryotic initiation factor 4A-I	EIF4A1	Homo sapiens	46.156	23	13	23968445	22168529
1751	1	P61201	P61201	COP9 signalosome complex subunit 2	COPS2	Homo sapiens	51.597	13	13	6648760	1908511
1752	1	P62258	P62258	14-3-3 protein epsilon	YWHAE	Homo sapiens	29.172	13	13	99681534	28813460
1753	1	P83111	P83111	Serine beta-lactamase-like protein LACTB, mitochondrial	LACTB	Homo sapiens	60.692	13	13	3282728	4358961
1754	1	P98082	P98082	Disabled homolog 2	DAB2	Homo sapiens	82.449	13	13	3337577	423935
1755	1	Q00325	Q00325	Phosphate carrier protein, mitochondrial	SLC25A3	Homo sapiens	40.095	13	13	37931652	23334035
1756	1	Q01432	Q01432	AMP deaminase 3	AMPD3	Homo sapiens	88.81	16	13	708054	4224980
1757	1	Q04864	Q04864	Proto-oncogene c-Rel	REL	Homo sapiens	68.522	13	13	1658762	13401044
1758	1	Q05655	Q05655	Protein kinase C delta type	PRKCD	Homo sapiens	77.506	14	13	2771780	16328906
1759	1	Q06323	Q06323	Proteasome activator complex subunit 1	PSME1	Homo sapiens	28.72	13	13	41296127	52198970
1760	1	Q08174	Q08174	Protocadherin-1	PCDH1	Homo sapiens	114.746	13	13	4206862	325353
1761	1	Q08257	Q08257	Quinone oxidoreductase	CRYZ	Homo sapiens	35.205	13	13	18360570	3235888
1762	1	Q10471	Q10471	Polypeptide N-acetylgalactosaminyltransferase 2	GALNT2	Homo sapiens	64.732	14	13	3408001	3564231
1763	1	Q12792	Q12792	Twinfilin-1	TWF1	Homo sapiens	40.283	13	13	7086183	3955642
1764	1	Q12874	Q12874	Splicing factor 3A subunit 3	SF3A3	Homo sapiens	58.849	13	13	6234378	918213
1765	1	Q13043	Q13043	Serine/threonine-protein kinase 4	STK4	Homo sapiens	55.63	19	13	1028326	7839104
1766	1	Q13075	Q13075	Baculoviral IAP repeat-containing protein 1	NAIP	Homo sapiens	159.583	13	13	736926	2352529
1767	1	Q13287	Q13287	N-myc-interactor	NMI	Homo sapiens	35.057	13	13	4670658	6422937
1768	1	Q13409	Q13409	Cytoplasmic dynein 1 intermediate chain 2	DYNC1I2	Homo sapiens	71.459	13	13	12450269	2613086
1769	1	Q13421	Q13421	Mesothelin	MSLN	Homo sapiens	68.984	13	13	0	2708735
1770	1	Q13443	Q13443	Disintegrin and metalloproteinase domain-containing protein 9	ADAM9	Homo sapiens	90.559	13	13	1036812	1097236
1771	1	Q13615	Q13615	Myotubularin-related protein 3	MTMR3	Homo sapiens	133.62	13	13	393412	1329212
1772	1	Q13642	Q13642	Four and a half LIM domains protein 1	FHL1	Homo sapiens	36.262	13	13	265906233	1184531
1773	1	Q13797	Q13797	Integrin alpha-9	ITGA9	Homo sapiens	114.49	13	13	1445697	285925
1774	1	Q14558	Q14558	Phosphoribosyl pyrophosphate synthase-associated protein 1	PRPSAP1	Homo sapiens	39.394	14	13	4062413	1695581
1775	1	Q14694	Q14694	Ubiquitin carboxyl-terminal hydrolase 10	USP10	Homo sapiens	87.137	13	13	5269156	1147245
1776	1	Q15063	Q15063	Periostin	POSTN	Homo sapiens	93.317	30	13	13293821	18284088
1777	1	Q15113	Q15113	Procollagen C-endopeptidase enhancer 1	PCOLCE	Homo sapiens	47.972	13	13	4313470	8859443
1778	1	Q15181	Q15181	Inorganic pyrophosphatase	PPA1	Homo sapiens	32.661	13	13	28540363	11249949
1779	1	Q15637	Q15637	Splicing factor 1	SF1	Homo sapiens	68.332	13	13	3622148	768654
1780	1	Q16222	Q16222	UDP-N-acetylhexosamine pyrophosphorylase	UAP1	Homo sapiens	58.769	14	13	6232686	812033
1781	1	Q16853	Q16853	Membrane primary amine oxidase	AOC3	Homo sapiens	84.621	16	13	162745265	356497
1782	1	Q49A26	Q49A26	Cytokine-like nuclear factor N-PAC	GLYR1	Homo sapiens	60.546	13	13	5617924	3123047
1783	1	Q4G0N4	Q4G0N4	NAD kinase 2, mitochondrial	NADK2	Homo sapiens	49.432	13	13	4352282	1095043
1784	1	Q4G176	Q4G176	Malonate--CoA ligase ACSF3, mitochondrial	ACSF3	Homo sapiens	64.131	13	13	2483371	236030
1785	1	Q53GS9	Q53GS9	U4/U6.U5 tri-snRNP-associated protein 2	USP39	Homo sapiens	65.379	13	13	2808292	1631018
1786	1	Q53H12	Q53H12	Acylglycerol kinase, mitochondrial	AGK	Homo sapiens	47.136	13	13	5172866	2501902
1787	1	Q5QJE6	Q5QJE6	Deoxynucleotidyltransferase terminal-interacting protein 2	DNTTIP2	Homo sapiens	84.469	13	13	2399932	842546
1788	1	Q5TEJ8	Q5TEJ8	Protein THEMIS2	THEMIS2	Homo sapiens	72.051	13	13	243471	2220773
1789	1	Q5VU43	Q5VU43	Myomegalin	PDE4DIP	Homo sapiens	265.106	13	13	3398983	321780
1790	1	Q5VZE5	Q5VZE5	N-alpha-acetyltransferase 35, NatC auxiliary subunit	NAA35	Homo sapiens	83.64	13	13	1404185	247095
1791	1	Q6NVY1	Q6NVY1	3-hydroxyisobutyryl-CoA hydrolase, mitochondrial	HIBCH	Homo sapiens	43.484	13	13	7874707	1366429
1792	1	Q6PD62	Q6PD62	RNA polymerase-associated protein CTR9 homolog	CTR9	Homo sapiens	133.503	13	13	1643790	592803
1793	1	Q6UX06	Q6UX06	Olfactomedin-4	OLFM4	Homo sapiens	57.281	13	13	2270330	86765606
1794	1	Q6V0I7	Q6V0I7	Protocadherin Fat 4	FAT4	Homo sapiens	542.694	13	13	936719	31382
1795	1	Q6ZUJ8	Q6ZUJ8	Phosphoinositide 3-kinase adapter protein 1	PIK3AP1	Homo sapiens	90.4	13	13	305463	6240793
1796	1	Q70CQ2	Q70CQ2	Ubiquitin carboxyl-terminal hydrolase 34	USP34	Homo sapiens	404.238	13	13	381867	90540

1797	1	Q7L591	Q7L591	Docking protein 3	DOK3	Homo sapiens	53.29	13	13	294732	9934429
1798	1	Q7L775	Q7L775	EPM2A-interacting protein 1	EPM2AIP1	Homo sapiens	70.37	13	13	2953816	104162
1799	1	Q7Z5K2	Q7Z5K2	Wings apart-like protein homolog	WAPL	Homo sapiens	132.946	13	13	1345727	1305891
1800	1	Q8IWA4	Q8IWA4	Mitofusin-1	MFN1	Homo sapiens	84.161	13	13	1688945	398963
1801	1	Q8N1F8	Q8N1F8	Serine/threonine-protein kinase 11-interacting protein	STK11IP	Homo sapiens	120.261	13	13	771446	620605
1802	1	Q8N7H5	Q8N7H5	RNA polymerase II-associated factor 1 homolog	PAF1	Homo sapiens	59.975	13	13	1489749	826475
1803	1	Q8NB90	Q8NB90	Ribosome biogenesis protein SPATA5	SPATA5	Homo sapiens	97.907	13	13	1761231	628062
1804	1	Q8ND24	Q8ND24	RING finger protein 214	RNF214	Homo sapiens	77.669	13	13	1911447	185154
1805	1	Q8NFD5	Q8NFD5	AT-rich interactive domain-containing protein 1B	ARID1B	Homo sapiens	243.945	13	13	1658252	114348
1806	1	Q8NI60	Q8NI60	Atypical kinase COQ8A, mitochondrial	COQ8A	Homo sapiens	71.949	13	13	2435786	161469
1807	1	Q8TAX9	Q8TAX9	Gasdermin-B	GSDMB	Homo sapiens	47.348	13	13	2996079	540056
1808	1	Q8TE67	Q8TE67	Epidermal growth factor receptor kinase substrate 8-like protein 3	EPS8L3	Homo sapiens	66.862	13	13	3599236	0
1809	1	Q8WUP2	Q8WUP2	Filamin-binding LIM protein 1	FBLIM1	Homo sapiens	40.668	13	13	2588576	312947
1810	1	Q8WVT3	Q8WVT3	Trafficking protein particle complex subunit 12	TRAPPC12	Homo sapiens	79.375	13	13	1174408	1218729
1811	1	Q8WWI5	Q8WWI5	Choline transporter-like protein 1	SLC44A1	Homo sapiens	73.303	13	13	6718318	1417916
1812	1	Q96AG4	Q96AG4	Leucine-rich repeat-containing protein 59	LRRC59	Homo sapiens	34.931	13	13	23380685	6958584
1813	1	Q96BP3	Q96BP3	Peptidylprolyl isomerase domain and WD repeat-containing protein 1	PPWD1	Homo sapiens	73.573	13	13	1076933	643479
1814	1	Q96CW1	Q96CW1	AP-2 complex subunit mu	AP2M1	Homo sapiens	49.657	13	13	9826056	4005971
1815	1	Q96EE3	Q96EE3	Nucleoporin SEH1	SEH1L	Homo sapiens	39.649	13	13	2578327	1927238
1816	1	Q96EY7	Q96EY7	Pentatricopeptide repeat domain-containing protein 3, mitochondrial	PTCD3	Homo sapiens	78.551	13	13	2742590	257967
1817	1	Q96FN4	Q96FN4	Copine-2	CPNE2	Homo sapiens	61.19	14	13	2146721	4104022
1818	1	Q96JP2	Q96JP2	Unconventional myosin-XVB	MYO15B	Homo sapiens	167.091	13	13	718831	0
1819	1	Q96PU5	Q96PU5	E3 ubiquitin-protein ligase NEDD4-like	NEDD4L	Homo sapiens	111.933	13	13	2340094	105059
1820	1	Q96PY5	Q96PY5	Formin-like protein 2	FMNL2	Homo sapiens	123.322	16	13	848782	464666
1821	1	Q96SB4	Q96SB4	SRSF protein kinase 1	SRPK1	Homo sapiens	74.327	14	13	1560104	1462813
1822	1	Q96SU4	Q96SU4	Oxysterol-binding protein-related protein 9	OSBPL9	Homo sapiens	83.188	13	13	1292698	1063030
1823	1	Q99700	Q99700	Ataxin-2	ATXN2	Homo sapiens	140.285	13	13	2114225	1102680
1824	1	Q99797	Q99797	Mitochondrial intermediate peptidase	MIPEP	Homo sapiens	80.64	13	13	1540967	192476
1825	1	Q99829	Q99829	Copine-1	CPNE1	Homo sapiens	59.059	13	13	8740063	9730928
1826	1	Q99985	Q99985	Semaphorin-3C	SEMA3C	Homo sapiens	85.205	13	13	616492	758432
1827	1	Q9BQ39	Q9BQ39	ATP-dependent RNA helicase DDX50	DDX50	Homo sapiens	82.563	13	13	1540845	318525
1828	1	Q9BVP2	Q9BVP2	Guanine nucleotide-binding protein-like 3	GNL3	Homo sapiens	61.993	13	13	2938754	1038931
1829	1	Q9BW27	Q9BW27	Nuclear pore complex protein Nup85	NUP85	Homo sapiens	75.019	13	13	1887432	1538090
1830	1	Q9BW92	Q9BW92	Threonine--tRNA ligase, mitochondrial	TARS2	Homo sapiens	81.039	13	13	3030609	785002
1831	1	Q9BWH6	Q9BWH6	RNA polymerase II-associated protein 1	RPAP1	Homo sapiens	152.757	13	13	824087	97870
1832	1	Q9BXB5	Q9BXB5	Oxysterol-binding protein-related protein 10	OSBPL10	Homo sapiens	83.97	13	13	1145649	160267
1833	1	Q9BXN1	Q9BXN1	Asporin	ASPN	Homo sapiens	43.419	13	13	26965262	322214
1834	1	Q9BZL6	Q9BZL6	Serine/threonine-protein kinase D2	PRKD2	Homo sapiens	96.723	15	13	942028	1419400
1835	1	Q9BZQ6	Q9BZQ6	ER degradation-enhancing alpha-mannosidase-like protein 3	EDEM3	Homo sapiens	104.666	13	13	1358884	187338
1836	1	Q9C0I1	Q9C0I1	Myotubularin-related protein 12	MTMR12	Homo sapiens	86.148	13	13	1310943	49387
1837	1	Q9H0B6	Q9H0B6	Kinesin light chain 2	KLC2	Homo sapiens	68.935	13	13	1770793	292113
1838	1	Q9H1K0	Q9H1K0	Rabenosyn-5	RBSN	Homo sapiens	88.87	13	13	1654101	103465
1839	1	Q9H6T3	Q9H6T3	RNA polymerase II-associated protein 3	RPAP3	Homo sapiens	75.72	13	13	1598766	1415168
1840	1	Q9H8L6	Q9H8L6	Multimerin-2	MMRN2	Homo sapiens	104.41	13	13	1657694	1258234
1841	1	Q9H9A5	Q9H9A5	CCR4-NOT transcription complex subunit 10	CNOT10	Homo sapiens	82.309	13	13	1219412	1324213
1842	1	Q9H9E3	Q9H9E3	Conserved oligomeric Golgi complex subunit 4	COG4	Homo sapiens	89.083	13	13	2689034	1355029
1843	1	Q9HA77	Q9HA77	Probable cysteine--tRNA ligase, mitochondrial	CARS2	Homo sapiens	62.224	13	13	2616640	1075775
1844	1	Q9HAV4	Q9HAV4	Exportin-5	XPO5	Homo sapiens	136.313	13	13	1176850	571684

1845	1	Q9HCS7	Q9HCS7	Pre-mRNA-splicing factor SYF1	XAB2	Homo sapiens	100.011	13	13	1162165	362175
1846	1	Q9NQR4	Q9NQR4	Omega-amidase NIT2	NIT2	Homo sapiens	30.609	13	13	8477442	5448938
1847	1	Q9NR16	Q9NR16	Scavenger receptor cysteine-rich type 1 protein M160	CD163L1	Homo sapiens	159.242	13	13	2279100	0
1848	1	Q9NRV9	Q9NRV9	Heme-binding protein 1	HEBP1	Homo sapiens	21.097	13	13	12676427	5472646
1849	1	Q9NUQ6	Q9NUQ6	SPATS2-like protein	SPATS2L	Homo sapiens	61.729	13	13	3819821	623111
1850	1	Q9NVM9	Q9NVM9	Integrator complex subunit 13	INTS13	Homo sapiens	80.227	13	13	845313	805625
1851	1	Q9NW82	Q9NW82	WD repeat-containing protein 70	WDR70	Homo sapiens	73.201	13	13	1082010	436672
1852	1	Q9NXR1	Q9NXR1	Nuclear distribution protein nudE homolog 1	NDE1	Homo sapiens	37.72	15	13	1585952	475937
1853	1	Q9NYL9	Q9NYL9	Tropomodulin-3	TMOD3	Homo sapiens	39.594	14	13	9649376	9126599
1854	1	Q9P2F8	Q9P2F8	Signal-induced proliferation-associated 1-like protein 2	SIPA1L2	Homo sapiens	190.439	14	13	537589	121560
1855	1	Q9P2R7	Q9P2R7	Succinate--CoA ligase [ADP-forming] subunit beta, mitochondrial	SUCLA2	Homo sapiens	50.316	13	13	17927400	6095484
1856	1	Q9UBP0	Q9UBP0	Spastin	SPAST	Homo sapiens	67.199	13	13	1360288	797178
1857	1	Q9UID3	Q9UID3	Vacuolar protein sorting-associated protein 51 homolog	VPS51	Homo sapiens	86.042	13	13	2199835	620431
1858	1	Q9UJX5	Q9UJX5	Anaphase-promoting complex subunit 4	ANAPC4	Homo sapiens	92.118	13	13	1010858	337588
1859	1	Q9UJZ1	Q9UJZ1	Stomatin-like protein 2, mitochondrial	STOML2	Homo sapiens	38.533	13	13	12763416	6430862
1860	1	Q9UPN9	Q9UPN9	E3 ubiquitin-protein ligase TRIM33	TRIM33	Homo sapiens	122.533	14	13	849030	640378
1861	1	Q9UPU9	Q9UPU9	Protein Smaug homolog 1	SAMD4A	Homo sapiens	79.415	13	13	1595702	0
1862	1	Q9UQ90	Q9UQ90	Paraplegin	SPG7	Homo sapiens	88.237	13	13	3018853	273441
1863	1	Q9Y285	Q9Y285	Phenylalanine--tRNA ligase alpha subunit	FARSA	Homo sapiens	57.561	13	13	5354461	1127623
1864	1	Q9Y295	Q9Y295	Developmentally-regulated GTP-binding protein 1	DRG1	Homo sapiens	40.543	13	13	4920568	1907252
1865	1	Q9Y2W2	Q9Y2W2	WW domain-binding protein 11	WBP11	Homo sapiens	69.997	13	13	3593016	697516
1866	1	Q9Y3F4	Q9Y3F4	Serine-threonine kinase receptor-associated protein	STRAP	Homo sapiens	38.438	13	13	15897940	6729402
1867	1	Q9Y4B6	Q9Y4B6	DDB1- and CUL4-associated factor 1	DCAF1	Homo sapiens	169.01	13	13	1284620	0
1868	1	Q9Y512	Q9Y512	Sorting and assembly machinery component 50 homolog	SAMM50	Homo sapiens	51.978	13	13	8975625	3969155
1869	1	Q9Y5X3	Q9Y5X3	Sorting nexin-5	SNX5	Homo sapiens	46.817	14	13	12080005	5553235
1870	1	Q9Y617	Q9Y617	Phosphoserine aminotransferase	PSAT1	Homo sapiens	40.423	13	13	2544139	9962513
1871	1	Q9Y6M1	Q9Y6M1	Insulin-like growth factor 2 mRNA-binding protein 2	IGF2BP2	Homo sapiens	66.124	14	13	10608695	1491213
1872	1	Q9Y6N9	Q9Y6N9	Harmonin	USH1C	Homo sapiens	62.211	13	13	3247999	0
1873	1	Q9Y6U3	Q9Y6U3	Scinderin	SCIN	Homo sapiens	80.49	13	13	4091700	106577
1874	1	A1L4H1	A1L4H1	Soluble scavenger receptor cysteine-rich domain-containing protein SSC5D	SSC5D	Homo sapiens	165.747	12	12	1330334	0
1875	1	O00178	O00178	GTP-binding protein 1	GTPBP1	Homo sapiens	72.457	12	12	3181195	889269
1876	1	O14579	O14579	Coatomer subunit epsilon	COPE	Homo sapiens	34.482	12	12	3939365	2071334
1877	1	O14772	O14772	Fucose-1-phosphate guanylyltransferase	FPGT	Homo sapiens	68.01	12	12	1474216	884909
1878	1	O14964	O14964	Hepatocyte growth factor-regulated tyrosine kinase substrate	HGS	Homo sapiens	86.193	12	12	4835938	2939452
1879	1	O15040	O15040	Tectonin beta-propeller repeat-containing protein 2	TECPR2	Homo sapiens	153.851	12	12	734260	770317
1880	1	O15143	O15143	Actin-related protein 2/3 complex subunit 1B	ARPC1B	Homo sapiens	40.95	13	12	19456163	49731392
1881	1	O15164	O15164	Transcription intermediary factor 1-alpha	TRIM24	Homo sapiens	116.831	13	12	906374	321525
1882	1	O15211	O15211	Ral guanine nucleotide dissociation stimulator-like 2	RGL2	Homo sapiens	83.549	12	12	863426	485608
1883	1	O15247	O15247	Chloride intracellular channel protein 2	CLIC2	Homo sapiens	28.355	12	12	3860125	432176
1884	1	O43395	O43395	U4/U6 small nuclear ribonucleoprotein Prp3	PRPF3	Homo sapiens	77.528	12	12	2825193	1176775
1885	1	O43866	O43866	CD5 antigen-like	CD5L	Homo sapiens	38.085	12	12	2534156	9580844
1886	1	O60547	O60547	GDP-mannose 4,6 dehydratase	GMDS	Homo sapiens	41.95	12	12	15646398	2324879
1887	1	O75170	O75170	Serine/threonine-protein phosphatase 6 regulatory subunit 2	PPP6R2	Homo sapiens	104.942	12	12	1363820	762537
1888	1	O75306	O75306	NADH dehydrogenase [ubiquinone] iron-sulfur protein 2, mitochondrial	NDUFS2	Homo sapiens	52.545	12	12	19061507	2106959
1889	1	O75367	O75367	Core histone macro-H2A.1	MACROH2A1	Homo sapiens	39.18	13	12	36456240	24905619
1890	1	O75970	O75970	Multiple PDZ domain protein	MPDZ	Homo sapiens	221.622	12	12	1114808	0
1891	1	O76003	O76003	Glutaredoxin-3	GLRX3	Homo sapiens	37.432	12	12	6979488	5186064

1892	1	O76031	O76031	ATP-dependent Clp protease ATP-binding subunit clpX-like, mitochondrial	CLPX	Homo sapiens	69.224	12	12	14789195	623164
1893	1	O76038	O76038	Secretagogin	SCGN	Homo sapiens	32.038	12	12	9030207	0
1894	1	O94808	O94808	Glutamine--fructose-6-phosphate aminotransferase [isomerizing] 2	GFPT2	Homo sapiens	76.931	12	12	103014	1320317
1895	1	O94913	O94913	Pre-mRNA cleavage complex 2 protein Pcf11	PCF11	Homo sapiens	173.054	12	12	501606	71500
1896	1	O95049	O95049	Tight junction protein ZO-3	TJP3	Homo sapiens	101.397	12	12	2389483	306917
1897	1	O95870	O95870	Phosphatidylserine lipase ABHD16A	ABHD16A	Homo sapiens	63.243	12	12	3880493	2369027
1898	1	P00738	P00738	Haptoglobin	HP	Homo sapiens	45.205	21	12	591770590	1358897707
1899	1	P00915	P00915	Carbonic anhydrase 1	CA1	Homo sapiens	28.87	12	12	399587670	125384320
1900	1	P00966	P00966	Argininosuccinate synthase	ASS1	Homo sapiens	46.529	12	12	17975960	7790187
1901	1	P01275	P01275	Pro-glucagon	GCG	Homo sapiens	20.908	12	12	6708940	0
1902	1	P01889	P01889	HLA class I histocompatibility antigen, B alpha chain	HLA-B	Homo sapiens	40.46	18	12	10027468	6461326
1903	1	P03951	P03951	Coagulation factor XI	F11	Homo sapiens	70.109	12	12	2172375	4627883
1904	1	P05089	P05089	Arginase-1	ARG1	Homo sapiens	34.734	12	12	327037	4922972
1905	1	P06744	P06744	Glucose-6-phosphate isomerase	GPI	Homo sapiens	63.147	16	12	42980684	35398657
1906	1	P06865	P06865	Beta-hexosaminidase subunit alpha	HEXA	Homo sapiens	60.701	12	12	9239006	4298144
1907	1	P07196	P07196	Neurofilament light polypeptide	NEFL	Homo sapiens	61.519	12	12	5483841	0
1908	1	P07225	P07225	Vitamin K-dependent protein S	PROS1	Homo sapiens	75.123	15	12	2540903	30268459
1909	1	P07360	P07360	Complement component C8 gamma chain	C8G	Homo sapiens	22.276	12	12	6127453	25981314
1910	1	P07585	P07585	Decorin	DCN	Homo sapiens	39.745	12	12	576612353	8187141
1911	1	P07996	P07996	Thrombospondin-1	THBS1	Homo sapiens	129.385	35	12	2120010	15172086
1912	1	P08236	P08236	Beta-glucuronidase	GUSB	Homo sapiens	74.731	12	12	10024052	6431131
1913	1	P08253	P08253	72 kDa type IV collagenase	MMP2	Homo sapiens	73.881	12	12	1077008	796661
1914	1	P08559	P08559	Pyruvate dehydrogenase E1 component subunit alpha, somatic form, mitochondrial	PDHA1	Homo sapiens	43.295	15	12	10526891	2648327
1915	1	P08621	P08621	U1 small nuclear ribonucleoprotein 70 kDa	SNRNP70	Homo sapiens	51.555	12	12	15905577	5804504
1916	1	P09104	P09104	Gamma-enolase	ENO2	Homo sapiens	47.271	13	12	14983406	2078604
1917	1	P09913	P09913	Interferon-induced protein with tetratricopeptide repeats 2	IFIT2	Homo sapiens	54.632	12	12	480326	2734081
1918	1	P10155	P10155	RNA-binding protein RO60	RO60	Homo sapiens	60.67	12	12	5106423	1473032
1919	1	P10515	P10515	Dihydrolipoyllysine-residue acetyltransferase component of pyruvate dehydrogenase complex, mitochondrial	DLAT	Homo sapiens	68.996	12	12	7408099	1642111
1920	1	P12821	P12821	Angiotensin-converting enzyme	ACE	Homo sapiens	149.717	12	12	907257	856016
1921	1	P13804	P13804	Electron transfer flavoprotein subunit alpha, mitochondrial	ETFA	Homo sapiens	35.081	12	12	31970141	8493300
1922	1	P15121	P15121	Aldo-keto reductase family 1 member B1	AKR1B1	Homo sapiens	35.852	12	12	21612363	7154797
1923	1	P15848	P15848	Arylsulfatase B	ARSB	Homo sapiens	59.688	12	12	2218048	1748358
1924	1	P16152	P16152	Carbonyl reductase [NADPH] 1	CBR1	Homo sapiens	30.375	14	12	30834588	9394965
1925	1	P16219	P16219	Short-chain specific acyl-CoA dehydrogenase, mitochondrial	ACADS	Homo sapiens	44.296	12	12	29573517	2715809
1926	1	P16870	P16870	Carboxypeptidase E	CPE	Homo sapiens	53.151	12	12	3319235	0
1927	1	P17600	P17600	Synapsin-1	SYN1	Homo sapiens	74.112	13	12	2337450	0
1928	1	P19338	P19338	Nucleolin	NCL	Homo sapiens	76.615	12	12	40270627	15248344
1929	1	P19525	P19525	Interferon-induced, double-stranded RNA-activated protein kinase	EIF2AK2	Homo sapiens	62.095	12	12	5208795	7176930
1930	1	P20702	P20702	Integrin alpha-X	ITGAX	Homo sapiens	127.832	13	12	387947	6410894
1931	1	P21281	P21281	V-type proton ATPase subunit B, brain isoform	ATP6V1B2	Homo sapiens	56.5	18	12	14302102	16775317
1932	1	P21964	P21964	Catechol O-methyltransferase	COMT	Homo sapiens	30.037	12	12	4499220	3717701
1933	1	P22234	P22234	Bifunctional phosphoribosylaminoimidazole carboxylase/phosphoribosylaminoimidazole succinocarboxamide synthetase	PAICS	Homo sapiens	47.077	12	12	15316011	7483718
1934	1	P22894	P22894	Neutrophil collagenase	MMP8	Homo sapiens	53.413	12	12	1903633	55395091

1935	1	P23378	P23378	Glycine dehydrogenase (decarboxylating), mitochondrial	GLDC	Homo sapiens	112.729	12	12	82485	1476764
1936	1	P23919	P23919	Thymidylate kinase	DTYMK	Homo sapiens	23.82	12	12	3783553	2353652
1937	1	P25325	P25325	3-mercaptopyruvate sulfurtransferase	MPST	Homo sapiens	33.181	12	12	16074208	4501356
1938	1	P27348	P27348	14-3-3 protein theta	YWHAQ	Homo sapiens	27.761	14	12	40536193	8518344
1939	1	P28799	P28799	Progranulin	GRN	Homo sapiens	63.544	12	12	4145810	9425183
1940	1	P29622	P29622	Kallistatin	SERPINA4	Homo sapiens	48.543	12	12	2233379	2474348
1941	1	P30038	P30038	Delta-1-pyrroline-5-carboxylate dehydrogenase, mitochondrial	ALDH4A1	Homo sapiens	61.72	12	12	3553271	3517900
1942	1	P30520	P30520	Adenylosuccinate synthetase isozyme 2	ADSS2	Homo sapiens	50.097	14	12	7811287	9517512
1943	1	P30711	P30711	Glutathione S-transferase theta-1	GSTT1	Homo sapiens	27.333	12	12	6758374	404831
1944	1	P31689	P31689	DnaJ homolog subfamily A member 1	DNAJA1	Homo sapiens	44.866	12	12	5725695	3892278
1945	1	P33241	P33241	Lymphocyte-specific protein 1	LSP1	Homo sapiens	37.192	12	12	3087972	17357222
1946	1	P35269	P35269	General transcription factor IIF subunit 1	GTF2F1	Homo sapiens	58.243	12	12	2289185	483544
1947	1	P35443	P35443	Thrombospondin-4	THBS4	Homo sapiens	105.871	23	12	12534686	15749
1948	1	P35612	P35612	Beta-adducin	ADD2	Homo sapiens	80.856	12	12	2349367	2160676
1949	1	P35813	P35813	Protein phosphatase 1A	PPM1A	Homo sapiens	42.449	15	12	2702017	1611665
1950	1	P36551	P36551	Oxygen-dependent coproporphyrinogen-III oxidase, mitochondrial	CPOX	Homo sapiens	50.153	12	12	3182335	1914495
1951	1	P36915	P36915	Guanine nucleotide-binding protein-like 1	GNL1	Homo sapiens	68.661	12	12	2859967	1325128
1952	1	P37837	P37837	Transaldolase	TALDO1	Homo sapiens	37.54	12	12	40276423	75708175
1953	1	P41214	P41214	Eukaryotic translation initiation factor 2D	EIF2D	Homo sapiens	64.707	12	12	1268805	295516
1954	1	P42658	P42658	Dipeptidyl aminopeptidase-like protein 6	DPP6	Homo sapiens	97.589	14	12	3715486	0
1955	1	P42765	P42765	3-ketoacyl-CoA thiolase, mitochondrial	ACAA2	Homo sapiens	41.923	12	12	102468711	4827690
1956	1	P48595	P48595	Serpin B10	SERPINB10	Homo sapiens	45.403	12	12	1095746	25435224
1957	1	P48739	P48739	Phosphatidylinositol transfer protein beta isoform	PITPNB	Homo sapiens	31.542	13	12	9217348	2575163
1958	1	P48960	P48960	Adhesion G protein-coupled receptor E5	ADGRE5	Homo sapiens	91.868	12	12	3180014	4275498
1959	1	P49593	P49593	Protein phosphatase 1F	PPM1F	Homo sapiens	49.83	12	12	3175041	2090855
1960	1	P50213	P50213	Isocitrate dehydrogenase [NAD] subunit alpha, mitochondrial	IDH3A	Homo sapiens	39.59	12	12	23560895	5238006
1961	1	P51531	P51531	Probable global transcription activator SNF2L2	SMARCA2	Homo sapiens	181.283	27	12	2416751	797946
1962	1	P51570	P51570	Galactokinase	GALK1	Homo sapiens	42.273	12	12	6254405	3679458
1963	1	P53367	P53367	Arfaptin-1	ARFIP1	Homo sapiens	41.735	12	12	9558593	2832893
1964	1	P54868	P54868	Hydroxymethylglutaryl-CoA synthase, mitochondrial	HMGCS2	Homo sapiens	56.637	13	12	90679929	312522
1965	1	P55039	P55039	Developmentally-regulated GTP-binding protein 2	DRG2	Homo sapiens	40.745	12	12	4050170	1702705
1966	1	P55290	P55290	Cadherin-13	CDH13	Homo sapiens	78.286	12	12	6095196	2279532
1967	1	P60900	P60900	Proteasome subunit alpha type-6	PSMA6	Homo sapiens	27.398	12	12	17992210	10405992
1968	1	P62241	P62241	40S ribosomal protein S8	RPS8	Homo sapiens	24.208	12	12	35000653	13109517
1969	1	P78539	P78539	Sushi repeat-containing protein SRPX	SRPX	Homo sapiens	51.573	12	12	2447187	159328
1970	1	P80108	P80108	Phosphatidylinositol-glycan-specific phospholipase D	GPLD1	Homo sapiens	92.337	12	12	441759	3196963
1971	1	P82987	P82987	ADAMTS-like protein 3	ADAMTSL3	Homo sapiens	188.694	12	12	2637243	0
1972	1	P98170	P98170	E3 ubiquitin-protein ligase XIAP	XIAP	Homo sapiens	56.685	12	12	1128397	345283
1973	1	Q02487	Q02487	Desmocollin-2	DSC2	Homo sapiens	99.961	14	12	2923192	186757
1974	1	Q02978	Q02978	Mitochondrial 2-oxoglutarate/malate carrier protein	SLC25A11	Homo sapiens	34.058	12	12	15101173	3095022
1975	1	Q03154	Q03154	Aminoacylase-1	ACY1	Homo sapiens	45.886	12	12	4826580	2264583
1976	1	Q04206	Q04206	Transcription factor p65	RELA	Homo sapiens	60.219	12	12	5237446	2895633
1977	1	Q06203	Q06203	Amidophosphoribosyltransferase	PPAT	Homo sapiens	57.399	12	12	2308530	1373442
1978	1	Q07890	Q07890	Son of sevenless homolog 2	SOS2	Homo sapiens	152.982	12	12	228208	1197512
1979	1	Q10713	Q10713	Mitochondrial-processing peptidase subunit alpha	PMPCA	Homo sapiens	58.252	12	12	4998494	1497934
1980	1	Q12904	Q12904	Aminoacyl tRNA synthase complex-interacting multifunctional protein 1	AIMP1	Homo sapiens	34.354	12	12	11357359	5191256
1981	1	Q12905	Q12905	Interleukin enhancer-binding factor 2	ILF2	Homo sapiens	43.064	12	12	21654492	5387866
1982	1	Q12912	Q12912	Inositol 1,4,5-triphosphate receptor associated 2	IRAG2	Homo sapiens	62.123	12	12	300131	1684154

1983	1	Q13206	Q13206	Probable ATP-dependent RNA helicase DDX10	DDX10	Homo sapiens	100.89	12	12	420494	0
1984	1	Q13347	Q13347	Eukaryotic translation initiation factor 3 subunit I	EIF3I	Homo sapiens	36.503	12	12	9230089	4770090
1985	1	Q14108	Q14108	Lysosome membrane protein 2	SCARB2	Homo sapiens	54.294	12	12	6139194	3966881
1986	1	Q14444	Q14444	Caprin-1	CAPRIN1	Homo sapiens	78.364	12	12	5088808	1756873
1987	1	Q14692	Q14692	Ribosome biogenesis protein BMS1 homolog	BMS1	Homo sapiens	145.811	12	12	968456	168827
1988	1	Q14847	Q14847	LIM and SH3 domain protein 1	LASP1	Homo sapiens	29.715	12	12	56849274	25415937
1989	1	Q15018	Q15018	BRISC complex subunit Abraxas 2	ABRAXAS2	Homo sapiens	46.902	12	12	4862439	1434378
1990	1	Q15120	Q15120	[Pyruvate dehydrogenase (acetyl-transferring)] kinase isozyme 3, mitochondrial	PDK3	Homo sapiens	46.939	12	12	2728015	2007613
1991	1	Q15293	Q15293	Reticulocalbin-1	RCN1	Homo sapiens	38.89	12	12	11792696	11587386
1992	1	Q15417	Q15417	Calponin-3	CNN3	Homo sapiens	36.414	13	12	5272214	792723
1993	1	Q15424	Q15424	Scaffold attachment factor B1	SAFB	Homo sapiens	102.643	22	12	3731668	1282166
1994	1	Q15654	Q15654	Thyroid receptor-interacting protein 6	TRIP6	Homo sapiens	50.286	12	12	3136474	181653
1995	1	Q15717	Q15717	ELAV-like protein 1	ELAVL1	Homo sapiens	36.091	13	12	17020119	6219965
1996	1	Q16543	Q16543	Hsp90 co-chaperone Cdc37	CDC37	Homo sapiens	44.467	12	12	18172165	10064146
1997	1	Q16647	Q16647	Prostacyclin synthase	PTGIS	Homo sapiens	57.101	12	12	7721778	0
1998	1	Q16698	Q16698	2,4-dienoyl-CoA reductase [(3E)-enoyl-CoA-producing], mitochondrial	DECR1	Homo sapiens	36.069	12	12	41988690	7376873
1999	1	Q16719	Q16719	Kynureninase	KYNU	Homo sapiens	52.351	12	12	1128600	5415666
2000	1	Q16762	Q16762	Thiosulfate sulfurtransferase	TST	Homo sapiens	33.43	12	12	43571759	4733016
2001	1	Q16832	Q16832	Discoidin domain-containing receptor 2	DDR2	Homo sapiens	96.737	12	12	3540280	41500
2002	1	Q16881	Q16881	Thioredoxin reductase 1, cytoplasmic	TXNRD1	Homo sapiens	70.876	13	12	9427055	18593133
2003	1	Q3KQU3	Q3KQU3	MAP7 domain-containing protein 1	MAP7D1	Homo sapiens	92.822	12	12	3170129	1077538
2004	1	Q4G0F5	Q4G0F5	Vacuolar protein sorting-associated protein 26B	VPS26B	Homo sapiens	39.155	12	12	4511962	2750431
2005	1	Q53EL6	Q53EL6	Programmed cell death protein 4	PDCD4	Homo sapiens	51.735	12	12	15411523	680002
2006	1	Q53GG5	Q53GG5	PDZ and LIM domain protein 3	PDLIM3	Homo sapiens	39.232	12	12	76911685	1773223
2007	1	Q53H82	Q53H82	Endoribonuclease LACTB2	LACTB2	Homo sapiens	32.804	12	12	3364293	860439
2008	1	Q5H9U9	Q5H9U9	Probable ATP-dependent RNA helicase DDX60-like	DDX60L	Homo sapiens	197.678	12	12	81170	695304
2009	1	Q5R3I4	Q5R3I4	Tetratricopeptide repeat protein 38	TTC38	Homo sapiens	52.788	12	12	3052064	65609
2010	1	Q5SWX8	Q5SWX8	Protein odr-4 homolog	ODR4	Homo sapiens	51.103	12	12	1040554	250322
2011	1	Q5T0F9	Q5T0F9	Coiled-coil and C2 domain-containing protein 1B	CC2D1B	Homo sapiens	94.224	12	12	1334841	857775
2012	1	Q5T0W9	Q5T0W9	Protein FAM83B	FAM83B	Homo sapiens	114.797	12	12	721571	45838
2013	1	Q5T2E6	Q5T2E6	Armadillo-like helical domain-containing protein 3	ARMH3	Homo sapiens	78.709	12	12	2598559	1018895
2014	1	Q5T6V5	Q5T6V5	Queuosine salvage protein	C9orf64	Homo sapiens	39.027	12	12	3047630	950861
2015	1	Q5XPI4	Q5XPI4	E3 ubiquitin-protein ligase RNF123	RNF123	Homo sapiens	148.518	12	12	16603336	566640
2016	1	Q6AI08	Q6AI08	HEAT repeat-containing protein 6	HEATR6	Homo sapiens	128.78	12	12	672373	72314
2017	1	Q6JQN1	Q6JQN1	Acyl-CoA dehydrogenase family member 10	ACAD10	Homo sapiens	118.837	12	12	942680	119316
2018	1	Q6NUQ4	Q6NUQ4	Transmembrane protein 214	TMEM214	Homo sapiens	77.151	12	12	2702883	1160622
2019	1	Q6PCB0	Q6PCB0	von Willebrand factor A domain-containing protein 1	VWA1	Homo sapiens	46.802	12	12	25754686	1707964
2020	1	Q6PJI9	Q6PJI9	GATOR complex protein WDR59	WDR59	Homo sapiens	109.795	12	12	1195766	279387
2021	1	Q6UXG2	Q6UXG2	Endosome/lysosome-associated apoptosis and autophagy regulator 1	ELAPOR1	Homo sapiens	111.384	12	12	3439423	0
2022	1	Q6Y7W6	Q6Y7W6	GRB10-interacting GYF protein 2	GIGYF2	Homo sapiens	150.073	12	12	2661803	483096
2023	1	Q6ZMP0	Q6ZMP0	Thrombospondin type-1 domain-containing protein 4	THSD4	Homo sapiens	112.453	12	12	1463710	0
2024	1	Q6ZS17	Q6ZS17	Rho family-interacting cell polarization regulator 1	RIPOR1	Homo sapiens	132.309	12	12	1349441	178447
2025	1	Q6ZXV5	Q6ZXV5	Protein O-mannosyl-transferase TMTC3	TMTC3	Homo sapiens	104.01	12	12	2657012	1080712
2026	1	Q70UQ0	Q70UQ0	Inhibitor of nuclear factor kappa-B kinase-interacting protein	IKBIP	Homo sapiens	39.308	12	12	1802855	3855172
2027	1	Q76MJ5	Q76MJ5	Serine/threonine-protein kinase/endoribonuclease IRE2	ERN2	Homo sapiens	102.481	12	12	1973885	0
2028	1	Q7L099	Q7L099	Protein RUFY3	RUFY3	Homo sapiens	52.965	12	12	2046978	37820
2029	1	Q7L7V1	Q7L7V1	Putative pre-mRNA-splicing factor ATP-dependent RNA helicase DHX32	DHX32	Homo sapiens	84.419	12	12	1312645	0

2030	1 Q7Z304	Q7Z304	MAM domain-containing protein 2	MAMDC2	Homo sapiens	77.556	12	12	2604008	0
2031	1 Q7Z5L9	Q7Z5L9	Interferon regulatory factor 2-binding protein 2	IRF2BP2	Homo sapiens	61.027	12	12	2312157	116825
2032	1 Q7Z5R6	Q7Z5R6	Amyloid beta A4 precursor protein-binding family B member 1-interacting protein	APBB1IP	Homo sapiens	73.18	12	12	1194336	13132316
2033	1 Q7Z7M9	Q7Z7M9	Polypeptide N-acetylgalactosaminyltransferase 5	GALNT5	Homo sapiens	106.265	12	12	1963038	13722
2034	1 Q86TI2	Q86TI2	Dipeptidyl peptidase 9	DPP9	Homo sapiens	98.264	12	12	2601105	1031480
2035	1 Q86TP1	Q86TP1	Exopolyphosphatase PRUNE1	PRUNE1	Homo sapiens	50.201	12	12	1960684	603971
2036	1 Q8IWA5	Q8IWA5	Choline transporter-like protein 2	SLC44A2	Homo sapiens	80.125	12	12	4950789	211980484
2037	1 Q8IWB1	Q8IWB1	Inositol 1,4,5-trisphosphate receptor-interacting protein	ITPRIP	Homo sapiens	62.059	12	12	342328	3816829
2038	1 Q8IWV8	Q8IWV8	E3 ubiquitin-protein ligase UBR2	UBR2	Homo sapiens	200.54	12	12	873294	484215
2039	1 Q8IX01	Q8IX01	SURP and G-patch domain-containing protein 2	SUGP2	Homo sapiens	120.208	12	12	534125	437053
2040	1 Q8IXB1	Q8IXB1	DnaJ homolog subfamily C member 10	DNAJC10	Homo sapiens	91.078	12	12	2289860	1288607
2041	1 Q8IYQ7	Q8IYQ7	Threonine synthase-like 1	THNSL1	Homo sapiens	83.071	12	12	1798358	0
2042	1 Q8IZ07	Q8IZ07	Ankyrin repeat domain-containing protein 13A	ANKRD13A	Homo sapiens	67.619	13	12	2418393	2030299
2043	1 Q8N3U4	Q8N3U4	Cohesin subunit SA-2	STAG2	Homo sapiens	141.329	14	12	1177763	1445206
2044	1 Q8NE86	Q8NE86	Calcium uniporter protein, mitochondrial	MCU	Homo sapiens	39.867	12	12	5071618	3628751
2045	1 Q8NF37	Q8NF37	Lysophosphatidylcholine acyltransferase 1	LPCAT1	Homo sapiens	59.153	12	12	671752	2330526
2046	1 Q8NFQ8	Q8NFQ8	Torsin-1A-interacting protein 2	TOR1AIP2	Homo sapiens	51.264	12	12	3262302	1908144
2047	1 Q8NHU6	Q8NHU6	Tudor domain-containing protein 7	TDRD7	Homo sapiens	123.585	12	12	1551415	1250282
2048	1 Q8TCJ2	Q8TCJ2	Dolichyl-diphosphooligosaccharide--protein glycosyltransferase subunit STT3B	STT3B	Homo sapiens	93.676	12	12	5043218	4787657
2049	1 Q8TD55	Q8TD55	Pleckstrin homology domain-containing family O member 2	PLEKHO2	Homo sapiens	53.349	12	12	3559768	6654743
2050	1 Q8TE77	Q8TE77	Protein phosphatase Slingshot homolog 3	SSH3	Homo sapiens	72.997	12	12	8137170	2187460
2051	1 Q8TEB1	Q8TEB1	DDB1- and CUL4-associated factor 11	DCAF11	Homo sapiens	61.67	12	12	841712	578480
2052	1 Q8WVQ1	Q8WVQ1	Soluble calcium-activated nucleotidase 1	CANT1	Homo sapiens	44.839	12	12	2895611	652261
2053	1 Q8WZA9	Q8WZA9	Immunity-related GTPase family Q protein	IRGQ	Homo sapiens	62.717	12	12	5086556	1248098
2054	1 Q92544	Q92544	Transmembrane 9 superfamily member 4	TM9SF4	Homo sapiens	74.519	12	12	7095486	2779646
2055	1 Q92597	Q92597	Protein NDRG1	NDRG1	Homo sapiens	42.836	12	12	17360204	15329489
2056	1 Q92917	Q92917	G-patch domain and KOW motifs-containing protein	GPKOW	Homo sapiens	52.231	12	12	1683618	289329
2057	1 Q969X5	Q969X5	Endoplasmic reticulum-Golgi intermediate compartment protein 1	ERGIC1	Homo sapiens	32.59	12	12	13113506	3127656
2058	1 Q96CS3	Q96CS3	FAS-associated factor 2	FAF2	Homo sapiens	52.624	12	12	3212769	1671091
2059	1 Q96I24	Q96I24	Far upstream element-binding protein 3	FUBP3	Homo sapiens	61.639	12	12	6838069	1875032
2060	1 Q96I25	Q96I25	Splicing factor 45	RBM17	Homo sapiens	44.96	12	12	2269081	545495
2061	1 Q96JJ3	Q96JJ3	Engulfment and cell motility protein 2	ELMO2	Homo sapiens	82.616	12	12	2103827	1381914
2062	1 Q96KG9	Q96KG9	N-terminal kinase-like protein	SCYL1	Homo sapiens	89.635	12	12	2798610	1208192
2063	1 Q96L93	Q96L93	Kinesin-like protein KIF16B	KIF16B	Homo sapiens	152.014	12	12	1229252	25205
2064	1 Q96NW4	Q96NW4	Ankyrin repeat domain-containing protein 27	ANKRD27	Homo sapiens	116.988	12	12	853676	359227
2065	1 Q96P70	Q96P70	Importin-9	IPO9	Homo sapiens	115.964	12	12	1548695	537546
2066	1 Q96QZ7	Q96QZ7	Membrane-associated guanylate kinase, WW and PDZ domain-containing protein 1	MAGI1	Homo sapiens	164.582	12	12	1060789	26683
2067	1 Q96RF0	Q96RF0	Sorting nexin-18	SNX18	Homo sapiens	68.895	12	12	4079209	3367255
2068	1 Q96RU3	Q96RU3	Formin-binding protein 1	FNBP1	Homo sapiens	71.309	12	12	5219665	2937741
2069	1 Q96S52	Q96S52	GPI transamidase component PIG-S	PIGS	Homo sapiens	61.657	12	12	2834181	1914933
2070	1 Q99102	Q99102	Mucin-4	MUC4	Homo sapiens	542.318	12	12	1063757	0
2071	1 Q99683	Q99683	Mitogen-activated protein kinase kinase kinase 5	MAP3K5	Homo sapiens	154.537	13	12	313903	858862
2072	1 Q9BQ52	Q9BQ52	Zinc phosphodiesterase ELAC protein 2	ELAC2	Homo sapiens	92.221	12	12	1347818	246052
2073	1 Q9BR39	Q9BR39	Junctophilin-2	JPH2	Homo sapiens	74.222	12	12	4486070	0
2074	1 Q9BR76	Q9BR76	Coronin-1B	CORO1B	Homo sapiens	54.239	12	12	12792389	3439848

2075	1	Q9BRK5	Q9BRK5	45 kDa calcium-binding protein	SDF4	Homo sapiens	41.808	12	12	5517406	1787264
2076	1	Q9BWS9	Q9BWS9	Chitinase domain-containing protein 1	CHID1	Homo sapiens	44.939	12	12	3523052	945875
2077	1	Q9BXR6	Q9BXR6	Complement factor H-related protein 5	CFHR5	Homo sapiens	64.42	13	12	1370281	12946165
2078	1	Q9BXS5	Q9BXS5	AP-1 complex subunit mu-1	AP1M1	Homo sapiens	48.586	14	12	4231294	4183188
2079	1	Q9BYT8	Q9BYT8	Neurolysin, mitochondrial	NLN	Homo sapiens	80.65	12	12	2693419	1617114
2080	1	Q9BYX2	Q9BYX2	TBC1 domain family member 2A	TBC1D2	Homo sapiens	105.414	12	12	800206	2143843
2081	1	Q9H078	Q9H078	Caseinolytic peptidase B protein homolog	CLPB	Homo sapiens	78.728	12	12	2968035	1729850
2082	1	Q9H0P0	Q9H0P0	Cytosolic 5'-nucleotidase 3A	NT5C3A	Homo sapiens	37.947	12	12	2671341	2017873
2083	1	Q9H2U2	Q9H2U2	Inorganic pyrophosphatase 2, mitochondrial	PPA2	Homo sapiens	37.92	12	12	20805665	6369981
2084	1	Q9H6R4	Q9H6R4	Nucleolar protein 6	NOL6	Homo sapiens	127.594	12	12	843193	139833
2085	1	Q9H9T3	Q9H9T3	Elongator complex protein 3	ELP3	Homo sapiens	62.259	12	12	1729320	367187
2086	1	Q9NPB8	Q9NPB8	Glycerophosphocholine phosphodiesterase GPCPD1	GPCPD1	Homo sapiens	76.035	12	12	981753	2207364
2087	1	Q9NR46	Q9NR46	Endophilin-B2	SH3GLB2	Homo sapiens	43.977	12	12	8389559	2349449
2088	1	Q9NT22	Q9NT22	EMILIN-3	EMILIN3	Homo sapiens	82.648	12	12	1412469	0
2089	1	Q9NUQ7	Q9NUQ7	Ufm1-specific protease 2	UFSP2	Homo sapiens	53.261	12	12	2455223	498978
2090	1	Q9NUQ9	Q9NUQ9	CYFIP-related Rac1 interactor B	CYRIB	Homo sapiens	36.75	14	12	7118188	14105411
2091	1	Q9NW15	Q9NW15	Anoctamin-10	ANO10	Homo sapiens	76.33	12	12	4773562	6223567
2092	1	Q9NYL2	Q9NYL2	Mitogen-activated protein kinase kinase kinase 20	MAP3K20	Homo sapiens	91.157	12	12	4880106	681979
2093	1	Q9P270	Q9P270	SLAIN motif-containing protein 2	SLAIN2	Homo sapiens	62.545	12	12	2611560	518569
2094	1	Q9UBQ7	Q9UBQ7	Glyoxylate reductase/hydroxypyruvate reductase	GRHPR	Homo sapiens	35.668	12	12	9343579	2252855
2095	1	Q9UBT7	Q9UBT7	Alpha-catulin	CTNNAL1	Homo sapiens	81.897	12	12	1081117	0
2096	1	Q9UBV2	Q9UBV2	Protein sel-1 homolog 1	SEL1L	Homo sapiens	88.754	12	12	5341715	1814709
2097	1	Q9UHX1	Q9UHX1	Poly(U)-binding-splicing factor PUF60	PUF60	Homo sapiens	59.876	12	12	7692305	1743140
2098	1	Q9UI10	Q9UI10	Translation initiation factor eIF-2B subunit delta	EIF2B4	Homo sapiens	57.558	12	12	2672440	680088
2099	1	Q9UI26	Q9UI26	Importin-11	IPO11	Homo sapiens	112.535	12	12	1928861	375969
2100	1	Q9UIJ7	Q9UIJ7	GTP:AMP phosphotransferase AK3, mitochondrial	AK3	Homo sapiens	25.566	12	12	24833007	5130003
2101	1	Q9UJ70	Q9UJ70	N-acetyl-D-glucosamine kinase	NAGK	Homo sapiens	37.374	12	12	13761472	12758186
2102	1	Q9UJC3	Q9UJC3	Protein Hook homolog 1	HOOK1	Homo sapiens	84.648	12	12	1950309	152172
2103	1	Q9UKF6	Q9UKF6	Cleavage and polyadenylation specificity factor subunit 3	CPSF3	Homo sapiens	77.486	12	12	3230840	858502
2104	1	Q9UKN8	Q9UKN8	General transcription factor 3C polypeptide 4	GTF3C4	Homo sapiens	91.982	12	12	1071441	271204
2105	1	Q9UKS6	Q9UKS6	Protein kinase C and casein kinase substrate in neurons protein 3	PACSIN3	Homo sapiens	48.486	12	12	3667742	33872
2106	1	Q9UKX7	Q9UKX7	Nuclear pore complex protein Nup50	NUP50	Homo sapiens	50.145	12	12	4092431	4167787
2107	1	Q9UN86	Q9UN86	Ras GTPase-activating protein-binding protein 2	G3BP2	Homo sapiens	54.12	12	12	2875617	242867
2108	1	Q9UNA1	Q9UNA1	Rho GTPase-activating protein 26	ARHGAP26	Homo sapiens	92.237	12	12	603707	1956392
2109	1	Q9UPR0	Q9UPR0	Inactive phospholipase C-like protein 2	PLCL2	Homo sapiens	125.866	12	12	528217	790121
2110	1	Q9Y224	Q9Y224	RNA transcription, translation and transport factor protein	RTRAF	Homo sapiens	28.069	12	12	8663358	2339840
2111	1	Q9Y2H1	Q9Y2H1	Serine/threonine-protein kinase 38-like	STK38L	Homo sapiens	54.006	14	12	3828023	414541
2112	1	Q9Y2Z4	Q9Y2Z4	Tyrosine--tRNA ligase, mitochondrial	YARS2	Homo sapiens	53.197	12	12	1428597	389475
2113	1	Q9Y2Z9	Q9Y2Z9	Ubiquinone biosynthesis monooxygenase COQ6, mitochondrial	COQ6	Homo sapiens	50.872	12	12	1536371	0
2114	1	Q9Y4D1	Q9Y4D1	Disheveled-associated activator of morphogenesis 1	DAAM1	Homo sapiens	123.474	13	12	2180702	171277
2115	1	Q9Y4D7	Q9Y4D7	Plexin-D1	PLXND1	Homo sapiens	212.011	12	12	571569	402394
2116	1	Q9Y570	Q9Y570	Protein phosphatase methylesterase 1	PPME1	Homo sapiens	42.316	12	12	4500655	2636508
2117	1	Q9Y5X1	Q9Y5X1	Sorting nexin-9	SNX9	Homo sapiens	66.593	12	12	7781189	1474419
2118	1	Q9Y666	Q9Y666	Solute carrier family 12 member 7	SLC12A7	Homo sapiens	119.107	13	12	806587	867314
2119	1	Q9Y6Q2	Q9Y6Q2	Stonin-1	STON1	Homo sapiens	83.143	12	12	3419657	0
2120	1	A1A4S6	A1A4S6	Rho GTPase-activating protein 10	ARHGAP10	Homo sapiens	89.377	11	11	3059848	55120
2121	1	A2RTX5	A2RTX5	Threonine--tRNA ligase 2, cytoplasmic	TARS3	Homo sapiens	92.649	11	11	1167841	0
2122	1	A2RUS2	A2RUS2	DENN domain-containing protein 3	DENND3	Homo sapiens	135.893	11	11	129656	1655169

2123	1	A4D1P6	A4D1P6	WD repeat-containing protein 91	WDR91	Homo sapiens	83.344	11	11	1478431	1165357
2124	1	O00411	O00411	DNA-directed RNA polymerase, mitochondrial	POLRMT	Homo sapiens	138.622	11	11	647674	27339
2125	1	O00499	O00499	Myc box-dependent-interacting protein 1	BIN1	Homo sapiens	64.701	11	11	4660640	1527917
2126	1	O14786	O14786	Neuropilin-1	NRP1	Homo sapiens	103.136	11	11	5865250	1184937
2127	1	O14879	O14879	Interferon-induced protein with tetratricopeptide repeats 3	IFIT3	Homo sapiens	55.985	11	11	1023673	13745639
2128	1	O14975	O14975	Long-chain fatty acid transport protein 2	SLC27A2	Homo sapiens	70.313	11	11	2160619	1869095
2129	1	O15111	O15111	Inhibitor of nuclear factor kappa-B kinase subunit alpha	CHUK	Homo sapiens	84.641	11	11	1123324	1071144
2130	1	O15254	O15254	Peroxisomal acyl-coenzyme A oxidase 3	ACOX3	Homo sapiens	77.63	11	11	1347900	1290950
2131	1	O15269	O15269	Serine palmitoyltransferase 1	SPTLC1	Homo sapiens	52.742	11	11	2418684	1303696
2132	1	O15355	O15355	Protein phosphatase 1G	PPM1G	Homo sapiens	59.271	11	11	6094979	3260852
2133	1	O15372	O15372	Eukaryotic translation initiation factor 3 subunit H	EIF3H	Homo sapiens	39.931	11	11	9854012	3609955
2134	1	O43493	O43493	Trans-Golgi network integral membrane protein 2	TGOLN2	Homo sapiens	45.88	11	11	5472342	3266523
2135	1	O43566	O43566	Regulator of G-protein signaling 14	RGS14	Homo sapiens	61.446	11	11	58357	1857407
2136	1	O43765	O43765	Small glutamine-rich tetratricopeptide repeat-containing protein alpha	SGTA	Homo sapiens	34.063	11	11	4905897	3132567
2137	1	O43896	O43896	Kinesin-like protein KIF1C	KIF1C	Homo sapiens	122.95	11	11	1770295	293754
2138	1	O60282	O60282	Kinesin heavy chain isoform 5C	KIF5C	Homo sapiens	109.495	12	11	939364	0
2139	1	O60508	O60508	Pre-mRNA-processing factor 17	CDC40	Homo sapiens	65.521	11	11	1436814	472463
2140	1	O75027	O75027	Iron-sulfur clusters transporter ABCB7, mitochondrial	ABCB7	Homo sapiens	82.644	11	11	2693983	782014
2141	1	O75155	O75155	Cullin-associated NEDD8-dissociated protein 2	CAND2	Homo sapiens	135.259	11	11	2459730	0
2142	1	O75436	O75436	Vacuolar protein sorting-associated protein 26A	VPS26A	Homo sapiens	38.169	11	11	16448932	13642188
2143	1	O75558	O75558	Syntaxin-11	STX11	Homo sapiens	33.197	11	11	303331	3441178
2144	1	O75781	O75781	Paralemmin-1	PALM	Homo sapiens	42.075	11	11	5827557	49006
2145	1	O75995	O75995	SAM and SH3 domain-containing protein 3	SASH3	Homo sapiens	41.594	11	11	666873	3560427
2146	1	O94759	O94759	Transient receptor potential cation channel subfamily M member 2	TRPM2	Homo sapiens	171.2	11	11	30063	1107818
2147	1	O94885	O94885	SAM and SH3 domain-containing protein 1	SASH1	Homo sapiens	136.655	13	11	574727	46986
2148	1	O94888	O94888	UBX domain-containing protein 7	UBXN7	Homo sapiens	54.863	11	11	1514664	313953
2149	1	O95210	O95210	Starch-binding domain-containing protein 1	STBD1	Homo sapiens	39.009	11	11	3397482	6573417
2150	1	O95260	O95260	Arginyl-tRNA--protein transferase 1	ATE1	Homo sapiens	59.093	11	11	1624106	734420
2151	1	O95470	O95470	Sphingosine-1-phosphate lyase 1	SGPL1	Homo sapiens	63.524	11	11	5335885	2592404
2152	1	O95671	O95671	Probable bifunctional dTTP/UTP pyrophosphatase/methyltransferase protein	ASMTL	Homo sapiens	68.856	11	11	5492988	960835
2153	1	O95721	O95721	Synaptosomal-associated protein 29	SNAP29	Homo sapiens	28.971	11	11	4597347	1916290
2154	1	O95741	O95741	Copine-6	CPNE6	Homo sapiens	61.991	11	11	1723885	0
2155	1	O95759	O95759	TBC1 domain family member 8	TBC1D8	Homo sapiens	130.837	11	11	13492	659286
2156	1	O95822	O95822	Malonyl-CoA decarboxylase, mitochondrial	MLYCD	Homo sapiens	55.004	11	11	2141810	721179
2157	1	O96000	O96000	NADH dehydrogenase [ubiquinone] 1 beta subcomplex subunit 10	NDUFB10	Homo sapiens	20.775	11	11	23513152	4712110
2158	1	P00519	P00519	Tyrosine-protein kinase ABL1	ABL1	Homo sapiens	122.873	13	11	1565062	42262
2159	1	P00742	P00742	Coagulation factor X	F10	Homo sapiens	54.731	14	11	530527	7375336
2160	1	P00813	P00813	Adenosine deaminase	ADA	Homo sapiens	40.765	11	11	2327044	1710994
2161	1	P02760	P02760	Protein AMBP	AMBP	Homo sapiens	39.001	11	11	11908190	21524778
2162	1	P04181	P04181	Ornithine aminotransferase, mitochondrial	OAT	Homo sapiens	48.533	11	11	3961774	2798988
2163	1	P04792	P04792	Heat shock protein beta-1	HSPB1	Homo sapiens	22.783	11	11	337848644	22417653
2164	1	P04899	P04899	Guanine nucleotide-binding protein G(i) subunit alpha-2	GNAI2	Homo sapiens	40.452	16	11	14826972	34097636
2165	1	P05546	P05546	Heparin cofactor 2	SERPIND1	Homo sapiens	57.071	11	11	9790186	35672966
2166	1	P07602	P07602	Prosaposin	PSAP	Homo sapiens	58.113	11	11	4956188	7927954
2167	1	P09110	P09110	3-ketoacyl-CoA thiolase, peroxisomal	ACAA1	Homo sapiens	44.291	11	11	7472775	4743518
2168	1	P09467	P09467	Fructose-1,6-bisphosphatase 1	FBP1	Homo sapiens	36.842	12	11	5685054	2738121
2169	1	P09661	P09661	U2 small nuclear ribonucleoprotein A'	SNRPA1	Homo sapiens	28.415	11	11	9999919	4842250

2170	1	P10398	P10398	Serine/threonine-protein kinase A-Raf	ARAF	Homo sapiens	67.586	15	11	2133071	1427390
2171	1	P11310	P11310	Medium-chain specific acyl-CoA dehydrogenase, mitochondrial	ACADM	Homo sapiens	46.589	11	11	13380735	4046566
2172	1	P11388	P11388	DNA topoisomerase 2-alpha	TOP2A	Homo sapiens	174.387	11	11	855581	341482
2173	1	P12532	P12532	Creatine kinase U-type, mitochondrial	CKMT1B	Homo sapiens	47.036	12	11	25572931	4394286
2174	1	P12955	P12955	Xaa-Pro dipeptidase	PEPD	Homo sapiens	54.549	11	11	8191342	3203605
2175	1	P13591	P13591	Neural cell adhesion molecule 1	NCAM1	Homo sapiens	94.574	22	11	9974106	170647
2176	1	P16930	P16930	Fumarylacetoacetase	FAH	Homo sapiens	46.374	11	11	5685464	2140481
2177	1	P18858	P18858	DNA ligase 1	LIG1	Homo sapiens	101.739	11	11	1413059	3640251
2178	1	P19021	P19021	Peptidyl-glycine alpha-amidating monooxygenase	PAM	Homo sapiens	108.333	11	11	1088228	25871
2179	1	P20591	P20591	Interferon-induced GTP-binding protein Mx1	MX1	Homo sapiens	75.524	11	11	3212761	5370634
2180	1	P21810	P21810	Biglycan	BGN	Homo sapiens	41.654	11	11	77918278	11595511
2181	1	P21912	P21912	Succinate dehydrogenase [ubiquinone] iron-sulfur subunit, mitochondrial	SDHB	Homo sapiens	31.629	11	11	17902484	4062019
2182	1	P22087	P22087	rRNA 2'-O-methyltransferase fibrillarin	FBL	Homo sapiens	33.783	12	11	13786695	6295927
2183	1	P22413	P22413	Ectonucleotide pyrophosphatase/phosphodiesterase family member 1	ENPP1	Homo sapiens	104.924	11	11	586350	0
2184	1	P22676	P22676	Calretinin	CALB2	Homo sapiens	31.541	12	11	3183948	873925
2185	1	P22748	P22748	Carbonic anhydrase 4	CA4	Homo sapiens	35.031	11	11	5000287	15049168
2186	1	P24752	P24752	Acetyl-CoA acetyltransferase, mitochondrial	ACAT1	Homo sapiens	45.197	11	11	54485241	7365682
2187	1	P25685	P25685	DnaJ homolog subfamily B member 1	DNAJB1	Homo sapiens	38.043	12	11	6353313	4205420
2188	1	P26440	P26440	Isovaleryl-CoA dehydrogenase, mitochondrial	IVD	Homo sapiens	46.652	11	11	4895305	1343288
2189	1	P27986	P27986	Phosphatidylinositol 3-kinase regulatory subunit alpha	PIK3R1	Homo sapiens	83.6	13	11	1452754	1546731
2190	1	P28074	P28074	Proteasome subunit beta type-5	PSMB5	Homo sapiens	28.48	11	11	9806378	2627973
2191	1	P30519	P30519	Heme oxygenase 2	HMOX2	Homo sapiens	36.034	11	11	5832484	3759812
2192	1	P32121	P32121	Beta-arrestin-2	ARRB2	Homo sapiens	46.107	11	11	619910	5656862
2193	1	P35240	P35240	Merlin	NF2	Homo sapiens	69.692	11	11	933736	771801
2194	1	P35249	P35249	Replication factor C subunit 4	RFC4	Homo sapiens	39.681	11	11	1603016	1199189
2195	1	P36269	P36269	Glutathione hydrolase 5 proenzyme	GGT5	Homo sapiens	62.259	11	11	3563023	414099
2196	1	P40121	P40121	Macrophage-capping protein	CAPG	Homo sapiens	38.499	11	11	22085395	44552837
2197	1	P42336	P42336	Phosphatidylinositol 4,5-bisphosphate 3-kinase catalytic subunit alpha isoform	PIK3CA	Homo sapiens	124.284	11	11	1010674	18526
2198	1	P42696	P42696	RNA-binding protein 34	RBM34	Homo sapiens	48.563	11	11	1046602	242006
2199	1	P42892	P42892	Endothelin-converting enzyme 1	ECE1	Homo sapiens	87.163	11	11	2361864	969062
2200	1	P43250	P43250	G protein-coupled receptor kinase 6	GRK6	Homo sapiens	65.992	13	11	629482	4885510
2201	1	P47895	P47895	Aldehyde dehydrogenase family 1 member A3	ALDH1A3	Homo sapiens	56.108	11	11	2948474	73945
2202	1	P48163	P48163	NADP-dependent malic enzyme	ME1	Homo sapiens	64.15	11	11	4173973	140870
2203	1	P48382	P48382	DNA-binding protein RFX5	RFX5	Homo sapiens	65.324	11	11	1561030	239830
2204	1	P48740	P48740	Mannan-binding lectin serine protease 1	MASP1	Homo sapiens	79.248	11	11	815053	2060763
2205	1	P49023	P49023	Paxillin	PXN	Homo sapiens	64.507	11	11	3702427	3426653
2206	1	P49354	P49354	Protein farnesyltransferase/geranylgeranyltransferase type-1 subunit alpha	FNTA	Homo sapiens	44.41	11	11	4065428	3382964
2207	1	P49407	P49407	Beta-arrestin-1	ARRB1	Homo sapiens	47.065	11	11	25930680	2820944
2208	1	P49746	P49746	Thrombospondin-3	THBS3	Homo sapiens	104.201	11	11	3397048	104964
2209	1	P50452	P50452	Serpin B8	SERPINB8	Homo sapiens	42.767	11	11	1962775	3807167
2210	1	P50542	P50542	Peroxisomal targeting signal 1 receptor	PEX5	Homo sapiens	70.864	11	11	1131695	111829
2211	1	P51532	P51532	Transcription activator BRG1	SMARCA4	Homo sapiens	184.647	11	11	2698475	460280
2212	1	P51813	P51813	Cytoplasmic tyrosine-protein kinase BMX	BMX	Homo sapiens	78.013	11	11	15181	1914481
2213	1	P52735	P52735	Guanine nucleotide exchange factor VAV2	VAV2	Homo sapiens	101.29	11	11	1349258	569776
2214	1	P53041	P53041	Serine/threonine-protein phosphatase 5	PPP5C	Homo sapiens	56.882	11	11	3352197	971139
2215	1	P53582	P53582	Methionine aminopeptidase 1	METAP1	Homo sapiens	43.216	11	11	4273229	1292336

2216	1	P54802	P54802	Alpha-N-acetylglucosaminidase	NAGLU	Homo sapiens	82.267	11	11	1099481	182165
2217	1	P55212	P55212	Caspase-6	CASP6	Homo sapiens	33.308	11	11	5048034	1447837
2218	1	P56524	P56524	Histone deacetylase 4	HDAC4	Homo sapiens	119.04	12	11	1122535	337148
2219	1	P61247	P61247	40S ribosomal protein S3a	RPS3A	Homo sapiens	29.947	11	11	45817157	15254834
2220	1	P78310	P78310	Coxsackievirus and adenovirus receptor	CXADR	Homo sapiens	40.032	11	11	2199925	230335
2221	1	P78357	P78357	Contactin-associated protein 1	CNTNAP1	Homo sapiens	156.268	11	11	1166389	0
2222	1	P80404	P80404	4-aminobutyrate aminotransferase, mitochondrial	ABAT	Homo sapiens	56.439	11	11	2495316	74984
2223	1	P98175	P98175	RNA-binding protein 10	RBM10	Homo sapiens	103.535	11	11	2134539	599640
2224	1	P98194	P98194	Calcium-transporting ATPase type 2C member 1	ATP2C1	Homo sapiens	100.577	11	11	3180919	967385
2225	1	Q00534	Q00534	Cyclin-dependent kinase 6	CDK6	Homo sapiens	36.939	11	11	1919866	0
2226	1	Q02388	Q02388	Collagen alpha-1(VII) chain	COL7A1	Homo sapiens	295.222	11	11	194680	1924105
2227	1	Q02878	Q02878	60S ribosomal protein L6	RPL6	Homo sapiens	32.728	11	11	24322116	7325999
2228	1	Q03519	Q03519	Antigen peptide transporter 2	TAP2	Homo sapiens	75.663	11	11	3644187	4857547
2229	1	Q03701	Q03701	CCAAT/enhancer-binding protein zeta	CEBPZ	Homo sapiens	120.977	11	11	929382	300602
2230	1	Q08AM6	Q08AM6	Protein VAC14 homolog	VAC14	Homo sapiens	87.975	11	11	3615852	1756908
2231	1	Q12765	Q12765	Secernin-1	SCRN1	Homo sapiens	46.383	11	11	4937653	1568572
2232	1	Q12846	Q12846	Syntaxin-4	STX4	Homo sapiens	34.18	11	11	4937839	2355684
2233	1	Q12849	Q12849	G-rich sequence factor 1	GRSF1	Homo sapiens	53.127	11	11	3099498	708370
2234	1	Q13126	Q13126	S-methyl-5'-thioadenosine phosphorylase	MTAP	Homo sapiens	31.235	11	11	9826874	4518793
2235	1	Q13177	Q13177	Serine/threonine-protein kinase PAK 2	PAK2	Homo sapiens	58.044	18	11	11453333	9069108
2236	1	Q13217	Q13217	DnaJ homolog subfamily C member 3	DNAJC3	Homo sapiens	57.58	11	11	3059355	6649564
2237	1	Q13325	Q13325	Interferon-induced protein with tetratricopeptide repeats 5	IFIT5	Homo sapiens	55.845	11	11	1356880	386687
2238	1	Q13332	Q13332	Receptor-type tyrosine-protein phosphatase S	PTPRS	Homo sapiens	217.045	14	11	1160999	23246
2239	1	Q13438	Q13438	Protein OS-9	OS9	Homo sapiens	75.562	11	11	1738576	1557334
2240	1	Q13510	Q13510	Acid ceramidase	ASAH1	Homo sapiens	44.662	11	11	12865309	7848552
2241	1	Q13523	Q13523	Serine/threonine-protein kinase PRP4 homolog	PRPF4B	Homo sapiens	116.989	11	11	1422502	416955
2242	1	Q13630	Q13630	GDP-L-fucose synthase	GFUS	Homo sapiens	35.893	11	11	10720663	5193127
2243	1	Q13884	Q13884	Beta-1-syntrophin	SNTB1	Homo sapiens	58.063	11	11	3236632	1313578
2244	1	Q14165	Q14165	Malectin	MLEC	Homo sapiens	32.231	11	11	16880540	8184112
2245	1	Q14194	Q14194	Dihydropyrimidinase-related protein 1	CRMP1	Homo sapiens	62.185	11	11	4924679	0
2246	1	Q14314	Q14314	Fibroleukin	FGL2	Homo sapiens	50.229	11	11	1207653	3805418
2247	1	Q14435	Q14435	Polypeptide N-acetylgalactosaminyltransferase 3	GALNT3	Homo sapiens	72.611	12	11	4320759	1313195
2248	1	Q14562	Q14562	ATP-dependent RNA helicase DHX8	DHX8	Homo sapiens	139.314	11	11	924864	271597
2249	1	Q14790	Q14790	Caspase-8	CASP8	Homo sapiens	55.389	11	11	1227994	2587035
2250	1	Q15067	Q15067	Peroxisomal acyl-coenzyme A oxidase 1	ACOX1	Homo sapiens	74.425	11	11	2967631	1758525
2251	1	Q15118	Q15118	[Pyruvate dehydrogenase (acetyl-transferring)] kinase isozyme 1, mitochondrial	PDK1	Homo sapiens	49.246	11	11	1978553	330573
2252	1	Q15365	Q15365	Poly(rC)-binding protein 1	PCBP1	Homo sapiens	37.497	14	11	50833079	30393732
2253	1	Q15599	Q15599	Na(+)/H(+) exchange regulatory cofactor NHE-RF2	NHERF2	Homo sapiens	37.414	11	11	2638811	215763
2254	1	Q15648	Q15648	Mediator of RNA polymerase II transcription subunit 1	MED1	Homo sapiens	168.48	11	11	7936922	189793
2255	1	Q15751	Q15751	Probable E3 ubiquitin-protein ligase HERC1	HERC1	Homo sapiens	532.236	11	11	340936	205122
2256	1	Q16658	Q16658	Fascin	FSCN1	Homo sapiens	54.531	11	11	7635946	4282122
2257	1	Q2NL82	Q2NL82	Pre-rRNA-processing protein TSR1 homolog	TSR1	Homo sapiens	91.811	11	11	2112751	993456
2258	1	Q2TAY7	Q2TAY7	WD40 repeat-containing protein SMU1	SMU1	Homo sapiens	57.547	11	11	3053654	1231742
2259	1	Q5HYK3	Q5HYK3	2-methoxy-6-polyprenyl-1,4-benzoquinol methylase, mitochondrial	COQ5	Homo sapiens	37.143	11	11	5854807	199250
2260	1	Q5JSP0	Q5JSP0	FYVE, RhoGEF and PH domain-containing protein 3	FGD3	Homo sapiens	79.402	11	11	105315	2386204
2261	1	Q5JTD0	Q5JTD0	Tight junction-associated protein 1	TJAP1	Homo sapiens	61.821	11	11	1179184	105208
2262	1	Q5K4L6	Q5K4L6	Long-chain fatty acid transport protein 3	SLC27A3	Homo sapiens	73.552	11	11	2233569	707431

2263	1	Q5M775	Q5M775	Cytospin-B	SPECC1	Homo sapiens	118.587	11	11	861435	259887
2264	1	Q5NDL2	Q5NDL2	EGF domain-specific O-linked N-acetylglucosamine transferase	EOGT	Homo sapiens	62.012	11	11	1752495	484661
2265	1	Q5QJ74	Q5QJ74	Tubulin-specific chaperone cofactor E-like protein	TBCEL	Homo sapiens	48.195	11	11	7050013	240262
2266	1	Q5R372	Q5R372	Rab GTPase-activating protein 1-like	RABGAP1L	Homo sapiens	92.513	11	11	1105768	122741
2267	1	Q5T8P6	Q5T8P6	RNA-binding protein 26	RBM26	Homo sapiens	113.598	12	11	1093916	798627
2268	1	Q5TBA9	Q5TBA9	Protein furry homolog	FRY	Homo sapiens	338.881	11	11	605815	162246
2269	1	Q5TDH0	Q5TDH0	Protein DDI1 homolog 2	DDI2	Homo sapiens	44.522	11	11	3901802	3573124
2270	1	Q5TH69	Q5TH69	Brefeldin A-inhibited guanine nucleotide-exchange protein 3	ARFGEF3	Homo sapiens	240.653	11	11	766734	0
2271	1	Q658Y4	Q658Y4	Protein FAM91A1	FAM91A1	Homo sapiens	93.912	12	11	1113760	717077
2272	1	Q684P5	Q684P5	Rap1 GTPase-activating protein 2	RAP1GAP2	Homo sapiens	80.056	12	11	582306	1135264
2273	1	Q68E01	Q68E01	Integrator complex subunit 3	INTS3	Homo sapiens	118.072	11	11	1152915	281282
2274	1	Q6DD88	Q6DD88	Atlastin-3	ATL3	Homo sapiens	60.542	11	11	10797218	5177198
2275	1	Q6GYQ0	Q6GYQ0	Ral GTPase-activating protein subunit alpha-1	RALGAPA1	Homo sapiens	229.837	11	11	916171	132082
2276	1	Q6NYC8	Q6NYC8	Phostensin	PPP1R18	Homo sapiens	67.943	11	11	4636331	2461828
2277	1	Q6P4A8	Q6P4A8	Phospholipase B-like 1	PLBD1	Homo sapiens	63.257	11	11	1947398	7741485
2278	1	Q6P4Q7	Q6P4Q7	Metal transporter CNNM4	CNNM4	Homo sapiens	86.605	14	11	2154467	1477927
2279	1	Q6UB35	Q6UB35	Monofunctional C1-tetrahydrofolate synthase, mitochondrial	MTHFD1L	Homo sapiens	105.788	11	11	594127	845561
2280	1	Q6ULP2	Q6ULP2	Aftiphilin	AFTPH	Homo sapiens	102.115	11	11	1947898	210324
2281	1	Q6UW02	Q6UW02	Cytochrome P450 20A1	CYP20A1	Homo sapiens	52.431	11	11	3372678	770452
2282	1	Q6UWP2	Q6UWP2	Dehydrogenase/reductase SDR family member 11	DHRS11	Homo sapiens	28.306	11	11	9974074	364050
2283	1	Q6UWY5	Q6UWY5	Olfactomedin-like protein 1	OLFML1	Homo sapiens	45.95	11	11	11207806	184024
2284	1	Q6XQN6	Q6XQN6	Nicotinate phosphoribosyltransferase	NAPRT	Homo sapiens	57.578	11	11	3563024	2092337
2285	1	Q6ZT12	Q6ZT12	E3 ubiquitin-protein ligase UBR3	UBR3	Homo sapiens	212.434	11	11	818468	0
2286	1	Q6ZU35	Q6ZU35	Capping protein-inhibiting regulator of actin dynamics	CRACD	Homo sapiens	136.762	11	11	733592	0
2287	1	Q7Z3C6	Q7Z3C6	Autophagy-related protein 9A	ATG9A	Homo sapiens	94.449	11	11	1236421	130805
2288	1	Q7Z3V4	Q7Z3V4	Ubiquitin-protein ligase E3B	UBE3B	Homo sapiens	123.1	11	11	632256	89625
2289	1	Q7Z4H8	Q7Z4H8	Protein O-glucosyltransferase 3	POGLUT3	Homo sapiens	58.572	11	11	2326333	605708
2290	1	Q86U44	Q86U44	N6-adenosine-methyltransferase catalytic subunit	METTL3	Homo sapiens	64.475	11	11	1640479	760288
2291	1	Q86X29	Q86X29	Lipolysis-stimulated lipoprotein receptor	LSR	Homo sapiens	71.439	11	11	5625247	757796
2292	1	Q86X55	Q86X55	Histone-arginine methyltransferase CARM1	CARM1	Homo sapiens	65.854	11	11	5092915	1190581
2293	1	Q86XA9	Q86XA9	HEAT repeat-containing protein 5A	HEATR5A	Homo sapiens	222.006	11	11	913657	172956
2294	1	Q8IUI8	Q8IUI8	Cytokine receptor-like factor 3	CRLF3	Homo sapiens	49.769	11	11	1333099	3665660
2295	1	Q8IV36	Q8IV36	Protein HID1	HID1	Homo sapiens	88.747	11	11	2328951	87912
2296	1	Q8IVH4	Q8IVH4	Methylmalonic aciduria type A protein, mitochondrial	MMAA	Homo sapiens	46.539	11	11	1431697	606773
2297	1	Q8IVI9	Q8IVI9	Nostrin	NOSTRIN	Homo sapiens	57.661	11	11	2673445	553338
2298	1	Q8IXJ6	Q8IXJ6	NAD-dependent protein deacetylase sirtuin-2	SIRT2	Homo sapiens	43.182	11	11	2416016	1126243
2299	1	Q8IY67	Q8IY67	Ribonucleoprotein PTB-binding 1	RAVER1	Homo sapiens	63.879	11	11	2045807	950622
2300	1	Q8IZ21	Q8IZ21	Phosphatase and actin regulator 4	PHACTR4	Homo sapiens	78.213	11	11	1393493	48823
2301	1	Q8IZL8	Q8IZL8	Proline-, glutamic acid- and leucine-rich protein 1	PELP1	Homo sapiens	119.704	11	11	1568014	300656
2302	1	Q8N335	Q8N335	Glycerol-3-phosphate dehydrogenase 1-like protein	GPD1L	Homo sapiens	38.418	11	11	11238725	2367811
2303	1	Q8N3E9	Q8N3E9	1-phosphatidylinositol 4,5-bisphosphate phosphodiesterase delta-3	PLCD3	Homo sapiens	89.26	11	11	1426502	0
2304	1	Q8N4X5	Q8N4X5	Actin filament-associated protein 1-like 2	AFAP1L2	Homo sapiens	91.301	11	11	1470119	420112
2305	1	Q8N556	Q8N556	Actin filament-associated protein 1	AFAP1	Homo sapiens	80.726	11	11	1598160	64666
2306	1	Q8N5C6	Q8N5C6	S1 RNA-binding domain-containing protein 1	SRBD1	Homo sapiens	111.776	11	11	1054443	149491
2307	1	Q8NBF6	Q8NBF6	Late secretory pathway protein AVL9 homolog	AVL9	Homo sapiens	71.946	11	11	2335604	1458512
2308	1	Q8NC56	Q8NC56	LEM domain-containing protein 2	LEMD2	Homo sapiens	56.975	11	11	7356502	1998214
2309	1	Q8NHP6	Q8NHP6	Motile sperm domain-containing protein 2	MOSPD2	Homo sapiens	59.746	11	11	884293	3244023
2310	1	Q8TB45	Q8TB45	DEP domain-containing mTOR-interacting protein	DEPTOR	Homo sapiens	46.293	11	11	2169157	98886

2311	1 Q8TD16	Q8TD16	Protein bicaudal D homolog 2	BICD2	Homo sapiens	93.535	14	11	1096513	1490142
2312	1 Q8TEW8	Q8TEW8	Partitioning defective 3 homolog B	PARD3B	Homo sapiens	132.495	11	11	934996	0
2313	1 Q8WUH2	Q8WUH2	Transforming growth factor-beta receptor-associated protein 1	TGFBRAP1	Homo sapiens	97.158	11	11	1695272	890025
2314	1 Q8WVM7	Q8WVM7	Cohesin subunit SA-1	STAG1	Homo sapiens	144.43	11	11	1507224	368048
2315	1 Q8WXF1	Q8WXF1	Paraspeckle component 1	PSPC1	Homo sapiens	58.742	11	11	2585360	1735897
2316	1 Q8WXF7	Q8WXF7	Atlastin-1	ATL1	Homo sapiens	63.546	12	11	4308456	100031
2317	1 Q92747	Q92747	Actin-related protein 2/3 complex subunit 1A	ARPC1A	Homo sapiens	41.57	11	11	17533492	2650241
2318	1 Q92925	Q92925	SWI/SNF-related matrix-associated actin-dependent regulator of chromatin subfamily D member 2	SMARCD2	Homo sapiens	58.921	13	11	3466314	1243973
2319	1 Q93074	Q93074	Mediator of RNA polymerase II transcription subunit 12	MED12	Homo sapiens	243.082	12	11	488046	220816
2320	1 Q969G3	Q969G3	SWI/SNF-related matrix-associated actin-dependent regulator of chromatin subfamily E member 1	SMARCE1	Homo sapiens	46.65	11	11	2598579	1344966
2321	1 Q969Z0	Q969Z0	FAST kinase domain-containing protein 4	TBRG4	Homo sapiens	70.738	11	11	2331460	165289
2322	1 Q96DM3	Q96DM3	Regulator of MON1-CCZ1 complex	RMC1	Homo sapiens	74.976	11	11	864465	572896
2323	1 Q96DR7	Q96DR7	Rho guanine nucleotide exchange factor 26	ARHGEF26	Homo sapiens	97.346	11	11	1115488	0
2324	1 Q96EP0	Q96EP0	E3 ubiquitin-protein ligase RNF31	RNF31	Homo sapiens	119.654	11	11	1986111	1055158
2325	1 Q96EY1	Q96EY1	DnaJ homolog subfamily A member 3, mitochondrial	DNAJA3	Homo sapiens	52.489	11	11	2931143	659312
2326	1 Q96F86	Q96F86	Enhancer of mRNA-decapping protein 3	EDC3	Homo sapiens	56.078	11	11	1206322	66590
2327	1 Q96GQ5	Q96GQ5	RUS family member 1	RUSF1	Homo sapiens	51.017	11	11	2514054	455038
2328	1 Q96GX9	Q96GX9	Methylthioribulose-1-phosphate dehydratase	APIP	Homo sapiens	27.125	11	11	2371545	688660
2329	1 Q96IJ6	Q96IJ6	Mannose-1-phosphate guanyltransferase alpha	GMPPA	Homo sapiens	46.29	11	11	4083400	2195153
2330	1 Q96J84	Q96J84	Kin of IRRE-like protein 1	KIRREL1	Homo sapiens	83.536	11	11	1254103	14274
2331	1 Q96JY6	Q96JY6	PDZ and LIM domain protein 2	PDLIM2	Homo sapiens	37.458	11	11	1610823	1493867
2332	1 Q96NA2	Q96NA2	Rab-interacting lysosomal protein	RILP	Homo sapiens	44.201	11	11	1281810	590873
2333	1 Q96PU8	Q96PU8	KH domain-containing RNA-binding protein QKI	QKI	Homo sapiens	37.67	11	11	1845010	1702045
2334	1 Q96RR4	Q96RR4	Calcium/calmodulin-dependent protein kinase kinase 2	CAMKK2	Homo sapiens	64.745	11	11	446483	2063283
2335	1 Q96S55	Q96S55	ATPase WRNIP1	WRNIP1	Homo sapiens	72.136	11	11	3381077	308180
2336	1 Q96ST2	Q96ST2	Protein IWS1 homolog	IWS1	Homo sapiens	91.956	11	11	1673604	899425
2337	1 Q96T23	Q96T23	Remodeling and spacing factor 1	RSF1	Homo sapiens	163.825	11	11	1471709	270076
2338	1 Q96T60	Q96T60	Bifunctional polynucleotide phosphatase/kinase	PNKP	Homo sapiens	57.074	11	11	1050520	408770
2339	1 Q99536	Q99536	Synaptic vesicle membrane protein VAT-1 homolog	VAT1	Homo sapiens	41.921	11	11	46261238	37761433
2340	1 Q99543	Q99543	DnaJ homolog subfamily C member 2	DNAJC2	Homo sapiens	71.997	11	11	1126769	62145
2341	1 Q99575	Q99575	Ribonucleases P/MRP protein subunit POP1	POP1	Homo sapiens	114.711	11	11	1563893	237291
2342	1 Q99623	Q99623	Prohibitin-2	PHB2	Homo sapiens	33.294	11	11	60202146	24751947
2343	1 Q99747	Q99747	Gamma-soluble NSF attachment protein	NAPG	Homo sapiens	34.746	11	11	6582824	3328638
2344	1 Q99873	Q99873	Protein arginine N-methyltransferase 1	PRMT1	Homo sapiens	42.462	13	11	8176544	6905319
2345	1 Q9BQ70	Q9BQ70	Transcription factor 25	TCF25	Homo sapiens	76.666	11	11	1865080	622988
2346	1 Q9BRZ2	Q9BRZ2	E3 ubiquitin-protein ligase TRIM56	TRIM56	Homo sapiens	81.487	11	11	1886772	354851
2347	1 Q9BTE3	Q9BTE3	Mini-chromosome maintenance complex-binding protein	MCMBP	Homo sapiens	72.984	11	11	2468867	1607711
2348	1 Q9BUP3	Q9BUP3	Oxidoreductase HTATIP2	HTATIP2	Homo sapiens	27.049	11	11	6716095	4301096
2349	1 Q9BV73	Q9BV73	Centrosome-associated protein CEP250	CEP250	Homo sapiens	281.141	11	11	300040	20973
2350	1 Q9BWM7	Q9BWM7	Sideroflexin-3	SFXN3	Homo sapiens	35.504	11	11	11979992	1311468
2351	1 Q9BWU0	Q9BWU0	Kanadaptin	SLC4A1AP	Homo sapiens	88.817	11	11	767726	622375
2352	1 Q9BXW7	Q9BXW7	Haloacid dehalogenase-like hydrolase domain-containing 5	HDHD5	Homo sapiens	46.323	11	11	3272369	625434
2353	1 Q9BZE1	Q9BZE1	39S ribosomal protein L37, mitochondrial	MRPL37	Homo sapiens	48.118	11	11	2200961	353997
2354	1 Q9H159	Q9H159	Cadherin-19	CDH19	Homo sapiens	87.004	11	11	4510311	0
2355	1 Q9H3G5	Q9H3G5	Probable serine carboxypeptidase CPVL	CPVL	Homo sapiens	54.163	11	11	4154196	1399679
2356	1 Q9H3Q1	Q9H3Q1	Cdc42 effector protein 4	CDC42EP4	Homo sapiens	37.98	11	11	2497826	473867

2357	1	Q9H7D7	Q9H7D7	WD repeat-containing protein 26	WDR26	Homo sapiens	72.125	11	11	1402337	991324
2358	1	Q9H8H0	Q9H8H0	Nucleolar protein 11	NOL11	Homo sapiens	81.125	11	11	1207336	187699
2359	1	Q9H9B4	Q9H9B4	Sideroflexin-1	SFXN1	Homo sapiens	35.62	11	11	9978373	2977339
2360	1	Q9H9S4	Q9H9S4	Calcium-binding protein 39-like	CAB39L	Homo sapiens	39.088	11	11	5099398	0
2361	1	Q9H9Y6	Q9H9Y6	DNA-directed RNA polymerase I subunit RPA2	POLR1B	Homo sapiens	128.232	11	11	723210	18118
2362	1	Q9HCD5	Q9HCD5	Nuclear receptor coactivator 5	NCOA5	Homo sapiens	65.537	11	11	2030080	868555
2363	1	Q9HCE0	Q9HCE0	Ectopic P granules protein 5 homolog	EPG5	Homo sapiens	292.486	11	11	254531	508547
2364	1	Q9HCU5	Q9HCU5	Prolactin regulatory element-binding protein	PREB	Homo sapiens	45.468	11	11	3795480	3245441
2365	1	Q9HD26	Q9HD26	Golgi-associated PDZ and coiled-coil motif-containing protein	GOPC	Homo sapiens	50.519	11	11	2663198	2073274
2366	1	Q9NQ48	Q9NQ48	Leucine zipper transcription factor-like protein 1	LZTFL1	Homo sapiens	34.591	11	11	3145480	255874
2367	1	Q9NQC7	Q9NQC7	Ubiquitin carboxyl-terminal hydrolase CYLD	CYLD	Homo sapiens	107.316	11	11	1201066	877935
2368	1	Q9NRK6	Q9NRK6	ATP-binding cassette sub-family B member 10, mitochondrial	ABCB10	Homo sapiens	79.149	11	11	1526579	643292
2369	1	Q9NSY1	Q9NSY1	BMP-2-inducible protein kinase	BMP2K	Homo sapiens	129.173	11	11	1320389	416018
2370	1	Q9NUV9	Q9NUV9	GTPase IMAP family member 4	GIMAP4	Homo sapiens	37.534	11	11	2553726	2939602
2371	1	Q9NVH0	Q9NVH0	Exonuclease 3'-5' domain-containing protein 2	EXD2	Homo sapiens	70.353	11	11	1194457	202127
2372	1	Q9NVH2	Q9NVH2	Integrator complex subunit 7	INTS7	Homo sapiens	106.835	11	11	721551	238856
2373	1	Q9NX40	Q9NX40	OCIA domain-containing protein 1	OCIAD1	Homo sapiens	27.625	11	11	2478189	1562290
2374	1	Q9NZT2	Q9NZT2	Opioid growth factor receptor	OGFR	Homo sapiens	73.326	11	11	1504937	1819563
2375	1	Q9P0V9	Q9P0V9	Septin-10	SEPTIN10	Homo sapiens	52.592	11	11	6759997	298238
2376	1	Q9P1Y5	Q9P1Y5	Calmodulin-regulated spectrin-associated protein 3	CAMSAP3	Homo sapiens	134.753	11	11	1031918	54863
2377	1	Q9P2K8	Q9P2K8	eIF-2-alpha kinase GCN2	EIF2AK4	Homo sapiens	186.912	11	11	776827	237627
2378	1	Q9P2W9	Q9P2W9	Syntaxin-18	STX18	Homo sapiens	38.673	11	11	1226735	692890
2379	1	Q9UBB4	Q9UBB4	Ataxin-10	ATXN10	Homo sapiens	53.491	11	11	3549212	856392
2380	1	Q9UGR2	Q9UGR2	Zinc finger CCCH domain-containing protein 7B	ZC3H7B	Homo sapiens	109.858	11	11	1130882	45608
2381	1	Q9UHD1	Q9UHD1	Cysteine and histidine-rich domain-containing protein 1	CHORDC1	Homo sapiens	37.489	11	11	2328235	2028135
2382	1	Q9UHL4	Q9UHL4	Dipeptidyl peptidase 2	DPP7	Homo sapiens	54.342	11	11	10121740	4266117
2383	1	Q9UHP3	Q9UHP3	Ubiquitin carboxyl-terminal hydrolase 25	USP25	Homo sapiens	122.218	11	11	954774	766056
2384	1	Q9UHQ9	Q9UHQ9	NADH-cytochrome b5 reductase 1	CYB5R1	Homo sapiens	34.092	11	11	3658550	2781718
2385	1	Q9UHR4	Q9UHR4	Brain-specific angiogenesis inhibitor 1-associated protein 2-like protein 1	BAIAP2L1	Homo sapiens	56.884	11	11	2170827	610192
2386	1	Q9UIW2	Q9UIW2	Plexin-A1	PLXNA1	Homo sapiens	211.069	18	11	863645	41643
2387	1	Q9UJV9	Q9UJV9	Probable ATP-dependent RNA helicase DDX41	DDX41	Homo sapiens	69.839	11	11	1549081	570659
2388	1	Q9UJX2	Q9UJX2	Cell division cycle protein 23 homolog	CDC23	Homo sapiens	68.834	11	11	1480622	283912
2389	1	Q9UJX3	Q9UJX3	Anaphase-promoting complex subunit 7	ANAPC7	Homo sapiens	63.133	11	11	1092208	544767
2390	1	Q9UKU7	Q9UKU7	Isobutyryl-CoA dehydrogenase, mitochondrial	ACAD8	Homo sapiens	45.071	11	11	2765756	263700
2391	1	Q9UKU9	Q9UKU9	Angiopoietin-related protein 2	ANGPTL2	Homo sapiens	57.104	12	11	3161392	693609
2392	1	Q9ULE0	Q9ULE0	Protein WWC3	WWC3	Homo sapiens	122.678	11	11	811983	320617
2393	1	Q9ULU4	Q9ULU4	MYND-type zinc finger-containing chromatin reader ZMYND8	ZMYND8	Homo sapiens	131.696	11	11	986519	196257
2394	1	Q9UMS4	Q9UMS4	Pre-mRNA-processing factor 19	PRPF19	Homo sapiens	55.18	11	11	10211263	5758933
2395	1	Q9UNF0	Q9UNF0	Protein kinase C and casein kinase substrate in neurons protein 2	PACSIN2	Homo sapiens	55.736	11	11	16405650	5568795
2396	1	Q9UNF1	Q9UNF1	Melanoma-associated antigen D2	MAGED2	Homo sapiens	64.954	12	11	1966626	324864
2397	1	Q9Y2G5	Q9Y2G5	GDP-fucose protein O-fucosyltransferase 2	POFUT2	Homo sapiens	49.976	11	11	1204719	345914
2398	1	Q9Y2R9	Q9Y2R9	28S ribosomal protein S7, mitochondrial	MRPS7	Homo sapiens	28.134	11	11	2712729	450817
2399	1	Q9Y2S7	Q9Y2S7	Polymerase delta-interacting protein 2	POLDIP2	Homo sapiens	42.032	11	11	2580996	621942
2400	1	Q9Y2T3	Q9Y2T3	Guanine deaminase	GDA	Homo sapiens	51.001	11	11	1273983	7304142
2401	1	Q9Y376	Q9Y376	Calcium-binding protein 39	CAB39	Homo sapiens	39.87	12	11	6987408	3417645
2402	1	Q9Y3T9	Q9Y3T9	Nucleolar complex protein 2 homolog	NOC2L	Homo sapiens	84.919	11	11	1203303	401681
2403	1	Q9Y4J8	Q9Y4J8	Dystrobrevin alpha	DTNA	Homo sapiens	83.901	15	11	5593087	0
2404	1	Q9Y5M8	Q9Y5M8	Signal recognition particle receptor subunit beta	SRPRB	Homo sapiens	29.702	11	11	5222864	2887896

2405	1	Q9Y5P4	Q9Y5P4	Ceramide transfer protein	CERT1	Homo sapiens	70.835	11	11	1674245	1910246
2406	1	Q9Y5Q8	Q9Y5Q8	General transcription factor 3C polypeptide 5	GTF3C5	Homo sapiens	59.572	11	11	1242163	1068701
2407	1	Q9Y5Q9	Q9Y5Q9	General transcription factor 3C polypeptide 3	GTF3C3	Homo sapiens	101.273	11	11	899995	261825
2408	1	Q9Y5X2	Q9Y5X2	Sorting nexin-8	SNX8	Homo sapiens	52.571	11	11	1538464	559332
2409	1	Q9Y616	Q9Y616	Interleukin-1 receptor-associated kinase 3	IRAK3	Homo sapiens	67.771	11	11	41828	1438181
2410	1	Q9Y680	Q9Y680	Peptidyl-prolyl cis-trans isomerase FKBP7	FKBP7	Homo sapiens	25.794	11	11	55447409	3145788
2411	1	Q9Y6Y0	Q9Y6Y0	Influenza virus NS1A-binding protein	IVNS1ABP	Homo sapiens	71.729	11	11	703726	1174332
2412	1	A0AV96	A0AV96	RNA-binding protein 47	RBM47	Homo sapiens	64.1	12	10	3107572	1628315
2413	1	A1IGU5	A1IGU5	Rho guanine nucleotide exchange factor 37	ARHGEF37	Homo sapiens	76.279	10	10	2084299	0
2414	1	A1L0T0	A1L0T0	2-hydroxyacyl-CoA lyase 2	ILVBL	Homo sapiens	67.868	10	10	3592990	733822
2415	1	A4D1S5	A4D1S5	Ras-related protein Rab-19	RAB19	Homo sapiens	24.4	11	10	1051961	2233415
2416	1	O00273	O00273	DNA fragmentation factor subunit alpha	DFFA	Homo sapiens	36.521	10	10	3453855	714656
2417	1	O00308	O00308	NEDD4-like E3 ubiquitin-protein ligase WWP2	WWP2	Homo sapiens	98.914	11	10	749497	486473
2418	1	O00442	O00442	RNA 3'-terminal phosphate cyclase	RTCA	Homo sapiens	39.335	10	10	2790952	2303015
2419	1	O14576	O14576	Cytoplasmic dynein 1 intermediate chain 1	DYNC1I1	Homo sapiens	72.956	10	10	1403568	0
2420	1	O14656	O14656	Torsin-1A	TOR1A	Homo sapiens	37.809	10	10	1652851	2202172
2421	1	O15050	O15050	TPR and ankyrin repeat-containing protein 1	TRANK1	Homo sapiens	336.227	10	10	1589210	268059
2422	1	O15084	O15084	Serine/threonine-protein phosphatase 6 regulatory ankyrin repeat subunit A	ANKRD28	Homo sapiens	112.967	10	10	359965	490282
2423	1	O15091	O15091	Mitochondrial ribonuclease P catalytic subunit	PRORP	Homo sapiens	67.316	10	10	529904	30168
2424	1	O15382	O15382	Branched-chain-amino-acid aminotransferase, mitochondrial	BCAT2	Homo sapiens	44.287	10	10	5326717	510472
2425	1	O43293	O43293	Death-associated protein kinase 3	DAPK3	Homo sapiens	52.535	11	10	2275656	217816
2426	1	O43399	O43399	Tumor protein D54	TPD52L2	Homo sapiens	22.237	10	10	16320084	9871135
2427	1	O43556	O43556	Epsilon-sarcoglycan	SGCE	Homo sapiens	49.85	10	10	3454161	0
2428	1	O43639	O43639	Cytoplasmic protein NCK2	NCK2	Homo sapiens	42.916	10	10	3885355	874698
2429	1	O43933	O43933	Peroxisomal ATPase PEX1	PEX1	Homo sapiens	142.87	10	10	891395	92823
2430	1	O60237	O60237	Protein phosphatase 1 regulatory subunit 12B	PPP1R12B	Homo sapiens	110.406	10	10	15400808	188088
2431	1	O60256	O60256	Phosphoribosyl pyrophosphate synthase-associated protein 2	PRPSAP2	Homo sapiens	40.924	10	10	3478567	1324547
2432	1	O60307	O60307	Microtubule-associated serine/threonine-protein kinase 3	MAST3	Homo sapiens	143.138	12	10	261081	769890
2433	1	O60318	O60318	Germinal-center associated nuclear protein	MCM3AP	Homo sapiens	218.405	10	10	856622	260698
2434	1	O60331	O60331	Phosphatidylinositol 4-phosphate 5-kinase type-1 gamma	PIP5K1C	Homo sapiens	73.26	11	10	2555294	131292
2435	1	O60476	O60476	Mannosyl-oligosaccharide 1,2-alpha-mannosidase IB	MAN1A2	Homo sapiens	73.007	10	10	959772	584412
2436	1	O60658	O60658	High affinity cAMP-specific and IBMX-insensitive 3',5'-cyclic phosphodiesterase 8A	PDE8A	Homo sapiens	93.305	10	10	532023	0
2437	1	O60861	O60861	Growth arrest-specific protein 7	GAS7	Homo sapiens	54.373	10	10	2438977	784717
2438	1	O60885	O60885	Bromodomain-containing protein 4	BRD4	Homo sapiens	152.22	13	10	2101975	998663
2439	1	O75094	O75094	Slit homolog 3 protein	SLIT3	Homo sapiens	167.717	10	10	990531	0
2440	1	O75151	O75151	Lysine-specific demethylase PHF2	PHF2	Homo sapiens	120.777	11	10	1728901	79578
2441	1	O75390	O75390	Citrate synthase, mitochondrial	CS	Homo sapiens	51.714	10	10	58415063	19378202
2442	1	O75396	O75396	Vesicle-trafficking protein SEC22b	SEC22B	Homo sapiens	24.74	10	10	6835805	4858529
2443	1	O75688	O75688	Protein phosphatase 1B	PPM1B	Homo sapiens	52.643	10	10	2443345	2705602
2444	1	O75822	O75822	Eukaryotic translation initiation factor 3 subunit J	EIF3J	Homo sapiens	29.063	10	10	6348220	1604423
2445	1	O75832	O75832	26S proteasome non-ATPase regulatory subunit 10	PSMD10	Homo sapiens	24.426	10	10	6311224	3568420
2446	1	O75843	O75843	AP-1 complex subunit gamma-like 2	AP1G2	Homo sapiens	87.117	10	10	1454685	2589979
2447	1	O75844	O75844	CAAX prenyl protease 1 homolog	ZMPSTE24	Homo sapiens	54.814	10	10	2891739	2210741
2448	1	O75886	O75886	Signal transducing adapter molecule 2	STAM2	Homo sapiens	58.166	11	10	4223557	2057084
2449	1	O76021	O76021	Ribosomal L1 domain-containing protein 1	RSL1D1	Homo sapiens	54.974	10	10	5307703	2033351
2450	1	O94919	O94919	Endonuclease domain-containing 1 protein	ENDOD1	Homo sapiens	55.015	10	10	6616733	1088906

2451	1	O94967	O94967	WD repeat-containing protein 47	WDR47	Homo sapiens	101.948	10	10	1040687	158382
2452	1	O95395	O95395	Beta-1,3-galactosyl-O-glycosyl-glycoprotein beta-1,6-N-acetylglucosaminyltransferase 3	GCNT3	Homo sapiens	50.862	10	10	593770	0
2453	1	O95453	O95453	Poly(A)-specific ribonuclease PARN	PARN	Homo sapiens	73.451	10	10	1384752	579374
2454	1	O95571	O95571	Persulfide dioxygenase ETHE1, mitochondrial	ETHE1	Homo sapiens	27.873	10	10	36509793	6966156
2455	1	O95630	O95630	STAM-binding protein	STAMBP	Homo sapiens	48.077	10	10	2570668	1185189
2456	1	O96013	O96013	Serine/threonine-protein kinase PAK 4	PAK4	Homo sapiens	64.073	10	10	2167182	217421
2457	1	P00492	P00492	Hypoxanthine-guanine phosphoribosyltransferase	HPRT1	Homo sapiens	24.579	10	10	6674853	7950442
2458	1	P00749	P00749	Urokinase-type plasminogen activator	PLAU	Homo sapiens	48.525	10	10	27537	1540309
2459	1	P01857	P01857	Immunoglobulin heavy constant gamma 1	IGHG1	Homo sapiens	36.105	18	10	1545485628	2859743884
2460	1	P02749	P02749	Beta-2-glycoprotein 1	APOH	Homo sapiens	38.3	11	10	38071228	73208256
2461	1	P02794	P02794	Ferritin heavy chain	FTH1	Homo sapiens	21.223	10	10	6426075	45867810
2462	1	P04070	P04070	Vitamin K-dependent protein C	PROC	Homo sapiens	52.072	10	10	408603	2012054
2463	1	P04150	P04150	Glucocorticoid receptor	NR3C1	Homo sapiens	85.66	10	10	999161	341651
2464	1	P05121	P05121	Plasminogen activator inhibitor 1	SERPINE1	Homo sapiens	45.06	10	10	83635	6996634
2465	1	P05154	P05154	Plasma serine protease inhibitor	SERPINA5	Homo sapiens	45.673	10	10	768691	3146846
2466	1	P06733	P06733	Alpha-enolase	ENO1	Homo sapiens	47.168	23	10	155851872	142267122
2467	1	P07197	P07197	Neurofilament medium polypeptide	NEFM	Homo sapiens	102.474	10	10	641363	0
2468	1	P07357	P07357	Complement component C8 alpha chain	C8A	Homo sapiens	65.165	10	10	4294802	21289634
2469	1	P07858	P07858	Cathepsin B	CTSB	Homo sapiens	37.821	10	10	19503271	46253743
2470	1	P07954	P07954	Fumarate hydratase, mitochondrial	FH	Homo sapiens	54.636	10	10	31537055	3483290
2471	1	P08183	P08183	ATP-dependent translocase ABCB1	ABCB1	Homo sapiens	141.479	13	10	2007680	0
2472	1	P08238	P08238	Heat shock protein HSP 90-beta	HSP90AB1	Homo sapiens	83.267	22	10	70806719	39960369
2473	1	P08571	P08571	Monocyte differentiation antigen CD14	CD14	Homo sapiens	40.076	10	10	3632575	4170003
2474	1	P09471	P09471	Guanine nucleotide-binding protein G(o) subunit alpha	GNAO1	Homo sapiens	40.048	10	10	18898691	0
2475	1	P09493	P09493	Tropomyosin alpha-1 chain	TPM1	Homo sapiens	32.708	13	10	801479145	3675511
2476	1	P09601	P09601	Heme oxygenase 1	HMOX1	Homo sapiens	32.818	10	10	2727391	9268894
2477	1	P09622	P09622	Dihydrolipoyl dehydrogenase, mitochondrial	DLD	Homo sapiens	54.177	10	10	29324233	4491456
2478	1	P10301	P10301	Ras-related protein R-Ras	RRAS	Homo sapiens	23.479	11	10	25710741	779893
2479	1	P11172	P11172	Uridine 5'-monophosphate synthase	UMPS	Homo sapiens	52.22	10	10	3611134	1775777
2480	1	P16591	P16591	Tyrosine-protein kinase Fer	FER	Homo sapiens	94.641	10	10	930365	63842
2481	1	P17677	P17677	Neuromodulin	GAP43	Homo sapiens	24.801	10	10	6610642	0
2482	1	P17936	P17936	Insulin-like growth factor-binding protein 3	IGFBP3	Homo sapiens	31.673	10	10	802259	10045534
2483	1	P18887	P18887	DNA repair protein XRCC1	XRCC1	Homo sapiens	69.496	10	10	1349460	856495
2484	1	P19022	P19022	Cadherin-2	CDH2	Homo sapiens	99.809	11	10	1971713	132371
2485	1	P19623	P19623	Spermidine synthase	SRM	Homo sapiens	33.824	10	10	5375200	3167423
2486	1	P19634	P19634	Sodium/hydrogen exchanger 1	SLC9A1	Homo sapiens	90.765	11	10	2160133	872213
2487	1	P19784	P19784	Casein kinase II subunit alpha'	CSNK2A2	Homo sapiens	41.213	10	10	3514125	1243557
2488	1	P20701	P20701	Integrin alpha-L	ITGAL	Homo sapiens	128.771	10	10	198510	3245090
2489	1	P20711	P20711	Aromatic-L-amino-acid decarboxylase	DDC	Homo sapiens	53.928	10	10	2245297	0
2490	1	P21291	P21291	Cysteine and glycine-rich protein 1	CSRP1	Homo sapiens	20.567	10	10	822737311	8980267
2491	1	P21580	P21580	Tumor necrosis factor alpha-induced protein 3	TNFAIP3	Homo sapiens	89.614	10	10	63078	955462
2492	1	P23219	P23219	Prostaglandin G/H synthase 1	PTGS1	Homo sapiens	68.688	10	10	14917410	1905759
2493	1	P24539	P24539	ATP synthase F(0) complex subunit B1, mitochondrial	ATP5PB	Homo sapiens	28.907	10	10	42543340	10548681
2494	1	P25786	P25786	Proteasome subunit alpha type-1	PSMA1	Homo sapiens	29.556	10	10	25584619	17423179
2495	1	P26022	P26022	Pentraxin-related protein PTX3	PTX3	Homo sapiens	41.976	10	10	2042077	14437375
2496	1	P26232	P26232	Catenin alpha-2	CTNNA2	Homo sapiens	105.315	10	10	1078413	55953
2497	1	P27448	P27448	MAP/microtubule affinity-regulating kinase 3	MARK3	Homo sapiens	84.429	12	10	838422	836608

2498	1	P28062	P28062	Proteasome subunit beta type-8	PSMB8	Homo sapiens	30.354	10	10	7437883	5401472
2499	1	P28370	P28370	Probable global transcription activator SNF2L1	SMARCA1	Homo sapiens	122.605	10	10	29581556	0
2500	1	P29466	P29466	Caspase-1	CASP1	Homo sapiens	45.159	12	10	4515906	7565440
2501	1	P29728	P29728	2'-5'-oligoadenylate synthase 2	OAS2	Homo sapiens	82.431	10	10	765802	3062664
2502	1	P29966	P29966	Myristoylated alanine-rich C-kinase substrate	MARCKS	Homo sapiens	31.554	10	10	97239796	31748502
2503	1	P30043	P30043	Flavin reductase (NADPH)	BLVRB	Homo sapiens	22.118	10	10	38640928	23835409
2504	1	P30044	P30044	Peroxiredoxin-5, mitochondrial	PRDX5	Homo sapiens	22.085	10	10	84374044	54304288
2505	1	P30419	P30419	Glycylpeptide N-tetradecanoyltransferase 1	NMT1	Homo sapiens	56.806	12	10	4595635	2198674
2506	1	P30613	P30613	Pyruvate kinase PKLR	PKLR	Homo sapiens	61.83	10	10	538535	673483
2507	1	P31153	P31153	S-adenosylmethionine synthase isoform type-2	MAT2A	Homo sapiens	43.663	11	10	10906130	4324145
2508	1	P31323	P31323	cAMP-dependent protein kinase type II-beta regulatory subunit	PRKAR2B	Homo sapiens	46.304	10	10	3679471	370100
2509	1	P31749	P31749	RAC-alpha serine/threonine-protein kinase	AKT1	Homo sapiens	55.688	13	10	1489770	592607
2510	1	P31751	P31751	RAC-beta serine/threonine-protein kinase	AKT2	Homo sapiens	55.769	10	10	1678714	1253585
2511	1	P31947	P31947	14-3-3 protein sigma	SFN	Homo sapiens	27.774	10	10	17829199	11615135
2512	1	P32856	P32856	Syntaxin-2	STX2	Homo sapiens	33.341	10	10	934186	18703
2513	1	P33897	P33897	ATP-binding cassette sub-family D member 1	ABCD1	Homo sapiens	82.935	11	10	1064695	741596
2514	1	P33908	P33908	Mannosyl-oligosaccharide 1,2-alpha-mannosidase IA	MAN1A1	Homo sapiens	72.969	10	10	1711192	557226
2515	1	P34896	P34896	Serine hydroxymethyltransferase, cytosolic	SHMT1	Homo sapiens	53.083	10	10	3693154	1636345
2516	1	P35236	P35236	Tyrosine-protein phosphatase non-receptor type 7	PTPN7	Homo sapiens	40.53	10	10	284938	2986048
2517	1	P35659	P35659	Protein DEK	DEK	Homo sapiens	42.675	10	10	7800098	5686562
2518	1	P37802	P37802	Transgelin-2	TAGLN2	Homo sapiens	22.391	11	10	64464100	22897705
2519	1	P38117	P38117	Electron transfer flavoprotein subunit beta	ETFB	Homo sapiens	27.842	10	10	34614402	7788663
2520	1	P39656	P39656	Dolichyl-diphosphooligosaccharide--protein glycosyltransferase 48 kDa subunit	DDOST	Homo sapiens	50.801	10	10	30011216	18817982
2521	1	P40925	P40925	Malate dehydrogenase, cytoplasmic	MDH1	Homo sapiens	36.427	10	10	119892360	23515197
2522	1	P46736	P46736	Lys-63-specific deubiquitinase BRCC36	BRCC3	Homo sapiens	36.071	10	10	2890408	803571
2523	1	P46781	P46781	40S ribosomal protein S9	RPS9	Homo sapiens	22.592	10	10	27338104	8666179
2524	1	P48047	P48047	ATP synthase subunit O, mitochondrial	ATP5PO	Homo sapiens	23.276	10	10	71269300	13882317
2525	1	P48637	P48637	Glutathione synthetase	GSS	Homo sapiens	52.387	10	10	6446996	1764454
2526	1	P48723	P48723	Heat shock 70 kDa protein 13	HSPA13	Homo sapiens	51.925	10	10	1604079	688830
2527	1	P48736	P48736	Phosphatidylinositol 4,5-bisphosphate 3-kinase catalytic subunit gamma isoform	PIK3CG	Homo sapiens	126.454	10	10	40418	1178752
2528	1	P49137	P49137	MAP kinase-activated protein kinase 2	MAPKAPK2	Homo sapiens	45.569	12	10	1476489	2074109
2529	1	P49590	P49590	Histidine--tRNA ligase, mitochondrial	HARS2	Homo sapiens	56.887	10	10	1863366	359408
2530	1	P49757	P49757	Protein numb homolog	NUMB	Homo sapiens	70.804	10	10	3020010	3005177
2531	1	P49902	P49902	Cytosolic purine 5'-nucleotidase	NT5C2	Homo sapiens	64.972	10	10	2863535	3833729
2532	1	P50336	P50336	Protoporphyrinogen oxidase	PPOX	Homo sapiens	50.764	10	10	1721469	666587
2533	1	P50402	P50402	Emerin	EMD	Homo sapiens	28.994	10	10	12990701	4107052
2534	1	P51114	P51114	RNA-binding protein FXR1	FXR1	Homo sapiens	69.723	10	10	2848219	2480140
2535	1	P51398	P51398	28S ribosomal protein S29, mitochondrial	DAP3	Homo sapiens	45.566	10	10	4251230	869995
2536	1	P51572	P51572	B-cell receptor-associated protein 31	BCAP31	Homo sapiens	27.99	10	10	21049247	15352603
2537	1	P51784	P51784	Ubiquitin carboxyl-terminal hydrolase 11	USP11	Homo sapiens	109.817	10	10	709164	25740
2538	1	P52597	P52597	Heterogeneous nuclear ribonucleoprotein F	HNRNPF	Homo sapiens	45.673	10	10	10643101	5992494
2539	1	P53602	P53602	Diphosphomevalonate decarboxylase	MVD	Homo sapiens	43.404	10	10	3009328	876948
2540	1	P55287	P55287	Cadherin-11	CDH11	Homo sapiens	87.967	11	10	1492155	71307
2541	1	P55809	P55809	Succinyl-CoA:3-ketoacid coenzyme A transferase 1, mitochondrial	OXCT1	Homo sapiens	56.156	11	10	11700064	1321324
2542	1	P60660	P60660	Myosin light polypeptide 6	MYL6	Homo sapiens	16.93	12	10	1020519633	87137651
2543	1	P61981	P61981	14-3-3 protein gamma	YWHAG	Homo sapiens	28.302	10	10	58777366	24910363

2544	1	P62330	P62330	ADP-ribosylation factor 6	ARF6	Homo sapiens	20.08	10	10	8503560	3681791
2545	1	P78316	P78316	Nucleolar protein 14	NOP14	Homo sapiens	97.67	10	10	889628	76104
2546	1	P78406	P78406	mRNA export factor RAE1	RAE1	Homo sapiens	40.967	10	10	2661209	1762039
2547	1	P78524	P78524	DENN domain-containing protein 2B	DENND2B	Homo sapiens	126.483	11	10	2192724	106902
2548	1	P78536	P78536	Disintegrin and metalloproteinase domain-containing protein 17	ADAM17	Homo sapiens	93.022	10	10	893301	918885
2549	1	P80188	P80188	Neutrophil gelatinase-associated lipocalin	LCN2	Homo sapiens	22.589	10	10	4108832	112943107
2550	1	P80217	P80217	Interferon-induced 35 kDa protein	IFI35	Homo sapiens	31.545	10	10	3602641	2750614
2551	1	P80723	P80723	Brain acid soluble protein 1	BASP1	Homo sapiens	22.693	10	10	13648649	67158788
2552	1	P82650	P82650	28S ribosomal protein S22, mitochondrial	MRPS22	Homo sapiens	41.282	10	10	3564314	656617
2553	1	P82663	P82663	28S ribosomal protein S25, mitochondrial	MRPS25	Homo sapiens	20.116	10	10	2368261	224155
2554	2	P86790;P86791	P86790	Vacuolar fusion protein CCZ1 homolog B	CCZ1B	Homo sapiens	55.867	10	10	865395	566604
2555	1	Q00688	Q00688	Peptidyl-prolyl cis-trans isomerase FKBP3	FKBP3	Homo sapiens	25.175	10	10	18487360	6221845
2556	1	Q01974	Q01974	Tyrosine-protein kinase transmembrane receptor ROR2	ROR2	Homo sapiens	104.758	11	10	1651153	34126
2557	1	Q02127	Q02127	Dihydroorotate dehydrogenase (quinone), mitochondrial	DHODH	Homo sapiens	42.866	10	10	1624000	831215
2558	1	Q02338	Q02338	D-beta-hydroxybutyrate dehydrogenase, mitochondrial	BDH1	Homo sapiens	38.159	10	10	15915044	2277348
2559	1	Q04656	Q04656	Copper-transporting ATPase 1	ATP7A	Homo sapiens	163.376	10	10	1074537	260307
2560	1	Q04917	Q04917	14-3-3 protein eta	YWHAH	Homo sapiens	28.216	10	10	26628968	15043982
2561	1	Q06830	Q06830	Peroxiredoxin-1	PRDX1	Homo sapiens	22.111	11	10	148869833	84548798
2562	1	Q08289	Q08289	Voltage-dependent L-type calcium channel subunit beta-2	CACNB2	Homo sapiens	73.583	10	10	633402	0
2563	1	Q08495	Q08495	Dematin	DMTN	Homo sapiens	45.516	10	10	3259300	2203157
2564	1	Q0VDF9	Q0VDF9	Heat shock 70 kDa protein 14	HSPA14	Homo sapiens	54.796	10	10	1041665	742345
2565	1	Q10472	Q10472	Polypeptide N-acetylgalactosaminyltransferase 1	GALNT1	Homo sapiens	64.217	12	10	2847818	2519022
2566	1	Q11206	Q11206	CMP-N-acetylneuraminate-beta-galactosamide-alpha-2,3-sialyltransferase 4	ST3GAL4	Homo sapiens	38.046	10	10	3052769	77957
2567	1	Q13011	Q13011	Delta(3,5)-Delta(2,4)-dienoyl-CoA isomerase, mitochondrial	ECH1	Homo sapiens	35.816	10	10	26223462	5358937
2568	1	Q13033	Q13033	Striatin-3	STRN3	Homo sapiens	87.21	10	10	3855522	1235545
2569	1	Q13137	Q13137	Calcium-binding and coiled-coil domain-containing protein 2	CALCOCO2	Homo sapiens	52.254	10	10	645350	449312
2570	1	Q13162	Q13162	Peroxiredoxin-4	PRDX4	Homo sapiens	30.538	10	10	16216758	6810300
2571	1	Q13330	Q13330	Metastasis-associated protein MTA1	MTA1	Homo sapiens	80.788	10	10	1365358	115340
2572	1	Q13363	Q13363	C-terminal-binding protein 1	CTBP1	Homo sapiens	47.535	12	10	2321231	551850
2573	1	Q13395	Q13395	Probable methyltransferase TARBP1	TARBP1	Homo sapiens	181.678	10	10	470262	218479
2574	1	Q13480	Q13480	GRB2-associated-binding protein 1	GAB1	Homo sapiens	76.618	10	10	2194481	0
2575	1	Q13627	Q13627	Dual specificity tyrosine-phosphorylation-regulated kinase 1A	DYRK1A	Homo sapiens	85.586	12	10	783415	1116589
2576	1	Q13769	Q13769	THO complex subunit 5 homolog	THOC5	Homo sapiens	78.508	10	10	6060924	729642
2577	1	Q13936	Q13936	Voltage-dependent L-type calcium channel subunit alpha-1C	CACNA1C	Homo sapiens	248.983	12	10	982682	0
2578	1	Q14240	Q14240	Eukaryotic initiation factor 4A-II	EIF4A2	Homo sapiens	46.403	10	10	24798230	4061926
2579	1	Q14244	Q14244	Ensconsin	MAP7	Homo sapiens	84.054	10	10	2834095	186619
2580	1	Q14432	Q14432	cGMP-inhibited 3',5'-cyclic phosphodiesterase 3A	PDE3A	Homo sapiens	124.978	10	10	2207914	62175
2581	1	Q14498	Q14498	RNA-binding protein 39	RBM39	Homo sapiens	59.379	11	10	5835453	2708112
2582	1	Q14667	Q14667	Bridge-like lipid transfer protein family member 2	BLTP2	Homo sapiens	253.703	10	10	519107	210581
2583	1	Q14676	Q14676	Mediator of DNA damage checkpoint protein 1	MDC1	Homo sapiens	226.672	10	10	827881	136688
2584	1	Q14678	Q14678	KN motif and ankyrin repeat domain-containing protein 1	KANK1	Homo sapiens	147.292	10	10	1121011	0
2585	1	Q14684	Q14684	Ribosomal RNA processing protein 1 homolog B	RRP1B	Homo sapiens	84.428	10	10	976647	378256
2586	1	Q14738	Q14738	Serine/threonine-protein phosphatase 2A 56 kDa regulatory subunit delta isoform	PPP2R5D	Homo sapiens	69.993	14	10	6956646	1257154
2587	1	Q14914	Q14914	Prostaglandin reductase 1	PTGR1	Homo sapiens	35.869	10	10	14416888	3492343
2588	1	Q14997	Q14997	Proteasome activator complex subunit 4	PSME4	Homo sapiens	211.337	10	10	744176	527745
2589	1	Q15021	Q15021	Condensin complex subunit 1	NCAPD2	Homo sapiens	157.186	10	10	288331	296775

2590	1	Q15036	Q15036	Sorting nexin-17	SNX17	Homo sapiens	52.9	10	10	2892989	1879941
2591	1	Q15059	Q15059	Bromodomain-containing protein 3	BRD3	Homo sapiens	79.544	10	10	803045	90441
2592	1	Q15208	Q15208	Serine/threonine-protein kinase 38	STK38	Homo sapiens	54.19	10	10	2497476	2793402
2593	1	Q15269	Q15269	Periodic tryptophan protein 2 homolog	PWP2	Homo sapiens	102.452	10	10	722235	82750
2594	1	Q15283	Q15283	Ras GTPase-activating protein 2	RASA2	Homo sapiens	96.618	10	10	524040	1600495
2595	1	Q15628	Q15628	Tumor necrosis factor receptor type 1-associated DEATH domain protein	TRADD	Homo sapiens	34.246	10	10	1655471	1816764
2596	1	Q15942	Q15942	Zyxin	ZYX	Homo sapiens	61.277	10	10	66143290	23267645
2597	1	Q16270	Q16270	Insulin-like growth factor-binding protein 7	IGFBP7	Homo sapiens	29.13	10	10	2947681	4190151
2598	1	Q16775	Q16775	Hydroxyacylglutathione hydrolase, mitochondrial	HAGH	Homo sapiens	33.805	10	10	10483217	5133812
2599	1	Q16831	Q16831	Uridine phosphorylase 1	UPP1	Homo sapiens	33.933	10	10	282587	4017123
2600	1	Q17RN3	Q17RN3	Protein FAM98C	FAM98C	Homo sapiens	37.328	10	10	1248002	73772
2601	1	Q2M1Z3	Q2M1Z3	Rho GTPase-activating protein 31	ARHGAP31	Homo sapiens	156.988	10	10	367854	129431
2602	1	Q53GL0	Q53GL0	Pleckstrin homology domain-containing family O member 1	PLEKHO1	Homo sapiens	46.234	10	10	951864	0
2603	1	Q53LP3	Q53LP3	Ankyrin repeat domain-containing protein SOWAHC	SOWAHC	Homo sapiens	55.673	10	10	2921224	0
2604	1	Q53QZ3	Q53QZ3	Rho GTPase-activating protein 15	ARHGAP15	Homo sapiens	54.545	10	10	62479	5440718
2605	1	Q5BKZ1	Q5BKZ1	DBIRD complex subunit ZNF326	ZNF326	Homo sapiens	65.653	10	10	2012253	811306
2606	1	Q5JTH9	Q5JTH9	RRP12-like protein	RRP12	Homo sapiens	143.703	10	10	1392903	291477
2607	1	Q5JWF2	Q5JWF2	Guanine nucleotide-binding protein G(s) subunit alpha isoforms XLas	GNAS	Homo sapiens	111.025	12	10	12003902	7447251
2608	1	Q5T8D3	Q5T8D3	Acyl-CoA-binding domain-containing protein 5	ACBD5	Homo sapiens	60.094	10	10	1244967	416425
2609	1	Q5TCZ1	Q5TCZ1	SH3 and PX domain-containing protein 2A	SH3PXD2A	Homo sapiens	125.292	10	10	1150445	28541
2610	1	Q5U651	Q5U651	Ras-interacting protein 1	RASIP1	Homo sapiens	103.46	10	10	935109	259451
2611	1	Q5VUB5	Q5VUB5	Protein FAM171A1	FAM171A1	Homo sapiens	97.855	11	10	812245	0
2612	1	Q5VWQ8	Q5VWQ8	Disabled homolog 2-interacting protein	DAB2IP	Homo sapiens	131.627	12	10	1429977	31057
2613	1	Q68CP9	Q68CP9	AT-rich interactive domain-containing protein 2	ARID2	Homo sapiens	197.392	10	10	793431	136972
2614	1	Q6IA86	Q6IA86	Elongator complex protein 2	ELP2	Homo sapiens	92.501	10	10	9272983	161332
2615	1	Q6IAN0	Q6IAN0	Dehydrogenase/reductase SDR family member 7B	DHRS7B	Homo sapiens	35.118	10	10	1989592	1033358
2616	1	Q6IQ22	Q6IQ22	Ras-related protein Rab-12	RAB12	Homo sapiens	27.248	10	10	2311736	713020
2617	1	Q6JBY9	Q6JBY9	CapZ-interacting protein	RCSD1	Homo sapiens	44.505	10	10	1544667	4454231
2618	1	Q6L8Q7	Q6L8Q7	2',5'-phosphodiesterase 12	PDE12	Homo sapiens	67.353	10	10	2538193	1339848
2619	1	Q6NZI2	Q6NZI2	Caveolae-associated protein 1	CAVIN1	Homo sapiens	43.477	10	10	170889453	2506667
2620	1	Q6P4E1	Q6P4E1	Protein GOLM2	GOLM2	Homo sapiens	49.498	10	10	3171237	3179613
2621	1	Q6P9B6	Q6P9B6	MTOR-associated protein MEAK7	MEAK7	Homo sapiens	50.996	10	10	2165625	906191
2622	1	Q6PIU2	Q6PIU2	Neutral cholesterol ester hydrolase 1	NCEH1	Homo sapiens	45.809	10	10	7755788	4252233
2623	1	Q6UWM9	Q6UWM9	UDP-glucuronosyltransferase 2A3	UGT2A3	Homo sapiens	60.252	10	10	3726988	0
2624	1	Q6UXV4	Q6UXV4	MICOS complex subunit MIC27	APOOL	Homo sapiens	29.158	10	10	9344536	423004
2625	1	Q6UY14	Q6UY14	ADAMTS-like protein 4	ADAMTSL4	Homo sapiens	116.545	10	10	35931702	495651
2626	1	Q6YN16	Q6YN16	Hydroxysteroid dehydrogenase-like protein 2	HSDL2	Homo sapiens	45.398	10	10	3991476	776566
2627	1	Q6ZUT6	Q6ZUT6	Coiled-coil domain-containing protein 9B	CCDC9B	Homo sapiens	57.325	10	10	2892158	0
2628	1	Q7L2H7	Q7L2H7	Eukaryotic translation initiation factor 3 subunit M	EIF3M	Homo sapiens	42.502	10	10	3667276	1685783
2629	1	Q7L5Y1	Q7L5Y1	Mitochondrial enolase superfamily member 1	ENOSF1	Homo sapiens	49.788	10	10	1413883	391446
2630	1	Q7LDG7	Q7LDG7	RAS guanyl-releasing protein 2	RASGRP2	Homo sapiens	69.249	10	10	327028	1851481
2631	1	Q7Z3K3	Q7Z3K3	Pogo transposable element with ZNF domain	POGZ	Homo sapiens	155.346	10	10	1436124	511330
2632	1	Q7Z6B7	Q7Z6B7	SLIT-ROBO Rho GTPase-activating protein 1	SRGAP1	Homo sapiens	124.267	12	10	504517	58698
2633	1	Q7Z6I6	Q7Z6I6	Rho GTPase-activating protein 30	ARHGAP30	Homo sapiens	118.585	11	10	383277	3266679
2634	1	Q7Z6L1	Q7Z6L1	Tectonin beta-propeller repeat-containing protein 1	TECPR1	Homo sapiens	129.697	10	10	549614	759014
2635	1	Q86V15	Q86V15	Zinc finger protein castor homolog 1	CASZ1	Homo sapiens	190.074	10	10	1461061	0
2636	1	Q8IWT6	Q8IWT6	Volume-regulated anion channel subunit LRRC8A	LRRC8A	Homo sapiens	94.2	11	10	943905	143834
2637	1	Q8IWU2	Q8IWU2	Serine/threonine-protein kinase LMTK2	LMTK2	Homo sapiens	164.903	10	10	732667	814216

2638	1	Q8IWW6	Q8IWW6	Rho GTPase-activating protein 12	ARHGAP12	Homo sapiens	96.256	10	10	2870583	317384
2639	1	Q8IWX8	Q8IWX8	Calcium homeostasis endoplasmic reticulum protein	CHERP	Homo sapiens	103.703	10	10	2141615	819413
2640	1	Q8IWZ3	Q8IWZ3	Ankyrin repeat and KH domain-containing protein 1	ANKHD1	Homo sapiens	269.462	10	10	1258090	623324
2641	1	Q8IX04	Q8IX04	Ubiquitin-conjugating enzyme E2 variant 3	UEVLD	Homo sapiens	52.267	10	10	2238116	486178
2642	1	Q8IXH7	Q8IXH7	Negative elongation factor C/D	NELFCD	Homo sapiens	66.248	10	10	1584161	383078
2643	1	Q8IXI2	Q8IXI2	Mitochondrial Rho GTPase 1	RHOT1	Homo sapiens	70.787	11	10	1979478	445378
2644	1	Q8IXT5	Q8IXT5	RNA-binding protein 12B	RBM12B	Homo sapiens	118.103	10	10	1140924	524377
2645	1	Q8IY63	Q8IY63	Angiomotin-like protein 1	AMOTL1	Homo sapiens	106.574	10	10	527212	0
2646	1	Q8IYJ3	Q8IYJ3	Synaptotagmin-like protein 1	SYTL1	Homo sapiens	61.856	10	10	349734	5243106
2647	1	Q8N0X7	Q8N0X7	Spartin	SPART	Homo sapiens	72.836	10	10	1485905	1116156
2648	1	Q8N2K0	Q8N2K0	Lysophosphatidylserine lipase ABHD12	ABHD12	Homo sapiens	45.096	10	10	1177671	1214098
2649	1	Q8N3V7	Q8N3V7	Synaptopodin	SYNPO	Homo sapiens	99.466	10	10	3296959	18452
2650	1	Q8N442	Q8N442	Translation factor GUF1, mitochondrial	GUF1	Homo sapiens	74.328	10	10	765248	89873
2651	1	Q8N6R0	Q8N6R0	eEF1A lysine and N-terminal methyltransferase	METTL13	Homo sapiens	78.768	10	10	1056306	421140
2652	1	Q8NB16	Q8NB16	Mixed lineage kinase domain-like protein	MLKL	Homo sapiens	54.48	10	10	586457	2153672
2653	1	Q8NCE2	Q8NCE2	Myotubularin-related protein 14	MTMR14	Homo sapiens	72.203	10	10	55856	785647
2654	1	Q8NE01	Q8NE01	Metal transporter CNNM3	CNNM3	Homo sapiens	76.121	11	10	1330104	173092
2655	1	Q8NEV8	Q8NEV8	Exophilin-5	EXPH5	Homo sapiens	222.525	10	10	511457	0
2656	1	Q8NFF5	Q8NFF5	FAD synthase	FLAD1	Homo sapiens	65.266	10	10	668909	105968
2657	1	Q8TAG9	Q8TAG9	Exocyst complex component 6	EXOC6	Homo sapiens	93.723	11	10	1129869	1495225
2658	1	Q8TDD1	Q8TDD1	ATP-dependent RNA helicase DDX54	DDX54	Homo sapiens	98.594	10	10	549573	119358
2659	1	Q8TDF6	Q8TDF6	RAS guanyl-releasing protein 4	RASGRP4	Homo sapiens	74.885	10	10	0	9691452
2660	1	Q8TEV9	Q8TEV9	Guanine nucleotide exchange protein SMCR8	SMCR8	Homo sapiens	105.023	10	10	137082	429031
2661	1	Q8WXA3	Q8WXA3	RUN and FYVE domain-containing protein 2	RUFY2	Homo sapiens	70.011	10	10	647044	53214
2662	1	Q8WXI9	Q8WXI9	Transcriptional repressor p66-beta	GATAD2B	Homo sapiens	65.262	10	10	2274697	178530
2663	1	Q8WYA6	Q8WYA6	Beta-catenin-like protein 1	CTNNBL1	Homo sapiens	65.173	10	10	1862621	666654
2664	1	Q92599	Q92599	Septin-8	SEPTIN8	Homo sapiens	55.756	10	10	6961323	498221
2665	1	Q92636	Q92636	Protein FAN	NSMAF	Homo sapiens	104.373	10	10	773869	241116
2666	1	Q92734	Q92734	Protein TFG	TFG	Homo sapiens	43.448	10	10	13058206	3370146
2667	1	Q92785	Q92785	Zinc finger protein ubi-d4	DPF2	Homo sapiens	44.154	10	10	1934227	688025
2668	1	Q92820	Q92820	Gamma-glutamyl hydrolase	GGH	Homo sapiens	35.965	10	10	5302965	5923587
2669	1	Q969G5	Q969G5	Caveolae-associated protein 3	CAVIN3	Homo sapiens	27.699	10	10	26941673	815520
2670	1	Q96C19	Q96C19	EF-hand domain-containing protein D2	EFHD2	Homo sapiens	26.698	12	10	18485560	18385476
2671	1	Q96D71	Q96D71	RalBP1-associated Eps domain-containing protein 1	REPS1	Homo sapiens	86.663	11	10	2125189	579181
2672	1	Q96EK5	Q96EK5	KIF-binding protein	KIFBP	Homo sapiens	71.815	10	10	2298707	630222
2673	1	Q96FZ2	Q96FZ2	Abasic site processing protein HMCES	HMCES	Homo sapiens	40.576	10	10	759033	80866
2674	1	Q96HS1	Q96HS1	Serine/threonine-protein phosphatase PGAM5, mitochondrial	PGAM5	Homo sapiens	32.002	10	10	3181152	2530587
2675	1	Q96I15	Q96I15	Selenocysteine lyase	SCLY	Homo sapiens	48.149	10	10	1321040	0
2676	1	Q96JJ7	Q96JJ7	Protein disulfide-isomerase TMX3	TMX3	Homo sapiens	51.871	10	10	4532129	3147185
2677	1	Q96KC8	Q96KC8	DnaJ homolog subfamily C member 1	DNAJC1	Homo sapiens	63.883	10	10	1081886	1221853
2678	1	Q96PC5	Q96PC5	Melanoma inhibitory activity protein 2	MIA2	Homo sapiens	159.838	25	10	2378837	1009371
2679	1	Q96SI9	Q96SI9	Spermatid perinuclear RNA-binding protein	STRBP	Homo sapiens	73.654	10	10	2269661	128656
2680	1	Q96SQ9	Q96SQ9	Cytochrome P450 2S1	CYP2S1	Homo sapiens	55.817	10	10	2895165	254530
2681	1	Q96TA2	Q96TA2	ATP-dependent zinc metalloprotease YME1L1	YME1L1	Homo sapiens	86.456	10	10	1320510	464681
2682	1	Q99614	Q99614	Tetratricopeptide repeat protein 1	TTC1	Homo sapiens	33.525	10	10	2472369	746578
2683	1	Q99685	Q99685	Monoglyceride lipase	MGLL	Homo sapiens	33.26	10	10	2529668	1351435
2684	1	Q99714	Q99714	3-hydroxyacyl-CoA dehydrogenase type-2	HSD17B10	Homo sapiens	26.923	10	10	14440584	3724120
2685	1	Q99795	Q99795	Cell surface A33 antigen	GPA33	Homo sapiens	35.631	10	10	40810406	380885

2686	1	Q99807	Q99807	5-demethoxyubiquinone hydroxylase, mitochondrial	COQ7	Homo sapiens	24.279	10	10	2699693	76792
2687	1	Q99986	Q99986	Serine/threonine-protein kinase VRK1	VRK1	Homo sapiens	45.479	10	10	1054664	4194451
2688	1	Q9BQE5	Q9BQE5	Apolipoprotein L2	APOL2	Homo sapiens	37.076	10	10	4072391	14924438
2689	1	Q9BRR6	Q9BRR6	ADP-dependent glucokinase	ADPGK	Homo sapiens	54.089	10	10	895688	2037112
2690	1	Q9BTX1	Q9BTX1	Nucleoporin NDC1	NDC1	Homo sapiens	76.304	10	10	1429577	699487
2691	1	Q9BVJ6	Q9BVJ6	U3 small nucleolar RNA-associated protein 14 homolog A	UTP14A	Homo sapiens	87.978	14	10	1015537	156799
2692	1	Q9BXW6	Q9BXW6	Oxysterol-binding protein-related protein 1	OSBPL1A	Homo sapiens	108.47	10	10	1658019	108768
2693	1	Q9BY43	Q9BY43	Charged multivesicular body protein 4a	CHMP4A	Homo sapiens	25.097	10	10	7045741	5873654
2694	1	Q9BY77	Q9BY77	Polymerase delta-interacting protein 3	POLDIP3	Homo sapiens	46.091	10	10	2095622	782205
2695	1	Q9BZV1	Q9BZV1	UBX domain-containing protein 6	UBXN6	Homo sapiens	49.756	10	10	3818154	936928
2696	1	Q9C0E8	Q9C0E8	Endoplasmic reticulum junction formation protein lunapark	LNPK	Homo sapiens	47.74	10	10	12272760	5486297
2697	1	Q9GZT8	Q9GZT8	NIF3-like protein 1	NIF3L1	Homo sapiens	41.97	10	10	2189432	445225
2698	1	Q9H0W9	Q9H0W9	Ester hydrolase C11orf54	C11orf54	Homo sapiens	35.114	10	10	3932603	895409
2699	1	Q9H2C0	Q9H2C0	Gigaxonin	GAN	Homo sapiens	67.64	10	10	1218309	172856
2700	1	Q9H3R2	Q9H3R2	Mucin-13	MUC13	Homo sapiens	54.605	10	10	3300708	80101
2701	1	Q9H488	Q9H488	GDP-fucose protein O-fucosyltransferase 1	POFUT1	Homo sapiens	43.953	10	10	4082609	1832560
2702	1	Q9H4G4	Q9H4G4	Golgi-associated plant pathogenesis-related protein 1	GLIPR2	Homo sapiens	17.217	10	10	22602478	13827183
2703	1	Q9H4L5	Q9H4L5	Oxysterol-binding protein-related protein 3	OSBPL3	Homo sapiens	101.224	10	10	436598	675105
2704	1	Q9H6Z4	Q9H6Z4	Ran-binding protein 3	RANBP3	Homo sapiens	60.21	10	10	4990588	1174470
2705	1	Q9H7C4	Q9H7C4	Syncoilin	SYNC	Homo sapiens	55.3	10	10	2357011	17317
2706	1	Q9H832	Q9H832	Ubiquitin-conjugating enzyme E2 Z	UBE2Z	Homo sapiens	38.209	10	10	2861974	1099302
2707	1	Q9H9G7	Q9H9G7	Protein argonaute-3	AGO3	Homo sapiens	97.359	11	10	1130389	0
2708	1	Q9HA64	Q9HA64	Ketosamine-3-kinase	FN3KRP	Homo sapiens	34.415	10	10	5505543	910125
2709	1	Q9HAT2	Q9HAT2	Sialate O-acetylesterase	SIAE	Homo sapiens	58.314	10	10	7895187	1071329
2710	1	Q9HB19	Q9HB19	Pleckstrin homology domain-containing family A member 2	PLEKHA2	Homo sapiens	47.257	10	10	1356272	1239407
2711	1	Q9HB71	Q9HB71	Calcyclin-binding protein	CACYBP	Homo sapiens	26.21	10	10	6426837	4366620
2712	1	Q9HC38	Q9HC38	Glyoxalase domain-containing protein 4	GLOD4	Homo sapiens	34.794	10	10	27142335	6646016
2713	1	Q9HCJ6	Q9HCJ6	Synaptic vesicle membrane protein VAT-1 homolog-like	VAT1L	Homo sapiens	45.901	10	10	2475734	24777
2714	1	Q9NNW7	Q9NNW7	Thioredoxin reductase 2, mitochondrial	TXNRD2	Homo sapiens	56.479	10	10	2408080	600850
2715	1	Q9NP72	Q9NP72	Ras-related protein Rab-18	RAB18	Homo sapiens	22.977	10	10	7172353	4563859
2716	1	Q9NQ94	Q9NQ94	APOBEC1 complementation factor	A1CF	Homo sapiens	65.203	10	10	1299259	115958
2717	1	Q9NR19	Q9NR19	Acetyl-coenzyme A synthetase, cytoplasmic	ACSS2	Homo sapiens	78.58	10	10	7510377	1464523
2718	1	Q9NR97	Q9NR97	Toll-like receptor 8	TLR8	Homo sapiens	119.829	10	10	20497	3609331
2719	1	Q9NSY0	Q9NSY0	Nuclear receptor-binding protein 2	NRBP2	Homo sapiens	57.804	10	10	1366191	0
2720	1	Q9NTX5	Q9NTX5	Ethylmalonyl-CoA decarboxylase	ECHDC1	Homo sapiens	33.697	10	10	12867637	2549581
2721	1	Q9NUL3	Q9NUL3	Double-stranded RNA-binding protein Staufen homolog 2	STAU2	Homo sapiens	62.608	10	10	1049612	251797
2722	1	Q9NW13	Q9NW13	RNA-binding protein 28	RBM28	Homo sapiens	85.738	10	10	1464425	370451
2723	1	Q9NX46	Q9NX46	ADP-ribosylhydrolase ARH3	ADPRS	Homo sapiens	38.947	10	10	3725512	1453464
2724	1	Q9NX57	Q9NX57	Ras-related protein Rab-20	RAB20	Homo sapiens	26.276	10	10	851485	649273
2725	1	Q9NX58	Q9NX58	Cell growth-regulating nucleolar protein	LYAR	Homo sapiens	43.635	10	10	2255234	834515
2726	1	Q9NZN3	Q9NZN3	EH domain-containing protein 3	EHD3	Homo sapiens	60.887	10	10	1476941	297270
2727	1	Q9P0J0	Q9P0J0	NADH dehydrogenase [ubiquinone] 1 alpha subcomplex subunit 13	NDUFA13	Homo sapiens	16.699	10	10	13891435	3444340
2728	1	Q9P2E3	Q9P2E3	NFX1-type zinc finger-containing protein 1	ZNFX1	Homo sapiens	220.231	10	10	771900	91811
2729	1	Q9P2K5	Q9P2K5	Myelin expression factor 2	MYEF2	Homo sapiens	64.124	10	10	3162385	110623
2730	1	Q9UK55	Q9UK55	Protein Z-dependent protease inhibitor	SERPINA10	Homo sapiens	50.707	11	10	747557	5656652
2731	1	Q9UKL0	Q9UKL0	REST corepressor 1	RCOR1	Homo sapiens	53.328	12	10	1149945	338309
2732	1	Q9UL25	Q9UL25	Ras-related protein Rab-21	RAB21	Homo sapiens	24.346	10	10	6215127	7669594
2733	1	Q9UL46	Q9UL46	Proteasome activator complex subunit 2	PSME2	Homo sapiens	27.399	10	10	36670835	31331591

2734	1	Q9ULD2	Q9ULD2	Microtubule-associated tumor suppressor 1	MTUS1	Homo sapiens	141.399	10	10	769799	0
2735	1	Q9ULU8	Q9ULU8	Calcium-dependent secretion activator 1	CADPS	Homo sapiens	152.786	14	10	815665	0
2736	1	Q9ULV3	Q9ULV3	Cip1-interacting zinc finger protein	CIZ1	Homo sapiens	100.046	10	10	729520	457069
2737	1	Q9UN36	Q9UN36	Protein NDRG2	NDRG2	Homo sapiens	40.799	10	10	10064207	3255986
2738	1	Q9UNE7	Q9UNE7	E3 ubiquitin-protein ligase CHIP	STUB1	Homo sapiens	34.854	10	10	1720114	143432
2739	1	Q9UNH6	Q9UNH6	Sorting nexin-7	SNX7	Homo sapiens	45.303	10	10	2711027	121530
2740	1	Q9UNX4	Q9UNX4	WD repeat-containing protein 3	WDR3	Homo sapiens	106.1	10	10	418875	104767
2741	1	Q9UPM8	Q9UPM8	AP-4 complex subunit epsilon-1	AP4E1	Homo sapiens	127.292	10	10	514541	240262
2742	1	Q9UQR1	Q9UQR1	Zinc finger protein 148	ZNF148	Homo sapiens	88.977	10	10	1316062	711091
2743	1	Q9Y2D4	Q9Y2D4	Exocyst complex component 6B	EXOC6B	Homo sapiens	94.201	10	10	2462006	188889
2744	1	Q9Y2I8	Q9Y2I8	WD repeat-containing protein 37	WDR37	Homo sapiens	54.665	10	10	741130	1531657
2745	1	Q9Y2Q3	Q9Y2Q3	Glutathione S-transferase kappa 1	GSTK1	Homo sapiens	25.495	10	10	19693123	9107132
2746	1	Q9Y4W2	Q9Y4W2	Ribosomal biogenesis protein LAS1L	LAS1L	Homo sapiens	83.067	10	10	1753432	669290
2747	1	Q9Y5A7	Q9Y5A7	NEDD8 ultimate buster 1	NUB1	Homo sapiens	70.54	10	10	3585459	332928
2748	1	Q9Y5B0	Q9Y5B0	RNA polymerase II subunit A C-terminal domain phosphatase	CTDP1	Homo sapiens	104.399	10	10	2249077	753931
2749	1	Q9Y608	Q9Y608	Leucine-rich repeat flightless-interacting protein 2	LRRFIP2	Homo sapiens	82.173	10	10	3931846	1629713
2750	1	Q9Y697	Q9Y697	Cysteine desulfurase	NFS1	Homo sapiens	50.197	10	10	3392289	463568
2751	1	Q9Y6M9	Q9Y6M9	NADH dehydrogenase [ubiquinone] 1 beta subcomplex subunit 9	NDUFB9	Homo sapiens	21.831	10	10	12310613	3240602
2752	1	Q9Y6R1	Q9Y6R1	Electrogenic sodium bicarbonate cotransporter 1	SLC4A4	Homo sapiens	121.461	11	10	3013950	0
2753	1	A0JNW5	A0JNW5	Bridge-like lipid transfer protein family member 3B	BLTP3B	Homo sapiens	164.202	9	9	451459	431145
2754	1	A3KN83	A3KN83	Protein strawberry notch homolog 1	SBNO1	Homo sapiens	154.316	13	9	383251	551645
2755	1	A4D1E9	A4D1E9	GTP-binding protein 10	GTPBP10	Homo sapiens	42.934	9	9	808151	0
2756	1	A8MXV4	A8MXV4	Acyl-coenzyme A diphosphatase NUDT19	NUDT19	Homo sapiens	42.232	9	9	2271784	814661
2757	1	B2RTY4	B2RTY4	Unconventional myosin-IXa	MYO9A	Homo sapiens	292.712	9	9	377064	0
2758	1	O00154	O00154	Cytosolic acyl coenzyme A thioester hydrolase	ACOT7	Homo sapiens	41.792	9	9	15117715	4327919
2759	1	O00161	O00161	Synaptosomal-associated protein 23	SNAP23	Homo sapiens	23.354	9	9	3700133	6600855
2760	1	O00268	O00268	Transcription initiation factor TFIID subunit 4	TAF4	Homo sapiens	110.116	10	9	685331	0
2761	1	O00329	O00329	Phosphatidylinositol 4,5-bisphosphate 3-kinase catalytic subunit delta isoform	PIK3CD	Homo sapiens	119.479	9	9	121625	4029867
2762	1	O00408	O00408	cGMP-dependent 3',5'-cyclic phosphodiesterase	PDE2A	Homo sapiens	105.718	9	9	1011469	0
2763	1	O00418	O00418	Eukaryotic elongation factor 2 kinase	EEF2K	Homo sapiens	82.145	9	9	1283362	25780
2764	1	O00459	O00459	Phosphatidylinositol 3-kinase regulatory subunit beta	PIK3R2	Homo sapiens	81.544	9	9	451460	179917
2765	1	O00584	O00584	Ribonuclease T2	RNASET2	Homo sapiens	29.481	9	9	6121049	7662483
2766	1	O00763	O00763	Acetyl-CoA carboxylase 2	ACACB	Homo sapiens	276.545	9	9	277934	239002
2767	1	O14686	O14686	Histone-lysine N-methyltransferase 2D	KMT2D	Homo sapiens	593.401	9	9	294151	166185
2768	1	O14908	O14908	PDZ domain-containing protein GIPC1	GIPC1	Homo sapiens	36.05	10	9	3824971	424894
2769	1	O15118	O15118	NPC intracellular cholesterol transporter 1	NPC1	Homo sapiens	142.169	9	9	567838	1336526
2770	1	O15231	O15231	Zinc finger protein 185	ZNF185	Homo sapiens	73.527	9	9	284821	560499
2771	1	O15305	O15305	Phosphomannomutase 2	PMM2	Homo sapiens	28.082	9	9	8394419	5159523
2772	1	O15400	O15400	Syntaxin-7	STX7	Homo sapiens	29.815	9	9	9523256	8332817
2773	1	O15553	O15553	Pyrin	MEFV	Homo sapiens	86.443	9	9	0	2684440
2774	1	O43586	O43586	Proline-serine-threonine phosphatase-interacting protein 1	PSTPIP1	Homo sapiens	47.591	9	9	804430	3335089
2775	1	O43681	O43681	ATPase GET3	GET3	Homo sapiens	38.792	9	9	4681755	2810278
2776	1	O60240	O60240	Perilipin-1	PLIN1	Homo sapiens	55.99	9	9	3635332	0
2777	1	O60333	O60333	Kinesin-like protein KIF1B	KIF1B	Homo sapiens	204.481	18	9	638322	1026548
2778	1	O60687	O60687	Sushi repeat-containing protein SRPX2	SRPX2	Homo sapiens	52.971	9	9	604503	1057045
2779	1	O60762	O60762	Dolichol-phosphate mannosyltransferase subunit 1	DPM1	Homo sapiens	29.633	9	9	5139902	3415429
2780	1	O60784	O60784	Target of Myb1 membrane trafficking protein	TOM1	Homo sapiens	53.817	9	9	1887937	3313000

2781	1	O60890	O60890	Oligophrenin-1	OPHN1	Homo sapiens	91.643	9	9	874488	0
2782	1	O75113	O75113	NEDD4-binding protein 1	N4BP1	Homo sapiens	100.38	9	9	1590715	684673
2783	1	O75356	O75356	Nucleoside diphosphate phosphatase ENTPD5	ENTPD5	Homo sapiens	47.518	9	9	5688876	133847
2784	1	O75448	O75448	Mediator of RNA polymerase II transcription subunit 24	MED24	Homo sapiens	110.306	9	9	795744	481037
2785	1	O75521	O75521	Enoyl-CoA delta isomerase 2	ECI2	Homo sapiens	43.586	9	9	16879775	202929
2786	1	O75828	O75828	Carbonyl reductase [NADPH] 3	CBR3	Homo sapiens	30.851	9	9	3071091	378161
2787	1	O75953	O75953	DnaJ homolog subfamily B member 5	DNAJB5	Homo sapiens	39.133	9	9	2628073	0
2788	1	O94830	O94830	Phospholipase DDHD2	DDHD2	Homo sapiens	81.033	9	9	630156	18049
2789	1	O94905	O94905	Erlin-2	ERLIN2	Homo sapiens	37.841	11	9	20025028	10217498
2790	1	O94985	O94985	Calsyntenin-1	CLSTN1	Homo sapiens	109.794	9	9	300262	1739884
2791	1	O95218	O95218	Zinc finger Ran-binding domain-containing protein 2	ZRANB2	Homo sapiens	37.405	9	9	1358674	499162
2792	1	O95336	O95336	6-phosphogluconolactonase	PGLS	Homo sapiens	27.546	9	9	16883672	6418382
2793	1	O95685	O95685	Protein phosphatase 1 regulatory subunit 3D	PPP1R3D	Homo sapiens	32.559	9	9	441165	1855531
2794	1	O95810	O95810	Caveolae-associated protein 2	CAVIN2	Homo sapiens	47.173	9	9	32569469	786808
2795	1	O95825	O95825	Quinone oxidoreductase-like protein 1	CRYZL1	Homo sapiens	38.697	9	9	2153423	957746
2796	1	O95865	O95865	N(G),N(G)-dimethylarginine dimethylaminohydrolase 2	DDAH2	Homo sapiens	29.645	9	9	20317744	1025857
2797	1	O96011	O96011	Peroxisomal membrane protein 11B	PEX11B	Homo sapiens	28.432	9	9	5270809	318534
2798	1	P00451	P00451	Coagulation factor VIII	F8	Homo sapiens	267.015	9	9	0	610002
2799	1	P00748	P00748	Coagulation factor XII	F12	Homo sapiens	67.792	9	9	1073863	3084457
2800	1	P00918	P00918	Carbonic anhydrase 2	CA2	Homo sapiens	29.243	10	9	75578454	21362698
2801	1	P01019	P01019	Angiotensinogen	AGT	Homo sapiens	53.154	9	9	23115078	37538872
2802	1	P01860	P01860	Immunoglobulin heavy constant gamma 3	IGHG3	Homo sapiens	41.288	11	9	44655846	120348060
2803	1	P02452	P02452	Collagen alpha-1(I) chain	COL1A1	Homo sapiens	138.912	25	9	1177596097	123584799
2804	1	P02763	P02763	Alpha-1-acid glycoprotein 1	ORM1	Homo sapiens	23.539	12	9	486561296	753187300
2805	1	P02766	P02766	Transthyretin	TTR	Homo sapiens	15.885	9	9	13268595	26424985
2806	1	P04062	P04062	Lysosomal acid glucosylceramidase	GBA1	Homo sapiens	59.718	9	9	3280371	2352997
2807	1	P06132	P06132	Uroporphyrinogen decarboxylase	UROD	Homo sapiens	40.788	9	9	3892390	999833
2808	1	P06276	P06276	Cholinesterase	BCHE	Homo sapiens	68.42	9	9	2318276	724175
2809	1	P06396	P06396	Gelsolin	GSN	Homo sapiens	85.699	21	9	22878332	10883240
2810	1	P06400	P06400	Retinoblastoma-associated protein	RB1	Homo sapiens	106.162	9	9	732314	624754
2811	1	P07203	P07203	Glutathione peroxidase 1	GPX1	Homo sapiens	22.061	9	9	7361604	8532702
2812	1	P07451	P07451	Carbonic anhydrase 3	CA3	Homo sapiens	29.558	9	9	4112741	1103065
2813	1	P07737	P07737	Profilin-1	PFN1	Homo sapiens	15.054	12	9	260135530	210184604
2814	1	P07738	P07738	Bisphosphoglycerate mutase	BPGM	Homo sapiens	30.005	9	9	8153883	5264713
2815	1	P07902	P07902	Galactose-1-phosphate uridylyltransferase	GALT	Homo sapiens	43.363	9	9	3698872	1205584
2816	1	P09455	P09455	Retinol-binding protein 1	RBP1	Homo sapiens	15.85	9	9	3764656	119139
2817	1	P10321	P10321	HLA class I histocompatibility antigen, C alpha chain	HLA-C	Homo sapiens	40.649	9	9	2814629	6123938
2818	1	P11177	P11177	Pyruvate dehydrogenase E1 component subunit beta, mitochondrial	PDHB	Homo sapiens	39.232	9	9	19473869	3262859
2819	1	P11182	P11182	Lipoamide acyltransferase component of branched-chain alpha-keto acid dehydrogenase complex, mitochondrial	DBT	Homo sapiens	53.487	9	9	6725018	1193116
2820	1	P14384	P14384	Carboxypeptidase M	CPM	Homo sapiens	50.515	9	9	962189	2150091
2821	1	P16234	P16234	Platelet-derived growth factor receptor alpha	PDGFRA	Homo sapiens	122.669	9	9	736973	0
2822	1	P16298	P16298	Serine/threonine-protein phosphatase 2B catalytic subunit beta isoform	PPP3CB	Homo sapiens	59.025	15	9	1851232	404082
2823	1	P18283	P18283	Glutathione peroxidase 2	GPX2	Homo sapiens	21.925	9	9	4514712	0
2824	1	P18615	P18615	Negative elongation factor E	NELFE	Homo sapiens	43.239	9	9	1758509	540233
2825	1	P18754	P18754	Regulator of chromosome condensation	RCC1	Homo sapiens	44.969	9	9	2771504	2952013
2826	2	P20231;Q15661	P20231	Tryptase beta-2	TPSB2	Homo sapiens	30.515	13	9	22166708	0
2827	1	P20618	P20618	Proteasome subunit beta type-1	PSMB1	Homo sapiens	26.49	9	9	17793853	10345414

2828	1	P20774	P20774	Mimecan	OGN	Homo sapiens	33.921	9	9	181504468	678979
2829	1	P21695	P21695	Glycerol-3-phosphate dehydrogenase [NAD(+)], cytoplasmic	GPD1	Homo sapiens	37.568	9	9	1422240	2441818
2830	1	P22061	P22061	Protein-L-isoaspartate(D-aspartate) O-methyltransferase	PCMT1	Homo sapiens	24.635	9	9	4302515	1894104
2831	1	P22830	P22830	Ferrochelatase, mitochondrial	FECH	Homo sapiens	47.861	9	9	2465941	445620
2832	1	P22891	P22891	Vitamin K-dependent protein Z	PROZ	Homo sapiens	44.744	9	9	83140	5772168
2833	1	P23284	P23284	Peptidyl-prolyl cis-trans isomerase B	PPIB	Homo sapiens	23.741	9	9	109728977	88811375
2834	1	P23921	P23921	Ribonucleoside-diphosphate reductase large subunit	RRM1	Homo sapiens	90.073	9	9	1292112	928591
2835	1	P23946	P23946	Chymase	CMA1	Homo sapiens	27.325	9	9	12588100	0
2836	1	P25774	P25774	Cathepsin S	CTSS	Homo sapiens	37.493	9	9	5447673	7133752
2837	1	P26373	P26373	60S ribosomal protein L13	RPL13	Homo sapiens	24.263	9	9	27050371	7319814
2838	1	P26599	P26599	Polypyrimidine tract-binding protein 1	PTBP1	Homo sapiens	59.632	10	9	19097072	10510488
2839	1	P27216	P27216	Annexin A13	ANXA13	Homo sapiens	35.415	9	9	2814970	50225
2840	1	P27361	P27361	Mitogen-activated protein kinase 3	MAPK3	Homo sapiens	43.134	9	9	7078061	2538620
2841	1	P28066	P28066	Proteasome subunit alpha type-5	PSMA5	Homo sapiens	26.412	9	9	15228146	7208173
2842	1	P28676	P28676	Grancalcin	GCA	Homo sapiens	24.01	9	9	8306980	288390966
2843	1	P28827	P28827	Receptor-type tyrosine-protein phosphatase mu	PTPRM	Homo sapiens	163.686	10	9	1050946	29003
2844	1	P28907	P28907	ADP-ribosyl cyclase/cyclic ADP-ribose hydrolase 1	CD38	Homo sapiens	34.328	9	9	926351	897762
2845	1	P29372	P29372	DNA-3-methyladenine glycosylase	MPG	Homo sapiens	32.867	9	9	1083702	1104718
2846	1	P30084	P30084	Enoyl-CoA hydratase, mitochondrial	ECHS1	Homo sapiens	31.385	9	9	11581021	2457610
2847	1	P30086	P30086	Phosphatidylethanolamine-binding protein 1	PEBP1	Homo sapiens	21.056	9	9	107879489	21231719
2848	1	P30260	P30260	Cell division cycle protein 27 homolog	CDC27	Homo sapiens	91.87	9	9	1128841	258487
2849	1	P31942	P31942	Heterogeneous nuclear ribonucleoprotein H3	HNRNPH3	Homo sapiens	36.924	9	9	17814423	8193767
2850	1	P31946	P31946	14-3-3 protein beta/alpha	YWHAB	Homo sapiens	28.083	11	9	65717201	36724736
2851	1	P32455	P32455	Guanylate-binding protein 1	GBP1	Homo sapiens	67.93	11	9	6903996	7649129
2852	1	P34949	P34949	Mannose-6-phosphate isomerase	MPI	Homo sapiens	46.656	9	9	4542796	1723859
2853	1	P35568	P35568	Insulin receptor substrate 1	IRS1	Homo sapiens	131.592	9	9	586589	0
2854	1	P36405	P36405	ADP-ribosylation factor-like protein 3	ARL3	Homo sapiens	20.456	9	9	4221128	1506175
2855	1	P36952	P36952	Serpin B5	SERPINB5	Homo sapiens	42.1	9	9	2390877	687893
2856	1	P36957	P36957	Dihydrolipoyllysine-residue succinyltransferase component of 2-oxoglutarate dehydrogenase complex, mitochondrial	DLST	Homo sapiens	48.758	9	9	14580072	4092201
2857	1	P40937	P40937	Replication factor C subunit 5	RFC5	Homo sapiens	38.498	9	9	1523016	321705
2858	1	P42226	P42226	Signal transducer and activator of transcription 6	STAT6	Homo sapiens	94.134	9	9	4253799	1910367
2859	1	P42574	P42574	Caspase-3	CASP3	Homo sapiens	31.609	9	9	4611994	3073113
2860	1	P42768	P42768	Actin nucleation-promoting factor WAS	WAS	Homo sapiens	52.912	9	9	1198983	6648284
2861	1	P43897	P43897	Elongation factor Ts, mitochondrial	TSFM	Homo sapiens	35.393	9	9	1604280	472334
2862	1	P45954	P45954	Short/branched chain specific acyl-CoA dehydrogenase, mitochondrial	ACADSB	Homo sapiens	47.486	9	9	3428221	354404
2863	1	P47755	P47755	F-actin-capping protein subunit alpha-2	CAPZA2	Homo sapiens	32.949	9	9	32477074	6571832
2864	1	P49788	P49788	Retinoic acid receptor responder protein 1	RARRES1	Homo sapiens	33.285	9	9	757592	7639788
2865	1	P49848	P49848	Transcription initiation factor TFIID subunit 6	TAF6	Homo sapiens	72.671	9	9	649750	126584
2866	1	P49961	P49961	Ectonucleoside triphosphate diphosphohydrolase 1	ENTPD1	Homo sapiens	57.966	9	9	7460943	2123275
2867	1	P50443	P50443	Sulfate transporter	SLC26A2	Homo sapiens	81.661	9	9	6268342	179619
2868	1	P50749	P50749	Ras association domain-containing protein 2	RASSF2	Homo sapiens	37.789	10	9	973844	6295136
2869	1	P51648	P51648	Aldehyde dehydrogenase family 3 member A2	ALDH3A2	Homo sapiens	54.847	10	9	7799121	2612084
2870	1	P51884	P51884	Lumican	LUM	Homo sapiens	38.43	12	9	87723270	5704301
2871	1	P52564	P52564	Dual specificity mitogen-activated protein kinase kinase 6	MAP2K6	Homo sapiens	37.491	9	9	3766510	105613
2872	1	P52907	P52907	F-actin-capping protein subunit alpha-1	CAPZA1	Homo sapiens	32.924	11	9	31259638	21086089
2873	1	P53004	P53004	Biliverdin reductase A	BLVRA	Homo sapiens	33.428	9	9	11887951	10994119
2874	1	P53609	P53609	Geranylgeranyl transferase type-1 subunit beta	PGGT1B	Homo sapiens	42.369	9	9	2188192	1576408

2875	1	P53671	P53671	LIM domain kinase 2	LIMK2	Homo sapiens	72.235	9	9	1075358	1269386
2876	1	P56545	P56545	C-terminal-binding protein 2	CTBP2	Homo sapiens	48.947	9	9	2639140	701610
2877	1	P61018	P61018	Ras-related protein Rab-4B	RAB4B	Homo sapiens	23.588	10	9	1860720	2022934
2878	1	P61289	P61289	Proteasome activator complex subunit 3	PSME3	Homo sapiens	29.504	9	9	2671231	1477364
2879	1	P62081	P62081	40S ribosomal protein S7	RPS7	Homo sapiens	22.126	9	9	7989028	4739481
2880	1	P62269	P62269	40S ribosomal protein S18	RPS18	Homo sapiens	17.72	9	9	47347401	25576935
2881	1	P62805	P62805	Histone H4	H4C16	Homo sapiens	11.365	9	9	1258227965	1761477946
2882	1	P62906	P62906	60S ribosomal protein L10a	RPL10A	Homo sapiens	24.829	9	9	21241048	7128503
2883	1	P68871	P68871	Hemoglobin subunit beta	HBB	Homo sapiens	15.997	16	9	4580808309	5988893008
2884	1	P78318	P78318	Immunoglobulin-binding protein 1	IGBP1	Homo sapiens	39.222	11	9	2889097	751675
2885	1	P78332	P78332	RNA-binding protein 6	RBM6	Homo sapiens	128.644	9	9	769852	170360
2886	1	P82933	P82933	28S ribosomal protein S9, mitochondrial	MRPS9	Homo sapiens	45.834	9	9	1625048	73098
2887	1	Q00059	Q00059	Transcription factor A, mitochondrial	TFAM	Homo sapiens	29.097	9	9	7755379	1092028
2888	1	Q00403	Q00403	Transcription initiation factor IIB	GTF2B	Homo sapiens	34.83	9	9	1115120	635475
2889	1	Q00796	Q00796	Sorbitol dehydrogenase	SORD	Homo sapiens	38.324	9	9	4908180	1472333
2890	1	Q01085	Q01085	Nucleolysin TIAR	TIAL1	Homo sapiens	41.589	12	9	2789323	925649
2891	1	Q01581	Q01581	Hydroxymethylglutaryl-CoA synthase, cytoplasmic	HMGCS1	Homo sapiens	57.291	9	9	2736591	441747
2892	1	Q01804	Q01804	OTU domain-containing protein 4	OTUD4	Homo sapiens	124.046	9	9	691758	134491
2893	1	Q02156	Q02156	Protein kinase C epsilon type	PRKCE	Homo sapiens	83.675	9	9	715918	89473
2894	1	Q02318	Q02318	Sterol 26-hydroxylase, mitochondrial	CYP27A1	Homo sapiens	60.235	9	9	4151593	1126643
2895	1	Q06278	Q06278	Aldehyde oxidase	AOX1	Homo sapiens	147.92	9	9	434888	128339
2896	1	Q06330	Q06330	Recombining binding protein suppressor of hairless	RBPJ	Homo sapiens	55.636	10	9	5007383	2342349
2897	1	Q06481	Q06481	Amyloid beta precursor like protein 2	APLP2	Homo sapiens	86.956	9	9	713386	832373
2898	1	Q06546	Q06546	GA-binding protein alpha chain	GABPA	Homo sapiens	51.293	9	9	1852793	1028561
2899	1	Q07617	Q07617	Sperm-associated antigen 1	SPAG1	Homo sapiens	103.641	9	9	893638	894938
2900	1	Q07812	Q07812	Apoptosis regulator BAX	BAX	Homo sapiens	21.182	9	9	5135385	4678565
2901	1	Q08431	Q08431	Lactadherin	MFGE8	Homo sapiens	43.105	9	9	2877709	2422642
2902	1	Q09013	Q09013	Myotonin-protein kinase	DMPK	Homo sapiens	69.385	9	9	2850735	0
2903	1	Q12972	Q12972	Nuclear inhibitor of protein phosphatase 1	PPP1R8	Homo sapiens	38.478	9	9	2502695	288291
2904	1	Q12981	Q12981	Vesicle transport protein SEC20	BNIP1	Homo sapiens	26.128	9	9	2020430	900351
2905	1	Q13042	Q13042	Cell division cycle protein 16 homolog	CDC16	Homo sapiens	71.658	9	9	1213804	588433
2906	1	Q13057	Q13057	Bifunctional coenzyme A synthase	COASY	Homo sapiens	62.327	9	9	3415669	2563993
2907	1	Q13094	Q13094	Lymphocyte cytosolic protein 2	LCP2	Homo sapiens	60.188	9	9	693971	5087697
2908	1	Q13123	Q13123	Protein Red	IK	Homo sapiens	65.602	9	9	1568507	556561
2909	1	Q13188	Q13188	Serine/threonine-protein kinase 3	STK3	Homo sapiens	56.3	9	9	1326453	637253
2910	1	Q13277	Q13277	Syntaxin-3	STX3	Homo sapiens	33.154	9	9	1667542	3420738
2911	1	Q13310	Q13310	Polyadenylate-binding protein 4	PABPC4	Homo sapiens	70.782	10	9	5702496	3202889
2912	1	Q13356	Q13356	RING-type E3 ubiquitin-protein ligase PPIL2	PPIL2	Homo sapiens	58.826	9	9	729320	77717
2913	1	Q13449	Q13449	Limbic system-associated membrane protein	LSAMP	Homo sapiens	37.393	9	9	6628075	0
2914	1	Q13492	Q13492	Phosphatidylinositol-binding clathrin assembly protein	PICALM	Homo sapiens	70.755	10	9	3536034	1354034
2915	1	Q13505	Q13505	Metaxin-1	MTX1	Homo sapiens	51.463	9	9	4351341	2393082
2916	1	Q13613	Q13613	Myotubularin-related protein 1	MTMR1	Homo sapiens	74.678	10	9	1858324	892717
2917	1	Q13636	Q13636	Ras-related protein Rab-31	RAB31	Homo sapiens	21.57	11	9	2546987	13637061
2918	1	Q13867	Q13867	Bleomycin hydrolase	BLMH	Homo sapiens	52.563	9	9	4359500	2417548
2919	1	Q14145	Q14145	Kelch-like ECH-associated protein 1	KEAP1	Homo sapiens	69.667	9	9	1021319	1006595
2920	1	Q14151	Q14151	Scaffold attachment factor B2	SAFB2	Homo sapiens	107.473	9	9	1610427	518469
2921	1	Q14192	Q14192	Four and a half LIM domains protein 2	FHL2	Homo sapiens	32.191	9	9	1956180	420897
2922	1	Q14318	Q14318	Peptidyl-prolyl cis-trans isomerase FKBP8	FKBP8	Homo sapiens	44.561	9	9	646773	785611

2923	1	Q14527	Q14527	Helicase-like transcription factor	HLTF	Homo sapiens	113.931	9	9	315299	99886
2924	1	Q14699	Q14699	Raftlin	RFTN1	Homo sapiens	63.145	9	9	1372041	438956
2925	1	Q14739	Q14739	Delta(14)-sterol reductase LBR	LBR	Homo sapiens	70.702	9	9	2124355	4119387
2926	1	Q14767	Q14767	Latent-transforming growth factor beta-binding protein 2	LTBP2	Homo sapiens	195.055	9	9	1068056	299341
2927	1	Q15007	Q15007	Pre-mRNA-splicing regulator WTAP	WTAP	Homo sapiens	44.243	9	9	1375472	927902
2928	1	Q15291	Q15291	Retinoblastoma-binding protein 5	RBBP5	Homo sapiens	59.153	9	9	1185248	744387
2929	1	Q15404	Q15404	Ras suppressor protein 1	RSU1	Homo sapiens	31.541	9	9	91466283	5349426
2930	1	Q15645	Q15645	Pachytene checkpoint protein 2 homolog	TRIP13	Homo sapiens	48.553	9	9	297583	1026466
2931	1	Q16527	Q16527	Cysteine and glycine-rich protein 2	CSRP2	Homo sapiens	20.952	9	9	9181864	631776
2932	1	Q16537	Q16537	Serine/threonine-protein phosphatase 2A 56 kDa regulatory subunit epsilon isoform	PPP2R5E	Homo sapiens	54.7	9	9	3208388	1057105
2933	1	Q16539	Q16539	Mitogen-activated protein kinase 14	MAPK14	Homo sapiens	41.295	9	9	3405598	9117753
2934	1	Q16630	Q16630	Cleavage and polyadenylation specificity factor subunit 6	CPSF6	Homo sapiens	59.211	9	9	5753789	2613562
2935	1	Q16740	Q16740	ATP-dependent Clp protease proteolytic subunit, mitochondrial	CLPP	Homo sapiens	30.179	9	9	3855058	1412666
2936	1	Q16774	Q16774	Guanylate kinase	GUK1	Homo sapiens	21.726	9	9	3465491	2010426
2937	1	Q16819	Q16819	Meprin A subunit alpha	MEP1A	Homo sapiens	84.422	9	9	1613267	0
2938	1	Q16875	Q16875	6-phosphofructo-2-kinase/fructose-2,6-bisphosphatase 3	PFKFB3	Homo sapiens	59.609	9	9	348752	1805277
2939	1	Q4KMP7	Q4KMP7	TBC1 domain family member 10B	TBC1D10B	Homo sapiens	87.2	9	9	1835633	618741
2940	1	Q53GL7	Q53GL7	Protein mono-ADP-ribosyltransferase PARP10	PARP10	Homo sapiens	110	9	9	1232086	2587781
2941	1	Q53T59	Q53T59	HCLS1-binding protein 3	HS1BP3	Homo sapiens	42.78	9	9	4770996	2617417
2942	1	Q5JPH6	Q5JPH6	Probable glutamate--tRNA ligase, mitochondrial	EARS2	Homo sapiens	58.688	9	9	904452	41162
2943	1	Q5T6F2	Q5T6F2	Ubiquitin-associated protein 2	UBAP2	Homo sapiens	117.119	9	9	1308985	225954
2944	1	Q5TC84	Q5TC84	Opioid growth factor receptor-like protein 1	OGFRL1	Homo sapiens	51.251	9	9	178593	1072569
2945	1	Q5TCQ9	Q5TCQ9	Membrane-associated guanylate kinase, WW and PDZ domain-containing protein 3	MAGI3	Homo sapiens	162.949	9	9	609496	18201
2946	1	Q5TFE4	Q5TFE4	5'-nucleotidase domain-containing protein 1	NT5DC1	Homo sapiens	51.845	9	9	2994367	1016524
2947	1	Q5VSL9	Q5VSL9	Striatin-interacting protein 1	STRIP1	Homo sapiens	95.579	10	9	1394410	787330
2948	1	Q5VTL8	Q5VTL8	Pre-mRNA-splicing factor 38B	PRPF38B	Homo sapiens	64.468	9	9	1196801	647572
2949	1	Q5VWJ9	Q5VWJ9	Sorting nexin-30	SNX30	Homo sapiens	49.677	9	9	750800	323224
2950	1	Q5W0V3	Q5W0V3	FHF complex subunit HOOK interacting protein 2A	FHIP2A	Homo sapiens	86.56	9	9	1648684	2719514
2951	1	Q676U5	Q676U5	Autophagy-related protein 16-1	ATG16L1	Homo sapiens	68.265	9	9	1302309	756585
2952	1	Q69YN2	Q69YN2	CWF19-like protein 1	CWF19L1	Homo sapiens	60.62	9	9	915137	806529
2953	1	Q6DKJ4	Q6DKJ4	Nucleoredoxin	NXN	Homo sapiens	48.393	9	9	4879220	675337
2954	1	Q6FI81	Q6FI81	Anamorsin	CIAPIN1	Homo sapiens	33.581	9	9	5146121	1846223
2955	1	Q6ICG6	Q6ICG6	Uncharacterized protein KIAA0930	KIAA0930	Homo sapiens	45.793	9	9	928526	1208824
2956	1	Q6IQ26	Q6IQ26	DENN domain-containing protein 5A	DENND5A	Homo sapiens	147.098	9	9	395317	175997
2957	1	Q6P158	Q6P158	Putative ATP-dependent RNA helicase DHX57	DHX57	Homo sapiens	155.606	9	9	544273	86984
2958	1	Q6PCB7	Q6PCB7	Long-chain fatty acid transport protein 1	SLC27A1	Homo sapiens	71.108	9	9	1223055	845091
2959	1	Q6Q788	Q6Q788	Apolipoprotein A-V	APOA5	Homo sapiens	41.215	9	9	0	1275284
2960	1	Q6R327	Q6R327	Rapamycin-insensitive companion of mTOR	RICTOR	Homo sapiens	192.221	9	9	763205	197947
2961	1	Q6VN20	Q6VN20	Ran-binding protein 10	RANBP10	Homo sapiens	67.257	9	9	1525366	486130
2962	1	Q6YP21	Q6YP21	Kynurenine--oxoglutarate transaminase 3	KYAT3	Homo sapiens	51.4	9	9	1702609	292500
2963	1	Q71RC2	Q71RC2	La-related protein 4	LARP4	Homo sapiens	80.597	9	9	529071	868827
2964	1	Q75QN2	Q75QN2	Integrator complex subunit 8	INTS8	Homo sapiens	113.09	9	9	285087	321131
2965	1	Q765P7	Q765P7	Protein MTSS 2	MTSS2	Homo sapiens	79.932	9	9	939954	0
2966	1	Q7L5D6	Q7L5D6	Golgi to ER traffic protein 4 homolog	GET4	Homo sapiens	36.504	9	9	1836773	1221113
2967	1	Q7Z401	Q7Z401	C-myc promoter-binding protein	DENND4A	Homo sapiens	209.247	9	9	291405	351712
2968	1	Q7Z6B0	Q7Z6B0	Coiled-coil domain-containing protein 91	CCDC91	Homo sapiens	49.972	9	9	1186612	103679

2969	1 Q7Z6K5	Q7Z6K5	Arpin	ARPIN	Homo sapiens	24.943	9	9	3384455	464553
2970	1 Q86T65	Q86T65	Disheveled-associated activator of morphogenesis 2	DAAM2	Homo sapiens	123.501	9	9	1391923	0
2971	1 Q86TB9	Q86TB9	Protein PAT1 homolog 1	PATL1	Homo sapiens	86.851	9	9	794505	773774
2972	1 Q86TV6	Q86TV6	Tetratricopeptide repeat protein 7B	TTC7B	Homo sapiens	94.182	9	9	962323	153890
2973	1 Q8IU81	Q8IU81	Interferon regulatory factor 2-binding protein 1	IRF2BP1	Homo sapiens	61.688	9	9	767865	80105
2974	1 Q8IUZ5	Q8IUZ5	5-phosphohydroxy-L-lysine phospho-lyase	PHYKPL	Homo sapiens	49.71	9	9	1330129	362645
2975	1 Q8IV04	Q8IV04	Carabin	TBC1D10C	Homo sapiens	49.714	9	9	161443	2900457
2976	1 Q8IVD9	Q8IVD9	NudC domain-containing protein 3	NUDCD3	Homo sapiens	40.821	9	9	2434050	615018
2977	1 Q8IWB9	Q8IWB9	Testis-expressed protein 2	TEX2	Homo sapiens	125.305	9	9	652798	345305
2978	1 Q8IY18	Q8IY18	Structural maintenance of chromosomes protein 5	SMC5	Homo sapiens	128.808	9	9	592714	304728
2979	1 Q8IZ81	Q8IZ81	ELMO domain-containing protein 2	ELMOD2	Homo sapiens	34.961	9	9	3118836	2622175
2980	1 Q8N0X4	Q8N0X4	Citramalyl-CoA lyase, mitochondrial	CLYBL	Homo sapiens	37.358	9	9	3152071	97283
2981	1 Q8N2G8	Q8N2G8	GH3 domain-containing protein	GHDC	Homo sapiens	57.523	9	9	948783	951230
2982	1 Q8N436	Q8N436	Inactive carboxypeptidase-like protein X2	CPXM2	Homo sapiens	85.871	9	9	1451244	0
2983	1 Q8N4C8	Q8N4C8	Misshapen-like kinase 1	MINK1	Homo sapiens	149.824	9	9	1094103	351974
2984	1 Q8N5V2	Q8N5V2	Ephexin-1	NGEF	Homo sapiens	82.499	9	9	709679	0
2985	1 Q8N684	Q8N684	Cleavage and polyadenylation specificity factor subunit 7	CPSF7	Homo sapiens	52.049	9	9	4016352	1763336
2986	1 Q8NB49	Q8NB49	Phospholipid-transporting ATPase IG	ATP11C	Homo sapiens	129.48	9	9	607014	754080
2987	1 Q8NBJ7	Q8NBJ7	Inactive C-alpha-formylglycine-generating enzyme 2	SUMF2	Homo sapiens	33.842	9	9	7024396	3121391
2988	1 Q8NBN7	Q8NBN7	Retinol dehydrogenase 13	RDH13	Homo sapiens	35.934	9	9	2027573	88048
2989	1 Q8NBP0	Q8NBP0	Tetratricopeptide repeat protein 13	TTC13	Homo sapiens	96.814	9	9	395269	243005
2990	1 Q8NBP7	Q8NBP7	Proprotein convertase subtilisin/kexin type 9	PCSK9	Homo sapiens	74.289	9	9	0	2068553
2991	1 Q8NBU5	Q8NBU5	Outer mitochondrial transmembrane helix translocase	ATAD1	Homo sapiens	40.744	9	9	2478640	759610
2992	1 Q8ND56	Q8ND56	Protein LSM14 homolog A	LSM14A	Homo sapiens	50.53	9	9	1546927	529019
2993	1 Q8NDT2	Q8NDT2	Putative RNA-binding protein 15B	RBM15B	Homo sapiens	97.206	9	9	490909	28367
2994	1 Q8NFV4	Q8NFV4	Protein ABHD11	ABHD11	Homo sapiens	34.688	9	9	7906902	758314
2995	1 Q8TBC4	Q8TBC4	NEDD8-activating enzyme E1 catalytic subunit	UBA3	Homo sapiens	51.854	9	9	4587203	253078
2996	1 Q8TC12	Q8TC12	Retinol dehydrogenase 11	RDH11	Homo sapiens	35.386	9	9	5200954	2870597
2997	1 Q8TCY9	Q8TCY9	Up-regulator of cell proliferation	URGCP	Homo sapiens	104.987	9	9	365225	0
2998	1 Q8TDX7	Q8TDX7	Serine/threonine-protein kinase Nek7	NEK7	Homo sapiens	34.55	9	9	4054778	1590709
2999	1 Q8TF42	Q8TF42	Ubiquitin-associated and SH3 domain-containing protein B	UBASH3B	Homo sapiens	72.698	9	9	470896	1123382
3000	1 Q8WU76	Q8WU76	Sec1 family domain-containing protein 2	SCFD2	Homo sapiens	75.127	9	9	1377521	134522
3001	1 Q8WUF5	Q8WUF5	RelA-associated inhibitor	PPP1R13L	Homo sapiens	89.092	9	9	562960	192517
3002	1 Q8WUX9	Q8WUX9	Charged multivesicular body protein 7	CHMP7	Homo sapiens	50.912	9	9	902619	738545
3003	1 Q8WW59	Q8WW59	SPRY domain-containing protein 4	SPRYD4	Homo sapiens	23.127	9	9	4355106	1618503
3004	1 Q8WWA0	Q8WWA0	Intelectin-1	ITLN1	Homo sapiens	34.964	13	9	22159114	408419
3005	1 Q8WWP7	Q8WWP7	GTPase IMAP family member 1	GIMAP1	Homo sapiens	34.369	9	9	1708707	1590280
3006	1 Q8WXG6	Q8WXG6	MAP kinase-activating death domain protein	MADD	Homo sapiens	183.305	9	9	310527	0
3007	1 Q8WYL5	Q8WYL5	Protein phosphatase Slingshot homolog 1	SSH1	Homo sapiens	115.512	10	9	680484	229570
3008	1 Q92539	Q92539	Phosphatidate phosphatase LPIN2	LPIN2	Homo sapiens	99.4	10	9	420011	674306
3009	1 Q92552	Q92552	28S ribosomal protein S27, mitochondrial	MRPS27	Homo sapiens	47.611	9	9	2148271	403787
3010	1 Q92736	Q92736	Ryanodine receptor 2	RYR2	Homo sapiens	564.578	10	9	292593	0
3011	1 Q92777	Q92777	Synapsin-2	SYN2	Homo sapiens	62.998	9	9	1584104	0
3012	1 Q92796	Q92796	Disks large homolog 3	DLG3	Homo sapiens	90.316	9	9	2192446	42984
3013	1 Q92851	Q92851	Caspase-10	CASP10	Homo sapiens	58.952	9	9	629201	701672
3014	1 Q92890	Q92890	Ubiquitin recognition factor in ER-associated degradation protein 1	UFD1	Homo sapiens	34.5	9	9	3150407	1152637
3015	1 Q92905	Q92905	COP9 signalosome complex subunit 5	COPS5	Homo sapiens	37.579	9	9	6114087	2070845
3016	1 Q92922	Q92922	SWI/SNF complex subunit SMARCC1	SMARCC1	Homo sapiens	122.87	9	9	1473294	328073

3017	1	Q969Q5	Q969Q5	Ras-related protein Rab-24	RAB24	Homo sapiens	23.123	9	9	739336	4485702
3018	1	Q96BJ3	Q96BJ3	Axin interactor, dorsalization-associated protein	AIDA	Homo sapiens	35.021	9	9	4214546	1248897
3019	1	Q96BY7	Q96BY7	Autophagy-related protein 2 homolog B	ATG2B	Homo sapiens	232.768	9	9	709616	0
3020	1	Q96C23	Q96C23	Galactose mutarotase	GALM	Homo sapiens	37.766	9	9	15051009	2778464
3021	1	Q96CN9	Q96CN9	GRIP and coiled-coil domain-containing protein 1	GCC1	Homo sapiens	87.812	9	9	393312	297589
3022	1	Q96CT7	Q96CT7	Coiled-coil domain-containing protein 124	CCDC124	Homo sapiens	25.835	9	9	4428616	1190193
3023	1	Q96DZ1	Q96DZ1	Endoplasmic reticulum lectin 1	ERLEC1	Homo sapiens	54.859	9	9	971014	729115
3024	1	Q96FV2	Q96FV2	Secernin-2	SCRN2	Homo sapiens	46.597	9	9	3058205	977946
3025	1	Q96FW1	Q96FW1	Ubiquitin thioesterase OTUB1	OTUB1	Homo sapiens	31.285	9	9	5292392	3972269
3026	1	Q96GS4	Q96GS4	BLOC-1-related complex subunit 6	BORCS6	Homo sapiens	37.225	9	9	1127739	941421
3027	1	Q96IV0	Q96IV0	Peptide-N(4)-(N-acetyl-beta-glucosaminyl)asparagine amidase	NGLY1	Homo sapiens	74.39	9	9	817673	447953
3028	1	Q96JK2	Q96JK2	DDB1- and CUL4-associated factor 5	DCAF5	Homo sapiens	103.965	9	9	537174	0
3029	1	Q96KA5	Q96KA5	Lipid scramblase CLPTM1L	CLPTM1L	Homo sapiens	62.23	9	9	1174662	448238
3030	1	Q96PP8	Q96PP8	Guanylate-binding protein 5	GBP5	Homo sapiens	66.618	10	9	0	3242678
3031	1	Q96PU4	Q96PU4	E3 ubiquitin-protein ligase UHRF2	UHRF2	Homo sapiens	89.988	10	9	1176538	99671
3032	1	Q96Q11	Q96Q11	CCA tRNA nucleotidyltransferase 1, mitochondrial	TRNT1	Homo sapiens	50.129	9	9	1943332	1126585
3033	1	Q96QC0	Q96QC0	Serine/threonine-protein phosphatase 1 regulatory subunit 10	PPP1R10	Homo sapiens	99.059	9	9	2647683	7602576
3034	1	Q96QG7	Q96QG7	Myotubularin-related protein 9	MTMR9	Homo sapiens	63.462	9	9	1592510	195246
3035	1	Q96S59	Q96S59	Ran-binding protein 9	RANBP9	Homo sapiens	77.848	9	9	3010302	690512
3036	1	Q96S66	Q96S66	Chloride channel CLIC-like protein 1	CLCC1	Homo sapiens	62.022	9	9	1914981	761643
3037	1	Q99424	Q99424	Peroxisomal acyl-coenzyme A oxidase 2	ACOX2	Homo sapiens	76.827	9	9	785299	22532
3038	1	Q99487	Q99487	Platelet-activating factor acetylhydrolase 2, cytoplasmic	PAFAH2	Homo sapiens	44.036	9	9	2011667	428432
3039	1	Q99598	Q99598	Translin-associated protein X	TSNAX	Homo sapiens	33.112	9	9	3364769	2645857
3040	1	Q99805	Q99805	Transmembrane 9 superfamily member 2	TM9SF2	Homo sapiens	75.775	9	9	9812803	4551263
3041	1	Q99816	Q99816	Tumor susceptibility gene 101 protein	TSG101	Homo sapiens	43.944	9	9	3541659	1232134
3042	1	Q9BPU6	Q9BPU6	Dihydropyrimidinase-related protein 5	DPYSL5	Homo sapiens	61.422	9	9	1345232	0
3043	1	Q9BPW8	Q9BPW8	Protein NipSnap homolog 1	NIPSNAP1	Homo sapiens	33.31	9	9	7432595	2966605
3044	1	Q9BPX7	Q9BPX7	UPF0415 protein C7orf25	C7orf25	Homo sapiens	46.452	9	9	524197	915552
3045	1	Q9BQL6	Q9BQL6	Fermitin family homolog 1	FERMT1	Homo sapiens	77.438	9	9	2399152	75482
3046	1	Q9BQP7	Q9BQP7	Mitochondrial genome maintenance exonuclease 1	MGME1	Homo sapiens	39.419	9	9	605242	117408
3047	1	Q9BSD7	Q9BSD7	Cancer-related nucleoside-triphosphatase	NTPCR	Homo sapiens	20.712	9	9	3488388	749412
3048	1	Q9BT22	Q9BT22	Chitobiosyldiphosphodolichol beta-mannosyltransferase	ALG1	Homo sapiens	52.519	9	9	1770277	1324504
3049	1	Q9BUF5	Q9BUF5	Tubulin beta-6 chain	TUBB6	Homo sapiens	49.856	15	9	14831651	2390239
3050	1	Q9BV20	Q9BV20	Methylthioribose-1-phosphate isomerase	MRI1	Homo sapiens	39.147	9	9	1818750	1966909
3051	1	Q9BV44	Q9BV44	tRNA (guanine(6)-N2)-methyltransferase THUMP3	THUMPD3	Homo sapiens	57.003	9	9	660076	373021
3052	1	Q9BVQ7	Q9BVQ7	Ribosome biogenesis protein SPATA5L1	SPATA5L1	Homo sapiens	80.71	9	9	593820	254467
3053	1	Q9BW91	Q9BW91	ADP-ribose pyrophosphatase, mitochondrial	NUDT9	Homo sapiens	39.124	9	9	1331397	274203
3054	1	Q9BWD1	Q9BWD1	Acetyl-CoA acetyltransferase, cytosolic	ACAT2	Homo sapiens	41.351	9	9	4425293	1791934
3055	1	Q9BX97	Q9BX97	Plasmalemma vesicle-associated protein	PLVAP	Homo sapiens	50.596	9	9	1703281	99065
3056	1	Q9BXB4	Q9BXB4	Oxysterol-binding protein-related protein 11	OSBPL11	Homo sapiens	83.642	10	9	731427	900152
3057	1	Q9BXD5	Q9BXD5	N-acetylneuraminate lyase	NPL	Homo sapiens	35.159	9	9	339232	333012
3058	1	Q9BXK5	Q9BXK5	Bcl-2-like protein 13	BCL2L13	Homo sapiens	52.724	9	9	1798727	473844
3059	1	Q9BY49	Q9BY49	Peroxisomal trans-2-enoyl-CoA reductase	PECR	Homo sapiens	32.545	9	9	1471152	4116284
3060	1	Q9BYD1	Q9BYD1	39S ribosomal protein L13, mitochondrial	MRPL13	Homo sapiens	20.69	9	9	3084167	607620
3061	1	Q9BZK7	Q9BZK7	F-box-like/WD repeat-containing protein TBL1XR1	TBL1XR1	Homo sapiens	55.597	13	9	1922316	1231908
3062	1	Q9C0B1	Q9C0B1	Alpha-ketoglutarate-dependent dioxygenase FTO	FTO	Homo sapiens	58.283	9	9	1411391	724568
3063	1	Q9GZS3	Q9GZS3	SKI8 subunit of superkiller complex protein	SKIC8	Homo sapiens	33.58	9	9	3108124	1351576
3064	1	Q9GZT9	Q9GZT9	Egl nine homolog 1	EGLN1	Homo sapiens	46.019	11	9	1479901	3368076

3065	1	Q9H0J9	Q9H0J9	Protein mono-ADP-ribosyltransferase PARP12	PARP12	Homo sapiens	79.065	9	9	1396779	377198
3066	1	Q9H115	Q9H115	Beta-soluble NSF attachment protein	NAPB	Homo sapiens	33.558	9	9	2303741	1199409
3067	1	Q9H1P3	Q9H1P3	Oxysterol-binding protein-related protein 2	OSBPL2	Homo sapiens	55.201	9	9	683509	644951
3068	1	Q9H479	Q9H479	Fructosamine-3-kinase	FN3K	Homo sapiens	35.172	9	9	5037463	726838
3069	1	Q9H6R3	Q9H6R3	Acyl-CoA synthetase short-chain family member 3, mitochondrial	ACSS3	Homo sapiens	74.779	9	9	887875	55756
3070	1	Q9H7Z7	Q9H7Z7	Prostaglandin E synthase 2	PTGES2	Homo sapiens	41.941	9	9	5039991	1152086
3071	1	Q9H939	Q9H939	Proline-serine-threonine phosphatase-interacting protein 2	PSTPIP2	Homo sapiens	38.858	9	9	108504	1818316
3072	1	Q9H9P8	Q9H9P8	L-2-hydroxyglutarate dehydrogenase, mitochondrial	L2HGDH	Homo sapiens	50.315	9	9	1171645	327959
3073	1	Q9HAB8	Q9HAB8	Phosphopantothenate--cysteine ligase	PPCS	Homo sapiens	34.006	9	9	3417371	2914878
3074	1	Q9HAN9	Q9HAN9	Nicotinamide/nicotinic acid mononucleotide adenylyltransferase 1	NMNAT1	Homo sapiens	31.936	9	9	1516521	839645
3075	1	Q9HB07	Q9HB07	MYG1 exonuclease	MYG1	Homo sapiens	42.478	9	9	3821432	2853699
3076	1	Q9HBF4	Q9HBF4	Zinc finger FYVE domain-containing protein 1	ZFYVE1	Homo sapiens	87.177	9	9	731821	717324
3077	1	Q9HBI0	Q9HBI0	Gamma-parvin	PARVG	Homo sapiens	37.486	9	9	812164	7299797
3078	1	Q9HCK8	Q9HCK8	Chromodomain-helicase-DNA-binding protein 8	CHD8	Homo sapiens	290.523	10	9	484452	92735
3079	1	Q9HCM4	Q9HCM4	Band 4.1-like protein 5	EPB41L5	Homo sapiens	81.858	10	9	1006233	105655
3080	1	Q9HD45	Q9HD45	Transmembrane 9 superfamily member 3	TM9SF3	Homo sapiens	67.89	9	9	7021671	4473214
3081	1	Q9NP61	Q9NP61	ADP-ribosylation factor GTPase-activating protein 3	ARFGAP3	Homo sapiens	56.929	9	9	2116792	1967800
3082	1	Q9NPH3	Q9NPH3	Interleukin-1 receptor accessory protein	IL1RAP	Homo sapiens	65.417	9	9	205804	1920357
3083	1	Q9NPR2	Q9NPR2	Semaphorin-4B	SEMA4B	Homo sapiens	92.769	9	9	1626379	796338
3084	1	Q9NQY0	Q9NQY0	Bridging integrator 3	BIN3	Homo sapiens	29.664	9	9	2201478	28643
3085	1	Q9NRL2	Q9NRL2	Bromodomain adjacent to zinc finger domain protein 1A	BAZ1A	Homo sapiens	178.703	9	9	331074	867955
3086	1	Q9NRL3	Q9NRL3	Striatin-4	STRN4	Homo sapiens	80.595	9	9	3237552	201482
3087	1	Q9NRN5	Q9NRN5	Olfactomedin-like protein 3	OLFML3	Homo sapiens	46.011	9	9	8167185	1039285
3088	1	Q9NSC5	Q9NSC5	Homer protein homolog 3	HOMER3	Homo sapiens	39.836	9	9	303639	388378
3089	1	Q9NTJ4	Q9NTJ4	Alpha-mannosidase 2C1	MAN2C1	Homo sapiens	115.836	9	9	1236233	105650
3090	1	Q9NUJ1	Q9NUJ1	Palmitoyl-protein thioesterase ABHD10, mitochondrial	ABHD10	Homo sapiens	33.932	9	9	4838321	929472
3091	1	Q9NVG8	Q9NVG8	TBC1 domain family member 13	TBC1D13	Homo sapiens	46.555	9	9	2235776	486901
3092	1	Q9NVR2	Q9NVR2	Integrator complex subunit 10	INTS10	Homo sapiens	82.238	9	9	396987	335706
3093	1	Q9NVS9	Q9NVS9	Pyridoxine-5'-phosphate oxidase	PNPO	Homo sapiens	29.989	9	9	1853778	564411
3094	1	Q9NW08	Q9NW08	DNA-directed RNA polymerase III subunit RPC2	POLR3B	Homo sapiens	127.786	9	9	430010	20864
3095	1	Q9NWQ8	Q9NWQ8	Phosphoprotein associated with glycosphingolipid-enriched microdomains 1	PAG1	Homo sapiens	46.979	9	9	1447709	1786435
3096	1	Q9NX05	Q9NX05	Constitutive coactivator of PPAR-gamma-like protein 2	FAM120C	Homo sapiens	120.59	9	9	709730	12363
3097	1	Q9NXE4	Q9NXE4	Sphingomyelin phosphodiesterase 4	SMPD4	Homo sapiens	97.809	9	9	1286678	538742
3098	1	Q9NXH8	Q9NXH8	Torsin-4A	TOR4A	Homo sapiens	46.914	9	9	867054	890911
3099	1	Q9NXH9	Q9NXH9	tRNA (guanine(26)-N(2))-dimethyltransferase	TRMT1	Homo sapiens	72.233	9	9	770769	195100
3100	1	Q9NXR7	Q9NXR7	BRISC and BRCA1-A complex member 2	BABAM2	Homo sapiens	43.55	9	9	2116281	580327
3101	1	Q9NY27	Q9NY27	Serine/threonine-protein phosphatase 4 regulatory subunit 2	PPP4R2	Homo sapiens	46.899	9	9	947573	331451
3102	1	Q9NY59	Q9NY59	Sphingomyelin phosphodiesterase 3	SMPD3	Homo sapiens	71.08	9	9	1431499	0
3103	1	Q9NYB0	Q9NYB0	Telomeric repeat-binding factor 2-interacting protein 1	TERF2IP	Homo sapiens	44.257	9	9	1643510	535285
3104	1	Q9NYV4	Q9NYV4	Cyclin-dependent kinase 12	CDK12	Homo sapiens	164.158	10	9	413731	299230
3105	1	Q9NZA1	Q9NZA1	Chloride intracellular channel protein 5	CLIC5	Homo sapiens	46.502	9	9	1809518	100074
3106	1	Q9NZC9	Q9NZC9	SWI/SNF-related matrix-associated actin-dependent regulator of chromatin subfamily A-like protein 1	SMARCAL1	Homo sapiens	105.941	9	9	390552	271319
3107	1	Q9NZJ7	Q9NZJ7	Mitochondrial carrier homolog 1	MTCH1	Homo sapiens	41.543	9	9	6735438	1525701
3108	1	Q9NZL4	Q9NZL4	Hsp70-binding protein 1	HSPBP1	Homo sapiens	39.302	9	9	1826787	709605
3109	1	Q9P0M6	Q9P0M6	Core histone macro-H2A.2	MACROH2A2	Homo sapiens	40.056	9	9	11495782	780503
3110	1	Q9P206	Q9P206	Uncharacterized protein KIAA1522	KIAA1522	Homo sapiens	107.096	9	9	1570194	128592

3111	1	Q9P219	Q9P219	Protein Daple	CCDC88C	Homo sapiens	228.234	9	9	359289	212426
3112	1	Q9P2K6	Q9P2K6	Kelch-like protein 42	KLHL42	Homo sapiens	56.869	9	9	1713611	0
3113	1	Q9P2N5	Q9P2N5	RNA-binding protein 27	RBM27	Homo sapiens	118.72	9	9	902375	290946
3114	1	Q9UBB5	Q9UBB5	Methyl-CpG-binding domain protein 2	MBD2	Homo sapiens	43.254	9	9	1672350	685900
3115	1	Q9UBE0	Q9UBE0	SUMO-activating enzyme subunit 1	SAE1	Homo sapiens	38.449	9	9	3450309	1163253
3116	1	Q9UBL3	Q9UBL3	Set1/Ash2 histone methyltransferase complex subunit ASH2	ASH2L	Homo sapiens	68.723	9	9	1414454	376358
3117	1	Q9UBQ0	Q9UBQ0	Vacuolar protein sorting-associated protein 29	VPS29	Homo sapiens	20.504	9	9	8413184	5516904
3118	1	Q9UBR2	Q9UBR2	Cathepsin Z	CTSZ	Homo sapiens	33.869	9	9	14893135	10539405
3119	1	Q9UBS4	Q9UBS4	DnaJ homolog subfamily B member 11	DNAJB11	Homo sapiens	40.511	9	9	5007479	5195471
3120	1	Q9UBW5	Q9UBW5	Bridging integrator 2	BIN2	Homo sapiens	61.877	9	9	360919	3995239
3121	1	Q9UBX5	Q9UBX5	Fibulin-5	FBLN5	Homo sapiens	50.181	9	9	10251996	3947172
3122	1	Q9UDY8	Q9UDY8	Mucosa-associated lymphoid tissue lymphoma translocation protein 1	MALT1	Homo sapiens	92.274	9	9	384005	297930
3123	1	Q9UEU0	Q9UEU0	Vesicle transport through interaction with t-SNAREs homolog 1B	VTI1B	Homo sapiens	26.69	9	9	1860512	1220536
3124	1	Q9UFC0	Q9UFC0	Leucine-rich repeat and WD repeat-containing protein 1	LRWD1	Homo sapiens	70.861	9	9	382488	175051
3125	1	Q9UGP4	Q9UGP4	LIM domain-containing protein 1	LIMD1	Homo sapiens	72.191	10	9	1497758	276761
3126	1	Q9UIF9	Q9UIF9	Bromodomain adjacent to zinc finger domain protein 2A	BAZ2A	Homo sapiens	211.2	9	9	550707	137083
3127	1	Q9UJX4	Q9UJX4	Anaphase-promoting complex subunit 5	ANAPC5	Homo sapiens	85.079	9	9	484627	0
3128	1	Q9UK99	Q9UK99	F-box only protein 3	FBXO3	Homo sapiens	54.56	9	9	812614	210073
3129	1	Q9UKM7	Q9UKM7	Endoplasmic reticulum mannosyl-oligosaccharide 1,2-alpha-mannosidase	MAN1B1	Homo sapiens	79.581	9	9	641355	343413
3130	1	Q9UL54	Q9UL54	Serine/threonine-protein kinase TAO2	TAOK2	Homo sapiens	138.255	9	9	586439	735109
3131	1	Q9ULB1	Q9ULB1	Neurexin-1	NRXN1	Homo sapiens	161.885	12	9	688015	0
3132	1	Q9ULH0	Q9ULH0	Kinase D-interacting substrate of 220 kDa	KIDINS220	Homo sapiens	196.545	9	9	381676	59212
3133	1	Q9ULJ7	Q9ULJ7	Ankyrin repeat domain-containing protein 50	ANKRD50	Homo sapiens	155.861	9	9	704950	0
3134	1	Q9UM07	Q9UM07	Protein-arginine deiminase type-4	PADI4	Homo sapiens	74.082	10	9	355373	35565381
3135	1	Q9UMY4	Q9UMY4	Sorting nexin-12	SNX12	Homo sapiens	18.884	9	9	3376831	1152392
3136	1	Q9UN37	Q9UN37	Vacuolar protein sorting-associated protein 4A	VPS4A	Homo sapiens	48.899	9	9	4288898	2909817
3137	1	Q9UNP9	Q9UNP9	Peptidyl-prolyl cis-trans isomerase E	PPIE	Homo sapiens	33.431	9	9	2024241	715227
3138	1	Q9UPN7	Q9UPN7	Serine/threonine-protein phosphatase 6 regulatory subunit 1	PPP6R1	Homo sapiens	96.724	9	9	1436312	2041573
3139	1	Q9UPR5	Q9UPR5	Sodium/calcium exchanger 2	SLC8A2	Homo sapiens	100.369	9	9	1105092	33253
3140	1	Q9Y243	Q9Y243	RAC-gamma serine/threonine-protein kinase	AKT3	Homo sapiens	55.777	9	9	834017	0
3141	1	Q9Y365	Q9Y365	START domain-containing protein 10	STARD10	Homo sapiens	33.05	9	9	13339571	333073
3142	1	Q9Y371	Q9Y371	Endophilin-B1	SH3GLB1	Homo sapiens	40.795	9	9	6423824	4179326
3143	1	Q9Y4B5	Q9Y4B5	Microtubule cross-linking factor 1	MTCL1	Homo sapiens	209.528	9	9	524414	0
3144	1	Q9Y4G8	Q9Y4G8	Rap guanine nucleotide exchange factor 2	RAPGEF2	Homo sapiens	167.42	9	9	532604	129991
3145	1	Q9Y4X5	Q9Y4X5	E3 ubiquitin-protein ligase ARIH1	ARIH1	Homo sapiens	64.118	9	9	1336026	616010
3146	1	Q9Y5K5	Q9Y5K5	Ubiquitin carboxyl-terminal hydrolase isozyme L5	UCHL5	Homo sapiens	37.607	9	9	3963671	3200010
3147	1	Q9Y5S1	Q9Y5S1	Transient receptor potential cation channel subfamily V member 2	TRPV2	Homo sapiens	85.981	9	9	717890	980082
3148	1	Q9Y5T5	Q9Y5T5	Ubiquitin carboxyl-terminal hydrolase 16	USP16	Homo sapiens	93.572	9	9	1263576	125632
3149	1	Q9Y6A5	Q9Y6A5	Transforming acidic coiled-coil-containing protein 3	TACC3	Homo sapiens	90.36	9	9	96717	692636
3150	1	Q9Y6N7	Q9Y6N7	Roundabout homolog 1	ROBO1	Homo sapiens	180.932	9	9	1199792	0
3151	1	Q9Y6Q1	Q9Y6Q1	Calpain-6	CAPN6	Homo sapiens	74.576	9	9	1024224	0
3152	1	Q9Y6Q5	Q9Y6Q5	AP-1 complex subunit mu-2	AP1M2	Homo sapiens	48.107	9	9	1696896	577746
3153	1	Q9Y6R4	Q9Y6R4	Mitogen-activated protein kinase kinase kinase 4	MAP3K4	Homo sapiens	181.687	9	9	558165	40380
3154	1	Q9Y6W5	Q9Y6W5	Actin-binding protein WASF2	WASF2	Homo sapiens	54.284	9	9	6646064	2241534
3155	2	A0A0B4J2D5;P0D	A0A0B4J2D5	Putative glutamine amidotransferase-like class 1 domain-containing protein 3B, mitochondrial	GATD3B	Homo sapiens	28.141	8	8	18772168	3086878
3156	2	G9CGD6;Q8WWN	G9CGD6	CNK3/IPCEF1 fusion protein	CNK3/IPCEF1	Homo sapiens	100.369	8	8	45857	1482479

3157	1	O00303	O00303	Eukaryotic translation initiation factor 3 subunit F	EIF3F	Homo sapiens	37.562	8	8	10028803	1719384
3158	1	O00330	O00330	Pyruvate dehydrogenase protein X component, mitochondrial	PDHX	Homo sapiens	54.124	8	8	2991125	940240
3159	1	O00519	O00519	Fatty-acid amide hydrolase 1	FAAH	Homo sapiens	63.064	8	8	1330679	234604
3160	1	O00571	O00571	ATP-dependent RNA helicase DDX3X	DDX3X	Homo sapiens	73.246	29	8	6030356	3546719
3161	1	O00750	O00750	Phosphatidylinositol 4-phosphate 3-kinase C2 domain-containing subunit beta	PIK3C2B	Homo sapiens	184.77	8	8	4052177	0
3162	1	O14495	O14495	Phospholipid phosphatase 3	PLPP3	Homo sapiens	35.117	8	8	1953657	335391
3163	1	O14545	O14545	TRAF-type zinc finger domain-containing protein 1	TRAFD1	Homo sapiens	64.844	8	8	228735	718213
3164	1	O14795	O14795	Protein unc-13 homolog B	UNC13B	Homo sapiens	180.684	9	8	431929	0
3165	1	O15381	O15381	Nuclear valosin-containing protein-like	NVL	Homo sapiens	95.053	8	8	467909	55467
3166	1	O15455	O15455	Toll-like receptor 3	TLR3	Homo sapiens	103.83	8	8	688963	624890
3167	1	O43148	O43148	mRNA cap guanine-N7 methyltransferase	RNMT	Homo sapiens	54.844	8	8	1539353	732793
3168	1	O43427	O43427	Acidic fibroblast growth factor intracellular-binding protein	FIBP	Homo sapiens	41.879	8	8	798914	202530
3169	1	O43488	O43488	Aflatoxin B1 aldehyde reductase member 2	AKR7A2	Homo sapiens	39.588	10	8	5369259	1487106
3170	1	O43684	O43684	Mitotic checkpoint protein BUB3	BUB3	Homo sapiens	37.154	8	8	9236223	6386839
3171	1	O43741	O43741	5'-AMP-activated protein kinase subunit beta-2	PRKAB2	Homo sapiens	30.302	8	8	1752832	214182
3172	1	O43823	O43823	A-kinase anchor protein 8	AKAP8	Homo sapiens	76.109	8	8	1593010	146982
3173	1	O60218	O60218	Aldo-keto reductase family 1 member B10	AKR1B10	Homo sapiens	36.02	14	8	6826912	37860590
3174	1	O60229	O60229	Kalirin	KALRN	Homo sapiens	340.265	8	8	768876	24144
3175	1	O60244	O60244	Mediator of RNA polymerase II transcription subunit 14	MED14	Homo sapiens	160.608	8	8	1047919	186344
3176	1	O60268	O60268	Uncharacterized protein KIAA0513	KIAA0513	Homo sapiens	46.639	8	8	2229276	2412670
3177	1	O60315	O60315	Zinc finger E-box-binding homeobox 2	ZEB2	Homo sapiens	136.448	10	8	430126	63686
3178	1	O75223	O75223	Gamma-glutamylcyclotransferase	GGCT	Homo sapiens	21.007	8	8	7417761	3895902
3179	1	O75347	O75347	Tubulin-specific chaperone A	TBCA	Homo sapiens	12.855	8	8	10017237	5073792
3180	1	O75381	O75381	Peroxisomal membrane protein PEX14	PEX14	Homo sapiens	41.236	8	8	1466999	573113
3181	1	O75431	O75431	Metaxin-2	MTX2	Homo sapiens	29.763	8	8	2785132	831807
3182	1	O75487	O75487	Glypican-4	GPC4	Homo sapiens	62.413	8	8	3363727	0
3183	1	O75569	O75569	Interferon-inducible double-stranded RNA-dependent protein kinase activator A	PRKRA	Homo sapiens	34.402	8	8	4130463	1204044
3184	1	O75663	O75663	TIP41-like protein	TIPRL	Homo sapiens	31.444	8	8	2530567	1523602
3185	1	O75764	O75764	Transcription elongation factor A protein 3	TCEA3	Homo sapiens	38.973	8	8	1989934	0
3186	1	O75821	O75821	Eukaryotic translation initiation factor 3 subunit G	EIF3G	Homo sapiens	35.61	8	8	5512854	2581431
3187	1	O75884	O75884	Serine hydrolase RBBP9	RBBP9	Homo sapiens	21	8	8	6216931	1224415
3188	1	O75947	O75947	ATP synthase subunit d, mitochondrial	ATP5PD	Homo sapiens	18.492	8	8	27068763	10741107
3189	1	O76070	O76070	Gamma-synuclein	SNCG	Homo sapiens	13.328	8	8	8389564	78504
3190	1	O94886	O94886	CSC1-like protein 1	TMEM63A	Homo sapiens	92.13	8	8	978519	2244652
3191	1	O95155	O95155	Ubiquitin conjugation factor E4 B	UBE4B	Homo sapiens	146.184	8	8	768474	246264
3192	1	O95297	O95297	Myelin protein zero-like protein 1	MPZL1	Homo sapiens	29.082	8	8	1992020	520466
3193	1	O95407	O95407	Tumor necrosis factor receptor superfamily member 6B	TNFRSF6B	Homo sapiens	32.679	8	8	103513	2101258
3194	1	O95428	O95428	Papilin	PAPLN	Homo sapiens	137.702	8	8	593297	77011
3195	1	O95747	O95747	Serine/threonine-protein kinase OSR1	OXSR1	Homo sapiens	58.023	12	8	7100544	3413481
3196	1	O95841	O95841	Angiopoietin-related protein 1	ANGPTL1	Homo sapiens	56.72	8	8	906112	0
3197	1	O95967	O95967	EGF-containing fibulin-like extracellular matrix protein 2	EFEMP2	Homo sapiens	49.404	8	8	2970378	1709271
3198	1	O95980	O95980	Reversion-inducing cysteine-rich protein with Kazal motifs	RECK	Homo sapiens	106.458	8	8	1209203	47598
3199	1	O96005	O96005	Putative lipid scramblase CLPTM1	CLPTM1	Homo sapiens	76.098	8	8	1979166	1822247
3200	1	P00387	P00387	NADH-cytochrome b5 reductase 3	CYB5R3	Homo sapiens	34.236	8	8	28078448	9013387
3201	1	P00750	P00750	Tissue-type plasminogen activator	PLAT	Homo sapiens	62.918	8	8	1213903	0
3202	1	P02462	P02462	Collagen alpha-1(IV) chain	COL4A1	Homo sapiens	160.612	10	8	30970518	9950596

3203	1	P04179	P04179	Superoxide dismutase [Mn], mitochondrial	SOD2	Homo sapiens	24.75	8	8	41995014	180336650
3204	1	P04278	P04278	Sex hormone-binding globulin	SHBG	Homo sapiens	43.779	8	8	527173	3373387
3205	1	P04626	P04626	Receptor tyrosine-protein kinase erbB-2	ERBB2	Homo sapiens	137.912	8	8	1671812	16442
3206	1	P04839	P04839	Cytochrome b-245 heavy chain	CYBB	Homo sapiens	65.334	8	8	4181059	62696336
3207	1	P05120	P05120	Plasminogen activator inhibitor 2	SERPINB2	Homo sapiens	46.596	8	8	0	3459052
3208	1	P05771	P05771	Protein kinase C beta type	PRKCB	Homo sapiens	76.869	13	8	3028179	2984355
3209	1	P05997	P05997	Collagen alpha-2(V) chain	COL5A2	Homo sapiens	144.912	8	8	836234	2404339
3210	1	P06280	P06280	Alpha-galactosidase A	GLA	Homo sapiens	48.769	8	8	2382086	2246609
3211	1	P07355	P07355	Annexin A2	ANXA2	Homo sapiens	38.605	25	8	140968465	41460223
3212	1	P08069	P08069	Insulin-like growth factor 1 receptor	IGF1R	Homo sapiens	154.798	8	8	980053	124214
3213	1	P08138	P08138	Tumor necrosis factor receptor superfamily member 16	NGFR	Homo sapiens	45.184	8	8	676025	0
3214	1	P08397	P08397	Porphobilinogen deaminase	HMBS	Homo sapiens	39.329	8	8	907225	410665
3215	1	P08567	P08567	Pleckstrin	PLEK	Homo sapiens	40.127	8	8	1048716	5866297
3216	1	P08581	P08581	Hepatocyte growth factor receptor	MET	Homo sapiens	155.544	8	8	391998	441933
3217	1	P08708	P08708	40S ribosomal protein S17	RPS17	Homo sapiens	15.55	8	8	8574842	3728435
3218	1	P08842	P08842	Steryl-sulfatase	STS	Homo sapiens	65.493	8	8	882267	2867044
3219	1	P09417	P09417	Dihydropteridine reductase	QDPR	Homo sapiens	25.792	8	8	13757001	1315185
3220	1	P09486	P09486	SPARC	SPARC	Homo sapiens	34.634	8	8	867955	1485741
3221	1	P09496	P09496	Clathrin light chain A	CLTA	Homo sapiens	27.075	8	8	4668998	4226150
3222	1	P09884	P09884	DNA polymerase alpha catalytic subunit	POLA1	Homo sapiens	165.915	8	8	395934	70981
3223	1	P09914	P09914	Interferon-induced protein with tetratricopeptide repeats 1	IFIT1	Homo sapiens	55.361	10	8	1419047	2675387
3224	3	P0DP23;P0DP24;I	P0DP23	Calmodulin-1	CALM1	Homo sapiens	16.836	8	8	191183858	65395516
3225	1	P10915	P10915	Hyaluronan and proteoglycan link protein 1	HAPLN1	Homo sapiens	40.166	9	8	4343870	351843
3226	1	P11117	P11117	Lysosomal acid phosphatase	ACP2	Homo sapiens	48.344	8	8	2361079	1970686
3227	1	P12036	P12036	Neurofilament heavy polypeptide	NEFH	Homo sapiens	112.483	8	8	1218182	0
3228	1	P12081	P12081	Histidine--tRNA ligase, cytoplasmic	HARS1	Homo sapiens	57.411	13	8	7781881	2797340
3229	1	P13747	P13747	HLA class I histocompatibility antigen, alpha chain E	HLA-E	Homo sapiens	40.058	8	8	932589	1538904
3230	1	P15374	P15374	Ubiquitin carboxyl-terminal hydrolase isozyme L3	UCHL3	Homo sapiens	26.18	8	8	4118615	2252879
3231	1	P15428	P15428	15-hydroxyprostaglandin dehydrogenase [NAD(+)]	HPGD	Homo sapiens	28.977	8	8	3234696	20662
3232	1	P15880	P15880	40S ribosomal protein S2	RPS2	Homo sapiens	31.324	8	8	28276160	13146779
3233	1	P17050	P17050	Alpha-N-acetylgalactosaminidase	NAGA	Homo sapiens	46.565	8	8	5028084	1074504
3234	1	P17213	P17213	Bactericidal permeability-increasing protein	BPI	Homo sapiens	53.901	8	8	434700	19703970
3235	1	P18440	P18440	Arylamine N-acetyltransferase 1	NAT1	Homo sapiens	33.898	8	8	2084151	1786230
3236	1	P19404	P19404	NADH dehydrogenase [ubiquinone] flavoprotein 2, mitochondrial	NDUFV2	Homo sapiens	27.39	8	8	18064934	1407230
3237	1	P19652	P19652	Alpha-1-acid glycoprotein 2	ORM2	Homo sapiens	23.603	8	8	34415429	54864429
3238	1	P20585	P20585	DNA mismatch repair protein Msh3	MSH3	Homo sapiens	127.415	8	8	716126	348281
3239	1	P23497	P23497	Nuclear autoantigen Sp-100	SP100	Homo sapiens	100.418	10	8	1030809	779426
3240	1	P23677	P23677	Inositol-trisphosphate 3-kinase A	ITPKA	Homo sapiens	51.008	8	8	563864	0
3241	1	P24941	P24941	Cyclin-dependent kinase 2	CDK2	Homo sapiens	33.929	11	8	489811	432821
3242	1	P25686	P25686	DnaJ homolog subfamily B member 2	DNAJB2	Homo sapiens	35.581	8	8	1556982	565529
3243	1	P25788	P25788	Proteasome subunit alpha type-3	PSMA3	Homo sapiens	28.435	8	8	10788320	6067664
3244	1	P26045	P26045	Tyrosine-protein phosphatase non-receptor type 3	PTPN3	Homo sapiens	103.992	8	8	807613	0
3245	1	P26368	P26368	Splicing factor U2AF 65 kDa subunit	U2AF2	Homo sapiens	53.501	8	8	6205399	2876600
3246	1	P26572	P26572	Alpha-1,3-mannosyl-glycoprotein 2-beta-N-acetylglucosaminyltransferase	MGAT1	Homo sapiens	50.878	8	8	1011883	2275221
3247	1	P28482	P28482	Mitogen-activated protein kinase 1	MAPK1	Homo sapiens	41.39	12	8	18210312	8228290
3248	1	P29218	P29218	Inositol monophosphatase 1	IMPA1	Homo sapiens	30.188	8	8	6486287	2932746
3249	1	P30039	P30039	Phenazine biosynthesis-like domain-containing protein	PBLD	Homo sapiens	31.783	8	8	4801855	414705

3250	1	P30040	P30040	Endoplasmic reticulum resident protein 29	ERP29	Homo sapiens	28.992	8	8	38799523	28967085
3251	1	P30154	P30154	Serine/threonine-protein phosphatase 2A 65 kDa regulatory subunit A beta isoform	PPP2R1B	Homo sapiens	66.213	8	8	339717	521524
3252	1	P30530	P30530	Tyrosine-protein kinase receptor UFO	AXL	Homo sapiens	98.337	10	8	922258	148691
3253	1	P30533	P30533	Alpha-2-macroglobulin receptor-associated protein	LRPAP1	Homo sapiens	41.465	8	8	3207940	1916046
3254	1	P31943	P31943	Heterogeneous nuclear ribonucleoprotein H	HNRNPH1	Homo sapiens	49.228	14	8	12223425	9237817
3255	1	P32942	P32942	Intercellular adhesion molecule 3	ICAM3	Homo sapiens	59.541	8	8	531449	13502959
3256	1	P34059	P34059	N-acetylgalactosamine-6-sulfatase	GALNS	Homo sapiens	58.025	8	8	1500075	1265295
3257	1	P35250	P35250	Replication factor C subunit 2	RFC2	Homo sapiens	39.156	8	8	1452812	492251
3258	1	P35914	P35914	Hydroxymethylglutaryl-CoA lyase, mitochondrial	HMGCL	Homo sapiens	34.36	8	8	8987442	1721993
3259	1	P36542	P36542	ATP synthase subunit gamma, mitochondrial	ATP5F1C	Homo sapiens	32.995	8	8	30946906	6342137
3260	1	P37198	P37198	Nuclear pore glycoprotein p62	NUP62	Homo sapiens	53.257	8	8	2231146	1953694
3261	1	P37268	P37268	Squalene synthase	FDFT1	Homo sapiens	48.114	8	8	648544	0
3262	1	P38935	P38935	DNA-binding protein SMUBP-2	IGHMBP2	Homo sapiens	109.15	8	8	380795	71225
3263	1	P39900	P39900	Macrophage metalloelastase	MMP12	Homo sapiens	54.002	8	8	70273	916549
3264	1	P40227	P40227	T-complex protein 1 subunit zeta	CCT6A	Homo sapiens	58.024	10	8	17946950	11461625
3265	1	P41223	P41223	Protein BUD31 homolog	BUD31	Homo sapiens	17.001	8	8	502151	280359
3266	1	P42898	P42898	Methylenetetrahydrofolate reductase (NADPH)	MTHFR	Homo sapiens	74.597	8	8	490995	762213
3267	1	P45381	P45381	Aspartoacylase	ASPA	Homo sapiens	35.733	8	8	1701138	0
3268	1	P46952	P46952	3-hydroxyanthranilate 3,4-dioxygenase	HAAO	Homo sapiens	32.555	8	8	5305391	169519
3269	1	P46976	P46976	Glycogenin-1	GYG1	Homo sapiens	39.384	8	8	9432242	6548710
3270	1	P47736	P47736	Rap1 GTPase-activating protein 1	RAP1GAP	Homo sapiens	73.361	8	8	735004	0
3271	1	P49116	P49116	Nuclear receptor subfamily 2 group C member 2	NR2C2	Homo sapiens	65.414	8	8	2175198	418910
3272	1	P49720	P49720	Proteasome subunit beta type-3	PSMB3	Homo sapiens	22.948	8	8	3946552	1507397
3273	1	P49721	P49721	Proteasome subunit beta type-2	PSMB2	Homo sapiens	22.835	8	8	6220433	3512024
3274	1	P49755	P49755	Transmembrane emp24 domain-containing protein 10	TMED10	Homo sapiens	24.975	8	8	14658072	10971680
3275	1	P49789	P49789	Bis(5'-adenosyl)-triphosphatase	FHIT	Homo sapiens	16.856	8	8	1677881	45639
3276	1	P49840	P49840	Glycogen synthase kinase-3 alpha	GSK3A	Homo sapiens	50.98	8	8	2276715	686234
3277	1	P49841	P49841	Glycogen synthase kinase-3 beta	GSK3B	Homo sapiens	46.743	11	8	2194389	564031
3278	1	P50281	P50281	Matrix metalloproteinase-14	MMP14	Homo sapiens	65.896	8	8	3404645	1647797
3279	1	P50897	P50897	Palmitoyl-protein thioesterase 1	PPT1	Homo sapiens	34.193	8	8	1021391	549514
3280	1	P51151	P51151	Ras-related protein Rab-9A	RAB9A	Homo sapiens	22.838	10	8	2089447	2574576
3281	1	P51553	P51553	Isocitrate dehydrogenase [NAD] subunit gamma, mitochondrial	IDH3G	Homo sapiens	42.793	8	8	3004414	559432
3282	1	P51589	P51589	Cytochrome P450 2J2	CYP2J2	Homo sapiens	57.611	8	8	1162636	0
3283	1	P51649	P51649	Succinate-semialdehyde dehydrogenase, mitochondrial	ALDH5A1	Homo sapiens	57.216	8	8	1847287	369587
3284	1	P53597	P53597	Succinate--CoA ligase [ADP/GDP-forming] subunit alpha, mitochondrial	SUCLG1	Homo sapiens	36.249	8	8	27533159	4517416
3285	1	P53701	P53701	Holocytochrome c-type synthase	HCCS	Homo sapiens	30.603	8	8	1768019	602294
3286	1	P54253	P54253	Ataxin-1	ATXN1	Homo sapiens	86.922	8	8	602551	314085
3287	1	P55058	P55058	Phospholipid transfer protein	PLTP	Homo sapiens	54.74	8	8	1647189	1785799
3288	1	P55263	P55263	Adenosine kinase	ADK	Homo sapiens	40.544	8	8	1478502	2081677
3289	1	P55735	P55735	Protein SEC13 homolog	SEC13	Homo sapiens	35.542	8	8	5206061	2672096
3290	1	P57678	P57678	Gem-associated protein 4	GEMIN4	Homo sapiens	120.037	8	8	547787	291369
3291	1	P59998	P59998	Actin-related protein 2/3 complex subunit 4	ARPC4	Homo sapiens	19.666	8	8	39051116	46509100
3292	1	P60953	P60953	Cell division control protein 42 homolog	CDC42	Homo sapiens	21.257	9	8	5232525	4176370
3293	1	P61086	P61086	Ubiquitin-conjugating enzyme E2 K	UBE2K	Homo sapiens	22.407	8	8	7840653	3226012
3294	1	P62249	P62249	40S ribosomal protein S16	RPS16	Homo sapiens	16.444	8	8	25721511	11106281
3295	1	P62277	P62277	40S ribosomal protein S13	RPS13	Homo sapiens	17.221	8	8	30203838	8412904
3296	1	P62280	P62280	40S ribosomal protein S11	RPS11	Homo sapiens	18.429	8	8	14321010	8006158

3297	1	P62316	P62316	Small nuclear ribonucleoprotein Sm D2	SNRPD2	Homo sapiens	13.525	8	8	9761864	5637609
3298	1	P62424	P62424	60S ribosomal protein L7a	RPL7A	Homo sapiens	29.994	8	8	47107737	25419217
3299	1	P62753	P62753	40S ribosomal protein S6	RPS6	Homo sapiens	28.679	8	8	13714747	6197075
3300	1	P62826	P62826	GTP-binding nuclear protein Ran	RAN	Homo sapiens	24.421	8	8	36039433	32010585
3301	1	P62993	P62993	Growth factor receptor-bound protein 2	GRB2	Homo sapiens	25.205	8	8	9586710	8293375
3302	1	P63208	P63208	S-phase kinase-associated protein 1	SKP1	Homo sapiens	18.657	8	8	20071557	8604805
3303	1	P67936	P67936	Tropomyosin alpha-4 chain	TPM4	Homo sapiens	28.519	16	8	486236822	29429222
3304	1	P78362	P78362	SRSF protein kinase 2	SRPK2	Homo sapiens	77.527	8	8	1371049	989010
3305	1	P78417	P78417	Glutathione S-transferase omega-1	GSTO1	Homo sapiens	27.565	8	8	90094227	62581420
3306	1	P82675	P82675	28S ribosomal protein S5, mitochondrial	MRPS5	Homo sapiens	48.008	8	8	1245494	253205
3307	1	P98066	P98066	Tumor necrosis factor-inducible gene 6 protein	TNFAIP6	Homo sapiens	31.202	8	8	0	1646159
3308	1	Q02543	Q02543	60S ribosomal protein L18a	RPL18A	Homo sapiens	20.76	8	8	9458451	5203157
3309	1	Q03188	Q03188	Centromere protein C	CENPC	Homo sapiens	106.835	8	8	539850	304775
3310	1	Q05084	Q05084	Islet cell autoantigen 1	ICA1	Homo sapiens	54.646	8	8	581279	289894
3311	1	Q05823	Q05823	2-5A-dependent ribonuclease	RNASEL	Homo sapiens	83.534	8	8	517027	328296
3312	1	Q07020	Q07020	60S ribosomal protein L18	RPL18	Homo sapiens	21.636	8	8	38780705	16717865
3313	1	Q07075	Q07075	Glutamyl aminopeptidase	ENPEP	Homo sapiens	109.246	8	8	117507	621843
3314	1	Q08209	Q08209	Protein phosphatase 3 catalytic subunit alpha	PPP3CA	Homo sapiens	58.688	8	8	3749976	1883546
3315	1	Q08499	Q08499	cAMP-specific 3',5'-cyclic phosphodiesterase 4D	PDE4D	Homo sapiens	91.115	12	8	1464124	99450
3316	1	Q08999	Q08999	Retinoblastoma-like protein 2	RBL2	Homo sapiens	128.367	8	8	453793	53891
3317	1	Q0IIM8	Q0IIM8	TBC1 domain family member 8B	TBC1D8B	Homo sapiens	128.712	8	8	478413	243731
3318	1	Q0JRZ9	Q0JRZ9	F-BAR domain only protein 2	FCHO2	Homo sapiens	88.928	8	8	1854015	987761
3319	1	Q12899	Q12899	Tripartite motif-containing protein 26	TRIM26	Homo sapiens	62.165	8	8	1055014	197747
3320	1	Q13190	Q13190	Syntaxin-5	STX5	Homo sapiens	39.673	8	8	1927509	1318613
3321	1	Q13557	Q13557	Calcium/calmodulin-dependent protein kinase type II subunit delta	CAMK2D	Homo sapiens	56.369	9	8	6212825	682645
3322	1	Q13574	Q13574	Diacylglycerol kinase zeta	DGKZ	Homo sapiens	103.982	9	8	281693	1784005
3323	1	Q13637	Q13637	Ras-related protein Rab-32	RAB32	Homo sapiens	24.997	10	8	3394759	7083024
3324	1	Q13895	Q13895	Bystin	BYSL	Homo sapiens	49.601	8	8	546388	320626
3325	1	Q13905	Q13905	Rap guanine nucleotide exchange factor 1	RAPGEF1	Homo sapiens	120.549	8	8	683721	202832
3326	1	Q13938	Q13938	Calcyphosin	CAPS	Homo sapiens	30.24	8	8	1241602	852564
3327	1	Q14103	Q14103	Heterogeneous nuclear ribonucleoprotein D0	HNRNPD	Homo sapiens	38.435	8	8	29451841	17976414
3328	1	Q14137	Q14137	Ribosome biogenesis protein BOP1	BOP1	Homo sapiens	83.628	8	8	931733	67781
3329	1	Q14141	Q14141	Septin-6	SEPTIN6	Homo sapiens	49.716	8	8	5799328	1424355
3330	1	Q14155	Q14155	Rho guanine nucleotide exchange factor 7	ARHGEF7	Homo sapiens	90.013	8	8	2457926	437051
3331	1	Q14520	Q14520	Hyaluronan-binding protein 2	HABP2	Homo sapiens	62.672	11	8	570199	8521499
3332	1	Q14642	Q14642	Inositol polyphosphate-5-phosphatase A	INPP5A	Homo sapiens	47.818	8	8	1469099	176537
3333	1	Q14CN2	Q14CN2	Calcium-activated chloride channel regulator 4	CLCA4	Homo sapiens	101.285	8	8	1940827	0
3334	1	Q15003	Q15003	Condensin complex subunit 2	NCAPH	Homo sapiens	82.566	8	8	169110	559653
3335	1	Q15006	Q15006	ER membrane protein complex subunit 2	EMC2	Homo sapiens	34.83	8	8	1762292	1185002
3336	1	Q15111	Q15111	Inactive phospholipase C-like protein 1	PLCL1	Homo sapiens	122.732	8	8	576892	0
3337	1	Q15119	Q15119	[Pyruvate dehydrogenase (acetyl-transferring)] kinase isozyme 2, mitochondrial	PDK2	Homo sapiens	46.154	8	8	1212909	0
3338	1	Q15257	Q15257	Serine/threonine-protein phosphatase 2A activator	PTPA	Homo sapiens	40.667	8	8	4061566	1658748
3339	1	Q15397	Q15397	Pumilio homolog 3	PUM3	Homo sapiens	73.585	8	8	995515	396156
3340	1	Q15428	Q15428	Splicing factor 3A subunit 2	SF3A2	Homo sapiens	49.255	8	8	4252161	2059554
3341	1	Q15545	Q15545	Transcription initiation factor TFIID subunit 7	TAF7	Homo sapiens	40.258	8	8	1223008	96936
3342	1	Q15554	Q15554	Telomeric repeat-binding factor 2	TERF2	Homo sapiens	59.593	8	8	951947	304364
3343	1	Q15582	Q15582	Transforming growth factor-beta-induced protein ig-h3	TGFBI	Homo sapiens	74.68	21	8	71307970	18200353

3344	1	Q15596	Q15596	Nuclear receptor coactivator 2	NCOA2	Homo sapiens	159.157	8	8	305433	242019
3345	1	Q15631	Q15631	Translin	TSN	Homo sapiens	26.182	8	8	6391437	3418108
3346	1	Q15758	Q15758	Neutral amino acid transporter B(0)	SLC1A5	Homo sapiens	56.599	8	8	1626764	651332
3347	1	Q15785	Q15785	Mitochondrial import receptor subunit TOM34	TOMM34	Homo sapiens	34.558	8	8	1259601	413705
3348	1	Q15904	Q15904	V-type proton ATPase subunit S1	ATP6AP1	Homo sapiens	52.024	8	8	844884	2384579
3349	1	Q16585	Q16585	Beta-sarcoglycan	SGCB	Homo sapiens	34.775	8	8	2881153	0
3350	1	Q16629	Q16629	Serine/arginine-rich splicing factor 7	SRSF7	Homo sapiens	27.365	9	8	11903661	6573325
3351	1	Q16798	Q16798	NADP-dependent malic enzyme, mitochondrial	ME3	Homo sapiens	67.069	8	8	1192182	13953
3352	1	Q16799	Q16799	Reticulon-1	RTN1	Homo sapiens	83.616	8	8	1449477	101793
3353	1	Q16836	Q16836	Hydroxyacyl-coenzyme A dehydrogenase, mitochondrial	HADH	Homo sapiens	34.293	8	8	56755607	5461942
3354	1	Q16877	Q16877	6-phosphofructo-2-kinase/fructose-2,6-bisphosphatase 4	PFKFB4	Homo sapiens	54.039	9	8	0	2100354
3355	1	Q52LJ0	Q52LJ0	Protein FAM98B	FAM98B	Homo sapiens	45.546	11	8	5456975	5482290
3356	1	Q53F19	Q53F19	Nuclear cap-binding protein subunit 3	NCBP3	Homo sapiens	70.593	8	8	1280669	511469
3357	1	Q5H8A4	Q5H8A4	GPI ethanolamine phosphate transferase 2	PIGG	Homo sapiens	108.173	8	8	1268109	186775
3358	1	Q5SYE7	Q5SYE7	NHS-like protein 1	NHSL1	Homo sapiens	170.668	8	8	443916	0
3359	1	Q5T013	Q5T013	Putative hydroxypyruvate isomerase	HYI	Homo sapiens	30.406	8	8	2796287	49051
3360	1	Q5T160	Q5T160	Probable arginine--tRNA ligase, mitochondrial	RARS2	Homo sapiens	65.505	8	8	638484	57345
3361	1	Q5T200	Q5T200	Zinc finger CCCH domain-containing protein 13	ZC3H13	Homo sapiens	196.64	8	8	1420662	0
3362	1	Q5T440	Q5T440	Putative transferase CAF17, mitochondrial	IBA57	Homo sapiens	38.156	8	8	1946611	250506
3363	1	Q5T5Y3	Q5T5Y3	Calmodulin-regulated spectrin-associated protein 1	CAMSAP1	Homo sapiens	177.974	8	8	393572	83475
3364	1	Q5TAQ9	Q5TAQ9	DDB1- and CUL4-associated factor 8	DCAF8	Homo sapiens	66.852	9	8	1509962	243952
3365	1	Q5VIR6	Q5VIR6	Vacuolar protein sorting-associated protein 53 homolog	VPS53	Homo sapiens	94.407	8	8	1309944	653620
3366	1	Q5VT06	Q5VT06	Centrosome-associated protein 350	CEP350	Homo sapiens	350.936	8	8	303950	0
3367	1	Q5VUA4	Q5VUA4	Zinc finger protein 318	ZNF318	Homo sapiens	251.117	8	8	933471	30546
3368	1	Q5VWQ0	Q5VWQ0	Lysine-specific demethylase 9	RSBN1	Homo sapiens	90.073	8	8	798384	272674
3369	1	Q5VZL5	Q5VZL5	Zinc finger MYM-type protein 4	ZMYM4	Homo sapiens	172.794	8	8	726092	100139
3370	1	Q5XXA6	Q5XXA6	Anoctamin-1	ANO1	Homo sapiens	114.079	8	8	654073	0
3371	1	Q643R3	Q643R3	Lysophospholipid acyltransferase LPCAT4	LPCAT4	Homo sapiens	57.218	8	8	1342873	185631
3372	1	Q687X5	Q687X5	Metalloreductase STEAP4	STEAP4	Homo sapiens	51.98	8	8	1467026	3211376
3373	1	Q6DT37	Q6DT37	Serine/threonine-protein kinase MRCK gamma	CDC42BPG	Homo sapiens	172.461	8	8	364208	0
3374	1	Q6IC98	Q6IC98	GRAM domain-containing protein 4	GRAMD4	Homo sapiens	66.405	8	8	1308619	1246705
3375	1	Q6ICL3	Q6ICL3	Transport and Golgi organization protein 2 homolog	TANGO2	Homo sapiens	30.937	8	8	2864129	2677378
3376	1	Q6NSJ0	Q6NSJ0	Myogenesis-regulating glycosidase	MYORG	Homo sapiens	81.089	8	8	561903	0
3377	1	Q6NXG1	Q6NXG1	Epithelial splicing regulatory protein 1	ESRP1	Homo sapiens	75.583	9	8	2964202	0
3378	1	Q6NY19	Q6NY19	KN motif and ankyrin repeat domain-containing protein 3	KANK3	Homo sapiens	85.892	8	8	1095856	0
3379	1	Q6P1N9	Q6P1N9	Deoxyribonuclease TATDN1	TATDN1	Homo sapiens	33.6	8	8	2061230	605773
3380	1	Q6P4R8	Q6P4R8	Nuclear factor related to kappa-B-binding protein	NFRKB	Homo sapiens	139.004	8	8	355491	0
3381	1	Q6PJG6	Q6PJG6	BRCA1-associated ATM activator 1	BRAT1	Homo sapiens	88.121	8	8	377632	0
3382	1	Q6PML9	Q6PML9	Proton-coupled zinc antiporter SLC30A9, mitochondrial	SLC30A9	Homo sapiens	63.515	8	8	952323	156042
3383	1	Q6UWP7	Q6UWP7	Lysocardiolipin acyltransferase 1	LCLAT1	Homo sapiens	48.918	8	8	2288573	577132
3384	1	Q6YHU6	Q6YHU6	Thyroid adenoma-associated protein	THADA	Homo sapiens	219.61	8	8	339907	28069
3385	1	Q6ZMB0	Q6ZMB0	Acetylgalactosaminyl-O-glycosyl-glycoprotein beta-1,3-N-acetylglucosaminyltransferase	B3GNT6	Homo sapiens	42.749	8	8	1645916	0
3386	1	Q6ZNL6	Q6ZNL6	FYVE, RhoGEF and PH domain-containing protein 5	FGD5	Homo sapiens	159.892	8	8	376046	0
3387	1	Q6ZUM4	Q6ZUM4	Rho GTPase-activating protein 27	ARHGAP27	Homo sapiens	98.399	8	8	859938	1882152
3388	1	Q6ZVF9	Q6ZVF9	G protein-regulated inducer of neurite outgrowth 3	GPRIN3	Homo sapiens	82.441	8	8	1125274	64408
3389	1	Q6ZVM7	Q6ZVM7	TOM1-like protein 2	TOM1L2	Homo sapiens	55.557	8	8	3379630	107091
3390	1	Q76M96	Q76M96	Coiled-coil domain-containing protein 80	CCDC80	Homo sapiens	108.177	8	8	428168	431814

3391	1	Q7L1Q6	Q7L1Q6	eIF5-mimic protein 2	BZW1	Homo sapiens	48.044	8	8	6072081	4692510
3392	1	Q7L2J0	Q7L2J0	7SK snRNA methylphosphate capping enzyme	MEPCE	Homo sapiens	74.356	8	8	1497675	298249
3393	1	Q7L5Y9	Q7L5Y9	E3 ubiquitin-protein transferase MAEA	MAEA	Homo sapiens	45.29	8	8	710357	227377
3394	1	Q7Z2T5	Q7Z2T5	TRMT1-like protein	TRMT1L	Homo sapiens	81.748	8	8	778807	529916
3395	1	Q7Z404	Q7Z404	Transmembrane channel-like protein 4	TMC4	Homo sapiens	79.209	8	8	1406736	534295
3396	1	Q7Z569	Q7Z569	BRCA1-associated protein	BRAP	Homo sapiens	67.304	8	8	392222	295935
3397	1	Q7Z589	Q7Z589	BRCA2-interacting transcriptional repressor EMSY	EMSY	Homo sapiens	141.469	8	8	299353	174766
3398	1	Q7Z5N4	Q7Z5N4	Protein sidekick-1	SDK1	Homo sapiens	242.116	8	8	550880	0
3399	1	Q7Z7L7	Q7Z7L7	Protein zer-1 homolog	ZER1	Homo sapiens	88.171	8	8	349604	0
3400	1	Q86V21	Q86V21	Acetoacetyl-CoA synthetase	AACS	Homo sapiens	75.144	8	8	1927456	1585885
3401	1	Q86Y56	Q86Y56	Dynein axonemal assembly factor 5	DNAAF5	Homo sapiens	93.52	8	8	611178	80255
3402	1	Q86YB7	Q86YB7	Enoyl-CoA hydratase domain-containing protein 2, mitochondrial	ECHDC2	Homo sapiens	31.126	8	8	2804983	214123
3403	1	Q86YH6	Q86YH6	All trans-polyprenyl-diphosphate synthase PDSS2	PDSS2	Homo sapiens	44.129	8	8	793294	0
3404	1	Q8IUH4	Q8IUH4	Palmitoyltransferase ZDHHC13	ZDHHC13	Homo sapiens	70.862	8	8	1707183	300450
3405	1	Q8IXK2	Q8IXK2	Polypeptide N-acetylgalactosaminyltransferase 12	GALNT12	Homo sapiens	66.936	8	8	4157284	343572
3406	1	Q8IY33	Q8IY33	MICAL-like protein 2	MICALL2	Homo sapiens	97.503	8	8	935816	0
3407	1	Q8IY37	Q8IY37	Probable ATP-dependent RNA helicase DHX37	DHX37	Homo sapiens	129.546	8	8	869152	102617
3408	1	Q8N108	Q8N108	Mesoderm induction early response protein 1	MIER1	Homo sapiens	57.984	8	8	2057320	283411
3409	1	Q8N183	Q8N183	NADH dehydrogenase [ubiquinone] 1 alpha subcomplex assembly factor 2	NDUFAF2	Homo sapiens	19.855	8	8	5112685	677529
3410	1	Q8N1P7	Q8N1P7	Beta/gamma crystallin domain-containing protein 2	CRYBG2	Homo sapiens	177.916	8	8	336572	39611
3411	1	Q8N3X1	Q8N3X1	Formin-binding protein 4	FNBP4	Homo sapiens	110.267	8	8	505087	24610
3412	1	Q8N4Q0	Q8N4Q0	Prostaglandin reductase 3	PTGR3	Homo sapiens	40.141	8	8	2041413	37245
3413	1	Q8N5N7	Q8N5N7	39S ribosomal protein L50, mitochondrial	MRPL50	Homo sapiens	18.324	8	8	3011105	107849
3414	1	Q8N612	Q8N612	FHF complex subunit HOOK interacting protein 1B	FHIP1B	Homo sapiens	105.572	8	8	1060751	464270
3415	1	Q8N9B5	Q8N9B5	Junction-mediating and -regulatory protein	JMY	Homo sapiens	111.444	8	8	938988	50379
3416	1	Q8NBX0	Q8NBX0	Saccharopine dehydrogenase-like oxidoreductase	SCCPDH	Homo sapiens	47.154	8	8	8923868	3274027
3417	1	Q8NC60	Q8NC60	Nitric oxide-associated protein 1	NOA1	Homo sapiens	78.458	8	8	457778	0
3418	1	Q8NCW5	Q8NCW5	NAD(P)H-hydrate epimerase	NAXE	Homo sapiens	31.675	8	8	6815479	915281
3419	1	Q8ND04	Q8ND04	Nonsense-mediated mRNA decay factor SMG8	SMG8	Homo sapiens	109.684	8	8	685330	270502
3420	1	Q8NEY1	Q8NEY1	Neuron navigator 1	NAV1	Homo sapiens	202.475	8	8	3676857	24065
3421	1	Q8NFH8	Q8NFH8	RalBP1-associated Eps domain-containing protein 2	REPS2	Homo sapiens	71.534	8	8	898038	475889
3422	1	Q8NFI3	Q8NFI3	Cytosolic endo-beta-N-acetylglucosaminidase	ENGASE	Homo sapiens	83.989	8	8	825734	16890
3423	1	Q8NHV4	Q8NHV4	Protein NEDD1	NEDD1	Homo sapiens	71.967	8	8	1254826	77341
3424	1	Q8NI35	Q8NI35	InaD-like protein	PATJ	Homo sapiens	196.368	8	8	110011	118955
3425	1	Q8TAA9	Q8TAA9	Vang-like protein 1	VANGL1	Homo sapiens	59.975	8	8	2041198	205915
3426	1	Q8TB24	Q8TB24	Ras and Rab interactor 3	RIN3	Homo sapiens	107.854	8	8	346953	1238060
3427	1	Q8TDY2	Q8TDY2	RB1-inducible coiled-coil protein 1	RB1CC1	Homo sapiens	183.094	8	8	145390	107744
3428	1	Q8TEQ8	Q8TEQ8	GPI ethanolamine phosphate transferase 3	PIGO	Homo sapiens	118.701	8	8	767536	220324
3429	1	Q8WU90	Q8WU90	Zinc finger CCCH domain-containing protein 15	ZC3H15	Homo sapiens	48.605	8	8	3282666	1375707
3430	1	Q8WUF8	Q8WUF8	Cotranscriptional regulator FAM172A	FAM172A	Homo sapiens	47.973	8	8	892157	42170
3431	1	Q8WV41	Q8WV41	Sorting nexin-33	SNX33	Homo sapiens	65.265	8	8	805320	207271
3432	1	Q8WW22	Q8WW22	DnaJ homolog subfamily A member 4	DNAJA4	Homo sapiens	44.797	8	8	1438592	426061
3433	1	Q8WWN8	Q8WWN8	Arf-GAP with Rho-GAP domain, ANK repeat and PH domain-containing protein 3	ARAP3	Homo sapiens	169.847	8	8	260014	304485
3434	1	Q8WWY3	Q8WWY3	U4/U6 small nuclear ribonucleoprotein Prp31	PRPF31	Homo sapiens	55.456	8	8	2237093	1279724
3435	1	Q8WXI4	Q8WXI4	Acyl-coenzyme A thioesterase 11	ACOT11	Homo sapiens	68.493	8	8	1649616	305701
3436	1	Q8WXX5	Q8WXX5	DnaJ homolog subfamily C member 9	DNAJC9	Homo sapiens	29.907	8	8	2614722	2136647

3437	1	Q8WZ42	Q8WZ42	Titin	TTN	Homo sapiens	3816.111	8	8	774391	205091
3438	1	Q92506	Q92506	(3R)-3-hydroxyacyl-CoA dehydrogenase	HSD17B8	Homo sapiens	26.973	8	8	12752087	120922
3439	1	Q92574	Q92574	Hamartin	TSC1	Homo sapiens	129.769	8	8	291556	167411
3440	1	Q92610	Q92610	Zinc finger protein 592	ZNF592	Homo sapiens	137.531	8	8	480518	191745
3441	1	Q92743	Q92743	Serine protease HTRA1	HTRA1	Homo sapiens	51.287	8	8	1201599	2786181
3442	1	Q92766	Q92766	Ras-responsive element-binding protein 1	RREB1	Homo sapiens	181.424	8	8	452878	0
3443	1	Q92882	Q92882	Osteoclast-stimulating factor 1	OSTF1	Homo sapiens	23.787	8	8	1134170	2193338
3444	1	Q93033	Q93033	Immunoglobulin superfamily member 2	CD101	Homo sapiens	115.111	8	8	38259	587296
3445	1	Q93062	Q93062	RNA-binding protein with multiple splicing	RBPMS	Homo sapiens	21.801	9	8	4078939	39728
3446	1	Q969P0	Q969P0	Immunoglobulin superfamily member 8	IGSF8	Homo sapiens	65.035	8	8	2208472	118540
3447	1	Q969S9	Q969S9	Ribosome-releasing factor 2, mitochondrial	GFM2	Homo sapiens	86.604	8	8	806909	123512
3448	1	Q96A49	Q96A49	Synapse-associated protein 1	SYAP1	Homo sapiens	39.933	8	8	4383292	905134
3449	1	Q96AE7	Q96AE7	Tetratricopeptide repeat protein 17	TTC17	Homo sapiens	129.559	8	8	782399	106313
3450	1	Q96C86	Q96C86	m7GpppX diphosphatase	DCPS	Homo sapiens	38.608	8	8	4059912	1718868
3451	1	Q96CP6	Q96CP6	Protein Aster-A	GRAMD1A	Homo sapiens	80.682	8	8	51443	759119
3452	1	Q96DB5	Q96DB5	Regulator of microtubule dynamics protein 1	RMDN1	Homo sapiens	35.807	8	8	4257966	2073306
3453	1	Q96DP5	Q96DP5	Methionyl-tRNA formyltransferase, mitochondrial	MTFMT	Homo sapiens	43.832	8	8	947522	73405
3454	1	Q96DV4	Q96DV4	39S ribosomal protein L38, mitochondrial	MRPL38	Homo sapiens	44.595	8	8	2511881	420618
3455	1	Q96EK6	Q96EK6	Glucosamine 6-phosphate N-acetyltransferase	GNPNAT1	Homo sapiens	20.75	8	8	1411415	512326
3456	1	Q96ER9	Q96ER9	Mitochondrial potassium channel	CCDC51	Homo sapiens	45.81	8	8	992770	253414
3457	1	Q96EY8	Q96EY8	Corrinoid adenosyltransferase MMAB	MMAB	Homo sapiens	27.386	8	8	2535880	760659
3458	1	Q96HN2	Q96HN2	Adenosylhomocysteinase 3	AHCYL2	Homo sapiens	66.723	18	8	1643173	218795
3459	1	Q96HW7	Q96HW7	Integrator complex subunit 4	INTS4	Homo sapiens	108.171	8	8	399578	106814
3460	1	Q96II8	Q96II8	DISP complex protein LRCH3	LRCH3	Homo sapiens	86.085	8	8	15649469	7099987
3461	1	Q96JA1	Q96JA1	Leucine-rich repeats and immunoglobulin-like domains protein 1	LRIG1	Homo sapiens	119.113	9	8	436872	0
3462	1	Q96KN2	Q96KN2	Beta-Ala-His dipeptidase	CNDP1	Homo sapiens	56.691	8	8	444244	817427
3463	1	Q96ME1	Q96ME1	F-box/LRR-repeat protein 18	FBXL18	Homo sapiens	78.919	8	8	573888	114739
3464	1	Q96NY7	Q96NY7	Chloride intracellular channel protein 6	CLIC6	Homo sapiens	73.013	8	8	1342236	0
3465	1	Q96PD5	Q96PD5	N-acetylmuramoyl-L-alanine amidase	PGLYRP2	Homo sapiens	62.217	8	8	4060268	6833662
3466	1	Q96PY6	Q96PY6	Serine/threonine-protein kinase Nek1	NEK1	Homo sapiens	142.829	8	8	810562	23472
3467	1	Q96PZ0	Q96PZ0	Pseudouridylate synthase 7 homolog	PUS7	Homo sapiens	75.036	8	8	1128545	260360
3468	1	Q96RK0	Q96RK0	Protein capicua homolog	CIC	Homo sapiens	163.821	8	8	709212	0
3469	1	Q96RT7	Q96RT7	Gamma-tubulin complex component 6	TUBGCP6	Homo sapiens	200.5	8	8	513656	98354
3470	1	Q96RT8	Q96RT8	Gamma-tubulin complex component 5	TUBGCP5	Homo sapiens	118.322	8	8	312470	35864
3471	1	Q96TC7	Q96TC7	Regulator of microtubule dynamics protein 3	RMDN3	Homo sapiens	52.117	8	8	3498240	2344245
3472	1	Q99439	Q99439	Calponin-2	CNN2	Homo sapiens	33.699	8	8	4815634	15275904
3473	1	Q99497	Q99497	Parkinson disease protein 7	PARK7	Homo sapiens	19.892	8	8	55223174	6434625
3474	1	Q99519	Q99519	Sialidase-1	NEU1	Homo sapiens	45.468	8	8	758766	1323901
3475	1	Q99653	Q99653	Calcineurin B homologous protein 1	CHP1	Homo sapiens	22.456	8	8	2240631	2551584
3476	1	Q99836	Q99836	Myeloid differentiation primary response protein MyD88	MYD88	Homo sapiens	33.232	8	8	2723414	1994854
3477	1	Q9BQA1	Q9BQA1	Methylosome protein 50	WDR77	Homo sapiens	36.725	8	8	4511205	2406004
3478	1	Q9BQG2	Q9BQG2	NAD-capped RNA hydrolase NUDT12	NUDT12	Homo sapiens	52.075	8	8	1296695	0
3479	1	Q9BRG1	Q9BRG1	Vacuolar protein-sorting-associated protein 25	VPS25	Homo sapiens	20.748	8	8	4887570	1880191
3480	1	Q9BRJ7	Q9BRJ7	Tudor-interacting repair regulator protein	NUDT16L1	Homo sapiens	23.339	8	8	830222	16196
3481	1	Q9BSH4	Q9BSH4	Translational activator of cytochrome c oxidase 1	TACO1	Homo sapiens	32.478	8	8	1539366	329373
3482	1	Q9BSH5	Q9BSH5	Haloacid dehalogenase-like hydrolase domain-containing protein 3	HDHD3	Homo sapiens	28.001	8	8	3157778	231276
3483	1	Q9BTE6	Q9BTE6	Alanyl-tRNA editing protein Aarsd1	AARSD1	Homo sapiens	45.48	8	8	1751988	401443
3484	1	Q9BV38	Q9BV38	WD repeat-containing protein 18	WDR18	Homo sapiens	47.405	8	8	1488765	1033318

3485	1	Q9BXF6	Q9BXF6	Rab11 family-interacting protein 5	RAB11FIP5	Homo sapiens	70.417	8	8	974648	76394
3486	1	Q9BXP2	Q9BXP2	Solute carrier family 12 member 9	SLC12A9	Homo sapiens	96.111	8	8	615895	752676
3487	1	Q9BYE9	Q9BYE9	Cadherin-related family member 2	CDHR2	Homo sapiens	141.544	8	8	261990	0
3488	1	Q9BYM8	Q9BYM8	RanBP-type and C3HC4-type zinc finger-containing protein 1	RBCK1	Homo sapiens	57.573	8	8	1235772	633806
3489	1	Q9BZG1	Q9BZG1	Ras-related protein Rab-34	RAB34	Homo sapiens	29.045	8	8	3438364	649902
3490	1	Q9C0D3	Q9C0D3	Protein zyg-11 homolog B	ZYG11B	Homo sapiens	83.923	8	8	658174	78357
3491	1	Q9GZL7	Q9GZL7	Ribosome biogenesis protein WDR12	WDR12	Homo sapiens	47.709	8	8	1539177	293455
3492	1	Q9GZP4	Q9GZP4	PITH domain-containing protein 1	PITHD1	Homo sapiens	24.177	8	8	4094309	1315823
3493	1	Q9GZT3	Q9GZT3	SRA stem-loop-interacting RNA-binding protein, mitochondrial	SLIRP	Homo sapiens	12.347	8	8	9149034	1356425
3494	1	Q9GZZ1	Q9GZZ1	N-alpha-acetyltransferase 50	NAA50	Homo sapiens	19.399	8	8	3252284	1165419
3495	1	Q9H008	Q9H008	Phospholysine phosphohistidine inorganic pyrophosphate phosphatase	LHPP	Homo sapiens	29.163	8	8	3213652	641257
3496	1	Q9H0B8	Q9H0B8	Cysteine-rich secretory protein LCCL domain-containing 2	CRISPLD2	Homo sapiens	55.921	9	8	366725	1269600
3497	1	Q9H0E3	Q9H0E3	Histone deacetylase complex subunit SAP130	SAP130	Homo sapiens	110.325	8	8	1113687	142308
3498	1	Q9H0Q0	Q9H0Q0	CYFIP-related Rac1 interactor A	CYRIA	Homo sapiens	37.314	8	8	1601344	2149805
3499	1	Q9H0V9	Q9H0V9	VIP36-like protein	LMAN2L	Homo sapiens	39.712	8	8	1831994	551494
3500	1	Q9H0W8	Q9H0W8	Nonsense-mediated mRNA decay factor SMG9	SMG9	Homo sapiens	57.653	8	8	844906	178824
3501	1	Q9H0X4	Q9H0X4	Protein FAM234A	FAM234A	Homo sapiens	59.661	8	8	1481169	56258
3502	1	Q9H1E5	Q9H1E5	Thioredoxin-related transmembrane protein 4	TMX4	Homo sapiens	38.951	8	8	2773349	1714591
3503	1	Q9H1Z4	Q9H1Z4	WD repeat-containing protein 13	WDR13	Homo sapiens	53.695	8	8	1821653	362822
3504	1	Q9H329	Q9H329	Band 4.1-like protein 4B	EPB41L4B	Homo sapiens	99.715	8	8	972233	0
3505	1	Q9H3N1	Q9H3N1	Thioredoxin-related transmembrane protein 1	TMX1	Homo sapiens	31.789	8	8	13030612	13674216
3506	1	Q9H3P2	Q9H3P2	Negative elongation factor A	NELFA	Homo sapiens	57.276	8	8	1165601	338415
3507	1	Q9H4Z3	Q9H4Z3	mRNA (2'-O-methyladenosine-N(6)-)-methyltransferase	PCIF1	Homo sapiens	80.671	8	8	845460	708252
3508	1	Q9H5V8	Q9H5V8	CUB domain-containing protein 1	CDCP1	Homo sapiens	92.934	8	8	672476	760744
3509	1	Q9H6T0	Q9H6T0	Epithelial splicing regulatory protein 2	ESRP2	Homo sapiens	78.401	8	8	1099250	81440
3510	1	Q9H6U6	Q9H6U6	BCAS3 microtubule associated cell migration factor	BCAS3	Homo sapiens	101.237	8	8	1773926	494983
3511	1	Q9H8H2	Q9H8H2	Probable ATP-dependent RNA helicase DDX31	DDX31	Homo sapiens	94.088	8	8	346483	5626
3512	1	Q9H8Y5	Q9H8Y5	Ankyrin repeat and zinc finger domain-containing protein 1	ANKZF1	Homo sapiens	80.927	8	8	330906	316564
3513	1	Q9H993	Q9H993	Damage-control phosphatase ARMT1	ARMT1	Homo sapiens	51.172	8	8	735795	463363
3514	1	Q9HB40	Q9HB40	Retinoid-inducible serine carboxypeptidase	SCPEP1	Homo sapiens	50.829	8	8	2297182	2393983
3515	1	Q9HBL8	Q9HBL8	NmrA-like family domain-containing protein 1	NMRAL1	Homo sapiens	33.344	8	8	986319	226240
3516	1	Q9HD33	Q9HD33	39S ribosomal protein L47, mitochondrial	MRPL47	Homo sapiens	29.451	8	8	2559068	488326
3517	1	Q9HD40	Q9HD40	O-phosphoseryl-tRNA(Sec) selenium transferase	SEPSECS	Homo sapiens	55.726	8	8	1102021	307291
3518	1	Q9NPF5	Q9NPF5	DNA methyltransferase 1-associated protein 1	DMAP1	Homo sapiens	52.993	8	8	703422	245748
3519	1	Q9NPL8	Q9NPL8	Complex I assembly factor TIMMDC1, mitochondrial	TIMMDC1	Homo sapiens	32.176	8	8	1070574	165994
3520	1	Q9NQX7	Q9NQX7	Integral membrane protein 2C	ITM2C	Homo sapiens	30.222	8	8	2128998	0
3521	1	Q9NRA8	Q9NRA8	Eukaryotic translation initiation factor 4E transporter	EIF4ENIF1	Homo sapiens	108.203	8	8	707482	44961
3522	1	Q9NRG9	Q9NRG9	Aladin	AAAS	Homo sapiens	59.576	8	8	1327411	936602
3523	1	Q9NRZ7	Q9NRZ7	1-acyl-sn-glycerol-3-phosphate acyltransferase gamma	AGPAT3	Homo sapiens	43.38	8	8	2023927	1538941
3524	1	Q9NSC7	Q9NSC7	Alpha-N-acetylgalactosaminide alpha-2,6-sialyltransferase 1	ST6GALNAC1	Homo sapiens	68.565	8	8	3090420	0
3525	1	Q9NUI1	Q9NUI1	Peroxisomal 2,4-dienoyl-CoA reductase [(3E)-enoyl-CoA-producing]	DECR2	Homo sapiens	30.777	8	8	2930181	1129389
3526	1	Q9NUJ3	Q9NUJ3	T-complex protein 11-like protein 1	TCP11L1	Homo sapiens	57.035	8	8	2616342	15440
3527	1	Q9NV96	Q9NV96	Cell cycle control protein 50A	TMEM30A	Homo sapiens	40.686	9	8	974637	5276016
3528	1	Q9NVA2	Q9NVA2	Septin-11	SEPTIN11	Homo sapiens	49.399	13	8	12491723	2180276
3529	1	Q9NVH6	Q9NVH6	Trimethyllysine dioxygenase, mitochondrial	TMLHE	Homo sapiens	49.518	8	8	2406664	686769
3530	1	Q9NVT9	Q9NVT9	Armadillo repeat-containing protein 1	ARMC1	Homo sapiens	31.283	8	8	3025157	385062
3531	1	Q9NX63	Q9NX63	MICOS complex subunit MIC19	CHCHD3	Homo sapiens	26.152	8	8	7385854	2694376
3532	1	Q9NXA8	Q9NXA8	NAD-dependent protein deacylase sirtuin-5, mitochondrial	SIRT5	Homo sapiens	33.882	8	8	1800023	844062

3533	1	Q9NXD2	Q9NXD2	Myotubularin-related protein 10	MTMR10	Homo sapiens	88.273	8	8	665768	607771
3534	1	Q9NXN4	Q9NXN4	Ganglioside-induced differentiation-associated protein 2	GDAP2	Homo sapiens	56.224	8	8	629677	993349
3535	1	Q9NXW2	Q9NXW2	DnaJ homolog subfamily B member 12	DNAJB12	Homo sapiens	41.86	8	8	1167882	680008
3536	1	Q9NY47	Q9NY47	Voltage-dependent calcium channel subunit alpha-2/delta-2	CACNA2D2	Homo sapiens	129.82	8	8	760574	0
3537	1	Q9NYK5	Q9NYK5	39S ribosomal protein L39, mitochondrial	MRPL39	Homo sapiens	38.713	8	8	2993243	901632
3538	1	Q9NZ32	Q9NZ32	Actin-related protein 10	ACTR10	Homo sapiens	46.307	8	8	2130713	581310
3539	1	Q9NZK5	Q9NZK5	Adenosine deaminase 2	ADA2	Homo sapiens	58.934	8	8	843284	813755
3540	1	Q9NZQ3	Q9NZQ3	NCK-interacting protein with SH3 domain	NCKIPSD	Homo sapiens	78.961	8	8	600574	188144
3541	1	Q9NZW5	Q9NZW5	Protein PALS2	PALS2	Homo sapiens	61.118	9	8	1392862	303764
3542	1	Q9P1Y6	Q9P1Y6	PHD and RING finger domain-containing protein 1	PHRF1	Homo sapiens	178.671	8	8	713371	435821
3543	1	Q9P2L0	Q9P2L0	WD repeat-containing protein 35	WDR35	Homo sapiens	133.551	8	8	253536	0
3544	1	Q9P2T1	Q9P2T1	GMP reductase 2	GMPR2	Homo sapiens	37.873	9	8	1844298	776131
3545	1	Q9UBN7	Q9UBN7	Histone deacetylase 6	HDAC6	Homo sapiens	131.422	8	8	1243083	3219083
3546	1	Q9UBS8	Q9UBS8	E3 ubiquitin-protein ligase RNF14	RNF14	Homo sapiens	53.838	8	8	980470	168434
3547	1	Q9UDY4	Q9UDY4	DnaJ homolog subfamily B member 4	DNAJB4	Homo sapiens	37.807	8	8	5502873	947622
3548	1	Q9UGV2	Q9UGV2	Protein NDRG3	NDRG3	Homo sapiens	41.407	8	8	2824335	1432104
3549	1	Q9UHI8	Q9UHI8	A disintegrin and metalloproteinase with thrombospondin motifs 1	ADAMTS1	Homo sapiens	105.358	8	8	118152	1127179
3550	1	Q9UHJ6	Q9UHJ6	Sedoheptulokinase	SHPK	Homo sapiens	51.504	8	8	1945163	1055310
3551	1	Q9UJX6	Q9UJX6	Anaphase-promoting complex subunit 2	ANAPC2	Homo sapiens	93.829	8	8	459704	0
3552	1	Q9UK41	Q9UK41	Vacuolar protein sorting-associated protein 28 homolog	VPS28	Homo sapiens	25.422	8	8	1952268	692011
3553	1	Q9UKD2	Q9UKD2	mRNA turnover protein 4 homolog	MRTO4	Homo sapiens	27.56	8	8	2133702	678584
3554	1	Q9UKK9	Q9UKK9	ADP-sugar pyrophosphatase	NUDT5	Homo sapiens	24.329	8	8	9779294	7246101
3555	1	Q9UL15	Q9UL15	BAG family molecular chaperone regulator 5	BAG5	Homo sapiens	51.2	8	8	1906388	617788
3556	1	Q9ULC3	Q9ULC3	Ras-related protein Rab-23	RAB23	Homo sapiens	26.658	8	8	14833073	1075383
3557	1	Q9ULK4	Q9ULK4	Mediator of RNA polymerase II transcription subunit 23	MED23	Homo sapiens	156.473	8	8	550802	392546
3558	1	Q9ULP9	Q9ULP9	TBC1 domain family member 24	TBC1D24	Homo sapiens	62.922	8	8	1582990	783497
3559	1	Q9ULX6	Q9ULX6	A-kinase anchor protein 8-like	AKAP8L	Homo sapiens	71.639	8	8	679810	299825
3560	1	Q9ULZ3	Q9ULZ3	Apoptosis-associated speck-like protein containing a CARD	PYCARD	Homo sapiens	21.627	8	8	5248960	4405354
3561	1	Q9UMD9	Q9UMD9	Collagen alpha-1(XVII) chain	COL17A1	Homo sapiens	150.42	8	8	591202	0
3562	1	Q9UN19	Q9UN19	Dual adapter for phosphotyrosine and 3-phosphotyrosine and 3-phosphoinositide	DAPP1	Homo sapiens	32.192	8	8	18174	1021745
3563	1	Q9Y228	Q9Y228	TRAF3-interacting JNK-activating modulator	TRAF3IP3	Homo sapiens	63.626	8	8	363064	1124848
3564	1	Q9Y259	Q9Y259	Choline/ethanolamine kinase	CHKB	Homo sapiens	45.272	8	8	1936617	1013112
3565	1	Q9Y277	Q9Y277	Voltage-dependent anion-selective channel protein 3	VDAC3	Homo sapiens	30.66	8	8	24163534	10568169
3566	1	Q9Y2B0	Q9Y2B0	Protein canopy homolog 2	CNPY2	Homo sapiens	20.652	8	8	2810271	2372516
3567	1	Q9Y2H0	Q9Y2H0	Disks large-associated protein 4	DLGAP4	Homo sapiens	108.012	8	8	1738314	1036840
3568	1	Q9Y2K2	Q9Y2K2	Serine/threonine-protein kinase SIK3	SIK3	Homo sapiens	144.852	8	8	496257	0
3569	1	Q9Y2S2	Q9Y2S2	Lambda-crystallin homolog	CRYL1	Homo sapiens	35.417	8	8	10407999	1409205
3570	1	Q9Y2T2	Q9Y2T2	AP-3 complex subunit mu-1	AP3M1	Homo sapiens	46.938	9	8	2600392	1647608
3571	1	Q9Y2V7	Q9Y2V7	Conserved oligomeric Golgi complex subunit 6	COG6	Homo sapiens	73.279	8	8	1720823	1068123
3572	1	Q9Y2X7	Q9Y2X7	ARF GTPase-activating protein GIT1	GIT1	Homo sapiens	84.339	8	8	765412	259297
3573	1	Q9Y303	Q9Y303	N-acetylglucosamine-6-phosphate deacetylase	AMDHD2	Homo sapiens	43.748	8	8	1036666	505061
3574	1	Q9Y383	Q9Y383	Putative RNA-binding protein Luc7-like 2	LUC7L2	Homo sapiens	46.515	11	8	4311884	3904046
3575	1	Q9Y3A5	Q9Y3A5	Ribosome maturation protein SBDS	SBDS	Homo sapiens	28.764	8	8	10077677	1211762
3576	1	Q9Y3D9	Q9Y3D9	28S ribosomal protein S23, mitochondrial	MRPS23	Homo sapiens	21.769	8	8	3698670	681613
3577	1	Q9Y3M8	Q9Y3M8	StAR-related lipid transfer protein 13	STARD13	Homo sapiens	124.968	8	8	977827	44131
3578	1	Q9Y478	Q9Y478	5'-AMP-activated protein kinase subunit beta-1	PRKAB1	Homo sapiens	30.382	9	8	1974095	1336866
3579	1	Q9Y487	Q9Y487	V-type proton ATPase 116 kDa subunit a 2	ATP6V0A2	Homo sapiens	98.085	8	8	484760	618154

3580	1	Q9Y5J1	Q9Y5J1	U3 small nucleolar RNA-associated protein 18 homolog	UTP18	Homo sapiens	62.006	8	8	1125260	325501
3581	1	Q9Y624	Q9Y624	Junctional adhesion molecule A	F11R	Homo sapiens	32.583	8	8	4274312	2762128
3582	1	Q9Y6C9	Q9Y6C9	Mitochondrial carrier homolog 2	MTCH2	Homo sapiens	33.331	8	8	6173100	2914803
3583	1	Q9Y6E0	Q9Y6E0	Serine/threonine-protein kinase 24	STK24	Homo sapiens	49.309	13	8	4570348	3470086
3584	1	Q9Y6E2	Q9Y6E2	eIF5-mimic protein 1	BZW2	Homo sapiens	48.163	8	8	3341037	1494150
3585	1	Q9Y6I3	Q9Y6I3	Epsin-1	EPN1	Homo sapiens	60.293	9	8	2681331	708631
3586	1	Q9Y6I4	Q9Y6I4	Ubiquitin carboxyl-terminal hydrolase 3	USP3	Homo sapiens	58.896	8	8	423040	943395
3587	1	Q9Y6K9	Q9Y6K9	NF-kappa-B essential modulator	IKBKG	Homo sapiens	48.196	8	8	1555074	1174421
3588	1	A7KAX9	A7KAX9	Rho GTPase-activating protein 32	ARHGAP32	Homo sapiens	230.534	7	7	621646	0
3589	1	O00233	O00233	26S proteasome non-ATPase regulatory subunit 9	PSMD9	Homo sapiens	24.68	7	7	5816939	2312665
3590	1	O00400	O00400	Acetyl-coenzyme A transporter 1	SLC33A1	Homo sapiens	60.908	7	7	1781944	785325
3591	1	O14558	O14558	Heat shock protein beta-6	HSPB6	Homo sapiens	17.135	7	7	16744682	0
3592	1	O14562	O14562	Ubiquitin domain-containing protein UBFD1	UBFD1	Homo sapiens	33.382	7	7	2167848	878524
3593	1	O14787	O14787	Transportin-2	TNPO2	Homo sapiens	101.39	7	7	1078870	174407
3594	1	O14791	O14791	Apolipoprotein L1	APOL1	Homo sapiens	43.974	7	7	3300380	6894509
3595	1	O14815	O14815	Calpain-9	CAPN9	Homo sapiens	79.098	7	7	1161356	0
3596	1	O14874	O14874	[3-methyl-2-oxobutanoate dehydrogenase [lipoamide]] kinase, mitochondrial	BCKDK	Homo sapiens	46.36	7	7	10182117	586885
3597	1	O15145	O15145	Actin-related protein 2/3 complex subunit 3	ARPC3	Homo sapiens	20.546	7	7	12541375	10999171
3598	1	O15204	O15204	ADAM DEC1	ADAMDEC1	Homo sapiens	52.776	7	7	1070109	1515754
3599	1	O15226	O15226	NF-kappa-B-repressing factor	NKRF	Homo sapiens	77.673	7	7	366438	187801
3600	1	O15321	O15321	Transmembrane 9 superfamily member 1	TM9SF1	Homo sapiens	68.861	7	7	790899	743986
3601	1	O15397	O15397	Importin-8	IPO8	Homo sapiens	119.941	7	7	1231236	280706
3602	1	O15511	O15511	Actin-related protein 2/3 complex subunit 5	ARPC5	Homo sapiens	16.321	7	7	22495682	26278214
3603	1	O43306	O43306	Adenylate cyclase type 6	ADCY6	Homo sapiens	130.619	10	7	689680	23477
3604	1	O43464	O43464	Serine protease HTRA2, mitochondrial	HTRA2	Homo sapiens	48.841	8	7	2721002	617698
3605	1	O43665	O43665	Regulator of G-protein signaling 10	RGS10	Homo sapiens	21.21	7	7	3013527	432780
3606	1	O43708	O43708	Maleylacetoacetate isomerase	GSTZ1	Homo sapiens	24.21	7	7	3233306	689917
3607	1	O43745	O43745	Calcineurin B homologous protein 2	CHP2	Homo sapiens	22.454	7	7	669733	40466
3608	1	O43824	O43824	Putative GTP-binding protein 6	GTPBP6	Homo sapiens	56.896	7	7	861036	0
3609	1	O43854	O43854	EGF-like repeat and discoidin I-like domain-containing protein 3	EDIL3	Homo sapiens	53.767	7	7	434394	556381
3610	1	O60234	O60234	Glia maturation factor gamma	GMFG	Homo sapiens	16.801	8	7	1513144	19005686
3611	1	O60287	O60287	Nucleolar pre-ribosomal-associated protein 1	URB1	Homo sapiens	254.393	7	7	365854	0
3612	1	O60443	O60443	Gasdermin-E	GSDME	Homo sapiens	54.556	7	7	606803	151417
3613	1	O60493	O60493	Sorting nexin-3	SNX3	Homo sapiens	18.762	8	7	3973385	4549353
3614	1	O60518	O60518	Ran-binding protein 6	RANBP6	Homo sapiens	124.714	7	7	208162	0
3615	1	O60674	O60674	Tyrosine-protein kinase JAK2	JAK2	Homo sapiens	130.674	7	7	125029	349719
3616	1	O60711	O60711	Leupaxin	LPXN	Homo sapiens	43.332	7	7	1166884	1184963
3617	1	O60942	O60942	mRNA-capping enzyme	RNGTT	Homo sapiens	68.557	7	7	287906	147354
3618	1	O75140	O75140	GATOR complex protein DEPDC5	DEPDC5	Homo sapiens	181.264	7	7	224767	47205
3619	1	O75175	O75175	CCR4-NOT transcription complex subunit 3	CNOT3	Homo sapiens	81.874	7	7	1108862	214006
3620	1	O75208	O75208	Ubiquinone biosynthesis protein COQ9, mitochondrial	COQ9	Homo sapiens	35.508	7	7	2207764	64877
3621	1	O75368	O75368	Adapter SH3BGRL	SH3BGRL	Homo sapiens	12.772	7	7	52525574	9463890
3622	1	O75530	O75530	Polycomb protein EED	EED	Homo sapiens	50.197	7	7	629456	326241
3623	1	O75676	O75676	Ribosomal protein S6 kinase alpha-4	RPS6KA4	Homo sapiens	85.605	7	7	695894	161470
3624	1	O75718	O75718	Cartilage-associated protein	CRTAP	Homo sapiens	46.562	7	7	1628069	2655847
3625	1	O75787	O75787	Renin receptor	ATP6AP2	Homo sapiens	39.008	7	7	250826	1421425
3626	1	O75794	O75794	Cell division cycle protein 123 homolog	CDC123	Homo sapiens	39.135	7	7	727312	631680

3627	1	O75808	O75808	Calpain-15	CAPN15	Homo sapiens	117.315	7	7	438472	90604
3628	1	O94763	O94763	Unconventional prefoldin RPB5 interactor 1	URI1	Homo sapiens	59.835	7	7	676439	218534
3629	1	O94880	O94880	PHD finger protein 14	PHF14	Homo sapiens	106.987	7	7	759372	45353
3630	1	O94903	O94903	Pyridoxal phosphate homeostasis protein	PLPBP	Homo sapiens	30.344	7	7	4377475	2378188
3631	1	O95182	O95182	NADH dehydrogenase [ubiquinone] 1 alpha subcomplex subunit 7	NDUFA7	Homo sapiens	12.551	7	7	5743594	1111677
3632	1	O95249	O95249	Golgi SNAP receptor complex member 1	GOSR1	Homo sapiens	28.61	7	7	937174	84354
3633	1	O95251	O95251	Histone acetyltransferase KAT7	KAT7	Homo sapiens	70.643	7	7	711636	0
3634	1	O95292	O95292	Vesicle-associated membrane protein-associated protein B/C	VAPB	Homo sapiens	27.228	8	7	9710621	2185725
3635	1	O95302	O95302	Peptidyl-prolyl cis-trans isomerase FKBP9	FKBP9	Homo sapiens	63.085	9	7	2511863	1286065
3636	1	O95363	O95363	Phenylalanine--tRNA ligase, mitochondrial	FARS2	Homo sapiens	52.356	7	7	932568	113279
3637	1	O95391	O95391	Pre-mRNA-splicing factor SLU7	SLU7	Homo sapiens	68.388	7	7	458965	179672
3638	1	O95497	O95497	Pantetheinase	VNN1	Homo sapiens	57.011	8	7	0	2865350
3639	1	O95498	O95498	Pantetheine hydrolase VNN2	VNN2	Homo sapiens	58.502	7	7	191497	10806360
3640	1	O95785	O95785	Protein Wiz	WIZ	Homo sapiens	178.676	7	7	734053	31318
3641	1	O96008	O96008	Mitochondrial import receptor subunit TOM40 homolog	TOMM40	Homo sapiens	37.893	7	7	5267370	1914383
3642	1	O96019	O96019	Actin-like protein 6A	ACTL6A	Homo sapiens	47.46	10	7	4260118	1982485
3643	1	P00746	P00746	Complement factor D	CFD	Homo sapiens	27.032	7	7	6264730	15532572
3644	1	P01137	P01137	Transforming growth factor beta-1 proprotein	TGFB1	Homo sapiens	44.34	7	7	344035	1563153
3645	1	P01834	P01834	Immunoglobulin kappa constant	IGKC	Homo sapiens	11.764	7	7	500246828	299798866
3646	1	P02743	P02743	Serum amyloid P-component	APCS	Homo sapiens	25.387	7	7	31427470	33935226
3647	1	P04049	P04049	RAF proto-oncogene serine/threonine-protein kinase	RAF1	Homo sapiens	73.053	8	7	1322045	617727
3648	1	P04632	P04632	Calpain small subunit 1	CAPNS1	Homo sapiens	28.315	7	7	22839125	6678182
3649	1	P05026	P05026	Sodium/potassium-transporting ATPase subunit beta-1	ATP1B1	Homo sapiens	35.061	7	7	66057473	6736987
3650	1	P06732	P06732	Creatine kinase M-type	CKM	Homo sapiens	43.1	7	7	1347428	16584
3651	1	P06748	P06748	Nucleophosmin	NPM1	Homo sapiens	32.576	7	7	34960143	17075012
3652	1	P07093	P07093	Glia-derived nexin	SERPINE2	Homo sapiens	44.005	7	7	1589251	224716
3653	1	P07711	P07711	Procathepsin L	CTSL	Homo sapiens	37.563	7	7	1410429	3048152
3654	1	P08123	P08123	Collagen alpha-2(I) chain	COL1A2	Homo sapiens	129.315	18	7	235020043	27971567
3655	1	P08246	P08246	Neutrophil elastase	ELANE	Homo sapiens	28.52	7	7	5491383	180208775
3656	1	P08294	P08294	Extracellular superoxide dismutase [Cu-Zn]	SOD3	Homo sapiens	25.851	7	7	10567276	200719
3657	1	P08319	P08319	All-trans-retinol dehydrogenase [NAD(+)] ADH4	ADH4	Homo sapiens	40.223	7	7	8734	817701
3658	1	P08574	P08574	Cytochrome c1, heme protein, mitochondrial	CYC1	Homo sapiens	35.423	7	7	7880987	2486729
3659	1	P08697	P08697	Alpha-2-antiplasmin	SERPINF2	Homo sapiens	54.565	8	7	4234510	24434460
3660	1	P0CAP1	P0CAP1	Myocardial zonula adherens protein	MYZAP	Homo sapiens	54.207	7	7	473198	0
3661	1	P10746	P10746	Uroporphyrinogen-III synthase	UROS	Homo sapiens	28.627	7	7	808633	421467
3662	1	P10768	P10768	S-formylglutathione hydrolase	ESD	Homo sapiens	31.463	7	7	46560379	11826769
3663	1	P12004	P12004	Proliferating cell nuclear antigen	PCNA	Homo sapiens	28.769	7	7	4830423	2937288
3664	1	P13073	P13073	Cytochrome c oxidase subunit 4 isoform 1, mitochondrial	COX4I1	Homo sapiens	19.578	7	7	103961850	14908996
3665	1	P14324	P14324	Farnesyl pyrophosphate synthase	FDPS	Homo sapiens	48.275	7	7	13998355	7433501
3666	1	P14902	P14902	Indoleamine 2,3-dioxygenase 1	IDO1	Homo sapiens	45.324	7	7	161700	2694654
3667	1	P15104	P15104	Glutamine synthetase	GLUL	Homo sapiens	42.066	7	7	825238	2153354
3668	1	P15169	P15169	Carboxypeptidase N catalytic chain	CPN1	Homo sapiens	52.286	9	7	1503873	5415518
3669	1	P15170	P15170	Eukaryotic peptide chain release factor GTP-binding subunit ERF3A	GSPT1	Homo sapiens	55.758	15	7	1754683	1053236
3670	1	P15735	P15735	Phosphorylase b kinase gamma catalytic chain, liver/testis isoform	PHKG2	Homo sapiens	46.442	8	7	353472	983902
3671	1	P15941	P15941	Mucin-1	MUC1	Homo sapiens	122.103	7	7	2651050	2216046
3672	1	P16070	P16070	CD44 antigen	CD44	Homo sapiens	81.539	7	7	7458190	7932791
3673	1	P16083	P16083	Ribosyldihydronicotinamide dehydrogenase [quinone]	NQO2	Homo sapiens	25.919	7	7	3038090	3155785
3674	1	P17252	P17252	Protein kinase C alpha type	PRKCA	Homo sapiens	76.753	7	7	2754353	328663

3675	1	P17540	P17540	Creatine kinase S-type, mitochondrial	CKMT2	Homo sapiens	47.507	7	7	625437	793227
3676	1	P18428	P18428	Lipopolysaccharide-binding protein	LBP	Homo sapiens	53.38	7	7	4726739	7103202
3677	1	P18621	P18621	60S ribosomal protein L17	RPL17	Homo sapiens	21.397	7	7	31561467	10364698
3678	1	P19086	P19086	Guanine nucleotide-binding protein G(z) subunit alpha	GNAZ	Homo sapiens	40.924	7	7	27751992	65840
3679	1	P19224	P19224	UDP-glucuronosyltransferase 1-6	UGT1A6	Homo sapiens	60.751	9	7	0	2644174
3680	1	P20338	P20338	Ras-related protein Rab-4A	RAB4A	Homo sapiens	24.387	7	7	3595795	584035
3681	1	P20594	P20594	Atrial natriuretic peptide receptor 2	NPR2	Homo sapiens	117.024	8	7	230589	0
3682	1	P20851	P20851	C4b-binding protein beta chain	C4BPB	Homo sapiens	28.357	7	7	1469208	22442403
3683	1	P21549	P21549	Alanine--glyoxylate aminotransferase	AGXT	Homo sapiens	43.01	7	7	118348	853890
3684	1	P22792	P22792	Carboxypeptidase N subunit 2	CPN2	Homo sapiens	60.557	7	7	3092688	9664302
3685	1	P24298	P24298	Alanine aminotransferase 1	GPT	Homo sapiens	54.636	7	7	1487641	0
3686	1	P24844	P24844	Myosin regulatory light polypeptide 9	MYL9	Homo sapiens	19.831	14	7	332216733	2088335
3687	1	P25092	P25092	Guanylyl cyclase C	GUCY2C	Homo sapiens	123.404	9	7	2454077	0
3688	1	P25098	P25098	Beta-adrenergic receptor kinase 1	GRK2	Homo sapiens	79.574	17	7	1661792	4460904
3689	1	P25398	P25398	40S ribosomal protein S12	RPS12	Homo sapiens	14.513	7	7	15954316	6316332
3690	1	P25440	P25440	Bromodomain-containing protein 2	BRD2	Homo sapiens	88.061	7	7	761007	88104
3691	1	P25787	P25787	Proteasome subunit alpha type-2	PSMA2	Homo sapiens	25.897	7	7	8174381	4525557
3692	1	P26927	P26927	Hepatocyte growth factor-like protein	MST1	Homo sapiens	80.32	16	7	564250	2653732
3693	1	P27540	P27540	Aryl hydrocarbon receptor nuclear translocator	ARNT	Homo sapiens	86.637	7	7	1473631	0
3694	1	P28715	P28715	DNA excision repair protein ERCC-5	ERCC5	Homo sapiens	133.109	7	7	346450	470073
3695	1	P29474	P29474	Nitric oxide synthase, endothelial	NOS3	Homo sapiens	133.275	8	7	451021	63279
3696	1	P29597	P29597	Non-receptor tyrosine-protein kinase TYK2	TYK2	Homo sapiens	133.65	7	7	186391	201049
3697	1	P30511	P30511	HLA class I histocompatibility antigen, alpha chain F	HLA-F	Homo sapiens	39.061	8	7	793973	737026
3698	1	P30626	P30626	Sorcin	SRI	Homo sapiens	21.674	7	7	8674978	2361976
3699	1	P31483	P31483	Cytotoxic granule associated RNA binding protein TIA1	TIA1	Homo sapiens	42.962	7	7	1127024	343375
3700	1	P31937	P31937	3-hydroxyisobutyrate dehydrogenase, mitochondrial	HIBADH	Homo sapiens	35.328	7	7	17562050	4264612
3701	1	P31997	P31997	Carcinoembryonic antigen-related cell adhesion molecule 8	CEACAM8	Homo sapiens	38.155	8	7	169237	21394637
3702	1	P32119	P32119	Peroxiredoxin-2	PRDX2	Homo sapiens	21.89	7	7	52266139	26828558
3703	1	P32322	P32322	Pyrroline-5-carboxylate reductase 1, mitochondrial	PYCR1	Homo sapiens	33.36	7	7	710912	895123
3704	1	P32418	P32418	Sodium/calcium exchanger 1	SLC8A1	Homo sapiens	108.548	7	7	1941925	281277
3705	1	P32519	P32519	ETS-related transcription factor Elf-1	ELF1	Homo sapiens	67.498	7	7	567551	333093
3706	1	P32929	P32929	Cystathionine gamma-lyase	CTH	Homo sapiens	44.509	7	7	1590980	2213589
3707	1	P32969	P32969	60S ribosomal protein L9	RPL9P9	Homo sapiens	21.863	7	7	20503331	7742695
3708	1	P33151	P33151	Cadherin-5	CDH5	Homo sapiens	87.528	8	7	1650680	1104650
3709	1	P33316	P33316	Deoxyuridine 5'-triphosphate nucleotidohydrolase, mitochondrial	DUT	Homo sapiens	26.564	7	7	1373644	370937
3710	1	P34096	P34096	Ribonuclease 4	RNASE4	Homo sapiens	16.841	7	7	1116867	530567
3711	1	P35613	P35613	Basigin	BSG	Homo sapiens	42.198	7	7	20656562	9281275
3712	1	P36404	P36404	ADP-ribosylation factor-like protein 2	ARL2	Homo sapiens	20.875	7	7	3081867	773222
3713	1	P36507	P36507	Dual specificity mitogen-activated protein kinase kinase 2	MAP2K2	Homo sapiens	44.424	7	7	1619964	1743412
3714	1	P36955	P36955	Pigment epithelium-derived factor	SERPINF1	Homo sapiens	46.31	10	7	7598926	8640984
3715	1	P36969	P36969	Phospholipid hydroperoxide glutathione peroxidase	GPX4	Homo sapiens	22.146	7	7	5250751	3773142
3716	1	P37173	P37173	TGF-beta receptor type-2	TGFBR2	Homo sapiens	64.568	7	7	447704	82737
3717	1	P39023	P39023	60S ribosomal protein L3	RPL3	Homo sapiens	46.112	8	7	17820522	7381867
3718	1	P39748	P39748	Flap endonuclease 1	FEN1	Homo sapiens	42.592	7	7	3470800	5537452
3719	1	P43353	P43353	Aldehyde dehydrogenase family 3 member B1	ALDH3B1	Homo sapiens	51.839	8	7	345875	1887311
3720	1	P45880	P45880	Voltage-dependent anion-selective channel protein 2	VDAC2	Homo sapiens	31.568	7	7	42956672	11101433
3721	1	P46199	P46199	Translation initiation factor IF-2, mitochondrial	MTIF2	Homo sapiens	81.32	7	7	1562964	383102
3722	1	P46926	P46926	Glucosamine-6-phosphate isomerase 1	GNPDA1	Homo sapiens	32.669	10	7	10562789	4870455

3723	1	P46937	P46937	Transcriptional coactivator YAP1	YAP1	Homo sapiens	54.463	7	7	3634066	523487
3724	1	P48200	P48200	Iron-responsive element-binding protein 2	IREB2	Homo sapiens	105.062	7	7	766277	164024
3725	1	P48307	P48307	Tissue factor pathway inhibitor 2	TFPI2	Homo sapiens	26.933	7	7	0	2531906
3726	1	P48426	P48426	Phosphatidylinositol 5-phosphate 4-kinase type-2 alpha	PIP4K2A	Homo sapiens	46.224	10	7	2715912	3006833
3727	1	P48507	P48507	Glutamate--cysteine ligase regulatory subunit	GCLM	Homo sapiens	30.726	7	7	1965562	7796930
3728	1	P48729	P48729	Casein kinase I isoform alpha	CSNK1A1	Homo sapiens	38.915	15	7	5106370	1092197
3729	1	P49005	P49005	DNA polymerase delta subunit 2	POLD2	Homo sapiens	51.288	7	7	793981	299726
3730	1	P49326	P49326	Flavin-containing monooxygenase 5	FMO5	Homo sapiens	60.22	7	7	1160459	24307
3731	1	P49662	P49662	Caspase-4	CASP4	Homo sapiens	43.263	9	7	428384	3197919
3732	1	P49796	P49796	Regulator of G-protein signaling 3	RGS3	Homo sapiens	132.338	7	7	294948	153026
3733	1	P50148	P50148	Guanine nucleotide-binding protein G(q) subunit alpha	GNAQ	Homo sapiens	42.143	7	7	4370698	2417380
3734	1	P50750	P50750	Cyclin-dependent kinase 9	CDK9	Homo sapiens	42.778	7	7	1362092	1050670
3735	1	P51148	P51148	Ras-related protein Rab-5C	RAB5C	Homo sapiens	23.481	7	7	19451617	7865682
3736	1	P51159	P51159	Ras-related protein Rab-27A	RAB27A	Homo sapiens	24.865	9	7	9668542	53032071
3737	1	P51571	P51571	Translocon-associated protein subunit delta	SSR4	Homo sapiens	19.001	7	7	8378397	5081302
3738	1	P51580	P51580	Thiopurine S-methyltransferase	TPMT	Homo sapiens	28.18	7	7	4114340	2497171
3739	1	P51687	P51687	Sulfite oxidase, mitochondrial	SUOX	Homo sapiens	60.283	7	7	2637222	381245
3740	1	P51690	P51690	Arylsulfatase L	ARSL	Homo sapiens	65.67	8	7	3369108	78053
3741	1	P51692	P51692	Signal transducer and activator of transcription 5B	STAT5B	Homo sapiens	89.865	22	7	1562864	2314375
3742	1	P52566	P52566	Rho GDP-dissociation inhibitor 2	ARHGDIB	Homo sapiens	22.991	7	7	7686420	57831888
3743	1	P52594	P52594	Arf-GAP domain and FG repeat-containing protein 1	AGFG1	Homo sapiens	58.261	7	7	1793522	2562315
3744	1	P53007	P53007	Tricarboxylate transport protein, mitochondrial	SLC25A1	Homo sapiens	34.012	7	7	10059785	2362470
3745	1	P53355	P53355	Death-associated protein kinase 1	DAPK1	Homo sapiens	160.047	7	7	434163	249186
3746	1	P53365	P53365	Arfaptin-2	ARFIP2	Homo sapiens	37.855	7	7	1134258	354284
3747	1	P53990	P53990	IST1 homolog	IST1	Homo sapiens	39.749	7	7	9627173	4855044
3748	1	P54687	P54687	Branched-chain-amino-acid aminotransferase, cytosolic	BCAT1	Homo sapiens	42.968	7	7	487585	832948
3749	1	P54725	P54725	UV excision repair protein RAD23 homolog A	RAD23A	Homo sapiens	39.609	7	7	4127024	1482421
3750	1	P54727	P54727	UV excision repair protein RAD23 homolog B	RAD23B	Homo sapiens	43.172	7	7	12709841	5411438
3751	1	P55199	P55199	RNA polymerase II elongation factor ELL	ELL	Homo sapiens	68.263	7	7	554383	175566
3752	1	P55210	P55210	Caspase-7	CASP7	Homo sapiens	34.275	7	7	3238956	898715
3753	1	P55795	P55795	Heterogeneous nuclear ribonucleoprotein H2	HNRNPH2	Homo sapiens	49.264	7	7	5988699	4407069
3754	1	P57735	P57735	Ras-related protein Rab-25	RAB25	Homo sapiens	23.493	7	7	4912448	600405
3755	1	P57764	P57764	Gasdermin-D	GSDMD	Homo sapiens	52.802	7	7	3847460	4860453
3756	1	P60981	P60981	Destrin	DSTN	Homo sapiens	18.506	7	7	160995216	7841394
3757	1	P61081	P61081	NEDD8-conjugating enzyme Ubc12	UBE2M	Homo sapiens	20.899	7	7	4093951	4513561
3758	2	P61326;Q96A72	P61326	Protein mago nashi homolog	MAGOH	Homo sapiens	17.163	7	7	4111026	7497183
3759	1	P61421	P61421	V-type proton ATPase subunit d 1	ATP6V0D1	Homo sapiens	40.329	7	7	4762501	15890493
3760	1	P61626	P61626	Lysozyme C	LYZ	Homo sapiens	16.537	7	7	19025543	344051065
3761	1	P61758	P61758	Prefoldin subunit 3	VBP1	Homo sapiens	22.624	7	7	2781446	1312657
3762	1	P61962	P61962	DDB1- and CUL4-associated factor 7	DCAF7	Homo sapiens	38.925	7	7	1598325	579153
3763	1	P62888	P62888	60S ribosomal protein L30	RPL30	Homo sapiens	12.784	7	7	29310796	9864950
3764	1	P62937	P62937	Peptidyl-prolyl cis-trans isomerase A	PPIA	Homo sapiens	18.013	8	7	343995938	223456560
3765	1	P62942	P62942	Peptidyl-prolyl cis-trans isomerase FKBP1A	FKBP1A	Homo sapiens	11.95	7	7	31558271	17770463
3766	1	P62995	P62995	Transformer-2 protein homolog beta	TRA2B	Homo sapiens	33.664	7	7	2950085	515106
3767	1	P69905	P69905	Hemoglobin subunit alpha	HBA2	Homo sapiens	15.257	10	7	2230467796	1380695605
3768	1	P82914	P82914	28S ribosomal protein S15, mitochondrial	MRPS15	Homo sapiens	29.842	7	7	2486827	894600
3769	1	P84095	P84095	Rho-related GTP-binding protein RhoG	RHOG	Homo sapiens	21.308	7	7	2093366	10390062
3770	1	P85037	P85037	Forkhead box protein K1	FOXK1	Homo sapiens	75.459	7	7	1198279	270295

3771	1	Q00169	Q00169	Phosphatidylinositol transfer protein alpha isoform	PITPNA	Homo sapiens	31.806	7	7	2359425	1539695
3772	1	Q01201	Q01201	Transcription factor RelB	RELB	Homo sapiens	62.134	7	7	175554	327609
3773	1	Q01543	Q01543	Friend leukemia integration 1 transcription factor	FLI1	Homo sapiens	50.983	8	7	117555	482156
3774	1	Q01844	Q01844	RNA-binding protein EWS	EWSR1	Homo sapiens	68.481	7	7	12848949	6702207
3775	1	Q02083	Q02083	N-acylethanolamine-hydrolyzing acid amidase	NAAA	Homo sapiens	40.066	7	7	1055713	0
3776	1	Q02750	Q02750	Dual specificity mitogen-activated protein kinase kinase 1	MAP2K1	Homo sapiens	43.438	10	7	1201540	1466524
3777	1	Q03135	Q03135	Caveolin-1	CAV1	Homo sapiens	20.472	8	7	6171963	147574
3778	1	Q03405	Q03405	Urokinase plasminogen activator surface receptor	PLAUR	Homo sapiens	36.979	7	7	58353	1996986
3779	1	Q04721	Q04721	Neurogenic locus notch homolog protein 2	NOTCH2	Homo sapiens	265.41	9	7	593203	360786
3780	1	Q04726	Q04726	Transducin-like enhancer protein 3	TLE3	Homo sapiens	83.417	10	7	714335	631491
3781	1	Q06787	Q06787	Fragile X messenger ribonucleoprotein 1	FMR1	Homo sapiens	71.176	7	7	626166	110059
3782	1	Q07507	Q07507	Dermatopontin	DPT	Homo sapiens	24.006	7	7	8296353	201115
3783	1	Q08345	Q08345	Epithelial discoidin domain-containing receptor 1	DDR1	Homo sapiens	101.127	7	7	941921	0
3784	1	Q08AF3	Q08AF3	Schlafen family member 5	SLFN5	Homo sapiens	101.056	8	7	927102	756644
3785	1	Q09472	Q09472	Histone acetyltransferase p300	EP300	Homo sapiens	264.167	11	7	605880	348126
3786	1	Q0VDG4	Q0VDG4	Secernin-3	SCRN3	Homo sapiens	48.545	7	7	1862189	456296
3787	1	Q10588	Q10588	ADP-ribosyl cyclase/cyclic ADP-ribose hydrolase 2	BST1	Homo sapiens	35.724	7	7	1468086	6157294
3788	1	Q12800	Q12800	Alpha-globin transcription factor CP2	TFCP2	Homo sapiens	57.256	10	7	685856	528006
3789	1	Q12860	Q12860	Contactin-1	CNTN1	Homo sapiens	113.322	17	7	779142	0
3790	1	Q13114	Q13114	TNF receptor-associated factor 3	TRAF3	Homo sapiens	64.49	7	7	346245	220790
3791	1	Q13144	Q13144	Translation initiation factor eIF-2B subunit epsilon	EIF2B5	Homo sapiens	80.381	7	7	2093537	853099
3792	1	Q13151	Q13151	Heterogeneous nuclear ribonucleoprotein A0	HNRNPA0	Homo sapiens	30.841	7	7	7335473	2746005
3793	1	Q13153	Q13153	Serine/threonine-protein kinase PAK 1	PAK1	Homo sapiens	60.646	12	7	2292399	559440
3794	1	Q13303	Q13303	Voltage-gated potassium channel subunit beta-2	KCNAB2	Homo sapiens	41	7	7	353580	1943372
3795	1	Q13362	Q13362	Serine/threonine-protein phosphatase 2A 56 kDa regulatory subunit gamma isoform	PPP2R5C	Homo sapiens	61.059	7	7	968075	158780
3796	1	Q13367	Q13367	AP-3 complex subunit beta-2	AP3B2	Homo sapiens	119.06	7	7	346249	0
3797	1	Q13371	Q13371	Phosducin-like protein	PDCL	Homo sapiens	34.281	7	7	856506	735440
3798	1	Q13426	Q13426	DNA repair protein XRCC4	XRCC4	Homo sapiens	38.289	7	7	549281	525937
3799	1	Q13427	Q13427	Peptidyl-prolyl cis-trans isomerase G	PPIG	Homo sapiens	88.617	7	7	1546162	953922
3800	1	Q13444	Q13444	Disintegrin and metalloproteinase domain-containing protein 15	ADAM15	Homo sapiens	92.961	7	7	742003	143864
3801	1	Q13485	Q13485	Mothers against decapentaplegic homolog 4	SMAD4	Homo sapiens	60.44	7	7	1110698	75647
3802	1	Q13501	Q13501	Sequestosome-1	SQSTM1	Homo sapiens	47.689	7	7	1601753	4497433
3803	1	Q13555	Q13555	Calcium/calmodulin-dependent protein kinase type II subunit gamma	CAMK2G	Homo sapiens	62.61	16	7	8140423	1751553
3804	1	Q13572	Q13572	Inositol-tetrakisphosphate 1-kinase	ITPK1	Homo sapiens	45.621	7	7	2542036	974512
3805	1	Q13614	Q13614	Myotubularin-related protein 2	MTMR2	Homo sapiens	73.38	7	7	1623555	30083
3806	1	Q13825	Q13825	Methylglutaconyl-CoA hydratase, mitochondrial	AUH	Homo sapiens	35.61	7	7	4596218	1035501
3807	1	Q13838	Q13838	Spliceosome RNA helicase DDX39B	DDX39B	Homo sapiens	48.993	16	7	3132850	2283636
3808	1	Q14011	Q14011	Cold-inducible RNA-binding protein	CIRBP	Homo sapiens	18.647	7	7	5359729	996467
3809	1	Q14012	Q14012	Calcium/calmodulin-dependent protein kinase type 1	CAMK1	Homo sapiens	41.337	9	7	931106	80541
3810	1	Q14142	Q14142	Tripartite motif-containing protein 14	TRIM14	Homo sapiens	49.772	7	7	597600	36546
3811	1	Q14257	Q14257	Reticulocalbin-2	RCN2	Homo sapiens	36.875	7	7	2426226	1043576
3812	1	Q14653	Q14653	Interferon regulatory factor 3	IRF3	Homo sapiens	47.218	7	7	726714	1658658
3813	1	Q14696	Q14696	LRP chaperone MESD	MESD	Homo sapiens	26.076	7	7	5793575	3310182
3814	1	Q14894	Q14894	Ketimine reductase mu-crystallin	CRYM	Homo sapiens	33.774	7	7	2691495	0
3815	1	Q15024	Q15024	Exosome complex component RRP42	EXOSC7	Homo sapiens	31.822	7	7	864570	27057
3816	1	Q15047	Q15047	Histone-lysine N-methyltransferase SETDB1	SETDB1	Homo sapiens	143.159	7	7	334719	71964
3817	1	Q15126	Q15126	Phosphomevalonate kinase	PMVK	Homo sapiens	21.993	7	7	4423416	1338794

3818	1	Q15349	Q15349	Ribosomal protein S6 kinase alpha-2	RPS6KA2	Homo sapiens	83.238	7	7	388664	318568
3819	1	Q15363	Q15363	Transmembrane emp24 domain-containing protein 2	TMED2	Homo sapiens	22.761	7	7	6140297	7672324
3820	1	Q15375	Q15375	Ephrin type-A receptor 7	EPHA7	Homo sapiens	112.099	8	7	871975	0
3821	1	Q15493	Q15493	Regucalcin	RGN	Homo sapiens	33.254	7	7	679597	0
3822	1	Q15691	Q15691	Microtubule-associated protein RP/EB family member 1	MAPRE1	Homo sapiens	30	9	7	6246544	1919719
3823	1	Q15738	Q15738	Sterol-4-alpha-carboxylate 3-dehydrogenase, decarboxylating	NSDHL	Homo sapiens	41.9	7	7	1626344	578589
3824	1	Q16625	Q16625	Occludin	OCLN	Homo sapiens	59.143	7	7	1604908	48664
3825	1	Q16850	Q16850	Lanosterol 14-alpha demethylase	CYP51A1	Homo sapiens	57.277	7	7	1890901	0
3826	1	Q2TAZ0	Q2TAZ0	Autophagy-related protein 2 homolog A	ATG2A	Homo sapiens	212.862	7	7	887184	92213
3827	1	Q32M88	Q32M88	Protein-glucosylgalactosylhydroxylysine glucosidase	PGGHG	Homo sapiens	80.654	7	7	408748	22814
3828	1	Q4KMQ1	Q4KMQ1	Taperin	TPRN	Homo sapiens	75.556	7	7	586286	0
3829	1	Q504Q3	Q504Q3	PAN2-PAN3 deadenylation complex catalytic subunit PAN2	PAN2	Homo sapiens	135.37	7	7	162322	96356
3830	1	Q53FA7	Q53FA7	Quinone oxidoreductase PIG3	TP53I3	Homo sapiens	35.537	7	7	3859268	1009403
3831	1	Q53GQ0	Q53GQ0	Very-long-chain 3-oxoacyl-CoA reductase	HSD17B12	Homo sapiens	34.325	7	7	10564154	8296815
3832	1	Q53H96	Q53H96	Pyrroline-5-carboxylate reductase 3	PYCR3	Homo sapiens	28.659	7	7	1441907	937729
3833	1	Q53HC9	Q53HC9	EARP and GARP complex-interacting protein 1	EIPR1	Homo sapiens	43.602	7	7	3101163	1102568
3834	1	Q58DX5	Q58DX5	Inactive N-acetylated-alpha-linked acidic dipeptidase-like protein 2	NAALADL2	Homo sapiens	88.684	7	7	773367	0
3835	1	Q58WW2	Q58WW2	DDB1- and CUL4-associated factor 6	DCAF6	Homo sapiens	96.293	7	7	5410768	0
3836	1	Q5JVF3	Q5JVF3	PCI domain-containing protein 2	PCID2	Homo sapiens	46.031	7	7	1340978	201582
3837	1	Q5KU26	Q5KU26	Collectin-12	COLEC12	Homo sapiens	81.517	7	7	902886	77378
3838	1	Q5SRE7	Q5SRE7	Phytanoyl-CoA dioxygenase domain-containing protein 1	PHYHD1	Homo sapiens	32.411	7	7	1287293	44819
3839	1	Q5SRH9	Q5SRH9	Tetratricopeptide repeat protein 39A	TTC39A	Homo sapiens	69.78	7	7	923946	0
3840	1	Q5T653	Q5T653	39S ribosomal protein L2, mitochondrial	MRPL2	Homo sapiens	33.302	7	7	1218618	393542
3841	1	Q5TC12	Q5TC12	ATP synthase mitochondrial F1 complex assembly factor 1	ATPAF1	Homo sapiens	36.436	7	7	1382781	53567
3842	1	Q5TF21	Q5TF21	Protein SOGA3	SOGA3	Homo sapiens	103.2	7	7	367763	0
3843	1	Q5TGY3	Q5TGY3	Transcription factor Gibbin	AHDC1	Homo sapiens	168.352	7	7	410346	0
3844	1	Q5VW32	Q5VW32	BRO1 domain-containing protein BROX	BROX	Homo sapiens	46.476	7	7	5058113	2477571
3845	1	Q5VWZ2	Q5VWZ2	Lysophospholipase-like protein 1	LYPLAL1	Homo sapiens	26.316	7	7	3841076	2104814
3846	1	Q6EMK4	Q6EMK4	Vasorin	VASN	Homo sapiens	71.713	7	7	1144683	425253
3847	1	Q6IAA8	Q6IAA8	Ragulator complex protein LAMTOR1	LAMTOR1	Homo sapiens	17.743	7	7	4594817	2371673
3848	1	Q6IN85	Q6IN85	Serine/threonine-protein phosphatase 4 regulatory subunit 3A	PPP4R3A	Homo sapiens	95.37	11	7	1596027	1008647
3849	1	Q6NUK4	Q6NUK4	Receptor expression-enhancing protein 3	REEP3	Homo sapiens	29.263	7	7	1871785	564203
3850	1	Q6P1Q0	Q6P1Q0	LETM1 domain-containing protein 1	LETMD1	Homo sapiens	41.791	7	7	1183588	91485
3851	1	Q6P3X3	Q6P3X3	Tetratricopeptide repeat protein 27	TTC27	Homo sapiens	96.632	7	7	734817	54416
3852	1	Q6PKC3	Q6PKC3	Thioredoxin domain-containing protein 11	TXNDC11	Homo sapiens	110.53	7	7	403969	91230
3853	1	Q6T4R5	Q6T4R5	Actin remodeling regulator NHS	NHS	Homo sapiens	179.138	7	7	489781	0
3854	1	Q6UWW8	Q6UWW8	Carboxylesterase 3	CES3	Homo sapiens	62.283	7	7	1936977	0
3855	1	Q6V1X1	Q6V1X1	Dipeptidyl peptidase 8	DPP8	Homo sapiens	103.359	7	7	607026	227742
3856	1	Q6ZSR9	Q6ZSR9	Uncharacterized protein FLJ45252	-	Homo sapiens	37.976	7	7	980789	434820
3857	1	Q6ZWT7	Q6ZWT7	Lysophospholipid acyltransferase 2	MBOAT2	Homo sapiens	59.527	7	7	898225	1539981
3858	1	Q70EL4	Q70EL4	Ubiquitin carboxyl-terminal hydrolase 43	USP43	Homo sapiens	122.811	7	7	196284	160720
3859	1	Q7L1T6	Q7L1T6	Cytochrome b5 reductase 4	CYB5R4	Homo sapiens	59.472	7	7	181764	763602
3860	1	Q7L1W4	Q7L1W4	Volume-regulated anion channel subunit LRRC8D	LRRC8D	Homo sapiens	98.2	7	7	277964	187658
3861	1	Q7L592	Q7L592	Protein arginine methyltransferase NDUFAF7, mitochondrial	NDUFAF7	Homo sapiens	49.238	7	7	734926	75166
3862	1	Q7L5N1	Q7L5N1	COP9 signalosome complex subunit 6	COPS6	Homo sapiens	36.162	7	7	4595171	1701891
3863	1	Q7L5N7	Q7L5N7	Lysophosphatidylcholine acyltransferase 2	LPCAT2	Homo sapiens	60.21	7	7	1848571	8094906
3864	1	Q7Z3E5	Q7Z3E5	LisH domain-containing protein ARMC9	ARMC9	Homo sapiens	91.819	7	7	636771	0
3865	1	Q7Z4W1	Q7Z4W1	L-xylulose reductase	DCXR	Homo sapiens	25.911	7	7	4559082	1200817

3866	1	Q7Z7K6	Q7Z7K6	Centromere protein V	CENPV	Homo sapiens	29.947	7	7	2677081	39429
3867	1	Q86U28	Q86U28	Iron-sulfur cluster assembly 2 homolog, mitochondrial	ISCA2	Homo sapiens	16.476	7	7	973350	280130
3868	1	Q86U38	Q86U38	Nucleolar protein 9	NOP9	Homo sapiens	69.438	7	7	869552	364848
3869	1	Q86UA1	Q86UA1	Pre-mRNA-processing factor 39	PRPF39	Homo sapiens	78.431	7	7	800095	239761
3870	1	Q86UK7	Q86UK7	E3 ubiquitin-protein ligase ZNF598	ZNF598	Homo sapiens	98.638	7	7	626536	99664
3871	1	Q86VW0	Q86VW0	SEC14 domain and spectrin repeat-containing protein 1	SESTD1	Homo sapiens	79.348	7	7	1341628	0
3872	1	Q86VW2	Q86VW2	Rho guanine nucleotide exchange factor 25	ARHGEF25	Homo sapiens	63.843	8	7	1476243	0
3873	1	Q86W56	Q86W56	Poly(ADP-ribose) glycohydrolase	PARG	Homo sapiens	111.114	7	7	470057	195319
3874	1	Q86WJ1	Q86WJ1	Chromodomain-helicase-DNA-binding protein 1-like	CHD1L	Homo sapiens	100.999	7	7	337656	0
3875	1	Q86XL3	Q86XL3	Ankyrin repeat and LEM domain-containing protein 2	ANKLE2	Homo sapiens	104.117	7	7	383361	132119
3876	1	Q86XX4	Q86XX4	Extracellular matrix organizing protein FRAS1	FRAS1	Homo sapiens	443.222	7	7	158867	0
3877	1	Q86Y82	Q86Y82	Syntaxin-12	STX12	Homo sapiens	31.64	7	7	6921107	2648645
3878	1	Q8IV38	Q8IV38	Ankyrin repeat and MYND domain-containing protein 2	ANKMY2	Homo sapiens	49.299	7	7	941059	257761
3879	1	Q8IVF7	Q8IVF7	Formin-like protein 3	FMNL3	Homo sapiens	117.213	7	7	493354	24713
3880	1	Q8IVH8	Q8IVH8	Mitogen-activated protein kinase kinase kinase kinase 3	MAP4K3	Homo sapiens	101.316	7	7	422935	0
3881	1	Q8IVM0	Q8IVM0	Coiled-coil domain-containing protein 50	CCDC50	Homo sapiens	35.821	7	7	2516894	76384
3882	1	Q8IVN8	Q8IVN8	Somatomedin-B and thrombospondin type-1 domain-containing protein	SBSPON	Homo sapiens	29.609	7	7	2338785	0
3883	1	Q8IVS2	Q8IVS2	Malonyl-CoA-acyl carrier protein transacylase, mitochondrial	MCAT	Homo sapiens	42.961	7	7	938038	177291
3884	1	Q8IX18	Q8IX18	Probable ATP-dependent RNA helicase DHX40	DHX40	Homo sapiens	88.561	7	7	317192	115241
3885	1	Q8IXI1	Q8IXI1	Mitochondrial Rho GTPase 2	RHOT2	Homo sapiens	68.12	7	7	2862777	888087
3886	1	Q8IY81	Q8IY81	pre-rRNA 2'-O-ribose RNA methyltransferase FTSJ3	FTSJ3	Homo sapiens	96.557	7	7	1521395	136385
3887	1	Q8IYB3	Q8IYB3	Serine/arginine repetitive matrix protein 1	SRRM1	Homo sapiens	102.336	7	7	2491944	1242631
3888	1	Q8IYM9	Q8IYM9	E3 ubiquitin-protein ligase TRIM22	TRIM22	Homo sapiens	56.948	7	7	838548	609499
3889	1	Q8IYS1	Q8IYS1	Xaa-Arg dipeptidase	PM20D2	Homo sapiens	47.777	7	7	2322020	247428
3890	1	Q8IYS2	Q8IYS2	Uncharacterized protein KIAA2013	KIAA2013	Homo sapiens	69.157	7	7	900774	236256
3891	1	Q8IZA0	Q8IZA0	Dyslexia-associated protein KIAA0319-like protein	KIAA0319L	Homo sapiens	115.657	7	7	457006	149506
3892	1	Q8N0Z6	Q8N0Z6	Tetratricopeptide repeat protein 5	TTC5	Homo sapiens	48.926	7	7	820728	71735
3893	1	Q8N126	Q8N126	Cell adhesion molecule 3	CADM3	Homo sapiens	43.299	7	7	2980094	0
3894	1	Q8N2F6	Q8N2F6	Armadillo repeat-containing protein 10	ARMC10	Homo sapiens	37.539	7	7	1560936	0
3895	1	Q8N323	Q8N323	NXPE family member 1	NXPE1	Homo sapiens	63.181	7	7	3428462	0
3896	1	Q8N4N3	Q8N4N3	Kelch-like protein 36	KLHL36	Homo sapiens	69.898	7	7	707237	62200
3897	1	Q8N6T3	Q8N6T3	ADP-ribosylation factor GTPase-activating protein 1	ARFGAP1	Homo sapiens	44.666	7	7	6111982	1466883
3898	1	Q8NBN3	Q8NBN3	Transmembrane protein 87A	TMEM87A	Homo sapiens	63.43	7	7	1252109	617216
3899	1	Q8NCA5	Q8NCA5	Protein FAM98A	FAM98A	Homo sapiens	55.273	7	7	1637757	783607
3900	1	Q8NCM8	Q8NCM8	Cytoplasmic dynein 2 heavy chain 1	DYNC2H1	Homo sapiens	492.631	7	7	128045	0
3901	1	Q8NDH3	Q8NDH3	Probable aminopeptidase NPEPL1	NPEPL1	Homo sapiens	55.859	7	7	3107389	497064
3902	1	Q8NDZ4	Q8NDZ4	Divergent protein kinase domain 2A	DIPK2A	Homo sapiens	49.481	7	7	462902	471355
3903	1	Q8NE62	Q8NE62	Choline dehydrogenase, mitochondrial	CHDH	Homo sapiens	65.359	7	7	1449158	0
3904	1	Q8NEZ5	Q8NEZ5	F-box only protein 22	FBXO22	Homo sapiens	44.51	7	7	780327	384442
3905	1	Q8NFL0	Q8NFL0	UDP-GlcNAc:betaGal beta-1,3-N-acetylglucosaminyltransferase 7	B3GNT7	Homo sapiens	45.986	7	7	2673455	0
3906	1	Q8NFZ8	Q8NFZ8	Cell adhesion molecule 4	CADM4	Homo sapiens	42.784	7	7	2332421	48787
3907	1	Q8NHH9	Q8NHH9	Atlastin-2	ATL2	Homo sapiens	66.231	7	7	1480642	315757
3908	1	Q8TB03	Q8TB03	Uncharacterized protein CXorf38	CXorf38	Homo sapiens	36.672	7	7	552022	1244618
3909	1	Q8TB72	Q8TB72	Pumilio homolog 2	PUM2	Homo sapiens	114.218	7	7	419525	191855
3910	1	Q8TBX8	Q8TBX8	Phosphatidylinositol 5-phosphate 4-kinase type-2 gamma	PIP4K2C	Homo sapiens	47.3	7	7	1501127	1053754
3911	1	Q8TCG1	Q8TCG1	Protein CIP2A	CIP2A	Homo sapiens	102.184	7	7	31435	441879
3912	1	Q8TDN6	Q8TDN6	Ribosome biogenesis protein BRX1 homolog	BRIX1	Homo sapiens	41.403	7	7	1089614	322372
3913	1	Q8TE68	Q8TE68	Epidermal growth factor receptor kinase substrate 8-like protein 1	EPS8L1	Homo sapiens	80.251	7	7	493098	556392

3914	1	Q8TEA7	Q8TEA7	TBC domain-containing protein kinase-like protein	TBCK	Homo sapiens	100.679	7	7	482020	145072
3915	1	Q8TF46	Q8TF46	DIS3-like exonuclease 1	DIS3L	Homo sapiens	120.788	7	7	242231	288545
3916	1	Q8TF74	Q8TF74	WAS/WASL-interacting protein family member 2	WIPF2	Homo sapiens	46.288	7	7	1676693	69198
3917	1	Q8WTS6	Q8WTS6	Histone-lysine N-methyltransferase SETD7	SETD7	Homo sapiens	40.723	7	7	1963937	312166
3918	1	Q8WU79	Q8WU79	Stromal membrane-associated protein 2	SMAP2	Homo sapiens	46.785	7	7	1647229	2368697
3919	1	Q8WUA4	Q8WUA4	General transcription factor 3C polypeptide 2	GTF3C2	Homo sapiens	100.679	7	7	483285	205780
3920	1	Q8WUI4	Q8WUI4	Histone deacetylase 7	HDAC7	Homo sapiens	102.926	7	7	925563	260223
3921	1	Q8WW12	Q8WW12	PEST proteolytic signal-containing nuclear protein	PCNP	Homo sapiens	18.926	7	7	3493314	1419444
3922	1	Q8WWM9	Q8WWM9	Cytoglobin	CYGB	Homo sapiens	21.404	7	7	2533707	121067
3923	1	Q8WWW0	Q8WWW0	Ras association domain-containing protein 5	RASSF5	Homo sapiens	47.09	7	7	157536	1768857
3924	1	Q8WWZ8	Q8WWZ8	Oncoprotein-induced transcript 3 protein	OIT3	Homo sapiens	60.024	7	7	0	634663
3925	1	Q8WYP3	Q8WYP3	Ras and Rab interactor 2	RIN2	Homo sapiens	100.163	7	7	356987	0
3926	1	Q92604	Q92604	Acyl-CoA:lysophosphatidylglycerol acyltransferase 1	LPGAT1	Homo sapiens	43.089	7	7	613090	2660369
3927	1	Q92615	Q92615	La-related protein 4B	LARP4B	Homo sapiens	80.552	7	7	1129908	1189198
3928	1	Q92629	Q92629	Delta-sarcoglycan	SGCD	Homo sapiens	32.072	7	7	9534544	41107
3929	1	Q92817	Q92817	Envoplakin	EVPL	Homo sapiens	231.606	8	7	566345	114557
3930	1	Q92823	Q92823	Neuronal cell adhesion molecule	NRCAM	Homo sapiens	143.889	7	7	123540	640989
3931	1	Q92828	Q92828	Coronin-2A	CORO2A	Homo sapiens	59.759	7	7	1357102	837137
3932	1	Q92845	Q92845	Kinesin-associated protein 3	KIFAP3	Homo sapiens	91.204	7	7	406573	72079
3933	1	Q92947	Q92947	Glutaryl-CoA dehydrogenase, mitochondrial	GCDH	Homo sapiens	48.126	7	7	4280860	942131
3934	1	Q92954	Q92954	Proteoglycan 4	PRG4	Homo sapiens	151.064	7	7	361648	1448763
3935	1	Q969N2	Q969N2	GPI transamidase component PIG-T	PIGT	Homo sapiens	65.699	7	7	1781843	2553830
3936	1	Q969S3	Q969S3	Cytoplasmic 60S subunit biogenesis factor ZNF622	ZNF622	Homo sapiens	54.273	7	7	1423960	300532
3937	1	Q96A35	Q96A35	39S ribosomal protein L24, mitochondrial	MRPL24	Homo sapiens	24.914	7	7	2842156	569328
3938	1	Q96A73	Q96A73	Putative monooxygenase p33MONOX	KIAA1191	Homo sapiens	33.245	7	7	842977	0
3939	1	Q96C11	Q96C11	FGGY carbohydrate kinase domain-containing protein	FGGY	Homo sapiens	59.996	7	7	369239	0
3940	1	Q96C24	Q96C24	Synaptotagmin-like protein 4	SYTL4	Homo sapiens	76.022	7	7	728693	14389
3941	1	Q96C36	Q96C36	Pyrroline-5-carboxylate reductase 2	PYCR2	Homo sapiens	33.637	8	7	1624610	788265
3942	1	Q96F24	Q96F24	Nuclear receptor-binding factor 2	NRBF2	Homo sapiens	32.377	7	7	67817	2800288
3943	1	Q96FF7	Q96FF7	Uncharacterized protein MISP3	MISP3	Homo sapiens	24.027	7	7	881434	0
3944	1	Q96GA3	Q96GA3	Protein LTV1 homolog	LTV1	Homo sapiens	54.856	7	7	668715	17588
3945	1	Q96GD0	Q96GD0	Chronophin	PDXP	Homo sapiens	31.698	8	7	3428404	854025
3946	1	Q96H20	Q96H20	Vacuolar-sorting protein SNF8	SNF8	Homo sapiens	28.864	7	7	2293974	1073905
3947	1	Q96HY6	Q96HY6	DDRGK domain-containing protein 1	DDRGK1	Homo sapiens	35.61	7	7	2451278	875412
3948	1	Q96IY4	Q96IY4	Carboxypeptidase B2	CPB2	Homo sapiens	48.424	7	7	3422135	6390755
3949	1	Q96LZ7	Q96LZ7	Regulator of microtubule dynamics protein 2	RMDN2	Homo sapiens	47.399	7	7	844063	58498
3950	1	Q96NE9	Q96NE9	FERM domain-containing protein 6	FRMD6	Homo sapiens	72.045	7	7	625016	0
3951	1	Q96QU8	Q96QU8	Exportin-6	XPO6	Homo sapiens	128.886	7	7	100072	523777
3952	1	Q96R05	Q96R05	Retinoid-binding protein 7	RBP7	Homo sapiens	15.534	7	7	960665	2871505
3953	1	Q96RL1	Q96RL1	BRCA1-A complex subunit RAP80	UIMC1	Homo sapiens	79.728	7	7	559765	167285
3954	1	Q96RU2	Q96RU2	Ubiquitin carboxyl-terminal hydrolase 28	USP28	Homo sapiens	122.491	7	7	181062	145140
3955	1	Q96S86	Q96S86	Hyaluronan and proteoglycan link protein 3	HAPLN3	Homo sapiens	40.896	7	7	85231	1288066
3956	1	Q96T66	Q96T66	Nicotinamide/nicotinic acid mononucleotide adenylyltransferase 3	NMNAT3	Homo sapiens	28.321	7	7	978667	86016
3957	1	Q99426	Q99426	Tubulin-folding cofactor B	TBCB	Homo sapiens	27.325	7	7	8469249	2348282
3958	1	Q99436	Q99436	Proteasome subunit beta type-7	PSMB7	Homo sapiens	29.966	7	7	11986979	4371227
3959	1	Q99523	Q99523	Sortilin	SORT1	Homo sapiens	92.069	7	7	1340203	139862
3960	1	Q99704	Q99704	Docking protein 1	DOK1	Homo sapiens	52.391	7	7	361668	1166758
3961	1	Q99719	Q99719	Septin-5	SEPTIN5	Homo sapiens	42.777	7	7	1207245	2266823

3962	1	Q9BPX6	Q9BPX6	Calcium uptake protein 1, mitochondrial	MICU1	Homo sapiens	54.353	7	7	640166	207908
3963	1	Q9BRF8	Q9BRF8	Serine/threonine-protein phosphatase CPPED1	CPPED1	Homo sapiens	35.55	7	7	4175502	3699402
3964	1	Q9BRX2	Q9BRX2	Protein pelota homolog	PELO	Homo sapiens	43.358	7	7	971886	806949
3965	1	Q9BRX8	Q9BRX8	Peroxiredoxin-like 2A	PRXL2A	Homo sapiens	25.764	7	7	3306763	934713
3966	1	Q9BTC8	Q9BTC8	Metastasis-associated protein MTA3	MTA3	Homo sapiens	67.504	7	7	809492	7800
3967	1	Q9BTT6	Q9BTT6	Leucine-rich repeat-containing protein 1	LRRC1	Homo sapiens	59.244	7	7	1642075	78402
3968	1	Q9BTY2	Q9BTY2	Plasma alpha-L-fucosidase	FUCA2	Homo sapiens	54.066	7	7	1022029	589990
3969	1	Q9BUB7	Q9BUB7	Transmembrane protein 70, mitochondrial	TMEM70	Homo sapiens	28.972	7	7	1057344	422890
3970	1	Q9BVG4	Q9BVG4	Protein PBDC1	PBDC1	Homo sapiens	26.054	7	7	3417432	1154674
3971	1	Q9BVJ7	Q9BVJ7	Dual specificity protein phosphatase 23	DUSP23	Homo sapiens	16.589	7	7	1735097	1268856
3972	1	Q9BW04	Q9BW04	Specifically androgen-regulated gene protein	SARG	Homo sapiens	63.963	7	7	544597	72092
3973	1	Q9BW30	Q9BW30	Tubulin polymerization-promoting protein family member 3	TPPP3	Homo sapiens	18.984	7	7	12320995	856533
3974	1	Q9BX69	Q9BX69	Caspase recruitment domain-containing protein 6	CARD6	Homo sapiens	116.467	7	7	0	361137
3975	1	Q9BXI6	Q9BXI6	TBC1 domain family member 10A	TBC1D10A	Homo sapiens	57.118	7	7	728243	201154
3976	1	Q9BXR0	Q9BXR0	Queuine tRNA-ribosyltransferase catalytic subunit 1	QTRT1	Homo sapiens	44.049	7	7	1745418	554561
3977	1	Q9BY42	Q9BY42	Replication termination factor 2	RTF2	Homo sapiens	33.889	7	7	653887	350280
3978	1	Q9BY50	Q9BY50	Signal peptidase complex catalytic subunit SEC11C	SEC11C	Homo sapiens	21.542	7	7	2899368	575344
3979	1	Q9BY76	Q9BY76	Angiopoietin-related protein 4	ANGPTL4	Homo sapiens	45.216	7	7	75366	1984301
3980	1	Q9BYG3	Q9BYG3	MKI67 FHA domain-interacting nucleolar phosphoprotein	NIFK	Homo sapiens	34.223	7	7	1072064	830879
3981	1	Q9BYX4	Q9BYX4	Interferon-induced helicase C domain-containing protein 1	IFIH1	Homo sapiens	116.69	7	7	386524	305344
3982	1	Q9BZE9	Q9BZE9	Tether containing UBX domain for GLUT4	ASPSCR1	Homo sapiens	60.182	7	7	2104125	679458
3983	1	Q9BZF2	Q9BZF2	Oxysterol-binding protein-related protein 7	OSBPL7	Homo sapiens	95.431	7	7	506310	0
3984	1	Q9C0B0	Q9C0B0	RING finger protein unkempt homolog	UNK	Homo sapiens	88.087	7	7	936486	46242
3985	1	Q9C0H9	Q9C0H9	SRC kinase signaling inhibitor 1	SRCIN1	Homo sapiens	127.106	7	7	503956	0
3986	1	Q9GZY8	Q9GZY8	Mitochondrial fission factor	MFF	Homo sapiens	38.466	7	7	865496	547411
3987	1	Q9H089	Q9H089	Large subunit GTPase 1 homolog	LSG1	Homo sapiens	75.225	7	7	1063216	327996
3988	1	Q9H098	Q9H098	Protein FAM107B	FAM107B	Homo sapiens	15.556	7	7	1332813	1839464
3989	1	Q9H0R6	Q9H0R6	Glutamyl-tRNA(Gln) amidotransferase subunit A, mitochondrial	QRSL1	Homo sapiens	57.463	7	7	847018	215847
3990	1	Q9H0S4	Q9H0S4	Probable ATP-dependent RNA helicase DDX47	DDX47	Homo sapiens	50.646	7	7	1368133	191697
3991	1	Q9H0X9	Q9H0X9	Oxysterol-binding protein-related protein 5	OSBPL5	Homo sapiens	98.619	7	7	696242	36356
3992	1	Q9H1B7	Q9H1B7	Probable E3 ubiquitin-protein ligase IRF2BPL	IRF2BPL	Homo sapiens	82.659	7	7	642874	34854
3993	1	Q9H1I8	Q9H1I8	Activating signal cointegrator 1 complex subunit 2	ASCC2	Homo sapiens	86.363	7	7	833025	0
3994	1	Q9H1Y0	Q9H1Y0	Autophagy protein 5	ATG5	Homo sapiens	32.45	7	7	1795775	1235110
3995	1	Q9H300	Q9H300	Presenilins-associated rhomboid-like protein, mitochondrial	PARL	Homo sapiens	42.191	7	7	900768	329815
3996	1	Q9H3K2	Q9H3K2	Growth hormone-inducible transmembrane protein	GHITM	Homo sapiens	37.205	7	7	9726737	5616346
3997	1	Q9H4B7	Q9H4B7	Tubulin beta-1 chain	TUBB1	Homo sapiens	50.327	11	7	543849	2073853
3998	1	Q9H6A0	Q9H6A0	DENN domain-containing protein 2D	DENND2D	Homo sapiens	53.672	7	7	520142	640173
3999	1	Q9H6Q4	Q9H6Q4	Cytosolic iron-sulfur assembly component 3	CIAO3	Homo sapiens	53.021	9	7	1993474	181173
4000	1	Q9H773	Q9H773	dCTP pyrophosphatase 1	DCTPP1	Homo sapiens	18.682	7	7	7471985	846897
4001	1	Q9H857	Q9H857	5'-nucleotidase domain-containing protein 2	NT5DC2	Homo sapiens	60.718	7	7	530854	540376
4002	1	Q9H8H3	Q9H8H3	Putative methyltransferase-like protein 7A	METTL7A	Homo sapiens	28.317	7	7	6555055	740370
4003	1	Q9HA65	Q9HA65	TBC1 domain family member 17	TBC1D17	Homo sapiens	72.673	7	7	3133743	208611
4004	1	Q9HB21	Q9HB21	Pleckstrin homology domain-containing family A member 1	PLEKHA1	Homo sapiens	45.554	7	7	926698	159932
4005	1	Q9HBK9	Q9HBK9	Arsenite methyltransferase	AS3MT	Homo sapiens	41.749	7	7	672110	0
4006	1	Q9NNW5	Q9NNW5	WD repeat-containing protein 6	WDR6	Homo sapiens	121.724	7	7	615888	81137
4007	1	Q9NNX6	Q9NNX6	CD209 antigen	CD209	Homo sapiens	45.774	9	7	926711	182405
4008	1	Q9NP77	Q9NP77	RNA polymerase II subunit A C-terminal domain phosphatase SSU72	SSU72	Homo sapiens	22.572	7	7	755776	452332
4009	1	Q9NPI1	Q9NPI1	Bromodomain-containing protein 7	BRD7	Homo sapiens	74.138	7	7	673139	0

4010	1	Q9NPI6	Q9NPI6	mRNA-decapping enzyme 1A	DCP1A	Homo sapiens	63.277	7	7	848169	213527
4011	1	Q9NRF2	Q9NRF2	SH2B adapter protein 1	SH2B1	Homo sapiens	79.365	7	7	978182	0
4012	1	Q9NRH2	Q9NRH2	SNF-related serine/threonine-protein kinase	SNRK	Homo sapiens	84.276	7	7	218798	278202
4013	1	Q9NRN7	Q9NRN7	L-aminoadipate-semialdehyde dehydrogenase-phosphopantetheinyl transferase	AASDHPPT	Homo sapiens	35.778	7	7	1556221	770384
4014	1	Q9NS00	Q9NS00	Glycoprotein-N-acetylgalactosamine 3-beta-galactosyltransferase 1	C1GALT1	Homo sapiens	42.204	7	7	425691	633312
4015	1	Q9NS86	Q9NS86	LanC-like protein 2	LANCL2	Homo sapiens	50.855	7	7	896216	409614
4016	1	Q9NT62	Q9NT62	Ubiquitin-like-conjugating enzyme ATG3	ATG3	Homo sapiens	35.866	7	7	3028067	4486767
4017	1	Q9NUL7	Q9NUL7	Probable ATP-dependent RNA helicase DDX28	DDX28	Homo sapiens	59.581	7	7	597789	23118
4018	1	Q9NUQ2	Q9NUQ2	1-acyl-sn-glycerol-3-phosphate acyltransferase epsilon	AGPAT5	Homo sapiens	42.072	7	7	1496219	425970
4019	1	Q9NUY8	Q9NUY8	TBC1 domain family member 23	TBC1D23	Homo sapiens	78.324	7	7	1335760	1205839
4020	1	Q9NV88	Q9NV88	Integrator complex subunit 9	INTS9	Homo sapiens	73.815	7	7	552749	114437
4021	1	Q9NVC6	Q9NVC6	Mediator of RNA polymerase II transcription subunit 17	MED17	Homo sapiens	72.889	7	7	518331	311993
4022	1	Q9NVD7	Q9NVD7	Alpha-parvin	PARVA	Homo sapiens	42.243	8	7	29450978	1186010
4023	1	Q9NVI7	Q9NVI7	ATPase family AAA domain-containing protein 3A	ATAD3A	Homo sapiens	71.371	19	7	2250913	2222144
4024	1	Q9NW68	Q9NW68	BSD domain-containing protein 1	BSDC1	Homo sapiens	47.163	7	7	480666	215331
4025	1	Q9NWV8	Q9NWV8	BRISC and BRCA1-A complex member 1	BABAM1	Homo sapiens	36.559	7	7	2107422	1171774
4026	1	Q9NX02	Q9NX02	NACHT, LRR and PYD domains-containing protein 2	NLRP2	Homo sapiens	120.514	9	7	115937	649108
4027	1	Q9NXG2	Q9NXG2	THUMP domain-containing protein 1	THUMPD1	Homo sapiens	39.316	7	7	2014447	274410
4028	1	Q9NY61	Q9NY61	Protein AATF	AATF	Homo sapiens	63.134	7	7	496989	8712
4029	1	Q9NYJ8	Q9NYJ8	TGF-beta-activated kinase 1 and MAP3K7-binding protein 2	TAB2	Homo sapiens	76.497	7	7	499051	277849
4030	1	Q9NZ43	Q9NZ43	Vesicle transport protein USE1	USE1	Homo sapiens	29.371	7	7	1474515	691628
4031	1	Q9NZ56	Q9NZ56	Formin-2	FMN2	Homo sapiens	180.107	7	7	351742	0
4032	1	Q9NZD8	Q9NZD8	Maspardin	SPG21	Homo sapiens	34.959	7	7	767587	2336659
4033	1	Q9NZN8	Q9NZN8	CCR4-NOT transcription complex subunit 2	CNOT2	Homo sapiens	59.74	7	7	9924667	302272
4034	1	Q9NZP8	Q9NZP8	Complement C1r subcomponent-like protein	C1RL	Homo sapiens	53.501	7	7	1480340	2007783
4035	1	Q9NZR1	Q9NZR1	Tropomodulin-2	TMOD2	Homo sapiens	39.595	7	7	2116113	1448001
4036	1	Q9P016	Q9P016	Thymocyte nuclear protein 1	THYN1	Homo sapiens	25.698	7	7	3068314	1099516
4037	1	Q9P0U4	Q9P0U4	CXXC-type zinc finger protein 1	CXXC1	Homo sapiens	75.713	7	7	415385	135871
4038	1	Q9P289	Q9P289	Serine/threonine-protein kinase 26	STK26	Homo sapiens	46.528	9	7	1311039	3989458
4039	1	Q9P2K3	Q9P2K3	REST corepressor 3	RCOR3	Homo sapiens	55.581	7	7	607810	21969
4040	1	Q9P2X3	Q9P2X3	Protein IMPACT	IMPACT	Homo sapiens	36.476	7	7	970612	323637
4041	1	Q9UBI1	Q9UBI1	COMM domain-containing protein 3	COMMD3	Homo sapiens	22.15	7	7	1099436	564953
4042	1	Q9UBW8	Q9UBW8	COP9 signalosome complex subunit 7a	COPS7A	Homo sapiens	30.277	7	7	1570836	870732
4043	1	Q9UBY9	Q9UBY9	Heat shock protein beta-7	HSPB7	Homo sapiens	18.608	7	7	11453156	0
4044	1	Q9UFN0	Q9UFN0	Protein NipSnap homolog 3A	NIPSNAP3A	Homo sapiens	28.465	9	7	6907644	1496541
4045	1	Q9UH62	Q9UH62	Armadillo repeat-containing X-linked protein 3	ARMCX3	Homo sapiens	42.503	7	7	1313983	640812
4046	1	Q9UHB7	Q9UHB7	AF4/FMR2 family member 4	AFF4	Homo sapiens	127.461	7	7	497217	35932
4047	1	Q9UHI6	Q9UHI6	Probable ATP-dependent RNA helicase DDX20	DDX20	Homo sapiens	92.239	7	7	685419	159876
4048	1	Q9UHQ4	Q9UHQ4	B-cell receptor-associated protein 29	BCAP29	Homo sapiens	28.319	7	7	2509636	1021864
4049	1	Q9UHY1	Q9UHY1	Nuclear receptor-binding protein	NRBP1	Homo sapiens	59.844	7	7	1752087	940494
4050	1	Q9UHY7	Q9UHY7	Enolase-phosphatase E1	ENOPH1	Homo sapiens	28.932	7	7	2913330	1072118
4051	1	Q9UJ83	Q9UJ83	2-hydroxyacyl-CoA lyase 1	HACL1	Homo sapiens	63.729	7	7	399910	643819
4052	1	Q9UJA5	Q9UJA5	tRNA (adenine(58)-N(1))-methyltransferase non-catalytic subunit TRM6	TRMT6	Homo sapiens	55.799	7	7	793229	246869
4053	1	Q9UJF2	Q9UJF2	Ras GTPase-activating protein nGAP	RASAL2	Homo sapiens	128.559	7	7	236695	21894
4054	1	Q9UJY5	Q9UJY5	ADP-ribosylation factor-binding protein GGA1	GGA1	Homo sapiens	70.386	7	7	2926356	1079721
4055	1	Q9UK59	Q9UK59	Lariat debranching enzyme	DBR1	Homo sapiens	61.557	7	7	917280	1064543
4056	1	Q9UKJ3	Q9UKJ3	G patch domain-containing protein 8	GPATCH8	Homo sapiens	164.199	7	7	414913	29448

4057	1	Q9UL26	Q9UL26	Ras-related protein Rab-22A	RAB22A	Homo sapiens	21.856	7	7	1274020	499464
4058	1	Q9UL33	Q9UL33	Trafficking protein particle complex subunit 2-like protein	TRAPPC2L	Homo sapiens	16.145	7	7	2866757	1572813
4059	1	Q9UL63	Q9UL63	Muskelin	MKLN1	Homo sapiens	84.768	7	7	367894	137177
4060	1	Q9ULG6	Q9ULG6	Cell cycle progression protein 1	CCPG1	Homo sapiens	87.343	7	7	273721	652800
4061	1	Q9ULJ8	Q9ULJ8	Neurabin-1	PPP1R9A	Homo sapiens	123.343	7	7	528321	0
4062	1	Q9UNW1	Q9UNW1	Multiple inositol polyphosphate phosphatase 1	MINPP1	Homo sapiens	55.051	7	7	1233286	329244
4063	1	Q9UPQ3	Q9UPQ3	Arf-GAP with GTPase, ANK repeat and PH domain-containing protein 1	AGAP1	Homo sapiens	94.473	8	7	697218	86857
4064	1	Q9UQ16	Q9UQ16	Dynamin-3	DNM3	Homo sapiens	97.746	7	7	551570	383891
4065	1	Q9Y232	Q9Y232	Chromodomain Y-like protein	CDYL	Homo sapiens	66.483	8	7	674759	0
4066	1	Q9Y281	Q9Y281	Cofilin-2	CFL2	Homo sapiens	18.737	7	7	10176579	118975
4067	1	Q9Y2E5	Q9Y2E5	Epididymis-specific alpha-mannosidase	MAN2B2	Homo sapiens	113.98	7	7	502846	635487
4068	1	Q9Y2G2	Q9Y2G2	Caspase recruitment domain-containing protein 8	CARD8	Homo sapiens	60.652	7	7	276845	639382
4069	1	Q9Y2L9	Q9Y2L9	Leucine-rich repeat and calponin homology domain-containing protein 1	LRCH1	Homo sapiens	80.876	7	7	1268932	58622
4070	1	Q9Y2R2	Q9Y2R2	Tyrosine-protein phosphatase non-receptor type 22	PTPN22	Homo sapiens	91.705	7	7	0	1231264
4071	1	Q9Y314	Q9Y314	Nitric oxide synthase-interacting protein	NOSIP	Homo sapiens	33.171	7	7	663677	274706
4072	1	Q9Y316	Q9Y316	Protein MEMO1	MEMO1	Homo sapiens	33.733	7	7	1613628	1835065
4073	1	Q9Y394	Q9Y394	Dehydrogenase/reductase SDR family member 7	DHRS7	Homo sapiens	38.295	7	7	7790355	7709711
4074	1	Q9Y399	Q9Y399	28S ribosomal protein S2, mitochondrial	MRPS2	Homo sapiens	33.247	7	7	1517342	121291
4075	1	Q9Y3X0	Q9Y3X0	Coiled-coil domain-containing protein 9	CCDC9	Homo sapiens	59.703	7	7	1400560	83635
4076	1	Q9Y4C2	Q9Y4C2	TRPM8 channel-associated factor 1	TCAF1	Homo sapiens	102.128	7	7	656328	30179
4077	1	Q9Y4F3	Q9Y4F3	Meiosis regulator and mRNA stability factor 1	MARF1	Homo sapiens	192.863	7	7	138504	10842
4078	1	Q9Y4F9	Q9Y4F9	Rho family-interacting cell polarization regulator 2	RIPOR2	Homo sapiens	118.52	7	7	0	1308435
4079	1	Q9Y4K4	Q9Y4K4	Mitogen-activated protein kinase kinase kinase kinase 5	MAP4K5	Homo sapiens	95.026	7	7	612427	898782
4080	1	Q9Y4P8	Q9Y4P8	WD repeat domain phosphoinositide-interacting protein 2	WIPI2	Homo sapiens	49.408	7	7	1951179	1609223
4081	1	Q9Y572	Q9Y572	Receptor-interacting serine/threonine-protein kinase 3	RIPK3	Homo sapiens	56.888	7	7	976162	1039647
4082	1	Q9Y5P6	Q9Y5P6	Mannose-1-phosphate guanyltransferase beta	GMPPB	Homo sapiens	39.834	7	7	7672600	4087671
4083	1	Q9Y5W7	Q9Y5W7	Sorting nexin-14	SNX14	Homo sapiens	110.183	7	7	312973	101568
4084	1	Q9Y5W8	Q9Y5W8	Sorting nexin-13	SNX13	Homo sapiens	112.189	7	7	426963	505607
4085	1	Q9Y5Z4	Q9Y5Z4	Heme-binding protein 2	HEBP2	Homo sapiens	22.874	7	7	11997509	4958636
4086	1	Q9Y639	Q9Y639	Neuroplastin	NPTN	Homo sapiens	44.388	7	7	16680105	2713060
4087	1	Q9Y646	Q9Y646	Carboxypeptidase Q	CPQ	Homo sapiens	51.888	7	7	3135413	582509
4088	1	Q9Y673	Q9Y673	Dolichyl-phosphate beta-glucosyltransferase	ALG5	Homo sapiens	36.949	7	7	6121659	2101935
4089	1	Q9Y676	Q9Y676	28S ribosomal protein S18b, mitochondrial	MRPS18B	Homo sapiens	29.396	7	7	3599530	435837
4090	1	Q9Y6G5	Q9Y6G5	COMM domain-containing protein 10	COMMD10	Homo sapiens	22.966	7	7	2301649	536320
4091	1	Q9Y6I9	Q9Y6I9	Testis-expressed protein 264	TEX264	Homo sapiens	34.189	7	7	2694389	887003
4092	1	Q9Y6R0	Q9Y6R0	Numb-like protein	NUMBL	Homo sapiens	64.892	7	7	1311242	1545009
4093	1	Q9Y6X9	Q9Y6X9	ATPase MORC2	MORC2	Homo sapiens	117.825	7	7	585369	208825
4094	1	A1A5D9	A1A5D9	BICD family-like cargo adapter 2	BICDL2	Homo sapiens	56.835	6	6	583198	0
4095	1	A6NI28	A6NI28	Rho GTPase-activating protein 42	ARHGAP42	Homo sapiens	98.57	6	6	433698	0
4096	2	B7ZAQ6;P0CG08	B7ZAQ6	Golgi pH regulator A	GPR89A	Homo sapiens	52.917	6	6	698999	568381
4097	1	O00148	O00148	ATP-dependent RNA helicase DDX39A	DDX39A	Homo sapiens	49.129	6	6	651022	540752
4098	1	O00214	O00214	Galectin-8	LGALS8	Homo sapiens	35.804	6	6	995272	398883
4099	1	O00217	O00217	NADH dehydrogenase [ubiquinone] iron-sulfur protein 8, mitochondrial	NDUFS8	Homo sapiens	23.705	6	6	3745951	691714
4100	1	O00255	O00255	Menin	MEN1	Homo sapiens	68.024	6	6	465522	140017
4101	1	O00264	O00264	Membrane-associated progesterone receptor component 1	PGRMC1	Homo sapiens	21.67	7	6	30607587	4268822
4102	1	O00425	O00425	Insulin-like growth factor 2 mRNA-binding protein 3	IGF2BP3	Homo sapiens	63.705	6	6	481713	805455
4103	1	O00566	O00566	U3 small nucleolar ribonucleoprotein protein MPP10	MPHOSPH10	Homo sapiens	78.864	6	6	1200621	135911
4104	1	O00602	O00602	Ficolin-1	FCN1	Homo sapiens	35.078	6	6	12693	1647450

4105	1	O00622	O00622	CCN family member 1	CCN1	Homo sapiens	42.026	6	6	145092	592087
4106	1	O00625	O00625	Pirin	PIR	Homo sapiens	32.113	6	6	1460501	898368
4107	1	O00635	O00635	E3 ubiquitin-protein ligase TRIM38	TRIM38	Homo sapiens	53.415	6	6	1206778	607637
4108	1	O14523	O14523	Phospholipid transfer protein C2CD2L	C2CD2L	Homo sapiens	76.182	6	6	120208	281032
4109	1	O14578	O14578	Citron Rho-interacting kinase	CIT	Homo sapiens	231.435	6	6	346256	14509
4110	2	O14640;P54792	O14640	Segment polarity protein dishevelled homolog DVL-1	DVL1	Homo sapiens	75.188	6	6	280610	0
4111	1	O14641	O14641	Segment polarity protein dishevelled homolog DVL-2	DVL2	Homo sapiens	78.95	9	6	704409	0
4112	1	O14646	O14646	Chromodomain-helicase-DNA-binding protein 1	CHD1	Homo sapiens	196.692	6	6	546513	246591
4113	1	O14657	O14657	Torsin-1B	TOR1B	Homo sapiens	37.979	6	6	379891	629779
4114	1	O14678	O14678	Lysosomal cobalamin transporter ABCD4	ABCD4	Homo sapiens	68.596	6	6	253180	129680
4115	1	O14733	O14733	Dual specificity mitogen-activated protein kinase kinase 7	MAP2K7	Homo sapiens	47.484	6	6	844133	46241
4116	1	O14773	O14773	Tripeptidyl-peptidase 1	TPP1	Homo sapiens	61.247	6	6	12133907	4611907
4117	1	O14832	O14832	Phytanoyl-CoA dioxygenase, peroxisomal	PHYH	Homo sapiens	38.538	6	6	711787	0
4118	1	O14867	O14867	Transcription regulator protein BACH1	BACH1	Homo sapiens	81.958	6	6	202094	532631
4119	1	O14896	O14896	Interferon regulatory factor 6	IRF6	Homo sapiens	53.129	6	6	758597	68188
4120	1	O14929	O14929	Histone acetyltransferase type B catalytic subunit	HAT1	Homo sapiens	49.541	6	6	860903	855624
4121	1	O14939	O14939	Phospholipase D2	PLD2	Homo sapiens	105.987	6	6	472296	0
4122	1	O14958	O14958	Calsequestrin-2	CASQ2	Homo sapiens	46.436	6	6	1025349	0
4123	1	O14966	O14966	Ras-related protein Rab-7L1	RAB29	Homo sapiens	23.152	6	6	524313	746655
4124	1	O14979	O14979	Heterogeneous nuclear ribonucleoprotein D-like	HNRNPDL	Homo sapiens	46.439	7	6	6206071	1557036
4125	1	O15020	O15020	Spectrin beta chain, non-erythrocytic 2	SPTBN2	Homo sapiens	271.33	6	6	266483	79563
4126	1	O15037	O15037	Protein KHNYN	KHNYN	Homo sapiens	74.533	6	6	575576	52443
4127	1	O15047	O15047	Histone-lysine N-methyltransferase SETD1A	SETD1A	Homo sapiens	186.038	6	6	281983	96327
4128	1	O15068	O15068	Guanine nucleotide exchange factor DBS	MCF2L	Homo sapiens	128.111	6	6	244547	0
4129	1	O15123	O15123	Angiopoietin-2	ANGPT2	Homo sapiens	56.918	6	6	17268	917869
4130	1	O15126	O15126	Secretory carrier-associated membrane protein 1	SCAMP1	Homo sapiens	37.921	6	6	2547328	850538
4131	1	O15173	O15173	Membrane-associated progesterone receptor component 2	PGRMC2	Homo sapiens	23.818	6	6	13490072	2317349
4132	1	O15212	O15212	Prefoldin subunit 6	PFDN6	Homo sapiens	14.58	6	6	3168011	1433557
4133	1	O15228	O15228	Dihydroxyacetone phosphate acyltransferase	GNPAT	Homo sapiens	77.19	6	6	394344	517469
4134	1	O15498	O15498	Synaptobrevin homolog YKT6	YKT6	Homo sapiens	22.417	6	6	5383664	1490583
4135	1	O15530	O15530	3-phosphoinositide-dependent protein kinase 1	PDPK1	Homo sapiens	63.155	17	6	871938	1018234
4136	1	O43164	O43164	E3 ubiquitin-protein ligase Praja-2	PJA2	Homo sapiens	78.215	6	6	497148	0
4137	1	O43299	O43299	AP-5 complex subunit zeta-1	AP5Z1	Homo sapiens	88.606	6	6	366832	245873
4138	1	O43324	O43324	Eukaryotic translation elongation factor 1 epsilon-1	EEF1E1	Homo sapiens	19.807	6	6	3288953	2608614
4139	1	O43516	O43516	WAS/WASL-interacting protein family member 1	WIPF1	Homo sapiens	51.258	6	6	2524163	4145383
4140	1	O43572	O43572	A-kinase anchor protein 10, mitochondrial	AKAP10	Homo sapiens	73.819	6	6	343145	536217
4141	1	O43813	O43813	Glutathione S-transferase LANCL1	LANCL1	Homo sapiens	45.284	6	6	11347470	2914287
4142	1	O43865	O43865	S-adenosylhomocysteine hydrolase-like protein 1	AHCYL1	Homo sapiens	58.953	6	6	1230379	686151
4143	1	O43913	O43913	Origin recognition complex subunit 5	ORC5	Homo sapiens	50.284	6	6	335457	17716
4144	1	O60513	O60513	Beta-1,4-galactosyltransferase 4	B4GALT4	Homo sapiens	40.041	6	6	1083323	189620
4145	1	O60573	O60573	Eukaryotic translation initiation factor 4E type 2	EIF4E2	Homo sapiens	28.362	6	6	1807729	727528
4146	1	O60844	O60844	Zymogen granule membrane protein 16	ZG16	Homo sapiens	18.134	6	6	47349132	3351031
4147	1	O75044	O75044	SLIT-ROBO Rho GTPase-activating protein 2	SRGAP2	Homo sapiens	120.871	17	6	357660	437389
4148	1	O75054	O75054	Immunoglobulin superfamily member 3	IGSF3	Homo sapiens	135.197	6	6	194159	0
4149	1	O75147	O75147	Obscurin-like protein 1	OBSL1	Homo sapiens	206.946	6	6	352338	0
4150	1	O75167	O75167	Phosphatase and actin regulator 2	PHACTR2	Homo sapiens	69.7	6	6	361149	199963
4151	1	O75323	O75323	Protein NipSnap homolog 2	NIPSNAP2	Homo sapiens	33.745	6	6	8160201	1965500
4152	1	O75355	O75355	Ectonucleoside triphosphate diphosphohydrolase 3	ENTPD3	Homo sapiens	59.107	6	6	753348	0

4153	1	O75636	O75636	Ficolin-3	FCN3	Homo sapiens	32.902	6	6	879563	3143245
4154	1	O75648	O75648	Mitochondrial tRNA-specific 2-thiouridylase 1	TRMU	Homo sapiens	47.745	6	6	479613	101340
4155	1	O75665	O75665	Centriole and centriolar satellite protein OFD1	OFD1	Homo sapiens	116.671	6	6	165836	2089580
4156	1	O75674	O75674	TOM1-like protein 1	TOM1L1	Homo sapiens	52.989	6	6	1056863	26961
4157	1	O75689	O75689	Arf-GAP with dual PH domain-containing protein 1	ADAP1	Homo sapiens	43.395	7	6	808070	769082
4158	1	O75792	O75792	Ribonuclease H2 subunit A	RNASEH2A	Homo sapiens	33.395	6	6	891376	784649
4159	1	O75911	O75911	Short-chain dehydrogenase/reductase 3	DHRS3	Homo sapiens	33.546	6	6	581957	510787
4160	1	O75934	O75934	Pre-mRNA-splicing factor SPF27	BCAS2	Homo sapiens	26.133	6	6	876799	405341
4161	1	O75937	O75937	DnaJ homolog subfamily C member 8	DNAJC8	Homo sapiens	29.839	6	6	1118412	594797
4162	1	O76071	O76071	Probable cytosolic iron-sulfur protein assembly protein CIAO1	CIAO1	Homo sapiens	37.839	6	6	1264452	1410069
4163	1	O94829	O94829	Importin-13	IPO13	Homo sapiens	108.198	6	6	800605	245663
4164	1	O94923	O94923	D-glucuronyl C5-epimerase	GLCE	Homo sapiens	70.103	6	6	729067	99671
4165	1	O95169	O95169	NADH dehydrogenase [ubiquinone] 1 beta subcomplex subunit 8, mitochondrial	NDUFB8	Homo sapiens	21.767	6	6	10052371	2703046
4166	1	O95376	O95376	E3 ubiquitin-protein ligase ARIH2	ARIH2	Homo sapiens	57.819	6	6	777842	457748
4167	1	O95400	O95400	CD2 antigen cytoplasmic tail-binding protein 2	CD2BP2	Homo sapiens	37.647	6	6	839361	2270367
4168	1	O95445	O95445	Apolipoprotein M	APOM	Homo sapiens	21.253	6	6	1045361	3828157
4169	1	O95477	O95477	Phospholipid-transporting ATPase ABCA1	ABCA1	Homo sapiens	254.303	6	6	186604	139219
4170	1	O95490	O95490	Adhesion G protein-coupled receptor L2	ADGRL2	Homo sapiens	163.351	6	6	300311	12594
4171	1	O95716	O95716	Ras-related protein Rab-3D	RAB3D	Homo sapiens	24.266	6	6	2167252	3586116
4172	1	O95758	O95758	Polypyrimidine tract-binding protein 3	PTBP3	Homo sapiens	59.691	6	6	2540854	2552011
4173	1	O95985	O95985	DNA topoisomerase 3-beta-1	TOP3B	Homo sapiens	96.662	6	6	627575	18072
4174	1	O96028	O96028	Histone-lysine N-methyltransferase NSD2	NSD2	Homo sapiens	152.262	6	6	909947	0
4175	1	P00441	P00441	Superoxide dismutase [Cu-Zn]	SOD1	Homo sapiens	15.935	6	6	85682716	23438712
4176	1	P01591	P01591	Immunoglobulin J chain	JCHAIN	Homo sapiens	18.097	6	6	21221435	21336149
4177	1	P01876	P01876	Immunoglobulin heavy constant alpha 1	IGHA1	Homo sapiens	37.653	6	6	104858220	153213584
4178	1	P01877	P01877	Immunoglobulin heavy constant alpha 2	IGHA2	Homo sapiens	36.59	9	6	28540364	12163752
4179	1	P02042	P02042	Hemoglobin subunit delta	HBD	Homo sapiens	16.056	6	6	170683599	156479215
4180	1	P02511	P02511	Alpha-crystallin B chain	CRYAB	Homo sapiens	20.155	6	6	39800740	614294
4181	1	P02745	P02745	Complement C1q subcomponent subunit A	C1QA	Homo sapiens	26.016	6	6	3817931	8015290
4182	1	P02746	P02746	Complement C1q subcomponent subunit B	C1QB	Homo sapiens	26.72	6	6	10650349	18957877
4183	1	P03950	P03950	Angiogenin	ANG	Homo sapiens	16.549	6	6	1458485	1894758
4184	1	P03973	P03973	Antileukoproteinase	SLPI	Homo sapiens	14.326	6	6	361216	10301168
4185	1	P04180	P04180	Phosphatidylcholine-sterol acyltransferase	LCAT	Homo sapiens	49.58	6	6	261247	1293058
4186	1	P04920	P04920	Anion exchange protein 2	SLC4A2	Homo sapiens	137.012	7	6	1690889	805697
4187	1	P05062	P05062	Fructose-bisphosphate aldolase B	ALDOB	Homo sapiens	39.473	6	6	0	540545
4188	1	P05090	P05090	Apolipoprotein D	APOD	Homo sapiens	21.276	6	6	7972022	19349242
4189	1	P05141	P05141	ADP/ATP translocase 2	SLC25A5	Homo sapiens	32.855	7	6	69337474	14841976
4190	1	P05161	P05161	Ubiquitin-like protein ISG15	ISG15	Homo sapiens	17.886	6	6	1580628	1917723
4191	1	P05423	P05423	DNA-directed RNA polymerase III subunit RPC4	POLR3D	Homo sapiens	44.395	6	6	386621	0
4192	1	P05543	P05543	Thyroxine-binding globulin	SERPINA7	Homo sapiens	46.325	9	6	2029449	3081197
4193	1	P06493	P06493	Cyclin-dependent kinase 1	CDK1	Homo sapiens	34.096	6	6	883579	473301
4194	1	P06753	P06753	Tropomyosin alpha-3 chain	TPM3	Homo sapiens	32.95	6	6	11340251	19539392
4195	1	P08174	P08174	Complement decay-accelerating factor	CD55	Homo sapiens	41.402	6	6	6177392	4149242
4196	1	P08651	P08651	Nuclear factor 1 C-type	NFIC	Homo sapiens	55.675	6	6	2292181	96629
4197	1	P09038	P09038	Fibroblast growth factor 2	FGF2	Homo sapiens	30.77	6	6	1296462	134884
4198	1	P09132	P09132	Signal recognition particle 19 kDa protein	SRP19	Homo sapiens	16.157	6	6	1699275	1144017
4199	1	P09172	P09172	Dopamine beta-hydroxylase	DBH	Homo sapiens	69.067	6	6	388009	191430

4200	1	P09326	P09326	CD48 antigen	CD48	Homo sapiens	27.684	6	6	906207	944506
4201	1	P09382	P09382	Galectin-1	LGALS1	Homo sapiens	14.713	6	6	217848080	37688629
4202	1	P09497	P09497	Clathrin light chain B	CLTB	Homo sapiens	25.19	6	6	8583069	1672565
4203	2	P0DMM9;P0DMN	P0DMM9	Sulfotransferase 1A3	SULT1A3	Homo sapiens	34.196	6	6	2595634	807997
4204	1	P10451	P10451	Osteopontin	SPP1	Homo sapiens	35.419	7	6	0	3178238
4205	1	P10619	P10619	Lysosomal protective protein	CTSA	Homo sapiens	54.465	6	6	2482961	2026623
4206	1	P11766	P11766	Alcohol dehydrogenase class-3	ADH5	Homo sapiens	39.726	6	6	18022457	2778910
4207	1	P11802	P11802	Cyclin-dependent kinase 4	CDK4	Homo sapiens	33.731	6	6	1127225	46266
4208	1	P12544	P12544	Granzyme A	GZMA	Homo sapiens	28.998	6	6	443409	866237
4209	1	P12931	P12931	Proto-oncogene tyrosine-protein kinase Src	SRC	Homo sapiens	59.836	10	6	3062705	1212839
4210	1	P13612	P13612	Integrin alpha-4	ITGA4	Homo sapiens	114.902	6	6	882369	141337
4211	1	P13693	P13693	Translationally-controlled tumor protein	TPT1	Homo sapiens	19.597	8	6	5152953	2786747
4212	1	P15088	P15088	Mast cell carboxypeptidase A	CPA3	Homo sapiens	48.669	6	6	29016711	28548
4213	1	P15289	P15289	Arylsulfatase A	ARSA	Homo sapiens	53.586	6	6	6390024	1112669
4214	1	P16383	P16383	Intron Large complex component GCFC2	GCFC2	Homo sapiens	89.387	6	6	242871	0
4215	1	P16422	P16422	Epithelial cell adhesion molecule	EPCAM	Homo sapiens	34.932	6	6	18337554	1444584
4216	1	P16989	P16989	Y-box-binding protein 3	YBX3	Homo sapiens	40.089	6	6	2817064	1447679
4217	1	P17676	P17676	CCAAT/enhancer-binding protein beta	CEBPB	Homo sapiens	36.104	6	6	255476	2591315
4218	1	P17813	P17813	Endoglin	ENG	Homo sapiens	70.58	6	6	393213	498578
4219	1	P17931	P17931	Galectin-3	LGALS3	Homo sapiens	26.153	6	6	64330146	8578303
4220	1	P18065	P18065	Insulin-like growth factor-binding protein 2	IGFBP2	Homo sapiens	34.814	10	6	750837	167984
4221	1	P18074	P18074	General transcription and DNA repair factor IIH helicase subunit XPD	ERCC2	Homo sapiens	86.91	6	6	192184	179750
4222	1	P18564	P18564	Integrin beta-6	ITGB6	Homo sapiens	85.935	6	6	537684	598429
4223	1	P19447	P19447	General transcription and DNA repair factor IIH helicase subunit XPB	ERCC3	Homo sapiens	89.279	6	6	273936	229013
4224	1	P20138	P20138	Myeloid cell surface antigen CD33	CD33	Homo sapiens	39.823	6	6	74140	647696
4225	1	P20336	P20336	Ras-related protein Rab-3A	RAB3A	Homo sapiens	24.984	6	6	2068429	1501894
4226	1	P20674	P20674	Cytochrome c oxidase subunit 5A, mitochondrial	COX5A	Homo sapiens	16.756	6	6	20101840	2105371
4227	1	P20908	P20908	Collagen alpha-1(V) chain	COL5A1	Homo sapiens	183.562	6	6	1520751	1992942
4228	1	P21579	P21579	Synaptotagmin-1	SYT1	Homo sapiens	47.573	9	6	973446	0
4229	1	P21953	P21953	2-oxoisovalerate dehydrogenase subunit beta, mitochondrial	BCKDHB	Homo sapiens	43.123	6	6	1466610	231611
4230	1	P22352	P22352	Glutathione peroxidase 3	GPX3	Homo sapiens	25.525	6	6	19591602	12447132
4231	1	P22692	P22692	Insulin-like growth factor-binding protein 4	IGFBP4	Homo sapiens	27.934	6	6	472905	1218900
4232	1	P23193	P23193	Transcription elongation factor A protein 1	TCEA1	Homo sapiens	33.97	8	6	4089656	984470
4233	1	P23528	P23528	Cofilin-1	CFL1	Homo sapiens	18.501	9	6	40164269	35002821
4234	1	P24158	P24158	Myeloblastin	PRTN3	Homo sapiens	27.807	6	6	3230670	34368240
4235	1	P25445	P25445	Tumor necrosis factor receptor superfamily member 6	FAS	Homo sapiens	37.733	6	6	854800	226706
4236	1	P26583	P26583	High mobility group protein B2	HMGB2	Homo sapiens	24.032	6	6	23759520	49136353
4237	1	P27635	P27635	60S ribosomal protein L10	RPL10	Homo sapiens	24.575	10	6	8077299	2693858
4238	1	P27918	P27918	Properdin	CFP	Homo sapiens	51.278	6	6	1165858	6043101
4239	1	P28065	P28065	Proteasome subunit beta type-9	PSMB9	Homo sapiens	23.263	6	6	5060651	4753887
4240	1	P28068	P28068	HLA class II histocompatibility antigen, DM beta chain	HLA-DMB	Homo sapiens	28.93	6	6	622601	325977
4241	1	P28070	P28070	Proteasome subunit beta type-4	PSMB4	Homo sapiens	29.206	6	6	7169517	3503638
4242	1	P28161	P28161	Glutathione S-transferase Mu 2	GSTM2	Homo sapiens	25.743	13	6	9687447	464282
4243	1	P28300	P28300	Protein-lysine 6-oxidase	LOX	Homo sapiens	46.946	6	6	0	1091877
4244	1	P29083	P29083	General transcription factor IIE subunit 1	GTF2E1	Homo sapiens	49.452	6	6	361690	537043
4245	1	P29375	P29375	Lysine-specific demethylase 5A	KDM5A	Homo sapiens	192.1	7	6	235502	9595
4246	1	P29762	P29762	Cellular retinoic acid-binding protein 1	CRABP1	Homo sapiens	15.566	6	6	1072817	0
4247	1	P29992	P29992	Guanine nucleotide-binding protein subunit alpha-11	GNA11	Homo sapiens	42.125	13	6	7753009	704118

4248	1	P30048	P30048	Thioredoxin-dependent peroxide reductase, mitochondrial	PRDX3	Homo sapiens	27.693	6	6	41904612	15463372
4249	1	P30838	P30838	Aldehyde dehydrogenase, dimeric NADP-preferring	ALDH3A1	Homo sapiens	50.394	6	6	378947	0
4250	1	P31431	P31431	Syndecan-4	SDC4	Homo sapiens	21.642	6	6	1052867	97477
4251	1	P35244	P35244	Replication protein A 14 kDa subunit	RPA3	Homo sapiens	13.569	6	6	1826225	2213894
4252	1	P35475	P35475	Alpha-L-iduronidase	IDUA	Homo sapiens	72.67	6	6	983023	130525
4253	1	P35610	P35610	Sterol O-acyltransferase 1	SOAT1	Homo sapiens	64.736	6	6	498222	609631
4254	1	P35625	P35625	Metalloproteinase inhibitor 3	TIMP3	Homo sapiens	24.145	6	6	2837578	1077963
4255	1	P36543	P36543	V-type proton ATPase subunit E 1	ATP6V1E1	Homo sapiens	26.145	8	6	4620186	5569859
4256	1	P37108	P37108	Signal recognition particle 14 kDa protein	SRP14	Homo sapiens	14.572	6	6	11628064	4105995
4257	1	P37275	P37275	Zinc finger E-box-binding homeobox 1	ZEB1	Homo sapiens	124.074	6	6	400359	0
4258	1	P39019	P39019	40S ribosomal protein S19	RPS19	Homo sapiens	16.062	6	6	21253643	5125319
4259	1	P40261	P40261	Nicotinamide N-methyltransferase	NNMT	Homo sapiens	29.574	6	6	1328779	8725907
4260	1	P40616	P40616	ADP-ribosylation factor-like protein 1	ARL1	Homo sapiens	20.418	6	6	4449178	1493485
4261	1	P41227	P41227	N-alpha-acetyltransferase 10	NAA10	Homo sapiens	26.459	8	6	5245401	1254545
4262	1	P42685	P42685	Tyrosine-protein kinase FRK	FRK	Homo sapiens	58.252	6	6	143950	250289
4263	1	P42694	P42694	Probable helicase with zinc finger domain	HELZ	Homo sapiens	218.974	6	6	230164	69712
4264	1	P43251	P43251	Biotinidase	BTD	Homo sapiens	61.131	6	6	1987972	1131581
4265	1	P45973	P45973	Chromobox protein homolog 5	CBX5	Homo sapiens	22.223	7	6	3782899	1857621
4266	1	P45985	P45985	Dual specificity mitogen-activated protein kinase kinase 4	MAP2K4	Homo sapiens	44.29	6	6	2079846	1392944
4267	1	P46439	P46439	Glutathione S-transferase Mu 5	GSTM5	Homo sapiens	25.676	7	6	642569	0
4268	1	P48651	P48651	Phosphatidylserine synthase 1	PTDSS1	Homo sapiens	55.529	6	6	481547	469090
4269	1	P48728	P48728	Aminomethyltransferase, mitochondrial	AMT	Homo sapiens	43.948	6	6	1665215	0
4270	1	P49418	P49418	Amphiphysin	AMPH	Homo sapiens	76.258	6	6	363025	0
4271	1	P49585	P49585	Choline-phosphate cytidylyltransferase A	PCYT1A	Homo sapiens	41.732	11	6	3109422	2016317
4272	1	P49641	P49641	Alpha-mannosidase 2x	MAN2A2	Homo sapiens	130.542	6	6	41724	236125
4273	1	P49642	P49642	DNA primase small subunit	PRIM1	Homo sapiens	49.902	6	6	376380	391575
4274	1	P49711	P49711	Transcriptional repressor CTCF	CTCF	Homo sapiens	82.788	7	6	1053884	438888
4275	1	P49773	P49773	Adenosine 5'-monophosphoramidase HINT1	HINT1	Homo sapiens	13.801	6	6	4510136	1124119
4276	1	P49913	P49913	Cathelicidin antimicrobial peptide	CAMP	Homo sapiens	19.3	6	6	721018	45707836
4277	1	P49917	P49917	DNA ligase 4	LIG4	Homo sapiens	103.973	6	6	199575	215135
4278	1	P50440	P50440	Glycine amidinotransferase, mitochondrial	GATM	Homo sapiens	48.454	6	6	1002826	0
4279	1	P50914	P50914	60S ribosomal protein L14	RPL14	Homo sapiens	23.429	6	6	29942072	17083639
4280	1	P51452	P51452	Dual specificity protein phosphatase 3	DUSP3	Homo sapiens	20.476	6	6	16494606	1950827
4281	1	P51617	P51617	Interleukin-1 receptor-associated kinase 1	IRAK1	Homo sapiens	76.538	6	6	355561	126651
4282	1	P51689	P51689	Arylsulfatase D	ARSD	Homo sapiens	64.86	6	6	597356	224319
4283	1	P51788	P51788	Chloride channel protein 2	CLCN2	Homo sapiens	98.536	6	6	900057	0
4284	1	P51798	P51798	H(+)/Cl(-) exchange transporter 7	CLCN7	Homo sapiens	88.68	6	6	726132	1185456
4285	1	P52292	P52292	Importin subunit alpha-1	KPNA2	Homo sapiens	57.864	6	6	946008	498995
4286	1	P52565	P52565	Rho GDP-dissociation inhibitor 1	ARHGDIA	Homo sapiens	23.208	6	6	36132405	20868421
4287	1	P52788	P52788	Spermine synthase	SMS	Homo sapiens	41.267	6	6	5450837	3155073
4288	1	P52824	P52824	Diacylglycerol kinase theta	DGKQ	Homo sapiens	101.157	6	6	328522	30636
4289	1	P53677	P53677	AP-3 complex subunit mu-2	AP3M2	Homo sapiens	46.976	6	6	490936	26897
4290	1	P53985	P53985	Monocarboxylate transporter 1	SLC16A1	Homo sapiens	53.943	6	6	4458588	663839
4291	1	P53999	P53999	Activated RNA polymerase II transcriptional coactivator p15	SUB1	Homo sapiens	14.393	6	6	19014407	6359896
4292	1	P54098	P54098	DNA polymerase subunit gamma-1	POLG	Homo sapiens	139.564	6	6	483529	66066
4293	1	P54709	P54709	Sodium/potassium-transporting ATPase subunit beta-3	ATP1B3	Homo sapiens	31.514	6	6	7658352	3004987
4294	1	P54922	P54922	ADP-ribosylhydrolase ARH1	ADPRH	Homo sapiens	39.507	6	6	1089365	54233
4295	1	P55036	P55036	26S proteasome non-ATPase regulatory subunit 4	PSMD4	Homo sapiens	40.738	7	6	6475928	4487371

4296	1	P55081	P55081	Microfibrillar-associated protein 1	MFAP1	Homo sapiens	51.958	6	6	1136026	391953
4297	1	P55209	P55209	Nucleosome assembly protein 1-like 1	NAP1L1	Homo sapiens	45.374	8	6	5861569	3599131
4298	1	P56470	P56470	Galectin-4	LGALS4	Homo sapiens	35.94	6	6	285166125	3632332
4299	1	P56945	P56945	Breast cancer anti-estrogen resistance protein 1	BCAR1	Homo sapiens	93.373	6	6	764284	128140
4300	1	P58215	P58215	Lysyl oxidase homolog 3	LOXL3	Homo sapiens	83.167	6	6	211853	0
4301	1	P60510	P60510	Serine/threonine-protein phosphatase 4 catalytic subunit	PPP4C	Homo sapiens	35.079	6	6	1315134	1526403
4302	1	P61020	P61020	Ras-related protein Rab-5B	RAB5B	Homo sapiens	23.706	9	6	10036775	5481477
4303	1	P61163	P61163	Alpha-centractin	ACTR1A	Homo sapiens	42.613	11	6	5494650	2593041
4304	1	P61601	P61601	Neurocalcin-delta	NCALD	Homo sapiens	22.245	6	6	1743260	0
4305	1	P61619	P61619	Protein transport protein Sec61 subunit alpha isoform 1	SEC61A1	Homo sapiens	52.264	7	6	7787396	5327286
4306	1	P61964	P61964	WD repeat-containing protein 5	WDR5	Homo sapiens	36.589	6	6	1762069	1530198
4307	1	P62070	P62070	Ras-related protein R-Ras2	RRAS2	Homo sapiens	23.398	6	6	1925030	476797
4308	1	P62244	P62244	40S ribosomal protein S15a	RPS15A	Homo sapiens	14.839	6	6	13918404	7289518
4309	1	P62263	P62263	40S ribosomal protein S14	RPS14	Homo sapiens	16.271	6	6	44315377	17554283
4310	1	P62745	P62745	Rho-related GTP-binding protein RhoB	RHOB	Homo sapiens	22.121	6	6	2527484	419774
4311	1	P62857	P62857	40S ribosomal protein S28	RPS28	Homo sapiens	7.839	6	6	18584968	4230979
4312	1	P63151	P63151	Serine/threonine-protein phosphatase 2A 55 kDa regulatory subunit B alpha isoform	PPP2R2A	Homo sapiens	51.692	12	6	3313785	1891565
4313	1	P67812	P67812	Signal peptidase complex catalytic subunit SEC11A	SEC11A	Homo sapiens	20.624	7	6	1570213	1418914
4314	1	P67870	P67870	Casein kinase II subunit beta	CSNK2B	Homo sapiens	24.943	6	6	3111822	1511312
4315	1	P78324	P78324	Tyrosine-protein phosphatase non-receptor type substrate 1	SIRPA	Homo sapiens	54.965	6	6	411807	3662836
4316	1	P78325	P78325	Disintegrin and metalloproteinase domain-containing protein 8	ADAM8	Homo sapiens	88.772	6	6	9292	919102
4317	1	P78356	P78356	Phosphatidylinositol 5-phosphate 4-kinase type-2 beta	PIP4K2B	Homo sapiens	47.377	6	6	1511582	306812
4318	1	P80365	P80365	11-beta-hydroxysteroid dehydrogenase type 2	HSD11B2	Homo sapiens	44.128	6	6	3528481	51797
4319	1	P82930	P82930	28S ribosomal protein S34, mitochondrial	MRPS34	Homo sapiens	25.651	6	6	1084168	283884
4320	1	P85298	P85298	Rho GTPase-activating protein 8	ARHGAP8	Homo sapiens	53.486	6	6	767893	133023
4321	1	Q00537	Q00537	Cyclin-dependent kinase 17	CDK17	Homo sapiens	59.585	8	6	575379	84212
4322	1	Q00587	Q00587	Cdc42 effector protein 1	CDC42EP1	Homo sapiens	40.295	6	6	964062	269030
4323	1	Q01105	Q01105	Protein SET	SET	Homo sapiens	33.487	10	6	1356079	1219749
4324	1	Q01469	Q01469	Fatty acid-binding protein 5	FABP5	Homo sapiens	15.163	7	6	15367713	4279567
4325	1	Q01831	Q01831	DNA repair protein complementing XP-C cells	XPC	Homo sapiens	105.953	6	6	732266	85793
4326	1	Q03518	Q03518	Antigen peptide transporter 1	TAP1	Homo sapiens	80.967	6	6	2797375	3628760
4327	1	Q04912	Q04912	Macrophage-stimulating protein receptor	MST1R	Homo sapiens	152.242	6	6	255952	195767
4328	1	Q05469	Q05469	Hormone-sensitive lipase	LIPE	Homo sapiens	116.6	6	6	193911	100631
4329	1	Q05519	Q05519	Serine/arginine-rich splicing factor 11	SRSF11	Homo sapiens	53.54	6	6	2192789	236976
4330	1	Q05639	Q05639	Elongation factor 1-alpha 2	EEF1A2	Homo sapiens	50.47	14	6	663234	858783
4331	1	Q06265	Q06265	Exosome complex component RRP45	EXOSC9	Homo sapiens	48.949	6	6	1081012	376487
4332	1	Q07666	Q07666	KH domain-containing, RNA-binding, signal transduction-associated protein 1	KHDRBS1	Homo sapiens	48.225	8	6	8217483	3345174
4333	1	Q08170	Q08170	Serine/arginine-rich splicing factor 4	SRSF4	Homo sapiens	56.678	9	6	1795896	441149
4334	1	Q08AD1	Q08AD1	Calmodulin-regulated spectrin-associated protein 2	CAMSAP2	Homo sapiens	168.091	6	6	324812	0
4335	1	Q09028	Q09028	Histone-binding protein RBBP4	RBBP4	Homo sapiens	47.655	6	6	4329188	1998842
4336	1	Q09328	Q09328	Alpha-1,6-mannosylglycoprotein 6-beta-N-acetylglucosaminyltransferase A	MGAT5	Homo sapiens	84.543	6	6	262334	104251
4337	1	Q0VF96	Q0VF96	Cingulin-like protein 1	CGNL1	Homo sapiens	149.083	6	6	300306	0
4338	1	Q10469	Q10469	Alpha-1,6-mannosyl-glycoprotein 2-beta-N-acetylglucosaminyltransferase	MGAT2	Homo sapiens	51.549	6	6	637523	7544262

4339	1 Q12824	Q12824	SWI/SNF-related matrix-associated actin-dependent regulator of chromatin subfamily B member 1	SMARCB1	Homo sapiens	44.144	6	6	1365190	462017
4340	1 Q12841	Q12841	Follistatin-related protein 1	FSTL1	Homo sapiens	34.989	6	6	1202452	1025613
4341	1 Q12857	Q12857	Nuclear factor 1 A-type	NFIA	Homo sapiens	55.944	8	6	3026742	0
4342	1 Q12872	Q12872	Splicing factor, suppressor of white-apricot homolog	SFSWAP	Homo sapiens	104.822	6	6	499079	154052
4343	1 Q12933	Q12933	TNF receptor-associated factor 2	TRAF2	Homo sapiens	55.859	6	6	842103	133391
4344	1 Q13049	Q13049	E3 ubiquitin-protein ligase TRIM32	TRIM32	Homo sapiens	71.991	6	6	917213	27057
4345	1 Q13099	Q13099	Intraflagellar transport protein 88 homolog	IFT88	Homo sapiens	93.191	6	6	320503	0
4346	1 Q13148	Q13148	TAR DNA-binding protein 43	TARDBP	Homo sapiens	44.74	6	6	3999067	3035908
4347	1 Q13164	Q13164	Mitogen-activated protein kinase 7	MAPK7	Homo sapiens	88.387	6	6	522028	405597
4348	1 Q13185	Q13185	Chromobox protein homolog 3	CBX3	Homo sapiens	20.81	7	6	5225810	7275721
4349	1 Q13232	Q13232	Nucleoside diphosphate kinase 3	NME3	Homo sapiens	19.016	6	6	4858007	1235746
4350	1 Q13442	Q13442	28 kDa heat- and acid-stable phosphoprotein	PDAP1	Homo sapiens	20.63	6	6	6643596	1752301
4351	1 Q13526	Q13526	Peptidyl-prolyl cis-trans isomerase NIMA-interacting 1	PIN1	Homo sapiens	18.246	6	6	2110371	510715
4352	1 Q13547	Q13547	Histone deacetylase 1	HDAC1	Homo sapiens	55.104	13	6	1703878	605520
4353	1 Q13563	Q13563	Polycystin-2	PKD2	Homo sapiens	109.693	6	6	935307	24135
4354	1 Q13610	Q13610	Periodic tryptophan protein 1 homolog	PWP1	Homo sapiens	55.83	6	6	1219056	118401
4355	1 Q13643	Q13643	Four and a half LIM domains protein 3	FHL3	Homo sapiens	31.191	6	6	642758	856990
4356	1 Q13868	Q13868	Exosome complex component RRP4	EXOSC2	Homo sapiens	32.787	6	6	2261156	2402255
4357	1 Q13951	Q13951	Core-binding factor subunit beta	CBFB	Homo sapiens	21.51	6	6	256140	869878
4358	1 Q14004	Q14004	Cyclin-dependent kinase 13	CDK13	Homo sapiens	164.927	6	6	297879	96598
4359	1 Q14146	Q14146	Unhealthy ribosome biogenesis protein 2 homolog	URB2	Homo sapiens	170.547	6	6	277951	36511
4360	1 Q14232	Q14232	Translation initiation factor eIF-2B subunit alpha	EIF2B1	Homo sapiens	33.713	6	6	3091175	1055836
4361	1 Q14376	Q14376	UDP-glucose 4-epimerase	GALE	Homo sapiens	38.28	6	6	783793	96548
4362	1 Q14457	Q14457	Beclin-1	BECN1	Homo sapiens	51.897	6	6	909473	3121446
4363	1 Q14687	Q14687	Genetic suppressor element 1	GSE1	Homo sapiens	136.166	6	6	226833	221577
4364	1 Q14807	Q14807	Kinesin-like protein KIF22	KIF22	Homo sapiens	73.262	6	6	18188383	0
4365	1 Q14934	Q14934	Nuclear factor of activated T-cells, cytoplasmic 4	NFATC4	Homo sapiens	95.453	6	6	371574	0
4366	1 Q14978	Q14978	Nucleolar and coiled-body phosphoprotein 1	NOLC1	Homo sapiens	73.604	6	6	3049356	697332
4367	1 Q15005	Q15005	Signal peptidase complex subunit 2	SPCS2	Homo sapiens	25.003	6	6	3136976	1826760
4368	1 Q15022	Q15022	Polycomb protein SUZ12	SUZ12	Homo sapiens	83.056	6	6	308747	203523
4369	1 Q15025	Q15025	TNFAIP3-interacting protein 1	TNIP1	Homo sapiens	71.864	6	6	285779	506374
4370	1 Q15056	Q15056	Eukaryotic translation initiation factor 4H	EIF4H	Homo sapiens	27.385	6	6	15521474	4951886
4371	1 Q15061	Q15061	WD repeat-containing protein 43	WDR43	Homo sapiens	74.889	6	6	1050719	294025
4372	1 Q15121	Q15121	Astrocytic phosphoprotein PEA-15	PEA15	Homo sapiens	15.037	6	6	10449534	1830259
4373	1 Q15345	Q15345	Leucine-rich repeat-containing protein 41	LRRC41	Homo sapiens	88.651	6	6	159491	28313
4374	1 Q15369	Q15369	Elongin-C	ELOC	Homo sapiens	12.473	6	6	6792919	5389109
4375	1 Q15438	Q15438	Cytohesin-1	CYTH1	Homo sapiens	46.412	7	6	227724	726396
4376	1 Q155Q3	Q155Q3	Dixin	DIXDC1	Homo sapiens	77.48	6	6	383972	0
4377	1 Q15723	Q15723	ETS-related transcription factor Elf-2	ELF2	Homo sapiens	63.967	6	6	371520	11740
4378	1 Q15782	Q15782	Chitinase-3-like protein 2	CHI3L2	Homo sapiens	43.502	6	6	0	446259
4379	1 Q16186	Q16186	Proteasomal ubiquitin receptor ADRM1	ADRM1	Homo sapiens	42.153	6	6	1149300	482527
4380	1 Q16576	Q16576	Histone-binding protein RBBP7	RBBP7	Homo sapiens	47.82	9	6	5746198	2516222
4381	1 Q16769	Q16769	Glutaminyl-peptide cyclotransferase	QPCT	Homo sapiens	40.877	6	6	814174	7817712
4382	1 Q16890	Q16890	Tumor protein D53	TPD52L1	Homo sapiens	22.448	6	6	2542978	309478
4383	1 Q17R89	Q17R89	Rho GTPase-activating protein 44	ARHGAP44	Homo sapiens	89.249	6	6	690699	0
4384	1 Q2KHT3	Q2KHT3	Protein CLEC16A	CLEC16A	Homo sapiens	117.718	6	6	329308	115470
4385	1 Q2PZI1	Q2PZI1	Probable C-mannosyltransferase DPY19L1	DPY19L1	Homo sapiens	77.319	7	6	1482558	287785

4386	1	Q2TAL8	Q2TAL8	Transcriptional regulator QRICH1	QRICH1	Homo sapiens	86.436	6	6	1024400	83138
4387	1	Q3KQV9	Q3KQV9	UDP-N-acetylhexosamine pyrophosphorylase-like protein 1	UAP1L1	Homo sapiens	57.03	6	6	1074997	969638
4388	1	Q3MIR4	Q3MIR4	Cell cycle control protein 50B	TMEM30B	Homo sapiens	38.942	6	6	1982601	0
4389	1	Q3SYC2	Q3SYC2	2-acylglycerol O-acyltransferase 2	MOGAT2	Homo sapiens	38.196	6	6	1044361	0
4390	1	Q3ZCQ8	Q3ZCQ8	Mitochondrial import inner membrane translocase subunit TIM50	TIMM50	Homo sapiens	39.647	6	6	2287200	669885
4391	1	Q4J6C6	Q4J6C6	Prolyl endopeptidase-like	PREPL	Homo sapiens	83.929	6	6	396955	0
4392	1	Q4LDE5	Q4LDE5	Sushi, von Willebrand factor type A, EGF and pentraxin domain-containing protein 1	SVEP1	Homo sapiens	390.18	6	6	519265	103903
4393	1	Q53ET0	Q53ET0	CREB-regulated transcription coactivator 2	CRTC2	Homo sapiens	73.304	6	6	1433976	440624
4394	1	Q53EU6	Q53EU6	Glycerol-3-phosphate acyltransferase 3	GPAT3	Homo sapiens	48.707	7	6	616763	1998124
4395	1	Q53GS7	Q53GS7	mRNA export factor GLE1	GLE1	Homo sapiens	79.837	6	6	4877086	3099601
4396	1	Q53GT1	Q53GT1	Kelch-like protein 22	KLHL22	Homo sapiens	71.667	6	6	521139	91340
4397	1	Q53HV7	Q53HV7	Single-strand selective monofunctional uracil DNA glycosylase	SMUG1	Homo sapiens	29.86	6	6	313043	0
4398	1	Q56VL3	Q56VL3	OCIA domain-containing protein 2	OCIAD2	Homo sapiens	16.953	6	6	1472518	811286
4399	1	Q5BJF6	Q5BJF6	Outer dense fiber protein 2	ODF2	Homo sapiens	95.401	6	6	176841	331149
4400	1	Q5C9Z4	Q5C9Z4	Nucleolar MIF4G domain-containing protein 1	NOM1	Homo sapiens	96.259	6	6	186683	114403
4401	1	Q5EBL4	Q5EBL4	RILP-like protein 1	RILPL1	Homo sapiens	47.108	6	6	678130	155254
4402	1	Q5EBM0	Q5EBM0	UMP-CMP kinase 2, mitochondrial	CMPK2	Homo sapiens	49.446	6	6	561798	695214
4403	1	Q5HYI7	Q5HYI7	Metaxin-3	MTX3	Homo sapiens	35.096	6	6	673781	0
4404	1	Q5JSZ5	Q5JSZ5	Protein PRRC2B	PRRC2B	Homo sapiens	242.969	6	6	311771	12083
4405	1	Q5MNZ6	Q5MNZ6	WD repeat domain phosphoinositide-interacting protein 3	WDR45B	Homo sapiens	38.12	6	6	580144	245322
4406	1	Q5ST30	Q5ST30	Valine--tRNA ligase, mitochondrial	VARS2	Homo sapiens	118.49	6	6	531809	34300
4407	1	Q5SW96	Q5SW96	Low density lipoprotein receptor adapter protein 1	LDLRAP1	Homo sapiens	33.883	6	6	613567	117665
4408	1	Q5T0Z8	Q5T0Z8	Uncharacterized protein C6orf132	C6orf132	Homo sapiens	124.037	6	6	340250	0
4409	1	Q5T5X7	Q5T5X7	BEN domain-containing protein 3	BEND3	Homo sapiens	94.477	6	6	169797	0
4410	1	Q5T7M9	Q5T7M9	Divergent protein kinase domain 1A	DIPK1A	Homo sapiens	49.024	6	6	382052	0
4411	1	Q5T9L3	Q5T9L3	Protein wntless homolog	WLS	Homo sapiens	62.254	6	6	1044549	489792
4412	1	Q5TA45	Q5TA45	Integrator complex subunit 11	INTS11	Homo sapiens	67.665	6	6	797225	163899
4413	1	Q5TBB1	Q5TBB1	Ribonuclease H2 subunit B	RNASEH2B	Homo sapiens	35.138	6	6	513118	520283
4414	1	Q5VUJ6	Q5VUJ6	Leucine-rich repeat and calponin homology domain-containing protein 2	LRCH2	Homo sapiens	84.588	6	6	426039	0
4415	1	Q5VV42	Q5VV42	Threonylcarbamoyladenosine tRNA methylthiotransferase	CDKAL1	Homo sapiens	65.115	6	6	453459	44687
4416	1	Q5W111	Q5W111	SPRY domain-containing protein 7	SPRYD7	Homo sapiens	21.664	6	6	629352	475280
4417	1	Q63HM1	Q63HM1	Kynurenine formamidase	AFMID	Homo sapiens	33.991	6	6	292870	503541
4418	1	Q63HQ2	Q63HQ2	Pikachurin	EGFLAM	Homo sapiens	111.272	6	6	47185	413495
4419	1	Q68DA7	Q68DA7	Formin-1	FMN1	Homo sapiens	157.578	6	6	322811	0
4420	1	Q6BCY4	Q6BCY4	NADH-cytochrome b5 reductase 2	CYB5R2	Homo sapiens	31.456	6	6	529046	455783
4421	1	Q6GMV2	Q6GMV2	Histone-lysine N-trimethyltransferase SMYD5	SMYD5	Homo sapiens	47.341	6	6	684588	213247
4422	1	Q6GQQ9	Q6GQQ9	OTU domain-containing protein 7B	OTUD7B	Homo sapiens	92.525	6	6	1485641	0
4423	1	Q6I9Y2	Q6I9Y2	THO complex subunit 7 homolog	THOC7	Homo sapiens	23.742	6	6	363083	93590
4424	1	Q6IA17	Q6IA17	Single Ig IL-1-related receptor	SIGIRR	Homo sapiens	45.679	6	6	562305	109031
4425	1	Q6IQ20	Q6IQ20	N-acyl-phosphatidylethanolamine-hydrolyzing phospholipase D	NAPEPLD	Homo sapiens	45.594	6	6	600567	0
4426	1	Q6P1A2	Q6P1A2	Lysophospholipid acyltransferase 5	LPCAT3	Homo sapiens	56.034	6	6	5174493	4471983
4427	1	Q6P1X6	Q6P1X6	UPF0598 protein C8orf82	C8orf82	Homo sapiens	23.889	6	6	1153559	68053
4428	1	Q6P2M8	Q6P2M8	Calcium/calmodulin-dependent protein kinase type 1B	PNCK	Homo sapiens	38.499	6	6	800999	0
4429	1	Q6P9B9	Q6P9B9	Integrator complex subunit 5	INTS5	Homo sapiens	107.995	6	6	629980	80493
4430	1	Q6PJ69	Q6PJ69	Tripartite motif-containing protein 65	TRIM65	Homo sapiens	57.353	6	6	691524	345673
4431	1	Q6PK18	Q6PK18	2-oxoglutarate and iron-dependent oxygenase domain-containing protein 3	OGFOD3	Homo sapiens	35.645	6	6	652851	77249

4432	1	Q6UUV7	Q6UUV7	CREB-regulated transcription coactivator 3	CRTC3	Homo sapiens	66.959	6	6	558392	44397
4433	1	Q6UW68	Q6UW68	Transmembrane protein 205	TMEM205	Homo sapiens	21.197	6	6	4131584	3714055
4434	1	Q6UX71	Q6UX71	Plexin domain-containing protein 2	PLXDC2	Homo sapiens	59.583	6	6	1884237	417732
4435	1	Q6UXN9	Q6UXN9	WD repeat-containing protein 82	WDR82	Homo sapiens	35.078	6	6	9681215	592020
4436	1	Q6Y288	Q6Y288	Beta-1,3-glucosyltransferase	B3GLCT	Homo sapiens	56.563	6	6	698145	152554
4437	1	Q6ZMJ2	Q6ZMJ2	Scavenger receptor class A member 5	SCARA5	Homo sapiens	53.995	6	6	367012	0
4438	1	Q6ZNB6	Q6ZNB6	NF-X1-type zinc finger protein NFXL1	NFXL1	Homo sapiens	101.341	6	6	423907	0
4439	1	Q6ZRP7	Q6ZRP7	Sulfhydryl oxidase 2	QSOX2	Homo sapiens	77.531	6	6	175526	225103
4440	1	Q6ZSS7	Q6ZSS7	Major facilitator superfamily domain-containing protein 6	MFSD6	Homo sapiens	88.089	6	6	1370957	0
4441	1	Q6ZW31	Q6ZW31	Rho GTPase-activating protein SYDE1	SYDE1	Homo sapiens	79.792	6	6	543620	0
4442	1	Q75T13	Q75T13	GPI inositol-deacylase	PGAP1	Homo sapiens	105.383	6	6	609018	0
4443	1	Q7L9B9	Q7L9B9	Endonuclease/exonuclease/phosphatase family domain-containing protein 1	EEPD1	Homo sapiens	62.4	6	6	867676	289437
4444	1	Q7LG56	Q7LG56	Ribonucleoside-diphosphate reductase subunit M2 B	RRM2B	Homo sapiens	40.74	8	6	714978	292629
4445	1	Q7RTN6	Q7RTN6	STE20-related kinase adapter protein alpha	STRADA	Homo sapiens	48.368	6	6	323629	184865
4446	1	Q7Z3B1	Q7Z3B1	Neuronal growth regulator 1	NEGR1	Homo sapiens	38.719	6	6	6427115	23552
4447	1	Q7Z3E2	Q7Z3E2	Coiled-coil domain-containing protein 186	CCDC186	Homo sapiens	103.685	6	6	452150	35144
4448	1	Q7Z434	Q7Z434	Mitochondrial antiviral-signaling protein	MAVS	Homo sapiens	56.528	6	6	4121112	1080107
4449	1	Q7Z4H3	Q7Z4H3	5'-deoxynucleotidase HDDC2	HDDC2	Homo sapiens	23.39	6	6	1039467	479780
4450	1	Q7Z6J0	Q7Z6J0	E3 ubiquitin-protein ligase SH3RF1	SH3RF1	Homo sapiens	93.131	6	6	357539	0
4451	1	Q7Z6K3	Q7Z6K3	Protein prenyltransferase alpha subunit repeat-containing protein 1	PTAR1	Homo sapiens	46.406	6	6	777075	146077
4452	1	Q86SX6	Q86SX6	Glutaredoxin-related protein 5, mitochondrial	GLRX5	Homo sapiens	16.628	6	6	1871705	192166
4453	1	Q86T03	Q86T03	Type 1 phosphatidylinositol 4,5-bisphosphate 4-phosphatase	PIP4P1	Homo sapiens	29.468	6	6	531487	1112149
4454	1	Q86VM9	Q86VM9	Zinc finger CCCH domain-containing protein 18	ZC3H18	Homo sapiens	106.38	6	6	1177448	112426
4455	1	Q86VP3	Q86VP3	Phosphofurin acidic cluster sorting protein 2	PACS2	Homo sapiens	97.703	6	6	709945	72537
4456	1	Q86WB0	Q86WB0	Zinc finger C3HC-type protein 1	ZC3HC1	Homo sapiens	55.262	6	6	418045	492733
4457	1	Q86WR0	Q86WR0	Coiled-coil domain-containing protein 25	CCDC25	Homo sapiens	24.479	6	6	1087959	275673
4458	1	Q86X76	Q86X76	Deaminated glutathione amidase	NIT1	Homo sapiens	35.896	6	6	3035531	705843
4459	1	Q86Y07	Q86Y07	Serine/threonine-protein kinase VRK2	VRK2	Homo sapiens	58.143	6	6	954195	165582
4460	1	Q86YS6	Q86YS6	Ras-related protein Rab-43	RAB43	Homo sapiens	23.341	6	6	439059	1095511
4461	1	Q8IU85	Q8IU85	Calcium/calmodulin-dependent protein kinase type 1D	CAMK1D	Homo sapiens	42.915	6	6	1036599	78778
4462	1	Q8IUC4	Q8IUC4	Rhophilin-2	RHPN2	Homo sapiens	76.993	10	6	403493	127746
4463	1	Q8IV08	Q8IV08	5'-3' exonuclease PLD3	PLD3	Homo sapiens	54.704	6	6	5433939	2008376
4464	1	Q8IV63	Q8IV63	Inactive serine/threonine-protein kinase VRK3	VRK3	Homo sapiens	52.884	6	6	407088	242173
4465	1	Q8IW35	Q8IW35	Centrosomal protein of 97 kDa	CEP97	Homo sapiens	96.982	6	6	280328	337371
4466	1	Q8IW92	Q8IW92	Beta-galactosidase-1-like protein 2	GLB1L2	Homo sapiens	72.081	6	6	275872	0
4467	1	Q8IWU6	Q8IWU6	Extracellular sulfatase Sulf-1	SULF1	Homo sapiens	101.028	6	6	362400	154832
4468	1	Q8IXL7	Q8IXL7	Methionine-R-sulfoxide reductase B3	MSRB3	Homo sapiens	20.702	6	6	4661147	444153
4469	1	Q8IYU8	Q8IYU8	Calcium uptake protein 2, mitochondrial	MICU2	Homo sapiens	49.669	6	6	928203	561082
4470	1	Q8IZE3	Q8IZE3	Protein-associating with the carboxyl-terminal domain of ezrin	SCYL3	Homo sapiens	82.858	6	6	355096	96438
4471	1	Q8IZQ5	Q8IZQ5	Selenoprotein H	SELENOH	Homo sapiens	13.42	6	6	1670892	813050
4472	1	Q8N128	Q8N128	Protein FAM177A1	FAM177A1	Homo sapiens	23.756	6	6	3023545	1727113
4473	1	Q8N139	Q8N139	ATP-binding cassette sub-family A member 6	ABCA6	Homo sapiens	184.289	6	6	489330	0
4474	1	Q8N264	Q8N264	Rho GTPase-activating protein 24	ARHGAP24	Homo sapiens	84.261	6	6	1521020	96369
4475	1	Q8N3J6	Q8N3J6	Cell adhesion molecule 2	CADM2	Homo sapiens	47.552	6	6	913848	0
4476	1	Q8N423	Q8N423	Leukocyte immunoglobulin-like receptor subfamily B member 2	LILRB2	Homo sapiens	65.003	10	6	64791	944702
4477	1	Q8N4C6	Q8N4C6	Ninein	NIN	Homo sapiens	243.251	6	6	46971	326860
4478	1	Q8N4T8	Q8N4T8	3-oxoacyl-[acyl-carrier-protein] reductase	CBR4	Homo sapiens	25.298	6	6	1241807	127283

4479	1	Q8N653	Q8N653	Leucine-zipper-like transcriptional regulator 1	LZTR1	Homo sapiens	94.721	6	6	269034	38616
4480	1	Q8N806	Q8N806	Putative E3 ubiquitin-protein ligase UBR7	UBR7	Homo sapiens	48	6	6	1027516	61920
4481	1	Q8N983	Q8N983	39S ribosomal protein L43, mitochondrial	MRPL43	Homo sapiens	23.432	6	6	1884663	369888
4482	1	Q8N9N7	Q8N9N7	Leucine-rich repeat-containing protein 57	LRRC57	Homo sapiens	26.754	6	6	3758485	990031
4483	1	Q8NB78	Q8NB78	Lysine-specific histone demethylase 2	KDM1B	Homo sapiens	92.099	6	6	288864	262286
4484	1	Q8NBM4	Q8NBM4	Ubiquitin-associated domain-containing protein 2	UBAC2	Homo sapiens	38.963	6	6	362601	232159
4485	1	Q8NBM8	Q8NBM8	Prenylcysteine oxidase-like	PCYOX1L	Homo sapiens	54.647	6	6	597223	530056
4486	1	Q8NBQ5	Q8NBQ5	Estradiol 17-beta-dehydrogenase 11	HSD17B11	Homo sapiens	32.935	6	6	9502141	9567106
4487	1	Q8NBZ7	Q8NBZ7	UDP-glucuronic acid decarboxylase 1	UXS1	Homo sapiens	47.579	6	6	437006	413335
4488	1	Q8NC42	Q8NC42	E3 ubiquitin-protein ligase RNF149	RNF149	Homo sapiens	43.163	6	6	169320	1339414
4489	1	Q8NCG7	Q8NCG7	Diacylglycerol lipase-beta	DAGLB	Homo sapiens	73.733	6	6	268572	1307319
4490	1	Q8NCL4	Q8NCL4	Polypeptide N-acetylgalactosaminyltransferase 6	GALNT6	Homo sapiens	71.159	6	6	181116	402241
4491	1	Q8NEF9	Q8NEF9	Serum response factor-binding protein 1	SRFBP1	Homo sapiens	48.635	6	6	649932	0
4492	1	Q8NEG4	Q8NEG4	Protein FAM83F	FAM83F	Homo sapiens	55.485	6	6	385038	0
4493	1	Q8NEJ9	Q8NEJ9	Neuroguidin	NGDN	Homo sapiens	35.895	6	6	426948	0
4494	1	Q8NFG4	Q8NFG4	Folliculin	FLCN	Homo sapiens	64.475	6	6	443755	193458
4495	1	Q8NFH3	Q8NFH3	Nucleoporin Nup43	NUP43	Homo sapiens	42.149	6	6	1673860	1071478
4496	1	Q8NFH5	Q8NFH5	Nucleoporin NUP35	NUP35	Homo sapiens	34.772	6	6	1555213	403232
4497	1	Q8NHJ6	Q8NHJ6	Leukocyte immunoglobulin-like receptor subfamily B member 4	LILRB4	Homo sapiens	49.358	6	6	71441	1043343
4498	1	Q8NHP8	Q8NHP8	Putative phospholipase B-like 2	PLBD2	Homo sapiens	65.469	6	6	1762175	561622
4499	1	Q8NI37	Q8NI37	Protein phosphatase PTC7 homolog	PPTC7	Homo sapiens	32.646	6	6	654693	45920
4500	1	Q8TB37	Q8TB37	Iron-sulfur protein NUBPL	NUBPL	Homo sapiens	34.084	6	6	1070783	0
4501	1	Q8TB52	Q8TB52	F-box only protein 30	FBXO30	Homo sapiens	82.306	6	6	687895	24039
4502	1	Q8TB96	Q8TB96	T-cell immunomodulatory protein	ITFG1	Homo sapiens	68.108	6	6	905840	339817
4503	1	Q8TBB5	Q8TBB5	Kelch domain-containing protein 4	KLHDC4	Homo sapiens	57.893	6	6	647184	172217
4504	1	Q8TBC3	Q8TBC3	SH3KBP1-binding protein 1	SHKBP1	Homo sapiens	76.345	8	6	476985	449726
4505	1	Q8TBM8	Q8TBM8	DnaJ homolog subfamily B member 14	DNAJB14	Homo sapiens	42.515	6	6	391196	246410
4506	1	Q8TCG2	Q8TCG2	Phosphatidylinositol 4-kinase type 2-beta	PI4K2B	Homo sapiens	54.745	6	6	246576	84130
4507	1	Q8TCT9	Q8TCT9	Minor histocompatibility antigen H13	HM13	Homo sapiens	41.489	6	6	10305354	19223889
4508	1	Q8TDH9	Q8TDH9	Biogenesis of lysosome-related organelles complex 1 subunit 5	BLOC1S5	Homo sapiens	21.61	6	6	972655	522481
4509	1	Q8WU39	Q8WU39	Marginal zone B- and B1-cell-specific protein	MZB1	Homo sapiens	20.693	6	6	11277156	245936
4510	1	Q8WUA2	Q8WUA2	Peptidyl-prolyl cis-trans isomerase-like 4	PPIL4	Homo sapiens	57.225	6	6	1686200	235763
4511	1	Q8WUA7	Q8WUA7	TBC1 domain family member 22A	TBC1D22A	Homo sapiens	59.121	6	6	1224543	468681
4512	1	Q8WUD1	Q8WUD1	Ras-related protein Rab-2B	RAB2B	Homo sapiens	24.213	6	6	824241	107296
4513	1	Q8WUK0	Q8WUK0	Phosphatidylglycerophosphatase and protein-tyrosine phosphatase 1	PTPMT1	Homo sapiens	22.843	6	6	540623	145451
4514	1	Q8WUY1	Q8WUY1	Protein THEM6	THEM6	Homo sapiens	23.864	6	6	1788603	724043
4515	1	Q8WV22	Q8WV22	Non-structural maintenance of chromosomes element 1 homolog	NSMCE1	Homo sapiens	30.856	6	6	359700	130797
4516	1	Q8WVK2	Q8WVK2	U4/U6.U5 small nuclear ribonucleoprotein 27 kDa protein	SNRNP27	Homo sapiens	18.86	6	6	1652641	347764
4517	1	Q8WVX9	Q8WVX9	Fatty acyl-CoA reductase 1	FAR1	Homo sapiens	59.358	6	6	499038	392861
4518	1	Q8WVY7	Q8WVY7	Ubiquitin-like domain-containing CTD phosphatase 1	UBLCP1	Homo sapiens	36.805	6	6	1426350	454774
4519	1	Q8WWH5	Q8WWH5	Pseudouridylate synthase TRUB1	TRUB1	Homo sapiens	37.251	6	6	551574	67179
4520	1	Q8WWV3	Q8WWV3	Reticulon-4-interacting protein 1, mitochondrial	RTN4IP1	Homo sapiens	43.589	6	6	1360555	120307
4521	1	Q8WX92	Q8WX92	Negative elongation factor B	NELFB	Homo sapiens	65.699	6	6	1703502	643096
4522	1	Q8WXA9	Q8WXA9	Splicing regulatory glutamine/lysine-rich protein 1	SREK1	Homo sapiens	59.381	6	6	1793182	288841
4523	1	Q8WZ82	Q8WZ82	Esterase OVCA2	OVCA2	Homo sapiens	24.416	6	6	957536	491714
4524	1	Q92520	Q92520	Protein FAM3C	FAM3C	Homo sapiens	24.68	6	6	4431722	1407321
4525	1	Q92522	Q92522	Histone H1.10	H1-10	Homo sapiens	22.484	6	6	7346712	8232994
4526	1	Q92540	Q92540	Nonsense-mediated mRNA decay factor SMG7	SMG7	Homo sapiens	127.282	6	6	227192	275271

4527	1	Q92542	Q92542	Nicastrin	NCSTN	Homo sapiens	78.411	6	6	2367240	4400379
4528	1	Q92551	Q92551	Inositol hexakisphosphate kinase 1	IP6K1	Homo sapiens	50.236	6	6	614324	13371
4529	1	Q92600	Q92600	CCR4-NOT transcription complex subunit 9	CNOT9	Homo sapiens	33.631	6	6	1376814	548785
4530	1	Q92643	Q92643	GPI-anchor transamidase	PIGK	Homo sapiens	45.252	6	6	1561719	1174250
4531	1	Q92665	Q92665	28S ribosomal protein S31, mitochondrial	MRPS31	Homo sapiens	45.318	6	6	2190775	61218
4532	1	Q92783	Q92783	Signal transducing adapter molecule 1	STAM	Homo sapiens	59.182	6	6	2326995	959343
4533	1	Q92870	Q92870	Amyloid beta precursor protein binding family B member 2	APBB2	Homo sapiens	83.375	6	6	269593	0
4534	1	Q92979	Q92979	Ribosomal RNA small subunit methyltransferase NEP1	EMG1	Homo sapiens	26.718	6	6	1084787	200528
4535	1	Q92990	Q92990	Glomulin	GLMN	Homo sapiens	68.208	6	6	982749	361525
4536	1	Q93099	Q93099	Homogentisate 1,2-dioxygenase	HGD	Homo sapiens	49.964	6	6	483820	69589
4537	1	Q969F9	Q969F9	BLOC-2 complex member HPS3	HPS3	Homo sapiens	113.738	6	6	1312241	326800
4538	1	Q969T3	Q969T3	Sorting nexin-21	SNX21	Homo sapiens	41.366	6	6	379461	0
4539	1	Q969T7	Q969T7	7-methylguanosine phosphate-specific 5'-nucleotidase	NT5C3B	Homo sapiens	34.387	6	6	475582	0
4540	1	Q969T9	Q969T9	WW domain-binding protein 2	WBP2	Homo sapiens	28.086	6	6	4615890	4221093
4541	1	Q969U6	Q969U6	F-box/WD repeat-containing protein 5	FBXW5	Homo sapiens	63.923	6	6	467922	32265
4542	1	Q969Z3	Q969Z3	Mitochondrial amidoxime reducing component 2	MTARC2	Homo sapiens	38.023	7	6	2113566	0
4543	1	Q96A26	Q96A26	Protein FAM162A	FAM162A	Homo sapiens	17.339	6	6	8019337	822549
4544	1	Q96B18	Q96B18	Dapper homolog 3	DACT3	Homo sapiens	64.951	6	6	598773	0
4545	1	Q96BJ8	Q96BJ8	Engulfment and cell motility protein 3	ELMO3	Homo sapiens	81.468	6	6	1398816	42281
4546	1	Q96BZ9	Q96BZ9	TBC1 domain family member 20	TBC1D20	Homo sapiens	45.855	6	6	455606	74323
4547	1	Q96CC6	Q96CC6	Inactive rhomboid protein 1	RHBDF1	Homo sapiens	97.402	6	6	552628	0
4548	1	Q96CN7	Q96CN7	Isochorismatase domain-containing protein 1	ISOC1	Homo sapiens	32.237	6	6	9965576	954334
4549	1	Q96D46	Q96D46	60S ribosomal export protein NMD3	NMD3	Homo sapiens	57.603	6	6	1139352	314530
4550	1	Q96DE0	Q96DE0	U8 snoRNA-decapping enzyme	NUDT16	Homo sapiens	21.274	7	6	1531893	440771
4551	1	Q96DG6	Q96DG6	Carboxymethylenebutenolidase homolog	CMBL	Homo sapiens	28.045	6	6	2761623	702114
4552	1	Q96DU7	Q96DU7	Inositol-trisphosphate 3-kinase C	ITPKC	Homo sapiens	75.209	6	6	216301	390360
4553	1	Q96ED9	Q96ED9	Protein Hook homolog 2	HOOK2	Homo sapiens	83.207	6	6	272620	0
4554	1	Q96EI5	Q96EI5	Transcription elongation factor A protein-like 4	TCEAL4	Homo sapiens	24.648	7	6	555446	0
4555	1	Q96EK9	Q96EK9	Protein KTI12 homolog	KTI12	Homo sapiens	38.613	6	6	336578	10079
4556	1	Q96EM0	Q96EM0	Trans-3-hydroxy-L-proline dehydratase	L3HYPDH	Homo sapiens	38.138	6	6	1034764	121178
4557	1	Q96EV8	Q96EV8	Dysbindin	DTNBP1	Homo sapiens	39.493	6	6	650143	202254
4558	1	Q96F15	Q96F15	GTPase IMAP family member 5	GIMAP5	Homo sapiens	34.847	6	6	519692	452966
4559	1	Q96G23	Q96G23	Ceramide synthase 2	CERS2	Homo sapiens	44.876	6	6	1149907	948139
4560	1	Q96GG9	Q96GG9	DCN1-like protein 1	DCUN1D1	Homo sapiens	30.122	7	6	1557734	785046
4561	1	Q96GW9	Q96GW9	Methionine--tRNA ligase, mitochondrial	MARS2	Homo sapiens	66.593	6	6	508826	83158
4562	1	Q96IU4	Q96IU4	Putative protein-lysine deacylase ABHD14B	ABHD14B	Homo sapiens	22.344	6	6	10600419	1666756
4563	1	Q96K19	Q96K19	E3 ubiquitin-protein ligase RNF170	RNF170	Homo sapiens	29.813	6	6	916216	80746
4564	1	Q96KN4	Q96KN4	Protein LRATD1	LRATD1	Homo sapiens	32.491	6	6	468774	0
4565	1	Q96LJ7	Q96LJ7	Dehydrogenase/reductase SDR family member 1	DHRS1	Homo sapiens	33.908	6	6	2822129	1687956
4566	1	Q96MW5	Q96MW5	Conserved oligomeric Golgi complex subunit 8	COG8	Homo sapiens	68.424	6	6	470568	284137
4567	1	Q96MX6	Q96MX6	Dynein axonemal assembly factor 10	DNAAF10	Homo sapiens	39.743	6	6	648430	54921
4568	1	Q96N66	Q96N66	Lysophospholipid acyltransferase 7	MBOAT7	Homo sapiens	52.764	6	6	3471779	7227923
4569	1	Q96P16	Q96P16	Regulation of nuclear pre-mRNA domain-containing protein 1A	RPRD1A	Homo sapiens	35.719	6	6	968684	415351
4570	1	Q96PB1	Q96PB1	N-acetylneuraminate 9-O-acetyltransferase	CASD1	Homo sapiens	91.68	6	6	745551	0
4571	1	Q96PV6	Q96PV6	Leukocyte receptor cluster member 8	LENG8	Homo sapiens	88.157	6	6	214276	74505
4572	1	Q96Q42	Q96Q42	Alsin	ALS2	Homo sapiens	183.635	6	6	106332	83521
4573	1	Q96QD9	Q96QD9	UAP56-interacting factor	FYTTD1	Homo sapiens	35.816	6	6	1252009	475693
4574	1	Q96S44	Q96S44	EKC/KEOPS complex subunit TP53RK	TP53RK	Homo sapiens	28.16	6	6	1996235	1183748

4575	1	Q96SB8	Q96SB8	Structural maintenance of chromosomes protein 6	SMC6	Homo sapiens	126.328	6	6	94570	228937
4576	1	Q96SW2	Q96SW2	Protein cereblon	CRBN	Homo sapiens	50.546	6	6	639114	206755
4577	1	Q96T88	Q96T88	E3 ubiquitin-protein ligase UHRF1	UHRF1	Homo sapiens	89.815	6	6	212924	34239
4578	1	Q99442	Q99442	Translocation protein SEC62	SEC62	Homo sapiens	45.863	6	6	1514373	602202
4579	1	Q99496	Q99496	E3 ubiquitin-protein ligase RING2	RNF2	Homo sapiens	37.655	8	6	310832	190187
4580	1	Q99808	Q99808	Equilibrative nucleoside transporter 1	SLC29A1	Homo sapiens	50.222	6	6	1968343	49921
4581	1	Q99933	Q99933	BAG family molecular chaperone regulator 1	BAG1	Homo sapiens	38.778	6	6	1125368	742245
4582	1	Q99961	Q99961	Endophilin-A2	SH3GL1	Homo sapiens	41.49	7	6	5306684	2900844
4583	1	Q99988	Q99988	Growth/differentiation factor 15	GDF15	Homo sapiens	34.14	6	6	933227	9547760
4584	1	Q9BPX5	Q9BPX5	Actin-related protein 2/3 complex subunit 5-like protein	ARPC5L	Homo sapiens	16.941	6	6	3544969	916710
4585	1	Q9BPY3	Q9BPY3	Protein FAM118B	FAM118B	Homo sapiens	39.497	6	6	1995405	0
4586	1	Q9BQ61	Q9BQ61	Telomerase RNA component interacting RNase	TRIR	Homo sapiens	18.417	6	6	3346186	1179737
4587	1	Q9BQ67	Q9BQ67	Glutamate-rich WD repeat-containing protein 1	GRWD1	Homo sapiens	49.42	6	6	2950896	35624
4588	1	Q9BQ95	Q9BQ95	Evolutionarily conserved signaling intermediate in Toll pathway, mitochondrial	ECSIT	Homo sapiens	49.15	6	6	825406	188193
4589	1	Q9BQE4	Q9BQE4	Selenoprotein S	SELENOS	Homo sapiens	21.131	6	6	1372472	172489
4590	1	Q9BRJ2	Q9BRJ2	39S ribosomal protein L45, mitochondrial	MRPL45	Homo sapiens	35.352	6	6	3933490	1230819
4591	1	Q9BRR8	Q9BRR8	G patch domain-containing protein 1	GPATCH1	Homo sapiens	103.345	6	6	314250	0
4592	1	Q9BRV8	Q9BRV8	Suppressor of IKBKE 1	SIKE1	Homo sapiens	23.719	6	6	381937	113822
4593	1	Q9BSC4	Q9BSC4	Nucleolar protein 10	NOL10	Homo sapiens	80.3	6	6	1373392	0
4594	1	Q9BSE5	Q9BSE5	Agmatinase, mitochondrial	AGMAT	Homo sapiens	37.662	6	6	3487223	40852
4595	1	Q9BSL1	Q9BSL1	Ubiquitin-associated domain-containing protein 1	UBAC1	Homo sapiens	45.338	6	6	1682894	624752
4596	1	Q9BSQ5	Q9BSQ5	Cerebral cavernous malformations 2 protein	CCM2	Homo sapiens	48.836	6	6	68664	360174
4597	1	Q9BT09	Q9BT09	Protein canopy homolog 3	CNPY3	Homo sapiens	30.749	6	6	1049681	2322100
4598	1	Q9BT40	Q9BT40	Inositol polyphosphate 5-phosphatase K	INPP5K	Homo sapiens	51.092	6	6	594508	255673
4599	1	Q9BTA9	Q9BTA9	WW domain-containing adapter protein with coiled-coil	WAC	Homo sapiens	70.725	6	6	457518	35744
4600	1	Q9BTY7	Q9BTY7	Protein HGH1 homolog	HGH1	Homo sapiens	42.131	6	6	1040704	371895
4601	1	Q9BU02	Q9BU02	Thiamine-triphosphatase	THTPA	Homo sapiens	25.565	6	6	1075641	357156
4602	1	Q9BUH6	Q9BUH6	Protein PAXX	PAXX	Homo sapiens	21.638	6	6	1382898	630983
4603	1	Q9BUI4	Q9BUI4	DNA-directed RNA polymerase III subunit RPC3	POLR3C	Homo sapiens	60.612	6	6	586804	18827
4604	1	Q9BUP0	Q9BUP0	EF-hand domain-containing protein D1	EFHD1	Homo sapiens	26.927	6	6	674826	0
4605	1	Q9BV19	Q9BV19	Uncharacterized protein C1orf50	C1orf50	Homo sapiens	21.876	6	6	551369	120659
4606	1	Q9BV23	Q9BV23	Monoacylglycerol lipase ABHD6	ABHD6	Homo sapiens	38.33	6	6	668463	126669
4607	1	Q9BV57	Q9BV57	Acireductone dioxygenase	ADI1	Homo sapiens	21.498	6	6	592094	174707
4608	1	Q9BVK6	Q9BVK6	Transmembrane emp24 domain-containing protein 9	TMED9	Homo sapiens	27.277	7	6	8552822	7675412
4609	1	Q9BVL4	Q9BVL4	Protein adenylyltransferase SelO, mitochondrial	SELENOO	Homo sapiens	73.458	6	6	460111	210914
4610	1	Q9BW85	Q9BW85	Splicing factor YJU2	YJU2	Homo sapiens	37.086	6	6	361506	0
4611	1	Q9BWN1	Q9BWN1	Proline-rich protein 14	PRR14	Homo sapiens	64.328	7	6	557600	156277
4612	1	Q9BX10	Q9BX10	GTP-binding protein 2	GTPBP2	Homo sapiens	65.767	6	6	158716	410048
4613	1	Q9BX59	Q9BX59	Tapasin-related protein	TAPBPL	Homo sapiens	50.183	6	6	1621738	882702
4614	1	Q9BY15	Q9BY15	Adhesion G protein-coupled receptor E3	ADGRE3	Homo sapiens	72.621	6	6	0	1292993
4615	1	Q9BY32	Q9BY32	Inosine triphosphate pyrophosphatase	ITPA	Homo sapiens	21.445	6	6	1992155	1139459
4616	1	Q9BYD3	Q9BYD3	39S ribosomal protein L4, mitochondrial	MRPL4	Homo sapiens	34.917	6	6	1656896	271240
4617	1	Q9BYN8	Q9BYN8	28S ribosomal protein S26, mitochondrial	MRPS26	Homo sapiens	24.212	6	6	1344627	209407
4618	1	Q9C037	Q9C037	E3 ubiquitin-protein ligase TRIM4	TRIM4	Homo sapiens	57.462	6	6	1050409	709431
4619	1	Q9C0B5	Q9C0B5	Palmitoyltransferase ZDHHC5	ZDHHC5	Homo sapiens	77.544	6	6	816308	113225
4620	1	Q9H0A8	Q9H0A8	COMM domain-containing protein 4	COMMD4	Homo sapiens	21.765	6	6	1091225	471250
4621	1	Q9H0C8	Q9H0C8	Integrin-linked kinase-associated serine/threonine phosphatase 2C	ILKAP	Homo sapiens	42.908	6	6	1395312	747118

4622	1	Q9H0E9	Q9H0E9	Bromodomain-containing protein 8	BRD8	Homo sapiens	135.338	6	6	293648	123463
4623	1	Q9H0U3	Q9H0U3	Magnesium transporter protein 1	MAGT1	Homo sapiens	38.038	6	6	1660763	1699443
4624	1	Q9H0U6	Q9H0U6	39S ribosomal protein L18, mitochondrial	MRPL18	Homo sapiens	20.576	6	6	1349284	302598
4625	1	Q9H2J4	Q9H2J4	Phosducin-like protein 3	PDCL3	Homo sapiens	27.614	6	6	1440094	234650
4626	1	Q9H330	Q9H330	Transmembrane protein 245	TMEM245	Homo sapiens	97.356	6	6	2551772	354988
4627	1	Q9H3M7	Q9H3M7	Thioredoxin-interacting protein	TXNIP	Homo sapiens	43.662	6	6	903619	377228
4628	1	Q9H3Q3	Q9H3Q3	Galactose-3-O-sulfotransferase 2	GAL3ST2	Homo sapiens	46.112	6	6	1158911	0
4629	1	Q9H3S1	Q9H3S1	Semaphorin-4A	SEMA4A	Homo sapiens	83.573	6	6	26589	654670
4630	1	Q9H444	Q9H444	Charged multivesicular body protein 4b	CHMP4B	Homo sapiens	24.951	7	6	14753599	11046471
4631	1	Q9H4L7	Q9H4L7	SWI/SNF-related matrix-associated actin-dependent regulator of chromatin subfamily A containing DEAD/H box 1	SMARCAD1	Homo sapiens	117.403	6	6	427315	11357
4632	1	Q9H5H4	Q9H5H4	Zinc finger protein 768	ZNF768	Homo sapiens	60.227	6	6	383795	17899
4633	1	Q9H5Z1	Q9H5Z1	Probable ATP-dependent RNA helicase DHX35	DHX35	Homo sapiens	78.91	6	6	476521	87549
4634	1	Q9H6S1	Q9H6S1	5-azacytidine-induced protein 2	AZI2	Homo sapiens	44.932	6	6	267923	184945
4635	1	Q9H7B2	Q9H7B2	Ribosome production factor 2 homolog	RPF2	Homo sapiens	35.585	6	6	622203	197431
4636	1	Q9H7E2	Q9H7E2	Tudor domain-containing protein 3	TDRD3	Homo sapiens	73.185	6	6	559464	99460
4637	1	Q9H7Z3	Q9H7Z3	Nuclear exosome regulator NRDE2	NRDE2	Homo sapiens	132.675	6	6	403122	0
4638	1	Q9H814	Q9H814	Phosphorylated adapter RNA export protein	PHAX	Homo sapiens	44.4	6	6	770357	84998
4639	1	Q9H871	Q9H871	E3 ubiquitin-protein transferase RMND5A	RMND5A	Homo sapiens	43.992	6	6	384400	34908
4640	1	Q9H8W4	Q9H8W4	Pleckstrin homology domain-containing family F member 2	PLEKHF2	Homo sapiens	27.799	6	6	1314279	1223183
4641	1	Q9H910	Q9H910	Jupiter microtubule associated homolog 2	JPT2	Homo sapiens	20.063	6	6	4352955	1081096
4642	1	Q9H9J2	Q9H9J2	39S ribosomal protein L44, mitochondrial	MRPL44	Homo sapiens	37.536	6	6	1200110	41940
4643	1	Q9HAD4	Q9HAD4	WD repeat-containing protein 41	WDR41	Homo sapiens	51.73	6	6	342634	382369
4644	1	Q9HAP2	Q9HAP2	MLX-interacting protein	MLXIP	Homo sapiens	101.184	6	6	591207	0
4645	1	Q9HAR2	Q9HAR2	Adhesion G protein-coupled receptor L3	ADGRL3	Homo sapiens	161.813	6	6	709448	0
4646	1	Q9HAV7	Q9HAV7	GrpE protein homolog 1, mitochondrial	GRPEL1	Homo sapiens	24.281	6	6	3267110	2530789
4647	1	Q9HBH5	Q9HBH5	Retinol dehydrogenase 14	RDH14	Homo sapiens	36.865	6	6	1353204	717596
4648	1	Q9HBI1	Q9HBI1	Beta-parvin	PARVB	Homo sapiens	41.715	6	6	688253	846043
4649	1	Q9HCD6	Q9HCD6	Protein TANC2	TANC2	Homo sapiens	219.653	6	6	162847	391779
4650	1	Q9HCJ3	Q9HCJ3	Ribonucleoprotein PTB-binding 2	RAVER2	Homo sapiens	74.339	6	6	880618	14764
4651	1	Q9NP58	Q9NP58	ATP-binding cassette sub-family B member 6	ABCB6	Homo sapiens	93.885	6	6	260769	147665
4652	1	Q9NP74	Q9NP74	Palmdelphin	PALMD	Homo sapiens	62.759	6	6	615713	0
4653	1	Q9NP79	Q9NP79	Vacuolar protein sorting-associated protein VTA1 homolog	VTA1	Homo sapiens	33.879	6	6	4720562	3345482
4654	1	Q9NP80	Q9NP80	Calcium-independent phospholipase A2-gamma	PNPLA8	Homo sapiens	88.478	6	6	1376135	888801
4655	1	Q9NPA0	Q9NPA0	ER membrane protein complex subunit 7	EMC7	Homo sapiens	26.471	6	6	1171861	822451
4656	1	Q9NPA2	Q9NPA2	Matrix metalloproteinase-25	MMP25	Homo sapiens	62.556	6	6	0	4128403
4657	1	Q9NPF4	Q9NPF4	tRNA N6-adenosine threonylcarbamoyltransferase	OSGEP	Homo sapiens	36.426	6	6	2205815	1235082
4658	1	Q9NPJ6	Q9NPJ6	Mediator of RNA polymerase II transcription subunit 4	MED4	Homo sapiens	29.744	6	6	731305	141070
4659	1	Q9NPY3	Q9NPY3	Complement component C1q receptor	CD93	Homo sapiens	68.56	6	6	316636	1367555
4660	1	Q9NQ50	Q9NQ50	39S ribosomal protein L40, mitochondrial	MRPL40	Homo sapiens	24.491	6	6	1206880	265977
4661	1	Q9NQ79	Q9NQ79	Cartilage acidic protein 1	CRTAC1	Homo sapiens	71.422	6	6	649814	516472
4662	1	Q9NQ88	Q9NQ88	Fructose-2,6-bisphosphatase TIGAR	TIGAR	Homo sapiens	30.062	6	6	802095	934940
4663	1	Q9NQS5	Q9NQS5	G-protein coupled receptor 84	GPR84	Homo sapiens	43.702	6	6	0	1453355
4664	1	Q9NR28	Q9NR28	Diablo IAP-binding mitochondrial protein	DIABLO	Homo sapiens	27.13	6	6	3013616	340648
4665	1	Q9NRD1	Q9NRD1	F-box only protein 6	FBXO6	Homo sapiens	33.934	6	6	276746	846223
4666	1	Q9NRS6	Q9NRS6	Sorting nexin-15	SNX15	Homo sapiens	38.291	6	6	588906	206209
4667	1	Q9NRX1	Q9NRX1	RNA-binding protein PNO1	PNO1	Homo sapiens	27.922	6	6	563965	137831
4668	1	Q9NSI8	Q9NSI8	SAM domain-containing protein SAMSN-1	SAMSN1	Homo sapiens	41.708	6	6	17282	795610

4669	1	Q9NUM4	Q9NUM4	Transmembrane protein 106B	TMEM106B	Homo sapiens	31.13	6	6	1023159	1053195
4670	1	Q9NUQ3	Q9NUQ3	Gamma-taxilin	TXLNG	Homo sapiens	60.585	7	6	577037	153979
4671	1	Q9NUT2	Q9NUT2	Mitochondrial potassium channel ATP-binding subunit	ABCB8	Homo sapiens	79.989	6	6	5170939	6303197
4672	1	Q9NUW8	Q9NUW8	Tyrosyl-DNA phosphodiesterase 1	TDP1	Homo sapiens	68.42	6	6	255917	314612
4673	1	Q9NVJ2	Q9NVJ2	ADP-ribosylation factor-like protein 8B	ARL8B	Homo sapiens	21.538	11	6	7498307	18029927
4674	1	Q9NVU7	Q9NVU7	Protein SDA1 homolog	SDAD1	Homo sapiens	79.872	6	6	522670	303117
4675	1	Q9NVV4	Q9NVV4	Poly(A) RNA polymerase, mitochondrial	MTPAP	Homo sapiens	66.172	6	6	890880	82985
4676	1	Q9NVX2	Q9NVX2	Notchless protein homolog 1	NLE1	Homo sapiens	53.321	6	6	540710	147865
4677	1	Q9NW64	Q9NW64	Pre-mRNA-splicing factor RBM22	RBM22	Homo sapiens	46.896	6	6	1485577	656030
4678	1	Q9NWS0	Q9NWS0	PIH1 domain-containing protein 1	PIH1D1	Homo sapiens	32.361	6	6	792190	189606
4679	1	Q9NWU5	Q9NWU5	39S ribosomal protein L22, mitochondrial	MRPL22	Homo sapiens	23.64	6	6	3729639	1399229
4680	1	Q9NX20	Q9NX20	39S ribosomal protein L16, mitochondrial	MRPL16	Homo sapiens	28.449	6	6	1064456	109814
4681	1	Q9NXS2	Q9NXS2	Glutaminyl-peptide cyclotransferase-like protein	QPCTL	Homo sapiens	42.923	6	6	566432	572863
4682	1	Q9NYU1	Q9NYU1	UDP-glucose:glycoprotein glucosyltransferase 2	UGGT2	Homo sapiens	174.739	6	6	39991	304767
4683	1	Q9NZ01	Q9NZ01	Very-long-chain enoyl-CoA reductase	TECR	Homo sapiens	36.037	6	6	8462363	3176349
4684	1	Q9NZD2	Q9NZD2	Glycolipid transfer protein	GLTP	Homo sapiens	23.849	6	6	2521653	1341305
4685	1	Q9P015	Q9P015	39S ribosomal protein L15, mitochondrial	MRPL15	Homo sapiens	33.417	6	6	2115698	360416
4686	1	Q9P032	Q9P032	NADH dehydrogenase [ubiquinone] 1 alpha subcomplex assembly factor 4	NDUFAF4	Homo sapiens	20.267	6	6	2733530	173974
4687	1	Q9P035	Q9P035	Very-long-chain (3R)-3-hydroxyacyl-CoA dehydratase 3	HACD3	Homo sapiens	43.16	6	6	3432136	1420889
4688	1	Q9P0M9	Q9P0M9	39S ribosomal protein L27, mitochondrial	MRPL27	Homo sapiens	16.072	6	6	1531333	871211
4689	1	Q9P0U3	Q9P0U3	Sentrin-specific protease 1	SENP1	Homo sapiens	73.481	6	6	422868	128858
4690	1	Q9P0V3	Q9P0V3	SH3 domain-binding protein 4	SH3BP4	Homo sapiens	107.496	6	6	443868	0
4691	1	Q9P1Q0	Q9P1Q0	Vacuolar protein sorting-associated protein 54	VPS54	Homo sapiens	110.593	6	6	175772	443177
4692	1	Q9P2A4	Q9P2A4	ABI gene family member 3	ABI3	Homo sapiens	39.034	6	6	973080	268605
4693	1	Q9UBQ5	Q9UBQ5	Eukaryotic translation initiation factor 3 subunit K	EIF3K	Homo sapiens	25.058	6	6	5053524	2657133
4694	1	Q9UBX3	Q9UBX3	Mitochondrial dicarboxylate carrier	SLC25A10	Homo sapiens	31.281	6	6	1783905	1257447
4695	1	Q9UEW8	Q9UEW8	STE20/SPS1-related proline-alanine-rich protein kinase	STK39	Homo sapiens	59.475	6	6	1434631	389805
4696	1	Q9UG01	Q9UG01	Intraflagellar transport protein 172 homolog	IFT172	Homo sapiens	197.58	6	6	287202	0
4697	1	Q9UGJ0	Q9UGJ0	5'-AMP-activated protein kinase subunit gamma-2	PRKAG2	Homo sapiens	63.065	7	6	552370	40156
4698	1	Q9UGT4	Q9UGT4	Sushi domain-containing protein 2	SUSD2	Homo sapiens	90.209	6	6	864387	364701
4699	1	Q9UH03	Q9UH03	Neuronal-specific septin-3	SEPTIN3	Homo sapiens	40.7	6	6	412643	36381
4700	1	Q9UHG2	Q9UHG2	ProSAAS	PCSK1N	Homo sapiens	27.369	6	6	2567947	0
4701	1	Q9UHW9	Q9UHW9	Solute carrier family 12 member 6	SLC12A6	Homo sapiens	127.62	8	6	139750	312824
4702	1	Q9UI15	Q9UI15	Transgelin-3	TAGLN3	Homo sapiens	22.471	6	6	2375246	0
4703	1	Q9UIC8	Q9UIC8	Leucine carboxyl methyltransferase 1	LCMT1	Homo sapiens	38.38	6	6	1359640	262296
4704	1	Q9UIV1	Q9UIV1	CCR4-NOT transcription complex subunit 7	CNOT7	Homo sapiens	32.744	6	6	855622	485564
4705	1	Q9UKA9	Q9UKA9	Polypyrimidine tract-binding protein 2	PTBP2	Homo sapiens	57.491	6	6	2206518	0
4706	1	Q9UKM9	Q9UKM9	RNA-binding protein Raly	RALY	Homo sapiens	32.463	6	6	15970692	6188540
4707	1	Q9UKW4	Q9UKW4	Guanine nucleotide exchange factor VAV3	VAV3	Homo sapiens	97.777	6	6	57986	490222
4708	1	Q9UKY7	Q9UKY7	Protein CDV3 homolog	CDV3	Homo sapiens	27.335	6	6	3008639	792104
4709	1	Q9UL03	Q9UL03	Integrator complex subunit 6	INTS6	Homo sapiens	100.392	7	6	386848	108358
4710	1	Q9ULR3	Q9ULR3	Protein phosphatase 1H	PPM1H	Homo sapiens	56.448	6	6	269393	166208
4711	1	Q9UM22	Q9UM22	Mammalian ependymin-related protein 1	EPDR1	Homo sapiens	25.436	6	6	1576675	343152
4712	1	Q9UMX5	Q9UMX5	Neudesin	NENF	Homo sapiens	18.854	6	6	3564399	584178
4713	1	Q9UNS2	Q9UNS2	COP9 signalosome complex subunit 3	COPS3	Homo sapiens	47.874	6	6	2971707	1424996
4714	1	Q9UPN6	Q9UPN6	SR-related and CTD-associated factor 8	SCAF8	Homo sapiens	140.522	7	6	649327	51315
4715	1	Q9UPP1	Q9UPP1	Histone lysine demethylase PHF8	PHF8	Homo sapiens	117.866	6	6	342331	0

4716	1	Q9UPX8	Q9UPX8	SH3 and multiple ankyrin repeat domains protein 2	SHANK2	Homo sapiens	201.265	6	6	140958	118345
4717	1	Q9UQ13	Q9UQ13	Leucine-rich repeat protein SHOC-2	SHOC2	Homo sapiens	64.888	6	6	1356491	383173
4718	1	Q9Y279	Q9Y279	V-set and immunoglobulin domain-containing protein 4	VSIG4	Homo sapiens	43.987	6	6	730146	1172854
4719	1	Q9Y2A9	Q9Y2A9	N-acetyllactosaminide beta-1,3-N-acetylglucosaminyltransferase 3	B3GNT3	Homo sapiens	42.534	6	6	5732657	15357
4720	1	Q9Y2C3	Q9Y2C3	Beta-1,3-galactosyltransferase 5	B3GALT5	Homo sapiens	36.19	6	6	1951090	0
4721	1	Q9Y2G8	Q9Y2G8	DnaJ homolog subfamily C member 16	DNAJC16	Homo sapiens	90.592	6	6	496670	76926
4722	1	Q9Y2H2	Q9Y2H2	Phosphatidylinositide phosphatase SAC2	INPP5F	Homo sapiens	128.409	6	6	475552	0
4723	1	Q9Y2I1	Q9Y2I1	Nischarin	NISCH	Homo sapiens	166.631	6	6	513362	41536
4724	1	Q9Y2P4	Q9Y2P4	Long-chain fatty acid transport protein 6	SLC27A6	Homo sapiens	70.112	6	6	363054	0
4725	1	Q9Y2W6	Q9Y2W6	Tudor and KH domain-containing protein	TDRKH	Homo sapiens	62.046	6	6	460011	126652
4726	1	Q9Y312	Q9Y312	Protein AAR2 homolog	AAR2	Homo sapiens	43.471	6	6	332352	0
4727	1	Q9Y385	Q9Y385	Ubiquitin-conjugating enzyme E2 J1	UBE2J1	Homo sapiens	35.199	6	6	1731288	986908
4728	1	Q9Y3B3	Q9Y3B3	Transmembrane emp24 domain-containing protein 7	TMED7	Homo sapiens	25.171	6	6	5390993	4661918
4729	1	Q9Y3B7	Q9Y3B7	39S ribosomal protein L11, mitochondrial	MRPL11	Homo sapiens	20.682	6	6	1870934	447006
4730	1	Q9Y3B8	Q9Y3B8	Oligoribonuclease, mitochondrial	REXO2	Homo sapiens	26.833	6	6	5701900	1312336
4731	1	Q9Y3C8	Q9Y3C8	Ubiquitin-fold modifier-conjugating enzyme 1	UFC1	Homo sapiens	19.459	6	6	3437929	1841862
4732	1	Q9Y3I1	Q9Y3I1	F-box only protein 7	FBXO7	Homo sapiens	58.502	6	6	1015213	687289
4733	1	Q9Y3Q8	Q9Y3Q8	Sperm acrosome developmental regulator	SPACDR	Homo sapiens	41.025	6	6	1116149	822868
4734	1	Q9Y4C8	Q9Y4C8	Probable RNA-binding protein 19	RBM19	Homo sapiens	107.331	6	6	405425	18002
4735	1	Q9Y4R8	Q9Y4R8	Telomere length regulation protein TEL2 homolog	TELO2	Homo sapiens	91.748	6	6	305919	51917
4736	1	Q9Y530	Q9Y530	ADP-ribose glycohydrolase OARD1	OARD1	Homo sapiens	17.024	6	6	1036610	502048
4737	1	Q9Y5W9	Q9Y5W9	Sorting nexin-11	SNX11	Homo sapiens	30.433	6	6	472643	827610
4738	1	Q9Y6M5	Q9Y6M5	Proton-coupled zinc antiporter SLC30A1	SLC30A1	Homo sapiens	55.302	6	6	1581857	305587
4739	1	A2RUC4	A2RUC4	tRNA wybutosine-synthesizing protein 5	TYW5	Homo sapiens	36.549	5	5	423891	104444
4740	1	A4D126	A4D126	D-ribitol-5-phosphate cytidylyltransferase	CRPPA	Homo sapiens	49.874	5	5	167592	51519
4741	1	A4D2B0	A4D2B0	Metallo-beta-lactamase domain-containing protein 1	MBLAC1	Homo sapiens	27.199	5	5	537590	15724
4742	1	A4FU01	A4FU01	Myotubularin-related protein 11	MTMR11	Homo sapiens	79.548	5	5	404715	0
4743	1	A6ND36	A6ND36	Protein FAM83G	FAM83G	Homo sapiens	90.835	5	5	129167	27997
4744	1	A6NDG6	A6NDG6	Glycerol-3-phosphate phosphatase	PGP	Homo sapiens	34.003	5	5	1798891	1635629
4745	1	A6NHL2	A6NHL2	Tubulin alpha chain-like 3	TUBAL3	Homo sapiens	49.907	5	5	399865	0
4746	1	A8MVS5	A8MVS5	Protein HIDE1	HIDE1	Homo sapiens	24.754	5	5	0	1532546
4747	1	O00213	O00213	Amyloid beta precursor protein binding family B member 1	APBB1	Homo sapiens	77.246	5	5	353891	22262
4748	1	O00221	O00221	NF-kappa-B inhibitor epsilon	NFKBIE	Homo sapiens	52.862	5	5	350682	204496
4749	1	O00401	O00401	Actin nucleation-promoting factor WASL	WASL	Homo sapiens	54.828	5	5	1958644	27929
4750	1	O00522	O00522	Krev interaction trapped protein 1	KRIT1	Homo sapiens	84.348	5	5	321101	0
4751	1	O00629	O00629	Importin subunit alpha-3	KPNA4	Homo sapiens	57.887	9	5	1615829	582376
4752	1	O00743	O00743	Serine/threonine-protein phosphatase 6 catalytic subunit	PPP6C	Homo sapiens	35.144	5	5	1108039	697939
4753	1	O00746	O00746	Nucleoside diphosphate kinase, mitochondrial	NME4	Homo sapiens	20.657	5	5	628745	0
4754	1	O14498	O14498	Immunoglobulin superfamily containing leucine-rich repeat protein	ISLR	Homo sapiens	45.998	5	5	1190719	532233
4755	1	O14638	O14638	Ectonucleotide pyrophosphatase/phosphodiesterase family member 3	ENPP3	Homo sapiens	100.125	5	5	132130	0
4756	1	O14647	O14647	Chromodomain-helicase-DNA-binding protein 2	CHD2	Homo sapiens	211.348	9	5	495897	293120
4757	1	O14730	O14730	Serine/threonine-protein kinase RIO3	RIOK3	Homo sapiens	59.092	5	5	263800	522351
4758	1	O14735	O14735	CDP-diacylglycerol--inositol 3-phosphatidyltransferase	CDIPT	Homo sapiens	23.538	5	5	3080233	1224810
4759	1	O14737	O14737	Programmed cell death protein 5	PDCD5	Homo sapiens	14.285	5	5	9221339	2174616
4760	1	O14828	O14828	Secretory carrier-associated membrane protein 3	SCAMP3	Homo sapiens	38.286	5	5	1918416	948973
4761	1	O14949	O14949	Cytochrome b-c1 complex subunit 8	UQCRQ	Homo sapiens	9.904	5	5	11155283	2774159
4762	2	O14950;P19105	O14950	Myosin regulatory light chain 12B	MYL12B	Homo sapiens	19.779	5	5	27070447	34396042
4763	1	O14972	O14972	Vacuolar protein sorting-associated protein 26C	VPS26C	Homo sapiens	33.01	5	5	644811	338852

4764	1	O15021	O15021	Microtubule-associated serine/threonine-protein kinase 4	MAST4	Homo sapiens	284.102	5	5	230630	32223
4765	1	O15066	O15066	Kinesin-like protein KIF3B	KIF3B	Homo sapiens	85.126	8	5	276209	74169
4766	1	O15120	O15120	1-acyl-sn-glycerol-3-phosphate acyltransferase beta	AGPAT2	Homo sapiens	30.916	5	5	1599856	1653999
4767	1	O15127	O15127	Secretory carrier-associated membrane protein 2	SCAMP2	Homo sapiens	36.647	5	5	1231552	2178887
4768	1	O15160	O15160	DNA-directed RNA polymerases I and III subunit RPAC1	POLR1C	Homo sapiens	39.251	5	5	1081864	792163
4769	1	O15389	O15389	Sialic acid-binding Ig-like lectin 5	SIGLEC5	Homo sapiens	60.715	7	5	0	2933094
4770	1	O15533	O15533	Tapasin	TAPBP	Homo sapiens	47.568	5	5	4749927	6464961
4771	1	O43181	O43181	NADH dehydrogenase [ubiquinone] iron-sulfur protein 4, mitochondrial	NDUFS4	Homo sapiens	20.108	5	5	8755064	1167937
4772	1	O43272	O43272	Proline dehydrogenase 1, mitochondrial	PRODH	Homo sapiens	68.002	5	5	42647	362254
4773	1	O43447	O43447	Peptidyl-prolyl cis-trans isomerase H	PPIH	Homo sapiens	19.207	5	5	2927473	829806
4774	1	O43570	O43570	Carbonic anhydrase 12	CA12	Homo sapiens	39.451	5	5	5833298	435536
4775	1	O43752	O43752	Syntaxin-6	STX6	Homo sapiens	29.175	5	5	557639	453042
4776	1	O43772	O43772	Mitochondrial carnitine/acylcarnitine carrier protein	SLC25A20	Homo sapiens	32.944	5	5	3316166	678297
4777	1	O43818	O43818	U3 small nucleolar RNA-interacting protein 2	RRP9	Homo sapiens	51.841	5	5	485265	81207
4778	1	O43861	O43861	Probable phospholipid-transporting ATPase IIB	ATP9B	Homo sapiens	129.306	5	5	2272139	5630798
4779	1	O43895	O43895	Xaa-Pro aminopeptidase 2	XPNPEP2	Homo sapiens	75.625	5	5	740843	0
4780	1	O60447	O60447	Ecotropic viral integration site 5 protein homolog	EVI5	Homo sapiens	92.953	5	5	284626	0
4781	1	O60496	O60496	Docking protein 2	DOK2	Homo sapiens	45.38	5	5	591429	652727
4782	1	O60503	O60503	Adenylate cyclase type 9	ADCY9	Homo sapiens	150.701	5	5	209280	0
4783	1	O60551	O60551	Glycylpeptide N-tetradecanoyltransferase 2	NMT2	Homo sapiens	56.981	5	5	2616380	0
4784	1	O60583	O60583	Cyclin-T2	CCNT2	Homo sapiens	81.028	5	5	160137	109858
4785	1	O60641	O60641	Clathrin coat assembly protein AP180	SNAP91	Homo sapiens	92.503	5	5	321359	0
4786	1	O60884	O60884	DnaJ homolog subfamily A member 2	DNAJA2	Homo sapiens	45.748	5	5	3444944	2260551
4787	1	O75052	O75052	Carboxyl-terminal PDZ ligand of neuronal nitric oxide synthase protein	NOS1AP	Homo sapiens	56.151	5	5	458768	0
4788	1	O75064	O75064	DENN domain-containing protein 4B	DENND4B	Homo sapiens	163.846	5	5	61270	592886
4789	1	O75143	O75143	Autophagy-related protein 13	ATG13	Homo sapiens	56.572	5	5	343862	184985
4790	1	O75340	O75340	Programmed cell death protein 6	PDCD6	Homo sapiens	21.87	5	5	5544220	2115267
4791	1	O75420	O75420	GRB10-interacting GYF protein 1	GIGYF1	Homo sapiens	114.601	5	5	636443	46395
4792	1	O75449	O75449	Katanin p60 ATPase-containing subunit A1	KATNA1	Homo sapiens	55.967	6	5	317582	241762
4793	1	O75477	O75477	Erlin-1	ERLIN1	Homo sapiens	39.172	5	5	4680141	6549353
4794	1	O75503	O75503	Ceroid-lipofuscinosis neuronal protein 5	CLN5	Homo sapiens	41.496	5	5	587254	400615
4795	1	O75616	O75616	GTPase Era, mitochondrial	ERAL1	Homo sapiens	48.352	5	5	580533	0
4796	1	O75717	O75717	WD repeat and HMG-box DNA-binding protein 1	WDHD1	Homo sapiens	125.971	5	5	154874	67886
4797	1	O75815	O75815	Breast cancer anti-estrogen resistance protein 3	BCAR3	Homo sapiens	92.566	5	5	263143	22559
4798	1	O75935	O75935	Dynactin subunit 3	DCTN3	Homo sapiens	21.119	5	5	2824726	1176335
4799	1	O94766	O94766	Galactosylgalactosylxylosylprotein 3-beta-glucuronosyltransferase 3	B3GAT3	Homo sapiens	37.122	5	5	629620	736704
4800	1	O94868	O94868	F-BAR and double SH3 domains protein 2	FCHSD2	Homo sapiens	84.277	5	5	435329	114782
4801	1	O94927	O94927	HAUS augmin-like complex subunit 5	HAUS5	Homo sapiens	71.685	5	5	293934	181790
4802	1	O94964	O94964	Protein SOGA1	SOGA1	Homo sapiens	159.761	5	5	86974	156191
4803	1	O95081	O95081	Arf-GAP domain and FG repeat-containing protein 2	AGFG2	Homo sapiens	48.965	5	5	448671	29848
4804	1	O95168	O95168	NADH dehydrogenase [ubiquinone] 1 beta subcomplex subunit 4	NDUFB4	Homo sapiens	15.207	5	5	13134168	3145963
4805	1	O95208	O95208	Epsin-2	EPN2	Homo sapiens	68.48	6	5	25562702	0
4806	1	O95232	O95232	Luc7-like protein 3	LUC7L3	Homo sapiens	51.466	5	5	1692798	874338
4807	1	O95236	O95236	Apolipoprotein L3	APOL3	Homo sapiens	44.278	5	5	1451202	800950
4808	1	O95319	O95319	CUGBP Elav-like family member 2	CELF2	Homo sapiens	54.285	6	5	368247	691148
4809	1	O95361	O95361	Tripartite motif-containing protein 16	TRIM16	Homo sapiens	63.957	10	5	112451	216534
4810	1	O95382	O95382	Mitogen-activated protein kinase kinase kinase 6	MAP3K6	Homo sapiens	142.597	5	5	284282	0
4811	1	O95436	O95436	Sodium-dependent phosphate transport protein 2B	SLC34A2	Homo sapiens	75.76	5	5	0	4617041

4812	1	O95551	O95551	Tyrosyl-DNA phosphodiesterase 2	TDP2	Homo sapiens	40.929	5	5	18759342	207470
4813	1	O95684	O95684	Centrosomal protein 43	CEP43	Homo sapiens	43.063	5	5	250810	268008
4814	1	O95714	O95714	E3 ubiquitin-protein ligase HERC2	HERC2	Homo sapiens	527.236	6	5	185660	22680
4815	1	O95749	O95749	Geranylgeranyl pyrophosphate synthase	GGPS1	Homo sapiens	34.869	5	5	835107	519892
4816	1	O95801	O95801	Tetratricopeptide repeat protein 4	TTC4	Homo sapiens	44.681	5	5	286653	219108
4817	1	O95858	O95858	Tetraspanin-15	TSPAN15	Homo sapiens	33.163	5	5	696088	33145
4818	1	O95900	O95900	Pseudouridylate synthase TRUB2, mitochondrial	TRUB2	Homo sapiens	36.695	5	5	180822	0
4819	1	O95989	O95989	Diphosphoinositol polyphosphate phosphohydrolase 1	NUDT3	Homo sapiens	19.471	5	5	2149850	671401
4820	1	O96006	O96006	E3 SUMO-protein ligase ZBED1	ZBED1	Homo sapiens	78.159	5	5	213786	60892
4821	1	P00480	P00480	Ornithine transcarbamylase, mitochondrial	OTC	Homo sapiens	39.936	5	5	2108145	124483
4822	1	P01034	P01034	Cystatin-C	CST3	Homo sapiens	15.799	5	5	24615693	5271742
4823	1	P01859	P01859	Immunoglobulin heavy constant gamma 2	IGHG2	Homo sapiens	35.902	5	5	130304204	150476754
4824	1	P01861	P01861	Immunoglobulin heavy constant gamma 4	IGHG4	Homo sapiens	35.941	7	5	11940780	19520826
4825	1	P02750	P02750	Leucine-rich alpha-2-glycoprotein	LRG1	Homo sapiens	38.179	5	5	6998302	6151251
4826	1	P02775	P02775	Platelet basic protein	PPBP	Homo sapiens	13.893	5	5	3417959	6063033
4827	1	P02792	P02792	Ferritin light chain	FTL	Homo sapiens	20.018	5	5	4010570	58253818
4828	1	P03915	P03915	NADH-ubiquinone oxidoreductase chain 5	MT-ND5	Homo sapiens	67.03	5	5	2838371	182427
4829	1	P04075	P04075	Fructose-bisphosphate aldolase A	ALDOA	Homo sapiens	39.418	21	5	120038990	71925167
4830	1	P04233	P04233	HLA class II histocompatibility antigen gamma chain	CD74	Homo sapiens	33.516	5	5	4483511	1985415
4831	1	P05387	P05387	60S acidic ribosomal protein P2	RPLP2	Homo sapiens	11.663	5	5	30186711	10703926
4832	1	P05413	P05413	Fatty acid-binding protein, heart	FABP3	Homo sapiens	14.855	5	5	2879953	140212
4833	1	P05452	P05452	Tetranectin	CLEC3B	Homo sapiens	22.537	8	5	2764936	1745084
4834	1	P06454	P06454	Prothymosin alpha	PTMA	Homo sapiens	12.201	5	5	31948755	10409763
4835	1	P06730	P06730	Eukaryotic translation initiation factor 4E	EIF4E	Homo sapiens	25.098	5	5	7179692	4896480
4836	1	P06870	P06870	Kallikrein-1	KLK1	Homo sapiens	28.89	5	5	3381339	35091
4837	1	P07101	P07101	Tyrosine 3-monooxygenase	TH	Homo sapiens	58.602	5	5	181042	0
4838	1	P07148	P07148	Fatty acid-binding protein, liver	FABP1	Homo sapiens	14.207	5	5	541794449	13186382
4839	1	P07359	P07359	Platelet glycoprotein Ib alpha chain	GP1BA	Homo sapiens	71.539	5	5	149921	415510
4840	1	P07741	P07741	Adenine phosphoribosyltransferase	APRT	Homo sapiens	19.606	5	5	5246575	3678618
4841	1	P08185	P08185	Corticosteroid-binding globulin	SERPINA6	Homo sapiens	45.14	5	5	4375521	4377040
4842	1	P09001	P09001	39S ribosomal protein L3, mitochondrial	MRPL3	Homo sapiens	38.633	5	5	1320340	326970
4843	1	P09668	P09668	Pro-cathepsin H	CTSH	Homo sapiens	37.392	5	5	2536006	814266
4844	1	P0DTL6	P0DTL6	Zinc finger TRAF-type-containing protein 1	ZFTRAF1	Homo sapiens	43.813	5	5	271718	174048
4845	1	P10588	P10588	Nuclear receptor subfamily 2 group F member 6	NR2F6	Homo sapiens	42.978	5	5	2504994	88316
4846	1	P11166	P11166	Solute carrier family 2, facilitated glucose transporter member 1	SLC2A1	Homo sapiens	54.085	5	5	2389957	4547630
4847	1	P11226	P11226	Mannose-binding protein C	MBL2	Homo sapiens	26.142	5	5	546139	1415602
4848	1	P11233	P11233	Ras-related protein Ral-A	RALA	Homo sapiens	23.565	6	5	9197037	9979894
4849	1	P11362	P11362	Fibroblast growth factor receptor 1	FGFR1	Homo sapiens	91.868	7	5	276973	13075
4850	1	P11940	P11940	Polyadenylate-binding protein 1	PABPC1	Homo sapiens	70.67	22	5	6239129	5179530
4851	1	P13727	P13727	Bone marrow proteoglycan	PRG2	Homo sapiens	25.205	5	5	1592255	173104
4852	1	P14770	P14770	Platelet glycoprotein IX	GP9	Homo sapiens	19.046	5	5	0	447875
4853	1	P14927	P14927	Cytochrome b-c1 complex subunit 7	UQCRB	Homo sapiens	13.53	5	5	12335585	4276502
4854	1	P15153	P15153	Ras-related C3 botulinum toxin substrate 2	RAC2	Homo sapiens	21.427	5	5	6439761	120339824
4855	1	P15309	P15309	Prostatic acid phosphatase	ACP3	Homo sapiens	44.564	5	5	466861	553663
4856	1	P16050	P16050	Polyunsaturated fatty acid lipoxygenase ALOX15	ALOX15	Homo sapiens	74.805	5	5	252338	23900
4857	1	P16455	P16455	Methylated-DNA--protein-cysteine methyltransferase	MGMT	Homo sapiens	21.647	5	5	922998	0
4858	1	P16949	P16949	Stathmin	STMN1	Homo sapiens	17.305	6	5	19034499	12094573
4859	1	P17706	P17706	Tyrosine-protein phosphatase non-receptor type 2	PTPN2	Homo sapiens	48.474	5	5	380979	317937

4860	1	P17948	P17948	Vascular endothelial growth factor receptor 1	FLT1	Homo sapiens	150.772	5	5	233225	228781
4861	1	P18077	P18077	60S ribosomal protein L35a	RPL35A	Homo sapiens	12.536	5	5	5329372	855927
4862	1	P18510	P18510	Interleukin-1 receptor antagonist protein	IL1RN	Homo sapiens	20.056	5	5	143765	1687365
4863	1	P18669	P18669	Phosphoglycerate mutase 1	PGAM1	Homo sapiens	28.803	12	5	27831840	19738770
4864	1	P19387	P19387	DNA-directed RNA polymerase II subunit RPB3	POLR2C	Homo sapiens	31.442	5	5	1163488	797243
4865	1	P20333	P20333	Tumor necrosis factor receptor superfamily member 1B	TNFRSF1B	Homo sapiens	48.292	5	5	27061	776636
4866	1	P20339	P20339	Ras-related protein Rab-5A	RAB5A	Homo sapiens	23.659	5	5	4076153	1971793
4867	1	P21860	P21860	Receptor tyrosine-protein kinase erbB-3	ERBB3	Homo sapiens	148.099	5	5	527147	0
4868	1	P22694	P22694	cAMP-dependent protein kinase catalytic subunit beta	PRKACB	Homo sapiens	40.622	5	5	3756645	227230
4869	1	P24666	P24666	Low molecular weight phosphotyrosine protein phosphatase	ACP1	Homo sapiens	18.042	5	5	4657536	2809260
4870	1	P25189	P25189	Myelin protein P0	MPZ	Homo sapiens	27.553	5	5	2613132	0
4871	1	P25789	P25789	Proteasome subunit alpha type-4	PSMA4	Homo sapiens	29.483	5	5	12086595	7655808
4872	1	P27169	P27169	Serum paraoxonase/arylesterase 1	PON1	Homo sapiens	39.731	5	5	3475768	8902613
4873	1	P27701	P27701	CD82 antigen	CD82	Homo sapiens	29.626	5	5	905693	2402424
4874	1	P27707	P27707	Deoxycytidine kinase	DCK	Homo sapiens	30.519	5	5	1191749	1819147
4875	1	P29279	P29279	CCN family member 2	CCN2	Homo sapiens	38.091	5	5	359431	582084
4876	1	P29317	P29317	Ephrin type-A receptor 2	EPHA2	Homo sapiens	108.268	5	5	186976	367988
4877	1	P29353	P29353	SHC-transforming protein 1	SHC1	Homo sapiens	62.821	6	5	2843681	503306
4878	1	P30050	P30050	60S ribosomal protein L12	RPL12	Homo sapiens	17.818	5	5	22375048	15680531
4879	1	P30405	P30405	Peptidyl-prolyl cis-trans isomerase F, mitochondrial	PPIF	Homo sapiens	22.04	5	5	5164317	2621771
4880	1	P30414	P30414	NK-tumor recognition protein	NKTR	Homo sapiens	165.678	5	5	179698	0
4881	1	P31321	P31321	cAMP-dependent protein kinase type I-beta regulatory subunit	PRKAR1B	Homo sapiens	43.071	5	5	411688	34900
4882	1	P33240	P33240	Cleavage stimulation factor subunit 2	CSTF2	Homo sapiens	60.96	9	5	2633160	375459
4883	1	P36959	P36959	GMP reductase 1	GMPR	Homo sapiens	37.419	5	5	276125	362589
4884	1	P37023	P37023	Serine/threonine-protein kinase receptor R3	ACVRL1	Homo sapiens	56.124	5	5	267295	0
4885	1	P37059	P37059	17-beta-hydroxysteroid dehydrogenase type 2	HSD17B2	Homo sapiens	42.786	5	5	1586798	24088
4886	1	P37235	P37235	Hippocalcin-like protein 1	HPCAL1	Homo sapiens	22.312	8	5	976807	385583
4887	1	P40189	P40189	Interleukin-6 receptor subunit beta	IL6ST	Homo sapiens	103.538	5	5	461634	87180
4888	1	P40306	P40306	Proteasome subunit beta type-10	PSMB10	Homo sapiens	28.935	5	5	3060037	2225614
4889	1	P40692	P40692	DNA mismatch repair protein Mlh1	MLH1	Homo sapiens	84.603	5	5	279516	57086
4890	1	P42126	P42126	Enoyl-CoA delta isomerase 1, mitochondrial	ECI1	Homo sapiens	32.814	5	5	12771840	2138811
4891	1	P42330	P42330	Aldo-keto reductase family 1 member C3	AKR1C3	Homo sapiens	36.854	11	5	1533023	1438242
4892	1	P42684	P42684	Tyrosine-protein kinase ABL2	ABL2	Homo sapiens	128.346	5	5	245078	17257
4893	1	P43007	P43007	Neutral amino acid transporter A	SLC1A4	Homo sapiens	55.721	5	5	764633	72420
4894	1	P43487	P43487	Ran-specific GTPase-activating protein	RANBP1	Homo sapiens	23.311	5	5	9915350	7882762
4895	1	P45984	P45984	Mitogen-activated protein kinase 9	MAPK9	Homo sapiens	48.139	10	5	610393	148825
4896	1	P46531	P46531	Neurogenic locus notch homolog protein 1	NOTCH1	Homo sapiens	272.512	6	5	162766	86886
4897	1	P46734	P46734	Dual specificity mitogen-activated protein kinase kinase 3	MAP2K3	Homo sapiens	39.319	5	5	385820	1049239
4898	1	P46782	P46782	40S ribosomal protein S5	RPS5	Homo sapiens	22.878	5	5	3326491	817700
4899	1	P48059	P48059	LIM and senescent cell antigen-like-containing domain protein 1	LIMS1	Homo sapiens	37.251	9	5	9102817	5464173
4900	1	P48454	P48454	Serine/threonine-protein phosphatase 2B catalytic subunit gamma isoform	PPP3CC	Homo sapiens	58.129	5	5	970810	501623
4901	1	P48556	P48556	26S proteasome non-ATPase regulatory subunit 8	PSMD8	Homo sapiens	39.613	5	5	2329637	1771513
4902	1	P49247	P49247	Ribose-5-phosphate isomerase	RPIA	Homo sapiens	33.273	5	5	773179	620908
4903	1	P49406	P49406	39S ribosomal protein L19, mitochondrial	MRPL19	Homo sapiens	33.536	5	5	1310920	188846
4904	1	P49903	P49903	Selenide, water dikinase 1	SEPHS1	Homo sapiens	42.912	5	5	3102358	985717
4905	1	P49914	P49914	5-formyltetrahydrofolate cyclo-ligase	MTHFS	Homo sapiens	23.255	5	5	679206	753585
4906	1	P50591	P50591	Tumor necrosis factor ligand superfamily member 10	TNFSF10	Homo sapiens	32.511	5	5	78179	547387

4907	1	P50613	P50613	Cyclin-dependent kinase 7	CDK7	Homo sapiens	39.039	5	5	282988	14661
4908	1	P51575	P51575	P2X purinoceptor 1	P2RX1	Homo sapiens	44.983	5	5	237363	564450
4909	1	P51606	P51606	N-acylglucosamine 2-epimerase	RENBP	Homo sapiens	47.748	5	5	917890	1008820
4910	1	P51636	P51636	Caveolin-2	CAV2	Homo sapiens	18.292	5	5	1283278	49967
4911	1	P51809	P51809	Vesicle-associated membrane protein 7	VAMP7	Homo sapiens	24.934	5	5	571395	1541819
4912	1	P52434	P52434	DNA-directed RNA polymerases I, II, and III subunit RPABC3	POLR2H	Homo sapiens	17.142	5	5	1744323	682510
4913	1	P52756	P52756	RNA-binding protein 5	RBM5	Homo sapiens	92.155	5	5	757098	133005
4914	1	P52815	P52815	39S ribosomal protein L12, mitochondrial	MRPL12	Homo sapiens	21.348	5	5	3976996	464923
4915	1	P53370	P53370	Nucleoside diphosphate-linked moiety X motif 6	NUDT6	Homo sapiens	35.68	5	5	296141	0
4916	1	P53634	P53634	Dipeptidyl peptidase 1	CTSC	Homo sapiens	51.853	5	5	2774338	2432347
4917	1	P53667	P53667	LIM domain kinase 1	LIMK1	Homo sapiens	72.586	5	5	255232	188907
4918	1	P54198	P54198	Protein HIRA	HIRA	Homo sapiens	111.837	5	5	282792	166062
4919	1	P54760	P54760	Ephrin type-B receptor 4	EPHB4	Homo sapiens	108.274	6	5	489063	0
4920	1	P54803	P54803	Galactocerebrosidase	GALC	Homo sapiens	77.063	5	5	776432	435172
4921	1	P55001	P55001	Microfibrillar-associated protein 2	MFAP2	Homo sapiens	20.825	5	5	1103837	0
4922	1	P55145	P55145	Mesencephalic astrocyte-derived neurotrophic factor	MANF	Homo sapiens	20.699	5	5	3844262	2855410
4923	1	P55198	P55198	Protein AF-17	MLLT6	Homo sapiens	112.05	5	5	223989	0
4924	1	P55327	P55327	Tumor protein D52	TPD52	Homo sapiens	24.326	5	5	19435038	3669518
4925	1	P55789	P55789	FAD-linked sulfhydryl oxidase ALR	GFER	Homo sapiens	23.451	5	5	805037	0
4926	1	P56182	P56182	Ribosomal RNA processing protein 1 homolog A	RRP1	Homo sapiens	52.841	5	5	662765	382933
4927	1	P56385	P56385	ATP synthase subunit e, mitochondrial	ATP5ME	Homo sapiens	7.931	5	5	28928283	6153809
4928	1	P56937	P56937	3-keto-steroid reductase/17-beta-hydroxysteroid dehydrogenase 7	HSD17B7	Homo sapiens	38.205	5	5	283947	26852
4929	1	P56962	P56962	Syntaxin-17	STX17	Homo sapiens	33.401	5	5	992018	410412
4930	1	P57105	P57105	Synaptojanin-2-binding protein	SYNJ2BP	Homo sapiens	15.928	5	5	4295949	1691908
4931	1	P58335	P58335	Anthrax toxin receptor 2	ANTXR2	Homo sapiens	53.663	7	5	2841993	770789
4932	1	P60201	P60201	Myelin proteolipid protein	PLP1	Homo sapiens	30.076	5	5	1963388	0
4933	1	P60484	P60484	Phosphatidylinositol 3,4,5-trisphosphate 3-phosphatase and dual-specificity protein phosphatase PTEN	PTEN	Homo sapiens	47.166	5	5	354671	644197
4934	1	P60880	P60880	Synaptosomal-associated protein 25	SNAP25	Homo sapiens	23.316	5	5	890125	0
4935	1	P60903	P60903	Protein S100-A10	S100A10	Homo sapiens	11.2	5	5	129777161	20637737
4936	1	P60983	P60983	Glia maturation factor beta	GMFB	Homo sapiens	16.711	5	5	2572818	1399865
4937	1	P61019	P61019	Ras-related protein Rab-2A	RAB2A	Homo sapiens	23.544	13	5	8603888	4084436
4938	1	P61026	P61026	Ras-related protein Rab-10	RAB10	Homo sapiens	22.542	5	5	3624345	2976182
4939	1	P61244	P61244	Protein max	MAX	Homo sapiens	18.277	5	5	585449	332058
4940	1	P61266	P61266	Syntaxin-1B	STX1B	Homo sapiens	33.245	5	5	485431	72836
4941	1	P61313	P61313	60S ribosomal protein L15	RPL15	Homo sapiens	24.145	5	5	20358579	8528108
4942	1	P61353	P61353	60S ribosomal protein L27	RPL27	Homo sapiens	15.796	5	5	23517019	6600088
4943	1	P61599	P61599	N-alpha-acetyltransferase 20	NAA20	Homo sapiens	20.368	5	5	209246	0
4944	1	P61604	P61604	10 kDa heat shock protein, mitochondrial	HSPE1	Homo sapiens	10.931	5	5	67530015	24716212
4945	1	P61769	P61769	Beta-2-microglobulin	B2M	Homo sapiens	13.713	5	5	7538673	7729622
4946	1	P61923	P61923	Coatomer subunit zeta-1	COPZ1	Homo sapiens	20.196	5	5	5462372	4623287
4947	1	P62166	P62166	Neuronal calcium sensor 1	NCS1	Homo sapiens	21.877	5	5	2865801	135835
4948	1	P62318	P62318	Small nuclear ribonucleoprotein Sm D3	SNRPD3	Homo sapiens	13.915	5	5	7860014	5212306
4949	1	P62701	P62701	40S ribosomal protein S4, X isoform	RPS4X	Homo sapiens	29.596	13	5	3755968	1415848
4950	1	P62750	P62750	60S ribosomal protein L23a	RPL23A	Homo sapiens	17.695	5	5	14864654	5208562
4951	1	P62760	P62760	Visinin-like protein 1	VSNL1	Homo sapiens	22.144	5	5	391793	141448
4952	1	P62829	P62829	60S ribosomal protein L23	RPL23	Homo sapiens	14.865	5	5	12807383	2991453
4953	1	P62910	P62910	60S ribosomal protein L32	RPL32	Homo sapiens	15.859	5	5	5934702	2805369

4954	1	P62917	P62917	60S ribosomal protein L8	RPL8	Homo sapiens	28.026	5	5	11775343	2575785
4955	1	P63279	P63279	SUMO-conjugating enzyme UBC9	UBE2I	Homo sapiens	18.008	5	5	15048286	13880585
4956	1	P67809	P67809	Y-box-binding protein 1	YBX1	Homo sapiens	35.921	7	5	14271320	3657231
4957	1	P78330	P78330	Phosphoserine phosphatase	PSPH	Homo sapiens	25.009	5	5	718333	766926
4958	1	P78537	P78537	Biogenesis of lysosome-related organelles complex 1 subunit 1	BLOC1S1	Homo sapiens	17.262	5	5	899905	645078
4959	1	P78563	P78563	Double-stranded RNA-specific editase 1	ADARB1	Homo sapiens	80.763	5	5	387990	0
4960	1	P82673	P82673	28S ribosomal protein S35, mitochondrial	MRPS35	Homo sapiens	36.844	5	5	661183	182520
4961	1	P82970	P82970	High mobility group nucleosome-binding domain-containing protein 5	HMGN5	Homo sapiens	31.525	5	5	75762988	404106
4962	1	P83110	P83110	Serine protease HTRA3	HTRA3	Homo sapiens	48.606	5	5	184885	292803
4963	1	P83876	P83876	Thioredoxin-like protein 4A	TXNL4A	Homo sapiens	16.786	5	5	1480596	525515
4964	1	P83916	P83916	Chromobox protein homolog 1	CBX1	Homo sapiens	21.42	5	5	3650954	1277141
4965	1	P98172	P98172	Ephrin-B1	EFNB1	Homo sapiens	38.006	5	5	1406984	121011
4966	1	P98179	P98179	RNA-binding protein 3	RBM3	Homo sapiens	17.171	5	5	6927822	688636
4967	1	Q00535	Q00535	Cyclin-dependent kinase 5	CDK5	Homo sapiens	33.303	5	5	979093	1022329
4968	1	Q01459	Q01459	Di-N-acetylchitobiase	CTBS	Homo sapiens	43.76	5	5	754627	1479140
4969	1	Q03426	Q03426	Mevalonate kinase	MVK	Homo sapiens	42.451	5	5	1080862	593019
4970	1	Q04323	Q04323	UBX domain-containing protein 1	UBXN1	Homo sapiens	33.325	5	5	23400264	459623
4971	1	Q04760	Q04760	Lactoylglutathione lyase	GLO1	Homo sapiens	20.778	5	5	19288642	5417325
4972	1	Q04837	Q04837	Single-stranded DNA-binding protein, mitochondrial	SSBP1	Homo sapiens	17.257	5	5	5793871	2091619
4973	1	Q05048	Q05048	Cleavage stimulation factor subunit 1	CSTF1	Homo sapiens	48.357	5	5	1055200	579456
4974	1	Q06136	Q06136	3-ketodihydrosphingosine reductase	KDSR	Homo sapiens	36.185	5	5	1709274	428765
4975	1	Q06190	Q06190	Serine/threonine-protein phosphatase 2A regulatory subunit B'' subunit alpha	PPP2R3A	Homo sapiens	130.278	5	5	917867	0
4976	1	Q07021	Q07021	Complement component 1 Q subcomponent-binding protein, mitochondrial	C1QBP	Homo sapiens	31.362	5	5	17104195	6263691
4977	1	Q08397	Q08397	Lysyl oxidase homolog 1	LOXL1	Homo sapiens	63.109	5	5	665153	0
4978	1	Q08623	Q08623	Pseudouridine-5'-phosphatase	PUDP	Homo sapiens	25.248	5	5	4395861	576339
4979	1	Q11201	Q11201	CMP-N-acetylneuraminate-beta-galactosamide-alpha-2,3-sialyltransferase 1	ST3GAL1	Homo sapiens	39.077	6	5	0	1253792
4980	1	Q12851	Q12851	Mitogen-activated protein kinase kinase kinase kinase 2	MAP4K2	Homo sapiens	91.559	5	5	317756	288678
4981	1	Q12986	Q12986	Transcriptional repressor NF-X1	NFX1	Homo sapiens	124.396	5	5	138429	18049
4982	1	Q13191	Q13191	E3 ubiquitin-protein ligase CBL-B	CBLB	Homo sapiens	109.452	5	5	725651	589389
4983	1	Q13214	Q13214	Semaphorin-3B	SEMA3B	Homo sapiens	83.122	5	5	447946	117772
4984	1	Q13361	Q13361	Microfibrillar-associated protein 5	MFAP5	Homo sapiens	19.608	5	5	4289179	48376
4985	1	Q13422	Q13422	DNA-binding protein Ikaros	IKZF1	Homo sapiens	57.531	5	5	236670	960209
4986	1	Q13424	Q13424	Alpha-1-syntrophin	SNTA1	Homo sapiens	53.896	5	5	688700	0
4987	1	Q13445	Q13445	Transmembrane emp24 domain-containing protein 1	TMED1	Homo sapiens	25.204	5	5	658844	1000138
4988	1	Q13469	Q13469	Nuclear factor of activated T-cells, cytoplasmic 2	NFATC2	Homo sapiens	100.145	5	5	540896	90976
4989	1	Q13509	Q13509	Tubulin beta-3 chain	TUBB3	Homo sapiens	50.432	6	5	2796720	281131
4990	1	Q13568	Q13568	Interferon regulatory factor 5	IRF5	Homo sapiens	56.044	5	5	50218	1244806
4991	1	Q13685	Q13685	Angio-associated migratory cell protein	AAMP	Homo sapiens	46.75	5	5	1460602	315690
4992	1	Q13823	Q13823	Nucleolar GTP-binding protein 2	GNL2	Homo sapiens	83.657	5	5	529909	134277
4993	1	Q13882	Q13882	Protein-tyrosine kinase 6	PTK6	Homo sapiens	51.834	5	5	1152955	27316
4994	1	Q13907	Q13907	Isopentenyl-diphosphate Delta-isomerase 1	IDI1	Homo sapiens	26.319	6	5	1270337	528915
4995	1	Q13952	Q13952	Nuclear transcription factor Y subunit gamma	NFYC	Homo sapiens	50.303	5	5	373537	110252
4996	1	Q14116	Q14116	Interleukin-18	IL18	Homo sapiens	22.328	5	5	2906182	6893762
4997	1	Q14164	Q14164	Inhibitor of nuclear factor kappa-B kinase subunit epsilon	IKBKE	Homo sapiens	80.464	5	5	69566	422292
4998	1	Q14181	Q14181	DNA polymerase alpha subunit B	POLA2	Homo sapiens	65.947	5	5	1475091	0

4999	1	Q14197	Q14197	Peptidyl-tRNA hydrolase ICT1, mitochondrial	MRPL58	Homo sapiens	23.629	5	5	2392731	422633
5000	1	Q14241	Q14241	Elongin-A	ELOA	Homo sapiens	89.91	5	5	715974	79391
5001	1	Q14320	Q14320	Protein FAM50A	FAM50A	Homo sapiens	40.243	6	5	1791549	1018583
5002	1	Q14331	Q14331	Protein FRG1	FRG1	Homo sapiens	29.172	5	5	1197999	533117
5003	1	Q14393	Q14393	Growth arrest-specific protein 6	GAS6	Homo sapiens	74.927	5	5	302201	259940
5004	1	Q14449	Q14449	Growth factor receptor-bound protein 14	GRB14	Homo sapiens	60.99	5	5	874656	0
5005	1	Q147X3	Q147X3	N-alpha-acetyltransferase 30	NAA30	Homo sapiens	39.319	5	5	670365	72474
5006	1	Q15050	Q15050	Ribosome biogenesis regulatory protein homolog	RRS1	Homo sapiens	41.193	5	5	1223306	357623
5007	1	Q15165	Q15165	Serum paraoxonase/arylesterase 2	PON2	Homo sapiens	39.382	5	5	1960586	550259
5008	1	Q15166	Q15166	Serum paraoxonase/lactonase 3	PON3	Homo sapiens	39.608	5	5	1014963	3431919
5009	1	Q15185	Q15185	Prostaglandin E synthase 3	PTGES3	Homo sapiens	18.696	5	5	7373862	6728701
5010	1	Q15274	Q15274	Nicotinate-nucleotide pyrophosphorylase [carboxylating]	QPRT	Homo sapiens	30.845	5	5	1510550	1874662
5011	1	Q15286	Q15286	Ras-related protein Rab-35	RAB35	Homo sapiens	23.024	5	5	2479914	925468
5012	1	Q15306	Q15306	Interferon regulatory factor 4	IRF4	Homo sapiens	51.775	5	5	269930	0
5013	1	Q15366	Q15366	Poly(rC)-binding protein 2	PCBP2	Homo sapiens	38.579	6	5	18594851	9509446
5014	1	Q15382	Q15382	GTP-binding protein Rheb	RHEB	Homo sapiens	20.497	5	5	1605858	483469
5015	1	Q15526	Q15526	Surfeit locus protein 1	SURF1	Homo sapiens	33.33	5	5	914474	370272
5016	1	Q15629	Q15629	Translocating chain-associated membrane protein 1	TRAM1	Homo sapiens	43.072	5	5	1334144	567163
5017	1	Q15633	Q15633	RISC-loading complex subunit TARBP2	TARBP2	Homo sapiens	39.039	5	5	381088	337204
5018	1	Q15650	Q15650	Activating signal cointegrator 1	TRIP4	Homo sapiens	66.147	5	5	787735	315757
5019	1	Q15653	Q15653	NF-kappa-B inhibitor beta	NFKBIB	Homo sapiens	37.772	5	5	810279	283260
5020	1	Q15678	Q15678	Tyrosine-protein phosphatase non-receptor type 14	PTPN14	Homo sapiens	135.26	6	5	561609	17908
5021	1	Q15742	Q15742	NGFI-A-binding protein 2	NAB2	Homo sapiens	56.596	6	5	249186	162424
5022	1	Q15814	Q15814	Tubulin-specific chaperone C	TBCC	Homo sapiens	39.248	5	5	495869	202725
5023	1	Q16082	Q16082	Heat shock protein beta-2	HSPB2	Homo sapiens	20.231	5	5	853032	0
5024	1	Q16566	Q16566	Calcium/calmodulin-dependent protein kinase type IV	CAMK4	Homo sapiens	51.926	5	5	254933	0
5025	1	Q16595	Q16595	Frataxin, mitochondrial	FXN	Homo sapiens	23.132	5	5	1753616	884614
5026	1	Q16644	Q16644	MAP kinase-activated protein kinase 3	MAPKAPK3	Homo sapiens	42.988	5	5	266002	953567
5027	1	Q16718	Q16718	NADH dehydrogenase [ubiquinone] 1 alpha subcomplex subunit 5	NDUFA5	Homo sapiens	13.459	5	5	4242080	951930
5028	1	Q16880	Q16880	2-hydroxyacylsphingosine 1-beta-galactosyltransferase	UGT8	Homo sapiens	61.44	5	5	555855	0
5029	1	Q2TAA2	Q2TAA2	Isoamyl acetate-hydrolyzing esterase 1 homolog	IAH1	Homo sapiens	27.6	5	5	2089083	573964
5030	1	Q32P41	Q32P41	tRNA (guanine(37)-N1)-methyltransferase	TRMT5	Homo sapiens	58.246	5	5	178041	31958
5031	1	Q3KQZ1	Q3KQZ1	Solute carrier family 25 member 35	SLC25A35	Homo sapiens	32.437	5	5	592408	120877
5032	1	Q3SXM5	Q3SXM5	Inactive hydroxysteroid dehydrogenase-like protein 1	HSDL1	Homo sapiens	37	5	5	1124775	235184
5033	1	Q3ZCW2	Q3ZCW2	Galectin-related protein	LGALSL	Homo sapiens	18.987	5	5	1248800	342148
5034	1	Q4LE39	Q4LE39	AT-rich interactive domain-containing protein 4B	ARID4B	Homo sapiens	147.812	5	5	270966	119504
5035	1	Q52LW3	Q52LW3	Rho GTPase-activating protein 29	ARHGAP29	Homo sapiens	142.068	5	5	237990	323342
5036	1	Q53R41	Q53R41	FAST kinase domain-containing protein 1, mitochondrial	FASTKD1	Homo sapiens	97.412	5	5	354031	0
5037	1	Q562R1	Q562R1	Beta-actin-like protein 2	ACTBL2	Homo sapiens	42.003	5	5	8644163	8433104
5038	1	Q58A45	Q58A45	PAN2-PAN3 deadenylation complex subunit PAN3	PAN3	Homo sapiens	95.616	5	5	186663	63093
5039	1	Q5H8C1	Q5H8C1	FRAS1-related extracellular matrix protein 1	FREM1	Homo sapiens	244.161	5	5	129465	0
5040	1	Q5HYJ3	Q5HYJ3	Protein FAM76B	FAM76B	Homo sapiens	38.707	5	5	333875	277729
5041	1	Q5RKV6	Q5RKV6	Exosome complex component MTR3	EXOSC6	Homo sapiens	28.235	5	5	1573316	565939
5042	1	Q5SZK8	Q5SZK8	FRAS1-related extracellular matrix protein 2	FREM2	Homo sapiens	351.172	5	5	123279	125748
5043	1	Q5TAX3	Q5TAX3	Terminal uridylyltransferase 4	TUT4	Homo sapiens	185.171	5	5	198339	10336
5044	1	Q5VTQ0	Q5VTQ0	Tetratricopeptide repeat protein 39B	TTC39B	Homo sapiens	76.959	5	5	358996	0
5045	1	Q5VW38	Q5VW38	Protein GPR107	GPR107	Homo sapiens	66.99	5	5	1011902	819239
5046	1	Q5XUX1	Q5XUX1	F-box/WD repeat-containing protein 9	FBXW9	Homo sapiens	50.76	5	5	541090	133115

5047	1 Q641Q3	Q641Q3	Meteorin-like protein	METRNL	Homo sapiens	34.397	5	5	53088	844561
5048	1 Q658P3	Q658P3	Metalloreductase STEAP3	STEAP3	Homo sapiens	54.601	5	5	795433	503128
5049	1 Q66GS9	Q66GS9	Centrosomal protein of 135 kDa	CEP135	Homo sapiens	133.491	5	5	156230	75100
5050	1 Q68CQ4	Q68CQ4	U3 small nucleolar RNA-associated protein 25 homolog	UTP25	Homo sapiens	87.058	5	5	238107	0
5051	1 Q6DKI1	Q6DKI1	60S ribosomal protein L7-like 1	RPL7L1	Homo sapiens	29.669	5	5	767847	185575
5052	1 Q6DKK2	Q6DKK2	Tetratricopeptide repeat protein 19, mitochondrial	TTC19	Homo sapiens	42.456	5	5	416011	103533
5053	1 Q6MZZ7	Q6MZZ7	Calpain-13	CAPN13	Homo sapiens	76.699	5	5	284070	0
5054	1 Q6P0Q8	Q6P0Q8	Microtubule-associated serine/threonine-protein kinase 2	MAST2	Homo sapiens	196.438	7	5	234473	46167
5055	1 Q6P1K2	Q6P1K2	Polyamine-modulated factor 1	PMF1	Homo sapiens	23.339	5	5	531096	208210
5056	1 Q6P1X5	Q6P1X5	Transcription initiation factor TFIID subunit 2	TAF2	Homo sapiens	136.974	5	5	2481151	0
5057	1 Q6PCB5	Q6PCB5	Lysine-specific demethylase RSBN1L	RSBN1L	Homo sapiens	94.871	5	5	295546	78770
5058	1 Q6PI78	Q6PI78	Transmembrane protein 65	TMEM65	Homo sapiens	25.497	5	5	1649160	380305
5059	1 Q6PL24	Q6PL24	Protein TMED8	TMED8	Homo sapiens	35.742	5	5	1212475	2160689
5060	1 Q6Q0C0	Q6Q0C0	E3 ubiquitin-protein ligase TRAF7	TRAF7	Homo sapiens	74.61	5	5	379956	99423
5061	1 Q6RFH5	Q6RFH5	WD repeat-containing protein 74	WDR74	Homo sapiens	42.441	5	5	452002	120384
5062	1 Q6UN15	Q6UN15	Pre-mRNA 3'-end-processing factor FIP1	FIP1L1	Homo sapiens	66.529	5	5	1467742	315675
5063	1 Q6UWH4	Q6UWH4	Golgi-associated kinase 1B	GASK1B	Homo sapiens	57.55	5	5	434660	110267
5064	1 Q6UX04	Q6UX04	Spliceosome-associated protein CWC27 homolog	CWC27	Homo sapiens	53.846	5	5	624517	266021
5065	1 Q6UX41	Q6UX41	Butyrophilin-like protein 8	BTNL8	Homo sapiens	56.75	6	5	377159	0
5066	1 Q6UX53	Q6UX53	Thiol S-methyltransferase METTL7B	METTL7B	Homo sapiens	27.776	5	5	1104077	345200
5067	1 Q6UXB8	Q6UXB8	Peptidase inhibitor 16	PI16	Homo sapiens	49.471	5	5	569571	71180
5068	1 Q6UXI9	Q6UXI9	Nephronectin	NPNT	Homo sapiens	61.906	5	5	365886	74443
5069	1 Q6ZRS2	Q6ZRS2	Helicase SRCAP	SRCAP	Homo sapiens	343.561	5	5	336803	23161
5070	1 Q6ZVK8	Q6ZVK8	8-oxo-dGDP phosphatase NUDT18	NUDT18	Homo sapiens	35.498	5	5	507002	300603
5071	1 Q76FK4	Q76FK4	Nucleolar protein 8	NOL8	Homo sapiens	131.619	5	5	132747	22219
5072	1 Q7L0J3	Q7L0J3	Synaptic vesicle glycoprotein 2A	SV2A	Homo sapiens	82.697	5	5	272891	0
5073	1 Q7L266	Q7L266	Isoaspartyl peptidase/L-asparaginase	ASRGL1	Homo sapiens	32.055	5	5	441020	454359
5074	1 Q7L273	Q7L273	BTB/POZ domain-containing protein KCTD9	KCTD9	Homo sapiens	42.568	5	5	203080	47011
5075	1 Q7L3T8	Q7L3T8	Probable proline--tRNA ligase, mitochondrial	PARS2	Homo sapiens	53.263	5	5	499955	38828
5076	1 Q7L8L6	Q7L8L6	FAST kinase domain-containing protein 5, mitochondrial	FASTKD5	Homo sapiens	86.577	5	5	664313	181604
5077	1 Q7RTS9	Q7RTS9	Dymeclin	DYM	Homo sapiens	75.935	5	5	548731	0
5078	1 Q7Z2W9	Q7Z2W9	39S ribosomal protein L21, mitochondrial	MRPL21	Homo sapiens	22.816	5	5	2054144	501762
5079	1 Q7Z333	Q7Z333	Probable helicase senataxin	SETX	Homo sapiens	302.884	5	5	155970	0
5080	1 Q7Z422	Q7Z422	SUZ domain-containing protein 1	SZRD1	Homo sapiens	16.995	5	5	1198968	184682
5081	1 Q7Z4I7	Q7Z4I7	LIM and senescent cell antigen-like-containing domain protein 2	LIMS2	Homo sapiens	38.915	5	5	5246107	0
5082	1 Q7Z4Q2	Q7Z4Q2	HEAT repeat-containing protein 3	HEATR3	Homo sapiens	74.583	5	5	766522	115073
5083	1 Q7Z4V5	Q7Z4V5	Hepatoma-derived growth factor-related protein 2	HDGFL2	Homo sapiens	74.317	6	5	2550570	742308
5084	1 Q7Z5J4	Q7Z5J4	Retinoic acid-induced protein 1	RAI1	Homo sapiens	203.354	5	5	215878	0
5085	1 Q7Z6J4	Q7Z6J4	FYVE, RhoGEF and PH domain-containing protein 2	FGD2	Homo sapiens	74.893	5	5	358035	197968
5086	1 Q7Z739	Q7Z739	YTH domain-containing family protein 3	YTHDF3	Homo sapiens	63.863	10	5	1265705	420348
5087	1 Q7Z7A4	Q7Z7A4	PX domain-containing protein kinase-like protein	PXK	Homo sapiens	64.95	5	5	873274	865969
5088	1 Q7Z7H5	Q7Z7H5	Transmembrane emp24 domain-containing protein 4	TMED4	Homo sapiens	25.943	5	5	1788165	1572737
5089	1 Q86SR1	Q86SR1	Polypeptide N-acetylgalactosaminyltransferase 10	GALNT10	Homo sapiens	68.991	5	5	164726	11381
5090	1 Q86SZ2	Q86SZ2	Trafficking protein particle complex subunit 6B	TRAPPC6B	Homo sapiens	17.984	5	5	784138	310229
5091	1 Q86UQ4	Q86UQ4	ATP-binding cassette sub-family A member 13	ABCA13	Homo sapiens	576.17	5	5	0	236453
5092	1 Q86US8	Q86US8	Telomerase-binding protein EST1A	SMG6	Homo sapiens	160.463	5	5	769993	48459
5093	1 Q86VR2	Q86VR2	Reticulophagy regulator 3	RETREG3	Homo sapiens	51.394	5	5	1050076	731132
5094	1 Q86VX9	Q86VX9	Vacuolar fusion protein MON1 homolog A	MON1A	Homo sapiens	72.897	5	5	221644	9893

5095	1	Q86VY4	Q86VY4	Testis-specific Y-encoded-like protein 5	TSPYL5	Homo sapiens	45.143	5	5	394324	0
5096	1	Q86WV6	Q86WV6	Stimulator of interferon genes protein	STING1	Homo sapiens	42.195	5	5	1057861	1450809
5097	1	Q86XP1	Q86XP1	Diacylglycerol kinase eta	DGKH	Homo sapiens	134.868	5	5	371326	0
5098	1	Q86XZ4	Q86XZ4	Spermatogenesis-associated serine-rich protein 2	SPATS2	Homo sapiens	59.544	5	5	375045	387675
5099	1	Q86Y34	Q86Y34	Adhesion G protein-coupled receptor G3	ADGRG3	Homo sapiens	60.862	5	5	0	2305709
5100	1	Q86Y39	Q86Y39	NADH dehydrogenase [ubiquinone] 1 alpha subcomplex subunit 11	NDUFA11	Homo sapiens	14.849	5	5	6647407	923806
5101	1	Q86YQ8	Q86YQ8	Copine-8	CPNE8	Homo sapiens	63.108	8	5	824801	93679
5102	1	Q86YV9	Q86YV9	BLOC-2 complex member HPS6	HPS6	Homo sapiens	82.973	5	5	326011	252854
5103	1	Q8IWA0	Q8IWA0	WD repeat-containing protein 75	WDR75	Homo sapiens	94.5	5	5	620223	109308
5104	1	Q8IWE5	Q8IWE5	Pleckstrin homology domain-containing family M member 2	PLEKHM2	Homo sapiens	112.78	5	5	497651	136696
5105	1	Q8IX19	Q8IX19	Mast cell-expressed membrane protein 1	MCEMP1	Homo sapiens	21.23	5	5	0	5364725
5106	1	Q8IXM2	Q8IXM2	Chromatin complexes subunit BAP18	BAP18	Homo sapiens	17.9	5	5	1761257	2295630
5107	1	Q8IXQ4	Q8IXQ4	GPALPP motifs-containing protein 1	GPALPP1	Homo sapiens	38.144	5	5	595428	0
5108	1	Q8IY22	Q8IY22	C-Maf-inducing protein	CMIP	Homo sapiens	86.331	5	5	518637	183748
5109	1	Q8IY95	Q8IY95	Transmembrane protein 192	TMEM192	Homo sapiens	30.923	5	5	1014379	926468
5110	1	Q8IZ69	Q8IZ69	tRNA (uracil-5-)-methyltransferase homolog A	TRMT2A	Homo sapiens	68.727	5	5	227805	334691
5111	1	Q8IZ73	Q8IZ73	Pseudouridylate synthase RPUSD2	RPUSD2	Homo sapiens	61.312	5	5	484212	36351
5112	1	Q8IZD4	Q8IZD4	mRNA-decapping enzyme 1B	DCP1B	Homo sapiens	67.725	5	5	324954	0
5113	1	Q8IZP0	Q8IZP0	Abl interactor 1	ABI1	Homo sapiens	55.081	9	5	2616487	1317561
5114	1	Q8N283	Q8N283	Ankyrin repeat domain-containing protein 35	ANKRD35	Homo sapiens	109.967	5	5	705062	0
5115	1	Q8N3R9	Q8N3R9	Protein PALS1	PALS1	Homo sapiens	77.295	5	5	248530	0
5116	1	Q8N474	Q8N474	Secreted frizzled-related protein 1	SFRP1	Homo sapiens	35.385	5	5	1666513	0
5117	1	Q8N568	Q8N568	Serine/threonine-protein kinase DCLK2	DCLK2	Homo sapiens	83.608	5	5	251004	0
5118	1	Q8N5A5	Q8N5A5	Zinc finger CCCH-type with G patch domain-containing protein	ZGPAT	Homo sapiens	57.36	5	5	226144	40263
5119	1	Q8N5J2	Q8N5J2	Ubiquitin carboxyl-terminal hydrolase MINDY-1	MINDY1	Homo sapiens	51.778	6	5	603063	555323
5120	1	Q8N5M4	Q8N5M4	Tetratricopeptide repeat protein 9C	TTC9C	Homo sapiens	20.013	5	5	1512047	460551
5121	1	Q8N697	Q8N697	Solute carrier family 15 member 4	SLC15A4	Homo sapiens	62.032	5	5	392270	3942304
5122	1	Q8N6G5	Q8N6G5	Chondroitin sulfate N-acetylgalactosaminyltransferase 2	CSGALNACT2	Homo sapiens	62.572	5	5	22094	232836
5123	1	Q8N6M0	Q8N6M0	Deubiquitinase OTUD6B	OTUD6B	Homo sapiens	33.812	5	5	595799	416049
5124	1	Q8N884	Q8N884	Cyclic GMP-AMP synthase	CGAS	Homo sapiens	58.815	5	5	27963	339279
5125	1	Q8N8A6	Q8N8A6	ATP-dependent RNA helicase DDX51	DDX51	Homo sapiens	72.459	5	5	329317	43811
5126	1	Q8N8N7	Q8N8N7	Prostaglandin reductase 2	PTGR2	Homo sapiens	38.498	5	5	2565799	275741
5127	1	Q8N8R5	Q8N8R5	Mitochondrial protein C2orf69	C2orf69	Homo sapiens	43.449	5	5	241032	20625
5128	1	Q8N960	Q8N960	Centrosomal protein of 120 kDa	CEP120	Homo sapiens	112.643	5	5	112744	48157
5129	1	Q8N9F7	Q8N9F7	Lysophospholipase D GDPD1	GDPD1	Homo sapiens	36.166	5	5	1076560	679712
5130	1	Q8N9T8	Q8N9T8	Protein KRI1 homolog	KRI1	Homo sapiens	82.601	5	5	647244	52615
5131	1	Q8NA72	Q8NA72	Centrosomal protein POC5	POC5	Homo sapiens	63.351	5	5	127472	18490
5132	1	Q8NBI6	Q8NBI6	Xyloside xylosyltransferase 1	XXYLT1	Homo sapiens	43.806	5	5	573745	0
5133	1	Q8NCF5	Q8NCF5	NFATC2-interacting protein	NFATC2IP	Homo sapiens	45.816	5	5	430159	80121
5134	1	Q8NDX5	Q8NDX5	Polyhomeotic-like protein 3	PHC3	Homo sapiens	106.162	6	5	661247	0
5135	1	Q8NEW0	Q8NEW0	Zinc transporter 7	SLC30A7	Homo sapiens	41.625	5	5	1437733	807638
5136	1	Q8NEY8	Q8NEY8	Periphilin-1	PPHLN1	Homo sapiens	52.736	5	5	442516	24020
5137	1	Q8NEZ2	Q8NEZ2	Vacuolar protein sorting-associated protein 37A	VPS37A	Homo sapiens	44.314	5	5	755536	710772
5138	1	Q8NFH4	Q8NFH4	Nucleoporin Nup37	NUP37	Homo sapiens	36.709	5	5	1181168	644145
5139	1	Q8NFX7	Q8NFX7	Syntaxin-binding protein 6	STXBP6	Homo sapiens	23.556	5	5	773302	0
5140	1	Q8NHG8	Q8NHG8	E3 ubiquitin-protein ligase ZNRF2	ZNRF2	Homo sapiens	24.115	5	5	1057061	193501
5141	1	Q8NHQ8	Q8NHQ8	Ras association domain-containing protein 8	RASSF8	Homo sapiens	48.327	5	5	768654	0
5142	1	Q8NI99	Q8NI99	Angiopoietin-related protein 6	ANGPTL6	Homo sapiens	51.694	5	5	47528	417210

5143	1	Q8TAD4	Q8TAD4	Proton-coupled zinc antiporter SLC30A5	SLC30A5	Homo sapiens	84.046	5	5	368236	159728
5144	1	Q8TAE8	Q8TAE8	Growth arrest and DNA damage-inducible proteins-interacting protein 1	GADD45GIP1	Homo sapiens	25.382	5	5	1023462	30158
5145	1	Q8TB61	Q8TB61	Adenosine 3'-phospho 5'-phosphosulfate transporter 1	SLC35B2	Homo sapiens	47.515	5	5	3779227	2684946
5146	1	Q8TBC5	Q8TBC5	Zinc finger and SCAN domain-containing protein 18	ZSCAN18	Homo sapiens	54.802	5	5	222533	0
5147	1	Q8TBN0	Q8TBN0	Guanine nucleotide exchange factor for Rab-3A	RAB3IL1	Homo sapiens	42.638	5	5	279613	19277
5148	1	Q8TBP6	Q8TBP6	Probable mitochondrial glutathione transporter SLC25A40	SLC25A40	Homo sapiens	38.124	5	5	695141	253792
5149	1	Q8TBZ3	Q8TBZ3	WD repeat-containing protein 20	WDR20	Homo sapiens	62.894	5	5	539838	26336
5150	1	Q8TC05	Q8TC05	Nuclear protein MDM1	MDM1	Homo sapiens	80.735	5	5	69243	78103
5151	1	Q8TCD5	Q8TCD5	5'(3')-deoxyribonucleotidase, cytosolic type	NT5C	Homo sapiens	23.384	5	5	3781228	280545
5152	1	Q8TD06	Q8TD06	Anterior gradient protein 3	AGR3	Homo sapiens	19.172	5	5	25025036	26600
5153	1	Q8TDM6	Q8TDM6	Disks large homolog 5	DLG5	Homo sapiens	213.869	5	5	237905	0
5154	1	Q8TDQ7	Q8TDQ7	Glucosamine-6-phosphate isomerase 2	GNPDA2	Homo sapiens	31.088	5	5	2543736	235319
5155	1	Q8TF01	Q8TF01	Arginine/serine-rich protein PNISR	PNISR	Homo sapiens	92.577	5	5	665756	18078
5156	1	Q8TF62	Q8TF62	Probable phospholipid-transporting ATPase IM	ATP8B4	Homo sapiens	135.869	5	5	0	412990
5157	1	Q8WTS1	Q8WTS1	1-acylglycerol-3-phosphate O-acyltransferase ABHD5	ABHD5	Homo sapiens	39.095	5	5	148455	581401
5158	1	Q8WTT2	Q8WTT2	Nucleolar complex protein 3 homolog	NOC3L	Homo sapiens	92.547	5	5	485829	41142
5159	1	Q8WUH6	Q8WUH6	Transmembrane protein 263	TMEM263	Homo sapiens	11.747	5	5	2698934	1552742
5160	1	Q8WUQ7	Q8WUQ7	Splicing factor Cactin	CACTIN	Homo sapiens	88.7	5	5	372014	39829
5161	1	Q8WUW1	Q8WUW1	Protein BRICK1	BRK1	Homo sapiens	8.74	5	5	466532	293837
5162	1	Q8WVC6	Q8WVC6	Dephospho-CoA kinase domain-containing protein	DCAKD	Homo sapiens	26.549	5	5	1406521	917290
5163	1	Q8WWC4	Q8WWC4	m-AAA protease-interacting protein 1, mitochondrial	MAIP1	Homo sapiens	32.545	5	5	1290667	338069
5164	1	Q8WYA0	Q8WYA0	Intraflagellar transport protein 81 homolog	IFT81	Homo sapiens	79.747	5	5	213106	0
5165	1	Q8WZ64	Q8WZ64	Arf-GAP with Rho-GAP domain, ANK repeat and PH domain-containing protein 2	ARAP2	Homo sapiens	193.456	5	5	30471	130922
5166	1	Q8WZA0	Q8WZA0	Protein LZIC	LZIC	Homo sapiens	21.491	5	5	2579182	513541
5167	1	Q8WZA1	Q8WZA1	Protein O-linked-mannose beta-1,2-N-acetylglucosaminyltransferase 1	POMGNT1	Homo sapiens	75.253	5	5	378047	223037
5168	1	Q92484	Q92484	Acid sphingomyelinase-like phosphodiesterase 3a	SMPDL3A	Homo sapiens	51.261	5	5	1207918	104755
5169	1	Q92530	Q92530	Proteasome inhibitor PI31 subunit	PSMF1	Homo sapiens	29.815	5	5	5025328	2977210
5170	1	Q92543	Q92543	Sorting nexin-19	SNX19	Homo sapiens	108.6	5	5	143351	0
5171	1	Q92562	Q92562	Polyphosphoinositide phosphatase	FIG4	Homo sapiens	103.636	5	5	313889	299102
5172	1	Q92575	Q92575	UBX domain-containing protein 4	UBXN4	Homo sapiens	56.779	5	5	3115876	887129
5173	1	Q92688	Q92688	Acidic leucine-rich nuclear phosphoprotein 32 family member B	ANP32B	Homo sapiens	28.786	5	5	21187514	15312558
5174	1	Q92769	Q92769	Histone deacetylase 2	HDAC2	Homo sapiens	55.362	5	5	534060	288830
5175	1	Q92791	Q92791	Endoplasmic reticulum protein SC65	P3H4	Homo sapiens	50.381	5	5	236379	212746
5176	1	Q92793	Q92793	CREB-binding protein	CREBBP	Homo sapiens	265.356	5	5	348352	89605
5177	1	Q92824	Q92824	Proprotein convertase subtilisin/kexin type 5	PCSK5	Homo sapiens	206.947	5	5	11370	401611
5178	1	Q92844	Q92844	TRAF family member-associated NF-kappa-B activator	TANK	Homo sapiens	47.817	5	5	385512	642042
5179	1	Q92871	Q92871	Phosphomannomutase 1	PMM1	Homo sapiens	29.748	5	5	749081	40457
5180	1	Q92889	Q92889	DNA repair endonuclease XPF	ERCC4	Homo sapiens	104.486	5	5	207581	74927
5181	1	Q969H8	Q969H8	Myeloid-derived growth factor	MYDGF	Homo sapiens	18.795	5	5	4385835	3591073
5182	1	Q969U7	Q969U7	Proteasome assembly chaperone 2	PSMG2	Homo sapiens	29.397	5	5	539216	275573
5183	1	Q969X2	Q969X2	Alpha-N-acetylgalactosaminide alpha-2,6-sialyltransferase 6	ST6GALNAC6	Homo sapiens	38.067	5	5	523317	0
5184	1	Q969X6	Q969X6	U3 small nucleolar RNA-associated protein 4 homolog	UTP4	Homo sapiens	76.89	5	5	505817	147330
5185	1	Q96AG3	Q96AG3	Mitochondrial outer membrane protein SLC25A46	SLC25A46	Homo sapiens	46.174	5	5	912196	59330
5186	1	Q96BH1	Q96BH1	E3 ubiquitin-protein ligase RNF25	RNF25	Homo sapiens	51.22	5	5	290551	0
5187	1	Q96BQ5	Q96BQ5	Coiled-coil domain-containing protein 127	CCDC127	Homo sapiens	30.833	5	5	434051	55924
5188	1	Q96BW1	Q96BW1	Uracil phosphoribosyltransferase homolog	UPRT	Homo sapiens	33.788	5	5	361884	141787
5189	1	Q96BW5	Q96BW5	Phosphotriesterase-related protein	PTER	Homo sapiens	39.018	5	5	2048656	666655

5190	1	Q96CF2	Q96CF2	Charged multivesicular body protein 4c	CHMP4C	Homo sapiens	26.409	5	5	686655	32921
5191	1	Q96CU9	Q96CU9	FAD-dependent oxidoreductase domain-containing protein 1	FOXRED1	Homo sapiens	53.812	5	5	929169	104322
5192	1	Q96D96	Q96D96	Voltage-gated hydrogen channel 1	HVCN1	Homo sapiens	31.683	5	5	1276466	2474037
5193	1	Q96DH6	Q96DH6	RNA-binding protein Musashi homolog 2	MSI2	Homo sapiens	35.195	6	5	1261126	190957
5194	1	Q96EB1	Q96EB1	Elongator complex protein 4	ELP4	Homo sapiens	46.588	5	5	358011	40153
5195	1	Q96EQ0	Q96EQ0	Small glutamine-rich tetratricopeptide repeat-containing protein beta	SGTB	Homo sapiens	33.432	5	5	1151846	1072015
5196	1	Q96EU7	Q96EU7	C1GALT1-specific chaperone 1	C1GALT1C1	Homo sapiens	36.382	5	5	497549	913635
5197	1	Q96FQ6	Q96FQ6	Protein S100-A16	S100A16	Homo sapiens	11.802	5	5	4710068	631593
5198	1	Q96GA7	Q96GA7	Serine dehydratase-like	SDSL	Homo sapiens	34.674	5	5	1173784	124149
5199	1	Q96GM5	Q96GM5	SWI/SNF-related matrix-associated actin-dependent regulator of chromatin subfamily D member 1	SMARCD1	Homo sapiens	58.232	6	5	407092	39223
5200	1	Q96GM8	Q96GM8	Target of EGR1 protein 1	TOE1	Homo sapiens	56.549	5	5	398843	52035
5201	1	Q96H79	Q96H79	Zinc finger CCCH-type antiviral protein 1-like	ZC3HAV1L	Homo sapiens	32.961	5	5	3630134	1422319
5202	1	Q96I51	Q96I51	RCC1-like G exchanging factor-like protein	RCC1L	Homo sapiens	49.897	5	5	452934	0
5203	1	Q96I59	Q96I59	Probable asparagine--tRNA ligase, mitochondrial	NARS2	Homo sapiens	54.093	5	5	847020	151114
5204	1	Q96IG2	Q96IG2	F-box/LRR-repeat protein 20	FBXL20	Homo sapiens	48.424	5	5	259528	370167
5205	1	Q96IQ7	Q96IQ7	V-set and immunoglobulin domain-containing protein 2	VSIG2	Homo sapiens	34.348	5	5	2424893	18680
5206	1	Q96IZ0	Q96IZ0	PRKC apoptosis WT1 regulator protein	PAWR	Homo sapiens	36.566	5	5	2643509	219005
5207	1	Q96JB6	Q96JB6	Lysyl oxidase homolog 4	LOXL4	Homo sapiens	84.487	5	5	796733	0
5208	1	Q96K49	Q96K49	Transmembrane protein 87B	TMEM87B	Homo sapiens	63.536	5	5	506687	0
5209	1	Q96M27	Q96M27	Protein PRRC1	PRRC1	Homo sapiens	46.701	5	5	2997378	543504
5210	1	Q96NB2	Q96NB2	Sideroflexin-2	SFXN2	Homo sapiens	36.232	5	5	1131816	99120
5211	1	Q96P47	Q96P47	Arf-GAP with GTPase, ANK repeat and PH domain-containing protein 3	AGAP3	Homo sapiens	95.046	5	5	785050	65804
5212	1	Q96PP9	Q96PP9	Guanylate-binding protein 4	GBP4	Homo sapiens	73.166	6	5	188936	1131160
5213	1	Q96RN5	Q96RN5	Mediator of RNA polymerase II transcription subunit 15	MED15	Homo sapiens	86.753	5	5	346642	110574
5214	1	Q96S97	Q96S97	Myeloid-associated differentiation marker	MYADM	Homo sapiens	35.275	5	5	9216874	2327173
5215	1	Q96SL4	Q96SL4	Glutathione peroxidase 7	GPX7	Homo sapiens	20.997	5	5	180930	218063
5216	1	Q96SZ5	Q96SZ5	2-aminoethanethiol dioxygenase	ADO	Homo sapiens	29.748	5	5	825182	178727
5217	1	Q99417	Q99417	c-Myc-binding protein	MYCBP	Homo sapiens	11.963	5	5	3174248	1309276
5218	1	Q99549	Q99549	M-phase phosphoprotein 8	MPHOSPH8	Homo sapiens	97.183	5	5	175257	112006
5219	1	Q99627	Q99627	COP9 signalosome complex subunit 8	COPS8	Homo sapiens	23.226	5	5	3117942	572687
5220	1	Q99755	Q99755	Phosphatidylinositol 4-phosphate 5-kinase type-1 alpha	PIP5K1A	Homo sapiens	62.632	8	5	630676	201131
5221	1	Q99848	Q99848	Probable rRNA-processing protein EBP2	EBNA1BP2	Homo sapiens	34.851	5	5	1417216	264685
5222	1	Q9BQ69	Q9BQ69	ADP-ribose glycohydrolase MACROD1	MACROD1	Homo sapiens	35.505	5	5	2510421	0
5223	1	Q9BRC7	Q9BRC7	1-phosphatidylinositol 4,5-bisphosphate phosphodiesterase delta-4	PLCD4	Homo sapiens	87.586	5	5	745137	0
5224	1	Q9BRJ6	Q9BRJ6	Uncharacterized protein C7orf50	C7orf50	Homo sapiens	22.087	5	5	1133947	335361
5225	1	Q9BRQ6	Q9BRQ6	MICOS complex subunit MIC25	CHCHD6	Homo sapiens	26.458	5	5	931478	0
5226	1	Q9BS40	Q9BS40	Latexin	LXN	Homo sapiens	25.749	5	5	3596004	2321249
5227	1	Q9BSU1	Q9BSU1	Phagosome assembly factor 1	PHAF1	Homo sapiens	47.525	5	5	637540	104830
5228	1	Q9BTE7	Q9BTE7	DCN1-like protein 5	DCUN1D5	Homo sapiens	27.509	5	5	327015	83105
5229	1	Q9BU61	Q9BU61	NADH dehydrogenase [ubiquinone] 1 alpha subcomplex assembly factor 3	NDUFAF3	Homo sapiens	20.352	5	5	1931473	298078
5230	1	Q9BU89	Q9BU89	Deoxyhypusine hydroxylase	DOHH	Homo sapiens	32.902	5	5	178506	49015
5231	1	Q9BUL8	Q9BUL8	Programmed cell death protein 10	PDCD10	Homo sapiens	24.7	5	5	4777291	2093983
5232	1	Q9BUT1	Q9BUT1	Dehydrogenase/reductase SDR family member 6	BDH2	Homo sapiens	26.723	5	5	8256919	1449685
5233	1	Q9BVI4	Q9BVI4	Nucleolar complex protein 4 homolog	NOC4L	Homo sapiens	58.47	5	5	697760	106507
5234	1	Q9BVV7	Q9BVV7	Mitochondrial import inner membrane translocase subunit Tim21	TIMM21	Homo sapiens	28.203	5	5	1796369	602495
5235	1	Q9BW62	Q9BW62	Katanin p60 ATPase-containing subunit A-like 1	KATNAL1	Homo sapiens	55.394	5	5	339299	34584

5236	1 Q9BYB0	Q9BYB0	SH3 and multiple ankyrin repeat domains protein 3	SHANK3	Homo sapiens	184.669	5	5	219998	0
5237	1 Q9BYB4	Q9BYB4	Guanine nucleotide-binding protein subunit beta-like protein 1	GNB1L	Homo sapiens	35.617	5	5	326811	65184
5238	1 Q9BYC5	Q9BYC5	Alpha-(1,6)-fucosyltransferase	FUT8	Homo sapiens	66.515	5	5	596538	173780
5239	1 Q9BYD2	Q9BYD2	39S ribosomal protein L9, mitochondrial	MRPL9	Homo sapiens	30.242	5	5	2354066	216889
5240	1 Q9BYD6	Q9BYD6	39S ribosomal protein L1, mitochondrial	MRPL1	Homo sapiens	36.909	5	5	3333098	441808
5241	1 Q9BYN0	Q9BYN0	Sulfiredoxin-1	SRXN1	Homo sapiens	14.257	5	5	19200	416641
5242	1 Q9BZ71	Q9BZ71	Membrane-associated phosphatidylinositol transfer protein 3	PITPNM3	Homo sapiens	106.782	5	5	444792	0
5243	1 Q9BZI7	Q9BZI7	Regulator of nonsense transcripts 3B	UPF3B	Homo sapiens	57.76	5	5	911114	178314
5244	1 Q9C0C4	Q9C0C4	Semaphorin-4C	SEMA4C	Homo sapiens	92.624	5	5	470399	0
5245	1 Q9GZN1	Q9GZN1	Actin-related protein 6	ACTR6	Homo sapiens	45.809	5	5	428183	0
5246	1 Q9GZN8	Q9GZN8	UPF0687 protein C20orf27	C20orf27	Homo sapiens	19.293	5	5	1993903	1471904
5247	1 Q9GZZ9	Q9GZZ9	Ubiquitin-like modifier-activating enzyme 5	UBA5	Homo sapiens	44.861	5	5	2490529	1559517
5248	1 Q9H074	Q9H074	Polyadenylate-binding protein-interacting protein 1	PAIP1	Homo sapiens	53.524	5	5	1986729	227007
5249	1 Q9H082	Q9H082	Ras-related protein Rab-33B	RAB33B	Homo sapiens	25.719	6	5	652280	1154508
5250	1 Q9H0E2	Q9H0E2	Toll-interacting protein	TOLLIP	Homo sapiens	30.283	5	5	2135183	1681082
5251	1 Q9H0F6	Q9H0F6	Sharpin	SHARPIN	Homo sapiens	39.951	5	5	225386	112253
5252	1 Q9H0F7	Q9H0F7	ADP-ribosylation factor-like protein 6	ARL6	Homo sapiens	21.099	5	5	309009	63104
5253	1 Q9H0H0	Q9H0H0	Integrator complex subunit 2	INTS2	Homo sapiens	134.326	5	5	573047	74455
5254	1 Q9H0L4	Q9H0L4	Cleavage stimulation factor subunit 2 tau variant	CSTF2T	Homo sapiens	64.435	5	5	636001	128434
5255	1 Q9H0N5	Q9H0N5	Pterin-4-alpha-carbinolamine dehydratase 2	PCBD2	Homo sapiens	14.365	5	5	1386790	342207
5256	1 Q9H0V1	Q9H0V1	Transmembrane protein 168	TMEM168	Homo sapiens	79.756	5	5	821264	100609
5257	1 Q9H147	Q9H147	Deoxynucleotidyltransferase terminal-interacting protein 1	DNTTIP1	Homo sapiens	37.011	5	5	546371	73691
5258	1 Q9H257	Q9H257	Caspase recruitment domain-containing protein 9	CARD9	Homo sapiens	62.243	5	5	1130589	2166610
5259	1 Q9H2P9	Q9H2P9	Diphthine methyl ester synthase	DPH5	Homo sapiens	31.65	5	5	406987	158150
5260	1 Q9H4A5	Q9H4A5	Golgi phosphoprotein 3-like	GOLPH3L	Homo sapiens	32.767	5	5	2670814	216820
5261	1 Q9H4A6	Q9H4A6	Golgi phosphoprotein 3	GOLPH3	Homo sapiens	33.812	5	5	2713823	1070644
5262	1 Q9H4I3	Q9H4I3	TraB domain-containing protein	TRABD	Homo sapiens	42.321	5	5	242652	300389
5263	1 Q9H4L4	Q9H4L4	Sentrin-specific protease 3	SENP3	Homo sapiens	65.012	5	5	468398	18716
5264	1 Q9H501	Q9H501	ESF1 homolog	ESF1	Homo sapiens	98.796	5	5	322941	40079
5265	1 Q9H553	Q9H553	Alpha-1,3/1,6-mannosyltransferase ALG2	ALG2	Homo sapiens	47.091	5	5	2435115	980490
5266	1 Q9H5X1	Q9H5X1	Cytosolic iron-sulfur assembly component 2A	CIAO2A	Homo sapiens	18.355	5	5	741595	646191
5267	1 Q9H6D7	Q9H6D7	HAUS augmin-like complex subunit 4	HAUS4	Homo sapiens	42.4	5	5	93614	296273
5268	1 Q9H6E4	Q9H6E4	Coiled-coil domain-containing protein 134	CCDC134	Homo sapiens	26.561	5	5	569343	326923
5269	1 Q9H6F5	Q9H6F5	Coiled-coil domain-containing protein 86	CCDC86	Homo sapiens	40.235	5	5	1399098	199263
5270	1 Q9H6U8	Q9H6U8	Alpha-1,2-mannosyltransferase ALG9	ALG9	Homo sapiens	69.865	5	5	782992	178434
5271	1 Q9H788	Q9H788	SH2 domain-containing protein 4A	SH2D4A	Homo sapiens	52.726	5	5	234884	92342
5272	1 Q9H7C9	Q9H7C9	Mth938 domain-containing protein	AAMDC	Homo sapiens	13.331	5	5	3296674	454254
5273	1 Q9H7L9	Q9H7L9	Sin3 histone deacetylase corepressor complex component SDS3	SUDS3	Homo sapiens	38.134	5	5	832048	131411
5274	1 Q9H7N4	Q9H7N4	Splicing factor, arginine/serine-rich 19	SCAF1	Homo sapiens	139.274	5	5	323852	87092
5275	1 Q9H7Z6	Q9H7Z6	Histone acetyltransferase KAT8	KAT8	Homo sapiens	52.404	5	5	575206	100042
5276	1 Q9H9B1	Q9H9B1	Histone-lysine N-methyltransferase EHMT1	EHMT1	Homo sapiens	141.469	5	5	263290	941961
5277	1 Q9HBL7	Q9HBL7	Plasminogen receptor (KT)	PLGRKT	Homo sapiens	17.201	5	5	5974056	2972487
5278	1 Q9HCN4	Q9HCN4	GPN-loop GTPase 1	GPN1	Homo sapiens	41.739	5	5	890632	79567
5279	1 Q9HCY8	Q9HCY8	Protein S100-A14	S100A14	Homo sapiens	11.661	5	5	2937208	63341
5280	1 Q9HD67	Q9HD67	Unconventional myosin-X	MYO10	Homo sapiens	237.352	5	5	148825	0
5281	1 Q9HD89	Q9HD89	Resistin	RETN	Homo sapiens	11.419	5	5	852350	31345938
5282	1 Q9NNX1	Q9NNX1	Tuftelin	TUFT1	Homo sapiens	44.264	5	5	268296	0
5283	1 Q9NP92	Q9NP92	39S ribosomal protein S30, mitochondrial	MRPS30	Homo sapiens	50.366	5	5	1161395	0

5284	1	Q9NPH0	Q9NPH0	Lysophosphatidic acid phosphatase type 6	ACP6	Homo sapiens	48.885	5	5	1274267	305396
5285	1	Q9NPH2	Q9NPH2	Inositol-3-phosphate synthase 1	ISYNA1	Homo sapiens	61.068	5	5	1534431	318199
5286	1	Q9NQ75	Q9NQ75	Cas scaffolding protein family member 4	CASS4	Homo sapiens	87.144	5	5	0	252406
5287	1	Q9NQ89	Q9NQ89	Protein C12orf4	C12orf4	Homo sapiens	63.8	5	5	447068	280762
5288	1	Q9NQE9	Q9NQE9	Adenosine 5'-monophosphoramidase HINT3	HINT3	Homo sapiens	20.362	5	5	1378627	934266
5289	1	Q9NQG5	Q9NQG5	Regulation of nuclear pre-mRNA domain-containing protein 1B	RPRD1B	Homo sapiens	36.9	8	5	2440555	877530
5290	1	Q9NQS1	Q9NQS1	Cell death regulator Aven	AVEN	Homo sapiens	38.505	5	5	766773	242785
5291	1	Q9NRR5	Q9NRR5	Ubiquilin-4	UBQLN4	Homo sapiens	63.854	8	5	545040	99319
5292	1	Q9NRS4	Q9NRS4	Transmembrane protease serine 4	TMPRSS4	Homo sapiens	48.245	5	5	474009	313895
5293	1	Q9NTM9	Q9NTM9	Copper homeostasis protein cutC homolog	CUTC	Homo sapiens	29.34	5	5	901323	386827
5294	1	Q9NTN9	Q9NTN9	Semaphorin-4G	SEMA4G	Homo sapiens	91.498	5	5	438807	0
5295	1	Q9NVK5	Q9NVK5	FGFR1 oncogene partner 2	FGFR1OP2	Homo sapiens	29.425	5	5	418835	779416
5296	1	Q9NVM6	Q9NVM6	DnaJ homolog subfamily C member 17	DNAJC17	Homo sapiens	34.686	5	5	591433	1310892
5297	1	Q9NWS8	Q9NWS8	Required for meiotic nuclear division protein 1 homolog	RMND1	Homo sapiens	51.605	5	5	344791	181840
5298	1	Q9NWU1	Q9NWU1	3-oxoacyl-[acyl-carrier-protein] synthase, mitochondrial	OXSM	Homo sapiens	48.845	5	5	765639	170064
5299	1	Q9NWV4	Q9NWV4	CXXC motif containing zinc binding protein	CZIB	Homo sapiens	18.049	5	5	7906130	2057774
5300	1	Q9NWY4	Q9NWY4	Histone PARylation factor 1	HPF1	Homo sapiens	39.435	6	5	217412	0
5301	1	Q9NWZ5	Q9NWZ5	Uridine-cytidine kinase-like 1	UCKL1	Homo sapiens	61.143	5	5	342523	19782
5302	1	Q9NX47	Q9NX47	E3 ubiquitin-protein ligase MARCHF5	MARCHF5	Homo sapiens	31.231	5	5	522710	233497
5303	1	Q9NX55	Q9NX55	Huntingtin-interacting protein K	HYPK	Homo sapiens	13.651	5	5	1339302	231182
5304	1	Q9NX74	Q9NX74	tRNA-dihydrouridine(20) synthase [NAD(P)+]-like	DUS2	Homo sapiens	55.052	5	5	246077	48896
5305	1	Q9NXC2	Q9NXC2	Glucose-fructose oxidoreductase domain-containing protein 1	GFOD1	Homo sapiens	43.158	5	5	284860	362200
5306	1	Q9NXW9	Q9NXW9	Alpha-ketoglutarate-dependent dioxygenase alkB homolog 4	ALKBH4	Homo sapiens	33.839	5	5	402233	55049
5307	1	Q9NY97	Q9NY97	N-acetyllactosaminide beta-1,3-N-acetylglucosaminyltransferase 2	B3GNT2	Homo sapiens	46.022	5	5	214817	94044
5308	1	Q9NYB9	Q9NYB9	Abl interactor 2	ABI2	Homo sapiens	55.662	5	5	1371944	0
5309	1	Q9NYH9	Q9NYH9	U3 small nucleolar RNA-associated protein 6 homolog	UTP6	Homo sapiens	70.195	5	5	430645	50249
5310	1	Q9NYY8	Q9NYY8	FAST kinase domain-containing protein 2, mitochondrial	FASTKD2	Homo sapiens	81.463	5	5	640222	0
5311	1	Q9NZ09	Q9NZ09	Ubiquitin-associated protein 1	UBAP1	Homo sapiens	55.083	5	5	537566	558791
5312	1	Q9NZ45	Q9NZ45	CDGSH iron-sulfur domain-containing protein 1	CISD1	Homo sapiens	12.199	5	5	7770331	2951556
5313	1	Q9NZ52	Q9NZ52	ADP-ribosylation factor-binding protein GGA3	GGA3	Homo sapiens	78.317	5	5	517854	449529
5314	1	Q9P0I2	Q9P0I2	ER membrane protein complex subunit 3	EMC3	Homo sapiens	29.951	5	5	712593	652344
5315	1	Q9P1Z2	Q9P1Z2	Calcium-binding and coiled-coil domain-containing protein 1	CALCOCO1	Homo sapiens	77.337	5	5	586611	190776
5316	1	Q9P215	Q9P215	Pogo transposable element with KRAB domain	POGK	Homo sapiens	69.445	5	5	336638	41078
5317	1	Q9P227	Q9P227	Rho GTPase-activating protein 23	ARHGAP23	Homo sapiens	162.195	5	5	292758	0
5318	1	Q9P273	Q9P273	Teneurin-3	TENM3	Homo sapiens	300.955	6	5	240165	0
5319	1	Q9P291	Q9P291	Armadillo repeat-containing X-linked protein 1	ARMCX1	Homo sapiens	49.179	5	5	1227593	88893
5320	1	Q9P2D0	Q9P2D0	Inhibitor of Bruton tyrosine kinase	IBTK	Homo sapiens	150.53	5	5	122198	0
5321	1	Q9UBD5	Q9UBD5	Origin recognition complex subunit 3	ORC3	Homo sapiens	82.254	5	5	400649	0
5322	1	Q9UBI9	Q9UBI9	Headcase protein homolog	HECA	Homo sapiens	58.838	5	5	267995	16044
5323	1	Q9UBK8	Q9UBK8	Methionine synthase reductase	MTRR	Homo sapiens	77.675	5	5	253143	184795
5324	1	Q9UBW7	Q9UBW7	Zinc finger MYM-type protein 2	ZMYM2	Homo sapiens	154.913	5	5	280031	10714
5325	1	Q9UFW8	Q9UFW8	CGG triplet repeat-binding protein 1	CGGBP1	Homo sapiens	18.82	5	5	630928	471490
5326	1	Q9UGJ1	Q9UGJ1	Gamma-tubulin complex component 4	TUBGCP4	Homo sapiens	76.088	5	5	704436	84023
5327	1	Q9UGK3	Q9UGK3	Signal-transducing adaptor protein 2	STAP2	Homo sapiens	44.893	5	5	1647313	0
5328	1	Q9UGM6	Q9UGM6	Tryptophan--tRNA ligase, mitochondrial	WARS2	Homo sapiens	40.148	5	5	562481	134000
5329	1	Q9UHK6	Q9UHK6	Alpha-methylacyl-CoA racemase	AMACR	Homo sapiens	42.389	5	5	919104	20837
5330	1	Q9UHN1	Q9UHN1	DNA polymerase subunit gamma-2, mitochondrial	POLG2	Homo sapiens	54.912	5	5	353435	0
5331	1	Q9UHR5	Q9UHR5	SAP30-binding protein	SAP30BP	Homo sapiens	33.869	5	5	1248263	407711

5332	1	Q9UI09	Q9UI09	NADH dehydrogenase [ubiquinone] 1 alpha subcomplex subunit 12	NDUFA12	Homo sapiens	17.114	5	5	6817562	74305
5333	1	Q9UI30	Q9UI30	Multifunctional methyltransferase subunit TRM112-like protein	TRMT112	Homo sapiens	14.199	5	5	888713	220975
5334	1	Q9UIS9	Q9UIS9	Methyl-CpG-binding domain protein 1	MBD1	Homo sapiens	66.608	5	5	386999	110593
5335	1	Q9UJ41	Q9UJ41	Rab5 GDP/GTP exchange factor	RABGEF1	Homo sapiens	56.891	5	5	538314	410973
5336	1	Q9UJY4	Q9UJY4	ADP-ribosylation factor-binding protein GGA2	GGA2	Homo sapiens	67.149	5	5	517248	268190
5337	1	Q9UKL6	Q9UKL6	Phosphatidylcholine transfer protein	PCTP	Homo sapiens	24.843	5	5	2214030	763246
5338	1	Q9UKZ1	Q9UKZ1	CCR4-NOT transcription complex subunit 11	CNOT11	Homo sapiens	55.215	5	5	679418	354320
5339	1	Q9UL40	Q9UL40	Zinc finger protein 346	ZNF346	Homo sapiens	32.933	5	5	498297	170514
5340	1	Q9ULD0	Q9ULD0	2-oxoglutarate dehydrogenase-like, mitochondrial	OGDHL	Homo sapiens	114.481	5	5	114172	19728
5341	1	Q9ULH7	Q9ULH7	Myocardin-related transcription factor B	MRTFB	Homo sapiens	118.131	5	5	451903	118403
5342	1	Q9ULQ1	Q9ULQ1	Two pore channel protein 1	TPCN1	Homo sapiens	94.148	5	5	458704	327627
5343	1	Q9ULS5	Q9ULS5	Transmembrane and coiled-coil domain protein 3	TMCC3	Homo sapiens	53.784	5	5	389225	556119
5344	1	Q9UNH7	Q9UNH7	Sorting nexin-6	SNX6	Homo sapiens	46.647	7	5	5343811	3228204
5345	1	Q9UNI6	Q9UNI6	Dual specificity protein phosphatase 12	DUSP12	Homo sapiens	37.687	5	5	565599	174628
5346	1	Q9UNQ2	Q9UNQ2	Probable dimethyladenosine transferase	DIMT1	Homo sapiens	35.235	5	5	600538	84135
5347	1	Q9UP38	Q9UP38	Frizzled-1	FZD1	Homo sapiens	71.159	6	5	1808031	0
5348	1	Q9UP95	Q9UP95	Solute carrier family 12 member 4	SLC12A4	Homo sapiens	120.65	5	5	627854	133936
5349	1	Q9UPZ3	Q9UPZ3	BLOC-2 complex member HPS5	HPS5	Homo sapiens	127.45	5	5	140091	201638
5350	1	Q9UQ53	Q9UQ53	Alpha-1,3-mannosyl-glycoprotein 4-beta-N-acetylglucosaminyltransferase B	MGAT4B	Homo sapiens	63.198	5	5	243751	0
5351	1	Q9UQC2	Q9UQC2	GRB2-associated-binding protein 2	GAB2	Homo sapiens	74.458	5	5	535182	829778
5352	1	Q9Y240	Q9Y240	C-type lectin domain family 11 member A	CLEC11A	Homo sapiens	35.694	5	5	105209	1837784
5353	1	Q9Y296	Q9Y296	Trafficking protein particle complex subunit 4	TRAPPC4	Homo sapiens	24.339	5	5	1348502	665840
5354	1	Q9Y2I7	Q9Y2I7	1-phosphatidylinositol 3-phosphate 5-kinase	PIKFYVE	Homo sapiens	237.139	5	5	179110	138110
5355	1	Q9Y320	Q9Y320	Thioredoxin-related transmembrane protein 2	TMX2	Homo sapiens	34.036	5	5	1289307	118994
5356	1	Q9Y333	Q9Y333	U6 snRNA-associated Sm-like protein LSm2	LSM2	Homo sapiens	10.832	5	5	4284885	1928640
5357	1	Q9Y3B4	Q9Y3B4	Splicing factor 3B subunit 6	SF3B6	Homo sapiens	14.585	5	5	3103111	355932
5358	1	Q9Y426	Q9Y426	C2 domain-containing protein 2	C2CD2	Homo sapiens	75.535	5	5	464016	0
5359	1	Q9Y496	Q9Y496	Kinesin-like protein KIF3A	KIF3A	Homo sapiens	80.042	5	5	245541	38130
5360	1	Q9Y4C1	Q9Y4C1	Lysine-specific demethylase 3A	KDM3A	Homo sapiens	147.343	5	5	99468	65529
5361	1	Q9Y4K3	Q9Y4K3	TNF receptor-associated factor 6	TRAF6	Homo sapiens	59.572	5	5	446780	50692
5362	1	Q9Y508	Q9Y508	E3 ubiquitin-protein ligase RNF114	RNF114	Homo sapiens	25.694	5	5	1100806	713666
5363	1	Q9Y580	Q9Y580	RNA-binding protein 7	RBM7	Homo sapiens	30.504	5	5	357294	0
5364	1	Q9Y597	Q9Y597	BTB/POZ domain-containing protein KCTD3	KCTD3	Homo sapiens	88.984	5	5	635139	0
5365	1	Q9Y5A9	Q9Y5A9	YTH domain-containing family protein 2	YTHDF2	Homo sapiens	62.333	6	5	694942	146739
5366	1	Q9Y5B8	Q9Y5B8	Nucleoside diphosphate kinase 7	NME7	Homo sapiens	42.489	5	5	1267092	487525
5367	1	Q9Y5K8	Q9Y5K8	V-type proton ATPase subunit D	ATP6V1D	Homo sapiens	28.263	5	5	1636377	1506645
5368	1	Q9Y5Z7	Q9Y5Z7	Host cell factor 2	HCFC2	Homo sapiens	86.78	5	5	400589	0
5369	1	Q9Y619	Q9Y619	Mitochondrial ornithine transporter 1	SLC25A15	Homo sapiens	32.732	5	5	992771	709421
5370	1	Q9Y625	Q9Y625	Glypican-6	GPC6	Homo sapiens	62.736	5	5	988588	12314
5371	1	Q9Y679	Q9Y679	Lipid droplet-regulating VLDL assembly factor AUP1	AUP1	Homo sapiens	45.786	5	5	1038668	507073
5372	1	Q9Y6J0	Q9Y6J0	Calcineurin-binding protein cabin-1	CABIN1	Homo sapiens	246.357	5	5	499564	0
5373	1	Q9Y6M7	Q9Y6M7	Sodium bicarbonate cotransporter 3	SLC4A7	Homo sapiens	136.046	5	5	191970	114107
5374	1	Q9Y6X4	Q9Y6X4	Soluble lamin-associated protein of 75 kDa	FAM169A	Homo sapiens	74.954	5	5	231514	25879
5375	1	Q9Y6X5	Q9Y6X5	Bis(5'-adenosyl)-triphosphatase ENPP4	ENPP4	Homo sapiens	51.641	6	5	636204	300221
5376	1	A0A0B4J1V0	A0A0B4J1V0	Immunoglobulin heavy variable 3-15	IGHV3-15	Homo sapiens	12.926	4	4	4638230	6906723
5377	1	A0AVF1	A0AVF1	Intraflagellar transport protein 56	TTC26	Homo sapiens	64.177	4	4	191343	0
5378	1	A4D1U4	A4D1U4	DENN domain-containing protein 11	DENND11	Homo sapiens	51.446	4	4	264020	0

5379	1 A5PLN9	A5PLN9	Trafficking protein particle complex subunit 13	TRAPPC13	Homo sapiens	46.525	4	4	416252	205895
5380	1 A6NIH7	A6NIH7	Protein unc-119 homolog B	UNC119B	Homo sapiens	28.137	5	4	750217	388592
5381	1 A6NJG2	A6NJG2	Ankyrin repeat domain-containing protein SOWAHD	SOWAHD	Homo sapiens	33.805	4	4	0	165769
5382	1 A6NKD9	A6NKD9	Coiled-coil domain-containing protein 85C	CCDC85C	Homo sapiens	45.209	4	4	199302	0
5383	1 A9UHW6	A9UHW6	MIF4G domain-containing protein	MIF4GD	Homo sapiens	25.423	4	4	866728	0
5384	1 L0R819	L0R819	ASNSD1 upstream open reading frame protein	ASDURF	Homo sapiens	11.249	4	4	913300	225284
5385	1 O00142	O00142	Thymidine kinase 2, mitochondrial	TK2	Homo sapiens	31.004	4	4	508563	300543
5386	1 O00182	O00182	Galectin-9	LGALS9	Homo sapiens	39.518	6	4	3838423	815558
5387	1 O00187	O00187	Mannan-binding lectin serine protease 2	MASP2	Homo sapiens	75.705	4	4	282239	679157
5388	1 O00194	O00194	Ras-related protein Rab-27B	RAB27B	Homo sapiens	24.608	4	4	1580381	702939
5389	1 O00241	O00241	Signal-regulatory protein beta-1	SIRPB1	Homo sapiens	43.211	4	4	0	608647
5390	1 O00463	O00463	TNF receptor-associated factor 5	TRAF5	Homo sapiens	64.407	4	4	169845	0
5391	1 O00470	O00470	Homeobox protein Meis1	MEIS1	Homo sapiens	43.018	7	4	321922	0
5392	1 O00487	O00487	26S proteasome non-ATPase regulatory subunit 14	PSMD14	Homo sapiens	34.577	4	4	8204430	5495276
5393	1 O00541	O00541	Pescadillo homolog	PES1	Homo sapiens	68.005	4	4	1111565	314437
5394	1 O00560	O00560	Syntenin-1	SDCBP	Homo sapiens	32.445	4	4	2090996	1852934
5395	1 O00592	O00592	Podocalyxin	PODXL	Homo sapiens	58.636	4	4	1123086	1666699
5396	1 O00712	O00712	Nuclear factor 1 B-type	NFIB	Homo sapiens	47.442	4	4	1030378	55763
5397	1 O14494	O14494	Phospholipid phosphatase 1	PLPP1	Homo sapiens	32.155	4	4	1342218	86906
5398	1 O14531	O14531	Dihydropyrimidinase-related protein 4	DPYSL4	Homo sapiens	61.877	4	4	2168847	0
5399	1 O14681	O14681	Etoposide-induced protein 2.4 homolog	EI24	Homo sapiens	38.965	4	4	755866	44585
5400	1 O14732	O14732	Inositol monophosphatase 2	IMPA2	Homo sapiens	31.32	4	4	3291093	2579892
5401	1 O14734	O14734	Acyl-coenzyme A thioesterase 8	ACOT8	Homo sapiens	35.915	4	4	2052701	721773
5402	1 O14807	O14807	Ras-related protein M-Ras	MRAS	Homo sapiens	23.847	4	4	311217	0
5403	1 O14818	O14818	Proteasome subunit alpha type-7	PSMA7	Homo sapiens	27.887	9	4	6072122	5137640
5404	1 O15213	O15213	WD repeat-containing protein 46	WDR46	Homo sapiens	68.07	4	4	424559	110756
5405	1 O15260	O15260	Surfeit locus protein 4	SURF4	Homo sapiens	30.396	4	4	6465222	4397923
5406	1 O15347	O15347	High mobility group protein B3	HMGB3	Homo sapiens	22.979	4	4	4597824	4570675
5407	1 O15379	O15379	Histone deacetylase 3	HDAC3	Homo sapiens	48.85	4	4	936489	110272
5408	1 O15427	O15427	Monocarboxylate transporter 4	SLC16A3	Homo sapiens	49.47	4	4	1398219	7752214
5409	1 O15551	O15551	Claudin-3	CLDN3	Homo sapiens	23.318	5	4	2037563	103738
5410	1 O43159	O43159	Ribosomal RNA-processing protein 8	RRP8	Homo sapiens	50.715	4	4	920531	153469
5411	1 O43169	O43169	Cytochrome b5 type B	CYB5B	Homo sapiens	16.697	4	4	9644287	6270854
5412	1 O43189	O43189	PHD finger protein 1	PHF1	Homo sapiens	62.106	4	4	218710	17728
5413	1 O43240	O43240	Kallikrein-10	KLK10	Homo sapiens	30.168	4	4	0	346352
5414	1 O43291	O43291	Kunitz-type protease inhibitor 2	SPINT2	Homo sapiens	28.229	4	4	699435	710697
5415	1 O43292	O43292	Glycosylphosphatidylinositol anchor attachment 1 protein	GPAA1	Homo sapiens	67.623	4	4	1427264	1432733
5416	1 O43318	O43318	Mitogen-activated protein kinase kinase kinase 7	MAP3K7	Homo sapiens	67.196	4	4	497086	82895
5417	1 O43402	O43402	ER membrane protein complex subunit 8	EMC8	Homo sapiens	23.771	4	4	1076364	411608
5418	1 O43422	O43422	52 kDa repressor of the inhibitor of the protein kinase	THAP12	Homo sapiens	87.707	4	4	211742	37862
5419	1 O43670	O43670	BUB3-interacting and GLEBS motif-containing protein ZNF207	ZNF207	Homo sapiens	50.751	4	4	2067261	1190641
5420	1 O43768	O43768	Alpha-endosulfine	ENSA	Homo sapiens	13.39	5	4	2078825	1230213
5421	1 O43822	O43822	Cilia- and flagella-associated protein 410	CFAP410	Homo sapiens	28.341	4	4	315860	0
5422	1 O43826	O43826	Glucose-6-phosphate exchanger SLC37A4	SLC37A4	Homo sapiens	46.36	4	4	1299439	685904
5423	1 O43920	O43920	NADH dehydrogenase [ubiquinone] iron-sulfur protein 5	NDUFS5	Homo sapiens	12.517	4	4	3060961	558793
5424	1 O60308	O60308	Centrosomal protein of 104 kDa	CEP104	Homo sapiens	104.449	4	4	140738	0
5425	1 O60336	O60336	Mitogen-activated protein kinase-binding protein 1	MAPKBP1	Homo sapiens	163.819	4	4	145263	0
5426	1 O60563	O60563	Cyclin-T1	CCNT1	Homo sapiens	80.687	4	4	188546	33327

5427	1	O60565	O60565	Gremlin-1	GREM1	Homo sapiens	20.698	5	4	615633	307875
5428	1	O60678	O60678	Protein arginine N-methyltransferase 3	PRMT3	Homo sapiens	59.903	4	4	212781	113278
5429	1	O60684	O60684	Importin subunit alpha-7	KPNA6	Homo sapiens	60.03	10	4	2708360	1445478
5430	1	O60830	O60830	Mitochondrial import inner membrane translocase subunit Tim17-B	TIMM17B	Homo sapiens	18.272	4	4	396210	260111
5431	1	O60870	O60870	DNA/RNA-binding protein KIN17	KIN	Homo sapiens	45.372	4	4	199291	63966
5432	1	O60936	O60936	Nucleolar protein 3	NOL3	Homo sapiens	22.631	4	4	1280786	153027
5433	1	O75023	O75023	Leukocyte immunoglobulin-like receptor subfamily B member 5	LILRB5	Homo sapiens	64.07	4	4	642187	32117
5434	1	O75061	O75061	Putative tyrosine-protein phosphatase auxilin	DNAJC6	Homo sapiens	99.997	4	4	241419	0
5435	1	O75063	O75063	Glycosaminoglycan xylosylkinase	FAM20B	Homo sapiens	46.435	4	4	466176	54187
5436	1	O75071	O75071	EF-hand calcium-binding domain-containing protein 14	EFCAB14	Homo sapiens	55.029	4	4	205817	0
5437	1	O75127	O75127	Pentatricopeptide repeat-containing protein 1, mitochondrial	PTCD1	Homo sapiens	78.858	4	4	105925	0
5438	1	O75191	O75191	Xylulose kinase	XYLB	Homo sapiens	58.382	4	4	2327789	0
5439	1	O75298	O75298	Reticulon-2	RTN2	Homo sapiens	59.265	4	4	1438763	173236
5440	1	O75348	O75348	V-type proton ATPase subunit G 1	ATP6V1G1	Homo sapiens	13.756	4	4	1868957	3337645
5441	1	O75380	O75380	NADH dehydrogenase [ubiquinone] iron-sulfur protein 6, mitochondrial	NDUFS6	Homo sapiens	13.711	4	4	1262046	182295
5442	1	O75391	O75391	Sperm-associated antigen 7	SPAG7	Homo sapiens	26.031	4	4	603671	141008
5443	1	O75494	O75494	Serine/arginine-rich splicing factor 10	SRSF10	Homo sapiens	31.303	6	4	173117	0
5444	1	O75531	O75531	Barrier-to-autointegration factor	BANF1	Homo sapiens	10.056	4	4	434108	104471
5445	1	O75594	O75594	Peptidoglycan recognition protein 1	PGLYRP1	Homo sapiens	21.728	4	4	465009	14423468
5446	1	O75608	O75608	Acyl-protein thioesterase 1	LYPLA1	Homo sapiens	24.67	4	4	7233124	3501576
5447	1	O75610	O75610	Left-right determination factor 1	LEFTY1	Homo sapiens	40.879	9	4	154349	861773
5448	1	O75791	O75791	GRB2-related adapter protein 2	GRAP2	Homo sapiens	37.908	4	4	405579	16268
5449	1	O75818	O75818	Ribonuclease P protein subunit p40	RPP40	Homo sapiens	41.831	4	4	434404	0
5450	1	O75864	O75864	Protein phosphatase 1 regulatory subunit 37	PPP1R37	Homo sapiens	74.768	4	4	156955	0
5451	1	O75879	O75879	Glutamyl-tRNA(Gln) amidotransferase subunit B, mitochondrial	GATB	Homo sapiens	61.864	4	4	419333	0
5452	1	O75915	O75915	PRA1 family protein 3	ARL6IP5	Homo sapiens	21.615	4	4	3964537	1434090
5453	1	O76075	O76075	DNA fragmentation factor subunit beta	DFFB	Homo sapiens	39.108	4	4	231291	43548
5454	1	O76083	O76083	High affinity cGMP-specific 3',5'-cyclic phosphodiesterase 9A	PDE9A	Homo sapiens	68.492	4	4	109456	0
5455	1	O94768	O94768	Serine/threonine-protein kinase 17B	STK17B	Homo sapiens	42.345	4	4	166150	2282458
5456	1	O94788	O94788	Retinal dehydrogenase 2	ALDH1A2	Homo sapiens	56.725	4	4	252301	0
5457	1	O94842	O94842	TOX high mobility group box family member 4	TOX4	Homo sapiens	66.197	6	4	385383	36913
5458	1	O94876	O94876	Transmembrane and coiled-coil domains protein 1	TMCC1	Homo sapiens	72.084	5	4	207387	0
5459	1	O94910	O94910	Adhesion G protein-coupled receptor L1	ADGRL1	Homo sapiens	162.72	4	4	136139	0
5460	1	O94916	O94916	Nuclear factor of activated T-cells 5	NFAT5	Homo sapiens	165.765	4	4	154468	35794
5461	1	O95154	O95154	Aflatoxin B1 aldehyde reductase member 3	AKR7A3	Homo sapiens	37.205	7	4	4351754	169599
5462	1	O95159	O95159	Zinc finger protein-like 1	ZFPL1	Homo sapiens	34.113	4	4	960884	487597
5463	1	O95164	O95164	Ubiquitin-like protein 3	UBL3	Homo sapiens	13.155	4	4	803885	378211
5464	1	O95295	O95295	SNARE-associated protein Snapin	SNAPIN	Homo sapiens	14.874	4	4	1238628	655906
5465	1	O95396	O95396	Adenylyltransferase and sulfurtransferase MOCS3	MOCS3	Homo sapiens	49.668	4	4	464136	34705
5466	1	O95427	O95427	GPI ethanolamine phosphate transferase 1	PIGN	Homo sapiens	105.811	4	4	88704	95928
5467	1	O95456	O95456	Proteasome assembly chaperone 1	PSMG1	Homo sapiens	32.855	4	4	874750	291745
5468	1	O95478	O95478	Ribosome biogenesis protein NSA2 homolog	NSA2	Homo sapiens	30.066	4	4	324637	107129
5469	1	O95544	O95544	NAD kinase	NADK	Homo sapiens	49.229	4	4	298522	1688605
5470	1	O95619	O95619	YEATS domain-containing protein 4	YEATS4	Homo sapiens	26.499	4	4	201339	68905
5471	1	O95622	O95622	Adenylate cyclase type 5	ADCY5	Homo sapiens	138.909	5	4	307112	0
5472	1	O95628	O95628	CCR4-NOT transcription complex subunit 4	CNOT4	Homo sapiens	63.511	4	4	544158	0
5473	1	O95670	O95670	V-type proton ATPase subunit G 2	ATP6V1G2	Homo sapiens	13.601	4	4	623630	0
5474	1	O95674	O95674	Phosphatidate cytidylyltransferase 2	CDS2	Homo sapiens	51.422	5	4	579355	118195

5475	1 O95707	O95707	Ribonuclease P protein subunit p29	POP4	Homo sapiens	25.422	4	4	429809	53730
5476	1 O95760	O95760	Interleukin-33	IL33	Homo sapiens	30.758	4	4	858394	0
5477	1 O95881	O95881	Thioredoxin domain-containing protein 12	TXNDC12	Homo sapiens	19.205	4	4	4886525	3682063
5478	1 O95926	O95926	Pre-mRNA-splicing factor SYF2	SYF2	Homo sapiens	28.722	4	4	240458	26875
5479	1 O95976	O95976	Immunoglobulin superfamily member 6	IGSF6	Homo sapiens	27.011	4	4	93752	251889
5480	1 O95994	O95994	Anterior gradient protein 2 homolog	AGR2	Homo sapiens	19.978	5	4	61262171	801425
5481	1 P00167	P00167	Cytochrome b5	CYB5A	Homo sapiens	15.33	4	4	15491013	4055234
5482	1 P00403	P00403	Cytochrome c oxidase subunit 2	MT-CO2	Homo sapiens	25.566	4	4	17590697	744699
5483	1 P00739	P00739	Haptoglobin-related protein	HPR	Homo sapiens	39.03	4	4	1569927	2364643
5484	1 P01033	P01033	Metalloproteinase inhibitor 1	TIMP1	Homo sapiens	23.17	4	4	1098428	4011320
5485	1 P01100	P01100	Protein c-Fos	FOS	Homo sapiens	40.695	4	4	165629	517967
5486	1 P01116	P01116	GTPase KRas	KRAS	Homo sapiens	21.655	5	4	2552567	1390328
5487	1 P01282	P01282	VIP peptides	VIP	Homo sapiens	19.167	4	4	487650	0
5488	1 P01583	P01583	Interleukin-1 alpha	IL1A	Homo sapiens	30.604	4	4	0	322761
5489	1 P01584	P01584	Interleukin-1 beta	IL1B	Homo sapiens	30.748	4	4	0	4435195
5490	1 P01903	P01903	HLA class II histocompatibility antigen, DR alpha chain	HLA-DRA	Homo sapiens	28.621	5	4	713371	1273741
5491	1 P02686	P02686	Myelin basic protein	MBP	Homo sapiens	33.117	4	4	713316	552253
5492	1 P02741	P02741	C-reactive protein	CRP	Homo sapiens	25.038	4	4	17117763	28445037
5493	1 P02747	P02747	Complement C1q subcomponent subunit C	C1QC	Homo sapiens	25.773	4	4	12008925	23598270
5494	1 P03905	P03905	NADH-ubiquinone oxidoreductase chain 4	MT-ND4	Homo sapiens	51.58	4	4	3136869	580300
5495	1 P03956	P03956	Interstitial collagenase	MMP1	Homo sapiens	54.007	4	4	0	314250
5496	1 P04080	P04080	Cystatin-B	CSTB	Homo sapiens	11.139	4	4	18716344	34458460
5497	1 P04156	P04156	Major prion protein	PRNP	Homo sapiens	27.659	4	4	4370307	35351
5498	1 P04183	P04183	Thymidine kinase, cytosolic	TK1	Homo sapiens	25.468	4	4	266401	119302
5499	1 P05109	P05109	Protein S100-A8	S100A8	Homo sapiens	10.832	4	4	28606337	1185495200
5500	1 P05114	P05114	Non-histone chromosomal protein HMG-14	HMGN1	Homo sapiens	10.657	4	4	11657504	8008708
5501	1 P05162	P05162	Galectin-2	LGALS2	Homo sapiens	14.645	4	4	1211843	0
5502	1 P06312	P06312	Immunoglobulin kappa variable 4-1	IGKV4-1	Homo sapiens	13.379	4	4	4760926	11833463
5503	1 P06702	P06702	Protein S100-A9	S100A9	Homo sapiens	13.241	4	4	32005130	664524307
5504	1 P06746	P06746	DNA polymerase beta	POLB	Homo sapiens	38.176	4	4	301527	167936
5505	1 P07108	P07108	Acyl-CoA-binding protein	DBI	Homo sapiens	10.042	4	4	18749177	8608388
5506	1 P07195	P07195	L-lactate dehydrogenase B chain	LDHB	Homo sapiens	36.639	11	4	76310489	47609047
5507	1 P07199	P07199	Major centromere autoantigen B	CENPB	Homo sapiens	65.169	4	4	269431	125734
5508	1 P07998	P07998	Ribonuclease pancreatic	RNASE1	Homo sapiens	17.644	4	4	947792	46592
5509	1 P08235	P08235	Mineralocorticoid receptor	NR3C2	Homo sapiens	107.083	4	4	191112	0
5510	1 P08476	P08476	Inhibin beta A chain	INHBA	Homo sapiens	47.443	4	4	0	614129
5511	1 P08579	P08579	U2 small nuclear ribonucleoprotein B''	SNRPB2	Homo sapiens	25.487	4	4	3467447	1374470
5512	1 P08709	P08709	Coagulation factor VII	F7	Homo sapiens	51.594	4	4	72618	500349
5513	1 P08962	P08962	CD63 antigen	CD63	Homo sapiens	25.636	4	4	13352450	27608837
5514	1 P09105	P09105	Hemoglobin subunit theta-1	HBQ1	Homo sapiens	15.508	4	4	474168	504711
5515	1 P09429	P09429	High mobility group protein B1	HMGB1	Homo sapiens	24.895	11	4	7531183	12181168
5516	1 P09936	P09936	Ubiquitin carboxyl-terminal hydrolase isozyme L1	UCHL1	Homo sapiens	24.825	4	4	17642188	2063522
5517	1 P0C870	P0C870	Bifunctional peptidase and (3S)-lysyl hydroxylase JMJD7	JMJD7	Homo sapiens	35.933	4	4	218141	0
5518	1 P0DJI8	P0DJI8	Serum amyloid A-1 protein	SAA1	Homo sapiens	13.532	7	4	12990861	13029313
5519	1 P0DJI9	P0DJI9	Serum amyloid A-2 protein	SAA2	Homo sapiens	13.526	4	4	5680941	4619867
5520	1 P10153	P10153	Non-secretory ribonuclease	RNASE2	Homo sapiens	18.354	4	4	6093727	15588700
5521	1 P10606	P10606	Cytochrome c oxidase subunit 5B, mitochondrial	COX5B	Homo sapiens	13.695	4	4	40089436	6503604
5522	1 P11279	P11279	Lysosome-associated membrane glycoprotein 1	LAMP1	Homo sapiens	44.882	4	4	9352144	13222373

5523	1	P11441	P11441	Ubiquitin-like protein 4A	UBL4A	Homo sapiens	17.774	4	4	1305273	222488
5524	1	P12235	P12235	ADP/ATP translocase 1	SLC25A4	Homo sapiens	33.066	12	4	29852452	1966935
5525	1	P12645	P12645	Bone morphogenetic protein 3	BMP3	Homo sapiens	53.372	4	4	233647	0
5526	1	P12829	P12829	Myosin light chain 4	MYL4	Homo sapiens	21.563	4	4	1254138	69881
5527	1	P13051	P13051	Uracil-DNA glycosylase	UNG	Homo sapiens	34.645	4	4	714186	0
5528	1	P13224	P13224	Platelet glycoprotein Ib beta chain	GP1BB	Homo sapiens	21.719	4	4	345896	5080674
5529	1	P13688	P13688	Carcinoembryonic antigen-related cell adhesion molecule 1	CEACAM1	Homo sapiens	57.561	4	4	272233	2717118
5530	1	P13984	P13984	General transcription factor IIF subunit 2	GTF2F2	Homo sapiens	28.382	4	4	852412	280326
5531	1	P13995	P13995	Bifunctional methylenetetrahydrofolate dehydrogenase/cyclohydrolase, mitochondrial	MTHFD2	Homo sapiens	37.895	4	4	406590	283662
5532	1	P14222	P14222	Perforin-1	PRF1	Homo sapiens	61.38	4	4	0	328375
5533	1	P14555	P14555	Phospholipase A2, membrane associated	PLA2G2A	Homo sapiens	16.084	4	4	619390	0
5534	1	P14649	P14649	Myosin light chain 6B	MYL6B	Homo sapiens	22.765	4	4	917703	0
5535	1	P14672	P14672	Solute carrier family 2, facilitated glucose transporter member 4	SLC2A4	Homo sapiens	54.788	4	4	213643	0
5536	2	P14678;P63162	P14678	Small nuclear ribonucleoprotein-associated proteins B and B'	SNRPB	Homo sapiens	24.61	4	4	3397356	1251983
5537	1	P14854	P14854	Cytochrome c oxidase subunit 6B1	COX6B1	Homo sapiens	10.192	4	4	14334952	1442774
5538	1	P15151	P15151	Poliovirus receptor	PVR	Homo sapiens	45.302	4	4	628411	426240
5539	1	P15336	P15336	Cyclic AMP-dependent transcription factor ATF-2	ATF2	Homo sapiens	54.536	4	4	225856	0
5540	1	P15529	P15529	Membrane cofactor protein	CD46	Homo sapiens	43.748	4	4	2305856	990569
5541	1	P15531	P15531	Nucleoside diphosphate kinase A	NME1	Homo sapiens	17.153	4	4	15098756	7945793
5542	1	P15882	P15882	N-chimaerin	CHN1	Homo sapiens	53.173	4	4	299310	0
5543	1	P15884	P15884	Transcription factor 4	TCF4	Homo sapiens	71.308	5	4	194645	0
5544	1	P15907	P15907	Beta-galactoside alpha-2,6-sialyltransferase 1	ST6GAL1	Homo sapiens	46.603	4	4	378788	19335
5545	1	P15927	P15927	Replication protein A 32 kDa subunit	RPA2	Homo sapiens	29.248	4	4	2162727	846757
5546	1	P16035	P16035	Metalloproteinase inhibitor 2	TIMP2	Homo sapiens	24.4	4	4	1548526	3715500
5547	1	P16109	P16109	P-selectin	SELP	Homo sapiens	90.835	4	4	84431	443875
5548	1	P16401	P16401	Histone H1.5	H1-5	Homo sapiens	22.579	4	4	168615087	324571380
5549	1	P17026	P17026	Zinc finger protein 22	ZNF22	Homo sapiens	25.914	4	4	574049	243277
5550	1	P17544	P17544	Cyclic AMP-dependent transcription factor ATF-7	ATF7	Homo sapiens	51.757	4	4	165608	23613
5551	1	P17568	P17568	NADH dehydrogenase [ubiquinone] 1 beta subcomplex subunit 7	NDUFB7	Homo sapiens	16.401	4	4	1101908	308142
5552	1	P17612	P17612	cAMP-dependent protein kinase catalytic subunit alpha	PRKACA	Homo sapiens	40.59	11	4	2715902	571680
5553	1	P18085	P18085	ADP-ribosylation factor 4	ARF4	Homo sapiens	20.511	4	4	16145355	5898258
5554	1	P19075	P19075	Tetraspanin-8	TSPAN8	Homo sapiens	26.042	4	4	20552310	768029
5555	1	P19793	P19793	Retinoic acid receptor RXR-alpha	RXRA	Homo sapiens	50.812	7	4	363148	290949
5556	1	P20023	P20023	Complement receptor type 2	CR2	Homo sapiens	112.919	4	4	242942	0
5557	1	P20160	P20160	Azurocidin	AZU1	Homo sapiens	26.884	4	4	4108488	139846585
5558	1	P20292	P20292	Arachidonate 5-lipoxygenase-activating protein	ALOX5AP	Homo sapiens	18.158	4	4	1036842	9906998
5559	1	P20340	P20340	Ras-related protein Rab-6A	RAB6A	Homo sapiens	23.591	10	4	4942708	7721268
5560	1	P20645	P20645	Cation-dependent mannose-6-phosphate receptor	M6PR	Homo sapiens	30.993	4	4	1603824	897731
5561	1	P20962	P20962	Parathymosin	PTMS	Homo sapiens	11.528	4	4	10930836	2406229
5562	1	P22083	P22083	Alpha-(1,3)-fucosyltransferase 4	FUT4	Homo sapiens	59.084	4	4	391408	0
5563	1	P22303	P22303	Acetylcholinesterase	ACHE	Homo sapiens	67.796	4	4	7814650	0
5564	1	P22670	P22670	MHC class II regulatory factor RFX1	RFX1	Homo sapiens	104.761	4	4	552249	106534
5565	1	P23327	P23327	Sarcoplasmic reticulum histidine-rich calcium-binding protein	HRC	Homo sapiens	80.243	4	4	201950	0
5566	1	P23443	P23443	Ribosomal protein S6 kinase beta-1	RPS6KB1	Homo sapiens	59.14	4	4	894325	456328
5567	1	P23610	P23610	40-kDa huntingtin-associated protein	F8A3	Homo sapiens	39.101	4	4	538633	220548
5568	1	P24588	P24588	A-kinase anchor protein 5	AKAP5	Homo sapiens	47.089	4	4	165028	0
5569	1	P26447	P26447	Protein S100-A4	S100A4	Homo sapiens	11.728	4	4	18251720	15227065

5570	1	P26885	P26885	Peptidyl-prolyl cis-trans isomerase FKBP2	FKBP2	Homo sapiens	15.647	4	4	15004951	5688562
5571	1	P27658	P27658	Collagen alpha-1(VIII) chain	COL8A1	Homo sapiens	73.363	4	4	934361	386203
5572	1	P28845	P28845	11-beta-hydroxysteroid dehydrogenase 1	HSD11B1	Homo sapiens	32.401	4	4	114450	226584
5573	1	P29084	P29084	Transcription initiation factor IIE subunit beta	GTF2E2	Homo sapiens	33.043	4	4	346828	156723
5574	1	P29972	P29972	Aquaporin-1	AQP1	Homo sapiens	28.526	4	4	1701868	207277
5575	1	P31350	P31350	Ribonucleoside-diphosphate reductase subunit M2	RRM2	Homo sapiens	44.878	4	4	629117	275743
5576	1	P31949	P31949	Protein S100-A11	S100A11	Homo sapiens	11.742	4	4	29031605	49599312
5577	1	P32019	P32019	Type II inositol 1,4,5-trisphosphate 5-phosphatase	INPP5B	Homo sapiens	112.853	4	4	141200	416669
5578	1	P32320	P32320	Cytidine deaminase	CDA	Homo sapiens	16.182	4	4	148209	3386606
5579	1	P32321	P32321	Deoxycytidylate deaminase	DCTD	Homo sapiens	20.014	4	4	159872	359517
5580	1	P35080	P35080	Profilin-2	PFN2	Homo sapiens	15.046	4	4	13225551	688415
5581	1	P35219	P35219	Carbonic anhydrase-related protein	CA8	Homo sapiens	32.971	4	4	389735	0
5582	1	P35542	P35542	Serum amyloid A-4 protein	SAA4	Homo sapiens	14.747	4	4	9541064	23424427
5583	1	P35637	P35637	RNA-binding protein FUS	FUS	Homo sapiens	53.425	6	4	4117374	1129790
5584	1	P36639	P36639	Oxidized purine nucleoside triphosphate hydrolase	NUDT1	Homo sapiens	17.951	4	4	212562	168247
5585	1	P36897	P36897	TGF-beta receptor type-1	TGFBR1	Homo sapiens	55.961	5	4	281598	280490
5586	1	P38159	P38159	RNA-binding motif protein, X chromosome	RBMX	Homo sapiens	42.33	17	4	1404261	393236
5587	1	P38571	P38571	Lysosomal acid lipase/cholesteryl ester hydrolase	LIPA	Homo sapiens	45.419	4	4	3113893	514876
5588	1	P39687	P39687	Acidic leucine-rich nuclear phosphoprotein 32 family member A	ANP32A	Homo sapiens	28.585	6	4	2098174	1524679
5589	1	P40855	P40855	Peroxisomal biogenesis factor 19	PEX19	Homo sapiens	32.808	4	4	1356680	312888
5590	1	P40938	P40938	Replication factor C subunit 3	RFC3	Homo sapiens	40.555	4	4	835147	565538
5591	1	P41212	P41212	Transcription factor ETV6	ETV6	Homo sapiens	53.003	4	4	358554	407877
5592	1	P41221	P41221	Protein Wnt-5a	WNT5A	Homo sapiens	42.34	6	4	0	699217
5593	1	P41235	P41235	Hepatocyte nuclear factor 4-alpha	HNF4A	Homo sapiens	52.784	4	4	561091	121695
5594	1	P42025	P42025	Beta-centractin	ACTR1B	Homo sapiens	42.29	4	4	2500262	1213839
5595	1	P42229	P42229	Signal transducer and activator of transcription 5A	STAT5A	Homo sapiens	90.652	4	4	440479	275560
5596	1	P42338	P42338	Phosphatidylinositol 4,5-bisphosphate 3-kinase catalytic subunit beta isoform	PIK3CB	Homo sapiens	122.764	4	4	255276	579810
5597	1	P42695	P42695	Condensin-2 complex subunit D3	NCAPD3	Homo sapiens	168.893	4	4	65640	0
5598	1	P42785	P42785	Lysosomal Pro-X carboxypeptidase	PRCP	Homo sapiens	55.799	4	4	1285439	1123358
5599	1	P43403	P43403	Tyrosine-protein kinase ZAP-70	ZAP70	Homo sapiens	69.875	4	4	75257	74210
5600	1	P46020	P46020	Phosphorylase b kinase regulatory subunit alpha, skeletal muscle isoform	PHKA1	Homo sapiens	137.314	4	4	114273	36789
5601	1	P46527	P46527	Cyclin-dependent kinase inhibitor 1B	CDKN1B	Homo sapiens	22.072	4	4	256068	146644
5602	1	P46776	P46776	60S ribosomal protein L27a	RPL27A	Homo sapiens	16.561	4	4	53785169	24887624
5603	1	P46783	P46783	40S ribosomal protein S10	RPS10	Homo sapiens	18.896	7	4	5216059	1658671
5604	1	P49356	P49356	Protein farnesyltransferase subunit beta	FNTB	Homo sapiens	48.774	4	4	1330819	576145
5605	1	P49366	P49366	Deoxyhypusine synthase	DHPS	Homo sapiens	40.969	4	4	532098	60047
5606	1	P49619	P49619	Diacylglycerol kinase gamma	DGKG	Homo sapiens	89.125	4	4	154876	22943
5607	1	P49795	P49795	Regulator of G-protein signaling 19	RGS19	Homo sapiens	24.637	4	4	15928	511452
5608	1	P49863	P49863	Granzyme K	GZMK	Homo sapiens	28.882	4	4	704564	153764
5609	1	P49908	P49908	Selenoprotein P	SELENOP	Homo sapiens	42.886	4	4	1696723	2372236
5610	1	P50135	P50135	Histamine N-methyltransferase	HNMT	Homo sapiens	33.295	4	4	3126571	72232
5611	1	P50548	P50548	ETS domain-containing transcription factor ERF	ERF	Homo sapiens	58.704	4	4	200499	0
5612	1	P50583	P50583	Bis(5'-nucleosyl)-tetraphosphatase [asymmetrical]	NUDT2	Homo sapiens	16.83	4	4	704182	461414
5613	1	P51153	P51153	Ras-related protein Rab-13	RAB13	Homo sapiens	22.773	4	4	3122942	3309750
5614	1	P51674	P51674	Neuronal membrane glycoprotein M6-a	GPM6A	Homo sapiens	31.211	4	4	1428426	0
5615	1	P51946	P51946	Cyclin-H	CCNH	Homo sapiens	37.643	4	4	126456	32948
5616	1	P51956	P51956	Serine/threonine-protein kinase Nek3	NEK3	Homo sapiens	57.704	4	4	353305	0

5617	1	P52823	P52823	Stanniocalcin-1	STC1	Homo sapiens	27.621	4	4	0	535405
5618	1	P53611	P53611	Geranylgeranyl transferase type-2 subunit beta	RABGGTB	Homo sapiens	36.925	4	4	623133	519903
5619	1	P53680	P53680	AP-2 complex subunit sigma	AP2S1	Homo sapiens	17.017	4	4	2900254	1280003
5620	1	P54105	P54105	Methylosome subunit pICln	CLNS1A	Homo sapiens	26.216	4	4	1920804	998366
5621	1	P54108	P54108	Cysteine-rich secretory protein 3	CRISP3	Homo sapiens	27.631	4	4	104308	2350763
5622	1	P54252	P54252	Ataxin-3	ATXN3	Homo sapiens	41.251	4	4	1764648	617621
5623	1	P54284	P54284	Voltage-dependent L-type calcium channel subunit beta-3	CACNB3	Homo sapiens	54.532	4	4	280517	0
5624	1	P54646	P54646	5'-AMP-activated protein kinase catalytic subunit alpha-2	PRKAA2	Homo sapiens	62.323	4	4	957565	0
5625	1	P55211	P55211	Caspase-9	CASP9	Homo sapiens	46.28	4	4	88707	68559
5626	1	P55345	P55345	Protein arginine N-methyltransferase 2	PRMT2	Homo sapiens	49.041	4	4	570438	315755
5627	1	P55769	P55769	NHP2-like protein 1	SNU13	Homo sapiens	14.173	4	4	7747349	3574911
5628	1	P55899	P55899	IgG receptor FcRn large subunit p51	FCGRT	Homo sapiens	39.743	4	4	1823266	305784
5629	1	P55957	P55957	BH3-interacting domain death agonist	BID	Homo sapiens	21.992	4	4	2637601	4049543
5630	1	P56159	P56159	GDNF family receptor alpha-1	GFRA1	Homo sapiens	51.457	4	4	661891	0
5631	1	P56537	P56537	Eukaryotic translation initiation factor 6	EIF6	Homo sapiens	26.599	4	4	7440525	3001122
5632	1	P57076	P57076	Cilia- and flagella-associated protein 298	CFAP298	Homo sapiens	33.222	4	4	594987	120254
5633	1	P57081	P57081	tRNA (guanine-N(7)-)-methyltransferase non-catalytic subunit WDR4	WDR4	Homo sapiens	45.49	4	4	141115	190556
5634	1	P57721	P57721	Poly(rC)-binding protein 3	PCBP3	Homo sapiens	39.464	4	4	892127	173760
5635	1	P58004	P58004	Sestrin-2	SESN2	Homo sapiens	54.495	4	4	23742	126433
5636	1	P61006	P61006	Ras-related protein Rab-8A	RAB8A	Homo sapiens	23.664	5	4	3639646	2929411
5637	1	P61009	P61009	Signal peptidase complex subunit 3	SPCS3	Homo sapiens	20.315	4	4	7718724	5067814
5638	1	P61457	P61457	Pterin-4-alpha-carbinolamine dehydratase	PCBD1	Homo sapiens	11.998	4	4	13394248	3329690
5639	1	P61586	P61586	Transforming protein RhoA	RHOA	Homo sapiens	21.767	10	4	5251934	8306974
5640	1	P62136	P62136	Serine/threonine-protein phosphatase PP1-alpha catalytic subunit	PPP1CA	Homo sapiens	37.511	5	4	6157092	6478055
5641	1	P62140	P62140	Serine/threonine-protein phosphatase PP1-beta catalytic subunit	PPP1CB	Homo sapiens	37.184	16	4	31327351	8779794
5642	1	P62266	P62266	40S ribosomal protein S23	RPS23	Homo sapiens	15.807	4	4	16121563	5663250
5643	1	P62314	P62314	Small nuclear ribonucleoprotein Sm D1	SNRPD1	Homo sapiens	13.28	4	4	7822767	3869033
5644	1	P62487	P62487	DNA-directed RNA polymerase II subunit RPB7	POLR2G	Homo sapiens	19.294	4	4	1117950	478794
5645	1	P62873	P62873	Guanine nucleotide-binding protein G(I)/G(S)/G(T) subunit beta-1	GNB1	Homo sapiens	37.377	5	4	6152466	3033782
5646	1	P62899	P62899	60S ribosomal protein L31	RPL31	Homo sapiens	14.463	4	4	14753105	3046854
5647	1	P63096	P63096	Guanine nucleotide-binding protein G(i) subunit alpha-1	GNAI1	Homo sapiens	40.358	4	4	1136346	181658
5648	1	P63220	P63220	40S ribosomal protein S21	RPS21	Homo sapiens	9.109	4	4	15755370	5473844
5649	1	P78345	P78345	Ribonuclease P protein subunit p38	RPP38	Homo sapiens	31.835	4	4	726436	134570
5650	1	P78346	P78346	Ribonuclease P protein subunit p30	RPP30	Homo sapiens	29.322	4	4	525597	218867
5651	1	P78380	P78380	Oxidized low-density lipoprotein receptor 1	OLR1	Homo sapiens	30.959	4	4	0	1195731
5652	1	P78549	P78549	Endonuclease III-like protein 1	NTHL1	Homo sapiens	34.39	4	4	407202	0
5653	1	P78560	P78560	Death domain-containing protein CRADD	CRADD	Homo sapiens	22.745	4	4	736220	144057
5654	1	P81274	P81274	G-protein-signaling modulator 2	GPSM2	Homo sapiens	76.663	4	4	117720	0
5655	1	P82664	P82664	28S ribosomal protein S10, mitochondrial	MRPS10	Homo sapiens	22.999	4	4	746992	437578
5656	1	P82979	P82979	SAP domain-containing ribonucleoprotein	SARNP	Homo sapiens	23.672	4	4	4866259	2253189
5657	1	P84085	P84085	ADP-ribosylation factor 5	ARF5	Homo sapiens	20.529	10	4	7059137	4154164
5658	1	P84098	P84098	60S ribosomal protein L19	RPL19	Homo sapiens	23.466	4	4	2505137	542344
5659	1	P84103	P84103	Serine/arginine-rich splicing factor 3	SRSF3	Homo sapiens	19.329	4	4	17569752	7154143
5660	1	Q00577	Q00577	Transcriptional activator protein Pur-alpha	PURA	Homo sapiens	34.908	4	4	5456834	986361
5661	1	Q00765	Q00765	Receptor expression-enhancing protein 5	REEP5	Homo sapiens	21.494	4	4	9982492	5555487
5662	1	Q00978	Q00978	Interferon regulatory factor 9	IRF9	Homo sapiens	43.695	4	4	308265	223684
5663	1	Q01415	Q01415	N-acetylgalactosamine kinase	GALK2	Homo sapiens	50.38	4	4	646574	282128
5664	1	Q01973	Q01973	Inactive tyrosine-protein kinase transmembrane receptor ROR1	ROR1	Homo sapiens	104.282	4	4	432950	0

5665	1	Q02447	Q02447	Transcription factor Sp3	SP3	Homo sapiens	81.927	4	4	400008	358396
5666	1	Q03468	Q03468	DNA excision repair protein ERCC-6	ERCC6	Homo sapiens	168.418	4	4	162073	80336
5667	1	Q06520	Q06520	Sulfotransferase 2A1	SULT2A1	Homo sapiens	33.78	4	4	0	630681
5668	1	Q06587	Q06587	E3 ubiquitin-protein ligase RING1	RING1	Homo sapiens	42.429	4	4	786775	24660
5669	1	Q07092	Q07092	Collagen alpha-1(XVI) chain	COL16A1	Homo sapiens	157.755	4	4	484883	0
5670	1	Q08117	Q08117	TLE family member 5	TLE5	Homo sapiens	21.969	4	4	1032220	34291
5671	1	Q08357	Q08357	Sodium-dependent phosphate transporter 2	SLC20A2	Homo sapiens	70.391	4	4	1611294	98766
5672	1	Q08477	Q08477	Cytochrome P450 4F3	CYP4F3	Homo sapiens	59.845	16	4	0	6265667
5673	1	Q08830	Q08830	Fibrinogen-like protein 1	FGL1	Homo sapiens	36.38	4	4	113198	375728
5674	1	Q12873	Q12873	Chromodomain-helicase-DNA-binding protein 3	CHD3	Homo sapiens	226.596	5	4	406283	0
5675	1	Q12968	Q12968	Nuclear factor of activated T-cells, cytoplasmic 3	NFATC3	Homo sapiens	115.594	4	4	44498	128998
5676	1	Q13084	Q13084	39S ribosomal protein L28, mitochondrial	MRPL28	Homo sapiens	30.157	4	4	1087148	241558
5677	1	Q13103	Q13103	Secreted phosphoprotein 24	SPP2	Homo sapiens	24.337	4	4	134585	1493488
5678	1	Q13155	Q13155	Aminoacyl tRNA synthase complex-interacting multifunctional protein 2	AIMP2	Homo sapiens	35.348	4	4	1374774	555515
5679	1	Q13163	Q13163	Dual specificity mitogen-activated protein kinase kinase 5	MAP2K5	Homo sapiens	50.112	4	4	206957	291520
5680	1	Q13219	Q13219	Pappalysin-1	PAPPA	Homo sapiens	180.977	4	4	269051	75269
5681	1	Q13322	Q13322	Growth factor receptor-bound protein 10	GRB10	Homo sapiens	67.232	4	4	239895	117134
5682	1	Q13370	Q13370	cGMP-inhibited 3',5'-cyclic phosphodiesterase 3B	PDE3B	Homo sapiens	124.335	4	4	177752	0
5683	1	Q13404	Q13404	Ubiquitin-conjugating enzyme E2 variant 1	UBE2V1	Homo sapiens	16.495	7	4	14343627	5171904
5684	1	Q13405	Q13405	39S ribosomal protein L49, mitochondrial	MRPL49	Homo sapiens	19.196	4	4	1012446	568630
5685	1	Q13470	Q13470	Non-receptor tyrosine-protein kinase TNK1	TNK1	Homo sapiens	72.468	4	4	402973	0
5686	1	Q13478	Q13478	Interleukin-18 receptor 1	IL18R1	Homo sapiens	62.303	4	4	62421	573151
5687	1	Q13530	Q13530	Serine incorporator 3	SERINC3	Homo sapiens	52.58	4	4	147987	252466
5688	1	Q13595	Q13595	Transformer-2 protein homolog alpha	TRA2A	Homo sapiens	32.688	4	4	2269759	609050
5689	1	Q13601	Q13601	KRR1 small subunit processome component homolog	KRR1	Homo sapiens	43.666	4	4	796145	266574
5690	1	Q14119	Q14119	Vascular endothelial zinc finger 1	VEZF1	Homo sapiens	56.931	4	4	127004	24222
5691	1	Q14147	Q14147	Probable ATP-dependent RNA helicase DHX34	DHX34	Homo sapiens	128.119	4	4	186427	17986
5692	1	Q14168	Q14168	MAGUK p55 subfamily member 2	MPP2	Homo sapiens	64.58	4	4	220631	0
5693	1	Q14191	Q14191	Bifunctional 3'-5' exonuclease/ATP-dependent helicase WRN	WRN	Homo sapiens	162.466	4	4	157713	22405
5694	1	Q14249	Q14249	Endonuclease G, mitochondrial	ENDOG	Homo sapiens	32.619	4	4	628274	101294
5695	1	Q14534	Q14534	Squalene monooxygenase	SQLE	Homo sapiens	63.922	4	4	141638	124354
5696	1	Q14728	Q14728	Major facilitator superfamily domain-containing protein 10	MFSD10	Homo sapiens	48.339	4	4	1111239	1732463
5697	1	Q14938	Q14938	Nuclear factor 1 X-type	NFIX	Homo sapiens	55.097	4	4	744914	0
5698	1	Q14999	Q14999	Cullin-7	CUL7	Homo sapiens	191.163	4	4	140489	17650
5699	1	Q15035	Q15035	Translocating chain-associated membrane protein 2	TRAM2	Homo sapiens	43.326	4	4	143721	221620
5700	1	Q15043	Q15043	Metal cation symporter ZIP14	SLC39A14	Homo sapiens	54.214	4	4	922296	213229
5701	1	Q15048	Q15048	Leucine-rich repeat-containing protein 14	LRRC14	Homo sapiens	54.513	4	4	147276	23703
5702	1	Q15054	Q15054	DNA polymerase delta subunit 3	POLD3	Homo sapiens	51.399	4	4	344788	14623
5703	1	Q15070	Q15070	Mitochondrial inner membrane protein OXA1L	OXA1L	Homo sapiens	48.549	4	4	927702	0
5704	1	Q15170	Q15170	Transcription elongation factor A protein-like 1	TCEAL1	Homo sapiens	18.638	4	4	1179491	0
5705	1	Q15223	Q15223	Nectin-1	NECTIN1	Homo sapiens	57.159	4	4	299924	0
5706	1	Q15326	Q15326	Zinc finger MYND domain-containing protein 11	ZMYND11	Homo sapiens	70.963	4	4	406014	0
5707	1	Q15370	Q15370	Elongin-B	ELOB	Homo sapiens	13.132	4	4	3936609	3034320
5708	1	Q15392	Q15392	Delta(24)-sterol reductase	DHCR24	Homo sapiens	60.102	4	4	469308	1524438
5709	1	Q15485	Q15485	Ficolin-2	FCN2	Homo sapiens	34.002	4	4	0	514090
5710	1	Q15527	Q15527	Surfeit locus protein 2	SURF2	Homo sapiens	29.617	4	4	193410	0
5711	1	Q15542	Q15542	Transcription initiation factor TFIID subunit 5	TAF5	Homo sapiens	86.832	4	4	357076	0
5712	1	Q15555	Q15555	Microtubule-associated protein RP/EB family member 2	MAPRE2	Homo sapiens	37.032	5	4	1670304	668549

5713	1	Q15700	Q15700	Disks large homolog 2	DLG2	Homo sapiens	97.552	4	4	124625	0
5714	1	Q15773	Q15773	Myeloid leukemia factor 2	MLF2	Homo sapiens	28.148	4	4	1386097	240887
5715	1	Q16540	Q16540	39S ribosomal protein L23, mitochondrial	MRPL23	Homo sapiens	17.78	4	4	1089275	123854
5716	1	Q16548	Q16548	Bcl-2-related protein A1	BCL2A1	Homo sapiens	20.132	4	4	0	889822
5717	1	Q16623	Q16623	Syntaxin-1A	STX1A	Homo sapiens	33.021	4	4	620653	0
5718	1	Q16654	Q16654	[Pyruvate dehydrogenase (acetyl-transferring)] kinase isozyme 4, mitochondrial	PDK4	Homo sapiens	46.47	4	4	218614	0
5719	1	Q16825	Q16825	Tyrosine-protein phosphatase non-receptor type 21	PTPN21	Homo sapiens	133.283	4	4	124128	0
5720	1	Q2TAA5	Q2TAA5	GDP-Man:Man(3)GlcNAc(2)-PP-Dol alpha-1,2-mannosyltransferase	ALG11	Homo sapiens	55.651	4	4	565000	191372
5721	1	Q2TB90	Q2TB90	Hexokinase HKDC1	HKDC1	Homo sapiens	102.545	4	4	0	257284
5722	1	Q3B726	Q3B726	DNA-directed RNA polymerase I subunit RPA43	POLR1F	Homo sapiens	37.429	4	4	136746	0
5723	1	Q3KR16	Q3KR16	Pleckstrin homology domain-containing family G member 6	PLEKHG6	Homo sapiens	88.963	4	4	308190	0
5724	1	Q3MIX3	Q3MIX3	Uncharacterized aarF domain-containing protein kinase 5	ADCK5	Homo sapiens	65.826	4	4	331674	17699
5725	1	Q3MJ16	Q3MJ16	Cytosolic phospholipase A2 epsilon	PLA2G4E	Homo sapiens	99.193	4	4	0	299250
5726	1	Q4G0X4	Q4G0X4	BTB/POZ domain-containing protein KCTD21	KCTD21	Homo sapiens	29.644	4	4	365243	76022
5727	1	Q4VC31	Q4VC31	Protein MIX23	MIX23	Homo sapiens	16.619	4	4	2665325	3230407
5728	1	Q52LD8	Q52LD8	Raftlin-2	RFTN2	Homo sapiens	55.921	4	4	981927	0
5729	1	Q53H47	Q53H47	Histone-lysine N-methyltransferase SETMAR	SETMAR	Homo sapiens	78.033	4	4	437398	0
5730	1	Q53HC0	Q53HC0	Coiled-coil domain-containing protein 92	CCDC92	Homo sapiens	36.961	4	4	190853	0
5731	1	Q53TN4	Q53TN4	Plasma membrane ascorbate-dependent reductase CYBRD1	CYBRD1	Homo sapiens	31.639	4	4	4144663	1792553
5732	1	Q56P03	Q56P03	E2F-associated phosphoprotein	EAPP	Homo sapiens	32.76	4	4	278921	47093
5733	1	Q5EBL8	Q5EBL8	PDZ domain-containing protein 11	PDZD11	Homo sapiens	16.13	4	4	1274091	0
5734	1	Q5F1R6	Q5F1R6	DnaJ homolog subfamily C member 21	DNAJC21	Homo sapiens	62.03	4	4	773769	57584
5735	1	Q5J8M3	Q5J8M3	ER membrane protein complex subunit 4	EMC4	Homo sapiens	20.086	4	4	789115	188867
5736	1	Q5JS37	Q5JS37	NHL repeat-containing protein 3	NHLRC3	Homo sapiens	38.284	4	4	451579	447012
5737	1	Q5JTJ3	Q5JTJ3	Cytochrome c oxidase assembly factor 6 homolog	COA6	Homo sapiens	14.117	4	4	2682863	752329
5738	1	Q5JVS0	Q5JVS0	Intracellular hyaluronan-binding protein 4	HABP4	Homo sapiens	45.785	4	4	190006	0
5739	1	Q5PRF9	Q5PRF9	Protein Smaug homolog 2	SAMD4B	Homo sapiens	75.483	4	4	198731	61932
5740	1	Q5QGZ9	Q5QGZ9	C-type lectin domain family 12 member A	CLEC12A	Homo sapiens	30.76	4	4	0	1177952
5741	1	Q5RI15	Q5RI15	Cytochrome c oxidase assembly protein COX20, mitochondrial	COX20	Homo sapiens	13.29	4	4	1741395	355012
5742	1	Q5SGD2	Q5SGD2	Protein phosphatase 1L	PPM1L	Homo sapiens	41.054	4	4	579347	0
5743	1	Q5SNT2	Q5SNT2	Transmembrane protein 201	TMEM201	Homo sapiens	72.236	4	4	384572	64175
5744	1	Q5T0D9	Q5T0D9	Tumor protein p63-regulated gene 1-like protein	TPRG1L	Homo sapiens	30.211	4	4	542514	180600
5745	1	Q5T1V6	Q5T1V6	Probable ATP-dependent RNA helicase DDX59	DDX59	Homo sapiens	68.813	4	4	234166	60553
5746	1	Q5T9C2	Q5T9C2	Early estrogen-induced gene 1 protein	EEIG1	Homo sapiens	41.784	4	4	83117	22806
5747	1	Q5TEA3	Q5TEA3	Dynein axonemal assembly factor 9	DNAAF9	Homo sapiens	132.287	4	4	403356	0
5748	1	Q5VT97	Q5VT97	Rho GTPase-activating protein SYDE2	SYDE2	Homo sapiens	133.233	4	4	159676	0
5749	1	Q5VWC8	Q5VWC8	Very-long-chain (3R)-3-hydroxyacyl-CoA dehydratase 4	HACD4	Homo sapiens	27.518	4	4	291796	428916
5750	1	Q63HM9	Q63HM9	PI-PLC X domain-containing protein 3	PLCXD3	Homo sapiens	36.312	4	4	179895	0
5751	1	Q659C4	Q659C4	La-related protein 1B	LARP1B	Homo sapiens	105.321	4	4	225114	57785
5752	1	Q66PJ3	Q66PJ3	ADP-ribosylation factor-like protein 6-interacting protein 4	ARL6IP4	Homo sapiens	26.372	4	4	1289932	378547
5753	1	Q68CP4	Q68CP4	Heparan-alpha-glucosaminide N-acetyltransferase	HGSNAT	Homo sapiens	73.294	4	4	87643	396099
5754	1	Q68CR1	Q68CR1	Protein sel-1 homolog 3	SEL1L3	Homo sapiens	128.567	4	4	214555	18299
5755	1	Q68CZ6	Q68CZ6	HAUS augmin-like complex subunit 3	HAUS3	Homo sapiens	69.651	4	4	28573	104655
5756	1	Q68D91	Q68D91	Acyl-coenzyme A thioesterase MBLAC2	MBLAC2	Homo sapiens	31.372	4	4	355154	20252
5757	1	Q68DD2	Q68DD2	Cytosolic phospholipase A2 zeta	PLA2G4F	Homo sapiens	95.083	4	4	3670064	0
5758	1	Q68DH5	Q68DH5	G-protein coupled receptor-associated protein LMBRD2	LMBRD2	Homo sapiens	81.174	4	4	711761	185363
5759	1	Q6DD87	Q6DD87	Zinc finger protein 787	ZNF787	Homo sapiens	40.427	4	4	429996	157798

5760	1	Q6DHV7	Q6DHV7	Adenosine deaminase-like protein	ADAL	Homo sapiens	40.264	4	4	219095	16743
5761	1	Q6N069	Q6N069	N-alpha-acetyltransferase 16, NatA auxiliary subunit	NAA16	Homo sapiens	101.465	4	4	594771	253239
5762	1	Q6NUQ1	Q6NUQ1	RAD50-interacting protein 1	RINT1	Homo sapiens	90.636	4	4	341010	161329
5763	1	Q6NXT6	Q6NXT6	Transmembrane anterior posterior transformation protein 1 homolog	TAPT1	Homo sapiens	64.264	4	4	448352	416770
5764	1	Q6NYC1	Q6NYC1	Bifunctional arginine demethylase and lysyl-hydroxylase JMJD6	JMJD6	Homo sapiens	46.462	4	4	706627	211361
5765	1	Q6P087	Q6P087	Mitochondrial mRNA pseudouridine synthase RPUSD3	RPUSD3	Homo sapiens	38.46	4	4	268906	0
5766	1	Q6P1L8	Q6P1L8	39S ribosomal protein L14, mitochondrial	MRPL14	Homo sapiens	15.949	4	4	887184	99901
5767	1	Q6P4A7	Q6P4A7	Sideroflexin-4	SFXN4	Homo sapiens	37.999	4	4	860602	312370
5768	1	Q6P587	Q6P587	Acylpyruvase FAHD1, mitochondrial	FAHD1	Homo sapiens	24.842	4	4	2336810	390449
5769	1	Q6P6C2	Q6P6C2	RNA demethylase ALKBH5	ALKBH5	Homo sapiens	44.255	4	4	351154	0
5770	1	Q6PFW1	Q6PFW1	Inositol hexakisphosphate and diphosphoinositol-pentakisphosphate kinase 1	PPIP5K1	Homo sapiens	159.526	4	4	402738	0
5771	1	Q6PHR2	Q6PHR2	Serine/threonine-protein kinase ULK3	ULK3	Homo sapiens	53.444	4	4	228682	0
5772	1	Q6PID6	Q6PID6	Tetratricopeptide repeat protein 33	TTC33	Homo sapiens	29.411	4	4	320615	0
5773	1	Q6PJF5	Q6PJF5	Inactive rhomboid protein 2	RHBDF2	Homo sapiens	96.686	4	4	0	405661
5774	1	Q6RW13	Q6RW13	Type-1 angiotensin II receptor-associated protein	AGTRAP	Homo sapiens	17.42	4	4	292812	2465119
5775	1	Q6UUV9	Q6UUV9	CREB-regulated transcription coactivator 1	CRTC1	Homo sapiens	67.299	4	4	350553	0
5776	1	Q6UVY6	Q6UVY6	DBH-like monooxygenase protein 1	MOXD1	Homo sapiens	69.652	4	4	626897	0
5777	1	Q6UW63	Q6UW63	Protein O-glucosyltransferase 2	POGLUT2	Homo sapiens	58.041	4	4	166017	146965
5778	1	Q6UWJ1	Q6UWJ1	Transmembrane and coiled-coil domain-containing protein 3	TMCO3	Homo sapiens	75.598	4	4	140767	88920
5779	1	Q6UXY8	Q6UXY8	Transmembrane channel-like protein 5	TMC5	Homo sapiens	114.799	4	4	0	307290
5780	1	Q6VMQ6	Q6VMQ6	Activating transcription factor 7-interacting protein 1	ATF7IP	Homo sapiens	136.396	4	4	153511	11606
5781	1	Q6ZNW5	Q6ZNW5	GDP-D-glucose phosphorylase 1	GDPGP1	Homo sapiens	42.363	4	4	402204	159277
5782	1	Q6ZQN7	Q6ZQN7	Solute carrier organic anion transporter family member 4C1	SLCO4C1	Homo sapiens	78.95	4	4	0	902767
5783	1	Q6ZRY4	Q6ZRY4	RNA-binding protein with multiple splicing 2	RBPMS2	Homo sapiens	22.497	4	4	742813	0
5784	1	Q6ZS72	Q6ZS72	Protein PEAK3	PEAK3	Homo sapiens	50.511	4	4	0	985351
5785	1	Q6ZTN6	Q6ZTN6	Ankyrin repeat domain-containing protein 13D	ANKRD13D	Homo sapiens	68.173	4	4	269784	272343
5786	1	Q6ZWJ1	Q6ZWJ1	Syntaxin-binding protein 4	STXBP4	Homo sapiens	61.662	4	4	245506	0
5787	1	Q70EL1	Q70EL1	Inactive ubiquitin carboxyl-terminal hydrolase 54	USP54	Homo sapiens	187.392	4	4	285210	0
5788	1	Q712K3	Q712K3	Ubiquitin-conjugating enzyme E2 R2	UBE2R2	Homo sapiens	27.164	4	4	1080958	543803
5789	1	Q76I76	Q76I76	Protein phosphatase Slingshot homolog 2	SSH2	Homo sapiens	158.217	4	4	25348	270079
5790	1	Q7KZN9	Q7KZN9	Cytochrome c oxidase assembly protein COX15 homolog	COX15	Homo sapiens	46.032	4	4	1132842	181386
5791	1	Q7L311	Q7L311	Armadillo repeat-containing X-linked protein 2	ARMCX2	Homo sapiens	65.683	4	4	627823	0
5792	1	Q7L4I2	Q7L4I2	Arginine/serine-rich coiled-coil protein 2	RSRC2	Homo sapiens	50.56	4	4	704091	154337
5793	1	Q7L5A8	Q7L5A8	Fatty acid 2-hydroxylase	FA2H	Homo sapiens	42.791	4	4	327226	0
5794	1	Q7L5L3	Q7L5L3	Lysophospholipase D GDPD3	GDPD3	Homo sapiens	36.596	4	4	341909	189954
5795	1	Q7L8J4	Q7L8J4	SH3 domain-binding protein 5-like	SH3BP5L	Homo sapiens	43.5	4	4	244002	26137
5796	1	Q7L8W6	Q7L8W6	Diphthine--ammonia ligase	DPH6	Homo sapiens	30.309	4	4	238832	99350
5797	2	Q7L9L4;Q9H8S9	Q7L9L4	MOB kinase activator 1B	MOB1B	Homo sapiens	25.087	4	4	8025116	5589122
5798	1	Q7Z449	Q7Z449	Cytochrome P450 2U1	CYP2U1	Homo sapiens	61.988	4	4	312151	0
5799	1	Q7Z4H7	Q7Z4H7	HAUS augmin-like complex subunit 6	HAUS6	Homo sapiens	108.623	4	4	225488	0
5800	1	Q7Z591	Q7Z591	Microtubule organization protein AKNA	AKNA	Homo sapiens	155.141	4	4	19689	79680
5801	1	Q7Z5G4	Q7Z5G4	Golgin subfamily A member 7	GOLGA7	Homo sapiens	15.823	4	4	1030373	1186351
5802	1	Q7Z6G3	Q7Z6G3	N-terminal EF-hand calcium-binding protein 2	NECAB2	Homo sapiens	43.194	4	4	213783	0
5803	1	Q7Z7A1	Q7Z7A1	Centriolin	CNTRL	Homo sapiens	268.89	4	4	0	178518
5804	1	Q7Z7F7	Q7Z7F7	39S ribosomal protein L55, mitochondrial	MRPL55	Homo sapiens	15.129	4	4	588138	88853
5805	1	Q86TW2	Q86TW2	AarF domain-containing protein kinase 1	ADCK1	Homo sapiens	60.578	4	4	361960	0
5806	1	Q86U42	Q86U42	Polyadenylate-binding protein 2	PABPN1	Homo sapiens	32.75	4	4	2341430	1581992

5807	1	Q86U90	Q86U90	Threonylcarbamoyl-AMP synthase	YRDC	Homo sapiens	29.326	4	4	1812744	217490
5808	1	Q86UX6	Q86UX6	Serine/threonine-protein kinase 32C	STK32C	Homo sapiens	54.994	4	4	204339	50375
5809	1	Q86V81	Q86V81	THO complex subunit 4	ALYREF	Homo sapiens	26.887	4	4	3662883	860977
5810	1	Q86V87	Q86V87	FHF complex subunit HOOK interacting protein 2B	FHIP2B	Homo sapiens	82.34	4	4	398695	15457
5811	1	Q86VI3	Q86VI3	Ras GTPase-activating-like protein IQGAP3	IQGAP3	Homo sapiens	184.7	4	4	120488	63117
5812	1	Q86VP1	Q86VP1	Tax1-binding protein 1	TAX1BP1	Homo sapiens	90.88	4	4	123729	408944
5813	1	Q86WN1	Q86WN1	F-BAR and double SH3 domains protein 1	FCHSD1	Homo sapiens	76.942	4	4	399108	129775
5814	1	Q86X27	Q86X27	Ras-specific guanine nucleotide-releasing factor RalGPS2	RALGPS2	Homo sapiens	65.166	5	4	393459	13073
5815	1	Q86YL5	Q86YL5	Testis development-related protein	TDRP	Homo sapiens	20.403	4	4	136018	17186
5816	1	Q86YR5	Q86YR5	G-protein-signaling modulator 1	GPSM1	Homo sapiens	74.511	4	4	136332	0
5817	1	Q86YT9	Q86YT9	Junctional adhesion molecule-like	JAML	Homo sapiens	44.339	4	4	47653	510149
5818	1	Q8IU60	Q8IU60	m7GpppN-mRNA hydrolase	DCP2	Homo sapiens	48.424	4	4	62268	189216
5819	1	Q8IUF8	Q8IUF8	Ribosomal oxygenase 2	RIOX2	Homo sapiens	52.802	4	4	354668	17992
5820	1	Q8IUH5	Q8IUH5	Palmitoyltransferase ZDHHC17	ZDHHC17	Homo sapiens	72.638	4	4	554466	324229
5821	1	Q8IV48	Q8IV48	3'-5' exoribonuclease 1	ERI1	Homo sapiens	40.065	4	4	0	415130
5822	1	Q8IVB4	Q8IVB4	Sodium/hydrogen exchanger 9	SLC9A9	Homo sapiens	72.565	4	4	840153	142104
5823	1	Q8IVB5	Q8IVB5	LIX1-like protein	LIX1L	Homo sapiens	36.561	4	4	861796	0
5824	1	Q8IVE3	Q8IVE3	Pleckstrin homology domain-containing family H member 2	PLEKHH2	Homo sapiens	168.231	4	4	47786	251079
5825	1	Q8IVL6	Q8IVL6	Prolyl 3-hydroxylase 3	P3H3	Homo sapiens	81.838	4	4	683297	189027
5826	1	Q8IWC1	Q8IWC1	MAP7 domain-containing protein 3	MAP7D3	Homo sapiens	98.431	4	4	132379	0
5827	1	Q8IWF6	Q8IWF6	Protein DENND6A	DENND6A	Homo sapiens	69.575	4	4	331781	104441
5828	1	Q8IWI9	Q8IWI9	MAX gene-associated protein	MGA	Homo sapiens	336.165	4	4	8053210	0
5829	1	Q8IWP9	Q8IWP9	Coiled-coil domain-containing protein 28A	CCDC28A	Homo sapiens	30.367	4	4	329472	0
5830	1	Q8IWS0	Q8IWS0	PHD finger protein 6	PHF6	Homo sapiens	41.29	4	4	241387	68897
5831	1	Q8IWZ6	Q8IWZ6	Bardet-Biedl syndrome 7 protein	BBS7	Homo sapiens	80.353	4	4	158993	0
5832	1	Q8IXM3	Q8IXM3	39S ribosomal protein L41, mitochondrial	MRPL41	Homo sapiens	15.383	4	4	1927987	164201
5833	1	Q8IXM6	Q8IXM6	Nurim	NRM	Homo sapiens	29.376	4	4	1023296	569073
5834	1	Q8IYS5	Q8IYS5	Osteoclast-associated immunoglobulin-like receptor	OSCAR	Homo sapiens	30.481	4	4	0	2139516
5835	1	Q8IYU2	Q8IYU2	E3 ubiquitin-protein ligase HACE1	HACE1	Homo sapiens	102.341	4	4	337375	146887
5836	1	Q8IZV5	Q8IZV5	Retinol dehydrogenase 10	RDH10	Homo sapiens	38.087	4	4	377608	1594307
5837	1	Q8N129	Q8N129	Protein canopy homolog 4	CNPY4	Homo sapiens	28.309	4	4	796970	154884
5838	1	Q8N149	Q8N149	Leukocyte immunoglobulin-like receptor subfamily A member 2	LILRA2	Homo sapiens	52.967	4	4	0	263936
5839	1	Q8N1A6	Q8N1A6	UPF0462 protein C4orf33	C4orf33	Homo sapiens	23.467	4	4	269319	33228
5840	1	Q8N465	Q8N465	D-2-hydroxyglutarate dehydrogenase, mitochondrial	D2HGDH	Homo sapiens	56.416	4	4	213902	27928
5841	1	Q8N4P3	Q8N4P3	Guanosine-3',5'-bis(diphosphate) 3'-pyrophosphohydrolase MESH1	HDDC3	Homo sapiens	20.329	4	4	463228	220659
5842	1	Q8N4Q1	Q8N4Q1	Mitochondrial intermembrane space import and assembly protein 40	CHCHD4	Homo sapiens	15.996	4	4	1857851	489493
5843	1	Q8N584	Q8N584	Tetratricopeptide repeat protein 39C	TTC39C	Homo sapiens	65.87	4	4	104375	172001
5844	1	Q8N5G2	Q8N5G2	Macoilin	MACO1	Homo sapiens	76.179	4	4	311561	0
5845	1	Q8N5H7	Q8N5H7	SH2 domain-containing protein 3C	SH2D3C	Homo sapiens	94.413	4	4	0	394624
5846	1	Q8N5I2	Q8N5I2	Arrestin domain-containing protein 1	ARRDC1	Homo sapiens	45.982	4	4	382635	702876
5847	1	Q8N5K1	Q8N5K1	CDGSH iron-sulfur domain-containing protein 2	CISD2	Homo sapiens	15.278	4	4	3339779	3769388
5848	1	Q8N5X7	Q8N5X7	Eukaryotic translation initiation factor 4E type 3	EIF4E3	Homo sapiens	24.44	4	4	726128	111304
5849	1	Q8N6Q3	Q8N6Q3	CD177 antigen	CD177	Homo sapiens	46.364	4	4	0	3118595
5850	1	Q8N6Y2	Q8N6Y2	Leucine-rich repeat-containing protein 17	LRRC17	Homo sapiens	51.801	4	4	333836	0
5851	1	Q8N8J7	Q8N8J7	Uncharacterized protein FAM241A	FAM241A	Homo sapiens	14.652	4	4	270565	283291
5852	1	Q8N9U0	Q8N9U0	Tandem C2 domains nuclear protein	TC2N	Homo sapiens	55.283	4	4	168254	24455
5853	1	Q8NAA5	Q8NAA5	Leucine-rich repeat-containing protein 75A	LRRC75A	Homo sapiens	37.779	4	4	262875	0
5854	1	Q8NAT1	Q8NAT1	Protein O-linked-mannose beta-1,4-N-acetylglucosaminyltransferase 2	POMGNT2	Homo sapiens	66.615	4	4	309840	33807

5855	1	Q8NAV1	Q8NAV1	Pre-mRNA-splicing factor 38A	PRPF38A	Homo sapiens	37.476	4	4	837491	442225
5856	1	Q8NB12	Q8NB12	Histone-lysine N-methyltransferase SMYD1	SMYD1	Homo sapiens	56.618	4	4	631413	0
5857	1	Q8NB37	Q8NB37	Glutamine amidotransferase-like class 1 domain-containing protein 1	GATD1	Homo sapiens	23.298	4	4	1210098	615375
5858	1	Q8NBL1	Q8NBL1	Protein O-glucosyltransferase 1	POGLUT1	Homo sapiens	46.189	4	4	1136316	11040
5859	1	Q8NC96	Q8NC96	Adaptin ear-binding coat-associated protein 1	NECAP1	Homo sapiens	29.734	4	4	1451975	298019
5860	1	Q8NCN4	Q8NCN4	E3 ubiquitin-protein ligase RNF169	RNF169	Homo sapiens	77.194	4	4	229353	17773
5861	1	Q8ND90	Q8ND90	Paraneoplastic antigen Ma1	PNMA1	Homo sapiens	39.763	4	4	437732	0
5862	1	Q8NE79	Q8NE79	Blood vessel epicardial substance	BVES	Homo sapiens	41.449	4	4	926630	0
5863	1	Q8NEC7	Q8NEC7	Glutathione S-transferase C-terminal domain-containing protein	GSTCD	Homo sapiens	71.08	4	4	119613	62954
5864	1	Q8NEZ3	Q8NEZ3	WD repeat-containing protein 19	WDR19	Homo sapiens	151.582	4	4	304644	0
5865	1	Q8NFA0	Q8NFA0	Ubiquitin carboxyl-terminal hydrolase 32	USP32	Homo sapiens	181.66	5	4	57467	90630
5866	1	Q8NFU0	Q8NFU0	Bestrophin-4	BEST4	Homo sapiens	53.5	4	4	602907	0
5867	1	Q8NFZ0	Q8NFZ0	F-box DNA helicase 1	FBH1	Homo sapiens	117.689	4	4	123349	0
5868	1	Q8NG68	Q8NG68	Tubulin--tyrosine ligase	TTL	Homo sapiens	43.214	4	4	441552	0
5869	1	Q8NHQ9	Q8NHQ9	ATP-dependent RNA helicase DDX55	DDX55	Homo sapiens	68.548	4	4	146109	183623
5870	1	Q8NHV1	Q8NHV1	GTPase IMAP family member 7	GIMAP7	Homo sapiens	34.508	4	4	414591	372518
5871	1	Q8TAL6	Q8TAL6	Fin bud initiation factor homolog	FIBIN	Homo sapiens	24.273	4	4	242878	0
5872	1	Q8TAV3	Q8TAV3	Cytochrome P450 2W1	CYP2W1	Homo sapiens	53.843	4	4	277259	0
5873	1	Q8TB36	Q8TB36	Ganglioside-induced differentiation-associated protein 1	GDAP1	Homo sapiens	41.347	4	4	985552	80936
5874	1	Q8TCF1	Q8TCF1	AN1-type zinc finger protein 1	ZFAND1	Homo sapiens	30.786	4	4	267230	195699
5875	1	Q8TCT8	Q8TCT8	Signal peptide peptidase-like 2A	SPPL2A	Homo sapiens	58.144	4	4	621511	1088067
5876	1	Q8TCZ2	Q8TCZ2	CD99 antigen-like protein 2	CD99L2	Homo sapiens	27.983	4	4	1094461	82376
5877	1	Q8TD22	Q8TD22	Sideroflexin-5	SFXN5	Homo sapiens	37.124	4	4	638417	62581
5878	1	Q8TDW0	Q8TDW0	Volume-regulated anion channel subunit LRRC8C	LRRC8C	Homo sapiens	92.451	4	4	180560	161159
5879	1	Q8TE04	Q8TE04	Pantothenate kinase 1	PANK1	Homo sapiens	64.343	5	4	314115	0
5880	1	Q8TED0	Q8TED0	U3 small nucleolar RNA-associated protein 15 homolog	UTP15	Homo sapiens	58.415	4	4	442510	170270
5881	1	Q8TED1	Q8TED1	Probable glutathione peroxidase 8	GPX8	Homo sapiens	23.883	4	4	809432	1196540
5882	1	Q8TEQ0	Q8TEQ0	Sorting nexin-29	SNX29	Homo sapiens	91.255	5	4	326584	101769
5883	1	Q8TF68	Q8TF68	Zinc finger protein 384	ZNF384	Homo sapiens	63.221	4	4	479634	535955
5884	1	Q8WUA8	Q8WUA8	Tsukushi	TSKU	Homo sapiens	37.808	4	4	55643	566828
5885	1	Q8WUB8	Q8WUB8	PHD finger protein 10	PHF10	Homo sapiens	56.051	4	4	546026	433640
5886	1	Q8WV93	Q8WV93	AFG1-like ATPase	AFG1L	Homo sapiens	54.848	4	4	448512	0
5887	1	Q8WVC0	Q8WVC0	RNA polymerase-associated protein LEO1	LEO1	Homo sapiens	75.405	4	4	332458	328173
5888	1	Q8WVM0	Q8WVM0	Dimethyladenosine transferase 1, mitochondrial	TFB1M	Homo sapiens	39.542	4	4	638143	172762
5889	1	Q8WVP5	Q8WVP5	Tumor necrosis factor alpha-induced protein 8-like protein 1	TNFAIP8L1	Homo sapiens	20.827	4	4	255967	0
5890	1	Q8WXW3	Q8WXW3	Progesterone-induced-blocking factor 1	PIBF1	Homo sapiens	89.804	4	4	311136	0
5891	1	Q8WYJ6	Q8WYJ6	Septin-1	SEPTIN1	Homo sapiens	42.384	4	4	575583	252160
5892	1	Q8WYQ5	Q8WYQ5	Microprocessor complex subunit DGCR8	DGCR8	Homo sapiens	86.044	4	4	163167	27613
5893	1	Q8WYR1	Q8WYR1	Phosphoinositide 3-kinase regulatory subunit 5	PIK3R5	Homo sapiens	97.349	4	4	0	298893
5894	1	Q92485	Q92485	Acid sphingomyelinase-like phosphodiesterase 3b	SMPDL3B	Homo sapiens	50.816	4	4	571031	677606
5895	1	Q92503	Q92503	SEC14-like protein 1	SEC14L1	Homo sapiens	81.251	4	4	112122	147367
5896	1	Q92733	Q92733	Proline-rich protein PRCC	PRCC	Homo sapiens	52.419	4	4	157817	11808
5897	1	Q92759	Q92759	General transcription factor IIH subunit 4	GTF2H4	Homo sapiens	52.186	4	4	283756	200679
5898	1	Q92879	Q92879	CUGBP Elav-like family member 1	CELF1	Homo sapiens	52.064	4	4	445317	156072
5899	1	Q92963	Q92963	GTP-binding protein Rit1	RIT1	Homo sapiens	25.143	4	4	249649	452620
5900	1	Q92995	Q92995	Ubiquitin carboxyl-terminal hydrolase 13	USP13	Homo sapiens	97.329	4	4	420330	0
5901	1	Q92997	Q92997	Segment polarity protein dishevelled homolog DVL-3	DVL3	Homo sapiens	78.057	4	4	716015	63795
5902	1	Q969J3	Q969J3	BLOC-1-related complex subunit 5	BORCS5	Homo sapiens	22.221	4	4	558621	322279

5903	1 Q969Q4	Q969Q4	ADP-ribosylation factor-like protein 11	ARL11	Homo sapiens	21.392	4	4	50744	1107145
5904	1 Q96AB6	Q96AB6	Protein N-terminal asparagine amidohydrolase	NTAN1	Homo sapiens	34.678	4	4	679319	225807
5905	1 Q96AP7	Q96AP7	Endothelial cell-selective adhesion molecule	ESAM	Homo sapiens	41.176	4	4	359433	341294
5906	1 Q96AT1	Q96AT1	Uncharacterized protein KIAA1143	KIAA1143	Homo sapiens	17.465	4	4	789709	169980
5907	1 Q96B23	Q96B23	Protein C18orf25	C18orf25	Homo sapiens	43.394	4	4	665694	746003
5908	1 Q96B36	Q96B36	Proline-rich AKT1 substrate 1	AKT1S1	Homo sapiens	27.384	4	4	1128937	244563
5909	1 Q96BQ1	Q96BQ1	Protein FAM3D	FAM3D	Homo sapiens	24.963	4	4	22477014	409023
5910	1 Q96BR1	Q96BR1	Serine/threonine-protein kinase Sgk3	SGK3	Homo sapiens	57.109	4	4	185320	294023
5911	1 Q96BR5	Q96BR5	Cytochrome c oxidase assembly factor 7	COA7	Homo sapiens	25.71	4	4	156453	0
5912	1 Q96BS2	Q96BS2	Calcineurin B homologous protein 3	TESC	Homo sapiens	24.749	4	4	0	747033
5913	1 Q96BZ8	Q96BZ8	Leukocyte receptor cluster member 1	LENG1	Homo sapiens	30.53	4	4	244057	0
5914	1 Q96C01	Q96C01	Protein FAM136A	FAM136A	Homo sapiens	15.64	4	4	1090901	322465
5915	1 Q96CD0	Q96CD0	F-box/LRR-repeat protein 8	FBXL8	Homo sapiens	40.516	4	4	917932	224331
5916	1 Q96CN4	Q96CN4	EVI5-like protein	EVI5L	Homo sapiens	91.376	4	4	155414	0
5917	1 Q96D53	Q96D53	Atypical kinase COQ8B, mitochondrial	COQ8B	Homo sapiens	60.071	4	4	181442	0
5918	1 Q96E11	Q96E11	Ribosome-recycling factor, mitochondrial	MRRF	Homo sapiens	29.277	4	4	1308325	161347
5919	1 Q96EL2	Q96EL2	28S ribosomal protein S24, mitochondrial	MRPS24	Homo sapiens	19.014	4	4	452810	58809
5920	1 Q96EP5	Q96EP5	DAZ-associated protein 1	DAZAP1	Homo sapiens	43.384	4	4	5176721	2528956
5921	1 Q96EV2	Q96EV2	RNA-binding protein 33	RBM33	Homo sapiens	129.989	4	4	318229	54139
5922	1 Q96EY4	Q96EY4	Translation machinery-associated protein 16	TMA16	Homo sapiens	23.865	4	4	468811	0
5923	1 Q96EY5	Q96EY5	Multivesicular body subunit 12A	MVB12A	Homo sapiens	28.78	4	4	793489	287923
5924	1 Q96EZ8	Q96EZ8	Microspherule protein 1	MCRS1	Homo sapiens	51.803	4	4	81485	0
5925	1 Q96FZ7	Q96FZ7	Charged multivesicular body protein 6	CHMP6	Homo sapiens	23.485	4	4	876316	443856
5926	1 Q96G25	Q96G25	Mediator of RNA polymerase II transcription subunit 8	MED8	Homo sapiens	29.078	4	4	180142	106680
5927	1 Q96G28	Q96G28	Cilia- and flagella-associated protein 36	CFAP36	Homo sapiens	39.447	4	4	279366	0
5928	1 Q96GC5	Q96GC5	39S ribosomal protein L48, mitochondrial	MRPL48	Homo sapiens	23.935	4	4	1088895	89272
5929	1 Q96GP6	Q96GP6	Scavenger receptor class F member 2	SCARF2	Homo sapiens	92.387	4	4	248356	0
5930	1 Q96HD1	Q96HD1	Protein disulfide isomerase CRELD1	CRELD1	Homo sapiens	45.439	4	4	423133	317938
5931	1 Q96HU1	Q96HU1	Small G protein signaling modulator 3	SGSM3	Homo sapiens	85.353	4	4	466978	31912
5932	1 Q96HY7	Q96HY7	2-oxoadipate dehydrogenase complex component E1	DHTKD1	Homo sapiens	103.081	4	4	322302	19056
5933	1 Q96J01	Q96J01	THO complex subunit 3	THOC3	Homo sapiens	38.77	4	4	1609527	1142849
5934	1 Q96JG8	Q96JG8	Melanoma-associated antigen D4	MAGED4B	Homo sapiens	81.377	4	4	167854	0
5935	1 Q96JP5	Q96JP5	E3 ubiquitin-protein ligase ZFP91	ZFP91	Homo sapiens	63.445	4	4	654265	50081
5936	1 Q96JX3	Q96JX3	Protein SERAC1	SERAC1	Homo sapiens	74.146	4	4	158338	0
5937	1 Q96K37	Q96K37	Solute carrier family 35 member E1	SLC35E1	Homo sapiens	44.773	4	4	306440	100104
5938	1 Q96KM6	Q96KM6	Zinc finger protein 512B	ZNF512B	Homo sapiens	97.269	4	4	215395	20099
5939	1 Q96MG7	Q96MG7	Non-structural maintenance of chromosomes element 3 homolog	NSMCE3	Homo sapiens	34.308	4	4	343073	80462
5940	1 Q96MH6	Q96MH6	Transmembrane protein 68	TMEM68	Homo sapiens	37.424	4	4	750900	194097
5941	1 Q96MI6	Q96MI6	Protein phosphatase 1M	PPM1M	Homo sapiens	51.134	4	4	34121	118225
5942	1 Q96MU7	Q96MU7	YTH domain-containing protein 1	YTHDC1	Homo sapiens	84.701	4	4	576429	344959
5943	1 Q96N64	Q96N64	PWWP domain-containing protein 2A	PWWP2A	Homo sapiens	81.961	4	4	210780	0
5944	1 Q96P11	Q96P11	28S rRNA (cytosine-C(5))-methyltransferase	NSUN5	Homo sapiens	46.692	5	4	401175	229212
5945	1 Q96P53	Q96P53	WD repeat and FYVE domain-containing protein 2	WDFY2	Homo sapiens	45.154	4	4	205985	286003
5946	1 Q96PQ0	Q96PQ0	VPS10 domain-containing receptor SorCS2	SORCS2	Homo sapiens	128.155	4	4	175399	0
5947	1 Q96Q07	Q96Q07	BTB/POZ domain-containing protein 9	BTBD9	Homo sapiens	69.189	4	4	63681	86235
5948	1 Q96QE2	Q96QE2	Proton myo-inositol cotransporter	SLC2A13	Homo sapiens	70.37	4	4	410735	792823
5949	1 Q96QR8	Q96QR8	Transcriptional activator protein Pur-beta	PURB	Homo sapiens	33.242	4	4	1567241	223018
5950	1 Q96RS0	Q96RS0	Trimethylguanosine synthase	TGS1	Homo sapiens	96.623	4	4	267636	53933

5951	1	Q96RY7	Q96RY7	Intraflagellar transport protein 140 homolog	IFT140	Homo sapiens	165.193	4	4	233717	0
5952	1	Q96S15	Q96S15	GATOR complex protein WDR24	WDR24	Homo sapiens	88.209	4	4	217442	0
5953	1	Q96S19	Q96S19	Methyltransferase-like 26	METTL26	Homo sapiens	22.578	4	4	1174097	380614
5954	1	Q96S90	Q96S90	LysM and putative peptidoglycan-binding domain-containing protein 1	LYSMD1	Homo sapiens	25.004	4	4	371540	0
5955	1	Q96S99	Q96S99	Pleckstrin homology domain-containing family F member 1	PLEKHF1	Homo sapiens	31.197	4	4	674461	455268
5956	1	Q96SM3	Q96SM3	Probable carboxypeptidase X1	CPXM1	Homo sapiens	81.669	4	4	0	548439
5957	1	Q96SY0	Q96SY0	Integrator complex subunit 14	INTS14	Homo sapiens	57.472	4	4	566201	205946
5958	1	Q99471	Q99471	Prefoldin subunit 5	PFDN5	Homo sapiens	17.329	4	4	1718481	389839
5959	1	Q99490	Q99490	Arf-GAP with GTPase, ANK repeat and PH domain-containing protein 2	AGAP2	Homo sapiens	124.677	4	4	0	565258
5960	1	Q99571	Q99571	P2X purinoceptor 4	P2RX4	Homo sapiens	43.369	4	4	1456424	2518922
5961	1	Q99572	Q99572	P2X purinoceptor 7	P2RX7	Homo sapiens	68.587	4	4	87866	31840
5962	1	Q99608	Q99608	Necdin	NDN	Homo sapiens	36.087	4	4	87166	0
5963	1	Q99729	Q99729	Heterogeneous nuclear ribonucleoprotein A/B	HNRNPAB	Homo sapiens	36.224	4	4	7664630	1675039
5964	1	Q99733	Q99733	Nucleosome assembly protein 1-like 4	NAP1L4	Homo sapiens	42.824	4	4	9612000	3103188
5965	1	Q99759	Q99759	Mitogen-activated protein kinase kinase kinase 3	MAP3K3	Homo sapiens	70.898	5	4	225039	466559
5966	1	Q99856	Q99856	AT-rich interactive domain-containing protein 3A	ARID3A	Homo sapiens	62.89	5	4	0	719419
5967	1	Q99943	Q99943	1-acyl-sn-glycerol-3-phosphate acyltransferase alpha	AGPAT1	Homo sapiens	31.717	4	4	1221904	484481
5968	1	Q99952	Q99952	Tyrosine-protein phosphatase non-receptor type 18	PTPN18	Homo sapiens	50.482	4	4	151950	216114
5969	1	Q99969	Q99969	Retinoic acid receptor responder protein 2	RARRES2	Homo sapiens	18.618	4	4	1042768	0
5970	1	Q9BPW9	Q9BPW9	Dehydrogenase/reductase SDR family member 9	DHRS9	Homo sapiens	35.226	4	4	277832	0
5971	1	Q9BPX3	Q9BPX3	Condensin complex subunit 3	NCAPG	Homo sapiens	114.337	4	4	129133	95607
5972	1	Q9BPZ7	Q9BPZ7	Target of rapamycin complex 2 subunit MAPKAP1	MAPKAP1	Homo sapiens	59.122	4	4	272447	62298
5973	1	Q9BQA9	Q9BQA9	Cytochrome b-245 chaperone 1	CYBC1	Homo sapiens	20.773	4	4	1830449	2806944
5974	1	Q9BQC3	Q9BQC3	2-(3-amino-3-carboxypropyl)histidine synthase subunit 2	DPH2	Homo sapiens	52.084	4	4	182641	0
5975	1	Q9BRA2	Q9BRA2	Thioredoxin domain-containing protein 17	TXNDC17	Homo sapiens	13.941	4	4	7159875	2752919
5976	1	Q9BRD0	Q9BRD0	BUD13 homolog	BUD13	Homo sapiens	70.52	4	4	324062	0
5977	1	Q9BRP4	Q9BRP4	Proteasomal ATPase-associated factor 1	PAAF1	Homo sapiens	42.189	4	4	1125660	370076
5978	1	Q9BRQ0	Q9BRQ0	Pygopus homolog 2	PYGO2	Homo sapiens	41.244	4	4	1944953	40636
5979	1	Q9BRQ8	Q9BRQ8	Ferroptosis suppressor protein 1	AIFM2	Homo sapiens	40.528	4	4	634037	356056
5980	1	Q9BRS2	Q9BRS2	Serine/threonine-protein kinase RIO1	RIOK1	Homo sapiens	65.584	4	4	131458	0
5981	1	Q9BSF4	Q9BSF4	Mitochondrial import inner membrane translocase subunit Tim29	TIMM29	Homo sapiens	29.233	4	4	1085792	0
5982	1	Q9BSV6	Q9BSV6	tRNA-splicing endonuclease subunit Sen34	TSEN34	Homo sapiens	33.65	4	4	297050	0
5983	1	Q9BU23	Q9BU23	Lipase maturation factor 2	LMF2	Homo sapiens	79.698	4	4	742416	586647
5984	1	Q9BUB5	Q9BUB5	MAP kinase-interacting serine/threonine-protein kinase 1	MKNK1	Homo sapiens	51.342	4	4	268192	323231
5985	1	Q9BUE6	Q9BUE6	Iron-sulfur cluster assembly 1 homolog, mitochondrial	ISCA1	Homo sapiens	14.18	4	4	460682	116401
5986	1	Q9BUL5	Q9BUL5	PHD finger protein 23	PHF23	Homo sapiens	43.817	4	4	529904	138488
5987	1	Q9BUL9	Q9BUL9	Ribonuclease P protein subunit p25	RPP25	Homo sapiens	20.629	4	4	467253	58347
5988	1	Q9BV35	Q9BV35	Calcium-binding mitochondrial carrier protein SCaMC-3	SLC25A23	Homo sapiens	52.379	5	4	870924	0
5989	1	Q9BV40	Q9BV40	Vesicle-associated membrane protein 8	VAMP8	Homo sapiens	11.434	4	4	3231392	8242271
5990	1	Q9BV79	Q9BV79	Enoyl-[acyl-carrier-protein] reductase, mitochondrial	MECR	Homo sapiens	40.462	4	4	1625680	389787
5991	1	Q9BVA0	Q9BVA0	Katanin p80 WD40 repeat-containing subunit B1	KATNB1	Homo sapiens	72.335	4	4	298629	0
5992	1	Q9BVC5	Q9BVC5	Ashwin	C2orf49	Homo sapiens	25.857	4	4	510917	0
5993	1	Q9BVL2	Q9BVL2	Nucleoporin p58/p45	NUP58	Homo sapiens	60.896	4	4	658953	344577
5994	1	Q9BVS4	Q9BVS4	Serine/threonine-protein kinase RIO2	RIOK2	Homo sapiens	63.285	4	4	252538	72647
5995	1	Q9BW83	Q9BW83	Intraflagellar transport protein 27 homolog	IFT27	Homo sapiens	20.482	4	4	1540063	1028981
5996	1	Q9BWE0	Q9BWE0	Replication initiator 1	REPIN1	Homo sapiens	63.574	4	4	429926	0
5997	1	Q9BWH2	Q9BWH2	FUN14 domain-containing protein 2	FUNDC2	Homo sapiens	20.675	4	4	7334753	1273370
5998	1	Q9BYJ9	Q9BYJ9	YTH domain-containing family protein 1	YTHDF1	Homo sapiens	60.871	4	4	345221	172435

5999	1 Q9BZ23	Q9BZ23	Pantothenate kinase 2, mitochondrial	PANK2	Homo sapiens	62.682	6	4	239614	230012
6000	1 Q9BZ67	Q9BZ67	FERM domain-containing protein 8	FRMD8	Homo sapiens	51.217	5	4	790786	669049
6001	1 Q9BZX2	Q9BZX2	Uridine-cytidine kinase 2	UCK2	Homo sapiens	29.3	4	4	676027	132127
6002	1 Q9C035	Q9C035	Tripartite motif-containing protein 5	TRIM5	Homo sapiens	56.342	5	4	674312	95840
6003	1 Q9GZM8	Q9GZM8	Nuclear distribution protein nudE-like 1	NDEL1	Homo sapiens	38.372	4	4	154495	42065
6004	1 Q9GZN7	Q9GZN7	Protein rogdi homolog	ROGDI	Homo sapiens	32.254	4	4	1182982	52629
6005	1 Q9GZQ3	Q9GZQ3	COMM domain-containing protein 5	COMMD5	Homo sapiens	24.669	4	4	844738	528765
6006	1 Q9GZS1	Q9GZS1	DNA-directed RNA polymerase I subunit RPA49	POLR1E	Homo sapiens	47.26	4	4	207244	0
6007	1 Q9GZS9	Q9GZS9	Carbohydrate sulfotransferase 5	CHST5	Homo sapiens	46.161	7	4	499561	0
6008	1 Q9GZY6	Q9GZY6	Linker for activation of T-cells family member 2	LAT2	Homo sapiens	26.551	4	4	0	369909
6009	1 Q9H000	Q9H000	E3 ubiquitin-protein ligase makorin-2	MKRN2	Homo sapiens	46.941	4	4	701251	205246
6010	1 Q9H019	Q9H019	Mitochondrial fission regulator 1-like	MTFR1L	Homo sapiens	31.957	4	4	345697	54419
6011	1 Q9H061	Q9H061	Transmembrane protein 126A	TMEM126A	Homo sapiens	21.526	4	4	3367623	1405566
6012	1 Q9H0K1	Q9H0K1	Serine/threonine-protein kinase SIK2	SIK2	Homo sapiens	103.916	4	4	4895429	12790
6013	1 Q9H0T7	Q9H0T7	Ras-related protein Rab-17	RAB17	Homo sapiens	23.492	4	4	74181	0
6014	1 Q9H0U4	Q9H0U4	Ras-related protein Rab-1B	RAB1B	Homo sapiens	22.171	11	4	1948489	400822
6015	1 Q9H0U9	Q9H0U9	Testis-specific Y-encoded-like protein 1	TSPYL1	Homo sapiens	49.194	4	4	262543	0
6016	1 Q9H173	Q9H173	Nucleotide exchange factor SIL1	SIL1	Homo sapiens	52.087	4	4	377278	405134
6017	1 Q9H190	Q9H190	Syntenin-2	SDCBP2	Homo sapiens	31.592	4	4	754903	142040
6018	1 Q9H1A3	Q9H1A3	Protein-L-histidine N-pros-methyltransferase	METTL9	Homo sapiens	36.536	4	4	197562	725207
6019	1 Q9H1C4	Q9H1C4	Protein unc-93 homolog B1	UNC93B1	Homo sapiens	66.632	4	4	133942	534983
6020	1 Q9H1D9	Q9H1D9	DNA-directed RNA polymerase III subunit RPC6	POLR3F	Homo sapiens	35.682	4	4	316654	41637
6021	1 Q9H1N7	Q9H1N7	Adenosine 3'-phospho 5'-phosphosulfate transporter 2	SLC35B3	Homo sapiens	44.589	4	4	212025	55859
6022	1 Q9H2H8	Q9H2H8	Peptidyl-prolyl cis-trans isomerase-like 3	PPIL3	Homo sapiens	18.154	4	4	1764706	815119
6023	1 Q9H2V7	Q9H2V7	Protein spinster homolog 1	SPNS1	Homo sapiens	56.63	4	4	641480	854673
6024	1 Q9H2W6	Q9H2W6	39S ribosomal protein L46, mitochondrial	MRPL46	Homo sapiens	31.706	4	4	1967034	449671
6025	1 Q9H334	Q9H334	Forkhead box protein P1	FOXP1	Homo sapiens	75.317	4	4	498895	0
6026	1 Q9H3H3	Q9H3H3	UPF0696 protein C11orf68	C11orf68	Homo sapiens	31.43	4	4	2024819	407092
6027	1 Q9H3K6	Q9H3K6	BolA-like protein 2	BOLA2B	Homo sapiens	10.115	4	4	3297752	3488196
6028	1 Q9H3Z4	Q9H3Z4	DnaJ homolog subfamily C member 5	DNAJC5	Homo sapiens	22.148	4	4	1356652	5901568
6029	1 Q9H410	Q9H410	Kinetochore-associated protein DSN1 homolog	DSN1	Homo sapiens	40.065	4	4	133858	20902
6030	1 Q9H446	Q9H446	RWD domain-containing protein 1	RWDD1	Homo sapiens	27.939	4	4	509425	289297
6031	1 Q9H477	Q9H477	Ribokinase	RBKS	Homo sapiens	34.142	4	4	761899	304282
6032	1 Q9H497	Q9H497	Torsin-3A	TOR3A	Homo sapiens	46.199	4	4	31818355	41031
6033	1 Q9H582	Q9H582	Zinc finger protein 644	ZNF644	Homo sapiens	149.569	4	4	144185	0
6034	1 Q9H5Q4	Q9H5Q4	Dimethyladenosine transferase 2, mitochondrial	TFB2M	Homo sapiens	45.349	4	4	249195	61064
6035	1 Q9H6A9	Q9H6A9	Pecanex-like protein 3	PCNX3	Homo sapiens	222.041	4	4	81332	0
6036	1 Q9H6W3	Q9H6W3	Ribosomal oxygenase 1	RIOX1	Homo sapiens	71.086	4	4	495247	74341
6037	1 Q9H6X2	Q9H6X2	Anthrax toxin receptor 1	ANTXR1	Homo sapiens	62.789	4	4	383359	686327
6038	1 Q9H6Y2	Q9H6Y2	WD repeat-containing protein 55	WDR55	Homo sapiens	42.068	4	4	552470	341041
6039	1 Q9H720	Q9H720	PGAP2-interacting protein	CWH43	Homo sapiens	78.585	4	4	410560	0
6040	1 Q9H7F0	Q9H7F0	Polyamine-transporting ATPase 13A3	ATP13A3	Homo sapiens	138.046	4	4	179318	280508
6041	1 Q9H8J5	Q9H8J5	MANSC domain-containing protein 1	MANSC1	Homo sapiens	46.809	4	4	312937	109840
6042	1 Q9H8M1	Q9H8M1	Coenzyme Q-binding protein COQ10 homolog B, mitochondrial	COQ10B	Homo sapiens	27.071	4	4	633988	0
6043	1 Q9H8Y8	Q9H8Y8	Golgi reassembly-stacking protein 2	GORASP2	Homo sapiens	47.146	4	4	2058483	787186
6044	1 Q9H944	Q9H944	Mediator of RNA polymerase II transcription subunit 20	MED20	Homo sapiens	23.222	4	4	591012	1989552
6045	1 Q9H974	Q9H974	Queuine tRNA-ribosyltransferase accessory subunit 2	QTRT2	Homo sapiens	46.712	4	4	351081	109369
6046	1 Q9H977	Q9H977	WD repeat-containing protein 54	WDR54	Homo sapiens	35.892	4	4	373442	50268

6047	1	Q9H9Q2	Q9H9Q2	COP9 signalosome complex subunit 7b	COPS7B	Homo sapiens	29.622	4	4	1910821	1034188
6048	1	Q9HAS0	Q9HAS0	Protein Njmu-R1	C17orf75	Homo sapiens	44.621	4	4	520729	364929
6049	1	Q9HAV0	Q9HAV0	Guanine nucleotide-binding protein subunit beta-4	GNB4	Homo sapiens	37.568	9	4	908599	437512
6050	1	Q9HB58	Q9HB58	Sp110 nuclear body protein	SP110	Homo sapiens	78.397	4	4	102181	255151
6051	1	Q9HBI6	Q9HBI6	Cytochrome P450 4F11	CYP4F11	Homo sapiens	60.145	7	4	97217	379642
6052	1	Q9HBM0	Q9HBM0	Vezatin	VEZT	Homo sapiens	88.665	4	4	315535	0
6053	1	Q9HC16	Q9HC16	DNA dC->dU-editing enzyme APOBEC-3G	APOBEC3G	Homo sapiens	46.408	5	4	228736	296468
6054	1	Q9HC21	Q9HC21	Mitochondrial thiamine pyrophosphate carrier	SLC25A19	Homo sapiens	35.509	4	4	381848	285015
6055	1	Q9HC78	Q9HC78	Zinc finger and BTB domain-containing protein 20	ZBTB20	Homo sapiens	81.083	4	4	352858	0
6056	1	Q9HCE5	Q9HCE5	N6-adenosine-methyltransferase non-catalytic subunit	METTL14	Homo sapiens	52.152	4	4	688584	214100
6057	1	Q9HCN8	Q9HCN8	Stromal cell-derived factor 2-like protein 1	SDF2L1	Homo sapiens	23.599	4	4	1277681	728106
6058	1	Q9HCU0	Q9HCU0	Endosialin	CD248	Homo sapiens	80.861	4	4	1409491	520796
6059	1	Q9HD15	Q9HD15	Steroid receptor RNA activator 1	SRA1	Homo sapiens	24.4	4	4	718946	223533
6060	1	Q9NP97	Q9NP97	Dynein light chain roadblock-type 1	DYNLRB1	Homo sapiens	10.922	6	4	1522729	415434
6061	1	Q9NQ11	Q9NQ11	Polyamine-transporting ATPase 13A2	ATP13A2	Homo sapiens	128.794	4	4	27230	277059
6062	1	Q9NQ29	Q9NQ29	Putative RNA-binding protein Luc7-like 1	LUC7L	Homo sapiens	43.728	4	4	881919	392706
6063	1	Q9NQ55	Q9NQ55	Suppressor of SWI4 1 homolog	PPAN	Homo sapiens	53.195	4	4	364330	81705
6064	1	Q9NQS3	Q9NQS3	Nectin-3	NECTIN3	Homo sapiens	61.002	4	4	434423	0
6065	1	Q9NQT4	Q9NQT4	Exosome complex component RRP46	EXOSC5	Homo sapiens	25.249	4	4	144372	400298
6066	1	Q9NQZ5	Q9NQZ5	StAR-related lipid transfer protein 7, mitochondrial	STARD7	Homo sapiens	43.112	4	4	806663	238902
6067	1	Q9NR31	Q9NR31	GTP-binding protein SAR1a	SAR1A	Homo sapiens	22.369	5	4	4144267	4173341
6068	1	Q9NRD5	Q9NRD5	PRKCA-binding protein	PICK1	Homo sapiens	46.601	4	4	115376	136481
6069	1	Q9NRW4	Q9NRW4	Dual specificity protein phosphatase 22	DUSP22	Homo sapiens	20.911	4	4	635908	203169
6070	1	Q9NRX2	Q9NRX2	39S ribosomal protein L17, mitochondrial	MRPL17	Homo sapiens	20.05	4	4	1329401	272929
6071	1	Q9NRX4	Q9NRX4	14 kDa phosphohistidine phosphatase	PHPT1	Homo sapiens	13.833	4	4	7907212	1791725
6072	1	Q9NRX5	Q9NRX5	Serine incorporator 1	SERINC1	Homo sapiens	50.493	4	4	535325	613839
6073	1	Q9NRZ5	Q9NRZ5	1-acyl-sn-glycerol-3-phosphate acyltransferase delta	AGPAT4	Homo sapiens	44.021	4	4	493582	0
6074	1	Q9NS69	Q9NS69	Mitochondrial import receptor subunit TOM22 homolog	TOMM22	Homo sapiens	15.521	4	4	2310078	474096
6075	1	Q9NS93	Q9NS93	Transmembrane 7 superfamily member 3	TM7SF3	Homo sapiens	64.167	4	4	112124	802315
6076	1	Q9NUA8	Q9NUA8	Zinc finger and BTB domain-containing protein 40	ZBTB40	Homo sapiens	138.121	4	4	72416	0
6077	1	Q9NUL5	Q9NUL5	Shiftless antiviral inhibitor of ribosomal frameshifting protein	SHFL	Homo sapiens	33.11	4	4	808700	442481
6078	1	Q9NUP9	Q9NUP9	Protein lin-7 homolog C	LIN7C	Homo sapiens	21.833	4	4	1450780	139162
6079	1	Q9NUS5	Q9NUS5	AP-5 complex subunit sigma-1	AP5S1	Homo sapiens	22.523	4	4	232491	110203
6080	1	Q9NV06	Q9NV06	DDB1- and CUL4-associated factor 13	DCAF13	Homo sapiens	51.403	4	4	361121	38170
6081	1	Q9NVN8	Q9NVN8	Guanine nucleotide-binding protein-like 3-like protein	GNL3L	Homo sapiens	65.574	4	4	238580	36759
6082	1	Q9NVU0	Q9NVU0	DNA-directed RNA polymerase III subunit RPC5	POLR3E	Homo sapiens	79.9	4	4	189808	0
6083	1	Q9NVX0	Q9NVX0	HAUS augmin-like complex subunit 2	HAUS2	Homo sapiens	26.933	4	4	104587	47021
6084	1	Q9NWM3	Q9NWM3	CUE domain-containing protein 1	CUEDC1	Homo sapiens	42.257	4	4	473951	39296
6085	1	Q9NWM8	Q9NWM8	Peptidyl-prolyl cis-trans isomerase FKBP14	FKBP14	Homo sapiens	24.172	4	4	318266	313949
6086	1	Q9NWT1	Q9NWT1	p21-activated protein kinase-interacting protein 1	PAK1IP1	Homo sapiens	43.965	4	4	367976	129607
6087	1	Q9NWT6	Q9NWT6	Hypoxia-inducible factor 1-alpha inhibitor	HIF1AN	Homo sapiens	40.285	4	4	335710	74166
6088	1	Q9NWU2	Q9NWU2	Glucose-induced degradation protein 8 homolog	GID8	Homo sapiens	26.748	4	4	631421	119599
6089	1	Q9NX62	Q9NX62	Golgi-resident adenosine 3',5'-bisphosphate 3'-phosphatase	BPNT2	Homo sapiens	38.681	4	4	802704	871614
6090	1	Q9NXG6	Q9NXG6	Transmembrane prolyl 4-hydroxylase	P4HTM	Homo sapiens	56.662	4	4	212480	0
6091	1	Q9NXX6	Q9NXX6	Non-structural maintenance of chromosomes element 4 homolog A	NSMCE4A	Homo sapiens	44.3	4	4	232367	260405
6092	1	Q9NY25	Q9NY25	C-type lectin domain family 5 member A	CLEC5A	Homo sapiens	21.523	4	4	0	1210283
6093	1	Q9NY93	Q9NY93	Probable ATP-dependent RNA helicase DDX56	DDX56	Homo sapiens	61.588	4	4	371360	32829
6094	1	Q9NYI0	Q9NYI0	PH and SEC7 domain-containing protein 3	PSD3	Homo sapiens	116.036	4	4	147887	0

6095	1	Q9NYN1	Q9NYN1	Ras-like protein family member 12	RASL12	Homo sapiens	29.663	4	4	192252	0
6096	1	Q9NYS0	Q9NYS0	NF-kappa-B inhibitor-interacting Ras-like protein 1	NKIRAS1	Homo sapiens	21.641	4	4	308565	38378
6097	1	Q9NYT0	Q9NYT0	Pleckstrin-2	PLEK2	Homo sapiens	39.969	4	4	1053191	0
6098	1	Q9NZQ7	Q9NZQ7	Programmed cell death 1 ligand 1	CD274	Homo sapiens	33.276	4	4	0	655349
6099	1	Q9P000	Q9P000	COMM domain-containing protein 9	COMMD9	Homo sapiens	21.819	4	4	701474	271154
6100	1	Q9P0L0	Q9P0L0	Vesicle-associated membrane protein-associated protein A	VAPA	Homo sapiens	27.893	4	4	13762516	4368048
6101	1	Q9P0L2	Q9P0L2	Serine/threonine-protein kinase MARK1	MARK1	Homo sapiens	89.006	4	4	262622	0
6102	1	Q9P0N9	Q9P0N9	TBC1 domain family member 7	TBC1D7	Homo sapiens	33.972	4	4	228282	102030
6103	1	Q9P0P0	Q9P0P0	E3 ubiquitin-protein ligase RNF181	RNF181	Homo sapiens	17.909	4	4	642535	200219
6104	1	Q9P0S2	Q9P0S2	Cytochrome c oxidase assembly protein COX16 homolog, mitochondrial	COX16	Homo sapiens	12.293	4	4	611661	0
6105	1	Q9P121	Q9P121	Neurotrimin	NTM	Homo sapiens	37.97	5	4	1005906	0
6106	1	Q9P1U1	Q9P1U1	Actin-related protein 3B	ACTR3B	Homo sapiens	47.606	4	4	478160	0
6107	1	Q9P212	Q9P212	1-phosphatidylinositol 4,5-bisphosphate phosphodiesterase epsilon-1	PLCE1	Homo sapiens	258.72	4	4	302107	0
6108	1	Q9P246	Q9P246	Stromal interaction molecule 2	STIM2	Homo sapiens	83.973	4	4	339595	214715
6109	1	Q9P287	Q9P287	BRCA2 and CDKN1A-interacting protein	BCCIP	Homo sapiens	35.981	4	4	474371	331360
6110	1	Q9P2P1	Q9P2P1	Protein NYNRIN	NYNRIN	Homo sapiens	208.372	4	4	102190	0
6111	1	Q9P2Q2	Q9P2Q2	FERM domain-containing protein 4A	FRMD4A	Homo sapiens	115.457	4	4	187595	0
6112	1	Q9P2S5	Q9P2S5	WD repeat-containing protein WRAP73	WRAP73	Homo sapiens	51.587	4	4	234134	49500
6113	1	Q9UBF8	Q9UBF8	Phosphatidylinositol 4-kinase beta	PI4KB	Homo sapiens	91.379	4	4	263666	0
6114	1	Q9UBP6	Q9UBP6	tRNA (guanine-N(7)-)-methyltransferase	METTL1	Homo sapiens	31.472	4	4	119245	46191
6115	1	Q9UBU6	Q9UBU6	Protein FAM8A1	FAM8A1	Homo sapiens	44.122	4	4	528706	141528
6116	1	Q9UBV8	Q9UBV8	Peflin	PEF1	Homo sapiens	30.38	4	4	1436273	259981
6117	1	Q9UDX3	Q9UDX3	SEC14-like protein 4	SEC14L4	Homo sapiens	46.645	4	4	195543	221277
6118	1	Q9UEE9	Q9UEE9	Craniofacial development protein 1	CFDP1	Homo sapiens	33.592	4	4	1308327	153832
6119	1	Q9UFF9	Q9UFF9	CCR4-NOT transcription complex subunit 8	CNOT8	Homo sapiens	33.541	4	4	220082	44546
6120	1	Q9UGI6	Q9UGI6	Small conductance calcium-activated potassium channel protein 3	KCNN3	Homo sapiens	81.388	6	4	442332	0
6121	1	Q9UGM5	Q9UGM5	Fetuin-B	FETUB	Homo sapiens	42.055	4	4	91023	1038148
6122	1	Q9UHC6	Q9UHC6	Contactin-associated protein-like 2	CNTNAP2	Homo sapiens	148.17	4	4	119786	0
6123	1	Q9UHF7	Q9UHF7	Zinc finger transcription factor Trps1	TRPS1	Homo sapiens	141.52	4	4	457824	0
6124	1	Q9UHV9	Q9UHV9	Prefoldin subunit 2	PFDN2	Homo sapiens	16.647	4	4	6266040	2931537
6125	1	Q9UHY8	Q9UHY8	Fasciculation and elongation protein zeta-2	FEZ2	Homo sapiens	39.669	4	4	421857	38882
6126	1	Q9UI47	Q9UI47	Catenin alpha-3	CTNNA3	Homo sapiens	99.812	4	4	149281	0
6127	1	Q9UIA0	Q9UIA0	Cytohesin-4	CYTH4	Homo sapiens	45.672	4	4	167167	1586781
6128	1	Q9UIB8	Q9UIB8	SLAM family member 5	CD84	Homo sapiens	38.783	4	4	112440	285500
6129	1	Q9UJ68	Q9UJ68	Mitochondrial peptide methionine sulfoxide reductase	MSRA	Homo sapiens	26.131	4	4	1732120	1750370
6130	1	Q9UJJ9	Q9UJJ9	N-acetylglucosamine-1-phosphotransferase subunit gamma	GNPTG	Homo sapiens	33.974	4	4	673503	628407
6131	1	Q9UJP4	Q9UJP4	Kelch-like protein 21	KLHL21	Homo sapiens	66.616	4	4	181789	0
6132	1	Q9UK22	Q9UK22	F-box only protein 2	FBXO2	Homo sapiens	33.326	4	4	365708	507041
6133	1	Q9UK39	Q9UK39	Nocturnin	NOCT	Homo sapiens	48.197	4	4	0	220646
6134	1	Q9UK76	Q9UK76	Jupiter microtubule associated homolog 1	JPT1	Homo sapiens	16.014	4	4	1772977	1911767
6135	1	Q9UKA4	Q9UKA4	A-kinase anchor protein 11	AKAP11	Homo sapiens	210.52	4	4	517396	0
6136	1	Q9UKV5	Q9UKV5	E3 ubiquitin-protein ligase AMFR	AMFR	Homo sapiens	72.995	4	4	497675	0
6137	1	Q9UKY1	Q9UKY1	Zinc fingers and homeoboxes protein 1	ZHX1	Homo sapiens	98.101	4	4	80280	229942
6138	1	Q9UL12	Q9UL12	Sarcosine dehydrogenase, mitochondrial	SARDH	Homo sapiens	101.039	4	4	135807	0
6139	1	Q9ULI0	Q9ULI0	ATPase family AAA domain-containing protein 2B	ATAD2B	Homo sapiens	164.918	5	4	95685	50288
6140	1	Q9ULM3	Q9ULM3	YEATS domain-containing protein 2	YEATS2	Homo sapiens	150.784	4	4	225119	0
6141	1	Q9ULX3	Q9ULX3	RNA-binding protein NOB1	NOB1	Homo sapiens	46.674	4	4	279820	136022
6142	1	Q9UMS0	Q9UMS0	NFU1 iron-sulfur cluster scaffold homolog, mitochondrial	NFU1	Homo sapiens	28.461	4	4	2779690	614317

6143	1	Q9UMX1	Q9UMX1	Suppressor of fused homolog	SUFU	Homo sapiens	53.947	4	4	196752	0
6144	1	Q9UN76	Q9UN76	Sodium- and chloride-dependent neutral and basic amino acid transporter B(0+)	SLC6A14	Homo sapiens	72.155	4	4	0	1155131
6145	1	Q9UNN8	Q9UNN8	Endothelial protein C receptor	PROCR	Homo sapiens	26.669	4	4	2265930	164836
6146	1	Q9UNY4	Q9UNY4	Transcription termination factor 2	TTF2	Homo sapiens	129.59	4	4	135764	0
6147	1	Q9UQ03	Q9UQ03	Coronin-2B	CORO2B	Homo sapiens	54.954	4	4	642803	0
6148	1	Q9UQL6	Q9UQL6	Histone deacetylase 5	HDAC5	Homo sapiens	121.978	4	4	145971	0
6149	1	Q9Y282	Q9Y282	Endoplasmic reticulum-Golgi intermediate compartment protein 3	ERGIC3	Homo sapiens	43.221	4	4	2117223	444440
6150	1	Q9Y291	Q9Y291	28S ribosomal protein S33, mitochondrial	MRPS33	Homo sapiens	12.626	4	4	1173516	155984
6151	1	Q9Y2B5	Q9Y2B5	VPS9 domain-containing protein 1	VPS9D1	Homo sapiens	68.957	4	4	169303	0
6152	1	Q9Y2C4	Q9Y2C4	Nuclease EXOG, mitochondrial	EXOG	Homo sapiens	41.084	4	4	337068	63077
6153	1	Q9Y2D0	Q9Y2D0	Carbonic anhydrase 5B, mitochondrial	CA5B	Homo sapiens	36.434	4	4	928992	69914
6154	1	Q9Y2P8	Q9Y2P8	RNA 3'-terminal phosphate cyclase-like protein	RCL1	Homo sapiens	40.842	4	4	422892	72143
6155	1	Q9Y2R0	Q9Y2R0	Cytochrome c oxidase assembly factor 3 homolog, mitochondrial	COA3	Homo sapiens	11.731	4	4	5218580	1265269
6156	1	Q9Y2V2	Q9Y2V2	Calcium-regulated heat-stable protein 1	CARHSP1	Homo sapiens	15.891	4	4	2839939	4445123
6157	1	Q9Y2X9	Q9Y2X9	Zinc finger protein 281	ZNF281	Homo sapiens	96.916	4	4	456598	380248
6158	1	Q9Y324	Q9Y324	rRNA-processing protein FCF1 homolog	FCF1	Homo sapiens	23.369	4	4	253744	68601
6159	1	Q9Y3A3	Q9Y3A3	MOB-like protein phocein	MOB4	Homo sapiens	26.031	4	4	4045151	3610502
6160	1	Q9Y3A6	Q9Y3A6	Transmembrane emp24 domain-containing protein 5	TMED5	Homo sapiens	26.005	4	4	2805618	2269434
6161	1	Q9Y3C6	Q9Y3C6	Peptidyl-prolyl cis-trans isomerase-like 1	PPIL1	Homo sapiens	18.235	4	4	4448247	1634880
6162	1	Q9Y3D2	Q9Y3D2	Methionine-R-sulfoxide reductase B2, mitochondrial	MSRB2	Homo sapiens	19.534	4	4	1860763	389242
6163	1	Q9Y3D3	Q9Y3D3	28S ribosomal protein S16, mitochondrial	MRPS16	Homo sapiens	15.345	4	4	1126689	215630
6164	1	Q9Y3D6	Q9Y3D6	Mitochondrial fission 1 protein	FIS1	Homo sapiens	16.938	4	4	2821925	889238
6165	1	Q9Y3D8	Q9Y3D8	Adenylate kinase isoenzyme 6	AK6	Homo sapiens	20.062	4	4	591963	51023
6166	1	Q9Y3E1	Q9Y3E1	Hepatoma-derived growth factor-related protein 3	HDGFL3	Homo sapiens	22.618	4	4	3448225	71544
6167	1	Q9Y3E5	Q9Y3E5	Peptidyl-tRNA hydrolase 2, mitochondrial	PTRH2	Homo sapiens	19.193	4	4	677487	325354
6168	1	Q9Y3T6	Q9Y3T6	R3H and coiled-coil domain-containing protein 1	R3HCC1	Homo sapiens	49.091	4	4	263972	17527
6169	1	Q9Y3U8	Q9Y3U8	60S ribosomal protein L36	RPL36	Homo sapiens	12.251	4	4	4204236	905021
6170	1	Q9Y3Y2	Q9Y3Y2	Chromatin target of PRMT1 protein	CHTOP	Homo sapiens	26.394	4	4	2864571	661076
6171	1	Q9Y4P1	Q9Y4P1	Cysteine protease ATG4B	ATG4B	Homo sapiens	44.293	4	4	544248	190264
6172	1	Q9Y5L4	Q9Y5L4	Mitochondrial import inner membrane translocase subunit Tim13	TIMM13	Homo sapiens	10.502	4	4	5409022	1079753
6173	1	Q9Y5Q6	Q9Y5Q6	Insulin-like peptide INSL5	INSL5	Homo sapiens	15.332	4	4	1327715	0
6174	1	Q9Y5R8	Q9Y5R8	Trafficking protein particle complex subunit 1	TRAPPC1	Homo sapiens	16.832	4	4	769733	654686
6175	1	Q9Y5S8	Q9Y5S8	NADPH oxidase 1	NOX1	Homo sapiens	64.873	4	4	116498	59747
6176	1	Q9Y5U2	Q9Y5U2	Protein TSSC4	TSSC4	Homo sapiens	34.327	4	4	624302	35874
6177	1	Q9Y6B6	Q9Y6B6	GTP-binding protein SAR1b	SAR1B	Homo sapiens	22.41	4	4	1716335	931336
6178	1	Q9Y6B7	Q9Y6B7	AP-4 complex subunit beta-1	AP4B1	Homo sapiens	83.26	4	4	156131	0
6179	1	Q9Y6X3	Q9Y6X3	MAU2 chromatid cohesion factor homolog	MAU2	Homo sapiens	69.083	4	4	274526	39250
6180	1	A0A075B6J9	A0A075B6J9	Immunoglobulin lambda variable 2-18	IGLV2-18	Homo sapiens	12.41	3	3	2108036	657670
6181	2	A0A075B6R9;A0A(	A0A075B6R9	Probable non-functional immunoglobulin kappa variable 2D-24	IGKV2D-24	Homo sapiens	13.076	3	3	10793880	13416951
6182	1	A0A075B7D0	A0A075B7D0	Immunoglobulin heavy variable 1/OR15-1 (non-functional) (Fragment)	IGHV1OR15-1	Homo sapiens	13.01	3	3	5434673	341969
6183	1	A0A0A0MS15	A0A0A0MS15	Immunoglobulin heavy variable 3-49	IGHV3-49	Homo sapiens	13.056	6	3	6935000	10536970
6184	1	A0A0B4J1Y8	A0A0B4J1Y8	Immunoglobulin lambda variable 9-49	IGLV9-49	Homo sapiens	13.022	3	3	1448974	3410269
6185	1	A0A0C4DH29	A0A0C4DH29	Immunoglobulin heavy variable 1-3	IGHV1-3	Homo sapiens	13.004	3	3	898452	841998
6186	1	A0A0C4DH31	A0A0C4DH31	Immunoglobulin heavy variable 1-18	IGHV1-18	Homo sapiens	12.817	3	3	824137	1793278
6187	1	A1Z1Q3	A1Z1Q3	ADP-ribose glycohydrolase MACROD2	MACROD2	Homo sapiens	47.421	3	3	154104	0
6188	1	A2A2Y4	A2A2Y4	FERM domain-containing protein 3	FRMD3	Homo sapiens	68.774	3	3	242143	0
6189	1	A2AJT9	A2AJT9	BCLAF1 and THRAP3 family member 3	BCLAF3	Homo sapiens	83.874	3	3	151114	33507

6190	1	A4D161	A4D161	Protein FAM221A	FAM221A	Homo sapiens	33.081	3	3	116982	0
6191	1	A6NDU8	A6NDU8	RAB7A-interacting MON1-CCZ1 complex subunit 1	RIMOC1	Homo sapiens	33.622	3	3	1198403	567741
6192	1	A6NHX0	A6NHX0	Cytosolic arginine sensor for mTORC1 subunit 2	CASTOR2	Homo sapiens	36.055	3	3	212745	59289
6193	1	A6NI79	A6NI79	Coiled-coil domain-containing protein 69	CCDC69	Homo sapiens	34.798	3	3	175225	0
6194	2	A6NIZ1;P61224	A6NIZ1	Ras-related protein Rap-1b-like protein	RAP1BL	Homo sapiens	20.924	3	3	16114241	19245339
6195	1	A6NJ78	A6NJ78	12S rRNA N4-methylcytidine (m4C) methyltransferase	METTL15	Homo sapiens	46.123	4	3	584286	0
6196	1	B6A8C7	B6A8C7	T-cell-interacting, activating receptor on myeloid cells protein 1	TARM1	Homo sapiens	29.474	3	3	0	1289086
6197	1	B7ZBB8	B7ZBB8	Protein phosphatase 1 regulatory subunit 3G	PPP1R3G	Homo sapiens	38.017	3	3	288624	194776
6198	1	C9JLW8	C9JLW8	Mapk-regulated corepressor-interacting protein 1	MCRIP1	Homo sapiens	10.918	3	3	1279835	82101
6199	1	O00115	O00115	Deoxyribonuclease-2-alpha	DNASE2	Homo sapiens	39.581	3	3	318240	212240
6200	1	O00165	O00165	HCLS1-associated protein X-1	HAX1	Homo sapiens	31.618	3	3	545729	0
6201	1	O00193	O00193	Small acidic protein	SMAP	Homo sapiens	20.333	3	3	369257	389168
6202	1	O00244	O00244	Copper transport protein ATOX1	ATOX1	Homo sapiens	7.398	3	3	1368274	635020
6203	1	O00338	O00338	Sulfotransferase 1C2	SULT1C2	Homo sapiens	34.881	3	3	333262	0
6204	1	O00399	O00399	Dynactin subunit 6	DCTN6	Homo sapiens	20.747	3	3	1131437	293760
6205	1	O00422	O00422	Histone deacetylase complex subunit SAP18	SAP18	Homo sapiens	17.557	3	3	561536	224633
6206	1	O00451	O00451	GDNF family receptor alpha-2	GFRA2	Homo sapiens	51.545	3	3	230108	0
6207	1	O00478	O00478	Butyrophilin subfamily 3 member A3	BTN3A3	Homo sapiens	65.002	5	3	163684	157619
6208	1	O00483	O00483	Cytochrome c oxidase subunit NDUFA4	NDUFA4	Homo sapiens	9.368	3	3	1548856	377369
6209	1	O00505	O00505	Importin subunit alpha-4	KPNA3	Homo sapiens	57.809	3	3	1666991	708201
6210	1	O14524	O14524	Nuclear envelope integral membrane protein 1	NEMP1	Homo sapiens	50.641	3	3	617416	0
6211	1	O14530	O14530	Thioredoxin domain-containing protein 9	TXNDC9	Homo sapiens	26.533	3	3	316304	76475
6212	1	O14548	O14548	Cytochrome c oxidase subunit 7A-related protein, mitochondrial	COX7A2L	Homo sapiens	12.614	3	3	1968549	0
6213	1	O14561	O14561	Acyl carrier protein, mitochondrial	NDUFAB1	Homo sapiens	17.415	3	3	3164423	1118658
6214	1	O14613	O14613	Cdc42 effector protein 2	CDC42EP2	Homo sapiens	22.482	3	3	491041	0
6215	1	O14618	O14618	Copper chaperone for superoxide dismutase	CCS	Homo sapiens	29.037	3	3	3014268	1760058
6216	1	O14662	O14662	Syntaxin-16	STX16	Homo sapiens	37.031	3	3	359155	148315
6217	1	O14683	O14683	Tumor protein p53-inducible protein 11	TP53I11	Homo sapiens	21.052	3	3	2150630	1676972
6218	1	O14756	O14756	17-beta-hydroxysteroid dehydrogenase type 6	HSD17B6	Homo sapiens	35.967	3	3	169473	25037
6219	1	O14763	O14763	Tumor necrosis factor receptor superfamily member 10B	TNFRSF10B	Homo sapiens	47.879	3	3	0	412050
6220	1	O14880	O14880	Microsomal glutathione S-transferase 3	MGST3	Homo sapiens	16.516	3	3	7593846	923181
6221	1	O14907	O14907	Tax1-binding protein 3	TAX1BP3	Homo sapiens	13.734	3	3	6112673	384394
6222	1	O14924	O14924	Regulator of G-protein signaling 12	RGS12	Homo sapiens	156.359	3	3	64326	0
6223	1	O14933	O14933	Ubiquitin/ISG15-conjugating enzyme E2 L6	UBE2L6	Homo sapiens	17.768	3	3	90841	41093
6224	1	O15033	O15033	Apoptosis-resistant E3 ubiquitin protein ligase 1	AREL1	Homo sapiens	94.221	3	3	13176	99640
6225	1	O15121	O15121	Sphingolipid delta(4)-desaturase DES1	DEGS1	Homo sapiens	37.867	3	3	584557	1420964
6226	1	O15156	O15156	Zinc finger and BTB domain-containing protein 7B	ZBTB7B	Homo sapiens	58.026	3	3	87970	129536
6227	1	O15162	O15162	Phospholipid scramblase 1	PLSCR1	Homo sapiens	35.049	3	3	836189	4344139
6228	1	O15446	O15446	DNA-directed RNA polymerase I subunit RPA34	POLR1G	Homo sapiens	54.987	3	3	470474	72593
6229	1	O15504	O15504	Nucleoporin NUP42	NUP42	Homo sapiens	44.872	3	3	159094	0
6230	1	O15514	O15514	DNA-directed RNA polymerase II subunit RPB4	POLR2D	Homo sapiens	16.312	3	3	483908	139892
6231	1	O15519	O15519	CASP8 and FADD-like apoptosis regulator	CFLAR	Homo sapiens	55.345	3	3	38885	58041
6232	1	O15525	O15525	Transcription factor MafG	MAFG	Homo sapiens	17.848	4	3	389627	961696
6233	1	O15541	O15541	E3 ubiquitin-protein ligase RNF113A	RNF113A	Homo sapiens	38.787	3	3	455438	252881
6234	1	O43155	O43155	Leucine-rich repeat transmembrane protein FLRT2	FLRT2	Homo sapiens	74.05	3	3	77833	0
6235	1	O43236	O43236	Septin-4	SEPTIN4	Homo sapiens	55.099	3	3	167301	0
6236	1	O43303	O43303	Centriolar coiled-coil protein of 110 kDa	CCP110	Homo sapiens	113.425	3	3	251749	0
6237	1	O43325	O43325	LYR motif-containing protein 1	LYRM1	Homo sapiens	14.277	3	3	175981	0

6238	1 O43353	O43353	Receptor-interacting serine/threonine-protein kinase 2	RIPK2	Homo sapiens	61.197	3	3	260555	200062
6239	1 O43414	O43414	ERI1 exoribonuclease 3	ERI3	Homo sapiens	37.237	3	3	398099	0
6240	1 O43505	O43505	Beta-1,4-glucuronyltransferase 1	B4GAT1	Homo sapiens	47.117	3	3	298579	21842
6241	1 O43567	O43567	E3 ubiquitin-protein ligase RNF13	RNF13	Homo sapiens	42.812	3	3	118092	499544
6242	1 O43581	O43581	Synaptotagmin-7	SYT7	Homo sapiens	45.502	3	3	309975	0
6243	1 O43598	O43598	2'-deoxynucleoside 5'-phosphate N-hydrolase 1	DNPH1	Homo sapiens	19.109	3	3	5005591	939061
6244	1 O43617	O43617	Trafficking protein particle complex subunit 3	TRAPPC3	Homo sapiens	20.275	3	3	1343120	1242572
6245	1 O43633	O43633	Charged multivesicular body protein 2a	CHMP2A	Homo sapiens	25.104	3	3	1968399	1511192
6246	1 O43657	O43657	Tetraspanin-6	TSPAN6	Homo sapiens	27.564	3	3	692681	56062
6247	1 O43678	O43678	NADH dehydrogenase [ubiquinone] 1 alpha subcomplex subunit 2	NDUFA2	Homo sapiens	10.921	3	3	5169007	842303
6248	1 O43688	O43688	Phospholipid phosphatase 2	PLPP2	Homo sapiens	32.574	3	3	677758	234839
6249	1 O43734	O43734	E3 ubiquitin ligase TRAF3IP2	TRAF3IP2	Homo sapiens	64.666	3	3	169249	27686
6250	1 O43760	O43760	Synaptogyrin-2	SYNGR2	Homo sapiens	24.81	3	3	2700034	4300392
6251	1 O43766	O43766	Lipoyl synthase, mitochondrial	LIAS	Homo sapiens	41.913	3	3	229617	0
6252	1 O43809	O43809	Cleavage and polyadenylation specificity factor subunit 5	NUDT21	Homo sapiens	26.228	3	3	2010691	1014830
6253	1 O43819	O43819	Protein SCO2 homolog, mitochondrial	SCO2	Homo sapiens	29.808	3	3	478192	448534
6254	1 O43924	O43924	Retinal rod rhodopsin-sensitive cGMP 3',5'-cyclic phosphodiesterase subunit delta	PDE6D	Homo sapiens	17.42	3	3	1087610	532341
6255	1 O60220	O60220	Mitochondrial import inner membrane translocase subunit Tim8 A	TIMM8A	Homo sapiens	10.996	3	3	352716	67428
6256	1 O60260	O60260	E3 ubiquitin-protein ligase parkin	PRKN	Homo sapiens	51.64	3	3	63285	0
6257	1 O60293	O60293	Zinc finger C3H1 domain-containing protein	ZFC3H1	Homo sapiens	226.36	3	3	68594	19032
6258	1 O60303	O60303	Katanin-interacting protein	KATNIP	Homo sapiens	180.922	3	3	128338	0
6259	1 O60487	O60487	Myelin protein zero-like protein 2	MPZL2	Homo sapiens	24.483	3	3	135846	447793
6260	1 O60609	O60609	GDNF family receptor alpha-3	GFRA3	Homo sapiens	44.509	3	3	635646	0
6261	1 O60613	O60613	Selenoprotein F	SELENOF	Homo sapiens	18.063	3	3	1367225	893127
6262	1 O60725	O60725	Protein-S-isoprenylcysteine O-methyltransferase	ICMT	Homo sapiens	31.937	3	3	725431	433881
6263	1 O60783	O60783	28S ribosomal protein S14, mitochondrial	MRPS14	Homo sapiens	15.138	3	3	1133220	44328
6264	1 O60869	O60869	Endothelial differentiation-related factor 1	EDF1	Homo sapiens	16.368	3	3	6886084	2511407
6265	1 O60925	O60925	Prefoldin subunit 1	PFDN1	Homo sapiens	14.208	3	3	1534249	559021
6266	1 O60927	O60927	E3 ubiquitin-protein ligase PPP1R11	PPP1R11	Homo sapiens	13.952	3	3	410853	0
6267	1 O75022	O75022	Leukocyte immunoglobulin-like receptor subfamily B member 3	LILRB3	Homo sapiens	69.385	5	3	57745	2075693
6268	1 O75030	O75030	Microphthalmia-associated transcription factor	MITF	Homo sapiens	58.795	3	3	172215	0
6269	1 O75037	O75037	Kinesin-like protein KIF21B	KIF21B	Homo sapiens	182.664	3	3	0	91998
6270	1 O75056	O75056	Syndecan-3	SDC3	Homo sapiens	45.496	3	3	1116783	0
6271	1 O75173	O75173	A disintegrin and metalloproteinase with thrombospondin motifs 4	ADAMTS4	Homo sapiens	90.197	3	3	53927	132422
6272	1 O75182	O75182	Paired amphipathic helix protein Sin3b	SIN3B	Homo sapiens	133.068	3	3	263860	49668
6273	1 O75251	O75251	NADH dehydrogenase [ubiquinone] iron-sulfur protein 7, mitochondrial	NDUFS7	Homo sapiens	23.564	3	3	11842350	1829814
6274	1 O75352	O75352	Mannose-P-dolichol utilization defect 1 protein	MPDU1	Homo sapiens	26.639	3	3	1523905	1702538
6275	1 O75379	O75379	Vesicle-associated membrane protein 4	VAMP4	Homo sapiens	16.394	3	3	89711	119329
6276	1 O75628	O75628	GTP-binding protein REM 1	REM1	Homo sapiens	32.947	3	3	265204	0
6277	1 O75695	O75695	Protein XRP2	RP2	Homo sapiens	39.641	3	3	1381232	3650709
6278	1 O75817	O75817	Ribonuclease P protein subunit p20	POP7	Homo sapiens	15.649	3	3	411714	28436
6279	1 O75871	O75871	Carcinoembryonic antigen-related cell adhesion molecule 4	CEACAM4	Homo sapiens	25.909	3	3	0	587181
6280	1 O75881	O75881	Cytochrome P450 7B1	CYP7B1	Homo sapiens	58.256	3	3	289816	0
6281	1 O75888	O75888	Tumor necrosis factor ligand superfamily member 13	TNFSF13	Homo sapiens	27.432	3	3	196159	399332
6282	1 O75940	O75940	Survival of motor neuron-related-splicing factor 30	SMNDC1	Homo sapiens	26.709	3	3	516093	264696
6283	1 O75943	O75943	Cell cycle checkpoint protein RAD17	RAD17	Homo sapiens	77.055	3	3	141245	0
6284	1 O75964	O75964	ATP synthase subunit g, mitochondrial	ATP5MG	Homo sapiens	11.427	3	3	14037149	3842867

6285	1	O76041	O76041	Nebulette	NEBL	Homo sapiens	116.456	3	3	376526	0
6286	1	O94762	O94762	ATP-dependent DNA helicase Q5	RECQL5	Homo sapiens	108.861	3	3	114882	0
6287	1	O94811	O94811	Tubulin polymerization-promoting protein	TPPP	Homo sapiens	23.693	3	3	1572127	19005
6288	1	O94929	O94929	Actin-binding LIM protein 3	ABLIM3	Homo sapiens	77.802	3	3	88904	0
6289	1	O94952	O94952	F-box only protein 21	FBXO21	Homo sapiens	72.272	3	3	142729	0
6290	1	O94992	O94992	Protein HEXIM1	HEXIM1	Homo sapiens	40.621	3	3	324163	16858
6291	1	O95070	O95070	Protein YIF1A	YIF1A	Homo sapiens	32.01	3	3	688993	143574
6292	1	O95084	O95084	Serine protease 23	PRSS23	Homo sapiens	43.002	3	3	230917	409612
6293	1	O95104	O95104	SR-related and CTD-associated factor 4	SCAF4	Homo sapiens	125.871	3	3	536841	67769
6294	1	O95139	O95139	NADH dehydrogenase [ubiquinone] 1 beta subcomplex subunit 6	NDUFB6	Homo sapiens	15.489	3	3	1593827	328341
6295	1	O95149	O95149	Snurportin-1	SNUPN	Homo sapiens	41.142	3	3	451382	154071
6296	1	O95199	O95199	RCC1 and BTB domain-containing protein 2	RCBTB2	Homo sapiens	60.314	3	3	261670	62006
6297	1	O95365	O95365	Zinc finger and BTB domain-containing protein 7A	ZBTB7A	Homo sapiens	61.442	3	3	383557	0
6298	1	O95372	O95372	Acyl-protein thioesterase 2	LYPLA2	Homo sapiens	24.737	3	3	1350355	553798
6299	1	O95379	O95379	Tumor necrosis factor alpha-induced protein 8	TNFAIP8	Homo sapiens	23.003	3	3	430068	250150
6300	1	O95476	O95476	CTD nuclear envelope phosphatase 1	CTDNEP1	Homo sapiens	28.378	3	3	682009	388581
6301	1	O95639	O95639	Cleavage and polyadenylation specificity factor subunit 4	CPSF4	Homo sapiens	30.255	3	3	302508	52647
6302	1	O95835	O95835	Serine/threonine-protein kinase LATS1	LATS1	Homo sapiens	126.87	3	3	106740	0
6303	1	O95999	O95999	B-cell lymphoma/leukemia 10	BCL10	Homo sapiens	26.252	3	3	705798	628319
6304	1	O96007	O96007	Molybdopterin synthase catalytic subunit	MOCS2	Homo sapiens	20.943	3	3	528921	0
6305	1	O96009	O96009	Napsin-A	NAPSA	Homo sapiens	45.386	3	3	0	427081
6306	1	P00326	P00326	Alcohol dehydrogenase 1C	ADH1C	Homo sapiens	39.867	4	3	8165284	185741
6307	1	P01040	P01040	Cystatin-A	CSTA	Homo sapiens	11.005	3	3	676444	10223244
6308	1	P01111	P01111	GTPase NRas	NRAS	Homo sapiens	21.228	8	3	757124	613325
6309	1	P01880	P01880	Immunoglobulin heavy constant delta	IGHD	Homo sapiens	42.35	3	3	71803	652280
6310	1	P01911	P01911	HLA class II histocompatibility antigen, DRB1 beta chain	HLA-DRB1	Homo sapiens	29.966	6	3	0	1424785
6311	1	P02144	P02144	Myoglobin	MB	Homo sapiens	17.183	3	3	1577899	518093
6312	1	P02654	P02654	Apolipoprotein C-I	APOC1	Homo sapiens	9.33	3	3	1206495	4088918
6313	1	P02655	P02655	Apolipoprotein C-II	APOC2	Homo sapiens	11.283	3	3	205215	977427
6314	1	P02753	P02753	Retinol-binding protein 4	RBP4	Homo sapiens	23.01	6	3	1321353	2777099
6315	1	P02765	P02765	Alpha-2-HS-glycoprotein	AHSG	Homo sapiens	39.342	5	3	16059576	24737510
6316	1	P04216	P04216	Thy-1 membrane glycoprotein	THY1	Homo sapiens	17.934	3	3	25305680	7723385
6317	1	P04279	P04279	Semenogelin-1	SEMG1	Homo sapiens	52.131	4	3	14571	52338
6318	1	P06241	P06241	Tyrosine-protein kinase Fyn	FYN	Homo sapiens	60.762	5	3	283191	174077
6319	1	P06731	P06731	Carcinoembryonic antigen-related cell adhesion molecule 5	CEACAM5	Homo sapiens	76.795	3	3	1271195	153268
6320	1	P07311	P07311	Acylphosphatase-1	ACYP1	Homo sapiens	11.258	3	3	1506398	526980
6321	1	P07910	P07910	Heterogeneous nuclear ribonucleoproteins C1/C2	HNRNPC	Homo sapiens	33.669	8	3	19892101	10169713
6322	1	P08172	P08172	Muscarinic acetylcholine receptor M2	CHRM2	Homo sapiens	51.719	3	3	388788	0
6323	1	P08754	P08754	Guanine nucleotide-binding protein G(i) subunit alpha-3	GNAI3	Homo sapiens	40.534	3	3	1620141	3386079
6324	1	P08913	P08913	Alpha-2A adrenergic receptor	ADRA2A	Homo sapiens	50.647	3	3	137800	0
6325	1	P09012	P09012	U1 small nuclear ribonucleoprotein A	SNRPA	Homo sapiens	31.279	3	3	1337945	289022
6326	1	P09758	P09758	Tumor-associated calcium signal transducer 2	TACSTD2	Homo sapiens	35.707	3	3	0	194574
6327	1	P0C0L5	P0C0L5	Complement C4-B	C4B_2	Homo sapiens	192.754	64	3	6755970	12886838
6328	2	P0C0S5;Q71UI9	P0C0S5	Histone H2A.Z	H2AZ1	Homo sapiens	13.552	3	3	7624800	5023676
6329	1	P0C2W1	P0C2W1	F-box/SPRY domain-containing protein 1	FBXO45	Homo sapiens	30.634	3	3	252896	163779
6330	1	P0C7P0	P0C7P0	CDGSH iron-sulfur domain-containing protein 3, mitochondrial	CISD3	Homo sapiens	14.213	3	3	966509	0
6331	2	P0DI81;P0DI82	P0DI81	Trafficking protein particle complex subunit 2	TRAPPC2	Homo sapiens	16.444	3	3	577707	109289
6332	1	P0DPB6	P0DPB6	DNA-directed RNA polymerases I and III subunit RPAC2	POLR1D	Homo sapiens	15.239	3	3	745791	47131

6333	1	P10124	P10124	Serglycin	SRGN	Homo sapiens	17.651	3	3	162898	2458299
6334	1	P10599	P10599	Thioredoxin	TXN	Homo sapiens	11.737	3	3	100260017	50659950
6335	1	P10620	P10620	Microsomal glutathione S-transferase 1	MGST1	Homo sapiens	17.597	3	3	5085260	3760931
6336	1	P10646	P10646	Tissue factor pathway inhibitor	TFPI	Homo sapiens	35.016	3	3	0	213864
6337	1	P10721	P10721	Mast/stem cell growth factor receptor Kit	KIT	Homo sapiens	109.866	3	3	137298	0
6338	1	P11308	P11308	Transcriptional regulator ERG	ERG	Homo sapiens	53.836	3	3	0	131802
6339	1	P11473	P11473	Vitamin D3 receptor	VDR	Homo sapiens	48.289	3	3	135738	0
6340	1	P11908	P11908	Ribose-phosphate pyrophosphokinase 2	PRPS2	Homo sapiens	34.769	4	3	2341527	1132543
6341	1	P12104	P12104	Fatty acid-binding protein, intestinal	FABP2	Homo sapiens	15.205	3	3	6764622	63782
6342	1	P12314	P12314	High affinity immunoglobulin gamma Fc receptor I	FCGR1A	Homo sapiens	42.63	10	3	161807	1237731
6343	1	P13473	P13473	Lysosome-associated membrane glycoprotein 2	LAMP2	Homo sapiens	44.962	3	3	1135265	1906244
6344	1	P13686	P13686	Tartrate-resistant acid phosphatase type 5	ACP5	Homo sapiens	36.599	3	3	619999	285763
6345	1	P13987	P13987	CD59 glycoprotein	CD59	Homo sapiens	14.176	3	3	9223948	5355626
6346	1	P14091	P14091	Cathepsin E	CTSE	Homo sapiens	42.794	3	3	357638	0
6347	1	P14174	P14174	Macrophage migration inhibitory factor	MIF	Homo sapiens	12.475	3	3	10572457	4513280
6348	1	P14207	P14207	Folate receptor beta	FOLR2	Homo sapiens	29.276	5	3	1023280	141239
6349	1	P14316	P14316	Interferon regulatory factor 2	IRF2	Homo sapiens	39.353	4	3	250711	59996
6350	1	P14373	P14373	Zinc finger protein RFP	TRIM27	Homo sapiens	58.49	3	3	114716	0
6351	1	P14621	P14621	Acylphosphatase-2	ACYP2	Homo sapiens	11.139	3	3	849467	343923
6352	1	P15259	P15259	Phosphoglycerate mutase 2	PGAM2	Homo sapiens	28.767	3	3	508890	0
6353	1	P15260	P15260	Interferon gamma receptor 1	IFNGR1	Homo sapiens	54.405	3	3	111999	492749
6354	1	P15408	P15408	Fos-related antigen 2	FOSL2	Homo sapiens	35.193	3	3	425411	63796
6355	1	P15502	P15502	Elastin	ELN	Homo sapiens	68.395	3	3	1046656	0
6356	1	P16066	P16066	Atrial natriuretic peptide receptor 1	NPR1	Homo sapiens	118.921	3	3	108054	0
6357	1	P16260	P16260	Solute carrier family 25 member 16	SLC25A16	Homo sapiens	36.225	3	3	326633	0
6358	1	P16671	P16671	Platelet glycoprotein 4	CD36	Homo sapiens	53.054	3	3	486270	453922
6359	1	P17028	P17028	Zinc finger protein 24	ZNF24	Homo sapiens	42.154	3	3	168062	10043
6360	1	P17029	P17029	Zinc finger protein with KRAB and SCAN domains 1	ZKSCAN1	Homo sapiens	63.633	3	3	449220	0
6361	1	P17081	P17081	Rho-related GTP-binding protein RhoQ	RHOQ	Homo sapiens	22.657	3	3	244344	0
6362	1	P17405	P17405	Sphingomyelin phosphodiesterase	SMPD1	Homo sapiens	69.935	3	3	123908	12846
6363	1	P17900	P17900	Ganglioside GM2 activator	GM2A	Homo sapiens	20.839	3	3	2248457	1224349
6364	1	P18627	P18627	Lymphocyte activation gene 3 protein	LAG3	Homo sapiens	57.447	3	3	0	230161
6365	1	P19388	P19388	DNA-directed RNA polymerases I, II, and III subunit RPABC1	POLR2E	Homo sapiens	24.548	3	3	1395734	737363
6366	1	P19484	P19484	Transcription factor EB	TFEB	Homo sapiens	52.862	5	3	337677	188216
6367	1	P19835	P19835	Bile salt-activated lipase	CEL	Homo sapiens	79.322	3	3	0	600754
6368	1	P20061	P20061	Transcobalamin-1	TCN1	Homo sapiens	48.206	3	3	50640	1278079
6369	1	P20062	P20062	Transcobalamin-2	TCN2	Homo sapiens	47.537	3	3	0	399336
6370	1	P21730	P21730	C5a anaphylatoxin chemotactic receptor 1	C5AR1	Homo sapiens	39.336	3	3	25395	5079471
6371	1	P21757	P21757	Macrophage scavenger receptor types I and II	MSR1	Homo sapiens	49.764	3	3	17858	81356
6372	1	P21926	P21926	CD9 antigen	CD9	Homo sapiens	25.415	3	3	23605570	2153754
6373	1	P23083	P23083	Immunoglobulin heavy variable 1-2	IGHV1-2	Homo sapiens	13.082	3	3	2029368	3298545
6374	1	P23258	P23258	Tubulin gamma-1 chain	TUBG1	Homo sapiens	51.17	12	3	427690	198383
6375	1	P23276	P23276	Kell blood group glycoprotein	KEL	Homo sapiens	82.827	3	3	24125	96962
6376	1	P23471	P23471	Receptor-type tyrosine-protein phosphatase zeta	PTPRZ1	Homo sapiens	254.591	3	3	123862	0
6377	1	P23508	P23508	Colorectal mutant cancer protein	MCC	Homo sapiens	93.028	3	3	135761	0
6378	1	P24386	P24386	Rab proteins geranylgeranyltransferase component A 1	CHM	Homo sapiens	73.476	3	3	368678	369044
6379	1	P24394	P24394	Interleukin-4 receptor subunit alpha	IL4R	Homo sapiens	89.661	3	3	64459	146540
6380	1	P24534	P24534	Elongation factor 1-beta	EEF1B2	Homo sapiens	24.762	3	3	9538805	5031251

6381	1	P24593	P24593	Insulin-like growth factor-binding protein 5	IGFBP5	Homo sapiens	30.569	3	3	316602	26969
6382	1	P25105	P25105	Platelet-activating factor receptor	PTAFR	Homo sapiens	39.206	3	3	0	438001
6383	1	P25963	P25963	NF-kappa-B inhibitor alpha	NFKBIA	Homo sapiens	35.609	3	3	2551418	22292
6384	1	P26010	P26010	Integrin beta-7	ITGB7	Homo sapiens	86.902	3	3	188949	17195
6385	1	P26651	P26651	mRNA decay activator protein ZFP36	ZFP36	Homo sapiens	34.002	3	3	29261	386895
6386	1	P27930	P27930	Interleukin-1 receptor type 2	IL1R2	Homo sapiens	45.422	3	3	605272	65788
6387	1	P28072	P28072	Proteasome subunit beta type-6	PSMB6	Homo sapiens	25.357	3	3	13000440	4922544
6388	1	P28332	P28332	Alcohol dehydrogenase 6	ADH6	Homo sapiens	39.072	3	3	326876	28208
6389	1	P28347	P28347	Transcriptional enhancer factor TEF-1	TEAD1	Homo sapiens	47.945	4	3	270234	0
6390	1	P28702	P28702	Retinoic acid receptor RXR-beta	RXRB	Homo sapiens	56.922	3	3	243436	74344
6391	1	P29120	P29120	Neuroendocrine convertase 1	PCSK1	Homo sapiens	84.154	3	3	120145	0
6392	1	P29373	P29373	Cellular retinoic acid-binding protein 2	CRABP2	Homo sapiens	15.69	3	3	733905	443584
6393	1	P30679	P30679	Guanine nucleotide-binding protein subunit alpha-15	GNA15	Homo sapiens	43.568	3	3	16234	325945
6394	1	P30793	P30793	GTP cyclohydrolase 1	GCH1	Homo sapiens	27.903	3	3	13472	150534
6395	1	P32927	P32927	Cytokine receptor common subunit beta	CSF2RB	Homo sapiens	97.338	3	3	0	186878
6396	1	P33402	P33402	Guanylate cyclase soluble subunit alpha-2	GUCY1A2	Homo sapiens	81.753	3	3	408290	0
6397	1	P34741	P34741	Syndecan-2	SDC2	Homo sapiens	22.158	3	3	1576142	18404
6398	1	P34910	P34910	Protein EVI2B	EVI2B	Homo sapiens	48.668	3	3	178083	2398507
6399	1	P34947	P34947	G protein-coupled receptor kinase 5	GRK5	Homo sapiens	67.787	3	3	173820	0
6400	1	P35318	P35318	Pro-adrenomedullin	ADM	Homo sapiens	20.419	3	3	0	129404
6401	1	P35590	P35590	Tyrosine-protein kinase receptor Tie-1	TIE1	Homo sapiens	125.09	3	3	447302	273485
6402	1	P35869	P35869	Aryl hydrocarbon receptor	AHR	Homo sapiens	96.15	3	3	521361	13137
6403	1	P36873	P36873	Serine/threonine-protein phosphatase PP1-gamma catalytic subunit	PPP1CC	Homo sapiens	36.982	3	3	1053994	1141995
6404	1	P36980	P36980	Complement factor H-related protein 2	CFHR2	Homo sapiens	30.652	3	3	235598	991220
6405	1	P37840	P37840	Alpha-synuclein	SNCA	Homo sapiens	14.46	3	3	2778784	954651
6406	1	P38432	P38432	Coilin	COIL	Homo sapiens	62.61	3	3	124058	20565
6407	1	P38435	P38435	Vitamin K-dependent gamma-carboxylase	GGCX	Homo sapiens	87.563	3	3	197028	153423
6408	1	P40198	P40198	Carcinoembryonic antigen-related cell adhesion molecule 3	CEACAM3	Homo sapiens	27.092	3	3	0	196551
6409	1	P40199	P40199	Carcinoembryonic antigen-related cell adhesion molecule 6	CEACAM6	Homo sapiens	37.236	4	3	136608	9670180
6410	1	P40424	P40424	Pre-B-cell leukemia transcription factor 1	PBX1	Homo sapiens	46.626	3	3	560874	0
6411	1	P40429	P40429	60S ribosomal protein L13a	RPL13A	Homo sapiens	23.576	5	3	4328390	2188161
6412	1	P41208	P41208	Centrin-2	CETN2	Homo sapiens	19.738	4	3	445043	0
6413	1	P41229	P41229	Lysine-specific demethylase 5C	KDM5C	Homo sapiens	175.721	6	3	134112	0
6414	1	P41743	P41743	Protein kinase C iota type	PRKCI	Homo sapiens	68.265	3	3	425673	285194
6415	1	P42680	P42680	Tyrosine-protein kinase Tec	TEC	Homo sapiens	73.581	3	3	562099	135971
6416	1	P43166	P43166	Carbonic anhydrase 7	CA7	Homo sapiens	29.656	3	3	1050908	0
6417	1	P45844	P45844	ATP-binding cassette sub-family G member 1	ABCG1	Homo sapiens	75.594	3	3	11113	124277
6418	1	P46778	P46778	60S ribosomal protein L21	RPL21	Homo sapiens	18.564	3	3	17671961	4363607
6419	1	P46779	P46779	60S ribosomal protein L28	RPL28	Homo sapiens	15.746	3	3	14499588	5645440
6420	1	P47813	P47813	Eukaryotic translation initiation factor 1A, X-chromosomal	EIF1AX	Homo sapiens	16.462	5	3	963694	277087
6421	1	P48060	P48060	Glioma pathogenesis-related protein 1	GLIPR1	Homo sapiens	30.368	3	3	119334	230671
6422	1	P48539	P48539	Calmodulin regulator protein PCP4	PCP4	Homo sapiens	6.789	3	3	2550389	0
6423	1	P48730	P48730	Casein kinase I isoform delta	CSNK1D	Homo sapiens	47.331	8	3	423577	143645
6424	1	P49006	P49006	MARCKS-related protein	MARCKSL1	Homo sapiens	19.528	3	3	4305356	1047005
6425	1	P49184	P49184	Deoxyribonuclease-1-like 1	DNASE1L1	Homo sapiens	33.892	3	3	261749	885449
6426	1	P49427	P49427	Ubiquitin-conjugating enzyme E2 R1	CDC34	Homo sapiens	26.736	3	3	505468	61393
6427	1	P49447	P49447	Transmembrane ascorbate-dependent reductase CYB561	CYB561	Homo sapiens	27.558	3	3	399686	211845
6428	1	P49458	P49458	Signal recognition particle 9 kDa protein	SRP9	Homo sapiens	10.111	3	3	2447395	1611934

6429	1	P49643	P49643	DNA primase large subunit	PRIM2	Homo sapiens	58.807	3	3	106579	43366
6430	1	P49747	P49747	Cartilage oligomeric matrix protein	COMP	Homo sapiens	82.864	8	3	144539	1028543
6431	1	P49761	P49761	Dual specificity protein kinase CLK3	CLK3	Homo sapiens	73.515	3	3	149785	45660
6432	1	P49842	P49842	Serine/threonine-protein kinase 19	STK19	Homo sapiens	40.914	3	3	364090	0
6433	1	P50225	P50225	Sulfotransferase 1A1	SULT1A1	Homo sapiens	34.166	10	3	5282530	8126743
6434	1	P50226	P50226	Sulfotransferase 1A2	SULT1A2	Homo sapiens	34.31	3	3	603004	0
6435	1	P51790	P51790	H(+)/Cl(-) exchange transporter 3	CLCN3	Homo sapiens	90.966	4	3	184717	106171
6436	1	P51948	P51948	CDK-activating kinase assembly factor MAT1	MNAT1	Homo sapiens	35.822	3	3	348528	222575
6437	1	P51970	P51970	NADH dehydrogenase [ubiquinone] 1 alpha subcomplex subunit 8	NDUFA8	Homo sapiens	20.103	3	3	6898031	1521739
6438	1	P51993	P51993	4-galactosyl-N-acetylglucosaminide 3-alpha-L-fucosyltransferase FUT6	FUT6	Homo sapiens	41.86	3	3	258228	0
6439	1	P52294	P52294	Importin subunit alpha-5	KPNA1	Homo sapiens	60.222	3	3	618197	274005
6440	1	P52429	P52429	Diacylglycerol kinase epsilon	DGKE	Homo sapiens	63.924	3	3	162724	0
6441	1	P52799	P52799	Ephrin-B2	EFNB2	Homo sapiens	36.925	3	3	334173	0
6442	1	P54278	P54278	Mismatch repair endonuclease PMS2	PMS2	Homo sapiens	95.796	3	3	127870	0
6443	1	P54317	P54317	Pancreatic lipase-related protein 2	PNLIPRP2	Homo sapiens	51.96	3	3	176093	0
6444	1	P54753	P54753	Ephrin type-B receptor 3	EPHB3	Homo sapiens	110.33	7	3	364490	0
6445	1	P55008	P55008	Allograft inflammatory factor 1	AIF1	Homo sapiens	16.702	3	3	3456739	6115445
6446	1	P55042	P55042	GTP-binding protein RAD	RRAD	Homo sapiens	33.244	3	3	308940	0
6447	1	P55056	P55056	Apolipoprotein C-IV	APOC4	Homo sapiens	14.552	3	3	478218	1206785
6448	1	P55103	P55103	Inhibin beta C chain	INHBC	Homo sapiens	38.237	3	3	0	362790
6449	1	P55285	P55285	Cadherin-6	CDH6	Homo sapiens	88.308	3	3	182271	0
6450	1	P56277	P56277	Cx9C motif-containing protein 4	CMC4	Homo sapiens	7.743	3	3	398609	58517
6451	1	P56377	P56377	AP-1 complex subunit sigma-2	AP1S2	Homo sapiens	18.616	4	3	1580873	2589540
6452	1	P56556	P56556	NADH dehydrogenase [ubiquinone] 1 alpha subcomplex subunit 6	NDUFA6	Homo sapiens	15.136	3	3	1215669	140888
6453	1	P56589	P56589	Peroxisomal biogenesis factor 3	PEX3	Homo sapiens	42.139	3	3	136626	175688
6454	1	P57088	P57088	Transmembrane protein 33	TMEM33	Homo sapiens	27.978	3	3	2036967	1516603
6455	1	P60033	P60033	CD81 antigen	CD81	Homo sapiens	25.807	3	3	5582303	503438
6456	1	P60468	P60468	Protein transport protein Sec61 subunit beta	SEC61B	Homo sapiens	9.974	3	3	6648140	4514107
6457	1	P60520	P60520	Gamma-aminobutyric acid receptor-associated protein-like 2	GABARAPL2	Homo sapiens	13.664	4	3	2199975	1412008
6458	1	P60604	P60604	Ubiquitin-conjugating enzyme E2 G2	UBE2G2	Homo sapiens	18.565	3	3	400888	305756
6459	1	P60866	P60866	40S ribosomal protein S20	RPS20	Homo sapiens	13.369	3	3	9534646	4019752
6460	1	P60891	P60891	Ribose-phosphate pyrophosphokinase 1	PRPS1	Homo sapiens	34.834	12	3	329383	225603
6461	1	P61073	P61073	C-X-C chemokine receptor type 4	CXCR4	Homo sapiens	39.745	3	3	8873	61494
6462	1	P61225	P61225	Ras-related protein Rap-2b	RAP2B	Homo sapiens	20.504	5	3	1205664	1952255
6463	1	P61587	P61587	Rho-related GTP-binding protein RhoE	RND3	Homo sapiens	27.365	3	3	217069	0
6464	1	P61803	P61803	Dolichyl-diphosphooligosaccharide--protein glycosyltransferase subunit DAD1	DAD1	Homo sapiens	12.493	3	3	398749	376719
6465	1	P61916	P61916	NPC intracellular cholesterol transporter 2	NPC2	Homo sapiens	16.569	3	3	4587990	5440990
6466	1	P62633	P62633	CCHC-type zinc finger nucleic acid binding protein	CNBP	Homo sapiens	19.463	3	3	1524672	472723
6467	1	P62699	P62699	Protein yippee-like 5	YPEL5	Homo sapiens	13.841	3	3	414095	256278
6468	1	P62820	P62820	Ras-related protein Rab-1A	RAB1A	Homo sapiens	22.675	3	3	15584468	3564168
6469	1	P62834	P62834	Ras-related protein Rap-1A	RAP1A	Homo sapiens	20.987	8	3	509186	1574008
6470	1	P62841	P62841	40S ribosomal protein S15	RPS15	Homo sapiens	17.039	3	3	4891073	1752455
6471	1	P62847	P62847	40S ribosomal protein S24	RPS24	Homo sapiens	15.423	3	3	8062368	2263188
6472	1	P62851	P62851	40S ribosomal protein S25	RPS25	Homo sapiens	13.741	3	3	17930359	7618520
6473	1	P62861	P62861	FAU ubiquitin-like and ribosomal protein S30	FAU	Homo sapiens	14.389	3	3	13810395	3450888
6474	1	P62913	P62913	60S ribosomal protein L11	RPL11	Homo sapiens	20.253	3	3	14217284	7203950
6475	1	P62979	P62979	Ubiquitin-40S ribosomal protein S27a	RPS27A	Homo sapiens	17.965	8	3	3844690	473668

6476	1	P63000	P63000	Ras-related C3 botulinum toxin substrate 1	RAC1	Homo sapiens	21.45	4	3	19576851	10793715
6477	1	P63172	P63172	Dynein light chain Tctex-type 1	DYNLT1	Homo sapiens	12.452	3	3	1714596	1280656
6478	1	P63302	P63302	Selenoprotein W	SELENOW	Homo sapiens	9.416	3	3	816772	0
6479	1	P68543	P68543	UBX domain-containing protein 2A	UBXN2A	Homo sapiens	29.28	3	3	230428	0
6480	1	P78314	P78314	SH3 domain-binding protein 2	SH3BP2	Homo sapiens	62.245	3	3	185037	610253
6481	1	P78382	P78382	CMP-sialic acid transporter	SLC35A1	Homo sapiens	36.78	3	3	653244	53216
6482	1	P80511	P80511	Protein S100-A12	S100A12	Homo sapiens	10.575	3	3	374799	4457429
6483	1	P82909	P82909	Alpha-ketoglutarate dehydrogenase component 4	MRPS36	Homo sapiens	11.463	3	3	7550696	1032498
6484	1	P82912	P82912	28S ribosomal protein S11, mitochondrial	MRPS11	Homo sapiens	20.613	3	3	1321807	278432
6485	1	P82921	P82921	28S ribosomal protein S21, mitochondrial	MRPS21	Homo sapiens	10.686	3	3	864052	207535
6486	1	P82932	P82932	28S ribosomal protein S6, mitochondrial	MRPS6	Homo sapiens	14.225	3	3	884625	89473
6487	1	P83731	P83731	60S ribosomal protein L24	RPL24	Homo sapiens	17.778	3	3	16677430	4962998
6488	1	P84022	P84022	Mothers against decapentaplegic homolog 3	SMAD3	Homo sapiens	48.081	3	3	539521	42926
6489	1	P98173	P98173	Protein FAM3A	FAM3A	Homo sapiens	25.155	3	3	361445	189578
6490	1	P99999	P99999	Cytochrome c	CYCS	Homo sapiens	11.746	5	3	5957483	2327950
6491	1	Q01081	Q01081	Splicing factor U2AF 35 kDa subunit	U2AF1	Homo sapiens	27.87	4	3	989567	547930
6492	1	Q01658	Q01658	Protein Dr1	DR1	Homo sapiens	19.444	3	3	1206972	259346
6493	1	Q02040	Q02040	A-kinase anchor protein 17A	AKAP17A	Homo sapiens	80.738	3	3	69118	15129
6494	1	Q02246	Q02246	Contactin-2	CNTN2	Homo sapiens	113.395	3	3	121887	0
6495	1	Q02410	Q02410	Amyloid-beta A4 precursor protein-binding family A member 1	APBA1	Homo sapiens	92.865	4	3	266244	0
6496	1	Q03013	Q03013	Glutathione S-transferase Mu 4	GSTM4	Homo sapiens	25.561	6	3	643596	64477
6497	1	Q03113	Q03113	Guanine nucleotide-binding protein subunit alpha-12	GNA12	Homo sapiens	44.279	3	3	598314	166995
6498	1	Q03167	Q03167	Transforming growth factor beta receptor type 3	TGFBR3	Homo sapiens	93.499	3	3	444617	45015
6499	1	Q04609	Q04609	Glutamate carboxypeptidase 2	FOLH1	Homo sapiens	84.332	7	3	16766	221278
6500	1	Q04756	Q04756	Hepatocyte growth factor activator	HGFAC	Homo sapiens	70.68	6	3	187883	301100
6501	1	Q05D32	Q05D32	CTD small phosphatase-like protein 2	CTDSPL2	Homo sapiens	52.996	3	3	223918	0
6502	1	Q07352	Q07352	mRNA decay activator protein ZFP36L1	ZFP36L1	Homo sapiens	36.314	4	3	412632	592972
6503	1	Q07864	Q07864	DNA polymerase epsilon catalytic subunit A	POLE	Homo sapiens	261.523	3	3	104919	0
6504	1	Q08722	Q08722	Leukocyte surface antigen CD47	CD47	Homo sapiens	35.214	3	3	3555040	13581552
6505	1	Q09019	Q09019	Dystrophia myotonica WD repeat-containing protein	DMWD	Homo sapiens	70.437	3	3	212480	0
6506	1	Q0P6H9	Q0P6H9	Transmembrane protein 62	TMEM62	Homo sapiens	73.132	3	3	91318	0
6507	1	Q12770	Q12770	Sterol regulatory element-binding protein cleavage-activating protein	SCAP	Homo sapiens	139.728	3	3	133056	20140
6508	1	Q12772	Q12772	Sterol regulatory element-binding protein 2	SREBF2	Homo sapiens	123.687	3	3	97330	0
6509	1	Q12923	Q12923	Tyrosine-protein phosphatase non-receptor type 13	PTPN13	Homo sapiens	276.909	3	3	75570	0
6510	1	Q12982	Q12982	BCL2/adenovirus E1B 19 kDa protein-interacting protein 2	BNIP2	Homo sapiens	36.016	3	3	151393	536262
6511	1	Q13009	Q13009	Rho guanine nucleotide exchange factor TIAM1	TIAM1	Homo sapiens	177.508	3	3	50463	0
6512	1	Q13077	Q13077	TNF receptor-associated factor 1	TRAF1	Homo sapiens	46.164	3	3	48325	72452
6513	1	Q13112	Q13112	Chromatin assembly factor 1 subunit B	CHAF1B	Homo sapiens	61.494	3	3	101617	27624
6514	1	Q13233	Q13233	Mitogen-activated protein kinase kinase kinase 1	MAP3K1	Homo sapiens	164.47	3	3	0	63882
6515	1	Q13243	Q13243	Serine/arginine-rich splicing factor 5	SRSF5	Homo sapiens	31.262	3	3	1729560	301418
6516	1	Q13416	Q13416	Origin recognition complex subunit 2	ORC2	Homo sapiens	65.971	3	3	1935322	11187
6517	1	Q13490	Q13490	Baculoviral IAP repeat-containing protein 2	BIRC2	Homo sapiens	69.901	6	3	367186	171316
6518	1	Q13535	Q13535	Serine/threonine-protein kinase ATR	ATR	Homo sapiens	301.371	3	3	22740	105034
6519	1	Q13542	Q13542	Eukaryotic translation initiation factor 4E-binding protein 2	EIF4EBP2	Homo sapiens	12.939	3	3	156806	41828
6520	1	Q13554	Q13554	Calcium/calmodulin-dependent protein kinase type II subunit beta	CAMK2B	Homo sapiens	72.678	3	3	241348	0
6521	1	Q13608	Q13608	Peroxisomal ATPase PEX6	PEX6	Homo sapiens	104.06	3	3	232123	0
6522	1	Q13625	Q13625	Apoptosis-stimulating of p53 protein 2	TP53BP2	Homo sapiens	125.619	3	3	377979	0
6523	1	Q13671	Q13671	Ras and Rab interactor 1	RIN1	Homo sapiens	84.1	3	3	79683	0

6524	1	Q13790	Q13790	Apolipoprotein F	APOF	Homo sapiens	35.399	3	3	1916515	6008423
6525	1	Q13822	Q13822	Ectonucleotide pyrophosphatase/phosphodiesterase family member 2	ENPP2	Homo sapiens	98.997	3	3	221592	116380
6526	1	Q13887	Q13887	Krueppel-like factor 5	KLF5	Homo sapiens	50.792	4	3	412838	0
6527	2	Q13888;Q6P1K8	Q13888	General transcription factor IIH subunit 2	GTF2H2	Homo sapiens	44.421	3	3	277297	149321
6528	1	Q14019	Q14019	Coactosin-like protein	COTL1	Homo sapiens	15.942	3	3	7889977	10501414
6529	1	Q14031	Q14031	Collagen alpha-6(IV) chain	COL4A6	Homo sapiens	163.808	3	3	248961	0
6530	1	Q14186	Q14186	Transcription factor Dp-1	TFDP1	Homo sapiens	45.069	3	3	180875	0
6531	1	Q14353	Q14353	Guanidinoacetate N-methyltransferase	GAMT	Homo sapiens	26.32	3	3	428338	30600
6532	1	Q14451	Q14451	Growth factor receptor-bound protein 7	GRB7	Homo sapiens	59.68	3	3	58554	27770
6533	1	Q14541	Q14541	Hepatocyte nuclear factor 4-gamma	HNF4G	Homo sapiens	45.876	3	3	198729	0
6534	1	Q14596	Q14596	Next to BRCA1 gene 1 protein	NBR1	Homo sapiens	107.415	3	3	45410	207631
6535	1	Q14623	Q14623	Indian hedgehog protein	IHH	Homo sapiens	45.251	3	3	260548	0
6536	1	Q14693	Q14693	Phosphatidate phosphatase LPIN1	LPIN1	Homo sapiens	98.666	3	3	326313	95974
6537	1	Q14814	Q14814	Myocyte-specific enhancer factor 2D	MEF2D	Homo sapiens	55.938	5	3	145576	79300
6538	1	Q14919	Q14919	Dr1-associated corepressor	DRAP1	Homo sapiens	22.348	3	3	1350356	754205
6539	1	Q14956	Q14956	Transmembrane glycoprotein NMB	GPNMB	Homo sapiens	63.923	3	3	101600	13936
6540	1	Q15051	Q15051	IQ calmodulin-binding motif-containing protein 1	IQCB1	Homo sapiens	68.929	3	3	69751	0
6541	1	Q15102	Q15102	Platelet-activating factor acetylhydrolase IB subunit alpha1	PAFAH1B3	Homo sapiens	25.734	3	3	1905890	1473538
6542	1	Q15173	Q15173	Serine/threonine-protein phosphatase 2A 56 kDa regulatory subunit beta isoform	PPP2R5B	Homo sapiens	57.395	3	3	426672	138566
6543	1	Q15287	Q15287	RNA-binding protein with serine-rich domain 1	RNPS1	Homo sapiens	34.209	3	3	2420587	750456
6544	1	Q15311	Q15311	RalA-binding protein 1	RALBP1	Homo sapiens	76.063	3	3	92174	449380
6545	1	Q15399	Q15399	Toll-like receptor 1	TLR1	Homo sapiens	90.293	5	3	30202	765441
6546	1	Q15528	Q15528	Mediator of RNA polymerase II transcription subunit 22	MED22	Homo sapiens	22.221	3	3	250208	62255
6547	1	Q15722	Q15722	Leukotriene B4 receptor 1	LTB4R	Homo sapiens	37.556	3	3	0	1533916
6548	1	Q15771	Q15771	Ras-related protein Rab-30	RAB30	Homo sapiens	23.057	3	3	472601	0
6549	1	Q15788	Q15788	Nuclear receptor coactivator 1	NCOA1	Homo sapiens	156.759	3	3	201946	82563
6550	1	Q15796	Q15796	Mothers against decapentaplegic homolog 2	SMAD2	Homo sapiens	52.307	6	3	509329	212572
6551	1	Q15800	Q15800	Methylsterol monooxygenase 1	MSMO1	Homo sapiens	35.216	3	3	614108	0
6552	1	Q15819	Q15819	Ubiquitin-conjugating enzyme E2 variant 2	UBE2V2	Homo sapiens	16.363	3	3	9745806	3769099
6553	1	Q15831	Q15831	Serine/threonine-protein kinase STK11	STK11	Homo sapiens	48.636	3	3	209422	23680
6554	1	Q15853	Q15853	Upstream stimulatory factor 2	USF2	Homo sapiens	36.955	4	3	110117	147987
6555	1	Q15911	Q15911	Zinc finger homeobox protein 3	ZFHX3	Homo sapiens	404.425	3	3	119697	0
6556	1	Q16254	Q16254	Transcription factor E2F4	E2F4	Homo sapiens	43.959	3	3	294713	112096
6557	1	Q16563	Q16563	Synaptophysin-like protein 1	SYPL1	Homo sapiens	28.565	3	3	12650916	5992502
6558	1	Q16568	Q16568	Cocaine- and amphetamine-regulated transcript protein	CARTPT	Homo sapiens	12.831	3	3	901654	0
6559	1	Q16584	Q16584	Mitogen-activated protein kinase kinase kinase 11	MAP3K11	Homo sapiens	92.691	3	3	145778	0
6560	1	Q16594	Q16594	Transcription initiation factor TFIID subunit 9	TAF9	Homo sapiens	28.973	7	3	366968	0
6561	1	Q16656	Q16656	Nuclear respiratory factor 1	NRF1	Homo sapiens	53.54	3	3	416417	510313
6562	1	Q16773	Q16773	Kynurenine--oxoglutarate transaminase 1	KYAT1	Homo sapiens	47.873	3	3	194170	39842
6563	1	Q16864	Q16864	V-type proton ATPase subunit F	ATP6V1F	Homo sapiens	13.371	3	3	1321704	1929697
6564	1	Q17R31	Q17R31	Putative deoxyribonuclease TATDN3	TATDN3	Homo sapiens	30.334	3	3	379302	64784
6565	1	Q2M2I3	Q2M2I3	Protein FAM83E	FAM83E	Homo sapiens	51.778	3	3	277875	0
6566	1	Q2M2W7	Q2M2W7	UPF0450 protein C17orf58	C17orf58	Homo sapiens	36.984	3	3	157552	0
6567	1	Q2M385	Q2M385	Macrophage-expressed gene 1 protein	MPEG1	Homo sapiens	78.587	3	3	201456	0
6568	1	Q2NKQ1	Q2NKQ1	Small G protein signaling modulator 1	SGSM1	Homo sapiens	129.716	3	3	189349	0
6569	1	Q2NKX8	Q2NKX8	DNA excision repair protein ERCC-6-like	ERCC6L	Homo sapiens	141.104	3	3	28198	64669
6570	1	Q2TAK8	Q2TAK8	PWWP domain-containing DNA repair factor 3A	PWWP3A	Homo sapiens	78.636	3	3	191762	0

6571	1	Q2TB18	Q2TB18	Protein asteroid homolog 1	ASTE1	Homo sapiens	77.096	3	3	128141	0
6572	1	Q2VPK5	Q2VPK5	Cytoplasmic tRNA 2-thiolation protein 2	CTU2	Homo sapiens	56.107	3	3	1077889	55230
6573	1	Q30154	Q30154	HLA class II histocompatibility antigen, DR beta 5 chain	HLA-DRB5	Homo sapiens	30.056	7	3	344023	737317
6574	1	Q32NB8	Q32NB8	CDP-diacylglycerol--glycerol-3-phosphate 3-phosphatidyltransferase, mitochondrial	PGS1	Homo sapiens	62.733	3	3	303990	22094
6575	1	Q38SD2	Q38SD2	Leucine-rich repeat serine/threonine-protein kinase 1	LRRK1	Homo sapiens	225.398	3	3	11789	137686
6576	1	Q3T906	Q3T906	N-acetylglucosamine-1-phosphotransferase subunits alpha/beta	GNPTAB	Homo sapiens	143.626	3	3	106352	26499
6577	1	Q4V9L6	Q4V9L6	Transmembrane protein 119	TMEM119	Homo sapiens	29.2	3	3	1770405	0
6578	1	Q4VX76	Q4VX76	Synaptotagmin-like protein 3	SYTL3	Homo sapiens	68.56	3	3	7362	234655
6579	1	Q53F39	Q53F39	Metallophosphoesterase 1	MPPE1	Homo sapiens	45.14	3	3	66080	18408
6580	1	Q53FP2	Q53FP2	Novel acetylcholine receptor chaperone	TMEM35A	Homo sapiens	18.437	3	3	3502472	0
6581	1	Q53FT3	Q53FT3	Protein Hikeshi	HIKESHI	Homo sapiens	21.628	3	3	2289798	560351
6582	1	Q53S33	Q53S33	BolA-like protein 3	BOLA3	Homo sapiens	12.114	3	3	363882	26833
6583	1	Q58EX2	Q58EX2	Protein sidekick-2	SDK2	Homo sapiens	239.401	3	3	323273	0
6584	2	Q5BKT4;Q5I7T1	Q5BKT4	Dol-P-Glc:Glc(2)Man(9)GlcNAc(2)-PP-Dol alpha-1,2-glucosyltransferase	ALG10	Homo sapiens	55.609	3	3	947093	1171490
6585	1	Q5EB52	Q5EB52	Mesoderm-specific transcript homolog protein	MEST	Homo sapiens	38.829	3	3	125927	0
6586	1	Q5HYI8	Q5HYI8	Rab-like protein 3	RABL3	Homo sapiens	26.423	3	3	1242551	567346
6587	1	Q5JU69	Q5JU69	Torsin-2A	TOR2A	Homo sapiens	35.711	3	3	123206	516583
6588	1	Q5JU85	Q5JU85	IQ motif and SEC7 domain-containing protein 2	IQSEC2	Homo sapiens	162.788	3	3	156130	0
6589	1	Q5MIZ7	Q5MIZ7	Serine/threonine-protein phosphatase 4 regulatory subunit 3B	PPP4R3B	Homo sapiens	97.46	3	3	280077	48609
6590	1	Q5MNZ9	Q5MNZ9	WD repeat domain phosphoinositide-interacting protein 1	WIPI1	Homo sapiens	48.674	3	3	222630	272866
6591	1	Q5SY16	Q5SY16	Polynucleotide 5'-hydroxyl-kinase NOL9	NOL9	Homo sapiens	79.324	3	3	261088	63806
6592	1	Q5T011	Q5T011	KICSTOR complex protein SZT2	SZT2	Homo sapiens	378.034	3	3	118204	0
6593	1	Q5T1C6	Q5T1C6	Acyl-coenzyme A thioesterase THEM4	THEM4	Homo sapiens	27.13	3	3	247359	0
6594	1	Q5T2W1	Q5T2W1	Na(+)/H(+) exchange regulatory cofactor NHE-RF3	PDZK1	Homo sapiens	57.13	7	3	410103	0
6595	1	Q5T3J3	Q5T3J3	Ligand-dependent nuclear receptor-interacting factor 1	LRIF1	Homo sapiens	84.568	3	3	59025	0
6596	1	Q5TEU4	Q5TEU4	Arginine-hydroxylase NDUFAF5, mitochondrial	NDUFAF5	Homo sapiens	38.918	3	3	360854	77886
6597	1	Q5TGI0	Q5TGI0	Failed axon connections homolog	FAXC	Homo sapiens	46.843	3	3	304467	0
6598	1	Q5TGY1	Q5TGY1	Transmembrane and coiled-coil domain-containing protein 4	TMCO4	Homo sapiens	67.91	3	3	216164	0
6599	1	Q5VT66	Q5VT66	Mitochondrial amidoxime-reducing component 1	MTARC1	Homo sapiens	37.5	3	3	7613	54909
6600	1	Q5VTE6	Q5VTE6	Protein angel homolog 2	ANGEL2	Homo sapiens	62.338	3	3	65387	40009
6601	1	Q5VVQ6	Q5VVQ6	Ubiquitin thioesterase OTU1	YOD1	Homo sapiens	38.321	3	3	166582	109401
6602	1	Q5VYY1	Q5VYY1	Ankyrin repeat domain-containing protein 22	ANKRD22	Homo sapiens	21.847	3	3	600926	1325194
6603	1	Q68BL7	Q68BL7	Olfactomedin-like protein 2A	OLFML2A	Homo sapiens	73.056	3	3	148866	0
6604	1	Q68CQ7	Q68CQ7	Glycosyltransferase 8 domain-containing protein 1	GLT8D1	Homo sapiens	41.936	3	3	446544	61251
6605	1	Q68D10	Q68D10	Protein SPT2 homolog	SPTY2D1	Homo sapiens	75.599	3	3	109511	81443
6606	1	Q68DK7	Q68DK7	Male-specific lethal 1 homolog	MSL1	Homo sapiens	67.127	3	3	600080	67776
6607	1	Q6BDS2	Q6BDS2	Bridge-like lipid transfer protein family member 3A	BLTP3A	Homo sapiens	159.487	3	3	117178	120714
6608	1	Q6GMV3	Q6GMV3	Putative peptidyl-tRNA hydrolase PTRHD1	PTRHD1	Homo sapiens	15.804	3	3	1436821	1489945
6609	1	Q6H8Q1	Q6H8Q1	Actin-binding LIM protein 2	ABLIM2	Homo sapiens	67.815	3	3	91021	0
6610	1	Q6ICB4	Q6ICB4	Sesquipedalian-2	PHETA2	Homo sapiens	28.339	3	3	348236	0
6611	1	Q6IQ49	Q6IQ49	Splicing regulator SDE2	SDE2	Homo sapiens	49.741	3	3	233971	64355
6612	1	Q6ISB3	Q6ISB3	Grainyhead-like protein 2 homolog	GRHL2	Homo sapiens	71.107	3	3	79120	0
6613	1	Q6NTE8	Q6NTE8	MRN complex-interacting protein	MRNIP	Homo sapiens	37.742	3	3	160867	0
6614	1	Q6NW29	Q6NW29	RWD domain-containing protein 4	RWDD4	Homo sapiens	21.249	3	3	471400	76023
6615	1	Q6NW34	Q6NW34	Nucleolus and neural progenitor protein	NEPRO	Homo sapiens	64.553	3	3	144720	0
6616	1	Q6NWY9	Q6NWY9	Pre-mRNA-processing factor 40 homolog B	PRPF40B	Homo sapiens	99.36	3	3	197472	0
6617	1	Q6NXE6	Q6NXE6	Armadillo repeat-containing protein 6	ARMC6	Homo sapiens	54.144	3	3	371301	163124

6618	1	Q6P050	Q6P050	F-box and leucine-rich protein 22	FBXL22	Homo sapiens	27.267	3	3	603796	0
6619	1	Q6P2C8	Q6P2C8	Mediator of RNA polymerase II transcription subunit 27	MED27	Homo sapiens	35.433	3	3	158216	0
6620	1	Q6P3S6	Q6P3S6	F-box only protein 42	FBXO42	Homo sapiens	77.84	3	3	190559	0
6621	1	Q6P589	Q6P589	Tumor necrosis factor alpha-induced protein 8-like protein 2	TNFAIP8L2	Homo sapiens	20.556	3	3	37637	413039
6622	1	Q6P6B1	Q6P6B1	Glutamate-rich protein 5	ERICH5	Homo sapiens	39.935	3	3	163762	0
6623	1	Q6PI47	Q6PI47	BTB/POZ domain-containing protein KCTD18	KCTD18	Homo sapiens	46.74	3	3	83772	15589
6624	1	Q6PI73	Q6PI73	Leukocyte immunoglobulin-like receptor subfamily A member 6	LILRA6	Homo sapiens	52.398	3	3	0	332111
6625	1	Q6PII3	Q6PII3	Coiled-coil domain-containing protein 174	CCDC174	Homo sapiens	53.957	3	3	114644	0
6626	1	Q6PJW8	Q6PJW8	Consortin	CNST	Homo sapiens	79.599	3	3	425258	0
6627	1	Q6PUV4	Q6PUV4	Complexin-2	CPLX2	Homo sapiens	15.393	3	3	363795	88394
6628	1	Q6STE5	Q6STE5	SWI/SNF-related matrix-associated actin-dependent regulator of chromatin subfamily D member 3	SMARCD3	Homo sapiens	55.015	3	3	106671	0
6629	1	Q6UB99	Q6UB99	Ankyrin repeat domain-containing protein 11	ANKRD11	Homo sapiens	297.92	3	3	68337	0
6630	1	Q6UWY2	Q6UWY2	Serine protease 57	PRSS57	Homo sapiens	30.336	3	3	0	743426
6631	1	Q6UX27	Q6UX27	V-set and transmembrane domain-containing protein 1	VSTM1	Homo sapiens	26.11	3	3	0	1789588
6632	1	Q6UXH1	Q6UXH1	Protein disulfide isomerase CRELD2	CRELD2	Homo sapiens	38.194	3	3	1308412	377014
6633	1	Q6UXM1	Q6UXM1	Leucine-rich repeats and immunoglobulin-like domains protein 3	LRIG3	Homo sapiens	123.435	3	3	80011	0
6634	1	Q6ZN28	Q6ZN28	Metastasis-associated in colon cancer protein 1	MACC1	Homo sapiens	96.641	3	3	61141	0
6635	1	Q6ZN55	Q6ZN55	Zinc finger protein 574	ZNF574	Homo sapiens	98.9	3	3	205497	0
6636	1	Q6ZQX7	Q6ZQX7	Protein LIAT1	LIAT1	Homo sapiens	49.658	3	3	251076	0
6637	1	Q6ZS11	Q6ZS11	Ras and Rab interactor-like protein	RINL	Homo sapiens	62.466	3	3	204297	441853
6638	1	Q6ZT21	Q6ZT21	Transmembrane protein with metallophosphoesterase domain	TMPPE	Homo sapiens	49.452	3	3	497248	320229
6639	1	Q6ZTI6	Q6ZTI6	Refilin-A	RFLNA	Homo sapiens	23.611	3	3	565106	0
6640	1	Q6ZV70	Q6ZV70	LanC-like protein 3	LANCL3	Homo sapiens	46.318	3	3	395088	0
6641	1	Q6ZVX7	Q6ZVX7	F-box only protein 50	NCCRP1	Homo sapiens	30.847	3	3	0	1108226
6642	1	Q6ZW49	Q6ZW49	PAX-interacting protein 1	PAXIP1	Homo sapiens	121.341	3	3	120723	108212
6643	1	Q70IA6	Q70IA6	MOB kinase activator 2	MOB2	Homo sapiens	26.927	3	3	871869	287039
6644	1	Q76N32	Q76N32	Centrosomal protein of 68 kDa	CEP68	Homo sapiens	81.102	3	3	106786	0
6645	1	Q7KYR7	Q7KYR7	Butyrophilin subfamily 2 member A1	BTN2A1	Homo sapiens	59.633	5	3	252010	30764
6646	1	Q7LGA3	Q7LGA3	Heparan sulfate 2-O-sulfotransferase 1	HS2ST1	Homo sapiens	41.88	3	3	540795	743957
6647	1	Q7Z2K8	Q7Z2K8	G protein-regulated inducer of neurite outgrowth 1	GPRIN1	Homo sapiens	102.4	3	3	105988	0
6648	1	Q7Z3B3	Q7Z3B3	KAT8 regulatory NSL complex subunit 1	KANSL1	Homo sapiens	121.026	4	3	190637	0
6649	1	Q7Z3G6	Q7Z3G6	Prickle-like protein 2	PRICKLE2	Homo sapiens	95.616	3	3	375518	0
6650	1	Q7Z403	Q7Z403	Transmembrane channel-like protein 6	TMC6	Homo sapiens	90.047	3	3	65962	315814
6651	1	Q7Z412	Q7Z412	Peroxisome assembly protein 26	PEX26	Homo sapiens	33.896	3	3	67448	0
6652	1	Q7Z494	Q7Z494	Nephrocystin-3	NPHP3	Homo sapiens	150.866	3	3	207338	0
6653	1	Q7Z4G4	Q7Z4G4	tRNA (guanine(10)-N2)-methyltransferase homolog	TRMT11	Homo sapiens	53.421	3	3	263636	84587
6654	1	Q7Z4L5	Q7Z4L5	Tetratricopeptide repeat protein 21B	TTC21B	Homo sapiens	150.941	3	3	303532	0
6655	1	Q7Z614	Q7Z614	Sorting nexin-20	SNX20	Homo sapiens	36.176	3	3	0	185722
6656	1	Q7Z624	Q7Z624	Calmodulin-lysine N-methyltransferase	CAMKMT	Homo sapiens	36.126	3	3	94786	0
6657	1	Q7Z6I8	Q7Z6I8	UPF0461 protein C5orf24	C5orf24	Homo sapiens	20.134	3	3	375200	0
6658	1	Q7Z7G8	Q7Z7G8	Intermembrane lipid transfer protein VPS13B	VPS13B	Homo sapiens	448.673	3	3	72933	0
6659	1	Q7Z7M8	Q7Z7M8	UDP-GlcNAc:betaGal beta-1,3-N-acetylglucosaminyltransferase 8	B3GNT8	Homo sapiens	43.395	3	3	499044	268363
6660	1	Q86TJ2	Q86TJ2	Transcriptional adapter 2-beta	TADA2B	Homo sapiens	48.47	3	3	117593	15358
6661	1	Q86TN4	Q86TN4	tRNA 2'-phosphotransferase 1	TRPT1	Homo sapiens	27.739	3	3	387008	12401
6662	1	Q86UB9	Q86UB9	Transmembrane protein 135	TMEM135	Homo sapiens	52.289	3	3	173465	186131
6663	1	Q86UL3	Q86UL3	Glycerol-3-phosphate acyltransferase 4	GPAT4	Homo sapiens	52.07	3	3	230508	8140

6664	1	Q86UL8	Q86UL8	Membrane-associated guanylate kinase, WW and PDZ domain-containing protein 2	MAGI2	Homo sapiens	158.758	3	3	110764	0
6665	1	Q86UW7	Q86UW7	Calcium-dependent secretion activator 2	CADPS2	Homo sapiens	147.738	3	3	203834	0
6666	1	Q86V40	Q86V40	Metalloprotease TIKI1	TRABD2A	Homo sapiens	57.675	4	3	89974	70982
6667	1	Q86V88	Q86V88	Magnesium-dependent phosphatase 1	MDP1	Homo sapiens	20.107	3	3	1791670	194604
6668	1	Q86VQ1	Q86VQ1	Glucocorticoid-induced transcript 1 protein	GLCCI1	Homo sapiens	58.023	3	3	98049	15287
6669	1	Q86VX2	Q86VX2	COMM domain-containing protein 7	COMMD7	Homo sapiens	22.539	3	3	530056	145043
6670	1	Q86W34	Q86W34	Archaemetzincin-2	AMZ2	Homo sapiens	41.264	3	3	324967	73353
6671	1	Q86W50	Q86W50	RNA N6-adenosine-methyltransferase METTL16	METTL16	Homo sapiens	63.62	3	3	538032	150425
6672	1	Q86WH2	Q86WH2	Ras association domain-containing protein 3	RASSF3	Homo sapiens	27.563	3	3	216595	115011
6673	1	Q86WQ0	Q86WQ0	Nuclear receptor 2C2-associated protein	NR2C2AP	Homo sapiens	15.876	3	3	268912	57499
6674	1	Q86WU2	Q86WU2	Probable D-lactate dehydrogenase, mitochondrial	LDHD	Homo sapiens	54.869	3	3	411836	0
6675	1	Q86X52	Q86X52	Chondroitin sulfate synthase 1	CHSY1	Homo sapiens	91.787	3	3	0	223952
6676	1	Q86X83	Q86X83	COMM domain-containing protein 2	COMMD2	Homo sapiens	22.744	3	3	142215	9151
6677	1	Q86XI2	Q86XI2	Condensin-2 complex subunit G2	NCAPG2	Homo sapiens	130.962	3	3	69255	48820
6678	1	Q86XT9	Q86XT9	Insulin-like growth factor-binding protein 3 receptor	TMEM219	Homo sapiens	25.724	3	3	117806	48776
6679	1	Q86Y37	Q86Y37	CDK2-associated and cullin domain-containing protein 1	CACUL1	Homo sapiens	41.066	3	3	131185	51320
6680	1	Q86Y38	Q86Y38	Xylosyltransferase 1	XYLT1	Homo sapiens	107.568	3	3	66218	0
6681	1	Q86YS3	Q86YS3	Rab11 family-interacting protein 4	RAB11FIP4	Homo sapiens	71.931	3	3	161010	0
6682	1	Q86YV5	Q86YV5	Inactive tyrosine-protein kinase PRAG1	PRAG1	Homo sapiens	149.628	3	3	68585	0
6683	1	Q8IUH3	Q8IUH3	RNA-binding protein 45	RBM45	Homo sapiens	53.501	3	3	126018	0
6684	1	Q8IUN9	Q8IUN9	C-type lectin domain family 10 member A	CLEC10A	Homo sapiens	35.445	3	3	148947	0
6685	1	Q8IUR0	Q8IUR0	Trafficking protein particle complex subunit 5	TRAPPC5	Homo sapiens	20.781	3	3	749540	635850
6686	1	Q8IUX1	Q8IUX1	Complex I assembly factor TMEM126B, mitochondrial	TMEM126B	Homo sapiens	25.944	3	3	372010	38327
6687	1	Q8IVT5	Q8IVT5	Kinase suppressor of Ras 1	KSR1	Homo sapiens	102.161	3	3	110444	89659
6688	1	Q8IVV7	Q8IVV7	Glucose-induced degradation protein 4 homolog	GID4	Homo sapiens	33.516	3	3	312007	95017
6689	1	Q8IWE4	Q8IWE4	DCN1-like protein 3	DCUN1D3	Homo sapiens	34.292	3	3	358791	183390
6690	1	Q8IWL3	Q8IWL3	Iron-sulfur cluster co-chaperone protein HscB	HSCB	Homo sapiens	27.423	3	3	297383	113845
6691	1	Q8IWR0	Q8IWR0	Zinc finger CCCH domain-containing protein 7A	ZC3H7A	Homo sapiens	110.54	3	3	438719	59268
6692	1	Q8IWW8	Q8IWW8	Hydroxyacid-oxoacid transhydrogenase, mitochondrial	ADHFE1	Homo sapiens	50.306	3	3	235867	0
6693	1	Q8IXK0	Q8IXK0	Polyhomeotic-like protein 2	PHC2	Homo sapiens	90.714	3	3	290742	74546
6694	1	Q8IYB1	Q8IYB1	Nucleotidyltransferase MB21D2	MB21D2	Homo sapiens	55.801	3	3	119899	0
6695	1	Q8IYB5	Q8IYB5	Stromal membrane-associated protein 1	SMAP1	Homo sapiens	50.386	3	3	1262685	269735
6696	1	Q8IZ41	Q8IZ41	Ras and EF-hand domain-containing protein	RASEF	Homo sapiens	82.882	3	3	128983	99093
6697	1	Q8N0U4	Q8N0U4	Protein FAM185A	FAM185A	Homo sapiens	42.302	3	3	252669	0
6698	1	Q8N0U8	Q8N0U8	Vitamin K epoxide reductase complex subunit 1-like protein 1	VKORC1L1	Homo sapiens	19.835	3	3	405781	739919
6699	1	Q8N137	Q8N137	Centrobin	CNTROB	Homo sapiens	101.255	3	3	36921	431712
6700	1	Q8N1G0	Q8N1G0	Zinc finger protein 687	ZNF687	Homo sapiens	129.529	3	3	286449	144795
6701	1	Q8N1S5	Q8N1S5	Zinc transporter ZIP11	SLC39A11	Homo sapiens	35.394	3	3	1499247	1259757
6702	1	Q8N2K1	Q8N2K1	Ubiquitin-conjugating enzyme E2 J2	UBE2J2	Homo sapiens	28.899	3	3	160911	0
6703	1	Q8N2M8	Q8N2M8	CLK4-associating serine/arginine rich protein	CLASRP	Homo sapiens	77.162	3	3	209465	0
6704	1	Q8N2U9	Q8N2U9	Solute carrier family 66 member 2	SLC66A2	Homo sapiens	30.478	3	3	272199	137735
6705	1	Q8N2W9	Q8N2W9	E3 SUMO-protein ligase PIAS4	PIAS4	Homo sapiens	56.505	3	3	64486	0
6706	1	Q8N370	Q8N370	Large neutral amino acids transporter small subunit 4	SLC43A2	Homo sapiens	62.746	3	3	142542	153772
6707	1	Q8N428	Q8N428	Polypeptide N-acetylgalactosaminyltransferase 16	GALNT16	Homo sapiens	63.074	4	3	84437	0
6708	1	Q8N490	Q8N490	Probable hydrolase PNKD	PNKD	Homo sapiens	42.875	3	3	374111	262086
6709	1	Q8N4L2	Q8N4L2	Type 2 phosphatidylinositol 4,5-bisphosphate 4-phosphatase	PIP4P2	Homo sapiens	28.082	3	3	60158	943557
6710	1	Q8N4S9	Q8N4S9	MARVEL domain-containing protein 2	MARVELD2	Homo sapiens	64.168	3	3	288041	0

6711	1 Q8N5D0	Q8N5D0	WD and tetratricopeptide repeats protein 1	WDTC1	Homo sapiens	75.921	3	3	561755	58186
6712	1 Q8N5H3	Q8N5H3	Leucine repeat adapter protein 25	FAM89B	Homo sapiens	20.147	3	3	262866	0
6713	1 Q8N5M1	Q8N5M1	ATP synthase mitochondrial F1 complex assembly factor 2	ATPAF2	Homo sapiens	32.776	3	3	273998	0
6714	1 Q8N5M9	Q8N5M9	Protein jagunal homolog 1	JAGN1	Homo sapiens	21.122	3	3	1398528	1094008
6715	1 Q8N668	Q8N668	COMM domain-containing protein 1	COMMD1	Homo sapiens	21.181	3	3	924181	693105
6716	1 Q8N699	Q8N699	Myc target protein 1	MYCT1	Homo sapiens	26.593	3	3	210324	88068
6717	1 Q8N6G6	Q8N6G6	ADAMTS-like protein 1	ADAMTSL1	Homo sapiens	193.412	3	3	49960	22052
6718	1 Q8N6M3	Q8N6M3	Acyl-coenzyme A diphosphatase FITM2	FITM2	Homo sapiens	29.855	3	3	656546	132744
6719	1 Q8N6Y1	Q8N6Y1	Protocadherin-20	PCDH20	Homo sapiens	104.92	3	3	47367	0
6720	1 Q8N8E3	Q8N8E3	Centrosomal protein of 112 kDa	CEP112	Homo sapiens	112.749	3	3	203800	0
6721	1 Q8N8V4	Q8N8V4	Ankyrin repeat and SAM domain-containing protein 4B	ANKS4B	Homo sapiens	46.597	3	3	553182	0
6722	1 Q8N9N5	Q8N9N5	Protein BANP	BANP	Homo sapiens	56.495	3	3	106708	81284
6723	1 Q8N9N8	Q8N9N8	Probable RNA-binding protein EIF1AD	EIF1AD	Homo sapiens	19.052	3	3	561361	100702
6724	1 Q8N9Z2	Q8N9Z2	Coiled-coil domain-containing protein 71L	CCDC71L	Homo sapiens	26.262	3	3	113612	425505
6725	1 Q8NAP3	Q8NAP3	Zinc finger and BTB domain-containing protein 38	ZBTB38	Homo sapiens	134.26	3	3	161192	0
6726	1 Q8NAX2	Q8NAX2	Keratinocyte differentiation factor 1	KDF1	Homo sapiens	43.641	3	3	80217	0
6727	1 Q8NBK3	Q8NBK3	Formylglycine-generating enzyme	SUMF1	Homo sapiens	40.556	3	3	516314	429623
6728	1 Q8NBP5	Q8NBP5	Major facilitator superfamily domain-containing protein 9	MFSD9	Homo sapiens	50.617	3	3	33750	200661
6729	1 Q8NBR6	Q8NBR6	Ubiquitin carboxyl-terminal hydrolase MINDY-2	MINDY2	Homo sapiens	67.108	3	3	109266	58993
6730	1 Q8NBT2	Q8NBT2	Kinetochore protein Spc24	SPC24	Homo sapiens	22.442	3	3	158818	42748
6731	1 Q8NCC3	Q8NCC3	Phospholipase A2 group XV	PLA2G15	Homo sapiens	46.657	3	3	327060	213584
6732	1 Q8NCH0	Q8NCH0	Carbohydrate sulfotransferase 14	CHST14	Homo sapiens	42.997	3	3	193067	47208
6733	1 Q8NEL9	Q8NEL9	Phospholipase DDHD1	DDHD1	Homo sapiens	100.437	3	3	438529	0
6734	1 Q8NET5	Q8NET5	NFAT activation molecule 1	NFAM1	Homo sapiens	29.686	3	3	0	484707
6735	1 Q8NFT2	Q8NFT2	Metalloreductase STEAP2	STEAP2	Homo sapiens	56.055	3	3	2243378	17552
6736	1 Q8NFU3	Q8NFU3	Thiosulfate:glutathione sulfurtransferase	TSTD1	Homo sapiens	12.529	3	3	894898	58074
6737	1 Q8NFZ5	Q8NFZ5	TNFAIP3-interacting protein 2	TNIP2	Homo sapiens	48.699	3	3	145955	289598
6738	1 Q8NG11	Q8NG11	Tetraspanin-14	TSPAN14	Homo sapiens	30.69	3	3	127935	357954
6739	1 Q8NHL6	Q8NHL6	Leukocyte immunoglobulin-like receptor subfamily B member 1	LILRB1	Homo sapiens	70.819	4	3	77687	281786
6740	1 Q8NHY0	Q8NHY0	Beta-1,4 N-acetylgalactosaminyltransferase 2	B4GALNT2	Homo sapiens	63.258	3	3	266836	0
6741	1 Q8TBF2	Q8TBF2	Prostamide/prostaglandin F synthase	PRXL2B	Homo sapiens	21.221	3	3	516634	94426
6742	1 Q8TCA0	Q8TCA0	Leucine-rich repeat-containing protein 20	LRRC20	Homo sapiens	20.508	3	3	563062	0
6743	1 Q8TCU4	Q8TCU4	Centrosome-associated protein ALMS1	ALMS1	Homo sapiens	461.071	3	3	64744	0
6744	1 Q8TCX1	Q8TCX1	Cytoplasmic dynein 2 light intermediate chain 1	DYNC2LI1	Homo sapiens	39.622	3	3	210514	7444
6745	1 Q8TD26	Q8TD26	Chromodomain-helicase-DNA-binding protein 6	CHD6	Homo sapiens	305.416	4	3	59836	0
6746	1 Q8TE02	Q8TE02	Elongator complex protein 5	ELP5	Homo sapiens	34.84	3	3	617875	224437
6747	1 Q8TE96	Q8TE96	ATP-dependent RNA helicase DQX1	DQX1	Homo sapiens	79.475	3	3	112543	0
6748	1 Q8TEP8	Q8TEP8	Centrosomal protein of 192 kDa	CEP192	Homo sapiens	279.115	3	3	131281	57819
6749	1 Q8TET4	Q8TET4	Neutral alpha-glucosidase C	GANC	Homo sapiens	104.334	3	3	99392	0
6750	1 Q8TEY7	Q8TEY7	Ubiquitin carboxyl-terminal hydrolase 33	USP33	Homo sapiens	106.726	3	3	186472	0
6751	1 Q8WTV0	Q8WTV0	Scavenger receptor class B member 1	SCARB1	Homo sapiens	60.878	3	3	159693	196870
6752	1 Q8WUD4	Q8WUD4	Coiled-coil domain-containing protein 12	CCDC12	Homo sapiens	19.177	3	3	1007870	119615
6753	1 Q8WUJ0	Q8WUJ0	Serine/threonine/tyrosine-interacting protein	STYX	Homo sapiens	25.491	3	3	121339	0
6754	1 Q8WUN7	Q8WUN7	Ubiquitin domain-containing protein 2	UBTD2	Homo sapiens	26.188	5	3	177621	40815
6755	1 Q8WUU5	Q8WUU5	GATA zinc finger domain-containing protein 1	GATAD1	Homo sapiens	28.691	3	3	82833	9090
6756	1 Q8WV44	Q8WV44	E3 ubiquitin-protein ligase TRIM41	TRIM41	Homo sapiens	71.67	3	3	131324	17066
6757	1 Q8WVB6	Q8WVB6	Chromosome transmission fidelity protein 18 homolog	CHTF18	Homo sapiens	107.383	3	3	129975	0
6758	1 Q8WVF1	Q8WVF1	Protein OSCP1	OSCP1	Homo sapiens	44.586	3	3	180405	0

6759	1	Q8WVJ2	Q8WVJ2	NudC domain-containing protein 2	NUDCD2	Homo sapiens	17.676	3	3	1398727	473155
6760	1	Q8WWX9	Q8WWX9	Selenoprotein M	SELENOM	Homo sapiens	16.202	3	3	905214	46438
6761	1	Q8WXD2	Q8WXD2	Secretogranin-3	SCG3	Homo sapiens	53.005	3	3	274656	0
6762	1	Q8WXD5	Q8WXD5	Gem-associated protein 6	GEMIN6	Homo sapiens	18.824	3	3	405552	46040
6763	1	Q8WXG1	Q8WXG1	S-adenosylmethionine-dependent nucleotide dehydratase RSAD2	RSAD2	Homo sapiens	42.17	3	3	0	128844
6764	1	Q8WXI7	Q8WXI7	Mucin-16	MUC16	Homo sapiens	1519.21	3	3	15999	181462
6765	1	Q92466	Q92466	DNA damage-binding protein 2	DDB2	Homo sapiens	47.864	3	3	350126	144576
6766	1	Q92504	Q92504	Zinc transporter SLC39A7	SLC39A7	Homo sapiens	50.117	3	3	424484	168332
6767	1	Q92545	Q92545	Transmembrane protein 131	TMEM131	Homo sapiens	205.143	3	3	220559	0
6768	1	Q92546	Q92546	RAB6A-GEF complex partner protein 2	RGP1	Homo sapiens	42.453	3	3	80108	25920
6769	1	Q92572	Q92572	AP-3 complex subunit sigma-1	AP3S1	Homo sapiens	21.734	3	3	445120	512533
6770	1	Q92581	Q92581	Sodium/hydrogen exchanger 6	SLC9A6	Homo sapiens	77.918	3	3	371426	122899
6771	1	Q92618	Q92618	Zinc finger protein 516	ZNF516	Homo sapiens	124.291	3	3	13797	37821
6772	1	Q92633	Q92633	Lysophosphatidic acid receptor 1	LPAR1	Homo sapiens	41.109	3	3	807432	0
6773	1	Q92685	Q92685	Dol-P-Man:Man(5)GlcNAc(2)-PP-Dol alpha-1,3-mannosyltransferase	ALG3	Homo sapiens	50.128	3	3	294570	163867
6774	1	Q92692	Q92692	Nectin-2	NECTIN2	Homo sapiens	57.743	3	3	435967	247024
6775	1	Q92794	Q92794	Histone acetyltransferase KAT6A	KAT6A	Homo sapiens	225.031	3	3	61249	100339
6776	1	Q92804	Q92804	TATA-binding protein-associated factor 2N	TAF15	Homo sapiens	61.83	3	3	589822	277089
6777	1	Q92843	Q92843	Bcl-2-like protein 2	BCL2L2	Homo sapiens	20.745	3	3	691345	0
6778	1	Q92854	Q92854	Semaphorin-4D	SEMA4D	Homo sapiens	96.151	3	3	193180	95817
6779	1	Q92930	Q92930	Ras-related protein Rab-8B	RAB8B	Homo sapiens	23.582	3	3	1472846	2801017
6780	1	Q92932	Q92932	Receptor-type tyrosine-protein phosphatase N2	PTPRN2	Homo sapiens	111.273	3	3	238040	0
6781	1	Q92934	Q92934	Bcl2-associated agonist of cell death	BAD	Homo sapiens	18.391	3	3	760974	0
6782	1	Q92959	Q92959	Solute carrier organic anion transporter family member 2A1	SLCO2A1	Homo sapiens	70.044	3	3	294407	0
6783	1	Q93045	Q93045	Stathmin-2	STMN2	Homo sapiens	20.826	3	3	289053	0
6784	1	Q93063	Q93063	Exostosin-2	EXT2	Homo sapiens	82.255	3	3	145234	53325
6785	1	Q969E8	Q969E8	Pre-rRNA-processing protein TSR2 homolog	TSR2	Homo sapiens	20.893	3	3	212132	0
6786	1	Q969H4	Q969H4	Connector enhancer of kinase suppressor of ras 1	CNKSR1	Homo sapiens	79.704	3	3	176310	0
6787	1	Q969M7	Q969M7	NEDD8-conjugating enzyme UBE2F	UBE2F	Homo sapiens	21.076	3	3	271696	104940
6788	1	Q969R8	Q969R8	KICSTOR complex protein ITFG2	ITFG2	Homo sapiens	49.314	3	3	287896	23384
6789	1	Q969V5	Q969V5	Mitochondrial ubiquitin ligase activator of NFKB 1	MUL1	Homo sapiens	39.798	3	3	158914	68273
6790	1	Q969V6	Q969V6	Myocardin-related transcription factor A	MRTFA	Homo sapiens	98.918	3	3	79798	84592
6791	1	Q969X0	Q969X0	RILP-like protein 2	RILPL2	Homo sapiens	23.987	3	3	68571	445349
6792	1	Q96A00	Q96A00	Protein phosphatase 1 regulatory subunit 14A	PPP1R14A	Homo sapiens	16.693	3	3	628624	0
6793	1	Q96A19	Q96A19	Coiled-coil domain-containing protein 102A	CCDC102A	Homo sapiens	62.596	3	3	244864	0
6794	1	Q96A29	Q96A29	GDP-fucose transporter 1	SLC35C1	Homo sapiens	39.806	3	3	386566	230419
6795	1	Q96A57	Q96A57	Transmembrane protein 230	TMEM230	Homo sapiens	13.188	3	3	893137	71638
6796	1	Q96AB3	Q96AB3	Isochorismatase domain-containing protein 2	ISOC2	Homo sapiens	22.335	3	3	668127	0
6797	1	Q96AX2	Q96AX2	Ras-related protein Rab-37	RAB37	Homo sapiens	24.815	3	3	178648	374764
6798	1	Q96AX9	Q96AX9	E3 ubiquitin-protein ligase MIB2	MIB2	Homo sapiens	103.66	3	3	141817	0
6799	1	Q96AZ6	Q96AZ6	Interferon-stimulated gene 20 kDa protein	ISG20	Homo sapiens	20.363	3	3	661000	771588
6800	1	Q96B26	Q96B26	Exosome complex component RRP43	EXOSC8	Homo sapiens	30.042	3	3	675974	368833
6801	1	Q96B77	Q96B77	Transmembrane protein 186	TMEM186	Homo sapiens	24.894	3	3	308189	0
6802	1	Q96BI1	Q96BI1	Solute carrier family 22 member 18	SLC22A18	Homo sapiens	44.847	3	3	1615989	520404
6803	1	Q96BN8	Q96BN8	Ubiquitin thioesterase otulin	OTULIN	Homo sapiens	40.263	3	3	408613	403728
6804	1	Q96BX8	Q96BX8	MOB kinase activator 3A	MOB3A	Homo sapiens	25.464	3	3	630951	2254911
6805	1	Q96C10	Q96C10	ATP-dependent RNA helicase DHX58	DHX58	Homo sapiens	76.613	3	3	304737	131026
6806	1	Q96C57	Q96C57	Protein CUSTOS	CUSTOS	Homo sapiens	28.172	3	3	738530	1715014

6807	1	Q96C92	Q96C92	Endosome-associated-trafficking regulator 1	ENTR1	Homo sapiens	47.961	3	3	237788	0
6808	1	Q96CB8	Q96CB8	Integrator complex subunit 12	INTS12	Homo sapiens	48.809	3	3	231563	38017
6809	1	Q96CB9	Q96CB9	5-methylcytosine rRNA methyltransferase NSUN4	NSUN4	Homo sapiens	43.09	3	3	399454	0
6810	1	Q96CM3	Q96CM3	Pseudouridylate synthase RPUSD4, mitochondrial	RPUSD4	Homo sapiens	42.205	3	3	150106	0
6811	1	Q96CS2	Q96CS2	HAUS augmin-like complex subunit 1	HAUS1	Homo sapiens	31.861	3	3	162032	49215
6812	1	Q96CX6	Q96CX6	Leucine-rich repeat-containing protein 58	LRRC58	Homo sapiens	40.586	3	3	158277	4584
6813	1	Q96DA6	Q96DA6	Mitochondrial import inner membrane translocase subunit TIM14	DNAJC19	Homo sapiens	12.497	3	3	1548933	0
6814	1	Q96DC8	Q96DC8	Enoyl-CoA hydratase domain-containing protein 3, mitochondrial	ECHDC3	Homo sapiens	32.634	3	3	437067	34893
6815	1	Q96DE5	Q96DE5	Anaphase-promoting complex subunit 16	ANAPC16	Homo sapiens	11.666	3	3	106418	12378
6816	1	Q96DX7	Q96DX7	Tripartite motif-containing protein 44	TRIM44	Homo sapiens	38.472	3	3	153735	0
6817	1	Q96E52	Q96E52	Metalloendopeptidase OMA1, mitochondrial	OMA1	Homo sapiens	60.12	3	3	283479	0
6818	1	Q96EB6	Q96EB6	NAD-dependent protein deacetylase sirtuin-1	SIRT1	Homo sapiens	81.684	3	3	360693	0
6819	1	Q96EC8	Q96EC8	Protein YIPF6	YIPF6	Homo sapiens	26.257	3	3	568322	283411
6820	1	Q96EL3	Q96EL3	39S ribosomal protein L53, mitochondrial	MRPL53	Homo sapiens	12.106	3	3	1039690	0
6821	1	Q96ES7	Q96ES7	SAGA-associated factor 29	SGF29	Homo sapiens	33.24	3	3	253802	99800
6822	1	Q96EX1	Q96EX1	Small integral membrane protein 12	SMIM12	Homo sapiens	10.796	3	3	434905	0
6823	1	Q96F46	Q96F46	Interleukin-17 receptor A	IL17RA	Homo sapiens	96.122	3	3	25153	514490
6824	1	Q96F85	Q96F85	CB1 cannabinoid receptor-interacting protein 1	CNRIP1	Homo sapiens	18.648	3	3	434998	0
6825	1	Q96FK6	Q96FK6	WD repeat-containing protein 89	WDR89	Homo sapiens	43.215	3	3	309314	41282
6826	1	Q96G46	Q96G46	tRNA-dihydrouridine(47) synthase [NAD(P)(+)]-like	DUS3L	Homo sapiens	72.595	3	3	193571	46292
6827	1	Q96GE6	Q96GE6	Calmodulin-like protein 4	CALML4	Homo sapiens	21.881	3	3	1293611	0
6828	1	Q96GY0	Q96GY0	Zinc finger C2HC domain-containing protein 1A	ZC2HC1A	Homo sapiens	35.092	3	3	392113	0
6829	1	Q96H78	Q96H78	Solute carrier family 25 member 44	SLC25A44	Homo sapiens	35.393	3	3	107000	92131
6830	1	Q96HR3	Q96HR3	Mediator of RNA polymerase II transcription subunit 30	MED30	Homo sapiens	20.275	3	3	184556	0
6831	1	Q96HR8	Q96HR8	H/ACA ribonucleoprotein complex non-core subunit NAF1	NAF1	Homo sapiens	53.72	3	3	119432	65789
6832	1	Q96K21	Q96K21	Abscission/NoCut checkpoint regulator	ZFYVE19	Homo sapiens	51.546	3	3	382342	129875
6833	1	Q96KB5	Q96KB5	Lymphokine-activated killer T-cell-originated protein kinase	PBK	Homo sapiens	36.086	3	3	242692	87069
6834	1	Q96LB3	Q96LB3	Intraflagellar transport protein 74 homolog	IFT74	Homo sapiens	69.24	3	3	1003849	0
6835	1	Q96LI5	Q96LI5	CCR4-NOT transcription complex subunit 6-like	CNOT6L	Homo sapiens	63.003	5	3	121036	56387
6836	1	Q96LT7	Q96LT7	Guanine nucleotide exchange factor C9orf72	C9orf72	Homo sapiens	54.328	3	3	110195	217175
6837	1	Q96LW7	Q96LW7	Caspase recruitment domain-containing protein 19	CARD19	Homo sapiens	25.59	3	3	797245	240733
6838	1	Q96MF7	Q96MF7	E3 SUMO-protein ligase NSE2	NSMCE2	Homo sapiens	27.933	3	3	190492	0
6839	1	Q96MV1	Q96MV1	TLC domain-containing protein 4	TLCD4	Homo sapiens	30.043	3	3	900972	558880
6840	1	Q96MW1	Q96MW1	Coiled-coil domain-containing protein 43	CCDC43	Homo sapiens	25.25	3	3	328481	164240
6841	1	Q96N96	Q96N96	Spermatogenesis-associated protein 13	SPATA13	Homo sapiens	74.821	4	3	0	537382
6842	1	Q96NB3	Q96NB3	Zinc finger protein 830	ZNF830	Homo sapiens	41.998	3	3	417051	0
6843	1	Q96ND0	Q96ND0	Protein FAM210A	FAM210A	Homo sapiens	30.776	3	3	333553	61206
6844	1	Q96P44	Q96P44	Collagen alpha-1(XXI) chain	COL21A1	Homo sapiens	99.37	3	3	1327993	0
6845	1	Q96P50	Q96P50	Arf-GAP with coiled-coil, ANK repeat and PH domain-containing protein 3	ACAP3	Homo sapiens	92.495	3	3	51859	0
6846	1	Q96PD7	Q96PD7	Diacylglycerol O-acyltransferase 2	DGAT2	Homo sapiens	43.831	3	3	0	135489
6847	1	Q96PE7	Q96PE7	Methylmalonyl-CoA epimerase, mitochondrial	MCEE	Homo sapiens	18.748	3	3	1715146	306794
6848	1	Q96Q83	Q96Q83	Alpha-ketoglutarate-dependent dioxygenase alkB homolog 3	ALKBH3	Homo sapiens	33.375	3	3	163352	0
6849	1	Q96QF0	Q96QF0	Rab-3A-interacting protein	RAB3IP	Homo sapiens	53.02	3	3	158254	162773
6850	1	Q96RD9	Q96RD9	Fc receptor-like protein 5	FCRL5	Homo sapiens	106.437	3	3	0	253412
6851	1	Q96RE7	Q96RE7	Nucleus accumbens-associated protein 1	NACC1	Homo sapiens	57.261	4	3	742622	573268
6852	1	Q96RQ1	Q96RQ1	Endoplasmic reticulum-Golgi intermediate compartment protein 2	ERGIC2	Homo sapiens	42.55	3	3	478315	270352
6853	1	Q96S38	Q96S38	Ribosomal protein S6 kinase delta-1	RPS6KC1	Homo sapiens	118.683	3	3	144511	0
6854	1	Q96S82	Q96S82	Ubiquitin-like protein 7	UBL7	Homo sapiens	40.507	3	3	417454	0

6855	1	Q99467	Q99467	CD180 antigen	CD180	Homo sapiens	74.182	3	3	156277	0
6856	1	Q99489	Q99489	D-aspartate oxidase	DDO	Homo sapiens	37.536	3	3	240718	0
6857	1	Q99542	Q99542	Matrix metalloproteinase-19	MMP19	Homo sapiens	57.358	3	3	47351	200906
6858	1	Q99584	Q99584	Protein S100-A13	S100A13	Homo sapiens	11.47	3	3	16452870	3097610
6859	1	Q99611	Q99611	Selenide, water dikinase 2	SEPHS2	Homo sapiens	47.276	3	3	583905	278611
6860	1	Q99661	Q99661	Kinesin-like protein KIF2C	KIF2C	Homo sapiens	81.317	3	3	71593	314382
6861	1	Q99828	Q99828	Calcium and integrin-binding protein 1	CIB1	Homo sapiens	21.702	3	3	791149	888670
6862	1	Q9BPY8	Q9BPY8	Homeodomain-only protein	HOPX	Homo sapiens	8.256	3	3	959338	0
6863	1	Q9BQI9	Q9BQI9	Nuclear receptor-interacting protein 2	NRIP2	Homo sapiens	31.33	3	3	834704	0
6864	1	Q9BQK8	Q9BQK8	Phosphatidate phosphatase LPIN3	LPIN3	Homo sapiens	93.615	3	3	153069	48236
6865	1	Q9BRK3	Q9BRK3	Matrix remodeling-associated protein 8	MXRA8	Homo sapiens	49.131	3	3	317455	222622
6866	1	Q9BRP8	Q9BRP8	Partner of Y14 and mago	PYM1	Homo sapiens	22.653	3	3	1820467	730655
6867	1	Q9BRR3	Q9BRR3	Post-GPI attachment to proteins factor 4	PGAP4	Homo sapiens	46.589	3	3	568933	0
6868	1	Q9BS92	Q9BS92	Protein NipSnap homolog 3B	NIPSNAP3B	Homo sapiens	28.313	3	3	662206	0
6869	1	Q9BSJ5	Q9BSJ5	Uncharacterized protein C17orf80	C17orf80	Homo sapiens	67.317	3	3	493727	0
6870	1	Q9BT30	Q9BT30	Alpha-ketoglutarate-dependent dioxygenase alkB homolog 7, mitochondrial	ALKBH7	Homo sapiens	24.517	3	3	267805	0
6871	1	Q9BTE1	Q9BTE1	Dynactin subunit 5	DCTN5	Homo sapiens	20.128	3	3	1564304	479423
6872	1	Q9BTT0	Q9BTT0	Acidic leucine-rich nuclear phosphoprotein 32 family member E	ANP32E	Homo sapiens	30.695	3	3	2851659	11841425
6873	1	Q9BTX7	Q9BTX7	Alpha-tocopherol transfer protein-like	TTPAL	Homo sapiens	38.515	3	3	308002	146116
6874	1	Q9BU76	Q9BU76	Multiple myeloma tumor-associated protein 2	MMTAG2	Homo sapiens	29.414	3	3	326362	9929
6875	1	Q9BUD6	Q9BUD6	Spondin-2	SPON2	Homo sapiens	35.789	3	3	0	870761
6876	1	Q9BUK6	Q9BUK6	Protein misato homolog 1	MSTO1	Homo sapiens	61.837	3	3	580642	126140
6877	1	Q9BUR5	Q9BUR5	MICOS complex subunit MIC26	APOO	Homo sapiens	22.284	3	3	2916660	720691
6878	1	Q9BV10	Q9BV10	Dol-P-Man:Man(7)GlcNAc(2)-PP-Dol alpha-1,6-mannosyltransferase	ALG12	Homo sapiens	54.655	3	3	765361	164024
6879	1	Q9BV86	Q9BV86	N-terminal Xaa-Pro-Lys N-methyltransferase 1	NTMT1	Homo sapiens	25.389	3	3	350436	89172
6880	1	Q9BVC3	Q9BVC3	Sister chromatid cohesion protein DCC1	DSCC1	Homo sapiens	44.825	3	3	224765	29411
6881	1	Q9BVC4	Q9BVC4	Target of rapamycin complex subunit LST8	MLST8	Homo sapiens	35.877	3	3	445865	174942
6882	1	Q9BVK2	Q9BVK2	Probable dolichyl pyrophosphate Glc1Man9GlcNAc2 alpha-1,3-glucosyltransferase	ALG8	Homo sapiens	60.087	3	3	657455	591371
6883	1	Q9BVM2	Q9BVM2	Protein DPCD	DPCD	Homo sapiens	23.238	3	3	392871	197352
6884	1	Q9BVN2	Q9BVN2	AP-4 complex accessory subunit RUSC1	RUSC1	Homo sapiens	96.446	3	3	278037	80569
6885	1	Q9BVT8	Q9BVT8	Transmembrane and ubiquitin-like domain-containing protein 1	TMUB1	Homo sapiens	26.262	3	3	209497	95655
6886	1	Q9BW61	Q9BW61	DET1- and DDB1-associated protein 1	DDA1	Homo sapiens	11.837	3	3	424420	82765
6887	1	Q9BWJ5	Q9BWJ5	Splicing factor 3B subunit 5	SF3B5	Homo sapiens	10.134	3	3	393854	132954
6888	1	Q9BWL3	Q9BWL3	Protein C1orf43	C1orf43	Homo sapiens	28.78	3	3	128927	54950
6889	1	Q9BWT7	Q9BWT7	Caspase recruitment domain-containing protein 10	CARD10	Homo sapiens	115.933	3	3	42812	0
6890	1	Q9BX40	Q9BX40	Protein LSM14 homolog B	LSM14B	Homo sapiens	42.07	3	3	345103	165626
6891	1	Q9BX67	Q9BX67	Junctional adhesion molecule C	JAM3	Homo sapiens	35.021	3	3	3441522	0
6892	1	Q9BX68	Q9BX68	Adenosine 5'-monophosphoramidase HINT2	HINT2	Homo sapiens	17.162	3	3	2522863	509204
6893	1	Q9BXB1	Q9BXB1	Leucine-rich repeat-containing G-protein coupled receptor 4	LGR4	Homo sapiens	104.477	3	3	64342	0
6894	1	Q9BXM9	Q9BXM9	FSD1-like protein	FSD1L	Homo sapiens	59.58	3	3	267558	0
6895	1	Q9BXS6	Q9BXS6	Nucleolar and spindle-associated protein 1	NUSAP1	Homo sapiens	49.449	3	3	116880	0
6896	1	Q9BXS9	Q9BXS9	Solute carrier family 26 member 6	SLC26A6	Homo sapiens	82.967	3	3	302890	0
6897	1	Q9BYC8	Q9BYC8	39S ribosomal protein L32, mitochondrial	MRPL32	Homo sapiens	21.405	3	3	634415	0
6898	1	Q9BYC9	Q9BYC9	39S ribosomal protein L20, mitochondrial	MRPL20	Homo sapiens	17.44	3	3	1397450	404842
6899	1	Q9BYG5	Q9BYG5	Partitioning defective 6 homolog beta	PARD6B	Homo sapiens	41.182	3	3	330379	333967
6900	1	Q9BYT3	Q9BYT3	Serine/threonine-protein kinase 33	STK33	Homo sapiens	57.831	3	3	183698	0

6901	1	Q9BYV8	Q9BYV8	Centrosomal protein of 41 kDa	CEP41	Homo sapiens	41.367	3	3	179066	0
6902	1	Q9BYW2	Q9BYW2	Histone-lysine N-methyltransferase SETD2	SETD2	Homo sapiens	287.602	3	3	212423	0
6903	1	Q9BZ72	Q9BZ72	Membrane-associated phosphatidylinositol transfer protein 2	PITPNM2	Homo sapiens	148.934	3	3	679825	28842
6904	1	Q9BZ95	Q9BZ95	Histone-lysine N-methyltransferase NSD3	NSD3	Homo sapiens	161.616	3	3	241814	0
6905	1	Q9BZR8	Q9BZR8	Apoptosis facilitator Bcl-2-like protein 14	BCL2L14	Homo sapiens	36.6	3	3	274637	0
6906	1	Q9C002	Q9C002	Normal mucosa of esophagus-specific gene 1 protein	NMES1	Homo sapiens	9.617	3	3	2004198	44828
6907	1	Q9C0H2	Q9C0H2	Protein tweety homolog 3	TTYH3	Homo sapiens	57.544	3	3	16718	299637
6908	1	Q9GZM5	Q9GZM5	Protein YIPF3	YIPF3	Homo sapiens	38.247	3	3	889938	487318
6909	1	Q9GZR2	Q9GZR2	RNA exonuclease 4	REXO4	Homo sapiens	46.671	3	3	158327	0
6910	1	Q9GZT6	Q9GZT6	Coiled-coil domain-containing protein 90B, mitochondrial	CCDC90B	Homo sapiens	29.504	3	3	248459	85197
6911	1	Q9GZU7	Q9GZU7	Carboxy-terminal domain RNA polymerase II polypeptide A small phosphatase 1	CTDSP1	Homo sapiens	29.203	3	3	948819	787356
6912	1	Q9GZU8	Q9GZU8	PSME3-interacting protein	PSME3IP1	Homo sapiens	28.912	3	3	474082	39185
6913	1	Q9H0K6	Q9H0K6	Pseudouridylate synthase PUS7L	PUS7L	Homo sapiens	80.701	3	3	688821	243803
6914	1	Q9H0W5	Q9H0W5	Coiled-coil domain-containing protein 8	CCDC8	Homo sapiens	59.375	3	3	134735	0
6915	1	Q9H1E3	Q9H1E3	Nuclear ubiquitous casein and cyclin-dependent kinase substrate 1	NUCKS1	Homo sapiens	27.297	3	3	1884833	1701247
6916	1	Q9H1J1	Q9H1J1	Regulator of nonsense transcripts 3A	UPF3A	Homo sapiens	54.696	3	3	97110	0
6917	1	Q9H1K1	Q9H1K1	Iron-sulfur cluster assembly enzyme ISCU	ISCU	Homo sapiens	18	3	3	971225	376985
6918	1	Q9H204	Q9H204	Mediator of RNA polymerase II transcription subunit 28	MED28	Homo sapiens	19.517	3	3	122467	38823
6919	1	Q9H2I8	Q9H2I8	Leucine-rich melanocyte differentiation-associated protein	LRMDA	Homo sapiens	22.566	3	3	79860	551264
6920	1	Q9H2K0	Q9H2K0	Translation initiation factor IF-3, mitochondrial	MTIF3	Homo sapiens	31.724	3	3	448247	234393
6921	1	Q9H3F6	Q9H3F6	BTB/POZ domain-containing adapter for CUL3-mediated RhoA degradation protein 3	KCTD10	Homo sapiens	35.432	4	3	462660	40602
6922	1	Q9H467	Q9H467	CUE domain-containing protein 2	CUEDC2	Homo sapiens	32.008	3	3	353259	0
6923	1	Q9H469	Q9H469	F-box/LRR-repeat protein 15	FBXL15	Homo sapiens	32.996	3	3	202883	0
6924	1	Q9H490	Q9H490	Phosphatidylinositol glycan anchor biosynthesis class U protein	PIGU	Homo sapiens	50.051	3	3	430350	185711
6925	1	Q9H6B4	Q9H6B4	CXADR-like membrane protein	CLMP	Homo sapiens	41.281	3	3	294689	0
6926	1	Q9H6E5	Q9H6E5	Speckle targeted PIP5K1A-regulated poly(A) polymerase	TUT1	Homo sapiens	93.848	3	3	68947	81555
6927	1	Q9H6H4	Q9H6H4	Receptor expression-enhancing protein 4	REEP4	Homo sapiens	29.395	3	3	452330	525602
6928	1	Q9H6K1	Q9H6K1	Protein ILRUN	ILRUN	Homo sapiens	32.874	3	3	142280	99864
6929	1	Q9H6R6	Q9H6R6	Palmitoyltransferase ZDHHC6	ZDHHC6	Homo sapiens	47.663	3	3	86629	146856
6930	1	Q9H706	Q9H706	GRB2-associated and regulator of MAPK protein 1	GAREM1	Homo sapiens	97.188	3	3	276240	0
6931	1	Q9H7B4	Q9H7B4	Histone-lysine N-methyltransferase SMYD3	SMYD3	Homo sapiens	49.095	3	3	332406	0
6932	1	Q9H7H0	Q9H7H0	Methyltransferase-like protein 17, mitochondrial	METTL17	Homo sapiens	50.733	3	3	438022	27340
6933	1	Q9H7X7	Q9H7X7	Intraflagellar transport protein 22 homolog	IFT22	Homo sapiens	20.837	3	3	178345	0
6934	1	Q9H8M5	Q9H8M5	Metal transporter CNNM2	CNNM2	Homo sapiens	96.623	5	3	139903	0
6935	1	Q9H8M7	Q9H8M7	Ubiquitin carboxyl-terminal hydrolase MINDY-3	MINDY3	Homo sapiens	49.724	3	3	661028	48828
6936	1	Q9H8T0	Q9H8T0	AKT-interacting protein	AKTIP	Homo sapiens	33.127	3	3	65565	70050
6937	1	Q9H900	Q9H900	Protein zwilch homolog	ZWILCH	Homo sapiens	67.214	3	3	25795	10241
6938	1	Q9H902	Q9H902	Receptor expression-enhancing protein 1	REEP1	Homo sapiens	22.255	3	3	257906	0
6939	1	Q9H936	Q9H936	Mitochondrial glutamate carrier 1	SLC25A22	Homo sapiens	34.469	4	3	670422	202691
6940	1	Q9H9L3	Q9H9L3	Interferon-stimulated 20 kDa exonuclease-like 2	ISG20L2	Homo sapiens	39.153	3	3	170834	0
6941	1	Q9HAW8	Q9HAW8	UDP-glucuronosyltransferase 1A10	UGT1A10	Homo sapiens	59.809	4	3	1159319	0
6942	1	Q9HB63	Q9HB63	Netrin-4	NTN4	Homo sapiens	70.071	3	3	350472	0
6943	1	Q9HBB8	Q9HBB8	Cadherin-related family member 5	CDHR5	Homo sapiens	88.226	3	3	410599	0
6944	1	Q9HBH0	Q9HBH0	Rho-related GTP-binding protein RhoF	RHOF	Homo sapiens	23.625	3	3	166329	970207
6945	1	Q9HBH1	Q9HBH1	Peptide deformylase, mitochondrial	PDF	Homo sapiens	27.012	3	3	204656	91091
6946	1	Q9HBM6	Q9HBM6	Transcription initiation factor TFIID subunit 9B	TAF9B	Homo sapiens	27.622	3	3	242548	0

6947	1	Q9HBU9	Q9HBU9	Popeye domain-containing protein 2	POPDC2	Homo sapiens	40.448	3	3	283988	0
6948	1	Q9HBW9	Q9HBW9	Adhesion G protein-coupled receptor L4	ADGRL4	Homo sapiens	77.813	3	3	117602	70586
6949	1	Q9HC07	Q9HC07	Transmembrane protein 165	TMEM165	Homo sapiens	34.907	3	3	128813	304414
6950	1	Q9HCI7	Q9HCI7	E3 ubiquitin-protein ligase MSL2	MSL2	Homo sapiens	62.541	3	3	108773	15164
6951	1	Q9HCJ1	Q9HCJ1	Progressive ankylosis protein homolog	ANKH	Homo sapiens	54.243	3	3	132629	0
6952	1	Q9HCK5	Q9HCK5	Protein argonaute-4	AGO4	Homo sapiens	97.1	3	3	167730	311822
6953	1	Q9HCL0	Q9HCL0	Protocadherin-18	PCDH18	Homo sapiens	126.15	3	3	170258	12936
6954	1	Q9HCM2	Q9HCM2	Plexin-A4	PLXNA4	Homo sapiens	212.459	3	3	44214	28852
6955	1	Q9HCP0	Q9HCP0	Casein kinase I isoform gamma-1	CSNK1G1	Homo sapiens	48.512	3	3	202752	157490
6956	1	Q9HCS2	Q9HCS2	Cytochrome P450 4F12	CYP4F12	Homo sapiens	60.309	3	3	106845	21762
6957	1	Q9NP66	Q9NP66	High mobility group protein 20A	HMG20A	Homo sapiens	40.142	3	3	351054	0
6958	1	Q9NP99	Q9NP99	Triggering receptor expressed on myeloid cells 1	TREM1	Homo sapiens	26.388	3	3	0	471900
6959	1	Q9NPD3	Q9NPD3	Exosome complex component RRP41	EXOSC4	Homo sapiens	26.382	3	3	219136	207666
6960	1	Q9NPE2	Q9NPE2	Neugrin	NGRN	Homo sapiens	32.409	3	3	168817	0
6961	1	Q9NPE3	Q9NPE3	H/ACA ribonucleoprotein complex subunit 3	NOP10	Homo sapiens	7.704	3	3	449678	60547
6962	1	Q9NPG4	Q9NPG4	Protocadherin-12	PCDH12	Homo sapiens	128.995	3	3	136914	159646
6963	1	Q9NQ86	Q9NQ86	E3 ubiquitin-protein ligase TRIM36	TRIM36	Homo sapiens	83.015	3	3	71924	0
6964	1	Q9NQH7	Q9NQH7	Xaa-Pro aminopeptidase 3	XPNPEP3	Homo sapiens	57.036	3	3	675924	371601
6965	1	Q9NQT5	Q9NQT5	Exosome complex component RRP40	EXOSC3	Homo sapiens	29.572	3	3	165871	0
6966	1	Q9NR34	Q9NR34	Mannosyl-oligosaccharide 1,2-alpha-mannosidase IC	MAN1C1	Homo sapiens	70.91	3	3	81984	0
6967	1	Q9NRA0	Q9NRA0	Sphingosine kinase 2	SPHK2	Homo sapiens	69.218	3	3	188931	0
6968	1	Q9NRG7	Q9NRG7	Epimerase family protein SDR39U1	SDR39U1	Homo sapiens	31.076	3	3	1227282	322931
6969	1	Q9NRW1	Q9NRW1	Ras-related protein Rab-6B	RAB6B	Homo sapiens	23.461	3	3	1539630	259525
6970	1	Q9NS15	Q9NS15	Latent-transforming growth factor beta-binding protein 3	LTBP3	Homo sapiens	139.36	3	3	41371	32612
6971	1	Q9NS28	Q9NS28	Regulator of G-protein signaling 18	RGS18	Homo sapiens	27.583	3	3	38480	525617
6972	1	Q9NSI2	Q9NSI2	Ribosome biogenesis protein SLX9 homolog	SLX9	Homo sapiens	25.456	3	3	140694	10697
6973	1	Q9NTG7	Q9NTG7	NAD-dependent protein deacetylase sirtuin-3, mitochondrial	SIRT3	Homo sapiens	43.573	3	3	268261	15008
6974	1	Q9NU19	Q9NU19	TBC1 domain family member 22B	TBC1D22B	Homo sapiens	59.082	3	3	23443	91594
6975	1	Q9NU23	Q9NU23	LYR motif-containing protein 2	LYRM2	Homo sapiens	10.447	3	3	713460	40485
6976	1	Q9NUE0	Q9NUE0	Palmitoyltransferase ZDHHC18	ZDHHC18	Homo sapiens	42.03	3	3	0	113303
6977	1	Q9NUN5	Q9NUN5	Lysosomal cobalamin transport escort protein LMBD1	LMBRD1	Homo sapiens	61.388	3	3	273185	149405
6978	1	Q9NUP1	Q9NUP1	Biogenesis of lysosome-related organelles complex 1 subunit 4	BLOC1S4	Homo sapiens	23.35	3	3	635469	389500
6979	1	Q9NV31	Q9NV31	U3 small nucleolar ribonucleoprotein protein IMP3	IMP3	Homo sapiens	21.849	3	3	535719	190184
6980	1	Q9NVA1	Q9NVA1	Ubiquinol-cytochrome-c reductase complex assembly factor 1	UQCC1	Homo sapiens	34.601	3	3	970509	174392
6981	1	Q9NVE5	Q9NVE5	Ubiquitin carboxyl-terminal hydrolase 40	USP40	Homo sapiens	140.132	3	3	142603	0
6982	1	Q9NVF7	Q9NVF7	F-box only protein 28	FBXO28	Homo sapiens	41.147	3	3	70576	28674
6983	1	Q9NVN3	Q9NVN3	Synembryn-B	RIC8B	Homo sapiens	58.827	3	3	219454	0
6984	1	Q9NVR0	Q9NVR0	Kelch-like protein 11	KLHL11	Homo sapiens	80.148	3	3	88119	0
6985	1	Q9NVS2	Q9NVS2	39S ribosomal protein S18a, mitochondrial	MRPS18A	Homo sapiens	22.184	3	3	94296	0
6986	1	Q9NVZ3	Q9NVZ3	Adaptin ear-binding coat-associated protein 2	NECAP2	Homo sapiens	28.337	3	3	2074695	1321786
6987	1	Q9NW97	Q9NW97	Transmembrane protein 51	TMEM51	Homo sapiens	27.758	3	3	273107	0
6988	1	Q9NWR8	Q9NWR8	Calcium uniporter regulatory subunit MCUb, mitochondrial	MCUB	Homo sapiens	39.085	3	3	180277	22629
6989	1	Q9NWW6	Q9NWW6	Nicotinamide riboside kinase 1	NMRK1	Homo sapiens	23.191	3	3	43934	173792
6990	1	Q9NWX5	Q9NWX5	Ankyrin repeat and SOCS box protein 6	ASB6	Homo sapiens	47.138	3	3	1209756	0
6991	1	Q9NWX6	Q9NWX6	Probable tRNA(His) guanylyltransferase	THG1L	Homo sapiens	34.831	3	3	1084670	217939
6992	1	Q9NX00	Q9NX00	Transmembrane protein 160	TMEM160	Homo sapiens	19.656	3	3	358064	83225
6993	1	Q9NX24	Q9NX24	H/ACA ribonucleoprotein complex subunit 2	NHP2	Homo sapiens	17.2	3	3	1838493	820694
6994	1	Q9NX78	Q9NX78	Transmembrane protein 260	TMEM260	Homo sapiens	79.537	3	3	156697	80601

6995	1	Q9NY12	Q9NY12	H/ACA ribonucleoprotein complex subunit 1	GAR1	Homo sapiens	22.347	3	3	2556029	978602
6996	1	Q9NYJ1	Q9NYJ1	Cytochrome c oxidase assembly factor 4 homolog, mitochondrial	COA4	Homo sapiens	10.133	3	3	591218	121965
6997	1	Q9NYL4	Q9NYL4	Peptidyl-prolyl cis-trans isomerase FKBP11	FKBP11	Homo sapiens	22.18	3	3	1972435	675897
6998	1	Q9NYR9	Q9NYR9	NF-kappa-B inhibitor-interacting Ras-like protein 2	NKIRAS2	Homo sapiens	21.509	3	3	301941	202883
6999	1	Q9NYV6	Q9NYV6	RNA polymerase I-specific transcription initiation factor RRN3	RRN3	Homo sapiens	74.107	3	3	139956	0
7000	1	Q9NZC3	Q9NZC3	Glycerophosphodiester phosphodiesterase 1	GDE1	Homo sapiens	37.719	3	3	547151	417288
7001	1	Q9NZC7	Q9NZC7	WW domain-containing oxidoreductase	WWOX	Homo sapiens	46.677	3	3	203142	0
7002	1	Q9NZI6	Q9NZI6	Transcription factor CP2-like protein 1	TFCP2L1	Homo sapiens	54.625	3	3	245738	0
7003	1	Q9NZI7	Q9NZI7	Upstream-binding protein 1	UBP1	Homo sapiens	60.491	3	3	454146	87396
7004	1	Q9NZJ6	Q9NZJ6	Ubiquinone biosynthesis O-methyltransferase, mitochondrial	COQ3	Homo sapiens	41.054	3	3	768355	0
7005	1	Q9NZM5	Q9NZM5	Ribosome biogenesis protein NOP53	NOP53	Homo sapiens	54.39	3	3	267944	0
7006	1	Q9NZU0	Q9NZU0	Leucine-rich repeat transmembrane protein FLRT3	FLRT3	Homo sapiens	73.002	3	3	232659	0
7007	1	Q9NZZ3	Q9NZZ3	Charged multivesicular body protein 5	CHMP5	Homo sapiens	24.57	3	3	639918	523057
7008	1	Q9P013	Q9P013	Spliceosome-associated protein CWC15 homolog	CWC15	Homo sapiens	26.621	3	3	412317	135590
7009	1	Q9P0M2	Q9P0M2	A-kinase anchor protein 7 isoform gamma	AKAP7	Homo sapiens	39.516	3	3	242302	55381
7010	1	Q9P0R6	Q9P0R6	GSK3B-interacting protein	GSKIP	Homo sapiens	15.647	3	3	450152	143680
7011	1	Q9P1Z0	Q9P1Z0	Zinc finger and BTB domain-containing protein 4	ZBTB4	Homo sapiens	105.114	3	3	211731	0
7012	1	Q9P2E5	Q9P2E5	Chondroitin sulfate glucuronyltransferase	CHPF2	Homo sapiens	85.949	3	3	17511	106181
7013	1	Q9P2N6	Q9P2N6	KAT8 regulatory NSL complex subunit 3	KANSL3	Homo sapiens	95.992	3	3	94543	27086
7014	1	Q9P2Y5	Q9P2Y5	UV radiation resistance-associated gene protein	UVRAG	Homo sapiens	78.153	3	3	306093	52118
7015	2	Q9UBK7;Q9UNT1	Q9UBK7	Rab-like protein 2A	RABL2A	Homo sapiens	26.115	3	3	480247	21154
7016	1	Q9UBK9	Q9UBK9	Protein UXT	UXT	Homo sapiens	18.247	3	3	238285	24114
7017	1	Q9UBM7	Q9UBM7	7-dehydrocholesterol reductase	DHCR7	Homo sapiens	54.49	3	3	634672	982501
7018	1	Q9UBP4	Q9UBP4	Dickkopf-related protein 3	DKK3	Homo sapiens	38.39	3	3	115257	261069
7019	1	Q9UBQ6	Q9UBQ6	Exostosin-like 2	EXTL2	Homo sapiens	37.464	3	3	230019	28211
7020	1	Q9UBS0	Q9UBS0	Ribosomal protein S6 kinase beta-2	RPS6KB2	Homo sapiens	53.455	3	3	132822	340107
7021	1	Q9UBS9	Q9UBS9	SUN domain-containing ossification factor	SUCO	Homo sapiens	139.432	3	3	64907	0
7022	1	Q9UD71	Q9UD71	Protein phosphatase 1 regulatory subunit 1B	PPP1R1B	Homo sapiens	22.963	3	3	1257553	0
7023	1	Q9UEW3	Q9UEW3	Macrophage receptor MARCO	MARCO	Homo sapiens	52.656	3	3	0	134094
7024	1	Q9UGN4	Q9UGN4	CMRF35-like molecule 8	CD300A	Homo sapiens	33.202	3	3	81070	779263
7025	1	Q9UGN5	Q9UGN5	Poly [ADP-ribose] polymerase 2	PARP2	Homo sapiens	66.206	3	3	106570	0
7026	1	Q9UHA4	Q9UHA4	Ragulator complex protein LAMTOR3	LAMTOR3	Homo sapiens	13.623	3	3	2809508	1690107
7027	1	Q9UHD9	Q9UHD9	Ubiquilin-2	UBQLN2	Homo sapiens	65.697	5	3	718568	493420
7028	1	Q9UHF1	Q9UHF1	Epidermal growth factor-like protein 7	EGFL7	Homo sapiens	29.618	3	3	317758	0
7029	1	Q9UHI5	Q9UHI5	Large neutral amino acids transporter small subunit 2	SLC7A8	Homo sapiens	58.381	3	3	175476	37963
7030	1	Q9UHV7	Q9UHV7	Mediator of RNA polymerase II transcription subunit 13	MED13	Homo sapiens	239.299	3	3	177068	0
7031	1	Q9UHX3	Q9UHX3	Adhesion G protein-coupled receptor E2	ADGRE2	Homo sapiens	90.472	3	3	5786	463548
7032	1	Q9UIK4	Q9UIK4	Death-associated protein kinase 2	DAPK2	Homo sapiens	42.899	3	3	66821	414427
7033	1	Q9UIM3	Q9UIM3	FK506-binding protein-like	FKBPL	Homo sapiens	38.174	3	3	139600	0
7034	1	Q9UJC5	Q9UJC5	SH3 domain-binding glutamic acid-rich-like protein 2	SH3BGRL2	Homo sapiens	12.326	3	3	801087	32749
7035	1	Q9UJG1	Q9UJG1	Motile sperm domain-containing protein 1	MOSPD1	Homo sapiens	24.086	3	3	115177	37232
7036	1	Q9UJV3	Q9UJV3	Probable E3 ubiquitin-protein ligase MID2	MID2	Homo sapiens	83.209	3	3	66946	0
7037	1	Q9UJY1	Q9UJY1	Heat shock protein beta-8	HSPB8	Homo sapiens	21.604	3	3	8692965	149349
7038	1	Q9UK73	Q9UK73	Protein fem-1 homolog B	FEM1B	Homo sapiens	70.267	3	3	92920	0
7039	1	Q9UKG9	Q9UKG9	Peroxisomal carnitine O-octanoyltransferase	CROT	Homo sapiens	70.179	3	3	858923	0
7040	1	Q9UKI2	Q9UKI2	Cdc42 effector protein 3	CDC42EP3	Homo sapiens	27.676	3	3	282476	0
7041	1	Q9UKI8	Q9UKI8	Serine/threonine-protein kinase tousled-like 1	TLK1	Homo sapiens	86.698	5	3	72161	0
7042	1	Q9UKJ1	Q9UKJ1	Paired immunoglobulin-like type 2 receptor alpha	PILRA	Homo sapiens	34.005	3	3	0	333205

7043	1	Q9UKT5	Q9UKT5	F-box only protein 4	FBXO4	Homo sapiens	44.136	3	3	522245	501081
7044	1	Q9UKT8	Q9UKT8	F-box/WD repeat-containing protein 2	FBXW2	Homo sapiens	51.514	3	3	323739	71461
7045	1	Q9UKZ9	Q9UKZ9	Procollagen C-endopeptidase enhancer 2	PCOLCE2	Homo sapiens	45.718	3	3	80981	193383
7046	1	Q9ULC4	Q9ULC4	Malignant T-cell-amplified sequence 1	MCTS1	Homo sapiens	20.555	7	3	3206393	3157914
7047	1	Q9ULE3	Q9ULE3	DENN domain-containing protein 2A	DENND2A	Homo sapiens	113.853	3	3	124215	0
7048	1	Q9ULG1	Q9ULG1	Chromatin-remodeling ATPase INO80	INO80	Homo sapiens	176.755	3	3	144217	167247
7049	1	Q9ULP0	Q9ULP0	Protein NDRG4	NDRG4	Homo sapiens	38.457	3	3	535219	0
7050	1	Q9ULR0	Q9ULR0	Pre-mRNA-splicing factor ISY1 homolog	ISY1	Homo sapiens	32.993	3	3	1103754	225239
7051	1	Q9ULW0	Q9ULW0	Targeting protein for Xklp2	TPX2	Homo sapiens	85.655	3	3	95494	0
7052	1	Q9UMX3	Q9UMX3	Bcl-2-related ovarian killer protein	BOK	Homo sapiens	23.281	3	3	715954	0
7053	1	Q9UMY1	Q9UMY1	Nucleolar protein 7	NOL7	Homo sapiens	29.424	3	3	1060330	40551
7054	1	Q9UN70	Q9UN70	Protocadherin gamma-C3	PCDHGC3	Homo sapiens	101.078	3	3	301463	0
7055	1	Q9UNK0	Q9UNK0	Syntaxin-8	STX8	Homo sapiens	26.905	3	3	970542	635490
7056	1	Q9UPN4	Q9UPN4	Centrosomal protein of 131 kDa	CEP131	Homo sapiens	122.15	3	3	15910656	0
7057	1	Q9UQN3	Q9UQN3	Charged multivesicular body protein 2b	CHMP2B	Homo sapiens	23.905	3	3	2767379	608548
7058	1	Q9UQP3	Q9UQP3	Tenascin-N	TNN	Homo sapiens	144.037	3	3	98439	0
7059	1	Q9Y237	Q9Y237	Peptidyl-prolyl cis-trans isomerase NIMA-interacting 4	PIN4	Homo sapiens	13.81	3	3	915320	431922
7060	1	Q9Y286	Q9Y286	Sialic acid-binding Ig-like lectin 7	SIGLEC7	Homo sapiens	51.144	3	3	118678	539422
7061	1	Q9Y287	Q9Y287	Integral membrane protein 2B	ITM2B	Homo sapiens	30.338	3	3	375813	1108082
7062	1	Q9Y2B9	Q9Y2B9	cAMP-dependent protein kinase inhibitor gamma	PKIG	Homo sapiens	7.909	3	3	623340	0
7063	1	Q9Y2M5	Q9Y2M5	Kelch-like protein 20	KLHL20	Homo sapiens	67.957	3	3	114058	0
7064	1	Q9Y2Q5	Q9Y2Q5	Ragulator complex protein LAMTOR2	LAMTOR2	Homo sapiens	13.506	3	3	288538	110594
7065	1	Q9Y2Q9	Q9Y2Q9	28S ribosomal protein S28, mitochondrial	MRPS28	Homo sapiens	20.842	3	3	1434946	306447
7066	1	Q9Y2R5	Q9Y2R5	28S ribosomal protein S17, mitochondrial	MRPS17	Homo sapiens	14.501	3	3	1542764	545973
7067	1	Q9Y2U5	Q9Y2U5	Mitogen-activated protein kinase kinase kinase 2	MAP3K2	Homo sapiens	69.741	3	3	174503	400914
7068	1	Q9Y2Y0	Q9Y2Y0	ADP-ribosylation factor-like protein 2-binding protein	ARL2BP	Homo sapiens	18.82	3	3	412163	63413
7069	1	Q9Y336	Q9Y336	Sialic acid-binding Ig-like lectin 9	SIGLEC9	Homo sapiens	50.083	3	3	0	737164
7070	1	Q9Y375	Q9Y375	Complex I intermediate-associated protein 30, mitochondrial	NDUFAF1	Homo sapiens	37.762	3	3	298683	0
7071	1	Q9Y388	Q9Y388	RNA-binding motif protein, X-linked 2	RBMX2	Homo sapiens	37.335	3	3	194176	40826
7072	1	Q9Y3B2	Q9Y3B2	Exosome complex component CSL4	EXOSC1	Homo sapiens	21.452	3	3	447032	403140
7073	1	Q9Y3C1	Q9Y3C1	Nucleolar protein 16	NOP16	Homo sapiens	21.189	3	3	222936	21095
7074	1	Q9Y3C4	Q9Y3C4	EKC/KEOPS complex subunit TPRKB	TPRKB	Homo sapiens	19.661	3	3	1016713	322020
7075	1	Q9Y3E2	Q9Y3E2	BolA-like protein 1	BOLA1	Homo sapiens	14.287	3	3	121643	20047
7076	1	Q9Y3E7	Q9Y3E7	Charged multivesicular body protein 3	CHMP3	Homo sapiens	25.071	3	3	5886832	2413477
7077	1	Q9Y3L5	Q9Y3L5	Ras-related protein Rap-2c	RAP2C	Homo sapiens	20.745	3	3	483103	645571
7078	1	Q9Y3Q0	Q9Y3Q0	N-acetylated-alpha-linked acidic dipeptidase 2	NAALAD2	Homo sapiens	83.593	3	3	94625	0
7079	1	Q9Y3Q3	Q9Y3Q3	Transmembrane emp24 domain-containing protein 3	TMED3	Homo sapiens	24.779	3	3	555873	774578
7080	1	Q9Y484	Q9Y484	WD repeat domain phosphoinositide-interacting protein 4	WDR45	Homo sapiens	39.869	3	3	317245	181183
7081	1	Q9Y4E1	Q9Y4E1	WASH complex subunit 2C	WASHC2C	Homo sapiens	144.912	18	3	204368	26126
7082	1	Q9Y4E5	Q9Y4E5	E3 SUMO-protein ligase ZNF451	ZNF451	Homo sapiens	121.488	3	3	166913	0
7083	1	Q9Y4H2	Q9Y4H2	Insulin receptor substrate 2	IRS2	Homo sapiens	137.337	3	3	247896	69053
7084	1	Q9Y4Z0	Q9Y4Z0	U6 snRNA-associated Sm-like protein LSm4	LSM4	Homo sapiens	15.347	3	3	785818	444622
7085	1	Q9Y547	Q9Y547	Intraflagellar transport protein 25 homolog	HSPB11	Homo sapiens	16.295	3	3	743227	595405
7086	1	Q9Y5J5	Q9Y5J5	Pleckstrin homology-like domain family A member 3	PHLDA3	Homo sapiens	13.888	3	3	3122007	54605
7087	1	Q9Y5N5	Q9Y5N5	Methyltransferase N6AMT1	N6AMT1	Homo sapiens	22.96	3	3	167253	0
7088	1	Q9Y5S9	Q9Y5S9	RNA-binding protein 8A	RBM8A	Homo sapiens	19.89	3	3	4552309	2466837
7089	1	Q9Y5V3	Q9Y5V3	Melanoma-associated antigen D1	MAGED1	Homo sapiens	86.159	3	3	123520	31042
7090	1	Q9Y657	Q9Y657	Spindlin-1	SPIN1	Homo sapiens	29.599	3	3	1219741	22351

7091	1	Q9Y664	Q9Y664	KICSTOR complex protein kaptin	KPTN	Homo sapiens	48.081	3	3	77401	230864
7092	1	Q9Y672	Q9Y672	Dolichyl pyrophosphate Man9GlcNAc2 alpha-1,3-glucosyltransferase	ALG6	Homo sapiens	58.121	3	3	838318	541046
7093	1	Q9Y692	Q9Y692	Glucocorticoid modulatory element-binding protein 1	GMEB1	Homo sapiens	62.591	3	3	179589	1647934
7094	1	Q9Y6A1	Q9Y6A1	Protein O-mannosyl-transferase 1	POMT1	Homo sapiens	84.88	3	3	132319	0
7095	1	Q9Y6J9	Q9Y6J9	TAF6-like RNA polymerase II p300/CBP-associated factor-associated factor 65 kDa subunit 6L	TAF6L	Homo sapiens	67.816	3	3	195774	33132
7096	1	Q9Y6V7	Q9Y6V7	Probable ATP-dependent RNA helicase DDX49	DDX49	Homo sapiens	54.227	3	3	365203	84742
7097	1	Q9Y6Z7	Q9Y6Z7	Collectin-10	COLEC10	Homo sapiens	30.705	3	3	13817	424283
7098	1	A0A075B6H9	A0A075B6H9	Immunoglobulin lambda variable 4-69	IGLV4-69	Homo sapiens	12.774	2	2	892220	2324888
7099	1	A0A075B6I0	A0A075B6I0	Immunoglobulin lambda variable 8-61	IGLV8-61	Homo sapiens	12.813	2	2	8014656	18113021
7100	1	A0A075B6I4	A0A075B6I4	Immunoglobulin lambda variable 10-54	IGLV10-54	Homo sapiens	12.392	2	2	249270	43517
7101	1	A0A075B6K4	A0A075B6K4	Immunoglobulin lambda variable 3-10	IGLV3-10	Homo sapiens	12.438	3	2	2491281	5655072
7102	1	A0A075B6S5	A0A075B6S5	Immunoglobulin kappa variable 1-27	IGKV1-27	Homo sapiens	12.71	2	2	189448	344815
7103	2	A0A087WW87;P0	A0A087WW87	Immunoglobulin kappa variable 2-40	IGKV2-40	Homo sapiens	13.31	3	2	151662	215109
7104	1	A0A0B4J1U7	A0A0B4J1U7	Immunoglobulin heavy variable 6-1	IGHV6-1	Homo sapiens	13.48	2	2	395255	725778
7105	1	A0A0B4J1V6	A0A0B4J1V6	Immunoglobulin heavy variable 3-73	IGHV3-73	Homo sapiens	12.857	2	2	924634	1682435
7106	1	A0A0B4J1Y9	A0A0B4J1Y9	Immunoglobulin heavy variable 3-72	IGHV3-72	Homo sapiens	13.202	3	2	1181282	2077300
7107	1	A0A0C4DH33	A0A0C4DH33	Immunoglobulin heavy variable 1-24	IGHV1-24	Homo sapiens	12.824	2	2	61983	50044
7108	1	A0A0C4DH36	A0A0C4DH36	Probable non-functional immunoglobulin heavy variable 3-38	IGHV3-38	Homo sapiens	12.761	2	2	1087854	1430809
7109	1	A0A0C4DH38	A0A0C4DH38	Immunoglobulin heavy variable 5-51	IGHV5-51	Homo sapiens	12.673	4	2	3378776	3874729
7110	5	A0A0C4DH72;A0A	A0A0C4DH72	Immunoglobulin kappa variable 1-6	IGKV1-6	Homo sapiens	12.698	2	2	1132023	1151970
7111	1	A0A0J9YW62	A0A0J9YW62	Immunoglobulin V-set domain-containing protein (Fragment)	-	Homo sapiens	13.249	2	2	84341	119690
7112	1	A0PJW6	A0PJW6	Transmembrane protein 223	TMEM223	Homo sapiens	22.049	2	2	285660	80225
7113	1	A1A5B4	A1A5B4	Anoctamin-9	ANO9	Homo sapiens	90.334	2	2	311509	0
7114	1	A2RU49	A2RU49	Hydroxylysine kinase	HYKK	Homo sapiens	41.934	2	2	41162	0
7115	1	A5D8V6	A5D8V6	Vacuolar protein sorting-associated protein 37C	VPS37C	Homo sapiens	38.66	2	2	1004339	273496
7116	2	A6NCE7;Q9GZQ8	A6NCE7	Microtubule-associated proteins 1A/1B light chain 3 beta 2	MAP1LC3B2	Homo sapiens	14.627	2	2	917378	240830
7117	1	A6NCS6	A6NCS6	Uncharacterized protein C2orf72	C2orf72	Homo sapiens	30.479	2	2	170061	0
7118	1	A6NI73	A6NI73	Leukocyte immunoglobulin-like receptor subfamily A member 5	LILRA5	Homo sapiens	32.754	2	2	0	520626
7119	1	A6NK44	A6NK44	Glyoxalase domain-containing protein 5	GLOD5	Homo sapiens	18.322	2	2	144462	0
7120	1	A6NKN8	A6NKN8	Purkinje cell protein 4-like protein 1	PCP4L1	Homo sapiens	7.474	2	2	1428037	0
7121	1	A8MVW0	A8MVW0	Protein FAM171A2	FAM171A2	Homo sapiens	87.435	2	2	19620	0
7122	2	A8MWD9;P62308	A8MWD9	Putative small nuclear ribonucleoprotein G-like protein 15	SNRPGP15	Homo sapiens	8.541	2	2	1105947	361004
7123	2	B9A064;P0CG04	B9A064	Immunoglobulin lambda-like polypeptide 5	IGLL5	Homo sapiens	23.061	4	2	8708363	20095442
7124	2	C4AMC7;Q6VEQ5	C4AMC7	Putative WAS protein family homolog 3	WASH3P	Homo sapiens	49.995	7	2	73058	26298
7125	1	C9J7I0	C9J7I0	UBAP1-MVB12-associated (UMA)-domain containing protein 1	UMAD1	Homo sapiens	15.198	2	2	764850	77612
7126	1	E9PRG8	E9PRG8	Uncharacterized protein C11orf98	C11orf98	Homo sapiens	14.232	2	2	435305	204694
7127	1	O00189	O00189	AP-4 complex subunit mu-1	AP4M1	Homo sapiens	49.978	2	2	168463	0
7128	1	O00287	O00287	Regulatory factor X-associated protein	RFXAP	Homo sapiens	28.233	2	2	142596	0
7129	1	O00421	O00421	C-C chemokine receptor-like 2	CCRL2	Homo sapiens	39.513	2	2	37626	107295
7130	1	O00445	O00445	Synaptotagmin-5	SYT5	Homo sapiens	42.901	2	2	62423	0
7131	1	O00453	O00453	Leukocyte-specific transcript 1 protein	LST1	Homo sapiens	10.793	2	2	91007	1259075
7132	1	O00488	O00488	Zinc finger protein 593	ZNF593	Homo sapiens	15.199	2	2	195959	0
7133	1	O00506	O00506	Serine/threonine-protein kinase 25	STK25	Homo sapiens	48.111	2	2	540938	184320
7134	1	O00512	O00512	B-cell CLL/lymphoma 9 protein	BCL9	Homo sapiens	149.291	2	2	35174	33912
7135	1	O00585	O00585	C-C motif chemokine 21	CCL21	Homo sapiens	14.645	2	2	49444	0
7136	1	O00628	O00628	Peroxisomal targeting signal 2 receptor	PEX7	Homo sapiens	35.893	2	2	100446	0
7137	1	O14492	O14492	SH2B adapter protein 2	SH2B2	Homo sapiens	67.739	2	2	0	77040

7138	1	O14508	O14508	Suppressor of cytokine signaling 2	SOCS2	Homo sapiens	22.175	2	2	63706	0
7139	1	O14526	O14526	F-BAR domain only protein 1	FCHO1	Homo sapiens	96.863	2	2	25280	214872
7140	1	O14653	O14653	Golgi SNAP receptor complex member 2	GOSR2	Homo sapiens	24.774	2	2	152397	0
7141	1	O14757	O14757	Serine/threonine-protein kinase Chk1	CHEK1	Homo sapiens	54.433	2	2	123703	168298
7142	1	O14777	O14777	Kinetochore protein NDC80 homolog	NDC80	Homo sapiens	73.916	2	2	106225	0
7143	1	O14792	O14792	Heparan sulfate glucosamine 3-O-sulfotransferase 1	HS3ST1	Homo sapiens	35.771	2	2	0	442228
7144	1	O14910	O14910	Protein lin-7 homolog A	LIN7A	Homo sapiens	25.999	6	2	117432	18756
7145	1	O14986	O14986	Phosphatidylinositol 4-phosphate 5-kinase type-1 beta	PIP5K1B	Homo sapiens	61.037	2	2	121817	0
7146	1	O15014	O15014	Zinc finger protein 609	ZNF609	Homo sapiens	151.193	2	2	704868	0
7147	1	O15069	O15069	NAC-alpha domain-containing protein 1	NACAD	Homo sapiens	161.104	2	2	76456	0
7148	1	O15116	O15116	U6 snRNA-associated Sm-like protein LSm1	LSM1	Homo sapiens	15.178	2	2	365085	129005
7149	1	O15119	O15119	T-box transcription factor TBX3	TBX3	Homo sapiens	79.39	2	2	758918	0
7150	1	O15169	O15169	Axin-1	AXIN1	Homo sapiens	95.636	2	2	79004	0
7151	1	O15182	O15182	Centrin-3	CETN3	Homo sapiens	19.548	2	2	125719	0
7152	1	O15194	O15194	CTD small phosphatase-like protein	CTDSPL	Homo sapiens	31.127	2	2	192788	114933
7153	1	O15217	O15217	Glutathione S-transferase A4	GSTA4	Homo sapiens	25.705	2	2	419397	0
7154	1	O15234	O15234	Protein CASC3	CASC3	Homo sapiens	76.279	2	2	108445	0
7155	1	O15235	O15235	28S ribosomal protein S12, mitochondrial	MRPS12	Homo sapiens	15.17	2	2	339740	70788
7156	1	O15417	O15417	Trinucleotide repeat-containing gene 18 protein	TNRC18	Homo sapiens	314.523	2	2	45959	0
7157	1	O15431	O15431	High affinity copper uptake protein 1	SLC31A1	Homo sapiens	21.09	2	2	393886	284490
7158	1	O43156	O43156	TELO2-interacting protein 1 homolog	TTI1	Homo sapiens	122.071	2	2	71500	17241
7159	1	O43281	O43281	Embryonal Fyn-associated substrate	EFS	Homo sapiens	58.815	2	2	158188	0
7160	1	O43295	O43295	SLIT-ROBO Rho GTPase-activating protein 3	SRGAP3	Homo sapiens	124.506	2	2	22925	0
7161	1	O43307	O43307	Rho guanine nucleotide exchange factor 9	ARHGEF9	Homo sapiens	60.981	2	2	199055	0
7162	1	O43312	O43312	Protein MTSS 1	MTSS1	Homo sapiens	82.251	2	2	103092	0
7163	1	O43504	O43504	Ragulator complex protein LAMTOR5	LAMTOR5	Homo sapiens	9.614	2	2	658807	791825
7164	1	O43521	O43521	Bcl-2-like protein 11	BCL2L11	Homo sapiens	22.172	2	2	15854	62165
7165	1	O43524	O43524	Forkhead box protein O3	FOXO3	Homo sapiens	71.277	2	2	104182	102355
7166	1	O43663	O43663	Protein regulator of cytokinesis 1	PRC1	Homo sapiens	71.608	2	2	89590	0
7167	1	O43674	O43674	NADH dehydrogenase [ubiquinone] 1 beta subcomplex subunit 5, mitochondrial	NDUFB5	Homo sapiens	21.751	2	2	6286890	1291794
7168	1	O43676	O43676	NADH dehydrogenase [ubiquinone] 1 beta subcomplex subunit 3	NDUFB3	Homo sapiens	11.4	2	2	8020270	1576034
7169	1	O43709	O43709	Probable 18S rRNA (guanine-N(7))-methyltransferase	BUD23	Homo sapiens	31.88	2	2	227960	0
7170	1	O43739	O43739	Cytohesin-3	CYTH3	Homo sapiens	46.346	2	2	129233	0
7171	1	O43759	O43759	Synaptogyrin-1	SYNGR1	Homo sapiens	25.456	2	2	491233	263885
7172	1	O43761	O43761	Synaptogyrin-3	SYNGR3	Homo sapiens	24.554	2	2	1142293	0
7173	1	O43805	O43805	Microtubule nucleation factor SSNA1	SSNA1	Homo sapiens	13.595	2	2	125500	31008
7174	1	O43820	O43820	Hyaluronidase-3	HYAL3	Homo sapiens	46.503	2	2	0	1298703
7175	1	O43929	O43929	Origin recognition complex subunit 4	ORC4	Homo sapiens	50.38	5	2	346201	183138
7176	1	O60266	O60266	Adenylate cyclase type 3	ADCY3	Homo sapiens	128.961	2	2	33845	0
7177	1	O60294	O60294	tRNA wybutosine-synthesizing protein 4	LCMT2	Homo sapiens	75.599	2	2	47203	0
7178	1	O60499	O60499	Syntaxin-10	STX10	Homo sapiens	28.114	2	2	327662	247983
7179	1	O60507	O60507	Protein-tyrosine sulfotransferase 1	TPST1	Homo sapiens	42.19	2	2	33452	35218
7180	1	O60602	O60602	Toll-like receptor 5	TLR5	Homo sapiens	97.834	2	2	0	17605
7181	1	O60636	O60636	Tetraspanin-2	TSPAN2	Homo sapiens	24.145	2	2	1527192	196148
7182	1	O60669	O60669	Monocarboxylate transporter 2	SLC16A7	Homo sapiens	52.199	2	2	161041	57533
7183	1	O60759	O60759	Cytohesin-interacting protein	CYTIP	Homo sapiens	40.011	2	2	0	133320
7184	1	O60831	O60831	PRA1 family protein 2	PRAF2	Homo sapiens	19.26	2	2	1696146	1067730

7185	1	O60888	O60888	Protein CutA	CUTA	Homo sapiens	19.114	2	2	760065	354662
7186	1	O60907	O60907	F-box-like/WD repeat-containing protein TBL1X	TBL1X	Homo sapiens	62.496	3	2	78624	25029
7187	1	O75144	O75144	ICOS ligand	ICOSLG	Homo sapiens	33.347	2	2	205076	0
7188	1	O75157	O75157	TSC22 domain family protein 2	TSC22D2	Homo sapiens	79.228	2	2	194492	47355
7189	1	O75164	O75164	Lysine-specific demethylase 4A	KDM4A	Homo sapiens	120.665	2	2	73532	23402
7190	1	O75185	O75185	Calcium-transporting ATPase type 2C member 2	ATP2C2	Homo sapiens	103.172	2	2	75473	0
7191	1	O75190	O75190	DnaJ homolog subfamily B member 6	DNAJB6	Homo sapiens	36.086	4	2	1062575	341299
7192	1	O75326	O75326	Semaphorin-7A	SEMA7A	Homo sapiens	74.824	2	2	18996	96672
7193	1	O75362	O75362	Zinc finger protein 217	ZNF217	Homo sapiens	115.274	2	2	61972	19083
7194	1	O75365	O75365	Protein tyrosine phosphatase type IVA 3	PTP4A3	Homo sapiens	19.532	2	2	46774	0
7195	1	O75386	O75386	Tubby-related protein 3	TULP3	Homo sapiens	49.644	2	2	116773	0
7196	1	O75387	O75387	Large neutral amino acids transporter small subunit 3	SLC43A1	Homo sapiens	61.474	2	2	87743	0
7197	1	O75394	O75394	39S ribosomal protein L33, mitochondrial	MRPL33	Homo sapiens	7.615	2	2	502757	0
7198	1	O75414	O75414	Nucleoside diphosphate kinase 6	NME6	Homo sapiens	21.142	2	2	261847	63060
7199	1	O75452	O75452	Retinol dehydrogenase 16	RDH16	Homo sapiens	35.674	2	2	0	138986
7200	1	O75525	O75525	KH domain-containing, RNA-binding, signal transduction-associated protein 3	KHDRBS3	Homo sapiens	38.798	2	2	258193	0
7201	1	O75528	O75528	Transcriptional adapter 3	TADA3	Homo sapiens	48.904	2	2	116737	0
7202	1	O75529	O75529	TAF5-like RNA polymerase II p300/CBP-associated factor-associated factor 65 kDa subunit 5L	TAF5L	Homo sapiens	66.155	2	2	102933	38869
7203	1	O75554	O75554	WW domain-binding protein 4	WBP4	Homo sapiens	42.506	2	2	264400	0
7204	1	O75581	O75581	Low-density lipoprotein receptor-related protein 6	LRP6	Homo sapiens	180.432	2	2	34856	0
7205	1	O75586	O75586	Mediator of RNA polymerase II transcription subunit 6	MED6	Homo sapiens	28.422	2	2	167512	96685
7206	1	O75607	O75607	Nucleoplasmin-3	NPM3	Homo sapiens	19.344	2	2	500376	265630
7207	1	O75629	O75629	Protein CREG1	CREG1	Homo sapiens	24.074	2	2	712757	683609
7208	1	O75683	O75683	Surfeit locus protein 6	SURF6	Homo sapiens	41.45	2	2	205295	0
7209	1	O75751	O75751	Solute carrier family 22 member 3	SLC22A3	Homo sapiens	61.282	2	2	638840	0
7210	1	O75880	O75880	Protein SCO1 homolog, mitochondrial	SCO1	Homo sapiens	33.813	2	2	107953	1535800
7211	1	O75907	O75907	Diacylglycerol O-acyltransferase 1	DGAT1	Homo sapiens	55.28	2	2	147186	0
7212	1	O75909	O75909	Cyclin-K	CCNK	Homo sapiens	64.242	2	2	571511	0
7213	1	O75928	O75928	E3 SUMO-protein ligase PIAS2	PIAS2	Homo sapiens	68.242	3	2	85216	16250
7214	1	O75954	O75954	Tetraspanin-9	TSPAN9	Homo sapiens	26.779	2	2	612569	187456
7215	1	O76054	O76054	SEC14-like protein 2	SEC14L2	Homo sapiens	46.144	2	2	123376	726599
7216	1	O94769	O94769	Extracellular matrix protein 2	ECM2	Homo sapiens	79.79	2	2	39547	28081
7217	1	O94823	O94823	Phospholipid-transporting ATPase VB	ATP10B	Homo sapiens	165.393	2	2	159357	0
7218	1	O94933	O94933	SLIT and NTRK-like protein 3	SLITRK3	Homo sapiens	108.934	2	2	128162	0
7219	1	O94956	O94956	Solute carrier organic anion transporter family member 2B1	SLCO2B1	Homo sapiens	76.713	2	2	235773	235711
7220	1	O94989	O94989	Rho guanine nucleotide exchange factor 15	ARHGEF15	Homo sapiens	91.943	2	2	62717	52621
7221	1	O95072	O95072	Meiotic recombination protein REC8 homolog	REC8	Homo sapiens	62.616	2	2	0	214414
7222	1	O95147	O95147	Dual specificity protein phosphatase 14	DUSP14	Homo sapiens	22.254	2	2	49570	0
7223	1	O95197	O95197	Reticulon-3	RTN3	Homo sapiens	112.616	2	2	812649	2463791
7224	1	O95198	O95198	Kelch-like protein 2	KLHL2	Homo sapiens	65.977	2	2	0	78913
7225	1	O95298	O95298	NADH dehydrogenase [ubiquinone] 1 subunit C2	NDUFC2	Homo sapiens	14.185	5	2	989429	255314
7226	1	O95429	O95429	BAG family molecular chaperone regulator 4	BAG4	Homo sapiens	49.595	2	2	117857	76217
7227	1	O95450	O95450	A disintegrin and metalloproteinase with thrombospondin motifs 2	ADAMTS2	Homo sapiens	134.756	2	2	0	75785
7228	1	O95528	O95528	Solute carrier family 2, facilitated glucose transporter member 10	SLC2A10	Homo sapiens	56.912	2	2	13431	128710
7229	1	O95563	O95563	Mitochondrial pyruvate carrier 2	MPC2	Homo sapiens	14.276	2	2	758768	58771
7230	1	O95644	O95644	Nuclear factor of activated T-cells, cytoplasmic 1	NFATC1	Homo sapiens	101.242	2	2	238560	107115

7231	1	O95696	O95696	Bromodomain-containing protein 1	BRD1	Homo sapiens	119.523	2	2	278513	21471
7232	1	O95777	O95777	U6 snRNA-associated Sm-like protein LSm8	LSM8	Homo sapiens	10.399	2	2	3927210	1464980
7233	1	O95837	O95837	Guanine nucleotide-binding protein subunit alpha-14	GNA14	Homo sapiens	41.573	2	2	187545	0
7234	1	O95848	O95848	Uridine diphosphate glucose pyrophosphatase NUDT14	NUDT14	Homo sapiens	24.119	2	2	234220	0
7235	1	O95866	O95866	Megakaryocyte and platelet inhibitory receptor G6b	MPIG6B	Homo sapiens	26.162	2	2	0	232642
7236	1	O95873	O95873	Uncharacterized protein C6orf47	C6orf47	Homo sapiens	31.709	2	2	488183	59502
7237	1	O95977	O95977	Sphingosine 1-phosphate receptor 4	S1PR4	Homo sapiens	41.626	2	2	0	118122
7238	1	P00325	P00325	All-trans-retinol dehydrogenase [NAD(+)] ADH1B	ADH1B	Homo sapiens	39.836	11	2	35605560	1018905
7239	1	P00367	P00367	Glutamate dehydrogenase 1, mitochondrial	GLUD1	Homo sapiens	61.398	21	2	1098950	418168
7240	1	P00374	P00374	Dihydrofolate reductase	DHFR	Homo sapiens	21.452	2	2	153143	0
7241	1	P00414	P00414	Cytochrome c oxidase subunit 3	MT-CO3	Homo sapiens	29.951	2	2	1990013	247121
7242	1	P00846	P00846	ATP synthase subunit a	MT-ATP6	Homo sapiens	24.818	2	2	2614406	517319
7243	1	P00995	P00995	Serine protease inhibitor Kazal-type 1	SPINK1	Homo sapiens	8.505	2	2	176845	0
7244	1	P01112	P01112	GTPase HRas	HRAS	Homo sapiens	21.298	2	2	1071795	205108
7245	1	P01130	P01130	Low-density lipoprotein receptor	LDLR	Homo sapiens	95.379	2	2	942812	51503
7246	1	P01599	P01599	Immunoglobulin kappa variable 1-17	IGKV1-17	Homo sapiens	12.778	2	2	2041185	3576902
7247	1	P01602	P01602	Immunoglobulin kappa variable 1-5	IGKV1-5	Homo sapiens	12.782	2	2	12942200	22067796
7248	1	P01703	P01703	Immunoglobulin lambda variable 1-40	IGLV1-40	Homo sapiens	12.301	2	2	4140225	7363105
7249	1	P01714	P01714	Immunoglobulin lambda variable 3-19	IGLV3-19	Homo sapiens	12.041	2	2	2273685	4116253
7250	1	P01730	P01730	T-cell surface glycoprotein CD4	CD4	Homo sapiens	51.112	2	2	133653	8952
7251	1	P01743	P01743	Immunoglobulin heavy variable 1-46	IGHV1-46	Homo sapiens	12.93	3	2	546507	576621
7252	1	P01766	P01766	Immunoglobulin heavy variable 3-13	IGHV3-13	Homo sapiens	12.506	2	2	510967	959894
7253	2	P01782;P0DP04	P01782	Immunoglobulin heavy variable 3-9	IGHV3-9	Homo sapiens	12.945	2	2	973622	1190347
7254	1	P02656	P02656	Apolipoprotein C-III	APOC3	Homo sapiens	10.853	2	2	3598744	10864060
7255	1	P04271	P04271	Protein S100-B	S100B	Homo sapiens	10.709	2	2	2437319	0
7256	1	P04350	P04350	Tubulin beta-4A chain	TUBB4A	Homo sapiens	49.585	2	2	633995	0
7257	1	P04440	P04440	HLA class II histocompatibility antigen, DP beta 1 chain	HLA-DPB1	Homo sapiens	29.158	2	2	1086384	1848953
7258	1	P04818	P04818	Thymidylate synthase	TYMS	Homo sapiens	35.715	2	2	38595	29206
7259	1	P05231	P05231	Interleukin-6	IL6	Homo sapiens	23.717	2	2	0	180430
7260	1	P05388	P05388	60S acidic ribosomal protein P0	RPLP0	Homo sapiens	34.272	10	2	71516	0
7261	1	P05412	P05412	Transcription factor Jun	JUN	Homo sapiens	35.676	2	2	639468	36064
7262	1	P05814	P05814	Beta-casein	CSN2	Homo sapiens	25.383	2	2	1068770	2240245
7263	1	P05976	P05976	Myosin light chain 1/3, skeletal muscle isoform	MYL1	Homo sapiens	21.145	2	2	35314	126372
7264	1	P06239	P06239	Tyrosine-protein kinase Lck	LCK	Homo sapiens	57.999	2	2	60623	0
7265	1	P07305	P07305	Histone H1.0	H1-0	Homo sapiens	20.861	2	2	78018100	8472140
7266	1	P07327	P07327	Alcohol dehydrogenase 1A	ADH1A	Homo sapiens	39.858	2	2	0	106423
7267	1	P07333	P07333	Macrophage colony-stimulating factor 1 receptor	CSF1R	Homo sapiens	107.986	2	2	82416	0
7268	1	P07498	P07498	Kappa-casein	CSN3	Homo sapiens	20.303	2	2	388310	1078396
7269	1	P07947	P07947	Tyrosine-protein kinase Yes	YES1	Homo sapiens	60.8	2	2	727217	243333
7270	1	P08047	P08047	Transcription factor Sp1	SP1	Homo sapiens	80.695	2	2	298565	430487
7271	1	P08247	P08247	Synaptophysin	SYP	Homo sapiens	33.845	2	2	1008837	0
7272	2	P08263;P09210	P08263	Glutathione S-transferase A1	GSTA1	Homo sapiens	25.629	2	2	441179	21607
7273	1	P08493	P08493	Matrix Gla protein	MGP	Homo sapiens	12.351	2	2	1358859	269525
7274	1	P08590	P08590	Myosin light chain 3	MYL3	Homo sapiens	21.931	2	2	116619	17902
7275	1	P08637	P08637	Low affinity immunoglobulin gamma Fc region receptor III-A	FCGR3A	Homo sapiens	29.089	3	2	347460	2484752
7276	1	P08865	P08865	40S ribosomal protein SA	RPSA	Homo sapiens	32.852	9	2	10307286	2576178
7277	1	P09234	P09234	U1 small nuclear ribonucleoprotein C	SNRPC	Homo sapiens	17.393	2	2	1526440	404191
7278	1	P09237	P09237	Matrilysin	MMP7	Homo sapiens	29.674	2	2	0	178677

7279	1	P09488	P09488	Glutathione S-transferase Mu 1	GSTM1	Homo sapiens	25.713	2	2	409215	65349
7280	1	P0CAP2	P0CAP2	DNA-directed RNA polymerase II subunit GRINL1A	POLR2M	Homo sapiens	41.739	2	2	270503	0
7281	1	P0CJ78	P0CJ78	Zinc finger protein 865	ZNF865	Homo sapiens	111.078	2	2	72551	0
7282	2	P0CW19;P0CW20	P0CW19	LIM and senescent cell antigen-like-containing domain protein 3	LIMS3	Homo sapiens	13.252	2	2	163556	53464
7283	1	P0DJ93	P0DJ93	Small integral membrane protein 13	SMIM13	Homo sapiens	10.348	2	2	66571	0
7284	1	P10071	P10071	Transcriptional activator GLI3	GLI3	Homo sapiens	169.862	2	2	79139	0
7285	1	P10082	P10082	Peptide YY	PYY	Homo sapiens	11.144	2	2	1406049	0
7286	1	P10109	P10109	Adrenodoxin, mitochondrial	FDX1	Homo sapiens	19.391	2	2	378237	182898
7287	1	P10145	P10145	Interleukin-8	CXCL8	Homo sapiens	11.097	2	2	0	4275240
7288	1	P10415	P10415	Apoptosis regulator Bcl-2	BCL2	Homo sapiens	26.263	2	2	85471	0
7289	1	P11169	P11169	Solute carrier family 2, facilitated glucose transporter member 3	SLC2A3	Homo sapiens	53.925	5	2	289056	4973061
7290	1	P11234	P11234	Ras-related protein Ral-B	RALB	Homo sapiens	23.406	2	2	2382272	3798091
7291	1	P11245	P11245	Arylamine N-acetyltransferase 2	NAT2	Homo sapiens	33.572	2	2	107385	0
7292	1	P11597	P11597	Cholesteryl ester transfer protein	CETP	Homo sapiens	54.757	2	2	0	343341
7293	2	P11712;P33261	P11712	Cytochrome P450 2C9	CYP2C9	Homo sapiens	55.629	2	2	0	238951
7294	1	P11831	P11831	Serum response factor	SRF	Homo sapiens	51.593	2	2	434681	0
7295	1	P12074	P12074	Cytochrome c oxidase subunit 6A1, mitochondrial	COX6A1	Homo sapiens	12.155	2	2	5097169	298779
7296	1	P12107	P12107	Collagen alpha-1(XI) chain	COL11A1	Homo sapiens	181.067	2	2	0	147629
7297	1	P12271	P12271	Retinaldehyde-binding protein 1	RLBP1	Homo sapiens	36.475	2	2	117541	0
7298	1	P12318	P12318	Low affinity immunoglobulin gamma Fc region receptor II-a	FCGR2A	Homo sapiens	35.001	4	2	66181	407713
7299	1	P12724	P12724	Eosinophil cationic protein	RNASE3	Homo sapiens	18.384	2	2	1576110	12448060
7300	1	P12882	P12882	Myosin-1	MYH1	Homo sapiens	223.15	2	2	98179	114756
7301	1	P13056	P13056	Nuclear receptor subfamily 2 group C member 1	NR2C1	Homo sapiens	67.317	2	2	67848	0
7302	1	P13196	P13196	5-aminolevulinate synthase, non-specific, mitochondrial	ALAS1	Homo sapiens	70.579	2	2	25298	61630
7303	1	P13498	P13498	Cytochrome b-245 light chain	CYBA	Homo sapiens	21.011	2	2	893184	10505126
7304	1	P13598	P13598	Intercellular adhesion molecule 2	ICAM2	Homo sapiens	30.654	2	2	112742	126939
7305	1	P13726	P13726	Tissue factor	F3	Homo sapiens	33.067	2	2	107708	99660
7306	1	P14151	P14151	L-selectin	SELL	Homo sapiens	42.187	2	2	92965	264240
7307	1	P14209	P14209	CD99 antigen	CD99	Homo sapiens	18.846	2	2	7289637	797649
7308	1	P14406	P14406	Cytochrome c oxidase subunit 7A2, mitochondrial	COX7A2	Homo sapiens	9.396	2	2	5330560	706819
7309	1	P14415	P14415	Sodium/potassium-transporting ATPase subunit beta-2	ATP1B2	Homo sapiens	33.368	2	2	250093	0
7310	1	P15090	P15090	Fatty acid-binding protein, adipocyte	FABP4	Homo sapiens	14.716	3	2	1498107	0
7311	1	P15291	P15291	Beta-1,4-galactosyltransferase 1	B4GALT1	Homo sapiens	43.921	2	2	406713	936810
7312	1	P15559	P15559	NAD(P)H dehydrogenase [quinone] 1	NQO1	Homo sapiens	30.867	2	2	275555	422181
7313	1	P16112	P16112	Aggrecan core protein	ACAN	Homo sapiens	261.33	2	2	262636	80303
7314	1	P16150	P16150	Leukosialin	SPN	Homo sapiens	40.323	2	2	0	770805
7315	1	P16220	P16220	Cyclic AMP-responsive element-binding protein 1	CREB1	Homo sapiens	35.135	3	2	343365	151292
7316	1	P16519	P16519	Neuroendocrine convertase 2	PCSK2	Homo sapiens	70.566	2	2	67699	0
7317	1	P16662	P16662	UDP-glucuronosyltransferase 2B7	UGT2B7	Homo sapiens	60.721	2	2	73756	54331
7318	1	P17066	P17066	Heat shock 70 kDa protein 6	HSPA6	Homo sapiens	71.031	5	2	0	289431
7319	1	P17096	P17096	High mobility group protein HMG-I/HMG-Y	HMGA1	Homo sapiens	11.675	2	2	2899309	3311121
7320	1	P17152	P17152	Transmembrane protein 11, mitochondrial	TMEM11	Homo sapiens	21.542	2	2	917700	369109
7321	1	P17302	P17302	Gap junction alpha-1 protein	GJA1	Homo sapiens	43.006	2	2	213407	91613
7322	1	P17535	P17535	Transcription factor JunD	JUND	Homo sapiens	35.175	2	2	174158	18673
7323	1	P17947	P17947	Transcription factor PU.1	SPI1	Homo sapiens	31.081	2	2	0	282498
7324	1	P18054	P18054	Polyunsaturated fatty acid lipoxygenase ALOX12	ALOX12	Homo sapiens	75.694	2	2	0	135451
7325	1	P18850	P18850	Cyclic AMP-dependent transcription factor ATF-6 alpha	ATF6	Homo sapiens	74.586	2	2	30066	21448
7326	1	P19440	P19440	Glutathione hydrolase 1 proenzyme	GGT1	Homo sapiens	61.41	8	2	0	219202

7327	1	P19532	P19532	Transcription factor E3	TFE3	Homo sapiens	61.522	2	2	59753	0
7328	1	P20290	P20290	Transcription factor BTF3	BTF3	Homo sapiens	22.168	2	2	1139672	159760
7329	1	P20309	P20309	Muscarinic acetylcholine receptor M3	CHRM3	Homo sapiens	66.127	2	2	93749	0
7330	1	P20337	P20337	Ras-related protein Rab-3B	RAB3B	Homo sapiens	24.757	2	2	365399	120230
7331	1	P20749	P20749	B-cell lymphoma 3 protein	BCL3	Homo sapiens	47.583	2	2	58353	45521
7332	1	P20823	P20823	Hepatocyte nuclear factor 1-alpha	HNF1A	Homo sapiens	67.355	3	2	123983	0
7333	1	P20933	P20933	N(4)-(beta-N-acetylglucosaminyl)-L-asparaginase	AGA	Homo sapiens	37.209	2	2	837723	529140
7334	1	P21217	P21217	3-galactosyl-N-acetylglucosaminide 4-alpha-L-fucosyltransferase FUT3	FUT3	Homo sapiens	42.118	8	2	186075	0
7335	1	P21462	P21462	fMet-Leu-Phe receptor	FPR1	Homo sapiens	38.444	2	2	0	1323626
7336	1	P21673	P21673	Diamine acetyltransferase 1	SAT1	Homo sapiens	20.025	2	2	0	174463
7337	1	P21675	P21675	Transcription initiation factor TFIID subunit 1	TAF1	Homo sapiens	212.679	7	2	128724	0
7338	1	P21741	P21741	Midkine	MDK	Homo sapiens	15.584	2	2	583152	47933
7339	1	P21854	P21854	B-cell differentiation antigen CD72	CD72	Homo sapiens	40.221	2	2	0	125698
7340	1	P22304	P22304	Iduronate 2-sulfatase	IDS	Homo sapiens	61.874	2	2	80366	131134
7341	1	P22392	P22392	Nucleoside diphosphate kinase B	NME2	Homo sapiens	17.3	9	2	558949	359761
7342	1	P22466	P22466	Galanin peptides	GAL	Homo sapiens	13.301	2	2	139413	0
7343	1	P22732	P22732	Solute carrier family 2, facilitated glucose transporter member 5	SLC2A5	Homo sapiens	54.974	2	2	65233	280934
7344	1	P22736	P22736	Nuclear receptor subfamily 4 group A member 1	NR4A1	Homo sapiens	64.466	2	2	146489	0
7345	1	P23434	P23434	Glycine cleavage system H protein, mitochondrial	GCSH	Homo sapiens	18.885	2	2	620906	28893
7346	1	P23511	P23511	Nuclear transcription factor Y subunit alpha	NFYA	Homo sapiens	36.874	2	2	243682	65247
7347	1	P24071	P24071	Immunoglobulin alpha Fc receptor	FCAR	Homo sapiens	32.265	2	2	0	5533062
7348	1	P25024	P25024	C-X-C chemokine receptor type 1	CXCR1	Homo sapiens	39.789	3	2	0	318437
7349	1	P25025	P25025	C-X-C chemokine receptor type 2	CXCR2	Homo sapiens	40.758	2	2	0	600575
7350	1	P25090	P25090	N-formyl peptide receptor 2	FPR2	Homo sapiens	38.964	2	2	0	1283020
7351	1	P25106	P25106	Atypical chemokine receptor 3	ACKR3	Homo sapiens	41.494	2	2	90737	0
7352	1	P25208	P25208	Nuclear transcription factor Y subunit beta	NFYB	Homo sapiens	22.83	2	2	361135	142840
7353	1	P25815	P25815	Protein S100-P	S100P	Homo sapiens	10.4	2	2	2229111	66535070
7354	1	P25940	P25940	Collagen alpha-3(V) chain	COL5A3	Homo sapiens	172.122	2	2	501472	136305
7355	1	P25942	P25942	Tumor necrosis factor receptor superfamily member 5	CD40	Homo sapiens	30.619	2	2	0	233148
7356	1	P28039	P28039	Acyloxyacyl hydrolase	AOAH	Homo sapiens	65.105	2	2	152750	319581
7357	1	P28067	P28067	HLA class II histocompatibility antigen, DM alpha chain	HLA-DMA	Homo sapiens	29.194	2	2	48804	36674
7358	1	P28906	P28906	Hematopoietic progenitor cell antigen CD34	CD34	Homo sapiens	40.716	2	2	2390085	281565
7359	1	P30049	P30049	ATP synthase subunit delta, mitochondrial	ATP5F1D	Homo sapiens	17.491	2	2	3989997	484075
7360	1	P30273	P30273	High affinity immunoglobulin epsilon receptor subunit gamma	FCER1G	Homo sapiens	9.667	2	2	379997	8028540
7361	1	P30825	P30825	High affinity cationic amino acid transporter 1	SLC7A1	Homo sapiens	67.638	3	2	169130	99303
7362	1	P31151	P31151	Protein S100-A7	S100A7	Homo sapiens	11.47	4	2	0	5154150
7363	1	P31271	P31271	Homeobox protein Hox-A13	HOXA13	Homo sapiens	39.726	3	2	119036	0
7364	1	P31513	P31513	Flavin-containing monooxygenase 3	FMO3	Homo sapiens	60.036	2	2	0	122958
7365	1	P31641	P31641	Sodium- and chloride-dependent taurine transporter	SLC6A6	Homo sapiens	69.832	2	2	0	81004
7366	1	P32780	P32780	General transcription factor IIH subunit 1	GTF2H1	Homo sapiens	62.03	2	2	89808	45248
7367	1	P33981	P33981	Dual specificity protein kinase TTK	TTK	Homo sapiens	97.073	2	2	87603	0
7368	1	P34931	P34931	Heat shock 70 kDa protein 1-like	HSPA1L	Homo sapiens	70.374	9	2	540106	126305
7369	1	P35030	P35030	Trypsin-3	PRSS3	Homo sapiens	32.532	2	2	517921	645460
7370	1	P35268	P35268	60S ribosomal protein L22	RPL22	Homo sapiens	14.786	2	2	49676200	17003240
7371	1	P35520	P35520	Cystathionine beta-synthase	CBS	Homo sapiens	60.588	2	2	0	38008
7372	1	P35626	P35626	Beta-adrenergic receptor kinase 2	GRK3	Homo sapiens	79.71	2	2	116858	99478
7373	1	P35680	P35680	Hepatocyte nuclear factor 1-beta	HNF1B	Homo sapiens	61.325	2	2	29480	0
7374	1	P35754	P35754	Glutaredoxin-1	GLRX	Homo sapiens	11.774	2	2	197493	1255898

7375	1	P35790	P35790	Choline kinase alpha	CHKA	Homo sapiens	52.248	2	2	46195	0
7376	1	P36021	P36021	Monocarboxylate transporter 8	SLC16A2	Homo sapiens	59.51	2	2	171160	0
7377	1	P36894	P36894	Bone morphogenetic protein receptor type-1A	BMPR1A	Homo sapiens	60.198	3	2	141920	0
7378	1	P36954	P36954	DNA-directed RNA polymerase II subunit RPB9	POLR2I	Homo sapiens	14.523	2	2	183777	0
7379	1	P40425	P40425	Pre-B-cell leukemia transcription factor 2	PBX2	Homo sapiens	45.883	6	2	102601	50997
7380	1	P40426	P40426	Pre-B-cell leukemia transcription factor 3	PBX3	Homo sapiens	47.19	2	2	127029	9164
7381	1	P41091	P41091	Eukaryotic translation initiation factor 2 subunit 3	EIF2S3	Homo sapiens	51.109	13	2	4879899	4106492
7382	1	P41236	P41236	Protein phosphatase inhibitor 2	PPP1R2	Homo sapiens	23.013	6	2	1065685	159757
7383	1	P42575	P42575	Caspase-2	CASP2	Homo sapiens	50.684	2	2	355348	0
7384	1	P42766	P42766	60S ribosomal protein L35	RPL35	Homo sapiens	14.551	2	2	5206739	2318810
7385	1	P43004	P43004	Excitatory amino acid transporter 2	SLC1A2	Homo sapiens	62.105	2	2	1790176	92457
7386	1	P43307	P43307	Translocon-associated protein subunit alpha	SSR1	Homo sapiens	32.233	2	2	5405821	6628995
7387	1	P45877	P45877	Peptidyl-prolyl cis-trans isomerase C	PPIC	Homo sapiens	22.764	2	2	861334	479820
7388	1	P45983	P45983	Mitogen-activated protein kinase 8	MAPK8	Homo sapiens	48.294	4	2	182388	35867
7389	1	P47224	P47224	Guanine nucleotide exchange factor MSS4	RABIF	Homo sapiens	13.839	2	2	572881	706896
7390	1	P47900	P47900	P2Y purinoceptor 1	P2RY1	Homo sapiens	42.073	2	2	95415	0
7391	1	P47914	P47914	60S ribosomal protein L29	RPL29	Homo sapiens	17.751	2	2	7688908	2175190
7392	1	P47974	P47974	mRNA decay activator protein ZFP36L2	ZFP36L2	Homo sapiens	51.064	2	2	129362	0
7393	1	P47985	P47985	Cytochrome b-c1 complex subunit Rieske, mitochondrial	UQCRFS1	Homo sapiens	29.667	6	2	4694987	1124349
7394	1	P48509	P48509	CD151 antigen	CD151	Homo sapiens	28.296	2	2	2788527	606069
7395	1	P49207	P49207	60S ribosomal protein L34	RPL34	Homo sapiens	13.293	2	2	1798786	563796
7396	1	P49279	P49279	Natural resistance-associated macrophage protein 1	SLC11A1	Homo sapiens	59.871	2	2	0	244709
7397	1	P49459	P49459	Ubiquitin-conjugating enzyme E2 A	UBE2A	Homo sapiens	17.314	2	2	433046	66539
7398	1	P49674	P49674	Casein kinase I isoform epsilon	CSNK1E	Homo sapiens	47.316	2	2	195392	33270
7399	1	P49760	P49760	Dual specificity protein kinase CLK2	CLK2	Homo sapiens	60.092	2	2	24915	0
7400	1	P49768	P49768	Presenilin-1	PSEN1	Homo sapiens	52.668	3	2	221984	138525
7401	1	P50607	P50607	Tubby protein homolog	TUB	Homo sapiens	55.652	2	2	69423	0
7402	1	P50747	P50747	Biotin--protein ligase	HLCS	Homo sapiens	80.761	2	2	222416	0
7403	1	P51161	P51161	Gastrotropin	FABP6	Homo sapiens	14.37	2	2	0	205386
7404	1	P51397	P51397	Death-associated protein 1	DAP	Homo sapiens	11.166	2	2	652535	59174
7405	1	P51795	P51795	H(+)/Cl(-) exchange transporter 5	CLCN5	Homo sapiens	90.785	2	2	80402	0
7406	1	P51805	P51805	Plexin-A3	PLXNA3	Homo sapiens	207.719	2	2	27879	0
7407	1	P51808	P51808	Dynein light chain Tctex-type 3	DYNLT3	Homo sapiens	13.061	2	2	447402	0
7408	1	P51825	P51825	AF4/FMR2 family member 1	AFF1	Homo sapiens	131.422	2	2	89376	0
7409	1	P51957	P51957	Serine/threonine-protein kinase Nek4	NEK4	Homo sapiens	94.597	2	2	38948	0
7410	3	P51965;Q969T4;Q	P51965	Ubiquitin-conjugating enzyme E2 E1	UBE2E1	Homo sapiens	21.405	2	2	653734	293484
7411	1	P52298	P52298	Nuclear cap-binding protein subunit 2	NCBP2	Homo sapiens	18.001	2	2	588982	104950
7412	3	P52435;Q9GZM3;	P52435	DNA-directed RNA polymerase II subunit RPB11-a	POLR2J	Homo sapiens	13.293	2	2	25568	73199
7413	1	P52739	P52739	Zinc finger protein 131	ZNF131	Homo sapiens	71.423	2	2	36960	0
7414	1	P52758	P52758	2-iminobutanoate/2-iminopropanoate deaminase	RIDA	Homo sapiens	14.494	2	2	1567515	461215
7415	1	P52895	P52895	Aldo-keto reductase family 1 member C2	AKR1C2	Homo sapiens	36.737	5	2	193417	784281
7416	1	P52943	P52943	Cysteine-rich protein 2	CRIP2	Homo sapiens	22.491	2	2	6690449	437196
7417	1	P53674	P53674	Beta-crystallin B1	CRYBB1	Homo sapiens	28.022	2	2	50625	19362
7418	1	P53805	P53805	Calcipressin-1	RCAN1	Homo sapiens	28.078	2	2	239927	77040
7419	1	P54277	P54277	PMS1 protein homolog 1	PMS1	Homo sapiens	105.832	2	2	49890	0
7420	1	P54750	P54750	Dual specificity calcium/calmodulin-dependent 3',5'-cyclic nucleotide phosphodiesterase 1A	PDE1A	Homo sapiens	61.253	3	2	227585	0
7421	1	P55040	P55040	GTP-binding protein GEM	GEM	Homo sapiens	33.947	2	2	117321	0

7422	1	P55064	P55064	Aquaporin-5	AQP5	Homo sapiens	28.291	2	2	0	203825
7423	1	P55085	P55085	Proteinase-activated receptor 2	F2RL1	Homo sapiens	44.126	2	2	189519	0
7424	1	P55317	P55317	Hepatocyte nuclear factor 3-alpha	FOXA1	Homo sapiens	49.15	2	2	50610	0
7425	1	P55347	P55347	Homeobox protein PKNOX1	PKNOX1	Homo sapiens	47.606	2	2	150535	57214
7426	1	P56134	P56134	ATP synthase subunit f, mitochondrial	ATP5MF	Homo sapiens	10.916	2	2	21432070	3362810
7427	1	P58166	P58166	Inhibin beta E chain	INHBE	Homo sapiens	38.56	2	2	0	98688
7428	1	P58546	P58546	Myotrophin	MTPN	Homo sapiens	12.894	2	2	778169	980140
7429	1	P59190	P59190	Ras-related protein Rab-15	RAB15	Homo sapiens	24.391	2	2	132232	43715600
7430	1	P59780	P59780	AP-3 complex subunit sigma-2	AP3S2	Homo sapiens	22.018	2	2	221456	39263
7431	1	P61088	P61088	Ubiquitin-conjugating enzyme E2 N	UBE2N	Homo sapiens	17.139	5	2	1136140	257762
7432	1	P61254	P61254	60S ribosomal protein L26	RPL26	Homo sapiens	17.259	2	2	3247256	1184060
7433	1	P61513	P61513	60S ribosomal protein L37a	RPL37A	Homo sapiens	10.273	3	2	993138	177211
7434	1	P61812	P61812	Transforming growth factor beta-2 proprotein	TGFB2	Homo sapiens	47.747	2	2	47451	39871
7435	1	P61952	P61952	Guanine nucleotide-binding protein G(I)/G(S)/G(O) subunit gamma-11	GNG11	Homo sapiens	8.48	2	2	241872	0
7436	1	P61960	P61960	Ubiquitin-fold modifier 1	UFM1	Homo sapiens	9.116	2	2	4114165	726554
7437	1	P61970	P61970	Nuclear transport factor 2	NUTF2	Homo sapiens	14.48	2	2	928847	172260
7438	1	P62072	P62072	Mitochondrial import inner membrane translocase subunit Tim10	TIMM10	Homo sapiens	10.332	2	2	599210	128002
7439	1	P62253	P62253	Ubiquitin-conjugating enzyme E2 G1	UBE2G1	Homo sapiens	19.509	2	2	251907	114753
7440	1	P62256	P62256	Ubiquitin-conjugating enzyme E2 H	UBE2H	Homo sapiens	20.654	2	2	1094564	341348
7441	1	P62273	P62273	40S ribosomal protein S29	RPS29	Homo sapiens	6.675	2	2	798143	64331
7442	1	P62304	P62304	Small nuclear ribonucleoprotein E	SNRPE	Homo sapiens	10.8	2	2	5651434	2275983
7443	1	P62306	P62306	Small nuclear ribonucleoprotein F	SNRPF	Homo sapiens	9.722	2	2	3535335	1103350
7444	1	P62312	P62312	U6 snRNA-associated Sm-like protein LSm6	LSM6	Homo sapiens	9.125	2	2	1618224	938803
7445	1	P62341	P62341	Thioredoxin reductase-like selenoprotein T	SELENOT	Homo sapiens	22.293	2	2	651364	456849
7446	1	P62380	P62380	TATA box-binding protein-like 1	TBPL1	Homo sapiens	20.886	2	2	95150	0
7447	1	P62714	P62714	Serine/threonine-protein phosphatase 2A catalytic subunit beta isoform	PPP2CB	Homo sapiens	35.575	10	2	927903	331082
7448	1	P62877	P62877	E3 ubiquitin-protein ligase RBX1	RBX1	Homo sapiens	12.275	2	2	1016524	890642
7449	1	P62879	P62879	Guanine nucleotide-binding protein G(I)/G(S)/G(T) subunit beta-2	GNB2	Homo sapiens	37.331	2	2	4797095	2138052
7450	1	P63098	P63098	Calcineurin subunit B type 1	PPP3R1	Homo sapiens	19.299	3	2	1788470	1015012
7451	1	P63146	P63146	Ubiquitin-conjugating enzyme E2 B	UBE2B	Homo sapiens	17.312	2	2	323509	129432
7452	1	P63173	P63173	60S ribosomal protein L38	RPL38	Homo sapiens	8.215	2	2	12063580	5431800
7453	1	P68036	P68036	Ubiquitin-conjugating enzyme E2 L3	UBE2L3	Homo sapiens	17.862	4	2	1730098	838284
7454	1	P68106	P68106	Peptidyl-prolyl cis-trans isomerase FKBP1B	FKBP1B	Homo sapiens	11.781	2	2	150436	0
7455	1	P68400	P68400	Casein kinase II subunit alpha	CSNK2A1	Homo sapiens	45.142	12	2	420720	594221
7456	1	P68402	P68402	Platelet-activating factor acetylhydrolase IB subunit alpha2	PAFAH1B2	Homo sapiens	25.567	2	2	1926110	727348
7457	1	P69891	P69891	Hemoglobin subunit gamma-1	HBG1	Homo sapiens	16.142	8	2	2517094	2141035
7458	1	P78368	P78368	Casein kinase I isoform gamma-2	CSNK1G2	Homo sapiens	47.458	6	2	168422	2231996
7459	1	P78381	P78381	UDP-galactose translocator	SLC35A2	Homo sapiens	41.307	2	2	799825	143818
7460	1	P78383	P78383	Solute carrier family 35 member B1	SLC35B1	Homo sapiens	35.76	2	2	139495	34936
7461	1	P78545	P78545	ETS-related transcription factor Elf-3	ELF3	Homo sapiens	41.456	2	2	102534	0
7462	1	P78552	P78552	Interleukin-13 receptor subunit alpha-1	IL13RA1	Homo sapiens	48.76	2	2	23324	21263
7463	1	P84157	P84157	Matrix-remodeling-associated protein 7	MXRA7	Homo sapiens	21.466	2	2	1772579	242017
7464	1	Q00613	Q00613	Heat shock factor protein 1	HSF1	Homo sapiens	57.261	2	2	303677	101557
7465	1	Q01064	Q01064	Dual specificity calcium/calmodulin-dependent 3',5'-cyclic nucleotide phosphodiesterase 1B	PDE1B	Homo sapiens	61.381	2	2	73852	0
7466	1	Q01130	Q01130	Serine/arginine-rich splicing factor 2	SRSF2	Homo sapiens	25.476	2	2	1173700	480090
7467	1	Q01196	Q01196	Runt-related transcription factor 1	RUNX1	Homo sapiens	48.738	2	2	0	260079
7468	1	Q02539	Q02539	Histone H1.1	H1-1	Homo sapiens	21.841	2	2	245135	159206

7469	1	Q02985	Q02985	Complement factor H-related protein 3	CFHR3	Homo sapiens	37.323	5	2	134081	1022327
7470	1	Q03111	Q03111	Protein ENL	MLLT1	Homo sapiens	62.055	2	2	196705	0
7471	1	Q03591	Q03591	Complement factor H-related protein 1	CFHR1	Homo sapiens	37.653	4	2	2392940	12121422
7472	1	Q04725	Q04725	Transducin-like enhancer protein 2	TLE2	Homo sapiens	79.843	2	2	97030	0
7473	1	Q04771	Q04771	Activin receptor type-1	ACVR1	Homo sapiens	57.154	2	2	194451	17629
7474	1	Q04941	Q04941	Proteolipid protein 2	PLP2	Homo sapiens	16.691	2	2	7542830	7641900
7475	1	Q05513	Q05513	Protein kinase C zeta type	PRKCZ	Homo sapiens	67.662	2	2	138278	46831
7476	1	Q07002	Q07002	Cyclin-dependent kinase 18	CDK18	Homo sapiens	54.425	2	2	214083	0
7477	1	Q07817	Q07817	Bcl-2-like protein 1	BCL2L1	Homo sapiens	26.049	2	2	416385	270579
7478	1	Q07820	Q07820	Induced myeloid leukemia cell differentiation protein Mcl-1	MCL1	Homo sapiens	37.338	2	2	224046	47636
7479	1	Q08116	Q08116	Regulator of G-protein signaling 1	RGS1	Homo sapiens	23.855	2	2	167998	0
7480	1	Q08334	Q08334	Interleukin-10 receptor subunit beta	IL10RB	Homo sapiens	36.995	2	2	165936	108836
7481	1	Q08AE8	Q08AE8	Protein spire homolog 1	SPIRE1	Homo sapiens	85.546	2	2	334891	68467
7482	2	Q08AH3;Q68CK6	Q08AH3	Acyl-coenzyme A synthetase ACSM2A, mitochondrial	ACSM2A	Homo sapiens	64.224	2	2	0	77336
7483	1	Q0PNE2	Q0PNE2	Elongator complex protein 6	ELP6	Homo sapiens	29.792	2	2	274572	28239
7484	1	Q10589	Q10589	Bone marrow stromal antigen 2	BST2	Homo sapiens	19.77	2	2	3364000	1630684
7485	1	Q12840	Q12840	Kinesin heavy chain isoform 5A	KIF5A	Homo sapiens	117.378	2	2	145079	0
7486	1	Q12866	Q12866	Tyrosine-protein kinase Mer	MERTK	Homo sapiens	110.252	2	2	113071	0
7487	1	Q12980	Q12980	GATOR complex protein NPRL3	NPRL3	Homo sapiens	63.606	2	2	260244	0
7488	1	Q13093	Q13093	Platelet-activating factor acetylhydrolase	PLA2G7	Homo sapiens	50.073	2	2	30335	127067
7489	1	Q13113	Q13113	PDZK1-interacting protein 1	PDZK1IP1	Homo sapiens	12.225	2	2	0	206690
7490	1	Q13158	Q13158	FAS-associated death domain protein	FADD	Homo sapiens	23.281	2	2	81786	265561
7491	1	Q13183	Q13183	Solute carrier family 13 member 2	SLC13A2	Homo sapiens	64.411	2	2	136432	0
7492	1	Q13239	Q13239	Src-like-adapter	SLA	Homo sapiens	31.157	2	2	0	139762
7493	1	Q13247	Q13247	Serine/arginine-rich splicing factor 6	SRSF6	Homo sapiens	39.589	2	2	913342	73947
7494	1	Q13257	Q13257	Mitotic spindle assembly checkpoint protein MAD2A	MAD2L1	Homo sapiens	23.508	2	2	355752	376944
7495	1	Q13336	Q13336	Urea transporter 1	SLC14A1	Homo sapiens	42.528	2	2	111476	291638
7496	1	Q13454	Q13454	Tumor suppressor candidate 3	TUSC3	Homo sapiens	39.674	2	2	251795	0
7497	1	Q13472	Q13472	DNA topoisomerase 3-alpha	TOP3A	Homo sapiens	112.375	2	2	5275	12848
7498	1	Q13503	Q13503	Mediator of RNA polymerase II transcription subunit 21	MED21	Homo sapiens	15.565	2	2	76244	36243
7499	1	Q13541	Q13541	Eukaryotic translation initiation factor 4E-binding protein 1	EIF4EBP1	Homo sapiens	12.581	2	2	552914	75617
7500	1	Q13641	Q13641	Trophoblast glycoprotein	TPBG	Homo sapiens	46.031	2	2	81596	0
7501	1	Q13772	Q13772	Nuclear receptor coactivator 4	NCOA4	Homo sapiens	69.725	2	2	0	146649
7502	1	Q14002	Q14002	Carcinoembryonic antigen-related cell adhesion molecule 7	CEACAM7	Homo sapiens	29.346	2	2	4845640	126421
7503	1	Q14061	Q14061	Cytochrome c oxidase copper chaperone	COX17	Homo sapiens	6.912	2	2	496071	159487
7504	1	Q14088	Q14088	Ras-related protein Rab-33A	RAB33A	Homo sapiens	26.591	2	2	0	119702
7505	1	Q14117	Q14117	Dihydropyrimidinase	DPYS	Homo sapiens	56.629	2	2	61249	464098
7506	1	Q14135	Q14135	Transcription cofactor vestigial-like protein 4	VGLL4	Homo sapiens	30.948	2	2	98212	0
7507	1	Q14162	Q14162	Scavenger receptor class F member 1	SCARF1	Homo sapiens	87.388	2	2	123739	73976
7508	1	Q14508	Q14508	WAP four-disulfide core domain protein 2	WFDC2	Homo sapiens	12.995	2	2	1196963	81938
7509	1	Q14511	Q14511	Enhancer of filamentation 1	NEDD9	Homo sapiens	92.862	2	2	36344	172828
7510	1	Q14526	Q14526	Hypermethylated in cancer 1 protein	HIC1	Homo sapiens	76.509	2	2	75052	0
7511	1	Q14686	Q14686	Nuclear receptor coactivator 6	NCOA6	Homo sapiens	219.149	2	2	130438	0
7512	1	Q14714	Q14714	Sarcospan	SSPN	Homo sapiens	26.615	2	2	132523	0
7513	1	Q14849	Q14849	StAR-related lipid transfer protein 3	STARD3	Homo sapiens	50.501	2	2	0	213137
7514	1	Q14872	Q14872	Metal regulatory transcription factor 1	MTF1	Homo sapiens	80.956	2	2	46732	70671
7515	1	Q149N8	Q149N8	E3 ubiquitin-protein ligase SHPRH	SHPRH	Homo sapiens	193.083	2	2	29520	0
7516	1	Q14CS0	Q14CS0	UBX domain-containing protein 2B	UBXN2B	Homo sapiens	37.077	2	2	61589	42304

7517	1	Q14CZ8	Q14CZ8	Hepatocyte cell adhesion molecule	HEPACAM	Homo sapiens	46.027	2	2	94231	0
7518	1	Q15013	Q15013	MAD2L1-binding protein	MAD2L1BP	Homo sapiens	31.051	2	2	44194	88813
7519	1	Q15014	Q15014	Mortality factor 4-like protein 2	MORF4L2	Homo sapiens	32.307	2	2	193592	0
7520	1	Q15034	Q15034	Probable E3 ubiquitin-protein ligase HERC3	HERC3	Homo sapiens	117.19	2	2	0	144785
7521	1	Q15040	Q15040	Josephin-1	JOSD1	Homo sapiens	23.199	2	2	76506	0
7522	1	Q15125	Q15125	3-beta-hydroxysteroid-Delta(8),Delta(7)-isomerase	EBP	Homo sapiens	26.354	2	2	3731524	1100384
7523	1	Q15155	Q15155	BOS complex subunit NOMO1	NOMO1	Homo sapiens	134.323	28	2	558384	551699
7524	1	Q15388	Q15388	Mitochondrial import receptor subunit TOM20 homolog	TOMM20	Homo sapiens	16.295	2	2	461745	270238
7525	1	Q15390	Q15390	Mitochondrial fission regulator 1	MTFR1	Homo sapiens	37.001	2	2	128505	0
7526	1	Q15427	Q15427	Splicing factor 3B subunit 4	SF3B4	Homo sapiens	44.386	2	2	1710417	134624
7527	1	Q15434	Q15434	RNA-binding motif, single-stranded-interacting protein 2	RBMS2	Homo sapiens	43.957	2	2	378823	458173
7528	1	Q15464	Q15464	SH2 domain-containing adapter protein B	SHB	Homo sapiens	55.041	2	2	105828	0
7529	1	Q15714	Q15714	TSC22 domain family protein 1	TSC22D1	Homo sapiens	109.678	2	2	144741	0
7530	1	Q15744	Q15744	CCAAT/enhancer-binding protein epsilon	CEBPE	Homo sapiens	30.602	2	2	0	237867
7531	1	Q15797	Q15797	Mothers against decapentaplegic homolog 1	SMAD1	Homo sapiens	52.259	7	2	182605	0
7532	1	Q15836	Q15836	Vesicle-associated membrane protein 3	VAMP3	Homo sapiens	11.307	4	2	777820	554504
7533	1	Q15843	Q15843	NEDD8	NEDD8	Homo sapiens	9.068	2	2	3815992	2673390
7534	1	Q15848	Q15848	Adiponectin	ADIPOQ	Homo sapiens	26.412	2	2	750609	360762
7535	1	Q15907	Q15907	Ras-related protein Rab-11B	RAB11B	Homo sapiens	24.487	13	2	3000835	1167840
7536	1	Q16394	Q16394	Exostosin-1	EXT1	Homo sapiens	86.258	2	2	26114	39474
7537	1	Q16581	Q16581	C3a anaphylatoxin chemotactic receptor	C3AR1	Homo sapiens	53.863	2	2	0	301010
7538	1	Q16621	Q16621	Transcription factor NF-E2 45 kDa subunit	NFE2	Homo sapiens	41.474	2	2	0	163532
7539	1	Q16626	Q16626	Male-enhanced antigen 1	MEA1	Homo sapiens	19.904	2	2	215847	154537
7540	1	Q16637	Q16637	Survival motor neuron protein	SMN2	Homo sapiens	31.847	2	2	447284	230043
7541	1	Q16649	Q16649	Nuclear factor interleukin-3-regulated protein	NFIL3	Homo sapiens	51.471	2	2	43044	27568
7542	1	Q16739	Q16739	Ceramide glucosyltransferase	UGCG	Homo sapiens	44.853	2	2	0	188730
7543	1	Q17RY0	Q17RY0	Cytoplasmic polyadenylation element-binding protein 4	CPEB4	Homo sapiens	80.152	4	2	52975	83152
7544	1	Q19T08	Q19T08	Endothelial cell-specific chemotaxis regulator	ECSCR	Homo sapiens	21.293	2	2	118999	0
7545	1	Q1ED39	Q1ED39	Lysine-rich nucleolar protein 1	KNOP1	Homo sapiens	51.586	2	2	84836	0
7546	1	Q2KHR3	Q2KHR3	Glutamine and serine-rich protein 1	QSER1	Homo sapiens	189.974	2	2	58584	0
7547	1	Q2M2Z5	Q2M2Z5	Centrosomal protein kizuna	KIZ	Homo sapiens	75.112	2	2	867849	0
7548	1	Q2M3G4	Q2M3G4	Protein Shroom1	SHROOM1	Homo sapiens	90.787	2	2	193490	14098
7549	1	Q2T9J0	Q2T9J0	Peroxisomal leader peptide-processing protease	TYSND1	Homo sapiens	59.312	2	2	27941	19270
7550	1	Q2TB10	Q2TB10	Zinc finger protein 800	ZNF800	Homo sapiens	75.237	2	2	94359	0
7551	1	Q30201	Q30201	Hereditary hemochromatosis protein	HFE	Homo sapiens	40.108	2	2	188898	0
7552	1	Q330K2	Q330K2	NADH dehydrogenase (ubiquinone) complex I, assembly factor 6	NDUFAF6	Homo sapiens	38.177	2	2	113321	19007
7553	1	Q3B7J2	Q3B7J2	Glucose-fructose oxidoreductase domain-containing protein 2	GFOD2	Homo sapiens	42.255	2	2	204076	0
7554	2	Q3B8N2;Q6DKI2	Q3B8N2	Galectin-9B	LGALS9B	Homo sapiens	39.661	2	2	422715	0
7555	1	Q3KP66	Q3KP66	Innate immunity activator protein	INAVA	Homo sapiens	72.915	2	2	91289	0
7556	1	Q3MHD2	Q3MHD2	Protein LSM12	LSM12	Homo sapiens	21.7	2	2	489242	240708
7557	1	Q3MIP1	Q3MIP1	Inositol 1,4,5-trisphosphate receptor-interacting protein-like 2	ITPRIPL2	Homo sapiens	58.446	2	2	46819	0
7558	1	Q3ZCM7	Q3ZCM7	Tubulin beta-8 chain	TUBB8	Homo sapiens	49.774	3	2	111549	0
7559	1	Q495W5	Q495W5	Alpha-(1,3)-fucosyltransferase 11	FUT11	Homo sapiens	55.816	2	2	142357	24998
7560	1	Q496J9	Q496J9	Synaptic vesicle glycoprotein 2C	SV2C	Homo sapiens	82.344	3	2	72476	0
7561	1	Q49AR2	Q49AR2	UPF0489 protein C5orf22	C5orf22	Homo sapiens	49.968	2	2	24507	44065
7562	1	Q4ADV7	Q4ADV7	Guanine nucleotide exchange factor subunit RIC1	RIC1	Homo sapiens	159.3	2	2	31995	29539
7563	1	Q4U2R6	Q4U2R6	39S ribosomal protein L51, mitochondrial	MRPL51	Homo sapiens	15.093	2	2	128565	0
7564	1	Q4VCS5	Q4VCS5	Angiomotin	AMOT	Homo sapiens	118.085	2	2	39281	0

7565	1	Q4ZIN3	Q4ZIN3	Membralin	TMEM259	Homo sapiens	67.888	2	2	441500	74088
7566	1	Q53G44	Q53G44	Interferon-induced protein 44-like	IFI44L	Homo sapiens	51.323	2	2	38202	80163
7567	1	Q53RD9	Q53RD9	Fibulin-7	FBLN7	Homo sapiens	47.377	2	2	50558	20447
7568	1	Q53RY4	Q53RY4	Keratinocyte-associated protein 3	KRTCAP3	Homo sapiens	25.628	2	2	223961	0
7569	1	Q53S58	Q53S58	Transmembrane protein 177	TMEM177	Homo sapiens	33.762	2	2	292877	0
7570	1	Q58FG1	Q58FG1	Putative heat shock protein HSP 90-alpha A4	HSP90AA4P	Homo sapiens	47.712	2	2	170307	1592910
7571	1	Q5BJD5	Q5BJD5	Transmembrane protein 41B	TMEM41B	Homo sapiens	32.513	2	2	468548	99465
7572	1	Q5BJF2	Q5BJF2	Sigma intracellular receptor 2	TMEM97	Homo sapiens	20.847	2	2	922977	296942
7573	1	Q5BKX5	Q5BKX5	Actin maturation protease	ACTMAP	Homo sapiens	37.777	2	2	185807	98510
7574	1	Q5D1E8	Q5D1E8	Endoribonuclease ZC3H12A	ZC3H12A	Homo sapiens	65.701	3	2	39713	0
7575	1	Q5GH76	Q5GH76	XK-related protein 4	XKR4	Homo sapiens	71.5	2	2	237228	0
7576	1	Q5JPI3	Q5JPI3	Uncharacterized protein C3orf38	C3orf38	Homo sapiens	37.541	2	2	165261	123011
7577	1	Q5JPI9	Q5JPI9	EEF1A lysine methyltransferase 2	EEF1AKMT2	Homo sapiens	31.828	2	2	216033	147530
7578	1	Q5JTW2	Q5JTW2	Centrosomal protein of 78 kDa	CEP78	Homo sapiens	76.396	2	2	33838	0
7579	1	Q5M7Z0	Q5M7Z0	E3 ubiquitin-protein ligase RNFT1	RNFT1	Homo sapiens	49.709	2	2	146246	186004
7580	1	Q5M8T2	Q5M8T2	Solute carrier family 35 member D3	SLC35D3	Homo sapiens	44.184	2	2	73737	0
7581	1	Q5QP82	Q5QP82	DDB1- and CUL4-associated factor 10	DCAF10	Homo sapiens	60.58	2	2	51626	0
7582	1	Q5R3K3	Q5R3K3	Calcium homeostasis modulator protein 6	CALHM6	Homo sapiens	34.459	2	2	0	219465
7583	1	Q5SQI0	Q5SQI0	Alpha-tubulin N-acetyltransferase 1	ATAT1	Homo sapiens	46.813	2	2	114419	261619
7584	1	Q5SVZ6	Q5SVZ6	Zinc finger MYM-type protein 1	ZMYM1	Homo sapiens	128.719	2	2	26012	0
7585	1	Q5SXH7	Q5SXH7	Pleckstrin homology domain-containing family S member 1	PLEKHS1	Homo sapiens	52.462	2	2	0	77370
7586	1	Q5SXM8	Q5SXM8	DNL-type zinc finger protein	DNLZ	Homo sapiens	19.204	2	2	490495	0
7587	1	Q5T280	Q5T280	Putative methyltransferase C9orf114	SPOUT1	Homo sapiens	42.009	2	2	66302	0
7588	1	Q5T3F8	Q5T3F8	CSC1-like protein 2	TMEM63B	Homo sapiens	94.958	2	2	66010	0
7589	1	Q5T4F4	Q5T4F4	Protrudin	ZFYVE27	Homo sapiens	45.845	2	2	94439	65526
7590	1	Q5T8I3	Q5T8I3	EEIG family member 2	EEIG2	Homo sapiens	39.309	2	2	153465	0
7591	1	Q5TAA0	Q5TAA0	Tetratricopeptide repeat protein 22	TTC22	Homo sapiens	63.361	2	2	87863	0
7592	1	Q5TBC7	Q5TBC7	Bcl-2-like protein 15	BCL2L15	Homo sapiens	17.724	2	2	61329	905526
7593	1	Q5TKA1	Q5TKA1	Protein lin-9 homolog	LIN9	Homo sapiens	61.946	2	2	141140	0
7594	1	Q5U5X0	Q5U5X0	Complex III assembly factor LYRM7	LYRM7	Homo sapiens	11.952	2	2	527872	100227
7595	1	Q5U623	Q5U623	Activating transcription factor 7-interacting protein 2	ATF7IP2	Homo sapiens	75.764	2	2	126874	0
7596	1	Q5UCC4	Q5UCC4	ER membrane protein complex subunit 10	EMC10	Homo sapiens	27.347	2	2	297496	181521
7597	1	Q5VST6	Q5VST6	Alpha/beta hydrolase domain-containing protein 17B	ABHD17B	Homo sapiens	32.216	2	2	92481	176672
7598	1	Q5VSY0	Q5VSY0	G kinase-anchoring protein 1	GKAP1	Homo sapiens	42.08	2	2	90352	0
7599	1	Q5VTB9	Q5VTB9	E3 ubiquitin-protein ligase RNF220	RNF220	Homo sapiens	62.766	2	2	170356	73182
7600	1	Q5VWP2	Q5VWP2	Terminal nucleotidyltransferase 5C	TENT5C	Homo sapiens	44.944	2	2	37182	0
7601	1	Q5VYX0	Q5VYX0	Renalase	RNLS	Homo sapiens	37.845	2	2	163059	0
7602	1	Q5W0Z9	Q5W0Z9	Palmitoyltransferase ZDHHC20	ZDHHC20	Homo sapiens	42.278	2	2	138026	709652
7603	1	Q5XKK7	Q5XKK7	Protein FAM219B	FAM219B	Homo sapiens	21.102	2	2	100946	0
7604	1	Q5ZPR3	Q5ZPR3	CD276 antigen	CD276	Homo sapiens	57.238	2	2	407820	67534
7605	1	Q641Q2	Q641Q2	WASH complex subunit 2A	WASHC2A	Homo sapiens	147.186	2	2	170997	170324
7606	1	Q66LE6	Q66LE6	Serine/threonine-protein phosphatase 2A 55 kDa regulatory subunit B delta isoform	PPP2R2D	Homo sapiens	52.043	2	2	395614	347547
7607	1	Q69YL0	Q69YL0	Protein NCBP2AS2	NCBP2AS2	Homo sapiens	10.887	2	2	221481	0
7608	1	Q6AI39	Q6AI39	BRD4-interacting chromatin-remodeling complex-associated protein-like	BICRAL	Homo sapiens	115.085	2	2	221314	0
7609	1	Q6BDI9	Q6BDI9	Rab15 effector protein	REP15	Homo sapiens	26.57	2	2	406347	0
7610	1	Q6DN12	Q6DN12	Multiple C2 and transmembrane domain-containing protein 2	MCTP2	Homo sapiens	99.597	2	2	52107	139074
7611	1	Q6FIF0	Q6FIF0	AN1-type zinc finger protein 6	ZFAND6	Homo sapiens	22.558	2	2	202089	106486

7612	1	Q6IN84	Q6IN84	rRNA methyltransferase 1, mitochondrial	MRM1	Homo sapiens	38.637	2	2	84248	0
7613	1	Q6IPR1	Q6IPR1	Electron transfer flavoprotein regulatory factor 1	ETFRF1	Homo sapiens	10.861	2	2	122423	15801
7614	1	Q6IQ32	Q6IQ32	Activity-dependent neuroprotector homeobox protein 2	ADNP2	Homo sapiens	122.835	2	2	42698	0
7615	1	Q6IWH7	Q6IWH7	Anoctamin-7	ANO7	Homo sapiens	105.533	2	2	202144	0
7616	1	Q6KCM7	Q6KCM7	Calcium-binding mitochondrial carrier protein SCaMC-2	SLC25A25	Homo sapiens	52.663	2	2	94671	19261
7617	1	Q6MZP7	Q6MZP7	Protein lin-54 homolog	LIN54	Homo sapiens	79.496	2	2	62130	22715
7618	1	Q6MZQ0	Q6MZQ0	Proline-rich protein 5-like	PRR5L	Homo sapiens	40.835	2	2	70393	0
7619	1	Q6N075	Q6N075	Molybdate-anion transporter	MFSD5	Homo sapiens	49.766	2	2	131545	105836
7620	1	Q6NV74	Q6NV74	CRACD-like protein	CRACDL	Homo sapiens	102.16	2	2	86997	0
7621	1	Q6NXR4	Q6NXR4	TELO2-interacting protein 2	TTI2	Homo sapiens	56.917	2	2	40186	12116
7622	1	Q6NXT4	Q6NXT4	Zinc transporter 6	SLC30A6	Homo sapiens	51.115	2	2	312863	105186
7623	1	Q6P1L5	Q6P1L5	Protein FAM117B	FAM117B	Homo sapiens	61.968	2	2	178679	0
7624	1	Q6P2P2	Q6P2P2	Protein arginine N-methyltransferase 9	PRMT9	Homo sapiens	94.502	2	2	124535	0
7625	1	Q6P531	Q6P531	Glutathione hydrolase 6	GGT6	Homo sapiens	50.51	2	2	72147	0
7626	1	Q6P6B7	Q6P6B7	Ankyrin repeat domain-containing protein 16	ANKRD16	Homo sapiens	39.285	2	2	51206	0
7627	1	Q6PCB8	Q6PCB8	Embigin	EMB	Homo sapiens	36.88	2	2	40702	296768
7628	1	Q6PD74	Q6PD74	Alpha- and gamma-adaptin-binding protein p34	AAGAB	Homo sapiens	34.594	2	2	636341	123189
7629	1	Q6PIJ6	Q6PIJ6	F-box only protein 38	FBXO38	Homo sapiens	133.945	2	2	191445	111863
7630	1	Q6QNY0	Q6QNY0	Biogenesis of lysosome-related organelles complex 1 subunit 3	BLOC1S3	Homo sapiens	21.255	2	2	557938	0
7631	1	Q6UVJ0	Q6UVJ0	Spindle assembly abnormal protein 6 homolog	SASS6	Homo sapiens	74.397	2	2	17563	34969
7632	1	Q6UWD8	Q6UWD8	Transmembrane protein C16orf54	C16orf54	Homo sapiens	24.359	2	2	0	101013
7633	1	Q6UWK7	Q6UWK7	Protein GPR15LG	GPR15LG	Homo sapiens	9.17	2	2	89916	73109
7634	1	Q6UWR7	Q6UWR7	Glycerophosphocholine cholinephosphodiesterase ENPP6	ENPP6	Homo sapiens	50.238	2	2	146106	0
7635	1	Q6UWZ7	Q6UWZ7	BRCA1-A complex subunit Abraxas 1	ABRAXAS1	Homo sapiens	46.662	2	2	118947	68486
7636	1	Q6UX15	Q6UX15	Layilin	LAYN	Homo sapiens	43.108	2	2	196714	0
7637	1	Q6UXE8	Q6UXE8	Butyrophilin-like protein 3	BTNL3	Homo sapiens	52.25	2	2	207276	0
7638	1	Q6UXH9	Q6UXH9	Inactive serine protease PAMR1	PAMR1	Homo sapiens	80.199	2	2	74305	34754
7639	1	Q6UXP7	Q6UXP7	Protein FAM151B	FAM151B	Homo sapiens	31.365	2	2	23268	33503
7640	1	Q6XE24	Q6XE24	RNA-binding motif, single-stranded-interacting protein 3	RBMS3	Homo sapiens	47.839	2	2	397382	0
7641	1	Q6XUX3	Q6XUX3	Dual serine/threonine and tyrosine protein kinase	DSTYK	Homo sapiens	105.207	2	2	11364	50969
7642	1	Q6ZMD2	Q6ZMD2	Protein spinster homolog 3	SPNS3	Homo sapiens	54.769	2	2	0	120519
7643	1	Q6ZMW3	Q6ZMW3	Echinoderm microtubule-associated protein-like 6	EML6	Homo sapiens	217.901	2	2	35726	158192
7644	1	Q6ZNC8	Q6ZNC8	Lysophospholipid acyltransferase 1	MBOAT1	Homo sapiens	56.557	2	2	227165	0
7645	1	Q6ZPD9	Q6ZPD9	Probable C-mannosyltransferase DPY19L3	DPY19L3	Homo sapiens	83.199	2	2	215689	250038
7646	1	Q6ZSC3	Q6ZSC3	RNA-binding protein 43	RBM43	Homo sapiens	40.665	2	2	92345	0
7647	1	Q6ZSZ5	Q6ZSZ5	Rho guanine nucleotide exchange factor 18	ARHGEF18	Homo sapiens	151.643	16	2	48783	119815
7648	1	Q6ZU80	Q6ZU80	Centrosomal protein of 128 kDa	CEP128	Homo sapiens	128.016	2	2	0	87473
7649	1	Q6ZUS6	Q6ZUS6	Coiled-coil domain-containing protein 149	CCDC149	Homo sapiens	52.796	2	2	42529	0
7650	1	Q6ZUX7	Q6ZUX7	LHFPL tetraspan subfamily member 2 protein	LHFPL2	Homo sapiens	24.483	2	2	536008	240174
7651	1	Q6ZVD8	Q6ZVD8	PH domain leucine-rich repeat-containing protein phosphatase 2	PHLPP2	Homo sapiens	146.753	2	2	52842	0
7652	1	Q70HW3	Q70HW3	S-adenosylmethionine mitochondrial carrier protein	SLC25A26	Homo sapiens	29.35	2	2	77876	0
7653	1	Q70IA8	Q70IA8	MOB kinase activator 3C	MOB3C	Homo sapiens	25.623	2	2	269937	0
7654	1	Q717R9	Q717R9	Cystin-1	CYS1	Homo sapiens	16.39	2	2	72608	0
7655	1	Q71F56	Q71F56	Mediator of RNA polymerase II transcription subunit 13-like	MED13L	Homo sapiens	242.606	2	2	147021	0
7656	1	Q71SY5	Q71SY5	Mediator of RNA polymerase II transcription subunit 25	MED25	Homo sapiens	78.17	3	2	234776	74027
7657	1	Q75N03	Q75N03	E3 ubiquitin-protein ligase Hakai	CBLL1	Homo sapiens	54.518	2	2	198449	50360
7658	1	Q76EJ3	Q76EJ3	UDP-N-acetylglucosamine/UDP-glucose/GDP-mannose transporter	SLC35D2	Homo sapiens	36.673	2	2	77783	0
7659	1	Q76L83	Q76L83	Putative Polycomb group protein ASXL2	ASXL2	Homo sapiens	153.822	2	2	17592	0

7660	1	Q7L1V2	Q7L1V2	Vacuolar fusion protein MON1 homolog B	MON1B	Homo sapiens	59.219	2	2	30635	67652
7661	1	Q7L211	Q7L211	Protein ABHD13	ABHD13	Homo sapiens	38.548	2	2	47005	89410
7662	1	Q7L2Z9	Q7L2Z9	Centromere protein Q	CENPQ	Homo sapiens	30.593	2	2	0	134245
7663	1	Q7L3B6	Q7L3B6	Hsp90 co-chaperone Cdc37-like 1	CDC37L1	Homo sapiens	38.832	2	2	90257	75500
7664	1	Q7L804	Q7L804	Rab11 family-interacting protein 2	RAB11FIP2	Homo sapiens	58.28	2	2	71021	0
7665	1	Q7LFX5	Q7LFX5	Carbohydrate sulfotransferase 15	CHST15	Homo sapiens	64.925	2	2	68403	0
7666	1	Q7RTS1	Q7RTS1	Class A basic helix-loop-helix protein 15	BHLHA15	Homo sapiens	20.818	2	2	118022	0
7667	1	Q7Z2E3	Q7Z2E3	Aprataxin	APTX	Homo sapiens	40.738	2	2	587101	243733
7668	1	Q7Z2H8	Q7Z2H8	Proton-coupled amino acid transporter 1	SLC36A1	Homo sapiens	53.074	2	2	63769	36262
7669	1	Q7Z2X4	Q7Z2X4	PTB-containing, cubilin and LRP1-interacting protein	PID1	Homo sapiens	28.271	2	2	277339	0
7670	1	Q7Z388	Q7Z388	Probable C-mannosyltransferase DPY19L4	DPY19L4	Homo sapiens	83.757	2	2	96414	0
7671	1	Q7Z402	Q7Z402	Transmembrane channel-like protein 7	TMC7	Homo sapiens	83.501	2	2	28222	25882
7672	1	Q7Z4F1	Q7Z4F1	Low-density lipoprotein receptor-related protein 10	LRP10	Homo sapiens	76.194	2	2	121743	113458
7673	1	Q7Z4G1	Q7Z4G1	COMM domain-containing protein 6	COMMD6	Homo sapiens	9.637	2	2	34184	64208
7674	1	Q7Z5L2	Q7Z5L2	Coiled-coil domain-containing protein R3HCC1L	R3HCC1L	Homo sapiens	87.883	2	2	37395	0
7675	1	Q7Z6L0	Q7Z6L0	Proline-rich transmembrane protein 2	PRRT2	Homo sapiens	34.946	2	2	48782	0
7676	1	Q7Z6M1	Q7Z6M1	Rab9 effector protein with kelch motifs	RABEPK	Homo sapiens	40.566	2	2	336258	57627
7677	1	Q7Z6M4	Q7Z6M4	Transcription termination factor 4, mitochondrial	MTERF4	Homo sapiens	43.959	2	2	159723	0
7678	1	Q7Z7A3	Q7Z7A3	Cytoplasmic tRNA 2-thiolation protein 1	CTU1	Homo sapiens	36.45	2	2	142935	0
7679	1	Q7Z7C8	Q7Z7C8	Transcription initiation factor TFIID subunit 8	TAF8	Homo sapiens	34.262	2	2	119285	0
7680	1	Q7Z7E8	Q7Z7E8	Ubiquitin-conjugating enzyme E2 Q1	UBE2Q1	Homo sapiens	46.127	3	2	143908	249050
7681	1	Q7Z7F0	Q7Z7F0	KH homology domain-containing protein 4	KHDC4	Homo sapiens	64.844	2	2	121082	0
7682	1	Q7Z7L8	Q7Z7L8	Uncharacterized protein C11orf96	C11orf96	Homo sapiens	46.114	2	2	191073	55334
7683	1	Q86SK9	Q86SK9	Stearoyl-CoA desaturase 5	SCD5	Homo sapiens	37.607	2	2	465781	0
7684	1	Q86SU0	Q86SU0	Immunoglobulin-like domain-containing receptor 1	ILDR1	Homo sapiens	62.814	2	2	34083	5931443
7685	1	Q86T24	Q86T24	Transcriptional regulator Kaiso	ZBTB33	Homo sapiens	74.483	2	2	51660	0
7686	1	Q86TM6	Q86TM6	E3 ubiquitin-protein ligase synoviolin	SYVN1	Homo sapiens	67.684	2	2	287115	218183
7687	1	Q86U06	Q86U06	Probable RNA-binding protein 23	RBM23	Homo sapiens	48.731	2	2	69568	29599
7688	1	Q86U70	Q86U70	LIM domain-binding protein 1	LDB1	Homo sapiens	46.532	3	2	91696	0
7689	1	Q86UD1	Q86UD1	Out at first protein homolog	OAF	Homo sapiens	30.688	2	2	374688	727419
7690	1	Q86UU0	Q86UU0	B-cell CLL/lymphoma 9-like protein	BCL9L	Homo sapiens	157.131	2	2	43634	28849
7691	1	Q86V85	Q86V85	Integral membrane protein GPR180	GPR180	Homo sapiens	49.395	2	2	862219	182344
7692	1	Q86VE9	Q86VE9	Serine incorporator 5	SERINC5	Homo sapiens	47.01	2	2	219334	51495
7693	1	Q86VQ6	Q86VQ6	Thioredoxin reductase 3	TXNRD3	Homo sapiens	70.654	2	2	84581	0
7694	1	Q86VY9	Q86VY9	Transmembrane protein 200A	TMEM200A	Homo sapiens	54.356	2	2	94982	0
7695	1	Q86WA6	Q86WA6	Valacyclovir hydrolase	BPHL	Homo sapiens	32.542	2	2	1232961	0
7696	1	Q86WA8	Q86WA8	Lon protease homolog 2, peroxisomal	LONP2	Homo sapiens	94.62	2	2	134925	30275
7697	1	Q86WX3	Q86WX3	Active regulator of SIRT1	RPS19BP1	Homo sapiens	15.431	2	2	207190	0
7698	1	Q86XN7	Q86XN7	Proline and serine-rich protein 1	PROSER1	Homo sapiens	95.699	2	2	36149	33090
7699	1	Q86XR8	Q86XR8	Centrosomal protein of 57 kDa	CEP57	Homo sapiens	57.09	2	2	97616	0
7700	1	Q86Y79	Q86Y79	Probable peptidyl-tRNA hydrolase	PTRH1	Homo sapiens	22.937	2	2	155199	0
7701	1	Q86YB8	Q86YB8	ERO1-like protein beta	ERO1B	Homo sapiens	53.543	2	2	93143	21250
7702	1	Q8IU68	Q8IU68	Transmembrane channel-like protein 8	TMC8	Homo sapiens	81.641	2	2	0	562272
7703	1	Q8IUA7	Q8IUA7	ATP-binding cassette sub-family A member 9	ABCA9	Homo sapiens	184.363	2	2	30586	0
7704	1	Q8IUD6	Q8IUD6	E3 ubiquitin-protein ligase RNF135	RNF135	Homo sapiens	47.888	2	2	119536	101017
7705	2	Q8IUF1;Q9BRT8	Q8IUF1	Zinc-regulated GTPase metalloprotein activator 1B	ZNG1B	Homo sapiens	44.032	7	2	122521	96983
7706	1	Q8IUW5	Q8IUW5	RELT-like protein 1	RELL1	Homo sapiens	29.341	2	2	45341	18804
7707	1	Q8IV20	Q8IV20	Purine nucleoside phosphorylase LACC1	LACC1	Homo sapiens	47.778	2	2	94197	12922

7708	1	Q8IV53	Q8IV53	DENN domain-containing protein 1C	DENND1C	Homo sapiens	87.066	2	2	148231	437268
7709	1	Q8IV56	Q8IV56	Proline-rich protein 15	PRR15	Homo sapiens	13.712	2	2	283863	0
7710	1	Q8IVH2	Q8IVH2	Forkhead box protein P4	FOXP4	Homo sapiens	73.488	2	2	220655	0
7711	1	Q8IVL1	Q8IVL1	Neuron navigator 2	NAV2	Homo sapiens	268.172	2	2	62226	0
7712	1	Q8IVL5	Q8IVL5	Prolyl 3-hydroxylase 2	P3H2	Homo sapiens	80.985	2	2	0	142567
7713	1	Q8IW19	Q8IW19	Aprataxin and PNK-like factor	APLF	Homo sapiens	56.959	2	2	36918	0
7714	1	Q8IW41	Q8IW41	MAP kinase-activated protein kinase 5	MAPKAPK5	Homo sapiens	54.221	2	2	303167	67198
7715	1	Q8IWT0	Q8IWT0	Protein archease	ZBTB8OS	Homo sapiens	19.492	2	2	145972	313005
7716	1	Q8IWV2	Q8IWV2	Contactin-4	CNTN4	Homo sapiens	113.453	3	2	63330	0
7717	1	Q8IX03	Q8IX03	Protein KIBRA	WWC1	Homo sapiens	125.302	2	2	0	48701
7718	1	Q8IXL6	Q8IXL6	Extracellular serine/threonine protein kinase FAM20C	FAM20C	Homo sapiens	66.236	2	2	82376	262166
7719	1	Q8IY26	Q8IY26	Polyisoprenoid diphosphate/phosphate phosphohydrolase PLPP6	PLPP6	Homo sapiens	32.196	2	2	99016	0
7720	1	Q8IY34	Q8IY34	Solute carrier family 15 member 3	SLC15A3	Homo sapiens	63.558	2	2	609537	45870
7721	1	Q8IY47	Q8IY47	Kelch repeat and BTB domain-containing protein 2	KBTBD2	Homo sapiens	71.331	2	2	68661	0
7722	1	Q8IYD1	Q8IYD1	Eukaryotic peptide chain release factor GTP-binding subunit ERF3B	GSPT2	Homo sapiens	68.885	2	2	146572	0
7723	1	Q8IYH5	Q8IYH5	ZZ-type zinc finger-containing protein 3	ZZZ3	Homo sapiens	102.025	2	2	55797	0
7724	1	Q8IZM8	Q8IZM8	Zinc finger protein 654	ZNF654	Homo sapiens	127.879	2	2	40516	0
7725	1	Q8N0V3	Q8N0V3	Putative ribosome-binding factor A, mitochondrial	RBFA	Homo sapiens	38.358	2	2	70646	0
7726	1	Q8N111	Q8N111	Cell cycle exit and neuronal differentiation protein 1	CEND1	Homo sapiens	14.953	2	2	727932	0
7727	1	Q8N131	Q8N131	Porimin	TMEM123	Homo sapiens	21.532	2	2	113849	343805
7728	1	Q8N142	Q8N142	Adenylosuccinate synthetase isozyme 1	ADSS1	Homo sapiens	50.209	2	2	0	97479
7729	1	Q8N1Q1	Q8N1Q1	Carbonic anhydrase 13	CA13	Homo sapiens	29.441	2	2	88062	32451
7730	1	Q8N271	Q8N271	Prominin-2	PROM2	Homo sapiens	91.883	2	2	88937	0
7731	1	Q8N2H3	Q8N2H3	Pyridine nucleotide-disulfide oxidoreductase domain-containing protein 2	PYROXD2	Homo sapiens	63.069	2	2	30013	0
7732	1	Q8N357	Q8N357	Solute carrier family 35 member F6	SLC35F6	Homo sapiens	40.214	2	2	487056	80090
7733	1	Q8N386	Q8N386	Leucine-rich repeat-containing protein 25	LRRC25	Homo sapiens	33.179	2	2	94105	349641
7734	1	Q8N394	Q8N394	Protein O-mannosyl-transferase TMTC2	TMTC2	Homo sapiens	94.131	2	2	58964	0
7735	1	Q8N3F0	Q8N3F0	Maturin	MTURN	Homo sapiens	14.925	2	2	52125	0
7736	1	Q8N3Z3	Q8N3Z3	GTP-binding protein 8	GTPBP8	Homo sapiens	32.144	2	2	64693	68203
7737	1	Q8N468	Q8N468	Major facilitator superfamily domain-containing protein 4A	MFSD4A	Homo sapiens	56.264	2	2	294641	0
7738	1	Q8N4J0	Q8N4J0	Carnosine N-methyltransferase	CARNMT1	Homo sapiens	47.186	2	2	182927	0
7739	1	Q8N4Y2	Q8N4Y2	EF-hand calcium-binding domain-containing protein 4A	CRACR2B	Homo sapiens	44.957	2	2	64016	0
7740	1	Q8N531	Q8N531	F-box/LRR-repeat protein 6	FBXL6	Homo sapiens	58.56	2	2	109197	0
7741	1	Q8N554	Q8N554	Zinc finger protein 276	ZNF276	Homo sapiens	67.219	2	2	70035	18645
7742	1	Q8N5C1	Q8N5C1	Calcium homeostasis modulator protein 5	CALHM5	Homo sapiens	35.169	2	2	667381	46264
7743	1	Q8N5C7	Q8N5C7	tRNA-uridine aminocarboxypropyltransferase 1	DTWD1	Homo sapiens	35.249	2	2	76249	0
7744	1	Q8N5C8	Q8N5C8	TGF-beta-activated kinase 1 and MAP3K7-binding protein 3	TAB3	Homo sapiens	78.655	2	2	32901	0
7745	1	Q8N5S9	Q8N5S9	Calcium/calmodulin-dependent protein kinase kinase 1	CAMKK1	Homo sapiens	55.737	2	2	68737	0
7746	1	Q8N5W9	Q8N5W9	Refilin-B	RFLNB	Homo sapiens	22.879	2	2	154555	620543
7747	1	Q8N5Y8	Q8N5Y8	Protein mono-ADP-ribosyltransferase PARP16	PARP16	Homo sapiens	36.384	2	2	176938	96330
7748	1	Q8N6N3	Q8N6N3	UPF0690 protein C1orf52	C1orf52	Homo sapiens	20.598	2	2	251576	237148
7749	1	Q8N6S4	Q8N6S4	Ankyrin repeat domain-containing protein 13C	ANKRD13C	Homo sapiens	60.82	2	2	16079	20306
7750	1	Q8N6S5	Q8N6S5	ADP-ribosylation factor-like protein 6-interacting protein 6	ARL6IP6	Homo sapiens	24.678	2	2	440476	141163
7751	1	Q8N6T7	Q8N6T7	NAD-dependent protein deacylase sirtuin-6	SIRT6	Homo sapiens	39.118	2	2	251031	173498
7752	1	Q8N755	Q8N755	Solute carrier family 66 member 3	SLC66A3	Homo sapiens	22.576	2	2	177370	0
7753	1	Q8N7R7	Q8N7R7	Cyclin-Y-like protein 1	CCNYL1	Homo sapiens	40.706	2	2	0	101082
7754	1	Q8N8Q8	Q8N8Q8	Cytochrome c oxidase assembly protein COX18, mitochondrial	COX18	Homo sapiens	37.063	2	2	100139	0
7755	1	Q8N954	Q8N954	G patch domain-containing protein 11	GPATCH11	Homo sapiens	33.277	2	2	85641	25845

7756	1	Q8N9I9	Q8N9I9	Probable E3 ubiquitin-protein ligase DTX3	DTX3	Homo sapiens	37.989	2	2	342819	0
7757	1	Q8N9M1	Q8N9M1	Uncharacterized protein C19orf47	C19orf47	Homo sapiens	44.746	2	2	51749	12776
7758	1	Q8N9M5	Q8N9M5	Transmembrane protein 102	TMEM102	Homo sapiens	54.175	2	2	219328	50591
7759	1	Q8N9N2	Q8N9N2	Activating signal cointegrator 1 complex subunit 1	ASCC1	Homo sapiens	45.507	2	2	12529	52382
7760	1	Q8N9R8	Q8N9R8	Protein SCAI	SCAI	Homo sapiens	70.396	2	2	147950	0
7761	1	Q8N9V3	Q8N9V3	WD repeat, SAM and U-box domain-containing protein 1	WDSUB1	Homo sapiens	52.833	2	2	169451	98590
7762	1	Q8NB14	Q8NB14	Ubiquitin carboxyl-terminal hydrolase 38	USP38	Homo sapiens	116.548	2	2	67901	0
7763	1	Q8NB46	Q8NB46	Serine/threonine-protein phosphatase 6 regulatory ankyrin repeat subunit C	ANKRD52	Homo sapiens	115.08	2	2	55599	0
7764	1	Q8NBE8	Q8NBE8	Kelch-like protein 23	KLHL23	Homo sapiens	63.922	2	2	79500	0
7765	1	Q8NCR9	Q8NCR9	Clarin-3	CLRN3	Homo sapiens	25.321	2	2	481785	0
7766	1	Q8NCW6	Q8NCW6	Polypeptide N-acetylgalactosaminyltransferase 11	GALNT11	Homo sapiens	68.916	2	2	87710	182927
7767	1	Q8ND76	Q8ND76	Cyclin-Y	CCNY	Homo sapiens	39.336	3	2	304875	184388
7768	1	Q8ND82	Q8ND82	Zinc finger protein 280C	ZNF280C	Homo sapiens	83.097	2	2	60034	0
7769	1	Q8NDD1	Q8NDD1	Uncharacterized protein C1orf131	C1orf131	Homo sapiens	32.61	2	2	207720	0
7770	1	Q8NDN9	Q8NDN9	RCC1 and BTB domain-containing protein 1	RCBTB1	Homo sapiens	58.255	2	2	104823	0
7771	1	Q8NDZ6	Q8NDZ6	Transmembrane protein 161B	TMEM161B	Homo sapiens	55.48	2	2	23236	0
7772	1	Q8NEM7	Q8NEM7	Transcription factor SPT20 homolog	SUPT20H	Homo sapiens	85.791	2	2	54192	0
7773	1	Q8NES3	Q8NES3	Beta-1,3-N-acetylglucosaminyltransferase lunatic fringe	LFNG	Homo sapiens	41.773	2	2	234409	61085
7774	1	Q8NFA2	Q8NFA2	NADPH oxidase organizer 1	NOXO1	Homo sapiens	41.253	2	2	85094	0
7775	1	Q8NFW9	Q8NFW9	Rab effector MyRIP	MYRIP	Homo sapiens	95.709	2	2	42789	0
7776	1	Q8NG27	Q8NG27	E3 ubiquitin-protein ligase Praja-1	PJA1	Homo sapiens	71.001	2	2	76501	0
7777	1	Q8NHG7	Q8NHG7	Small VCP/p97-interacting protein	SVIP	Homo sapiens	8.44	2	2	261321	470564
7778	1	Q8NI22	Q8NI22	Multiple coagulation factor deficiency protein 2	MCFD2	Homo sapiens	16.392	2	2	845640	793079
7779	1	Q8TAD8	Q8TAD8	Smad nuclear-interacting protein 1	SNIP1	Homo sapiens	45.776	2	2	256136	23339
7780	1	Q8TAM2	Q8TAM2	Tetratricopeptide repeat protein 8	TTC8	Homo sapiens	61.533	2	2	95503	0
7781	1	Q8TB40	Q8TB40	(Lyso)-N-acylphosphatidylethanolamine lipase	ABHD4	Homo sapiens	38.793	2	2	172001	86277
7782	1	Q8TCB0	Q8TCB0	Interferon-induced protein 44	IFI44	Homo sapiens	50.49	2	2	51806	155408
7783	1	Q8TCC3	Q8TCC3	39S ribosomal protein L30, mitochondrial	MRPL30	Homo sapiens	18.544	2	2	225202	24167
7784	1	Q8TCT1	Q8TCT1	Phosphoethanolamine/phosphocholine phosphatase	PHOSPHO1	Homo sapiens	29.711	2	2	0	292988
7785	1	Q8TCT7	Q8TCT7	Signal peptide peptidase-like 2B	SPPL2B	Homo sapiens	64.645	2	2	279818	16970
7786	1	Q8TD30	Q8TD30	Alanine aminotransferase 2	GPT2	Homo sapiens	57.905	2	2	0	44855
7787	1	Q8TDP1	Q8TDP1	Ribonuclease H2 subunit C	RNASEH2C	Homo sapiens	17.84	2	2	60416	254018
7788	1	Q8TDQ1	Q8TDQ1	CMRF35-like molecule 1	CD300LF	Homo sapiens	32.336	2	2	0	162835
7789	1	Q8TDR0	Q8TDR0	TRAF3-interacting protein 1	TRAF3IP1	Homo sapiens	78.635	2	2	93157	0
7790	1	Q8TDY4	Q8TDY4	Arf-GAP with SH3 domain, ANK repeat and PH domain-containing protein 3	ASAP3	Homo sapiens	99.156	2	2	82350	0
7791	1	Q8TEA1	Q8TEA1	tRNA (cytosine(72)-C(5))-methyltransferase NSUN6	NSUN6	Homo sapiens	51.77	2	2	23179	16647
7792	1	Q8TEA8	Q8TEA8	D-aminoacyl-tRNA deacylase 1	DTD1	Homo sapiens	23.424	2	2	1313206	604254
7793	1	Q8TEJ3	Q8TEJ3	E3 ubiquitin-protein ligase SH3RF3	SH3RF3	Homo sapiens	92.776	2	2	56816	0
7794	1	Q8TEL6	Q8TEL6	Short transient receptor potential channel 4-associated protein	TRPC4AP	Homo sapiens	90.854	2	2	60922	0
7795	1	Q8TF65	Q8TF65	PDZ domain-containing protein GIPC2	GIPC2	Homo sapiens	34.355	2	2	783638	0
7796	1	Q8TF71	Q8TF71	Monocarboxylate transporter 10	SLC16A10	Homo sapiens	55.492	2	2	20829	175168
7797	1	Q8WU10	Q8WU10	Pyridine nucleotide-disulfide oxidoreductase domain-containing protein 1	PYROXD1	Homo sapiens	55.795	2	2	78283	55755
7798	1	Q8WUD6	Q8WUD6	Cholinephosphotransferase 1	CHPT1	Homo sapiens	45.097	2	2	368400	145456
7799	1	Q8WUR7	Q8WUR7	UPF0235 protein C15orf40	C15orf40	Homo sapiens	16.35	2	2	303916	0
7800	1	Q8WUX2	Q8WUX2	Glutathione-specific gamma-glutamylcyclotransferase 2	CHAC2	Homo sapiens	20.875	2	2	16184	235126
7801	1	Q8WUZ0	Q8WUZ0	B-cell CLL/lymphoma 7 protein family member C	BCL7C	Homo sapiens	23.468	3	2	243922	286460

7802	1 Q8WV92	Q8WV92	MIT domain-containing protein 1	MITD1	Homo sapiens	29.314	2	2	79444	0
7803	1 Q8WVD5	Q8WVD5	RING finger protein 141	RNF141	Homo sapiens	25.535	2	2	109409	334263
7804	1 Q8WVI0	Q8WVI0	Ubiquinol-cytochrome-c reductase complex assembly factor 5	UQCC5	Homo sapiens	8.696	2	2	83098	17395
7805	1 Q8WVL7	Q8WVL7	Ankyrin repeat domain-containing protein 49	ANKRD49	Homo sapiens	27.29	2	2	61051	0
7806	1 Q8WVN6	Q8WVN6	Secreted and transmembrane protein 1	SECTM1	Homo sapiens	27.039	2	2	217722	486256
7807	1 Q8WVR3	Q8WVR3	Trafficking protein particle complex subunit 14	TRAPPC14	Homo sapiens	62.598	2	2	47677	0
7808	1 Q8WW01	Q8WW01	tRNA-splicing endonuclease subunit Sen15	TSEN15	Homo sapiens	18.64	2	2	119314	0
7809	1 Q8WXB1	Q8WXB1	Protein N-lysine methyltransferase METTL21A	METTL21A	Homo sapiens	24.601	2	2	46100	109821
7810	1 Q8WY21	Q8WY21	VPS10 domain-containing receptor SorCS1	SORCS1	Homo sapiens	129.636	2	2	61137	0
7811	1 Q8WY22	Q8WY22	BRI3-binding protein	BRI3BP	Homo sapiens	27.834	2	2	2226959	3809109
7812	1 Q8WY36	Q8WY36	HMG box transcription factor BBX	BBX	Homo sapiens	105.132	2	2	114105	0
7813	1 Q8WY91	Q8WY91	Peroxynitrite isomerase THAP4	THAP4	Homo sapiens	62.89	2	2	137053	0
7814	1 Q8WYN0	Q8WYN0	Cysteine protease ATG4A	ATG4A	Homo sapiens	45.379	2	2	155588	0
7815	1 Q8WZ73	Q8WZ73	E3 ubiquitin-protein ligase rififylin	RFFL	Homo sapiens	40.512	2	2	24603	25477
7816	1 Q92478	Q92478	C-type lectin domain family 2 member B	CLEC2B	Homo sapiens	17.307	2	2	65855	394630
7817	1 Q92496	Q92496	Complement factor H-related protein 4	CFHR4	Homo sapiens	65.353	2	2	61188	428478
7818	1 Q92564	Q92564	DCN1-like protein 4	DCUN1D4	Homo sapiens	34.069	2	2	129358	0
7819	1 Q92611	Q92611	ER degradation-enhancing alpha-mannosidase-like protein 1	EDEM1	Homo sapiens	73.767	2	2	62522	0
7820	1 Q92623	Q92623	Tetratricopeptide repeat protein 9A	TTC9	Homo sapiens	24.379	2	2	58154	73079
7821	1 Q92637	Q92637	Putative high affinity immunoglobulin gamma Fc receptor IB	FCGR1BP	Homo sapiens	32.232	2	2	0	95468
7822	1 Q92738	Q92738	USP6 N-terminal-like protein	USP6NL	Homo sapiens	94.104	2	2	115895	0
7823	1 Q92826	Q92826	Homeobox protein Hox-B13	HOXB13	Homo sapiens	30.678	2	2	208890	0
7824	1 Q92831	Q92831	Histone acetyltransferase KAT2B	KAT2B	Homo sapiens	93.015	3	2	95790	0
7825	1 Q92903	Q92903	Phosphatidate cytidylyltransferase 1	CDS1	Homo sapiens	53.306	2	2	75848	0
7826	1 Q92956	Q92956	Tumor necrosis factor receptor superfamily member 14	TNFRSF14	Homo sapiens	30.392	2	2	132657	32377
7827	1 Q92968	Q92968	Peroxisomal membrane protein PEX13	PEX13	Homo sapiens	44.129	2	2	168925	204090
7828	1 Q92993	Q92993	Histone acetyltransferase KAT5	KAT5	Homo sapiens	58.582	2	2	8877	12920
7829	1 Q92994	Q92994	Transcription factor IIIB 90 kDa subunit	BRF1	Homo sapiens	73.842	2	2	103277	0
7830	1 Q93015	Q93015	N-alpha-acetyltransferase 80	NAA80	Homo sapiens	31.446	2	2	28064	61746
7831	1 Q93088	Q93088	Betaine--homocysteine S-methyltransferase 1	BHMT	Homo sapiens	44.999	2	2	93253	18105
7832	1 Q93091	Q93091	Ribonuclease K6	RNASE6	Homo sapiens	17.197	2	2	622150	0
7833	1 Q93096	Q93096	Protein tyrosine phosphatase type IVA 1	PTP4A1	Homo sapiens	19.814	4	2	99571	0
7834	1 Q969E2	Q969E2	Secretory carrier-associated membrane protein 4	SCAMP4	Homo sapiens	25.727	2	2	856164	863532
7835	1 Q969E4	Q969E4	Transcription elongation factor A protein-like 3	TCEAL3	Homo sapiens	22.5	6	2	1072500	283702
7836	1 Q969G6	Q969G6	Riboflavin kinase	RFK	Homo sapiens	17.622	2	2	19682	116482
7837	1 Q969M1	Q969M1	Mitochondrial import receptor subunit TOM40B	TOMM40L	Homo sapiens	33.916	2	2	556302	0
7838	1 Q969M3	Q969M3	Protein YIPF5	YIPF5	Homo sapiens	27.988	2	2	912897	910788
7839	1 Q969X1	Q969X1	Protein lifeguard 3	TMBIM1	Homo sapiens	34.607	2	2	1440013	3471250
7840	1 Q96A23	Q96A23	Copine-4	CPNE4	Homo sapiens	62.395	2	2	143971	0
7841	1 Q96AA3	Q96AA3	Protein RFT1 homolog	RFT1	Homo sapiens	60.337	2	2	396038	507601
7842	1 Q96AH8	Q96AH8	Ras-related protein Rab-7b	RAB7B	Homo sapiens	22.511	2	2	0	85315
7843	1 Q96AM1	Q96AM1	Mas-related G-protein coupled receptor member F	MRGPRF	Homo sapiens	38.172	2	2	4931434	0
7844	1 Q96AN5	Q96AN5	Transmembrane protein 143	TMEM143	Homo sapiens	51.714	2	2	47769	0
7845	1 Q96AQ8	Q96AQ8	Mitochondrial calcium uniporter regulator 1	MCUR1	Homo sapiens	39.695	2	2	265277	0
7846	1 Q96AT9	Q96AT9	Ribulose-phosphate 3-epimerase	RPE	Homo sapiens	24.929	3	2	270283	0
7847	1 Q96B21	Q96B21	Transmembrane protein 45B	TMEM45B	Homo sapiens	31.826	2	2	251463	0
7848	1 Q96B45	Q96B45	BLOC-1-related complex subunit 7	BORCS7	Homo sapiens	11.694	2	2	0	170831
7849	1 Q96B54	Q96B54	Zinc finger protein 428	ZNF428	Homo sapiens	20.479	2	2	291828	0

7850	1	Q96B70	Q96B70	Leukocyte receptor cluster member 9	LENG9	Homo sapiens	53.167	2	2	123391	0
7851	1	Q96B86	Q96B86	Repulsive guidance molecule A	RGMA	Homo sapiens	49.362	2	2	134601	0
7852	1	Q96BF6	Q96BF6	Nucleus accumbens-associated protein 2	NACC2	Homo sapiens	62.836	2	2	30680	231111
7853	1	Q96BI3	Q96BI3	Gamma-secretase subunit APH-1A	APH1A	Homo sapiens	28.996	2	2	147021	264583
7854	1	Q96BK5	Q96BK5	PIN2/TERF1-interacting telomerase inhibitor 1	PINX1	Homo sapiens	37.033	2	2	85311	21882
7855	1	Q96BN2	Q96BN2	Transcriptional adapter 1	TADA1	Homo sapiens	37.384	2	2	60097	0
7856	1	Q96BP2	Q96BP2	Coiled-coil-helix-coiled-coil-helix domain-containing protein 1	CHCHD1	Homo sapiens	13.474	2	2	610450	198514
7857	1	Q96BW9	Q96BW9	Phosphatidate cytidylyltransferase, mitochondrial	TAMM41	Homo sapiens	51.067	2	2	185232	20931
7858	1	Q96BY9	Q96BY9	Store-operated calcium entry-associated regulatory factor	SARAF	Homo sapiens	36.979	2	2	144354	73783
7859	1	Q96C90	Q96C90	Protein phosphatase 1 regulatory subunit 14B	PPP1R14B	Homo sapiens	15.91	2	2	897174	525511
7860	1	Q96CD2	Q96CD2	Phosphopantothenoylcysteine decarboxylase	PPCDC	Homo sapiens	22.395	2	2	192124	0
7861	1	Q96CG8	Q96CG8	Collagen triple helix repeat-containing protein 1	CTHRC1	Homo sapiens	26.222	2	2	30882	193369
7862	1	Q96CP2	Q96CP2	FLYWCH family member 2	FLYWCH2	Homo sapiens	14.562	3	2	214085	111421
7863	1	Q96CQ1	Q96CQ1	Solute carrier family 25 member 36	SLC25A36	Homo sapiens	34.282	3	2	57005	0
7864	1	Q96D15	Q96D15	Reticulocalbin-3	RCN3	Homo sapiens	37.492	9	2	336379	289943
7865	1	Q96DA2	Q96DA2	Ras-related protein Rab-39B	RAB39B	Homo sapiens	24.621	2	2	180363	0
7866	1	Q96DF8	Q96DF8	Splicing factor ESS-2 homolog	ESS2	Homo sapiens	52.567	2	2	40983	0
7867	1	Q96DT0	Q96DT0	Galectin-12	LGALS12	Homo sapiens	37.539	2	2	0	79322
7868	1	Q96DX4	Q96DX4	RING finger and SPRY domain-containing protein 1	RSPRY1	Homo sapiens	64.18	2	2	98150	65932
7869	1	Q96E17	Q96E17	Ras-related protein Rab-3C	RAB3C	Homo sapiens	25.953	2	2	228761	0
7870	1	Q96E22	Q96E22	Dehydrodolichyl diphosphate synthase complex subunit NUS1	NUS1	Homo sapiens	33.226	2	2	44198	59512
7871	1	Q96E39	Q96E39	RNA binding motif protein, X-linked-like-1	RBMXL1	Homo sapiens	42.14	2	2	158303	42297
7872	1	Q96EH3	Q96EH3	Mitochondrial assembly of ribosomal large subunit protein 1	MALSU1	Homo sapiens	26.17	2	2	341907	269263
7873	1	Q96ES6	Q96ES6	Major facilitator superfamily domain-containing protein 3	MFSD3	Homo sapiens	42.694	2	2	74340	110343
7874	1	Q96EW2	Q96EW2	HSPB1-associated protein 1	HSPBAP1	Homo sapiens	55.166	2	2	62478	79105
7875	1	Q96EX3	Q96EX3	Cytoplasmic dynein 2 intermediate chain 2	DYNC2I2	Homo sapiens	57.801	2	2	105723	0
7876	1	Q96F25	Q96F25	UDP-N-acetylglucosamine transferase subunit ALG14 homolog	ALG14	Homo sapiens	24.151	2	2	384297	0
7877	1	Q96F63	Q96F63	Coiled-coil domain-containing protein 97	CCDC97	Homo sapiens	38.949	2	2	112613	0
7878	1	Q96FC7	Q96FC7	Phytanoyl-CoA hydroxylase-interacting protein-like	PHYHIPL	Homo sapiens	42.487	2	2	326683	0
7879	1	Q96FJ0	Q96FJ0	AMSH-like protease	STAMBPL1	Homo sapiens	49.784	2	2	156646	82305
7880	1	Q96FN9	Q96FN9	D-aminoacyl-tRNA deacylase 2	DTD2	Homo sapiens	18.662	2	2	750779	0
7881	1	Q96FX7	Q96FX7	tRNA (adenine(58)-N(1))-methyltransferase catalytic subunit TRMT61A	TRMT61A	Homo sapiens	31.384	2	2	145455	60129
7882	1	Q96G21	Q96G21	U3 small nucleolar ribonucleoprotein protein IMP4	IMP4	Homo sapiens	33.758	2	2	173225	151256
7883	1	Q96G97	Q96G97	Seipin	BSCL2	Homo sapiens	44.39	2	2	453890	100758
7884	1	Q96GK7	Q96GK7	Fumarylacetoacetate hydrolase domain-containing protein 2A	FAHD2A	Homo sapiens	34.595	10	2	1315224	193288
7885	1	Q96GS6	Q96GS6	Alpha/beta hydrolase domain-containing protein 17A	ABHD17A	Homo sapiens	33.99	2	2	57310	170221
7886	1	Q96GX1	Q96GX1	Tectonic-2	TCTN2	Homo sapiens	76.874	2	2	60898	0
7887	1	Q96HA8	Q96HA8	Protein N-terminal glutamine amidohydrolase	NTAQ1	Homo sapiens	23.681	2	2	38927	0
7888	1	Q96HF1	Q96HF1	Secreted frizzled-related protein 2	SFRP2	Homo sapiens	33.491	2	2	215159	69634
7889	1	Q96HP4	Q96HP4	Oxidoreductase NAD-binding domain-containing protein 1	OXNAD1	Homo sapiens	34.855	2	2	121064	10593
7890	1	Q96HV5	Q96HV5	Transmembrane protein 41A	TMEM41A	Homo sapiens	29.664	2	2	140699	181086
7891	1	Q96IW7	Q96IW7	Vesicle-trafficking protein SEC22a	SEC22A	Homo sapiens	34.947	2	2	306213	208007
7892	1	Q96IX5	Q96IX5	ATP synthase membrane subunit K, mitochondrial	ATP5MK	Homo sapiens	6.454	2	2	1699074	583251
7893	1	Q96IY1	Q96IY1	Kinetochore-associated protein NSL1 homolog	NSL1	Homo sapiens	32.161	2	2	440000	71577
7894	1	Q96IZ7	Q96IZ7	Serine/Arginine-related protein 53	RSRC1	Homo sapiens	38.675	2	2	253077	44695
7895	1	Q96J42	Q96J42	Thioredoxin domain-containing protein 15	TXNDC15	Homo sapiens	39.886	2	2	330766	0
7896	1	Q96J88	Q96J88	Epithelial-stromal interaction protein 1	EPSTI1	Homo sapiens	36.792	2	2	111028	0
7897	1	Q96JM7	Q96JM7	Lethal(3)malignant brain tumor-like protein 3	L3MBTL3	Homo sapiens	88.337	2	2	157134	0

7898	1 Q96JZ2	Q96JZ2	Hematopoietic SH2 domain-containing protein	HSH2D	Homo sapiens	39.002	2	2	0	134602
7899	1 Q96K17	Q96K17	Transcription factor BTF3 homolog 4	BTF3L4	Homo sapiens	17.268	2	2	640361	280182
7900	1 Q96KN1	Q96KN1	Protein LRATD2	LRATD2	Homo sapiens	34.474	2	2	339324	8562
7901	1 Q96KQ4	Q96KQ4	Apoptosis-stimulating of p53 protein 1	PPP1R13B	Homo sapiens	119.566	2	2	4262191	0
7902	1 Q96KQ7	Q96KQ7	Histone-lysine N-methyltransferase EHMT2	EHMT2	Homo sapiens	132.375	2	2	73574	0
7903	1 Q96L08	Q96L08	Sushi domain-containing protein 3	SUSD3	Homo sapiens	27.12	2	2	0	89324
7904	1 Q96LD8	Q96LD8	Sentrin-specific protease 8	SENP8	Homo sapiens	24.107	2	2	50831	0
7905	1 Q96LJ8	Q96LJ8	UBX domain-containing protein 10	UBXN10	Homo sapiens	30.812	2	2	94048	0
7906	1 Q96MG8	Q96MG8	Protein-L-isoaspartate O-methyltransferase domain-containing protein 1	PCMTD1	Homo sapiens	40.674	2	2	206988	25543
7907	1 Q96MK3	Q96MK3	Pseudokinase FAM20A	FAM20A	Homo sapiens	61.415	2	2	0	45541
7908	1 Q96MP8	Q96MP8	BTB/POZ domain-containing protein KCTD7	KCTD7	Homo sapiens	33.134	2	2	85662	0
7909	1 Q96N21	Q96N21	AP-4 complex accessory subunit Tepsin	TEPSIN	Homo sapiens	55.137	2	2	18916	0
7910	1 Q96NT0	Q96NT0	Coiled-coil domain-containing protein 115	CCDC115	Homo sapiens	19.761	2	2	186160	370176
7911	1 Q96NT5	Q96NT5	Proton-coupled folate transporter	SLC46A1	Homo sapiens	49.772	2	2	195329	136045
7912	1 Q96PC3	Q96PC3	AP-1 complex subunit sigma-3	AP1S3	Homo sapiens	18.279	2	2	102360	79111
7913	1 Q96PL5	Q96PL5	Erythroid membrane-associated protein	ERMAP	Homo sapiens	52.604	2	2	0	135452
7914	1 Q96PM5	Q96PM5	RING finger and CHY zinc finger domain-containing protein 1	RCHY1	Homo sapiens	30.111	2	2	57067	35487
7915	1 Q96PM9	Q96PM9	Zinc finger protein 385A	ZNF385A	Homo sapiens	40.454	2	2	85923	0
7916	1 Q96PQ7	Q96PQ7	Kelch-like protein 5	KLHL5	Homo sapiens	84.46	2	2	161017	0
7917	1 Q96PZ2	Q96PZ2	Serine protease FAM111A	FAM111A	Homo sapiens	70.196	2	2	12656	63739
7918	1 Q96Q27	Q96Q27	Ankyrin repeat and SOCS box protein 2	ASB2	Homo sapiens	70.213	2	2	130405	0
7919	1 Q96QE5	Q96QE5	Transcription elongation factor, mitochondrial	TEFM	Homo sapiens	41.677	2	2	145332	0
7920	1 Q96QT4	Q96QT4	Transient receptor potential cation channel subfamily M member 7	TRPM7	Homo sapiens	212.703	2	2	42066	0
7921	1 Q96RD7	Q96RD7	Pannexin-1	PANX1	Homo sapiens	48.051	2	2	27671	107778
7922	1 Q96RP7	Q96RP7	Galactose-3-O-sulfotransferase 4	GAL3ST4	Homo sapiens	54.167	2	2	125276	71106
7923	1 Q96S53	Q96S53	Dual specificity testis-specific protein kinase 2	TESK2	Homo sapiens	63.641	2	2	29214	142994
7924	1 Q96S94	Q96S94	Cyclin-L2	CCNL2	Homo sapiens	58.147	2	2	132803	0
7925	1 Q96SK2	Q96SK2	Transmembrane protein 209	TMEM209	Homo sapiens	62.922	2	2	600677	111552
7926	1 Q96SN8	Q96SN8	CDK5 regulatory subunit-associated protein 2	CDK5RAP2	Homo sapiens	215.042	2	2	94998	0
7927	1 Q99062	Q99062	Granulocyte colony-stimulating factor receptor	CSF3R	Homo sapiens	92.157	2	2	0	1006387
7928	1 Q99081	Q99081	Transcription factor 12	TCF12	Homo sapiens	72.966	2	2	105458	0
7929	1 Q99418	Q99418	Cytohesin-2	CYTH2	Homo sapiens	46.547	3	2	188848	0
7930	1 Q99470	Q99470	Stromal cell-derived factor 2	SDF2	Homo sapiens	23.027	2	2	439099	500348
7931	1 Q99501	Q99501	GAS2-like protein 1	GAS2L1	Homo sapiens	72.718	2	2	51002	0
7932	1 Q99504	Q99504	Eyes absent homolog 3	EYA3	Homo sapiens	62.662	2	2	73457	32990
7933	1 Q99538	Q99538	Legumain	LGMN	Homo sapiens	49.411	2	2	685078	199995
7934	1 Q99551	Q99551	Transcription termination factor 1, mitochondrial	MTERF1	Homo sapiens	45.777	2	2	44224	0
7935	1 Q99576	Q99576	TSC22 domain family protein 3	TSC22D3	Homo sapiens	14.809	2	2	156033	307273
7936	1 Q99594	Q99594	Transcriptional enhancer factor TEF-5	TEAD3	Homo sapiens	48.673	2	2	540186	0
7937	1 Q99626	Q99626	Homeobox protein CDX-2	CDX2	Homo sapiens	33.519	4	2	203317	0
7938	1 Q99638	Q99638	Cell cycle checkpoint control protein RAD9A	RAD9A	Homo sapiens	42.547	2	2	45927	19099
7939	1 Q99650	Q99650	Oncostatin-M-specific receptor subunit beta	OSMR	Homo sapiens	110.509	2	2	5906	36445
7940	1 Q99674	Q99674	Cell growth regulator with EF hand domain protein 1	CGREF1	Homo sapiens	33.456	2	2	40146	0
7941	1 Q99708	Q99708	DNA endonuclease RBBP8	RBBP8	Homo sapiens	101.944	2	2	0	152418
7942	1 Q99712	Q99712	ATP-sensitive inward rectifier potassium channel 15	KCNJ15	Homo sapiens	42.577	2	2	57597	55885
7943	1 Q99717	Q99717	Mothers against decapentaplegic homolog 5	SMAD5	Homo sapiens	52.26	2	2	374128	0
7944	1 Q99735	Q99735	Microsomal glutathione S-transferase 2	MGST2	Homo sapiens	16.621	2	2	13549503	9949793
7945	1 Q99757	Q99757	Thioredoxin, mitochondrial	TXN2	Homo sapiens	18.379	2	2	879479	137211

7946	1	Q99871	Q99871	HAUS augmin-like complex subunit 7	HAUS7	Homo sapiens	39.794	2	2	19689	12568
7947	1	Q9BPV8	Q9BPV8	P2Y purinoceptor 13	P2RY13	Homo sapiens	40.79	2	2	0	760963
7948	1	Q9BQ13	Q9BQ13	BTB/POZ domain-containing protein KCTD14	KCTD14	Homo sapiens	29.592	2	2	878631	75793
7949	1	Q9BQ48	Q9BQ48	39S ribosomal protein L34, mitochondrial	MRPL34	Homo sapiens	10.164	2	2	675669	31613
7950	1	Q9BQB6	Q9BQB6	Vitamin K epoxide reductase complex subunit 1	VKORC1	Homo sapiens	18.232	2	2	200258	203051
7951	1	Q9BQI0	Q9BQI0	Allograft inflammatory factor 1-like	AIF1L	Homo sapiens	17.069	2	2	248591	0
7952	1	Q9BQT8	Q9BQT8	Mitochondrial 2-oxodicarboxylate carrier	SLC25A21	Homo sapiens	33.302	2	2	239722	0
7953	1	Q9BQT9	Q9BQT9	Calsyntenin-3	CLSTN3	Homo sapiens	106.1	2	2	21294	54350
7954	1	Q9BR61	Q9BR61	Acyl-CoA-binding domain-containing protein 6	ACBD6	Homo sapiens	31.15	2	2	206459	0
7955	1	Q9BRG2	Q9BRG2	SH2 domain-containing protein 3A	SH2D3A	Homo sapiens	63.096	2	2	71112	0
7956	1	Q9BRT2	Q9BRT2	Ubiquinol-cytochrome-c reductase complex assembly factor 2	UQCC2	Homo sapiens	14.874	2	2	1125351	303341
7957	1	Q9BRT3	Q9BRT3	Migration and invasion enhancer 1	MIEN1	Homo sapiens	12.402	2	2	1508929	4054468
7958	1	Q9BRU9	Q9BRU9	rRNA-processing protein UTP23 homolog	UTP23	Homo sapiens	28.4	2	2	51500	22156
7959	1	Q9BRX5	Q9BRX5	DNA replication complex GINS protein PSF3	GINS3	Homo sapiens	24.534	2	2	88016	20295
7960	1	Q9BSB4	Q9BSB4	Autophagy-related protein 101	ATG101	Homo sapiens	25.004	2	2	45703	57813
7961	1	Q9BSR8	Q9BSR8	Protein YIPF4	YIPF4	Homo sapiens	27.082	2	2	544478	335982
7962	1	Q9BST9	Q9BST9	Rhotekin	RTKN	Homo sapiens	62.667	2	2	48954	0
7963	1	Q9BT23	Q9BT23	LIM domain-containing protein 2	LIMD2	Homo sapiens	14.068	2	2	94039	171303
7964	1	Q9BT73	Q9BT73	Proteasome assembly chaperone 3	PSMG3	Homo sapiens	13.105	2	2	272692	0
7965	1	Q9BTV5	Q9BTV5	Fibronectin type III and SPRY domain-containing protein 1	FSD1	Homo sapiens	55.819	2	2	51949	0
7966	1	Q9BTV6	Q9BTV6	Diphthine methyltransferase	DPH7	Homo sapiens	50.575	2	2	69407	0
7967	1	Q9BUE0	Q9BUE0	Mediator of RNA polymerase II transcription subunit 18	MED18	Homo sapiens	23.662	2	2	408519	289457
7968	1	Q9BUN8	Q9BUN8	Derlin-1	DERL1	Homo sapiens	28.799	2	2	1729147	920014
7969	1	Q9BUV8	Q9BUV8	GEL complex subunit OPTI	RAB5IF	Homo sapiens	15.489	2	2	343374	660594
7970	1	Q9BV94	Q9BV94	ER degradation-enhancing alpha-mannosidase-like protein 2	EDEM2	Homo sapiens	64.752	2	2	609900	110813
7971	1	Q9BVC6	Q9BVC6	Transmembrane protein 109	TMEM109	Homo sapiens	26.21	2	2	8567217	2588844
7972	1	Q9BVG8	Q9BVG8	Kinesin-like protein KIFC3	KIFC3	Homo sapiens	92.777	2	2	0	38657
7973	1	Q9BVG9	Q9BVG9	Phosphatidylserine synthase 2	PTDSS2	Homo sapiens	56.253	2	2	338148	206643
7974	1	Q9BVS5	Q9BVS5	tRNA (adenine(58)-N(1))-methyltransferase, mitochondrial	TRMT61B	Homo sapiens	52.963	2	2	196185	12765
7975	1	Q9BWF3	Q9BWF3	RNA-binding protein 4	RBM4	Homo sapiens	40.313	11	2	116804	28560
7976	1	Q9BWP8	Q9BWP8	Collectin-11	COLEC11	Homo sapiens	28.664	2	2	0	433648
7977	1	Q9BWT3	Q9BWT3	Poly(A) polymerase gamma	PAPOLG	Homo sapiens	82.805	2	2	30011	26904
7978	1	Q9BWU1	Q9BWU1	Cyclin-dependent kinase 19	CDK19	Homo sapiens	56.802	2	2	0	90788
7979	1	Q9BWV1	Q9BWV1	Brother of CDO	BOC	Homo sapiens	121.06	2	2	80127	0
7980	1	Q9BWW8	Q9BWW8	Apolipoprotein L6	APOL6	Homo sapiens	38.125	2	2	51503	253615
7981	1	Q9BX95	Q9BX95	Sphingosine-1-phosphate phosphatase 1	SGPP1	Homo sapiens	49.107	2	2	122881	0
7982	1	Q9BXC9	Q9BXC9	Bardet-Biedl syndrome 2 protein	BBS2	Homo sapiens	79.846	2	2	85316	0
7983	1	Q9BXJ0	Q9BXJ0	Complement C1q tumor necrosis factor-related protein 5	C1QTNF5	Homo sapiens	25.297	2	2	460243	100686
7984	1	Q9BXJ2	Q9BXJ2	Complement C1q tumor necrosis factor-related protein 7	C1QTNF7	Homo sapiens	30.683	2	2	399973	0
7985	1	Q9BXJ5	Q9BXJ5	Complement C1q tumor necrosis factor-related protein 2	C1QTNF2	Homo sapiens	29.951	2	2	268915	0
7986	1	Q9BXS4	Q9BXS4	Transmembrane protein 59	TMEM59	Homo sapiens	36.223	2	2	36548	0
7987	1	Q9BXY0	Q9BXY0	Protein MAK16 homolog	MAK16	Homo sapiens	35.369	2	2	1276728	141204
7988	1	Q9BY11	Q9BY11	Protein kinase C and casein kinase substrate in neurons protein 1	PACSIN1	Homo sapiens	50.966	2	2	189710	0
7989	1	Q9BY12	Q9BY12	S phase cyclin A-associated protein in the endoplasmic reticulum	SCAPER	Homo sapiens	158.289	2	2	89249	0
7990	1	Q9BY67	Q9BY67	Cell adhesion molecule 1	CADM1	Homo sapiens	48.508	2	2	1681309	34282
7991	1	Q9BYF1	Q9BYF1	Angiotensin-converting enzyme 2	ACE2	Homo sapiens	92.461	2	2	33173	49449
7992	1	Q9BYG4	Q9BYG4	Partitioning defective 6 homolog gamma	PARD6G	Homo sapiens	40.883	2	2	156265	0
7993	1	Q9BYI3	Q9BYI3	Hyccin	HYCC1	Homo sapiens	57.628	2	2	404022	0

7994	1	Q9BYZ8	Q9BYZ8	Regenerating islet-derived protein 4	REG4	Homo sapiens	18.232	2	2	1715988	545036
7995	1	Q9BZC7	Q9BZC7	ATP-binding cassette sub-family A member 2	ABCA2	Homo sapiens	269.837	2	2	63035	0
7996	1	Q9BZL1	Q9BZL1	Ubiquitin-like protein 5	UBL5	Homo sapiens	8.545	2	2	747807	103653
7997	1	Q9BZW5	Q9BZW5	Transmembrane 6 superfamily member 1	TM6SF1	Homo sapiens	41.635	2	2	300909	423599
7998	1	Q9C005	Q9C005	Protein dpy-30 homolog	DPY30	Homo sapiens	11.248	2	2	262498	176026
7999	1	Q9C019	Q9C019	Tripartite motif-containing protein 15	TRIM15	Homo sapiens	52.113	2	2	61797	0
8000	1	Q9C0B7	Q9C0B7	Transport and Golgi organization protein 6 homolog	TANGO6	Homo sapiens	120.749	2	2	89269	82061
8001	1	Q9C0F1	Q9C0F1	Centrosomal protein of 44 kDa	CEP44	Homo sapiens	44.14	2	2	115903	72987
8002	1	Q9C0H5	Q9C0H5	Rho GTPase-activating protein 39	ARHGAP39	Homo sapiens	121.288	2	2	62763	0
8003	1	Q9C0K0	Q9C0K0	B-cell lymphoma/leukemia 11B	BCL11B	Homo sapiens	95.522	2	2	45118	0
8004	1	Q9GZN4	Q9GZN4	Brain-specific serine protease 4	PRSS22	Homo sapiens	33.733	2	2	0	123555
8005	1	Q9GZR1	Q9GZR1	Sentrin-specific protease 6	SENP6	Homo sapiens	126.149	2	2	57595	0
8006	1	Q9GZT4	Q9GZT4	Serine racemase	SRR	Homo sapiens	36.566	2	2	38721	0
8007	1	Q9GZV5	Q9GZV5	WW domain-containing transcription regulator protein 1	WWTR1	Homo sapiens	44.102	2	2	150403	0
8008	1	Q9GZX9	Q9GZX9	Twisted gastrulation protein homolog 1	TWSG1	Homo sapiens	25.016	2	2	375096	87870
8009	1	Q9GZY4	Q9GZY4	Cytochrome c oxidase assembly factor 1 homolog	COA1	Homo sapiens	16.693	2	2	221605	46776
8010	1	Q9H0H5	Q9H0H5	Rac GTPase-activating protein 1	RACGAP1	Homo sapiens	71.027	2	2	98265	24393
8011	1	Q9H0M0	Q9H0M0	NEDD4-like E3 ubiquitin-protein ligase WWP1	WWP1	Homo sapiens	105.203	2	2	297949	0
8012	1	Q9H0R1	Q9H0R1	AP-5 complex subunit mu-1	AP5M1	Homo sapiens	54.765	2	2	63505	36466
8013	1	Q9H0R4	Q9H0R4	Haloacid dehalogenase-like hydrolase domain-containing protein 2	HDHD2	Homo sapiens	28.536	2	2	564024	68394
8014	1	Q9H0R5	Q9H0R5	Guanylate-binding protein 3	GBP3	Homo sapiens	68.116	2	2	95481	0
8015	1	Q9H171	Q9H171	Z-DNA-binding protein 1	ZBP1	Homo sapiens	46.342	2	2	0	166860
8016	1	Q9H1B5	Q9H1B5	Xylosyltransferase 2	XYLT2	Homo sapiens	96.769	2	2	86668	29829
8017	1	Q9H1C3	Q9H1C3	Glycosyltransferase 8 domain-containing protein 2	GLT8D2	Homo sapiens	40.026	2	2	161610	0
8018	1	Q9H1U4	Q9H1U4	Multiple epidermal growth factor-like domains protein 9	MEGF9	Homo sapiens	62.985	2	2	0	75232
8019	1	Q9H1X3	Q9H1X3	DnaJ homolog subfamily C member 25	DNAJC25	Homo sapiens	42.403	2	2	160652	0
8020	1	Q9H201	Q9H201	Epsin-3	EPN3	Homo sapiens	68.222	2	2	40836	0
8021	1	Q9H246	Q9H246	Uncharacterized protein C1orf21	C1orf21	Homo sapiens	13.864	2	2	202954	0
8022	1	Q9H299	Q9H299	SH3 domain-binding glutamic acid-rich-like protein 3	SH3BGRL3	Homo sapiens	10.436	2	2	24820773	34165060
8023	1	Q9H2A7	Q9H2A7	C-X-C motif chemokine 16	CXCL16	Homo sapiens	27.579	2	2	34778	284550
8024	1	Q9H2E6	Q9H2E6	Semaphorin-6A	SEMA6A	Homo sapiens	114.37	2	2	91660	0
8025	1	Q9H2F5	Q9H2F5	Enhancer of polycomb homolog 1	EPC1	Homo sapiens	93.463	2	2	59303	37113
8026	1	Q9H2G9	Q9H2G9	Golgin-45	BLZF1	Homo sapiens	44.913	2	2	113593	49749
8027	1	Q9H3C7	Q9H3C7	Gametogenetin-binding protein 2	GGNBP2	Homo sapiens	79.088	2	2	61461	0
8028	1	Q9H3H1	Q9H3H1	tRNA dimethylallyltransferase	TRIT1	Homo sapiens	52.724	2	2	60779	0
8029	1	Q9H3U5	Q9H3U5	Major facilitator superfamily domain-containing protein 1	MFSD1	Homo sapiens	51.209	2	2	264578	399883
8030	1	Q9H3U7	Q9H3U7	SPARC-related modular calcium-binding protein 2	SMOC2	Homo sapiens	49.675	2	2	44834	0
8031	1	Q9H4I2	Q9H4I2	Zinc fingers and homeoboxes protein 3	ZHX3	Homo sapiens	104.658	2	2	124258	0
8032	1	Q9H4K7	Q9H4K7	Mitochondrial ribosome-associated GTPase 2	MTG2	Homo sapiens	43.954	2	2	65018	0
8033	1	Q9H5K3	Q9H5K3	Protein O-mannose kinase	POMK	Homo sapiens	40.049	2	2	165218	0
8034	1	Q9H5V9	Q9H5V9	STING ER exit protein	STEEP1	Homo sapiens	25.624	2	2	165389	39443
8035	1	Q9H6V9	Q9H6V9	Lipid droplet-associated hydrolase	LDAH	Homo sapiens	37.317	2	2	297689	95074
8036	1	Q9H7E9	Q9H7E9	UPF0488 protein C8orf33	C8orf33	Homo sapiens	24.992	2	2	436687	0
8037	1	Q9H7J1	Q9H7J1	Protein phosphatase 1 regulatory subunit 3E	PPP1R3E	Homo sapiens	30.641	2	2	53270	48052
8038	1	Q9H7M9	Q9H7M9	V-type immunoglobulin domain-containing suppressor of T-cell activation	VSIR	Homo sapiens	33.907	2	2	87995	306747
8039	1	Q9H7P6	Q9H7P6	Multivesicular body subunit 12B	MVB12B	Homo sapiens	35.621	2	2	237053	167128
8040	1	Q9H7P9	Q9H7P9	Pleckstrin homology domain-containing family G member 2	PLEKHG2	Homo sapiens	147.97	2	2	59461	0
8041	1	Q9H867	Q9H867	Protein N-lysine methyltransferase METTL21D	VCPKMT	Homo sapiens	25.805	2	2	53384	0

8042	1	Q9H8G2	Q9H8G2	Caspase activity and apoptosis inhibitor 1	CAAP1	Homo sapiens	38.368	2	2	90975	34738
8043	1	Q9H8M2	Q9H8M2	Bromodomain-containing protein 9	BRD9	Homo sapiens	67.001	2	2	169567	63976
8044	1	Q9H981	Q9H981	Actin-related protein 8	ACTR8	Homo sapiens	70.483	2	2	247148	0
8045	1	Q9H9F9	Q9H9F9	Actin-related protein 5	ACTR5	Homo sapiens	68.3	2	2	46387	0
8046	1	Q9H9H4	Q9H9H4	Vacuolar protein sorting-associated protein 37B	VPS37B	Homo sapiens	31.308	2	2	608489	618760
8047	1	Q9H9L4	Q9H9L4	KAT8 regulatory NSL complex subunit 2	KANSL2	Homo sapiens	55.043	2	2	27102	11059
8048	1	Q9H9Q4	Q9H9Q4	Non-homologous end-joining factor 1	NHEJ1	Homo sapiens	33.337	2	2	327371	0
8049	1	Q9H9Y2	Q9H9Y2	Ribosome production factor 1	RPF1	Homo sapiens	40.111	2	2	65886	0
8050	1	Q9HA47	Q9HA47	Uridine-cytidine kinase 1	UCK1	Homo sapiens	31.434	2	2	278243	0
8051	1	Q9HAU4	Q9HAU4	E3 ubiquitin-protein ligase SMURF2	SMURF2	Homo sapiens	86.196	4	2	35738	0
8052	1	Q9HB90	Q9HB90	Ras-related GTP-binding protein C	RRAGC	Homo sapiens	44.223	8	2	757735	861150
8053	1	Q9HBG6	Q9HBG6	Intraflagellar transport protein 122 homolog	IFT122	Homo sapiens	141.827	2	2	141483	0
8054	1	Q9HBM1	Q9HBM1	Kinetochore protein Spc25	SPC25	Homo sapiens	26.15	2	2	88546	18941
8055	1	Q9HBY8	Q9HBY8	Serine/threonine-protein kinase Sgk2	SGK2	Homo sapiens	41.176	2	2	44952	0
8056	1	Q9HC36	Q9HC36	rRNA methyltransferase 3, mitochondrial	MRM3	Homo sapiens	47.019	2	2	554102	0
8057	1	Q9HC98	Q9HC98	Serine/threonine-protein kinase Nek6	NEK6	Homo sapiens	35.714	2	2	169922	118228
8058	1	Q9HCC8	Q9HCC8	Glycerophosphoinositol inositolphosphodiesterase GDPD2	GDPD2	Homo sapiens	61.73	2	2	48324	0
8059	1	Q9HCN6	Q9HCN6	Platelet glycoprotein VI	GP6	Homo sapiens	36.865	2	2	87391	0
8060	1	Q9HD34	Q9HD34	LYR motif-containing protein 4	LYRM4	Homo sapiens	10.757	2	2	746013	0
8061	1	Q9HD42	Q9HD42	Charged multivesicular body protein 1a	CHMP1A	Homo sapiens	21.7	2	2	1458910	130314
8062	1	Q9HDC5	Q9HDC5	Junctophilin-1	JPH1	Homo sapiens	71.685	2	2	500934	0
8063	1	Q9NP73	Q9NP73	Putative bifunctional UDP-N-acetylglucosamine transferase and deubiquitinase ALG13	ALG13	Homo sapiens	126.057	2	2	300692	63494
8064	1	Q9NPA8	Q9NPA8	Transcription and mRNA export factor ENY2	ENY2	Homo sapiens	11.525	2	2	744515	180707
8065	1	Q9NPF2	Q9NPF2	Carbohydrate sulfotransferase 11	CHST11	Homo sapiens	41.555	2	2	0	441690
8066	1	Q9NPF8	Q9NPF8	Arf-GAP with dual PH domain-containing protein 2	ADAP2	Homo sapiens	44.349	2	2	117894	93998
8067	1	Q9NPG3	Q9NPG3	Ubinuclein-1	UBN1	Homo sapiens	121.522	2	2	40418	0
8068	1	Q9NPJ3	Q9NPJ3	Acyl-coenzyme A thioesterase 13	ACOT13	Homo sapiens	14.958	2	2	2530117	404330
8069	1	Q9NPR9	Q9NPR9	Protein GPR108	GPR108	Homo sapiens	60.633	2	2	244662	133811
8070	1	Q9NQ30	Q9NQ30	Endothelial cell-specific molecule 1	ESM1	Homo sapiens	20.094	2	2	0	191234
8071	1	Q9NQB0	Q9NQB0	Transcription factor 7-like 2	TCF7L2	Homo sapiens	67.917	3	2	98620	0
8072	1	Q9NQC8	Q9NQC8	Intraflagellar transport protein 46 homolog	IFT46	Homo sapiens	34.283	2	2	123761	0
8073	1	Q9NQL2	Q9NQL2	Ras-related GTP-binding protein D	RRAGD	Homo sapiens	45.588	2	2	0	244466
8074	1	Q9NQP4	Q9NQP4	Prefoldin subunit 4	PFDN4	Homo sapiens	15.314	2	2	1285689	480575
8075	1	Q9NQZ2	Q9NQZ2	Something about silencing protein 10	UTP3	Homo sapiens	54.558	2	2	170356	0
8076	1	Q9NRA2	Q9NRA2	Sialin	SLC17A5	Homo sapiens	54.639	2	2	612266	1201769
8077	1	Q9NRG1	Q9NRG1	Phosphoribosyltransferase domain-containing protein 1	PRTFDC1	Homo sapiens	25.672	2	2	495014	93781
8078	1	Q9NRH3	Q9NRH3	Tubulin gamma-2 chain	TUBG2	Homo sapiens	51.092	2	2	104145	0
8079	1	Q9NRN9	Q9NRN9	rRNA N6-adenosine-methyltransferase METTL5	METTL5	Homo sapiens	23.719	2	2	167931	0
8080	1	Q9NRP2	Q9NRP2	COX assembly mitochondrial protein 2 homolog	CMC2	Homo sapiens	9.457	2	2	512584	0
8081	1	Q9NRR4	Q9NRR4	Ribonuclease 3	DROSHA	Homo sapiens	159.316	2	2	47059	0
8082	1	Q9NRW3	Q9NRW3	DNA dC->dU-editing enzyme APOBEC-3C	APOBEC3C	Homo sapiens	22.825	3	2	538097	87213
8083	1	Q9NRX3	Q9NRX3	NADH dehydrogenase [ubiquinone] 1 alpha subcomplex subunit 4-like 2	NDUFA4L2	Homo sapiens	9.965	2	2	174119	0
8084	1	Q9NRY6	Q9NRY6	Phospholipid scramblase 3	PLSCR3	Homo sapiens	31.649	2	2	0	184660
8085	1	Q9NRZ9	Q9NRZ9	Lymphoid-specific helicase	HELLS	Homo sapiens	97.077	2	2	95946	0
8086	1	Q9NS87	Q9NS87	Kinesin-like protein KIF15	KIF15	Homo sapiens	160.162	2	2	53006	30267
8087	1	Q9NSU2	Q9NSU2	Three-prime repair exonuclease 1	TREX1	Homo sapiens	33.209	2	2	143714	0
8088	1	Q9NSY2	Q9NSY2	StAR-related lipid transfer protein 5	STARD5	Homo sapiens	23.795	2	2	271667	33645

8089	1	Q9NU53	Q9NU53	Glycoprotein integral membrane protein 1	GINM1	Homo sapiens	36.842	2	2	49224	0
8090	1	Q9NUP7	Q9NUP7	tRNA:m(4)X modification enzyme TRM13 homolog	TRMT13	Homo sapiens	54.247	2	2	132302	0
8091	1	Q9NV66	Q9NV66	S-adenosyl-L-methionine-dependent tRNA 4-demethylwyosine synthase TYW1	TYW1	Homo sapiens	83.704	2	2	150886	0
8092	1	Q9NVC3	Q9NVC3	Sodium-coupled neutral amino acid transporter 7	SLC38A7	Homo sapiens	49.965	2	2	148341	188958
8093	1	Q9NVM4	Q9NVM4	Protein arginine N-methyltransferase 7	PRMT7	Homo sapiens	78.46	2	2	195507	37815
8094	1	Q9NVQ4	Q9NVQ4	Fas apoptotic inhibitory molecule 1	FAIM	Homo sapiens	20.214	2	2	162599	0
8095	1	Q9NVR5	Q9NVR5	Protein kintoun	DNAAF2	Homo sapiens	91.114	2	2	91535	0
8096	1	Q9NVV0	Q9NVV0	Trimeric intracellular cation channel type B	TMEM38B	Homo sapiens	32.508	2	2	309205	150104
8097	1	Q9NVV5	Q9NVV5	Androgen-induced gene 1 protein	AIG1	Homo sapiens	27.459	2	2	634451	934911
8098	1	Q9NW81	Q9NW81	Distal membrane-arm assembly complex protein 2	DMAC2	Homo sapiens	29.267	2	2	96581	29750
8099	1	Q9NWD8	Q9NWD8	Transmembrane protein 248	TMEM248	Homo sapiens	35.054	2	2	26744	0
8100	1	Q9NWW5	Q9NWW5	Ceroid-lipofuscinosis neuronal protein 6	CLN6	Homo sapiens	35.919	2	2	635800	248779
8101	1	Q9NX01	Q9NX01	Thioredoxin-like protein 4B	TXNL4B	Homo sapiens	17.013	2	2	18282	57507
8102	1	Q9NX14	Q9NX14	NADH dehydrogenase [ubiquinone] 1 beta subcomplex subunit 11, mitochondrial	NDUFB11	Homo sapiens	17.316	2	2	1378440	415248
8103	1	Q9NX95	Q9NX95	Syntabulin	SYBU	Homo sapiens	72.389	2	2	97112	0
8104	1	Q9NXU5	Q9NXU5	ADP-ribosylation factor-like protein 15	ARL15	Homo sapiens	22.875	2	2	685541	218728
8105	1	Q9NXV2	Q9NXV2	BTB/POZ domain-containing protein KCTD5	KCTD5	Homo sapiens	26.091	2	2	108097	61540
8106	1	Q9NY35	Q9NY35	Claudin domain-containing protein 1	CLDND1	Homo sapiens	28.605	2	2	17360	241438
8107	1	Q9NY65	Q9NY65	Tubulin alpha-8 chain	TUBA8	Homo sapiens	50.093	2	2	20019	134838
8108	1	Q9NYF3	Q9NYF3	Protein FAM53C	FAM53C	Homo sapiens	43.09	2	2	0	141880
8109	1	Q9NYG2	Q9NYG2	Palmitoyltransferase ZDHHC3	ZDHHC3	Homo sapiens	34.171	2	2	280226	196935
8110	1	Q9NYM9	Q9NYM9	BET1-like protein	BET1L	Homo sapiens	12.386	2	2	496211	0
8111	1	Q9NZ63	Q9NZ63	Splicing factor C9orf78	C9orf78	Homo sapiens	33.688	2	2	314599	27887
8112	1	Q9NZB8	Q9NZB8	Molybdenum cofactor biosynthesis protein 1	MOCS1	Homo sapiens	70.104	2	2	71086	0
8113	1	Q9NZI8	Q9NZI8	Insulin-like growth factor 2 mRNA-binding protein 1	IGF2BP1	Homo sapiens	63.482	2	2	21289	127794
8114	1	Q9NZJ4	Q9NZJ4	Sacsin	SACS	Homo sapiens	521.137	2	2	54411	0
8115	1	Q9NZJ5	Q9NZJ5	Eukaryotic translation initiation factor 2-alpha kinase 3	EIF2AK3	Homo sapiens	125.218	2	2	149549	87171
8116	1	Q9NZJ9	Q9NZJ9	Diphosphoinositol polyphosphate phosphohydrolase 2	NUDT4	Homo sapiens	20.306	6	2	357970	266808
8117	1	Q9NZV6	Q9NZV6	Methionine-R-sulfoxide reductase B1	MSRB1	Homo sapiens	12.731	2	2	109330	585516
8118	1	Q9P003	Q9P003	Protein cornichon homolog 4	CNIH4	Homo sapiens	16.094	2	2	0	866248
8119	1	Q9P031	Q9P031	Thyroid transcription factor 1-associated protein 26	CCDC59	Homo sapiens	28.669	2	2	63932	13428
8120	1	Q9P086	Q9P086	Mediator of RNA polymerase II transcription subunit 11	MED11	Homo sapiens	13.127	2	2	140618	0
8121	1	Q9P0J7	Q9P0J7	E3 ubiquitin-protein ligase KCMF1	KCMF1	Homo sapiens	41.947	2	2	247187	163152
8122	1	Q9P0T7	Q9P0T7	Proton-transporting V-type ATPase complex assembly regulator TMEM9	TMEM9	Homo sapiens	20.572	2	2	198499	115863
8123	1	Q9P0W2	Q9P0W2	SWI/SNF-related matrix-associated actin-dependent regulator of chromatin subfamily E member 1-related	HMG20B	Homo sapiens	35.812	2	2	218763	0
8124	1	Q9P0Z9	Q9P0Z9	Peroxisomal sarcosine oxidase	PIPOX	Homo sapiens	44.067	2	2	0	40569
8125	1	Q9P232	Q9P232	Contactin-3	CNTN3	Homo sapiens	112.884	2	2	16795	51095
8126	1	Q9P275	Q9P275	Ubiquitin carboxyl-terminal hydrolase 36	USP36	Homo sapiens	122.91	2	2	51425	0
8127	1	Q9P278	Q9P278	Folliculin-interacting protein 2	FNIP2	Homo sapiens	122.117	2	2	44099	0
8128	1	Q9P2C4	Q9P2C4	Transmembrane protein 181	TMEM181	Homo sapiens	69.324	2	2	924554	381651
8129	1	Q9P2M4	Q9P2M4	TBC1 domain family member 14	TBC1D14	Homo sapiens	78.138	2	2	50950	28839
8130	1	Q9P2X0	Q9P2X0	Dolichol-phosphate mannosyltransferase subunit 3	DPM3	Homo sapiens	10.092	2	2	107304	74687
8131	1	Q9UBI6	Q9UBI6	Guanine nucleotide-binding protein G(I)/G(S)/G(O) subunit gamma-12	GNG12	Homo sapiens	8.004	2	2	903200	94684
8132	1	Q9UBP9	Q9UBP9	PTB domain-containing engulfment adapter protein 1	GULP1	Homo sapiens	34.491	2	2	539299	0
8133	1	Q9UBV7	Q9UBV7	Beta-1,4-galactosyltransferase 7	B4GALT7	Homo sapiens	37.405	2	2	152135	86799

8134	1 Q9UBY0	Q9UBY0	Sodium/hydrogen exchanger 2	SLC9A2	Homo sapiens	91.523	2	2	756598	0
8135	1 Q9UDX5	Q9UDX5	Mitochondrial fission process protein 1	MTFP1	Homo sapiens	18.008	2	2	423997	41611
8136	1 Q9UER7	Q9UER7	Death domain-associated protein 6	DAXX	Homo sapiens	81.373	2	2	161463	58272
8137	1 Q9UFG5	Q9UFG5	UPF0449 protein C19orf25	C19orf25	Homo sapiens	12.874	2	2	46968	0
8138	1 Q9UG22	Q9UG22	GTPase IMAP family member 2	GIMAP2	Homo sapiens	38.015	2	2	157212	0
8139	1 Q9UG56	Q9UG56	Phosphatidylserine decarboxylase proenzyme, mitochondrial	PISD	Homo sapiens	46.67	2	2	360342	0
8140	1 Q9UHB4	Q9UHB4	NADPH-dependent diflavin oxidoreductase 1	NDOR1	Homo sapiens	66.762	2	2	46768	0
8141	1 Q9UHP9	Q9UHP9	Small muscular protein	SMPX	Homo sapiens	9.559	2	2	532363	0
8142	1 Q9UHR6	Q9UHR6	Zinc finger HIT domain-containing protein 2	ZNHIT2	Homo sapiens	42.883	2	2	46809	0
8143	1 Q9UHV5	Q9UHV5	Rap guanine nucleotide exchange factor-like 1	RAPGEFL1	Homo sapiens	73.267	2	2	101933	0
8144	1 Q9UI14	Q9UI14	Prenylated Rab acceptor protein 1	RABAC1	Homo sapiens	20.646	2	2	1212289	446783
8145	1 Q9UI36	Q9UI36	Dachshund homolog 1	DACH1	Homo sapiens	78.561	2	2	74860	0
8146	1 Q9UIF8	Q9UIF8	Bromodomain adjacent to zinc finger domain protein 2B	BAZ2B	Homo sapiens	240.461	2	2	41075	0
8147	1 Q9UIJ5	Q9UIJ5	Palmitoyltransferase ZDHHC2	ZDHHC2	Homo sapiens	42.022	2	2	15808	184723
8148	1 Q9UJ04	Q9UJ04	Testis-specific Y-encoded-like protein 4	TSPYL4	Homo sapiens	45.124	2	2	106228	0
8149	1 Q9UJ14	Q9UJ14	Glutathione hydrolase 7	GGT7	Homo sapiens	70.468	2	2	89686	0
8150	1 Q9UJA2	Q9UJA2	Cardiolipin synthase (CMP-forming)	CRLS1	Homo sapiens	32.594	2	2	68030	0
8151	1 Q9UK23	Q9UK23	N-acetylglucosamine-1-phosphodiester alpha-N-acetylglucosaminidase	NAGPA	Homo sapiens	56.072	2	2	126053	0
8152	1 Q9UK53	Q9UK53	Inhibitor of growth protein 1	ING1	Homo sapiens	46.737	2	2	123410	41668
8153	1 Q9UK58	Q9UK58	Cyclin-L1	CCNL1	Homo sapiens	59.633	2	2	147568	80035
8154	1 Q9UKA2	Q9UKA2	F-box/LRR-repeat protein 4	FBXL4	Homo sapiens	70.098	2	2	70738	13636
8155	1 Q9UKB1	Q9UKB1	F-box/WD repeat-containing protein 11	FBXW11	Homo sapiens	62.089	3	2	168055	110797
8156	1 Q9UKD1	Q9UKD1	Glucocorticoid modulatory element-binding protein 2	GMEB2	Homo sapiens	56.421	3	2	192752	198112
8157	1 Q9UKX5	Q9UKX5	Integrin alpha-11	ITGA11	Homo sapiens	133.471	2	2	90535	0
8158	1 Q9UKY0	Q9UKY0	Prion-like protein doppel	PRND	Homo sapiens	20.293	2	2	406801	0
8159	1 Q9UKY3	Q9UKY3	Putative inactive carboxylesterase 4	CES1P1	Homo sapiens	30.679	2	2	1919189	164793
8160	1 Q9UKY4	Q9UKY4	Protein O-mannosyl-transferase 2	POMT2	Homo sapiens	84.214	2	2	58319	0
8161	1 Q9ULD4	Q9ULD4	Bromodomain and PHD finger-containing protein 3	BRPF3	Homo sapiens	135.748	2	2	288871	0
8162	1 Q9ULJ3	Q9ULJ3	Zinc finger and BTB domain-containing protein 21	ZBTB21	Homo sapiens	118.871	2	2	18610	0
8163	1 Q9ULJ6	Q9ULJ6	Zinc finger MIZ domain-containing protein 1	ZMIZ1	Homo sapiens	115.485	3	2	214407	0
8164	1 Q9ULL5	Q9ULL5	Proline-rich protein 12	PRR12	Homo sapiens	211.05	2	2	97133	0
8165	1 Q9ULV5	Q9ULV5	Heat shock factor protein 4	HSF4	Homo sapiens	53.014	2	2	660225	0
8166	1 Q9ULY5	Q9ULY5	C-type lectin domain family 4 member E	CLEC4E	Homo sapiens	25.075	2	2	0	202938
8167	1 Q9UM00	Q9UM00	Calcium load-activated calcium channel	TMCO1	Homo sapiens	27.079	2	2	1540287	2602502
8168	1 Q9UM19	Q9UM19	Hippocalcin-like protein 4	HPCAL4	Homo sapiens	22.204	2	2	37286	0
8169	1 Q9UM21	Q9UM21	Alpha-1,3-mannosyl-glycoprotein 4-beta-N-acetylglucosaminyltransferase A	MGAT4A	Homo sapiens	61.546	2	2	36605	86850
8170	1 Q9UM44	Q9UM44	HERV-H LTR-associating protein 2	HHLA2	Homo sapiens	46.849	2	2	164960	0
8171	1 Q9UM47	Q9UM47	Neurogenic locus notch homolog protein 3	NOTCH3	Homo sapiens	243.637	2	2	50703	62373
8172	1 Q9UM73	Q9UM73	ALK tyrosine kinase receptor	ALK	Homo sapiens	176.443	2	2	13808	237470
8173	1 Q9UMR7	Q9UMR7	C-type lectin domain family 4 member A	CLEC4A	Homo sapiens	27.513	2	2	0	226086
8174	1 Q9UMX0	Q9UMX0	Ubiquilin-1	UBQLN1	Homo sapiens	62.52	2	2	323966	110508
8175	1 Q9UNA4	Q9UNA4	DNA polymerase iota	POLI	Homo sapiens	83.007	2	2	42628	0
8176	1 Q9UNL4	Q9UNL4	Inhibitor of growth protein 4	ING4	Homo sapiens	28.531	3	2	102732	0
8177	1 Q9UNS1	Q9UNS1	Protein timeless homolog	TIMELESS	Homo sapiens	138.662	2	2	33115	0
8178	1 Q9UNX3	Q9UNX3	60S ribosomal protein L26-like 1	RPL26L1	Homo sapiens	17.255	6	2	237863	202504
8179	1 Q9UP65	Q9UP65	Cytosolic phospholipase A2 gamma	PLA2G4C	Homo sapiens	60.94	2	2	118891	0
8180	1 Q9UPI3	Q9UPI3	Feline leukemia virus subgroup C receptor-related protein 2	FLVCR2	Homo sapiens	57.239	2	2	22228	247603

8181	1	Q9UPR3	Q9UPR3	Nonsense-mediated mRNA decay factor SMG5	SMG5	Homo sapiens	113.928	2	2	264339	48964
8182	1	Q9UPW0	Q9UPW0	Forkhead box protein J3	FOXJ3	Homo sapiens	68.96	2	2	670603	0
8183	1	Q9UPX0	Q9UPX0	Protein turtle homolog B	IGSF9B	Homo sapiens	147.09	2	2	74100	61720
8184	1	Q9UQ49	Q9UQ49	Sialidase-3	NEU3	Homo sapiens	48.253	2	2	277082	0
8185	1	Q9UQM7	Q9UQM7	Calcium/calmodulin-dependent protein kinase type II subunit alpha	CAMK2A	Homo sapiens	54.089	2	2	10261420	7648190
8186	1	Q9UQQ2	Q9UQQ2	SH2B adapter protein 3	SH2B3	Homo sapiens	63.225	2	2	31603	0
8187	1	Q9UQR0	Q9UQR0	Sex comb on midleg-like protein 2	SCML2	Homo sapiens	77.257	2	2	22633	49440
8188	1	Q9Y221	Q9Y221	60S ribosome subunit biogenesis protein NIP7 homolog	NIP7	Homo sapiens	20.463	2	2	282255	131730
8189	1	Q9Y227	Q9Y227	Ectonucleoside triphosphate diphosphohydrolase 4	ENTPD4	Homo sapiens	70.255	2	2	210334	1198969
8190	1	Q9Y248	Q9Y248	DNA replication complex GINS protein PSF2	GINS2	Homo sapiens	21.427	2	2	61994	16917
8191	1	Q9Y256	Q9Y256	CAAX prenyl protease 2	RCE1	Homo sapiens	35.832	2	2	144827	116702
8192	1	Q9Y294	Q9Y294	Histone chaperone ASF1A	ASF1A	Homo sapiens	22.967	2	2	235192	0
8193	1	Q9Y2D2	Q9Y2D2	UDP-N-acetylglucosamine transporter	SLC35A3	Homo sapiens	35.983	2	2	98181	23580
8194	1	Q9Y2R4	Q9Y2R4	Probable ATP-dependent RNA helicase DDX52	DDX52	Homo sapiens	67.466	2	2	160476	76148
8195	1	Q9Y2T7	Q9Y2T7	Y-box-binding protein 2	YBX2	Homo sapiens	38.517	2	2	125349	0
8196	1	Q9Y2Z2	Q9Y2Z2	Protein MTO1 homolog, mitochondrial	MTO1	Homo sapiens	79.967	2	2	56696	0
8197	1	Q9Y343	Q9Y343	Sorting nexin-24	SNX24	Homo sapiens	19.815	2	2	106728	30758
8198	1	Q9Y3A0	Q9Y3A0	Ubiquinone biosynthesis protein COQ4 homolog, mitochondrial	COQ4	Homo sapiens	29.657	2	2	13945	34096
8199	1	Q9Y3A4	Q9Y3A4	Ribosomal RNA-processing protein 7 homolog A	RRP7A	Homo sapiens	32.335	4	2	100868	44201
8200	1	Q9Y3B9	Q9Y3B9	RRP15-like protein	RRP15	Homo sapiens	31.484	2	2	164831	0
8201	1	Q9Y3C0	Q9Y3C0	WASH complex subunit 3	WASHC3	Homo sapiens	21.169	2	2	734347	568520
8202	1	Q9Y3C5	Q9Y3C5	RING finger protein 11	RNF11	Homo sapiens	17.443	2	2	95577	0
8203	1	Q9Y3D0	Q9Y3D0	Cytosolic iron-sulfur assembly component 2B	CIAO2B	Homo sapiens	17.664	2	2	518113	117176
8204	1	Q9Y3D5	Q9Y3D5	28S ribosomal protein S18c, mitochondrial	MRPS18C	Homo sapiens	15.851	2	2	475202	51686
8205	1	Q9Y3D7	Q9Y3D7	Mitochondrial import inner membrane translocase subunit TIM16	PAM16	Homo sapiens	13.823	2	2	586321	222180
8206	1	Q9Y3E0	Q9Y3E0	Vesicle transport protein GOT1B	GOLT1B	Homo sapiens	15.422	2	2	175393	222803
8207	1	Q9Y3L3	Q9Y3L3	SH3 domain-binding protein 1	SH3BP1	Homo sapiens	75.713	18	2	455319	357047
8208	1	Q9Y3M2	Q9Y3M2	Protein chibby homolog 1	CBY1	Homo sapiens	14.469	2	2	260612	0
8209	1	Q9Y3S2	Q9Y3S2	Zinc finger protein 330	ZNF330	Homo sapiens	36.203	2	2	39830	654748
8210	1	Q9Y4B4	Q9Y4B4	Helicase ARIP4	RAD54L2	Homo sapiens	162.771	2	2	42766	0
8211	1	Q9Y4C4	Q9Y4C4	Malignant fibrous histiocytoma-amplified sequence 1	MFHAS1	Homo sapiens	116.952	2	2	142043	0
8212	1	Q9Y4D2	Q9Y4D2	Diacylglycerol lipase-alpha	DAGLA	Homo sapiens	114.951	2	2	88170	0
8213	1	Q9Y4H4	Q9Y4H4	G-protein-signaling modulator 3	GPSM3	Homo sapiens	17.862	2	2	0	1197504
8214	1	Q9Y586	Q9Y586	Protein mab-21-like 2	MAB21L2	Homo sapiens	40.923	5	2	599317	0
8215	1	Q9Y587	Q9Y587	AP-4 complex subunit sigma-1	AP4S1	Homo sapiens	17.005	2	2	233054	306054
8216	1	Q9Y5C1	Q9Y5C1	Angiopoietin-related protein 3	ANGPTL3	Homo sapiens	53.639	2	2	0	178309
8217	1	Q9Y5J7	Q9Y5J7	Mitochondrial import inner membrane translocase subunit Tim9	TIMM9	Homo sapiens	10.376	2	2	251855	98947
8218	1	Q9Y5L3	Q9Y5L3	Ectonucleoside triphosphate diphosphohydrolase 2	ENTPD2	Homo sapiens	53.668	2	2	438822	0
8219	1	Q9Y5Y0	Q9Y5Y0	Feline leukemia virus subgroup C receptor-related protein 1	FLVCR1	Homo sapiens	59.863	2	2	113032	0
8220	1	Q9Y5Y2	Q9Y5Y2	Cytosolic Fe-S cluster assembly factor NUBP2	NUBP2	Homo sapiens	28.824	2	2	220591	193459
8221	1	Q9Y5Y5	Q9Y5Y5	Peroxisomal membrane protein PEX16	PEX16	Homo sapiens	38.628	2	2	206743	148112
8222	1	Q9Y605	Q9Y605	MORF4 family-associated protein 1	MRFAP1	Homo sapiens	14.648	2	2	155218	0
8223	1	Q9Y6A4	Q9Y6A4	Cilia- and flagella-associated protein 20	CFAP20	Homo sapiens	22.773	2	2	569647	77628
8224	1	Q9Y6K1	Q9Y6K1	DNA (cytosine-5)-methyltransferase 3A	DNMT3A	Homo sapiens	101.858	2	2	58118	11562
8225	1	Q9Y6K8	Q9Y6K8	Adenylate kinase isoenzyme 5	AK5	Homo sapiens	63.334	2	2	24203	55013
8226	1	Q9Y6M4	Q9Y6M4	Casein kinase I isoform gamma-3	CSNK1G3	Homo sapiens	51.389	2	2	226558	20018
8227	1	Q9Y6P5	Q9Y6P5	Sestrin-1	SESN1	Homo sapiens	56.557	3	2	211509	0
8228	1	Q9Y6Q9	Q9Y6Q9	Nuclear receptor coactivator 3	NCOA3	Homo sapiens	155.295	2	2	282367	0

8229	1	Q9Y6R9	Q9Y6R9	Centrosomal protein CCDC61	CCDC61	Homo sapiens	57.369	2	2	49039	0
8230	1	Q9Y6V0	Q9Y6V0	Protein piccolo	PCLO	Homo sapiens	560.712	2	2	67746	0
8231	1	Q9Y6X8	Q9Y6X8	Zinc fingers and homeoboxes protein 2	ZHX2	Homo sapiens	92.309	2	2	144704	0
8232	1	A0A075B6I1	A0A075B6I1	Immunoglobulin lambda variable 4-60	IGLV4-60	Homo sapiens	12.986	1	1	678743	929425
8233	1	A0A075B6I9	A0A075B6I9	Immunoglobulin lambda variable 7-46	IGLV7-46	Homo sapiens	12.468	1	1	1366230	1454320
8234	1	A0A075B6J1	A0A075B6J1	Immunoglobulin lambda variable 5-37	IGLV5-37	Homo sapiens	13.277	1	1	64646	133367
8235	1	A0A075B6K0	A0A075B6K0	Immunoglobulin lambda variable 3-16	IGLV3-16	Homo sapiens	12.464	2	1	103306	200424
8236	1	A0A075B6K2	A0A075B6K2	Immunoglobulin lambda variable 3-12	IGLV3-12	Homo sapiens	12.386	1	1	0	209107
8237	1	A0A075B6K5	A0A075B6K5	Immunoglobulin lambda variable 3-9	IGLV3-9	Homo sapiens	12.331	3	1	31613	82524
8238	1	A0A075B6Q5	A0A075B6Q5	Immunoglobulin heavy variable 3-64	IGHV3-64	Homo sapiens	12.888	2	1	387254	378773
8239	2	A0A075B6S2;A2N	A0A075B6S2	Immunoglobulin kappa variable 2D-29	IGKV2D-29	Homo sapiens	13.141	2	1	412843	754870
8240	2	A0A075B6S9;P0D	A0A075B6S9	Probable non-functional immunoglobulinn kappa variable 1-37	IGKV1-37	Homo sapiens	12.684	1	1	1341998	1553192
8241	1	A0A075B7B8	A0A075B7B8	Immunoglobulin heavy variable 3/OR16-12 (non-functional) (Fragment)	IGHV3OR16-12	Homo sapiens	12.875	1	1	1705900	1677920
8242	1	A0A087WSY4	A0A087WSY4	Immunoglobulin heavy variable 4-30-2	IGHV4-30-2	Homo sapiens	13.024	1	1	0	57268
8243	1	A0A087WSY6	A0A087WSY6	Immunoglobulin kappa variable 3D-15	IGKV3D-15	Homo sapiens	12.533	1	1	3396480	3156620
8244	2	A0A087WUL8;Q6I	A0A087WUL8	Neuroblastoma breakpoint family member 19	NBPF19	Homo sapiens	440.419	1	1	296852	510186
8245	2	A0A0A0MRZ8;P04	A0A0A0MRZ8	Immunoglobulin kappa variable 3D-11	IGKV3D-11	Homo sapiens	12.624	1	1	14603300	42155200
8246	1	A0A0A0MS14	A0A0A0MS14	Immunoglobulin heavy variable 1-45	IGHV1-45	Homo sapiens	13.505	1	1	74124	137853
8247	2	A0A0A0MT36;A0A	A0A0A0MT36	Immunoglobulin kappa variable 6D-21	IGKV6D-21	Homo sapiens	12.339	1	1	1333560	2473710
8248	1	A0A0A0MT96	A0A0A0MT96	Immunoglobulin kappa joining 3 (Fragment)	IGKJ3	Homo sapiens	1.307	1	1	0	139076
8249	1	A0A0B4J1U3	A0A0B4J1U3	Immunoglobulin lambda variable 1-36	IGLV1-36	Homo sapiens	12.479	1	1	23553	17550
8250	1	A0A0B4J1V2	A0A0B4J1V2	Immunoglobulin heavy variable 2-26	IGHV2-26	Homo sapiens	13.18	1	1	58222	87068
8251	1	A0A0B4J1X5	A0A0B4J1X5	Immunoglobulin heavy variable 3-74	IGHV3-74	Homo sapiens	12.838	4	1	257779	854711
8252	1	A0A0B4J1X8	A0A0B4J1X8	Immunoglobulin heavy variable 3-43	IGHV3-43	Homo sapiens	13.077	3	1	241879	443480
8253	1	A0A0B4J2B5	A0A0B4J2B5	Immunoglobulin heavy variable 3/OR16-9 (non-functional) (Fragment)	IGHV3OR16-9	Homo sapiens	10.656	1	1	5380850	5499500
8254	1	A0A0B4J2B8	A0A0B4J2B8	Immunoglobulin heavy variable 1/OR15-9 (non-functional) (Fragment)	IGHV1OR15-9	Homo sapiens	12.953	1	1	22513	0
8255	2	A0A0B4J2D9;P0D	A0A0B4J2D9	Immunoglobulin kappa variable 1D-13	IGKV1D-13	Homo sapiens	12.567	1	1	0	137502
8256	1	A0A0C4DH24	A0A0C4DH24	Immunoglobulin kappa variable 6-21	IGKV6-21	Homo sapiens	12.428	2	1	961877	1682320
8257	1	A0A0C4DH25	A0A0C4DH25	Immunoglobulin kappa variable 3D-20	IGKV3D-20	Homo sapiens	12.513	3	1	6076210	8242560
8258	1	A0A0C4DH32	A0A0C4DH32	Immunoglobulin heavy variable 3-20	IGHV3-20	Homo sapiens	12.673	1	1	23231	19855
8259	1	A0A0C4DH34	A0A0C4DH34	Immunoglobulin heavy variable 4-28	IGHV4-28	Homo sapiens	13.121	1	1	153802	246322
8260	1	A0A0C4DH35	A0A0C4DH35	Probable non-functional immunoglobulin heavy variable 3-35	IGHV3-35	Homo sapiens	12.81	1	1	765927	1156160
8261	1	A0A0C4DH39	A0A0C4DH39	Immunoglobulin heavy variable 1-58	IGHV1-58	Homo sapiens	13.003	1	1	86004	73933
8262	1	A0A0C4DH67	A0A0C4DH67	Immunoglobulin kappa variable 1-8	IGKV1-8	Homo sapiens	12.535	2	1	250458	194635
8263	1	A0A0G2JMI3	A0A0G2JMI3	Immunoglobulin heavy variable 1-69-2	IGHV1-69-2	Homo sapiens	12.87	1	1	31865	25096
8264	1	A0A0J9YX35	A0A0J9YX35	Immunoglobulin heavy variable 3-64D	IGHV3-64D	Homo sapiens	12.822	2	1	2489290	2478390
8265	1	A0A0J9YY99	A0A0J9YY99	Ig-like domain-containing protein (Fragment)	-	Homo sapiens	12.962	1	1	130769	128930
8266	1	A0A0U1RQS6	A0A0U1RQS6	Transmembrane protein 275	TMEM275	Homo sapiens	17.234	1	1	87112	0
8267	3	A0A5B9;P01850;P	A0A5B9	T cell receptor beta constant 2	TRBC2	Homo sapiens	19.967	1	1	26037	0
8268	1	A0AV02	A0AV02	Solute carrier family 12 member 8	SLC12A8	Homo sapiens	78.239	1	1	17878	0
8269	1	A0AVI2	A0AVI2	Fer-1-like protein 5	FER1L5	Homo sapiens	237.939	1	1	59665	0
8270	1	A0AVI4	A0AVI4	E3 ubiquitin-protein ligase TM129	TMEM129	Homo sapiens	40.464	1	1	45665	0
8271	1	A0M8Q6	A0M8Q6	Immunoglobulin lambda constant 7	IGLC7	Homo sapiens	11.251	4	1	143678	278997
8272	1	A0PK05	A0PK05	Transmembrane protein 72	TMEM72	Homo sapiens	29.892	1	1	88895	0
8273	1	A1XBS5	A1XBS5	CBY1-interacting BAR domain-containing protein 1	CIBAR1	Homo sapiens	33.429	1	1	34967	0
8274	1	A2A3K4	A2A3K4	Protein tyrosine phosphatase domain-containing protein 1	PTPDC1	Homo sapiens	84.459	1	1	9284	0
8275	1	A2RUB1	A2RUB1	Meiosis-specific coiled-coil domain-containing protein MEIOC	MEIOC	Homo sapiens	107.56	1	1	17711	53349
8276	1	A3QJZ6	A3QJZ6	PRAME family member 22	PRAMEF22	Homo sapiens	55.49	1	1	0	70404

8277	1	A6NC57	A6NC57	Ankyrin repeat domain-containing protein 62	ANKRD62	Homo sapiens	106.448	1	1	22319	0
8278	3	A6NDR6;A8K0S8;	A6NDR6	Putative homeobox protein Meis3-like 1	MEIS3P1	Homo sapiens	30.202	1	1	37848	0
8279	4	A6NEL3;P0C5J1;C	A6NEL3	Putative protein FAM86C2P	FAM86C2P	Homo sapiens	18.476	1	1	30119	0
8280	1	A6NFI3	A6NFI3	Zinc finger protein 316	ZNF316	Homo sapiens	108.439	1	1	62671	0
8281	1	A6NFR6	A6NFR6	Uncharacterized protein C5orf60	C5orf60	Homo sapiens	39.25	1	1	203683	0
8282	1	A6NGC4	A6NGC4	TLC domain-containing protein 2	TLCD2	Homo sapiens	28.734	1	1	96709	0
8283	1	A6NK58	A6NK58	Putative lipoyltransferase 2, mitochondrial	LIPT2	Homo sapiens	25.197	1	1	47965	0
8284	1	A8CG34	A8CG34	Nuclear envelope pore membrane protein POM 121C	POM121C	Homo sapiens	125.094	9	1	51538	0
8285	1	A8MSI8	A8MSI8	LYR motif-containing protein 9	LYRM9	Homo sapiens	9.37	1	1	79169	0
8286	1	A8MTT3	A8MTT3	Protein CEBPZOS	CEBPZOS	Homo sapiens	9.334	1	1	84676	0
8287	1	A8MW92	A8MW92	PHD finger protein 20-like protein 1	PHF20L1	Homo sapiens	115.011	1	1	60562	0
8288	2	A8MYZ6;Q12778	A8MYZ6	Forkhead box protein O6	FOXO6	Homo sapiens	50.595	1	1	20016	8592
8289	1	B2RBV5	B2RBV5	Putative MORF4 family-associated protein 1-like protein UPP	-	Homo sapiens	13.367	1	1	10269	0
8290	2	B3KU38;P0DPB3	B3KU38	IQCJ-SCHIP1 readthrough transcript protein	IQCJ-SCHIP1	Homo sapiens	62.25	1	1	80165	0
8291	1	C9JI98	C9JI98	Transmembrane protein 238	TMEM238	Homo sapiens	18.041	1	1	235867	0
8292	1	C9JQL5	C9JQL5	Putative dispanin subfamily A member 2d	-	Homo sapiens	14.796	1	1	0	1084780
8293	1	C9JTQ0	C9JTQ0	Ankyrin repeat domain-containing protein 63	ANKRD63	Homo sapiens	39.619	1	1	64369	0
8294	1	F5H4A9	F5H4A9	Uncharacterized membrane protein C3orf80	C3orf80	Homo sapiens	25.681	1	1	19665	0
8295	1	O00167	O00167	Eyes absent homolog 2	EYA2	Homo sapiens	59.233	1	1	0	48504
8296	1	O00168	O00168	Phospholemman	FXYD1	Homo sapiens	10.44	1	1	789595	29442
8297	1	O00204	O00204	Sulfotransferase 2B1	SULT2B1	Homo sapiens	41.307	1	1	0	38799
8298	1	O00258	O00258	Guided entry of tail-anchored proteins factor 1	GET1	Homo sapiens	19.779	1	1	0	100122
8299	1	O00292	O00292	Left-right determination factor 2	LEFTY2	Homo sapiens	40.92	1	1	0	122602
8300	1	O00458	O00458	Interferon-related developmental regulator 1	IFRD1	Homo sapiens	50.268	1	1	19657	0
8301	1	O00507	O00507	Probable ubiquitin carboxyl-terminal hydrolase FAF-Y	USP9Y	Homo sapiens	291.083	1	1	72437	35898
8302	1	O00559	O00559	Receptor-binding cancer antigen expressed on SiSo cells	EBAG9	Homo sapiens	24.376	1	1	61876	14374
8303	1	O00757	O00757	Fructose-1,6-bisphosphatase isozyme 2	FBP2	Homo sapiens	36.741	1	1	1488510	1116250
8304	1	O00767	O00767	Stearoyl-CoA desaturase	SCD	Homo sapiens	41.521	1	1	141914	193094
8305	1	O14493	O14493	Claudin-4	CLDN4	Homo sapiens	22.075	1	1	0	13651
8306	1	O14519	O14519	Cyclin-dependent kinase 2-associated protein 1	CDK2AP1	Homo sapiens	12.364	3	1	39282	0
8307	1	O14569	O14569	Transmembrane reductase CYB561D2	CYB561D2	Homo sapiens	23.973	1	1	55527	115982
8308	1	O14595	O14595	Carboxy-terminal domain RNA polymerase II polypeptide A small phosphatase 2	CTDSP2	Homo sapiens	30.665	1	1	88120	0
8309	1	O14684	O14684	Prostaglandin E synthase	PTGES	Homo sapiens	17.101	1	1	655944	461279
8310	1	O14715	O14715	RANBP2-like and GRIP domain-containing protein 8	RGPD8	Homo sapiens	198.996	3	1	0	72804
8311	1	O14798	O14798	Tumor necrosis factor receptor superfamily member 10C	TNFRSF10C	Homo sapiens	27.407	1	1	0	168338
8312	1	O14893	O14893	Gem-associated protein 2	GEMIN2	Homo sapiens	31.584	1	1	92022	0
8313	1	O14925	O14925	Mitochondrial import inner membrane translocase subunit Tim23	TIMM23	Homo sapiens	21.943	2	1	435962	162175
8314	1	O14948	O14948	Transcription factor EC	TFEC	Homo sapiens	38.786	1	1	33290	0
8315	1	O14957	O14957	Cytochrome b-c1 complex subunit 10	UQCR11	Homo sapiens	6.567	1	1	150394	26890
8316	1	O15063	O15063	Granule associated Rac and RHOG effector protein 1	GARRE1	Homo sapiens	116.022	1	1	17725	0
8317	1	O15131	O15131	Importin subunit alpha-6	KPNA5	Homo sapiens	60.667	1	1	26530	0
8318	1	O15155	O15155	BET1 homolog	BET1	Homo sapiens	13.288	1	1	311510	259316
8319	1	O15198	O15198	Mothers against decapentaplegic homolog 9	SMAD9	Homo sapiens	52.493	1	1	23080	0
8320	1	O15239	O15239	NADH dehydrogenase [ubiquinone] 1 alpha subcomplex subunit 1	NDUFA1	Homo sapiens	8.07	1	1	404429	0
8321	1	O15243	O15243	Leptin receptor gene-related protein	LEPROT	Homo sapiens	14.253	1	1	47258	43205
8322	1	O15258	O15258	Protein RER1	RER1	Homo sapiens	22.958	1	1	1884130	966168
8323	1	O15263	O15263	Defensin beta 4A	DEFB4B	Homo sapiens	7.036	1	1	0	277314

8324	1	O15297	O15297	Protein phosphatase 1D	PPM1D	Homo sapiens	66.676	1	1	11623	0
8325	1	O15304	O15304	Apoptosis regulatory protein Siva	SIVA1	Homo sapiens	18.692	1	1	9785	0
8326	1	O15391	O15391	Transcription factor YY2	YY2	Homo sapiens	41.346	1	1	71355	54286
8327	1	O15393	O15393	Transmembrane protease serine 2	TMPRSS2	Homo sapiens	53.86	1	1	28765	0
8328	1	O15405	O15405	TOX high mobility group box family member 3	TOX3	Homo sapiens	63.342	1	1	17013	0
8329	1	O15516	O15516	Circadian locomoter output cycles protein kaput	CLOCK	Homo sapiens	95.303	1	1	20958	0
8330	1	O15550	O15550	Lysine-specific demethylase 6A	KDM6A	Homo sapiens	154.177	2	1	38632	0
8331	1	O15552	O15552	Free fatty acid receptor 2	FFAR2	Homo sapiens	37.144	1	1	0	208787
8332	1	O15554	O15554	Intermediate conductance calcium-activated potassium channel protein 4	KCNN4	Homo sapiens	47.695	1	1	31124	0
8333	1	O43194	O43194	G-protein coupled receptor 39	GPR39	Homo sapiens	51.33	1	1	56049	0
8334	1	O43251	O43251	RNA binding protein fox-1 homolog 2	RBFOX2	Homo sapiens	41.374	3	1	75331	0
8335	1	O43257	O43257	Zinc finger HIT domain-containing protein 1	ZNHIT1	Homo sapiens	17.534	1	1	20359	0
8336	1	O43374	O43374	Ras GTPase-activating protein 4	RASA4	Homo sapiens	90.458	8	1	83679	0
8337	1	O43583	O43583	Density-regulated protein	DENR	Homo sapiens	22.089	1	1	0	124359
8338	1	O43715	O43715	TP53-regulated inhibitor of apoptosis 1	TRIAP1	Homo sapiens	8.783	1	1	119568	0
8339	1	O43716	O43716	Glutamyl-tRNA(Gln) amidotransferase subunit C, mitochondrial	GATC	Homo sapiens	15.085	1	1	58325	0
8340	1	O43731	O43731	ER lumen protein-retaining receptor 3	KDELR3	Homo sapiens	25.028	1	1	104959	58313
8341	1	O43914	O43914	TYRO protein tyrosine kinase-binding protein	TYROBP	Homo sapiens	12.178	1	1	0	333228
8342	1	O60232	O60232	Protein ZNRD2	ZNRD2	Homo sapiens	21.473	1	1	331321	54184
8343	1	O60238	O60238	BCL2/adenovirus E1B 19 kDa protein-interacting protein 3-like	BNIP3L	Homo sapiens	23.929	2	1	171201	80943
8344	1	O60291	O60291	E3 ubiquitin-protein ligase MGRN1	MGRN1	Homo sapiens	60.754	1	1	33363	0
8345	1	O60296	O60296	Trafficking kinesin-binding protein 2	TRAK2	Homo sapiens	101.42	1	1	22583	0
8346	1	O60356	O60356	Nuclear protein 1	NUPR1	Homo sapiens	8.871	1	1	329536	0
8347	1	O60512	O60512	Beta-1,4-galactosyltransferase 3	B4GALT3	Homo sapiens	43.928	1	1	51199	0
8348	1	O60635	O60635	Tetraspanin-1	TSPAN1	Homo sapiens	26.301	1	1	320295	99616
8349	1	O60671	O60671	Cell cycle checkpoint protein RAD1	RAD1	Homo sapiens	31.829	1	1	219842	83731
8350	1	O60704	O60704	Protein-tyrosine sulfotransferase 2	TPST2	Homo sapiens	41.913	1	1	72476	180675
8351	1	O60760	O60760	Hematopoietic prostaglandin D synthase	HPGDS	Homo sapiens	23.341	1	1	997341	0
8352	1	O60828	O60828	Polyglutamine-binding protein 1	PQBP1	Homo sapiens	30.473	1	1	680241	102855
8353	1	O60880	O60880	SH2 domain-containing protein 1A	SH2D1A	Homo sapiens	14.187	1	1	28421	0
8354	1	O60906	O60906	Sphingomyelin phosphodiesterase 2	SMPD2	Homo sapiens	47.645	1	1	70491	34779
8355	1	O60921	O60921	Checkpoint protein HUS1	HUS1	Homo sapiens	31.691	1	1	156429	105945
8356	1	O60941	O60941	Dystrobrevin beta	DTNB	Homo sapiens	71.357	1	1	8909	0
8357	1	O75015	O75015	Low affinity immunoglobulin gamma Fc region receptor III-B	FCGR3B	Homo sapiens	26.214	1	1	69472	1728421
8358	1	O75084	O75084	Frizzled-7	FZD7	Homo sapiens	63.621	2	1	67487	0
8359	1	O75093	O75093	Slit homolog 1 protein	SLIT1	Homo sapiens	167.929	1	1	0	100262
8360	1	O75197	O75197	Low-density lipoprotein receptor-related protein 5	LRP5	Homo sapiens	179.147	1	1	23262	0
8361	1	O75264	O75264	Small integral membrane protein 24	SMIM24	Homo sapiens	14.962	1	1	0	93897
8362	1	O75317	O75317	Ubiquitin carboxyl-terminal hydrolase 12	USP12	Homo sapiens	42.858	2	1	77097	0
8363	1	O75319	O75319	RNA/RNP complex-1-interacting phosphatase	DUSP11	Homo sapiens	43.71	1	1	34081	0
8364	1	O75366	O75366	Advillin	AVIL	Homo sapiens	92.027	1	1	11721	0
8365	1	O75438	O75438	NADH dehydrogenase [ubiquinone] 1 beta subcomplex subunit 1	NDUFB1	Homo sapiens	6.958	1	1	463323	186133
8366	1	O75446	O75446	Histone deacetylase complex subunit SAP30	SAP30	Homo sapiens	23.304	1	1	34198	0
8367	1	O75467	O75467	Zinc finger protein 324A	ZNF324	Homo sapiens	61.105	2	1	20330	0
8368	1	O75496	O75496	Geminin	GMNN	Homo sapiens	23.567	1	1	54553	0
8369	1	O75553	O75553	Disabled homolog 1	DAB1	Homo sapiens	63.776	1	1	0	53586
8370	1	O75570	O75570	Peptide chain release factor 1, mitochondrial	MTRF1	Homo sapiens	52.306	1	1	245447	0
8371	1	O75575	O75575	DNA-directed RNA polymerase III subunit RPC9	CRCP	Homo sapiens	16.871	1	1	29291	0

8372	2	O75795;P54855	O75795	UDP-glucuronosyltransferase 2B17	UGT2B17	Homo sapiens	61.095	1	1	0	83591
8373	1	O75896	O75896	Tumor suppressor candidate 2	TUSC2	Homo sapiens	12.072	1	1	0	30262
8374	1	O75900	O75900	Matrix metalloproteinase-23	MMP23B	Homo sapiens	43.935	1	1	33939	0
8375	1	O75925	O75925	E3 SUMO-protein ligase PIAS1	PIAS1	Homo sapiens	71.835	1	1	38157	16716
8376	1	O76095	O76095	Protein JTB	JTB	Homo sapiens	16.358	1	1	504139	408853
8377	1	O76096	O76096	Cystatin-F	CST7	Homo sapiens	16.456	1	1	56005	460054
8378	1	O94772	O94772	Lymphocyte antigen 6H	LY6H	Homo sapiens	14.669	1	1	21029	0
8379	1	O94782	O94782	Ubiquitin carboxyl-terminal hydrolase 1	USP1	Homo sapiens	88.211	1	1	22103	0
8380	1	O94817	O94817	Ubiquitin-like protein ATG12	ATG12	Homo sapiens	15.111	1	1	19523	0
8381	1	O94864	O94864	STAGA complex 65 subunit gamma	SUPT7L	Homo sapiens	46.194	1	1	67472	0
8382	1	O94900	O94900	Thymocyte selection-associated high mobility group box protein TOX	TOX	Homo sapiens	57.512	1	1	81935	0
8383	1	O94921	O94921	Cyclin-dependent kinase 14	CDK14	Homo sapiens	53.054	2	1	45868	44861
8384	1	O94953	O94953	Lysine-specific demethylase 4B	KDM4B	Homo sapiens	121.897	2	1	22861	0
8385	1	O94955	O94955	Rho-related BTB domain-containing protein 3	RHOBTB3	Homo sapiens	69.412	1	1	42565	0
8386	1	O95059	O95059	Ribonuclease P protein subunit p14	RPP14	Homo sapiens	13.692	1	1	10181	0
8387	1	O95136	O95136	Sphingosine 1-phosphate receptor 2	S1PR2	Homo sapiens	38.866	1	1	162859	0
8388	1	O95183	O95183	Vesicle-associated membrane protein 5	VAMP5	Homo sapiens	12.803	1	1	106635	106641
8389	1	O95239	O95239	Chromosome-associated kinesin KIF4A	KIF4A	Homo sapiens	139.883	2	1	103090	0
8390	1	O95273	O95273	Cyclin-D1-binding protein 1	CCNDBP1	Homo sapiens	40.262	1	1	1912578	43919
8391	1	O95274	O95274	Ly6/PLAUR domain-containing protein 3	LYPD3	Homo sapiens	35.968	1	1	0	42734
8392	1	O95405	O95405	Zinc finger FYVE domain-containing protein 9	ZFYVE9	Homo sapiens	156.404	1	1	4157	0
8393	1	O95415	O95415	Brain protein I3	BRI3	Homo sapiens	13.642	1	1	45747	81856
8394	1	O95471	O95471	Claudin-7	CLDN7	Homo sapiens	22.418	1	1	95659	0
8395	1	O95562	O95562	Vesicle transport protein SFT2B	SFT2D2	Homo sapiens	17.775	1	1	462043	282846
8396	1	O95772	O95772	STARD3 N-terminal-like protein	STARD3NL	Homo sapiens	26.657	1	1	295154	223790
8397	1	O95833	O95833	Chloride intracellular channel protein 3	CLIC3	Homo sapiens	26.648	1	1	0	99037
8398	1	O95838	O95838	Glucagon-like peptide 2 receptor	GLP2R	Homo sapiens	62.999	1	1	72364	0
8399	1	O95905	O95905	Protein ecdysoneless homolog	ECD	Homo sapiens	72.759	1	1	63101	0
8400	1	O95931	O95931	Chromobox protein homolog 7	CBX7	Homo sapiens	28.341	1	1	172749	0
8401	1	P00156	P00156	Cytochrome b	MT-CYB	Homo sapiens	42.716	1	1	1012340	130367
8402	1	P00395	P00395	Cytochrome c oxidase subunit 1	MT-CO1	Homo sapiens	57.042	1	1	13004854	0
8403	1	P00709	P00709	Alpha-lactalbumin	LALBA	Homo sapiens	16.224	1	1	299844	572047
8404	1	P00973	P00973	2'-5'-oligoadenylate synthase 1	OAS1	Homo sapiens	46.03	1	1	200061	70332
8405	2	P01593;P01594	P01593	Immunoglobulin kappa variable 1D-33	IGKV1D-33	Homo sapiens	12.848	1	1	45849	51634
8406	1	P01601	P01601	Immunoglobulin kappa variable 1D-16	IGKV1D-16	Homo sapiens	12.731	1	1	1127237	691704
8407	1	P01700	P01700	Immunoglobulin lambda variable 1-47	IGLV1-47	Homo sapiens	12.283	3	1	13081200	16513800
8408	1	P01704	P01704	Immunoglobulin lambda variable 2-14	IGLV2-14	Homo sapiens	12.596	1	1	10202200	12876500
8409	2	P01706;P01709	P01706	Immunoglobulin lambda variable 2-11	IGLV2-11	Homo sapiens	12.643	1	1	25844	27724
8410	1	P01718	P01718	Immunoglobulin lambda variable 3-27	IGLV3-27	Homo sapiens	12.163	1	1	110689	294872
8411	1	P01742	P01742	Immunoglobulin heavy variable 1-69	IGHV1-69	Homo sapiens	12.658	2	1	551993	709527
8412	1	P01780	P01780	Immunoglobulin heavy variable 3-7	IGHV3-7	Homo sapiens	12.941	1	1	14914600	25994800
8413	1	P01854	P01854	Immunoglobulin heavy constant epsilon	IGHE	Homo sapiens	47.017	1	1	246697	0
8414	1	P01893	P01893	Putative HLA class I histocompatibility antigen, alpha chain H	HLA-H	Homo sapiens	40.891	3	1	157991	531749
8415	1	P02008	P02008	Hemoglobin subunit zeta	HBZ	Homo sapiens	15.635	1	1	0	90679
8416	1	P02100	P02100	Hemoglobin subunit epsilon	HBE1	Homo sapiens	16.203	1	1	0	161847
8417	1	P02776	P02776	Platelet factor 4	PF4	Homo sapiens	10.846	3	1	0	238970
8418	1	P03886	P03886	NADH-ubiquinone oxidoreductase chain 1	MT-ND1	Homo sapiens	35.66	1	1	90077	23092
8419	1	P03891	P03891	NADH-ubiquinone oxidoreductase chain 2	MT-ND2	Homo sapiens	38.962	1	1	350314	56572

8420	1	P03897	P03897	NADH-ubiquinone oxidoreductase chain 3	MT-ND3	Homo sapiens	13.184	1	1	527858	101425
8421	1	P03923	P03923	NADH-ubiquinone oxidoreductase chain 6	MT-ND6	Homo sapiens	18.619	1	1	242575	0
8422	1	P04430	P04430	Immunoglobulin kappa variable 1-16	IGKV1-16	Homo sapiens	12.618	1	1	2509261	4254724
8423	1	P04439	P04439	HLA class I histocompatibility antigen, A alpha chain	HLA-A	Homo sapiens	40.84	1	1	1843006	1246720
8424	3	P04908;Q7L7L0;Q	P04908	Histone H2A type 1-B/E	H2AC8	Homo sapiens	14.136	4	1	90193	0
8425	1	P04921	P04921	Glycophorin-C	GYPC	Homo sapiens	13.81	1	1	82967	0
8426	1	P05204	P05204	Non-histone chromosomal protein HMG-17	HMGN2	Homo sapiens	9.39	1	1	430802	352484
8427	1	P05230	P05230	Fibroblast growth factor 1	FGF1	Homo sapiens	17.458	1	1	31028	0
8428	1	P05386	P05386	60S acidic ribosomal protein P1	RPLP1	Homo sapiens	11.513	1	1	9880759	1936691
8429	2	P05451;P48304	P05451	Lithostathine-1-alpha	REG1A	Homo sapiens	18.732	1	1	47702	50865
8430	1	P06310	P06310	Immunoglobulin kappa variable 2-30	IGKV2-30	Homo sapiens	13.184	2	1	1779128	3506810
8431	1	P06331	P06331	Immunoglobulin heavy variable 4-34	IGHV4-34	Homo sapiens	13.814	3	1	719121	484885
8432	1	P06703	P06703	Protein S100-A6	S100A6	Homo sapiens	10.179	1	1	24003800	10497300
8433	1	P06729	P06729	T-cell surface antigen CD2	CD2	Homo sapiens	39.447	1	1	29632	0
8434	1	P06734	P06734	Low affinity immunoglobulin epsilon Fc receptor	FCER2	Homo sapiens	36.468	1	1	35295	0
8435	1	P07204	P07204	Thrombomodulin	THBD	Homo sapiens	60.329	1	1	103188	56824
8436	3	P07477;P07478;Q	P07477	Serine protease 1	PRSS1	Homo sapiens	26.559	1	1	1699890	2058597
8437	1	P07766	P07766	T-cell surface glycoprotein CD3 epsilon chain	CD3E	Homo sapiens	23.146	1	1	0	15954
8438	1	P07919	P07919	Cytochrome b-c1 complex subunit 6, mitochondrial	UQCRH	Homo sapiens	10.739	2	1	1251847	335746
8439	2	P08048;P17010	P08048	Zinc finger Y-chromosomal protein	ZFY	Homo sapiens	90.506	1	1	97301	43246
8440	1	P08134	P08134	Rho-related GTP-binding protein RhoC	RHOC	Homo sapiens	22.006	1	1	899300	280034
8441	2	P08684;P24462	P08684	Cytochrome P450 3A4	CYP3A4	Homo sapiens	57.343	2	1	0	23875
8442	1	P09341	P09341	Growth-regulated alpha protein	CXCL1	Homo sapiens	11.299	2	1	0	318648
8443	1	P09603	P09603	Macrophage colony-stimulating factor 1	CSF1	Homo sapiens	60.18	1	1	16512	0
8444	1	P09651	P09651	Heterogeneous nuclear ribonucleoprotein A1	HNRNPA1	Homo sapiens	38.744	18	1	7067	0
8445	1	P09958	P09958	Furin	FURIN	Homo sapiens	86.681	1	1	37681	22719
8446	1	P0C024	P0C024	Peroxisomal coenzyme A diphosphatase NUDT7	NUDT7	Homo sapiens	26.941	1	1	35256	0
8447	1	P0C0L4	P0C0L4	Complement C4-A	C4A	Homo sapiens	192.788	2	1	3811700	6963030
8448	7	P0C0S8;P20671;Q	P0C0S8	Histone H2A type 1	H2AC17	Homo sapiens	14.093	1	1	38306	0
8449	1	P0C7M7	P0C7M7	Acyl-coenzyme A synthetase ACSM4, mitochondrial	ACSM4	Homo sapiens	65.701	1	1	417381	0
8450	1	P0CF51	P0CF51	T cell receptor gamma constant 1	TRGC1	Homo sapiens	19.803	2	1	33240	0
8451	2	P0CG29;P0CG30	P0CG29	Glutathione S-transferase theta-2	GSTT2	Homo sapiens	27.506	1	1	20272	0
8452	1	P0CK96	P0CK96	Solute carrier family 35 member E2B	SLC35E2B	Homo sapiens	43.775	2	1	41353	0
8453	1	P0DJ07	P0DJ07	Protein PET100 homolog, mitochondrial	PET100	Homo sapiens	9.113	1	1	324050	0
8454	1	P0DTE1	P0DTE1	Probable non-functional immunoglobulin heavy variable 3-38-3	IGHV3-38-3	Homo sapiens	12.671	1	1	31258	172543
8455	1	P10070	P10070	Zinc finger protein GLI2	GLI2	Homo sapiens	167.787	1	1	1765527	0
8456	1	P10074	P10074	Telomere zinc finger-associated protein	ZBTB48	Homo sapiens	77.055	1	1	11056	0
8457	1	P10114	P10114	Ras-related protein Rap-2a	RAP2A	Homo sapiens	20.615	1	1	280728	122995
8458	1	P10827	P10827	Thyroid hormone receptor alpha	THRA	Homo sapiens	54.818	1	1	15925	0
8459	3	P11161;P18146;Q	P11161	E3 SUMO-protein ligase EGR2	EGR2	Homo sapiens	50.302	1	1	10790	0
8460	1	P11474	P11474	Steroid hormone receptor ERR1	ESRRA	Homo sapiens	45.51	2	1	68909	0
8461	1	P11836	P11836	B-lymphocyte antigen CD20	MS4A1	Homo sapiens	33.077	1	1	197307	0
8462	1	P12236	P12236	ADP/ATP translocase 3	SLC25A6	Homo sapiens	32.866	1	1	7703760	1894580
8463	1	P12838	P12838	Defensin alpha 4	DEFA4	Homo sapiens	10.502	1	1	0	88939
8464	1	P13284	P13284	Gamma-interferon-inducible lysosomal thiol reductase	IFI30	Homo sapiens	27.964	1	1	3168960	2562600
8465	1	P13929	P13929	Beta-enolase	ENO3	Homo sapiens	46.988	1	1	65752	0
8466	1	P13994	P13994	Probable splicing factor YJU2B	YJU2B	Homo sapiens	44.8	1	1	40170	0
8467	1	P14598	P14598	Neutrophil cytosol factor 1	NCF1	Homo sapiens	44.684	20	1	0	53691

8468	1	P14859	P14859	POU domain, class 2, transcription factor 1	POU2F1	Homo sapiens	76.471	4	1	63025	0
8469	1	P15056	P15056	Serine/threonine-protein kinase B-raf	BRAF	Homo sapiens	84.438	1	1	0	65924
8470	1	P15509	P15509	Granulocyte-macrophage colony-stimulating factor receptor subunit alpha	CSF2RA	Homo sapiens	46.207	1	1	0	128961
8471	4	P16389;P22001;P	P16389	Potassium voltage-gated channel subfamily A member 2	KCNA2	Homo sapiens	56.716	1	1	11952	0
8472	1	P16402	P16402	Histone H1.3	H1-3	Homo sapiens	22.351	1	1	0	94739
8473	1	P16403	P16403	Histone H1.2	H1-2	Homo sapiens	21.364	4	1	273183	1108180
8474	1	P16442	P16442	Histo-blood group ABO system transferase	ABO	Homo sapiens	40.936	1	1	403141	41506
8475	1	P17275	P17275	Transcription factor JunB	JUNB	Homo sapiens	35.877	1	1	95990	124669
8476	2	P18577;Q02161	P18577	Blood group Rh(CE) polypeptide	RHCE	Homo sapiens	45.56	1	1	189586	393338
8477	1	P18827	P18827	Syndecan-1	SDC1	Homo sapiens	32.462	1	1	290615	0
8478	1	P18859	P18859	ATP synthase-coupling factor 6, mitochondrial	ATP5PF	Homo sapiens	12.586	1	1	189857	0
8479	1	P19099	P19099	Cytochrome P450 11B2, mitochondrial	CYP11B2	Homo sapiens	57.561	1	1	50266	0
8480	1	P19256	P19256	Lymphocyte function-associated antigen 3	CD58	Homo sapiens	28.15	1	1	887398	869728
8481	1	P19438	P19438	Tumor necrosis factor receptor superfamily member 1A	TNFRSF1A	Homo sapiens	50.495	1	1	0	45203
8482	1	P19957	P19957	Elafin	PI3	Homo sapiens	12.269	1	1	0	166091
8483	1	P20036	P20036	HLA class II histocompatibility antigen, DP alpha 1 chain	HLA-DPA1	Homo sapiens	29.38	1	1	301234	335647
8484	1	P20366	P20366	Protachykinin-1	TAC1	Homo sapiens	15.002	1	1	187089	0
8485	1	P20718	P20718	Granzyme H	GZMH	Homo sapiens	27.315	1	1	0	57282
8486	1	P21583	P21583	Kit ligand	KITLG	Homo sapiens	30.899	1	1	68222	0
8487	1	P23025	P23025	DNA repair protein complementing XP-A cells	XPA	Homo sapiens	31.368	1	1	178206	0
8488	1	P23297	P23297	Protein S100-A1	S100A1	Homo sapiens	10.544	1	1	132124	120664
8489	1	P23975	P23975	Sodium-dependent noradrenaline transporter	SLC6A2	Homo sapiens	69.331	1	1	38640	0
8490	1	P24310	P24310	Cytochrome c oxidase subunit 7A1, mitochondrial	COX7A1	Homo sapiens	9.118	1	1	622662	0
8491	1	P24311	P24311	Cytochrome c oxidase subunit 7B, mitochondrial	COX7B	Homo sapiens	9.157	1	1	142614	0
8492	1	P24390	P24390	ER lumen protein-retaining receptor 1	KDELR1	Homo sapiens	24.543	2	1	29662	0
8493	1	P24468	P24468	COUP transcription factor 2	NR2F2	Homo sapiens	45.57	2	1	187287	0
8494	1	P24592	P24592	Insulin-like growth factor-binding protein 6	IGFBP6	Homo sapiens	25.323	1	1	644660	409566
8495	1	P24723	P24723	Protein kinase C eta type	PRKCH	Homo sapiens	77.831	1	1	31377	0
8496	1	P25089	P25089	N-formyl peptide receptor 3	FPR3	Homo sapiens	39.966	1	1	18308	0
8497	1	P25490	P25490	Transcriptional repressor protein YY1	YY1	Homo sapiens	44.713	3	1	266302	374054
8498	1	P25713	P25713	Metallothionein-3	MT3	Homo sapiens	6.925	1	1	398637	0
8499	1	P26012	P26012	Integrin beta-8	ITGB8	Homo sapiens	85.633	1	1	0	10851
8500	1	P26374	P26374	Rab proteins geranylgeranyltransferase component A 2	CHML	Homo sapiens	74.073	1	1	138664	68423
8501	1	P26378	P26378	ELAV-like protein 4	ELAVL4	Homo sapiens	42.4	2	1	36815	0
8502	1	P26992	P26992	Ciliary neurotrophic factor receptor subunit alpha	CNTFR	Homo sapiens	40.632	1	1	76173	0
8503	1	P27144	P27144	Adenylate kinase 4, mitochondrial	AK4	Homo sapiens	25.268	9	1	1297600	356784
8504	1	P27449	P27449	V-type proton ATPase 16 kDa proteolipid subunit c	ATP6V0C	Homo sapiens	15.735	1	1	1474460	4043770
8505	1	P29122	P29122	Proprotein convertase subtilisin/kexin type 6	PCSK6	Homo sapiens	106.419	1	1	0	132117
8506	1	P29374	P29374	AT-rich interactive domain-containing protein 4A	ARID4A	Homo sapiens	142.754	1	1	28161	112101
8507	1	P29508	P29508	Serpin B3	SERPINB3	Homo sapiens	44.564	3	1	0	78663
8508	1	P29558	P29558	RNA-binding motif, single-stranded-interacting protein 1	RBMS1	Homo sapiens	44.507	2	1	247591	178716
8509	1	P30046	P30046	D-dopachrome decarboxylase	DDT	Homo sapiens	12.709	4	1	3353430	706447
8510	1	P30047	P30047	GTP cyclohydrolase 1 feedback regulatory protein	GCHFR	Homo sapiens	9.696	1	1	20811	41320
8511	1	P30408	P30408	Transmembrane 4 L6 family member 1	TM4SF1	Homo sapiens	21.632	1	1	65875	0
8512	1	P31785	P31785	Cytokine receptor common subunit gamma	IL2RG	Homo sapiens	42.287	1	1	72408	0
8513	1	P31944	P31944	Caspase-14	CASP14	Homo sapiens	27.681	1	1	0	175021
8514	1	P31994	P31994	Low affinity immunoglobulin gamma Fc region receptor II-b	FCGR2B	Homo sapiens	34.046	2	1	0	124364
8515	1	P32189	P32189	Glycerol kinase	GK	Homo sapiens	61.246	10	1	256885	538937

8516	1	P32246	P32246	C-C chemokine receptor type 1	CCR1	Homo sapiens	41.175	1	1	35069	88225
8517	1	P34810	P34810	Macrosialin	CD68	Homo sapiens	37.407	1	1	350045	343497
8518	1	P35226	P35226	Polycomb complex protein BMI-1	BMI1	Homo sapiens	36.949	1	1	40712	0
8519	1	P35367	P35367	Histamine H1 receptor	HRH1	Homo sapiens	55.784	1	1	93930	0
8520	1	P36896	P36896	Activin receptor type-1B	ACVR1B	Homo sapiens	56.806	1	1	57276	0
8521	1	P36941	P36941	Tumor necrosis factor receptor superfamily member 3	LTBR	Homo sapiens	46.709	1	1	55768	0
8522	1	P41217	P41217	OX-2 membrane glycoprotein	CD200	Homo sapiens	31.264	1	1	165153	0
8523	1	P41247	P41247	Patatin-like phospholipase domain-containing protein 4	PNPLA4	Homo sapiens	27.98	1	1	9287	0
8524	1	P41567	P41567	Eukaryotic translation initiation factor 1	EIF1	Homo sapiens	12.731	5	1	12865	30088
8525	1	P42677	P42677	40S ribosomal protein S27	RPS27	Homo sapiens	9.46	3	1	4402310	876925
8526	1	P42771	P42771	Cyclin-dependent kinase inhibitor 2A	CDKN2A	Homo sapiens	16.53	2	1	0	56082
8527	1	P42830	P42830	C-X-C motif chemokine 5	CXCL5	Homo sapiens	11.97	1	1	0	162209
8528	1	P46092	P46092	C-C chemokine receptor type 10	CCR10	Homo sapiens	38.415	1	1	185231	0
8529	1	P47710	P47710	Alpha-S1-casein	CSN1S1	Homo sapiens	21.671	1	1	0	80895
8530	1	P48061	P48061	Stromal cell-derived factor 1	CXCL12	Homo sapiens	10.663	1	1	251715	587920
8531	1	P48436	P48436	Transcription factor SOX-9	SOX9	Homo sapiens	56.137	2	1	30275	0
8532	1	P49019	P49019	Hydroxycarboxylic acid receptor 3	HCAR3	Homo sapiens	44.477	4	1	0	15294
8533	1	P49069	P49069	Guided entry of tail-anchored proteins factor CAMLG	CAMLG	Homo sapiens	32.951	1	1	140839	62057
8534	1	P49441	P49441	Inositol polyphosphate 1-phosphatase	INPP1	Homo sapiens	43.998	1	1	277414	141135
8535	1	P49703	P49703	ADP-ribosylation factor-like protein 4D	ARL4D	Homo sapiens	22.156	1	1	39712	0
8536	1	P49716	P49716	CCAAT/enhancer-binding protein delta	CEBPD	Homo sapiens	28.464	1	1	123454	0
8537	2	P49759;Q9HAZ1	P49759	Dual specificity protein kinase CLK1	CLK1	Homo sapiens	57.292	1	1	0	38681
8538	1	P49918	P49918	Cyclin-dependent kinase inhibitor 1C	CDKN1C	Homo sapiens	32.175	1	1	13465	0
8539	1	P50222	P50222	Homeobox protein MOX-2	MEOX2	Homo sapiens	33.594	1	1	18131	0
8540	1	P50238	P50238	Cysteine-rich protein 1	CRIP1	Homo sapiens	8.531	1	1	761844	0
8541	1	P51157	P51157	Ras-related protein Rab-28	RAB28	Homo sapiens	24.841	1	1	451419	58188
8542	1	P51511	P51511	Matrix metalloproteinase-15	MMP15	Homo sapiens	75.808	1	1	32139	0
8543	2	P51668;Q9Y2X8	P51668	Ubiquitin-conjugating enzyme E2 D1	UBE2D1	Homo sapiens	16.604	1	1	815651	822248
8544	1	P51878	P51878	Caspase-5	CASP5	Homo sapiens	49.734	1	1	0	36713
8545	1	P52179	P52179	Myomesin-1	MYOM1	Homo sapiens	187.629	1	1	17573	0
8546	1	P52655	P52655	Transcription initiation factor IIA subunit 1	GTF2A1	Homo sapiens	41.512	1	1	49289	19408
8547	1	P52732	P52732	Kinesin-like protein KIF11	KIF11	Homo sapiens	119.16	1	1	37144	0
8548	1	P52926	P52926	High mobility group protein HMGI-C	HMGA2	Homo sapiens	11.831	1	1	38399	19295
8549	1	P53778	P53778	Mitogen-activated protein kinase 12	MAPK12	Homo sapiens	41.939	1	1	30056	0
8550	1	P53794	P53794	Sodium/myo-inositol cotransporter	SLC5A3	Homo sapiens	79.665	1	1	34429	0
8551	1	P53816	P53816	Phospholipase A and acyltransferase 3	PLAAT3	Homo sapiens	17.937	1	1	40460	0
8552	1	P54259	P54259	Atrophin-1	ATN1	Homo sapiens	125.415	1	1	29027	0
8553	1	P54764	P54764	Ephrin type-A receptor 4	EPHA4	Homo sapiens	109.861	2	1	45446	0
8554	1	P54826	P54826	Growth arrest-specific protein 1	GAS1	Homo sapiens	35.693	1	1	94299	0
8555	1	P54852	P54852	Epithelial membrane protein 3	EMP3	Homo sapiens	18.429	1	1	416616	226233
8556	1	P55061	P55061	Bax inhibitor 1	TMBIM6	Homo sapiens	26.539	1	1	307809	164749
8557	1	P55083	P55083	Microfibril-associated glycoprotein 4	MFAP4	Homo sapiens	28.648	1	1	1059100	21588
8558	1	P55089	P55089	Urocortin	UCN	Homo sapiens	13.456	1	1	34209	0
8559	1	P55201	P55201	Peregrin	BRPF1	Homo sapiens	137.499	1	1	28351	0
8560	1	P55822	P55822	SH3 domain-binding glutamic acid-rich protein	SH3BGR	Homo sapiens	26.084	1	1	96324	0
8561	1	P55854	P55854	Small ubiquitin-related modifier 3	SUMO3	Homo sapiens	11.636	1	1	238567	171798
8562	1	P56181	P56181	NADH dehydrogenase [ubiquinone] flavoprotein 3, mitochondrial	NDUFV3	Homo sapiens	11.94	1	1	1652330	218058
8563	1	P56211	P56211	cAMP-regulated phosphoprotein 19	ARPP19	Homo sapiens	12.323	1	1	107547	0

8564	1	P56270	P56270	Myc-associated zinc finger protein	MAZ	Homo sapiens	48.608	1	1	33176	0
8565	1	P57060	P57060	RWD domain-containing protein 2B	RWDD2B	Homo sapiens	36.329	1	1	43834	0
8566	1	P57087	P57087	Junctional adhesion molecule B	JAM2	Homo sapiens	33.206	1	1	217823	0
8567	1	P57682	P57682	Krueppel-like factor 3	KLF3	Homo sapiens	38.828	1	1	80188	0
8568	1	P57729	P57729	Ras-related protein Rab-38	RAB38	Homo sapiens	23.713	1	1	42672	0
8569	1	P57768	P57768	Sorting nexin-16	SNX16	Homo sapiens	39.167	1	1	93460	0
8570	1	P58005	P58005	Sestrin-3	SESN3	Homo sapiens	57.291	1	1	14328	0
8571	1	P59666	P59666	Neutrophil defensin 3	DEFA3	Homo sapiens	10.242	4	1	0	3334410
8572	1	P60602	P60602	Reactive oxygen species modulator 1	ROMO1	Homo sapiens	8.18	1	1	184967	923198
8573	1	P61024	P61024	Cyclin-dependent kinases regulatory subunit 1	CKS1B	Homo sapiens	9.66	1	1	88262	0
8574	1	P61077	P61077	Ubiquitin-conjugating enzyme E2 D3	UBE2D3	Homo sapiens	16.688	3	1	0	36385
8575	1	P61278	P61278	Somatostatin	SST	Homo sapiens	12.734	1	1	1071900	0
8576	1	P61956	P61956	Small ubiquitin-related modifier 2	SUMO2	Homo sapiens	10.869	2	1	800215	509062
8577	1	P61966	P61966	AP-1 complex subunit sigma-1A	AP1S1	Homo sapiens	18.733	1	1	753249	144722
8578	1	P62310	P62310	U6 snRNA-associated Sm-like protein LSm3	LSM3	Homo sapiens	11.844	1	1	1561440	750389
8579	1	P62854	P62854	40S ribosomal protein S26	RPS26	Homo sapiens	13.012	3	1	2234886	559290
8580	2	P62891;Q59GN2	P62891	60S ribosomal protein L39	RPL39	Homo sapiens	6.403	1	1	171340	0
8581	1	P63027	P63027	Vesicle-associated membrane protein 2	VAMP2	Homo sapiens	12.661	1	1	181287	0
8582	1	P63165	P63165	Small ubiquitin-related modifier 1	SUMO1	Homo sapiens	11.559	2	1	782478	986094
8583	1	P63167	P63167	Dynein light chain 1, cytoplasmic	DYNLL1	Homo sapiens	10.365	4	1	499849	87787
8584	1	P63241	P63241	Eukaryotic translation initiation factor 5A-1	EIF5A	Homo sapiens	16.833	6	1	1180380	610326
8585	1	P63252	P63252	Inward rectifier potassium channel 2	KCNJ2	Homo sapiens	48.287	1	1	0	147448
8586	1	P67775	P67775	Serine/threonine-protein phosphatase 2A catalytic subunit alpha isoform	PPP2CA	Homo sapiens	35.594	1	1	243986	175176
8587	1	P68371	P68371	Tubulin beta-4B chain	TUBB4B	Homo sapiens	49.83	26	1	55648500	11874300
8588	2	P69849;Q5JPE7	P69849	BOS complex subunit NOMO3	NOMO3	Homo sapiens	134.133	1	1	182619	0
8589	1	P69892	P69892	Hemoglobin subunit gamma-2	HBG2	Homo sapiens	16.128	1	1	5612560	5111410
8590	1	P78312	P78312	Protein FAM193A	FAM193A	Homo sapiens	139.99	1	1	163051	0
8591	1	P78333	P78333	Glypican-5	GPC5	Homo sapiens	63.71	1	1	6974	0
8592	1	P78504	P78504	Protein jagged-1	JAG1	Homo sapiens	133.801	1	1	19105	0
8593	1	P78556	P78556	C-C motif chemokine 20	CCL20	Homo sapiens	10.761	1	1	0	216683
8594	1	P81605	P81605	Dermcidin	DCD	Homo sapiens	11.282	1	1	266264	702152
8595	1	P81877	P81877	Single-stranded DNA-binding protein 2	SSBP2	Homo sapiens	37.828	3	1	137199	0
8596	1	P82980	P82980	Retinol-binding protein 5	RBP5	Homo sapiens	15.931	1	1	193324	0
8597	1	P84077	P84077	ADP-ribosylation factor 1	ARF1	Homo sapiens	20.694	5	1	31850	0
8598	1	P84090	P84090	Enhancer of rudimentary homolog	ERH	Homo sapiens	12.256	1	1	909361	350413
8599	1	P84101	P84101	Small EDRK-rich factor 2	SERF2	Homo sapiens	6.897	1	1	48505	0
8600	1	P84243	P84243	Histone H3.3	H3-3B	Homo sapiens	15.325	3	1	0	123138
8601	1	P98153	P98153	Integral membrane protein DGCR2/IDD	DGCR2	Homo sapiens	60.812	1	1	155266	0
8602	1	Q00536	Q00536	Cyclin-dependent kinase 16	CDK16	Homo sapiens	55.718	1	1	141187	58164
8603	4	Q00887;Q00889;Q	Q00887	Pregnancy-specific beta-1-glycoprotein 9	PSG9	Homo sapiens	48.272	1	1	0	21516
8604	1	Q00G26	Q00G26	Perilipin-5	PLIN5	Homo sapiens	50.79	1	1	0	14909
8605	1	Q01628	Q01628	Interferon-induced transmembrane protein 3	IFITM3	Homo sapiens	14.633	2	1	171075	423810
8606	1	Q01650	Q01650	Large neutral amino acids transporter small subunit 1	SLC7A5	Homo sapiens	55.011	1	1	213041	444023
8607	1	Q01664	Q01664	Transcription factor AP-4	TFAP4	Homo sapiens	38.725	1	1	633465	61156
8608	1	Q02094	Q02094	Ammonium transporter Rh type A	RHAG	Homo sapiens	44.197	1	1	668947	2385270
8609	1	Q02241	Q02241	Kinesin-like protein KIF23	KIF23	Homo sapiens	110.06	1	1	43244	25175
8610	1	Q02556	Q02556	Interferon regulatory factor 8	IRF8	Homo sapiens	48.357	1	1	70707	0

8611	1	Q02742	Q02742	Beta-1,3-galactosyl-O-glycosyl-glycoprotein beta-1,6-N-acetylglucosaminyltransferase	GCNT1	Homo sapiens	49.8	1	1	77877	0
8612	1	Q02747	Q02747	Guanylin	GUCA2A	Homo sapiens	12.386	1	1	67924	0
8613	1	Q03393	Q03393	6-pyruvoyl tetrahydrobiopterin synthase	PTS	Homo sapiens	16.387	1	1	51248	18886
8614	1	Q03692	Q03692	Collagen alpha-1(X) chain	COL10A1	Homo sapiens	66.158	1	1	0	54781
8615	1	Q04724	Q04724	Transducin-like enhancer protein 1	TLE1	Homo sapiens	83.201	4	1	224543	0
8616	1	Q05315	Q05315	Galectin-10	CLC	Homo sapiens	16.452	1	1	849715	194823
8617	1	Q05932	Q05932	Folylpolyglutamate synthase, mitochondrial	FPGS	Homo sapiens	64.608	1	1	64218	0
8618	1	Q05BQ5	Q05BQ5	MBT domain-containing protein 1	MBTD1	Homo sapiens	70.546	1	1	73762	0
8619	1	Q06413	Q06413	Myocyte-specific enhancer factor 2C	MEF2C	Homo sapiens	51.22	1	1	68693	0
8620	1	Q06828	Q06828	Fibromodulin	FMOD	Homo sapiens	43.179	6	1	1127130	58733
8621	1	Q07108	Q07108	Early activation antigen CD69	CD69	Homo sapiens	22.561	1	1	0	67226
8622	1	Q07654	Q07654	Trefoil factor 3	TFF3	Homo sapiens	8.639	1	1	720665	0
8623	1	Q08043	Q08043	Alpha-actinin-3	ACTN3	Homo sapiens	103.244	1	1	1019460	0
8624	1	Q08AI8	Q08AI8	Protein mab-21-like 4	MAB21L4	Homo sapiens	49.563	1	1	6193	0
8625	1	Q08ET2	Q08ET2	Sialic acid-binding Ig-like lectin 14	SIGLEC14	Homo sapiens	43.973	1	1	0	58233
8626	1	Q10587	Q10587	Thyrotroph embryonic factor	TEF	Homo sapiens	33.246	1	1	43008	0
8627	1	Q12774	Q12774	Rho guanine nucleotide exchange factor 5	ARHGEF5	Homo sapiens	176.801	20	1	0	30431
8628	1	Q12809	Q12809	Potassium voltage-gated channel subfamily H member 2	KCNH2	Homo sapiens	126.656	1	1	20749	0
8629	1	Q12834	Q12834	Cell division cycle protein 20 homolog	CDC20	Homo sapiens	54.723	1	1	13306	0
8630	1	Q12891	Q12891	Hyaluronidase-2	HYAL2	Homo sapiens	53.858	1	1	155543	50010
8631	1	Q12893	Q12893	Transmembrane protein 115	TMEM115	Homo sapiens	38.198	1	1	199973	0
8632	1	Q12894	Q12894	Interferon-related developmental regulator 2	IFRD2	Homo sapiens	54.813	1	1	160410	0
8633	1	Q12946	Q12946	Forkhead box protein F1	FOXF1	Homo sapiens	40.124	1	1	139510	0
8634	1	Q12974	Q12974	Protein tyrosine phosphatase type IVA 2	PTP4A2	Homo sapiens	19.126	1	1	37890	0
8635	1	Q12999	Q12999	Tetraspanin-31	TSPAN31	Homo sapiens	23.053	1	1	106948	37109
8636	1	Q13105	Q13105	Zinc finger and BTB domain-containing protein 17	ZBTB17	Homo sapiens	87.928	1	1	39354	0
8637	1	Q13227	Q13227	G protein pathway suppressor 2	GPS2	Homo sapiens	36.687	1	1	94686	0
8638	1	Q13432	Q13432	Protein unc-119 homolog A	UNC119	Homo sapiens	26.962	1	1	105174	197646
8639	1	Q13467	Q13467	Frizzled-5	FZD5	Homo sapiens	64.506	1	1	74693	0
8640	1	Q13477	Q13477	Mucosal addressin cell adhesion molecule 1	MADCAM1	Homo sapiens	40.158	1	1	126719	0
8641	1	Q13491	Q13491	Neuronal membrane glycoprotein M6-b	GPM6B	Homo sapiens	28.989	1	1	263227	0
8642	1	Q13506	Q13506	NGFI-A-binding protein 1	NAB1	Homo sapiens	54.401	1	1	41135	0
8643	1	Q13522	Q13522	Protein phosphatase 1 regulatory subunit 1A	PPP1R1A	Homo sapiens	19.009	1	1	55478	0
8644	1	Q13571	Q13571	Lysosomal-associated transmembrane protein 5	LAPTM5	Homo sapiens	29.935	1	1	41228	529025
8645	1	Q13670	Q13670	Putative postmeiotic segregation increased 2-like protein 11	PMS2P11	Homo sapiens	28.554	1	1	117637	115991
8646	1	Q13873	Q13873	Bone morphogenetic protein receptor type-2	BMPR2	Homo sapiens	115.204	1	1	89459	0
8647	1	Q13885	Q13885	Tubulin beta-2A chain	TUBB2A	Homo sapiens	49.906	1	1	15315600	231438
8648	1	Q14190	Q14190	Single-minded homolog 2	SIM2	Homo sapiens	73.22	1	1	85657	0
8649	1	Q14202	Q14202	Zinc finger MYM-type protein 3	ZMYM3	Homo sapiens	152.381	1	1	22611	0
8650	1	Q14512	Q14512	Fibroblast growth factor-binding protein 1	FGFBP1	Homo sapiens	26.266	1	1	91788	0
8651	1	Q14542	Q14542	Equilibrative nucleoside transporter 2	SLC29A2	Homo sapiens	50.111	1	1	50272	0
8652	1	Q14657	Q14657	EKC/KEOPS complex subunit LAGE3	LAGE3	Homo sapiens	14.804	1	1	267971	71515
8653	1	Q14681	Q14681	BTB/POZ domain-containing protein KCTD2	KCTD2	Homo sapiens	28.525	1	1	126742	0
8654	1	Q14765	Q14765	Signal transducer and activator of transcription 4	STAT4	Homo sapiens	85.939	1	1	20416	0
8655	1	Q14802	Q14802	FXYD domain-containing ion transport regulator 3	FXYD3	Homo sapiens	9.261	1	1	900347	0
8656	1	Q14865	Q14865	AT-rich interactive domain-containing protein 5B	ARID5B	Homo sapiens	132.375	1	1	100032	0
8657	1	Q14CZ7	Q14CZ7	FAST kinase domain-containing protein 3, mitochondrial	FASTKD3	Homo sapiens	75.689	1	1	39405	0

8658	1	Q15011	Q15011	Homocysteine-responsive endoplasmic reticulum-resident ubiquitin-like domain member 1 protein	HERPUD1	Homo sapiens	43.719	1	1	61528	0
8659	1	Q15041	Q15041	ADP-ribosylation factor-like protein 6-interacting protein 1	ARL6IP1	Homo sapiens	23.363	1	1	107455	0
8660	1	Q15053	Q15053	Uncharacterized protein KIAA0040	KIAA0040	Homo sapiens	11.433	1	1	0	60890
8661	1	Q15139	Q15139	Serine/threonine-protein kinase D1	PRKD1	Homo sapiens	101.701	3	1	22067	0
8662	1	Q15198	Q15198	Platelet-derived growth factor receptor-like protein	PDGFRL	Homo sapiens	41.861	1	1	17422	0
8663	1	Q15532	Q15532	Protein SSXT	SS18	Homo sapiens	45.929	1	1	331667	61107
8664	2	Q15583;Q9GZN2	Q15583	Homeobox protein TGIF1	TGIF1	Homo sapiens	43.013	1	1	35816	0
8665	1	Q15651	Q15651	High mobility group nucleosome-binding domain-containing protein 3	HMGN3	Homo sapiens	10.663	1	1	127878	54942
8666	1	Q15669	Q15669	Rho-related GTP-binding protein RhoH	RHOH	Homo sapiens	21.329	1	1	0	16460
8667	1	Q15762	Q15762	CD226 antigen	CD226	Homo sapiens	38.614	1	1	0	16213
8668	1	Q15818	Q15818	Neuronal pentraxin-1	NPTX1	Homo sapiens	47.122	1	1	43909	0
8669	1	Q15847	Q15847	Adipogenesis regulatory factor	ADIRF	Homo sapiens	7.851	1	1	387177	0
8670	1	Q16143	Q16143	Beta-synuclein	SNCB	Homo sapiens	14.287	1	1	35343	0
8671	1	Q16342	Q16342	Programmed cell death protein 2	PDCD2	Homo sapiens	38.591	1	1	129529	28683
8672	1	Q16514	Q16514	Transcription initiation factor TFIID subunit 12	TAF12	Homo sapiens	17.922	1	1	124071	0
8673	1	Q16558	Q16558	Calcium-activated potassium channel subunit beta-1	KCNMB1	Homo sapiens	21.795	1	1	379080	0
8674	1	Q16570	Q16570	Atypical chemokine receptor 1	ACKR1	Homo sapiens	35.552	1	1	56890	0
8675	1	Q16602	Q16602	Calcitonin gene-related peptide type 1 receptor	CALCRL	Homo sapiens	52.977	1	1	149641	0
8676	1	Q16619	Q16619	Cardiotrophin-1	CTF1	Homo sapiens	21.228	1	1	63998	0
8677	1	Q16651	Q16651	Prostasin	PRSS8	Homo sapiens	36.432	1	1	175901	0
8678	1	Q16760	Q16760	Diacylglycerol kinase delta	DGKD	Homo sapiens	134.527	1	1	30744	0
8679	1	Q16842	Q16842	CMP-N-acetylneuraminate-beta-galactosamide-alpha-2,3-sialyltransferase 2	ST3GAL2	Homo sapiens	40.173	1	1	0	19475
8680	1	Q16854	Q16854	Deoxyguanosine kinase, mitochondrial	DGUOK	Homo sapiens	32.057	1	1	38601	0
8681	1	Q16873	Q16873	Leukotriene C4 synthase	LTC4S	Homo sapiens	16.564	1	1	145341	0
8682	1	Q2M3V2	Q2M3V2	Ankyrin repeat domain-containing protein SOWAHA	SOWAHA	Homo sapiens	57.408	1	1	79047	0
8683	1	Q2T9K0	Q2T9K0	Transmembrane protein 44	TMEM44	Homo sapiens	52.199	1	1	48502	0
8684	1	Q2TBE0	Q2TBE0	CWF19-like protein 2	CWF19L2	Homo sapiens	103.789	1	1	19888	0
8685	1	Q3B7T1	Q3B7T1	Erythroid differentiation-related factor 1	EDRF1	Homo sapiens	138.529	1	1	21172	0
8686	1	Q3KRA6	Q3KRA6	UPF0538 protein C2orf76	C2orf76	Homo sapiens	14.608	1	1	151261	124539
8687	1	Q3T8J9	Q3T8J9	GON-4-like protein	GON4L	Homo sapiens	248.626	1	1	22627	0
8688	1	Q3YBM2	Q3YBM2	Transmembrane protein 176B	TMEM176B	Homo sapiens	29.056	1	1	2222264	520095
8689	1	Q499Z4	Q499Z4	Zinc finger protein 672	ZNF672	Homo sapiens	50.224	1	1	6975	0
8690	1	Q49AJ0	Q49AJ0	Protein FAM135B	FAM135B	Homo sapiens	155.772	1	1	52614	0
8691	1	Q49B96	Q49B96	Cytochrome c oxidase assembly protein COX19	COX19	Homo sapiens	10.393	1	1	171756	0
8692	1	Q4KWH8	Q4KWH8	1-phosphatidylinositol 4,5-bisphosphate phosphodiesterase eta-1	PLCH1	Homo sapiens	189.225	1	1	19721	0
8693	1	Q53GA4	Q53GA4	Pleckstrin homology-like domain family A member 2	PHLDA2	Homo sapiens	17.091	1	1	14456	0
8694	1	Q53GD3	Q53GD3	Choline transporter-like protein 4	SLC44A4	Homo sapiens	79.254	1	1	868202	229878
8695	1	Q53HC5	Q53HC5	Kelch-like protein 26	KLHL26	Homo sapiens	68.141	1	1	24550	0
8696	1	Q53QV2	Q53QV2	Protein LBH	LBH	Homo sapiens	12.217	1	1	69098	0
8697	2	Q53S08;Q9H0N0	Q53S08	Ras-related protein Rab-6D	RAB6D	Homo sapiens	28.243	1	1	137086	0
8698	1	Q587I9	Q587I9	Vesicle transport protein SFT2C	SFT2D3	Homo sapiens	21.79	1	1	394798	61827
8699	1	Q58FG0	Q58FG0	Putative heat shock protein HSP 90-alpha A5	HSP90AA5P	Homo sapiens	38.739	1	1	0	85849
8700	1	Q5BJH7	Q5BJH7	Protein YIF1B	YIF1B	Homo sapiens	34.435	1	1	0	277087
8701	2	Q5BKY9;Q8N9E0	Q5BKY9	Protein FAM133B	FAM133B	Homo sapiens	28.386	1	1	159789	0
8702	1	Q5EG05	Q5EG05	Caspase recruitment domain-containing protein 16	CARD16	Homo sapiens	22.627	1	1	77588	391462
8703	1	Q5GJ75	Q5GJ75	Tumor necrosis factor alpha-induced protein 8-like protein 3	TNFAIP8L3	Homo sapiens	32.658	1	1	180698	0

8704	1	Q5H9L2	Q5H9L2	Transcription elongation factor A protein-like 5	TCEAL5	Homo sapiens	23.306	1	1	104772	0
8705	1	Q5JS13	Q5JS13	Ras-specific guanine nucleotide-releasing factor RalGPS1	RALGPS1	Homo sapiens	62.133	1	1	14757	0
8706	1	Q5JTB6	Q5JTB6	Placenta-specific protein 9	PLAC9	Homo sapiens	10.307	1	1	37732	0
8707	1	Q5JTZ5	Q5JTZ5	Uncharacterized protein C9orf152	C9orf152	Homo sapiens	26.316	1	1	28936	0
8708	1	Q5JXM2	Q5JXM2	Probable methyltransferase-like protein 24	METTL24	Homo sapiens	41.329	1	1	96379	0
8709	1	Q5MY95	Q5MY95	Ectonucleoside triphosphate diphosphohydrolase 8	ENTPD8	Homo sapiens	53.905	1	1	188810	0
8710	1	Q5NUL3	Q5NUL3	Free fatty acid receptor 4	FFAR4	Homo sapiens	40.495	1	1	122613	0
8711	1	Q5PSV4	Q5PSV4	Breast cancer metastasis-suppressor 1-like protein	BRMS1L	Homo sapiens	37.63	1	1	64405	0
8712	1	Q5SQH8	Q5SQH8	Uncharacterized protein C6orf136	C6orf136	Homo sapiens	35.797	1	1	59474	0
8713	1	Q5SRI9	Q5SRI9	Glycoprotein endo-alpha-1,2-mannosidase	MANEA	Homo sapiens	53.674	1	1	49129	0
8714	1	Q5T0B9	Q5T0B9	Zinc finger protein 362	ZNF362	Homo sapiens	45.814	1	1	20495	0
8715	2	Q5T1J5;Q9Y6H1	Q5T1J5	Putative coiled-coil-helix-coiled-coil-helix domain-containing protein CHCHD2P9, mitochondrial	CHCHD2P9	Homo sapiens	15.488	1	1	153980	153429
8716	1	Q5T3I0	Q5T3I0	G patch domain-containing protein 4	GPATCH4	Homo sapiens	50.383	1	1	65637	0
8717	1	Q5T601	Q5T601	Adhesion G-protein coupled receptor F1	ADGRF1	Homo sapiens	101.368	1	1	0	60398
8718	1	Q5T6J7	Q5T6J7	Probable gluconokinase	IDNK	Homo sapiens	20.58	1	1	76280	0
8719	1	Q5T7N3	Q5T7N3	KN motif and ankyrin repeat domain-containing protein 4	KANK4	Homo sapiens	107.343	1	1	36224	0
8720	1	Q5T9A4	Q5T9A4	ATPase family AAA domain-containing protein 3B	ATAD3B	Homo sapiens	72.573	1	1	703691	499914
8721	1	Q5TA50	Q5TA50	Ceramide-1-phosphate transfer protein	CPTP	Homo sapiens	24.364	1	1	65112	0
8722	1	Q5TC63	Q5TC63	Growth hormone-regulated TBC protein 1	GRTP1	Homo sapiens	38.553	1	1	72713	0
8723	2	Q5TC82;Q9HBD1	Q5TC82	Roquin-1	RC3H1	Homo sapiens	125.738	1	1	23731	0
8724	1	Q5TD97	Q5TD97	Four and a half LIM domains protein 5	FHL5	Homo sapiens	32.72	1	1	18394	0
8725	1	Q5TYW1	Q5TYW1	Zinc finger protein 658	ZNF658	Homo sapiens	122.277	1	1	110969	60962
8726	1	Q5U3C3	Q5U3C3	Transmembrane protein 164	TMEM164	Homo sapiens	33.507	1	1	88002	170648
8727	1	Q5U5R9	Q5U5R9	Probable E3 ubiquitin-protein ligase HECTD2	HECTD2	Homo sapiens	88.124	1	1	104400	0
8728	1	Q5VVW2	Q5VVW2	GTPase-activating Rap/Ran-GAP domain-like protein 3	GARNL3	Homo sapiens	112.854	1	1	11089	0
8729	1	Q5VWG9	Q5VWG9	Transcription initiation factor TFIID subunit 3	TAF3	Homo sapiens	103.583	1	1	108169	0
8730	1	Q5VXD3	Q5VXD3	Sterile alpha motif domain-containing protein 13	SAMD13	Homo sapiens	13.57	1	1	62185	0
8731	1	Q5VZF2	Q5VZF2	Muscleblind-like protein 2	MBNL2	Homo sapiens	40.518	1	1	41720	0
8732	1	Q5W0U4	Q5W0U4	B box and SPRY domain-containing protein	BSPRY	Homo sapiens	44.38	1	1	109164	0
8733	1	Q5XUX0	Q5XUX0	F-box only protein 31	FBXO31	Homo sapiens	60.663	1	1	57157	0
8734	1	Q63HQ0	Q63HQ0	AP-1 complex-associated regulatory protein	AP1AR	Homo sapiens	34.28	1	1	67956	0
8735	1	Q64LD2	Q64LD2	WD repeat-containing protein 25	WDR25	Homo sapiens	60.16	1	1	42995	0
8736	1	Q659A1	Q659A1	Little elongation complex subunit 2	ICE2	Homo sapiens	110.013	1	1	36647	0
8737	1	Q68BL8	Q68BL8	Olfactomedin-like protein 2B	OLFML2B	Homo sapiens	83.999	1	1	0	187333
8738	1	Q695T7	Q695T7	Sodium-dependent neutral amino acid transporter B(0)AT1	SLC6A19	Homo sapiens	71.11	1	1	121235	0
8739	1	Q69YW2	Q69YW2	Protein stum homolog	STUM	Homo sapiens	15.007	1	1	408831	0
8740	1	Q6DN14	Q6DN14	Multiple C2 and transmembrane domain-containing protein 1	MCTP1	Homo sapiens	111.626	1	1	167811	42985
8741	1	Q6GTX8	Q6GTX8	Leukocyte-associated immunoglobulin-like receptor 1	LAIR1	Homo sapiens	31.466	1	1	0	30424
8742	1	Q6ICB0	Q6ICB0	Desumoylating isopeptidase 1	DESI1	Homo sapiens	18.261	1	1	0	113959
8743	1	Q6IE81	Q6IE81	Protein Jade-1	JADE1	Homo sapiens	95.534	1	1	55623	0
8744	1	Q6IPR3	Q6IPR3	tRNA wybutosine-synthesizing protein 3 homolog	TYW3	Homo sapiens	29.793	1	1	34751	0
8745	1	Q6IPX3	Q6IPX3	Transcription elongation factor A protein-like 6	TCEAL6	Homo sapiens	22.297	1	1	45154	0
8746	1	Q6J4K2	Q6J4K2	Mitochondrial sodium/calcium exchanger protein	SLC8B1	Homo sapiens	64.233	1	1	248528	573770
8747	1	Q6L9W6	Q6L9W6	Beta-1,4-N-acetylgalactosaminyltransferase 3	B4GALNT3	Homo sapiens	114.977	1	1	60774	0
8748	1	Q6NT76	Q6NT76	Homeobox-containing protein 1	HMBOX1	Homo sapiens	47.277	1	1	24814	0
8749	1	Q6NTF9	Q6NTF9	Rhomboid domain-containing protein 2	RHBDD2	Homo sapiens	39.2	1	1	98579	0
8750	1	Q6NUS6	Q6NUS6	Tectonic-3	TCTN3	Homo sapiens	66.157	1	1	90927	0

8751	1	Q6NVV3	Q6NVV3	Magnesium transporter NIPA3	NIPAL1	Homo sapiens	44.637	1	1	61503	0
8752	1	Q6NXT1	Q6NXT1	Ankyrin repeat domain-containing protein 54	ANKRD54	Homo sapiens	32.505	1	1	72195	0
8753	1	Q6NZ67	Q6NZ67	Mitotic-spindle organizing protein 2B	MZT2B	Homo sapiens	16.224	1	1	332421	0
8754	1	Q6P1Q9	Q6P1Q9	tRNA N(3)-methylcytidine methyltransferase METTL2B	METTL2B	Homo sapiens	43.427	2	1	215834	75653
8755	1	Q6P1R3	Q6P1R3	Myb/SANT-like DNA-binding domain-containing protein 2	MSANTD2	Homo sapiens	61.321	1	1	51405	0
8756	1	Q6P1R4	Q6P1R4	tRNA-dihydrouridine(16/17) synthase [NAD(P)(+)]-like	DUS1L	Homo sapiens	53.233	1	1	33513	0
8757	1	Q6P4F2	Q6P4F2	Ferredoxin-2, mitochondrial	FDX2	Homo sapiens	19.889	1	1	54034	0
8758	1	Q6P4I2	Q6P4I2	WD repeat-containing protein 73	WDR73	Homo sapiens	41.685	1	1	101262	0
8759	1	Q6P582	Q6P582	Mitotic-spindle organizing protein 2A	MZT2A	Homo sapiens	16.219	2	1	138921	0
8760	1	Q6P597	Q6P597	Kinesin light chain 3	KLC3	Homo sapiens	55.364	1	1	331161	184294
8761	1	Q6P5W5	Q6P5W5	Zinc transporter ZIP4	SLC39A4	Homo sapiens	68.407	1	1	5279	0
8762	1	Q6P5X5	Q6P5X5	UPF0545 protein C22orf39	C22orf39	Homo sapiens	16.806	1	1	76620	0
8763	1	Q6P9H4	Q6P9H4	Connector enhancer of kinase suppressor of ras 3	CNKSR3	Homo sapiens	61.906	3	1	8535	0
8764	1	Q6P9H5	Q6P9H5	GTPase IMAP family member 6	GIMAP6	Homo sapiens	32.949	1	1	0	39394
8765	1	Q6PCB6	Q6PCB6	Alpha/beta hydrolase domain-containing protein 17C	ABHD17C	Homo sapiens	35.829	1	1	19366	0
8766	1	Q6PHW0	Q6PHW0	Iodotyrosine deiodinase 1	IYD	Homo sapiens	33.356	1	1	30990	0
8767	1	Q6PIY7	Q6PIY7	Poly(A) RNA polymerase GLD2	TENT2	Homo sapiens	56.027	1	1	51403	39030
8768	1	Q6QEF8	Q6QEF8	Coronin-6	CORO6	Homo sapiens	52.762	1	1	94463	0
8769	1	Q6QNY1	Q6QNY1	Biogenesis of lysosome-related organelles complex 1 subunit 2	BLOC1S2	Homo sapiens	15.961	1	1	223046	141308
8770	1	Q6SPF0	Q6SPF0	Sterile alpha motif domain-containing protein 1	SAMD1	Homo sapiens	56.05	1	1	154663	41137
8771	1	Q6UWP8	Q6UWP8	Suprabasin	SBSN	Homo sapiens	60.541	1	1	18770	0
8772	1	Q6UWU2	Q6UWU2	Beta-galactosidase-1-like protein	GLB1L	Homo sapiens	74.16	1	1	41080	0
8773	1	Q6UWU4	Q6UWU4	Bombesin receptor-activated protein C6orf89	C6orf89	Homo sapiens	39.869	1	1	26389	0
8774	1	Q6UX07	Q6UX07	Dehydrogenase/reductase SDR family member 13	DHRS13	Homo sapiens	40.844	1	1	145993	55652
8775	1	Q6UX65	Q6UX65	DNA damage-regulated autophagy modulator protein 2	DRAM2	Homo sapiens	29.767	1	1	85688	84726
8776	1	Q6UXH8	Q6UXH8	Collagen and calcium-binding EGF domain-containing protein 1	CCBE1	Homo sapiens	44.104	1	1	355212	0
8777	1	Q6UY01	Q6UY01	Leucine-rich repeat-containing protein 31	LRRC31	Homo sapiens	61.488	1	1	89279	473818
8778	1	Q6WRI0	Q6WRI0	Immunoglobulin superfamily member 10	IGSF10	Homo sapiens	290.843	1	1	106414	0
8779	1	Q6Y1H2	Q6Y1H2	Very-long-chain (3R)-3-hydroxyacyl-CoA dehydratase 2	HACD2	Homo sapiens	28.369	1	1	979364	545607
8780	1	Q6YBV0	Q6YBV0	Neutral amino acid uniporter 4	SLC36A4	Homo sapiens	56.159	1	1	4423	0
8781	1	Q6ZMG9	Q6ZMG9	Ceramide synthase 6	CERS6	Homo sapiens	44.889	1	1	386714	160329
8782	1	Q6ZMH5	Q6ZMH5	Zinc transporter ZIP5	SLC39A5	Homo sapiens	56.464	1	1	25468	0
8783	1	Q6ZN30	Q6ZN30	Zinc finger protein basonuclin-2	BNC2	Homo sapiens	122.332	1	1	17104	0
8784	1	Q6ZN54	Q6ZN54	Differentially expressed in FDCP 8 homolog	DEF8	Homo sapiens	58.711	1	1	61866	46198
8785	1	Q6ZNA5	Q6ZNA5	Ferric-chelate reductase 1	FRRS1	Homo sapiens	66.115	1	1	12399	0
8786	1	Q6ZSJ8	Q6ZSJ8	Uncharacterized protein C1orf122	C1orf122	Homo sapiens	11.469	1	1	118681	61560
8787	1	Q6ZU65	Q6ZU65	Ubinuclein-2	UBN2	Homo sapiens	146.091	1	1	36894	14340
8788	1	Q6ZW13	Q6ZW13	Uncharacterized protein C16orf86	C16orf86	Homo sapiens	33.511	1	1	52908	0
8789	1	Q709F0	Q709F0	Acyl-CoA dehydrogenase family member 11	ACAD11	Homo sapiens	87.264	1	1	0	26319
8790	1	Q70EK8	Q70EK8	Inactive ubiquitin carboxyl-terminal hydrolase 53	USP53	Homo sapiens	120.807	1	1	36981	0
8791	1	Q70Z53	Q70Z53	Protein FRA10AC1	FRA10AC1	Homo sapiens	37.548	1	1	79004	0
8792	1	Q719H9	Q719H9	BTB/POZ domain-containing protein KCTD1	KCTD1	Homo sapiens	29.404	1	1	23361	0
8793	1	Q71UM5	Q71UM5	40S ribosomal protein S27-like	RPS27L	Homo sapiens	9.476	1	1	2320950	662315
8794	1	Q7L523	Q7L523	Ras-related GTP-binding protein A	RRAGA	Homo sapiens	36.566	6	1	36453	22302
8795	1	Q7L5Y6	Q7L5Y6	DET1 homolog	DET1	Homo sapiens	63.849	1	1	27592	0
8796	1	Q7L8C5	Q7L8C5	Synaptotagmin-13	SYT13	Homo sapiens	46.886	1	1	28148	0
8797	1	Q7RTP0	Q7RTP0	Magnesium transporter NIPA1	NIPA1	Homo sapiens	34.557	1	1	48681	44771
8798	1	Q7RTV0	Q7RTV0	PHD finger-like domain-containing protein 5A	PHF5A	Homo sapiens	12.404	1	1	93452	54409

8799	1	Q7Z309	Q7Z309	PABIR family member 2	PABIR2	Homo sapiens	26.928	1	1	74811	0
8800	1	Q7Z3D4	Q7Z3D4	LysM and putative peptidoglycan-binding domain-containing protein 3	LYSMD3	Homo sapiens	34.539	1	1	37969	0
8801	1	Q7Z3F1	Q7Z3F1	Integral membrane protein GPR155	GPR155	Homo sapiens	96.92	1	1	21641	0
8802	1	Q7Z3J3	Q7Z3J3	RanBP2-like and GRIP domain-containing protein 4	RGPD4	Homo sapiens	197.29	2	1	41342	0
8803	1	Q7Z3K6	Q7Z3K6	Mesoderm induction early response protein 3	MIER3	Homo sapiens	61.439	1	1	209312	0
8804	1	Q7Z3Q1	Q7Z3Q1	Solute carrier family 46 member 3	SLC46A3	Homo sapiens	51.52	1	1	156624	82675
8805	1	Q7Z4R8	Q7Z4R8	UPF0669 protein C6orf120	C6orf120	Homo sapiens	20.772	1	1	232154	260771
8806	1	Q7Z736	Q7Z736	Pleckstrin homology domain-containing family H member 3	PLEKHH3	Homo sapiens	85.32	1	1	29998	0
8807	1	Q7Z7D3	Q7Z7D3	V-set domain-containing T-cell activation inhibitor 1	VTCN1	Homo sapiens	30.878	1	1	0	114427
8808	1	Q7Z7H8	Q7Z7H8	39S ribosomal protein L10, mitochondrial	MRPL10	Homo sapiens	29.281	1	1	21440	0
8809	1	Q7Z7K0	Q7Z7K0	COX assembly mitochondrial protein homolog	CMC1	Homo sapiens	12.488	1	1	110419	74572
8810	1	Q7Z7L1	Q7Z7L1	Schlafen family member 11	SLFN11	Homo sapiens	102.837	3	1	0	44899
8811	1	Q7Z7N9	Q7Z7N9	Transmembrane protein 179B	TMEM179B	Homo sapiens	23.548	1	1	214053	113146
8812	1	Q86SJ2	Q86SJ2	Amphoterin-induced protein 2	AMIGO2	Homo sapiens	57.935	1	1	58471	0
8813	1	Q86TG7	Q86TG7	Retrotransposon-derived protein PEG10	PEG10	Homo sapiens	80.176	1	1	15940	0
8814	1	Q86TX2	Q86TX2	Acyl-coenzyme A thioesterase 1	ACOT1	Homo sapiens	46.277	19	1	139410	0
8815	1	Q86UE6	Q86UE6	Leucine-rich repeat transmembrane neuronal protein 1	LRRTM1	Homo sapiens	58.643	1	1	139721	0
8816	1	Q86UE8	Q86UE8	Serine/threonine-protein kinase tousled-like 2	TLK2	Homo sapiens	87.662	1	1	34613	36698
8817	1	Q86UT5	Q86UT5	Na(+)/H(+) exchange regulatory cofactor NHE-RF4	NHERF4	Homo sapiens	61.034	1	1	26136	0
8818	1	Q86UY6	Q86UY6	N-alpha-acetyltransferase 40	NAA40	Homo sapiens	27.194	1	1	80501	45724
8819	1	Q86V97	Q86V97	Kelch repeat and BTB domain-containing protein 6	KBTBD6	Homo sapiens	76.139	1	1	68819	0
8820	1	Q86VF5	Q86VF5	2-acylglycerol O-acyltransferase 3	MOGAT3	Homo sapiens	38.732	1	1	25041	0
8821	1	Q86VR8	Q86VR8	Four-jointed box protein 1	FJX1	Homo sapiens	48.507	1	1	0	68519
8822	1	Q86VU5	Q86VU5	Catechol O-methyltransferase domain-containing protein 1	COMTD1	Homo sapiens	28.809	1	1	198143	0
8823	1	Q86VZ1	Q86VZ1	P2Y purinoceptor 8	P2RY8	Homo sapiens	40.636	1	1	0	71849
8824	1	Q86WT1	Q86WT1	Tetratricopeptide repeat protein 30A	TTC30A	Homo sapiens	76.137	5	1	26668	0
8825	1	Q86WV1	Q86WV1	Src kinase-associated phosphoprotein 1	SKAP1	Homo sapiens	41.433	1	1	56472	0
8826	1	Q86WW8	Q86WW8	Cytochrome c oxidase assembly factor 5	COA5	Homo sapiens	8.373	1	1	85006	0
8827	1	Q86X40	Q86X40	Leucine-rich repeat-containing protein 28	LRRC28	Homo sapiens	41.913	1	1	28271	0
8828	1	Q86X53	Q86X53	Glutamate-rich protein 1	ERICH1	Homo sapiens	48.984	1	1	784141	0
8829	1	Q86X95	Q86X95	Corepressor interacting with RBPJ 1	CIR1	Homo sapiens	52.314	1	1	42573	0
8830	1	Q86XD5	Q86XD5	Protein FAM131B	FAM131B	Homo sapiens	35.768	1	1	25788	0
8831	1	Q86XE3	Q86XE3	Calcium uptake protein 3, mitochondrial	MICU3	Homo sapiens	60.713	1	1	30837	0
8832	1	Q86XI6	Q86XI6	Protein phosphatase 1 regulatory subunit 3B	PPP1R3B	Homo sapiens	32.696	1	1	77265	68076
8833	1	Q86XI8	Q86XI8	Uncharacterized protein ZSWIM9	ZSWIM9	Homo sapiens	70.073	1	1	8223	0
8834	1	Q86XN6	Q86XN6	Zinc finger protein 761	ZNF761	Homo sapiens	87.719	3	1	137147	0
8835	1	Q86XR7	Q86XR7	TIR domain-containing adapter molecule 2	TICAM2	Homo sapiens	26.917	1	1	74970	55644
8836	1	Q86XS8	Q86XS8	E3 ubiquitin-protein ligase RNF130	RNF130	Homo sapiens	46.406	1	1	25480	0
8837	1	Q86YL7	Q86YL7	Podoplanin	PDPN	Homo sapiens	16.696	1	1	182538	15884
8838	1	Q86YM7	Q86YM7	Homer protein homolog 1	HOMER1	Homo sapiens	40.275	1	1	23479	0
8839	1	Q86YN1	Q86YN1	Dolichyldiphosphatase 1	DOLPP1	Homo sapiens	27.029	1	1	124191	0
8840	1	Q86YW5	Q86YW5	Trem-like transcript 1 protein	TREML1	Homo sapiens	32.678	1	1	0	171262
8841	1	Q8IUE6	Q8IUE6	Histone H2A type 2-B	H2AC21	Homo sapiens	13.994	4	1	1947393	2085764
8842	1	Q8IUR5	Q8IUR5	Protein O-mannosyl-transferase TMTC1	TMTC1	Homo sapiens	98.85	1	1	5722	0
8843	1	Q8IUW3	Q8IUW3	Spermatogenesis-associated protein 2-like protein	SPATA2L	Homo sapiens	46.179	1	1	0	40154
8844	1	Q8IUX4	Q8IUX4	DNA dC->dU-editing enzyme APOBEC-3F	APOBEC3F	Homo sapiens	45.02	2	1	88737	50169
8845	1	Q8IV03	Q8IV03	Leucine rich adaptor protein 1-like	LURAP1L	Homo sapiens	24.413	1	1	65424	0
8846	1	Q8IVN3	Q8IVN3	Musculoskeletal embryonic nuclear protein 1	MUSTN1	Homo sapiens	8.91	1	1	25196	0

8847	1	Q8IWV1	Q8IWV1	Lymphocyte transmembrane adapter 1	LAX1	Homo sapiens	44.085	1	1	21018	0
8848	1	Q8IXH6	Q8IXH6	Tumor protein p53-inducible nuclear protein 2	TP53INP2	Homo sapiens	23.979	1	1	145027	0
8849	1	Q8IXQ3	Q8IXQ3	Uncharacterized protein C9orf40	C9orf40	Homo sapiens	21.064	1	1	520804	0
8850	1	Q8IXS8	Q8IXS8	Hyccin 2	HYCC2	Homo sapiens	58.646	1	1	58608	60269
8851	1	Q8IXW5	Q8IXW5	Putative RNA polymerase II subunit B1 CTD phosphatase RPAP2	RPAP2	Homo sapiens	69.509	1	1	252350	12127
8852	1	Q8IY31	Q8IY31	Intraflagellar transport protein 20 homolog	IFT20	Homo sapiens	15.279	1	1	9220	0
8853	1	Q8IY57	Q8IY57	YY1-associated factor 2	YAF2	Homo sapiens	19.9	1	1	0	35870
8854	1	Q8IZ57	Q8IZ57	Neurensin-1	NRSN1	Homo sapiens	21.473	1	1	47322	0
8855	1	Q8IZN3	Q8IZN3	Palmitoyltransferase ZDHHC14	ZDHHC14	Homo sapiens	53.39	1	1	60036	0
8856	1	Q8IZU9	Q8IZU9	Kin of IRRE-like protein 3	KIRREL3	Homo sapiens	85.258	1	1	23953	0
8857	1	Q8J025	Q8J025	Protein APCDD1	APCDD1	Homo sapiens	58.797	1	1	390152	0
8858	2	Q8N0W4;Q8NFZ3	Q8N0W4	Neuroligin-4, X-linked	NLGN4X	Homo sapiens	91.916	1	1	83457	0
8859	1	Q8N0Z8	Q8N0Z8	tRNA pseudouridine synthase-like 1	PUSL1	Homo sapiens	33.234	1	1	74407	0
8860	1	Q8N0Z9	Q8N0Z9	V-set and immunoglobulin domain-containing protein 10	VSIG10	Homo sapiens	59.218	1	1	37607	0
8861	1	Q8N118	Q8N118	Cytochrome P450 4X1	CYP4X1	Homo sapiens	58.876	1	1	38123	0
8862	1	Q8N135	Q8N135	Leucine-rich repeat LGI family member 4	LGI4	Homo sapiens	59.142	1	1	86433	0
8863	1	Q8N2G4	Q8N2G4	Ly6/PLAUR domain-containing protein 1	LYPD1	Homo sapiens	15.239	1	1	22863	0
8864	1	Q8N2G6	Q8N2G6	Zinc finger CCHC domain-containing protein 24	ZCCHC24	Homo sapiens	26.954	1	1	184513	0
8865	1	Q8N302	Q8N302	Angiogenic factor with G patch and FHA domains 1	AGGF1	Homo sapiens	80.98	1	1	161320	0
8866	1	Q8N307	Q8N307	Mucin-20	MUC20	Homo sapiens	71.983	1	1	44225	0
8867	1	Q8N371	Q8N371	Bifunctional peptidase and arginyl-hydroxylase JMJD5	KDM8	Homo sapiens	47.269	1	1	93636	0
8868	1	Q8N4C7	Q8N4C7	Syntaxin-19	STX19	Homo sapiens	34.323	1	1	84756	0
8869	1	Q8N4S0	Q8N4S0	Coiled-coil domain-containing protein 82	CCDC82	Homo sapiens	64.002	1	1	40200	0
8870	1	Q8N4V1	Q8N4V1	ER membrane protein complex subunit 5	MMGT1	Homo sapiens	14.686	1	1	604024	103018
8871	1	Q8N4Z0	Q8N4Z0	Ras-related protein Rab-42	RAB42	Homo sapiens	24.134	1	1	0	52597
8872	1	Q8N511	Q8N511	Transmembrane protein 199	TMEM199	Homo sapiens	23.128	1	1	213628	0
8873	1	Q8N567	Q8N567	Zinc finger CCHC domain-containing protein 9	ZCCHC9	Homo sapiens	30.478	1	1	96450	27647
8874	1	Q8N5F7	Q8N5F7	NF-kappa-B-activating protein	NKAP	Homo sapiens	47.138	3	1	134318	137747
8875	1	Q8N5I4	Q8N5I4	Dehydrogenase/reductase SDR family member on chromosome X	DHRSX	Homo sapiens	36.444	1	1	88737	100174
8876	1	Q8N5Y2	Q8N5Y2	Male-specific lethal 3 homolog	MSL3	Homo sapiens	59.825	2	1	12089	14251
8877	1	Q8N6L1	Q8N6L1	Keratinocyte-associated protein 2	KRTCAP2	Homo sapiens	14.678	1	1	42638	14573
8878	1	Q8N6N6	Q8N6N6	Protein NATD1	NATD1	Homo sapiens	13.03	1	1	0	65007
8879	1	Q8N6Q8	Q8N6Q8	Probable methyltransferase-like protein 25	METTL25	Homo sapiens	68.212	1	1	57798	31795
8880	1	Q8N8L6	Q8N8L6	ADP-ribosylation factor-like protein 10	ARL10	Homo sapiens	27.459	1	1	46163	0
8881	1	Q8N8Q9	Q8N8Q9	Magnesium transporter NIPA2	NIPA2	Homo sapiens	39.186	1	1	17463	0
8882	1	Q8N999	Q8N999	Uncharacterized protein C12orf29	C12orf29	Homo sapiens	37.49	1	1	102191	0
8883	1	Q8NAA4	Q8NAA4	Protein Atg16l2	ATG16L2	Homo sapiens	68.999	1	1	11205	0
8884	1	Q8NAF0	Q8NAF0	Zinc finger protein 579	ZNF579	Homo sapiens	60.51	1	1	2079903	0
8885	1	Q8NB15	Q8NB15	Zinc finger protein 511	ZNF511	Homo sapiens	28.265	1	1	75883	0
8886	1	Q8NB25	Q8NB25	Protein FAM184A	FAM184A	Homo sapiens	132.967	1	1	0	233311
8887	1	Q8NBA8	Q8NBA8	tRNA-uridine aminocarboxypropyltransferase 2	DTWD2	Homo sapiens	33.416	1	1	128753	0
8888	1	Q8NBI5	Q8NBI5	Equilibrative nucleobase transporter 1	SLC43A3	Homo sapiens	54.527	1	1	216615	173085
8889	1	Q8NBJ9	Q8NBJ9	SID1 transmembrane family member 2	SIDT2	Homo sapiens	94.456	1	1	133233	95088
8890	1	Q8NBV4	Q8NBV4	Inactive phospholipid phosphatase 7	PLPP7	Homo sapiens	29.447	1	1	43241	0
8891	1	Q8NC44	Q8NC44	Reticulophagy regulator 2	RETREG2	Homo sapiens	57.831	1	1	183697	44420
8892	1	Q8NC54	Q8NC54	Keratinocyte-associated transmembrane protein 2	KCT2	Homo sapiens	29.231	1	1	81878	82498
8893	1	Q8NCJ5	Q8NCJ5	SPRY domain-containing protein 3	SPRYD3	Homo sapiens	49.691	1	1	0	59843
8894	1	Q8NCS4	Q8NCS4	Transmembrane protein 35B	TMEM35B	Homo sapiens	16.883	1	1	57842	0

8895	1	Q8NDF8	Q8NDF8	Terminal nucleotidyltransferase 4B	TENT4B	Homo sapiens	63.266	1	1	38419	0
8896	1	Q8NDH6	Q8NDH6	Islet cell autoantigen 1-like protein	ICA1L	Homo sapiens	54.409	1	1	34888	0
8897	1	Q8NDX6	Q8NDX6	Zinc finger protein 740	ZNF740	Homo sapiens	21.858	1	1	32708	0
8898	1	Q8NEQ5	Q8NEQ5	Transmembrane protein C1orf162	C1orf162	Homo sapiens	16.885	1	1	0	122713
8899	1	Q8NEZ4	Q8NEZ4	Histone-lysine N-methyltransferase 2C	KMT2C	Homo sapiens	541.379	1	1	8840	0
8900	1	Q8NFJ5	Q8NFJ5	Retinoic acid-induced protein 3	GPRC5A	Homo sapiens	40.252	1	1	135131	291463
8901	1	Q8NFJ9	Q8NFJ9	Bardet-Biedl syndrome 1 protein	BBS1	Homo sapiens	65.082	1	1	40773	0
8902	1	Q8NFP7	Q8NFP7	Diphosphoinositol polyphosphate phosphohydrolase 3-alpha	NUDT10	Homo sapiens	18.5	1	1	82102	0
8903	1	Q8NHM5	Q8NHM5	Lysine-specific demethylase 2B	KDM2B	Homo sapiens	152.618	1	1	21193	15002
8904	1	Q8NHS3	Q8NHS3	Major facilitator superfamily domain-containing protein 8	MFSD8	Homo sapiens	57.628	1	1	0	41571
8905	1	Q8NHY2	Q8NHY2	E3 ubiquitin-protein ligase COP1	COP1	Homo sapiens	80.475	1	1	100437	66826
8906	1	Q8NHZ8	Q8NHZ8	Anaphase-promoting complex subunit CDC26	CDC26	Homo sapiens	9.777	1	1	303632	0
8907	1	Q8TAC1	Q8TAC1	Rieske domain-containing protein	RFESD	Homo sapiens	17.761	1	1	82376	77992
8908	1	Q8TAC9	Q8TAC9	Secretory carrier-associated membrane protein 5	SCAMP5	Homo sapiens	26.106	1	1	98424	0
8909	1	Q8TAV0	Q8TAV0	Protein FAM76A	FAM76A	Homo sapiens	35.05	1	1	63671	35354
8910	1	Q8TBF5	Q8TBF5	Phosphatidylinositol-glycan biosynthesis class X protein	PIGX	Homo sapiens	28.789	1	1	164432	104219
8911	1	Q8TBM7	Q8TBM7	Transmembrane protein 254	TMEM254	Homo sapiens	14.24	1	1	337502	132352
8912	1	Q8TBQ9	Q8TBQ9	Protein kish-A	TMEM167A	Homo sapiens	8.057	1	1	190453	236423
8913	1	Q8TBR7	Q8TBR7	TLC domain-containing protein 3A	TLCD3A	Homo sapiens	29.382	1	1	30470	0
8914	1	Q8TBZ6	Q8TBZ6	tRNA methyltransferase 10 homolog A	TRMT10A	Homo sapiens	39.719	1	1	33486	0
8915	1	Q8TC44	Q8TC44	POC1 centriolar protein homolog B	POC1B	Homo sapiens	53.668	1	1	445420	0
8916	1	Q8TC71	Q8TC71	Mitochondria-eating protein	SPATA18	Homo sapiens	61.108	1	1	12267	0
8917	1	Q8TCD1	Q8TCD1	UPF0729 protein C18orf32	C18orf32	Homo sapiens	8.666	1	1	273163	308872
8918	1	Q8TCE6	Q8TCE6	DENN domain-containing protein 10	DENND10	Homo sapiens	40.514	9	1	77878	75381
8919	1	Q8TD46	Q8TD46	Cell surface glycoprotein CD200 receptor 1	CD200R1	Homo sapiens	36.606	1	1	23133	230970
8920	1	Q8TD84	Q8TD84	Cell adhesion molecule DSCAML1	DSCAML1	Homo sapiens	224.466	1	1	43830	0
8921	1	Q8TDS5	Q8TDS5	Oxoeicosanoid receptor 1	OXER1	Homo sapiens	45.811	1	1	0	134283
8922	1	Q8TE82	Q8TE82	SH3 domain and tetratricopeptide repeat-containing protein 1	SH3TC1	Homo sapiens	146.961	1	1	26330	0
8923	1	Q8TEC5	Q8TEC5	E3 ubiquitin-protein ligase SH3RF2	SH3RF2	Homo sapiens	79.32	1	1	133108	0
8924	1	Q8TED4	Q8TED4	Glucose-6-phosphate exchanger SLC37A2	SLC37A2	Homo sapiens	54.436	1	1	49375	18022
8925	1	Q8TER0	Q8TER0	Sushi, nidogen and EGF-like domain-containing protein 1	SNED1	Homo sapiens	152.207	1	1	52212	0
8926	1	Q8TF47	Q8TF47	Zinc finger protein 90 homolog	ZFP90	Homo sapiens	73.033	1	1	29143	0
8927	1	Q8TF64	Q8TF64	PDZ domain-containing protein GIPC3	GIPC3	Homo sapiens	33.983	1	1	272404	0
8928	1	Q8WU67	Q8WU67	Phospholipase ABHD3	ABHD3	Homo sapiens	46.009	1	1	131467	131659
8929	1	Q8WUH1	Q8WUH1	Protein Churchill	CHURC1	Homo sapiens	12.886	1	1	533647	316964
8930	1	Q8WUM9	Q8WUM9	Sodium-dependent phosphate transporter 1	SLC20A1	Homo sapiens	73.698	1	1	0	77546
8931	1	Q8WV74	Q8WV74	Mitochondrial coenzyme A diphosphatase NUDT8	NUDT8	Homo sapiens	25.369	1	1	287222	0
8932	1	Q8WV83	Q8WV83	Solute carrier family 35 member F5	SLC35F5	Homo sapiens	58.89	1	1	126772	0
8933	1	Q8WVB3	Q8WVB3	Hexosaminidase D	HEXD	Homo sapiens	53.793	1	1	131474	0
8934	1	Q8WVN8	Q8WVN8	Ubiquitin-conjugating enzyme E2 Q2	UBE2Q2	Homo sapiens	42.817	1	1	37940	0
8935	1	Q8WVP7	Q8WVP7	Limb region 1 protein homolog	LMBR1	Homo sapiens	55.097	1	1	91656033	0
8936	1	Q8WW43	Q8WW43	Gamma-secretase subunit APH-1B	APH1B	Homo sapiens	28.461	1	1	0	125464
8937	1	Q8WWK9	Q8WWK9	Cytoskeleton-associated protein 2	CKAP2	Homo sapiens	76.989	1	1	14799	0
8938	1	Q8WWQ2	Q8WWQ2	Inactive heparanase-2	HPSE2	Homo sapiens	66.596	1	1	16059	0
8939	1	Q8WWU7	Q8WWU7	Intelectin-2	ITLN2	Homo sapiens	36.211	1	1	140661	0
8940	1	Q8WWY8	Q8WWY8	Lipase member H	LIPH	Homo sapiens	50.863	1	1	44035	0
8941	1	Q8WXE9	Q8WXE9	Stonin-2	STON2	Homo sapiens	101.166	1	1	48573	0
8942	1	Q8WXI8	Q8WXI8	C-type lectin domain family 4 member D	CLEC4D	Homo sapiens	24.707	1	1	0	825012

8943	1	Q8WXR4	Q8WXR4	Myosin-IIIb	MYO3B	Homo sapiens	151.831	1	1	235163	61942
8944	1	Q8WYQ3	Q8WYQ3	Coiled-coil-helix-coiled-coil-helix domain-containing protein 10, mitochondrial	CHCHD10	Homo sapiens	14.15	1	1	190745	0
8945	1	Q8WZA2	Q8WZA2	Rap guanine nucleotide exchange factor 4	RAPGEF4	Homo sapiens	115.521	1	1	23159	0
8946	1	Q92187	Q92187	CMP-N-acetylneuraminate-poly-alpha-2,8-sialyltransferase	ST8SIA4	Homo sapiens	41.295	1	1	0	79616
8947	1	Q92481	Q92481	Transcription factor AP-2-beta	TFAP2B	Homo sapiens	50.474	1	1	0	51852
8948	1	Q92521	Q92521	GPI mannosyltransferase 3	PIGB	Homo sapiens	65.056	1	1	0	56594
8949	1	Q92536	Q92536	Y+L amino acid transporter 2	SLC7A6	Homo sapiens	56.828	1	1	61727	0
8950	1	Q92537	Q92537	Sushi domain-containing protein 6	SUSD6	Homo sapiens	32.091	1	1	57375	145464
8951	1	Q92561	Q92561	Phytanoyl-CoA hydroxylase-interacting protein	PHYHIP	Homo sapiens	37.574	1	1	292473	86713
8952	1	Q92569	Q92569	Phosphatidylinositol 3-kinase regulatory subunit gamma	PIK3R3	Homo sapiens	54.449	1	1	0	112831
8953	1	Q92622	Q92622	Run domain Beclin-1-interacting and cysteine-rich domain-containing protein	RUBCN	Homo sapiens	108.622	1	1	25966	20241
8954	1	Q92681	Q92681	Regulatory solute carrier protein family 1 member 1	RSC1A1	Homo sapiens	66.79	1	1	10924	0
8955	1	Q92784	Q92784	Zinc finger protein DPF3	DPF3	Homo sapiens	43.085	1	1	46271	0
8956	1	Q92908	Q92908	Transcription factor GATA-6	GATA6	Homo sapiens	60.031	1	1	48247	0
8957	1	Q92918	Q92918	Mitogen-activated protein kinase kinase kinase kinase 1	MAP4K1	Homo sapiens	91.297	1	1	83136	75251
8958	1	Q92985	Q92985	Interferon regulatory factor 7	IRF7	Homo sapiens	54.277	1	1	18966	56182
8959	1	Q92989	Q92989	Polyribonucleotide 5'-hydroxyl-kinase Clp1	CLP1	Homo sapiens	47.646	1	1	161823	0
8960	1	Q93073	Q93073	Selenocysteine insertion sequence-binding protein 2-like	SECISBP2L	Homo sapiens	121.778	1	1	62043	26091
8961	1	Q93075	Q93075	Putative deoxyribonuclease TATDN2	TATDN2	Homo sapiens	85.024	1	1	20687	0
8962	1	Q93097	Q93097	Protein Wnt-2b	WNT2B	Homo sapiens	43.772	1	1	32890	0
8963	1	Q969F1	Q969F1	General transcription factor 3C polypeptide 6	GTF3C6	Homo sapiens	24.05	1	1	176557	57659
8964	1	Q969K3	Q969K3	E3 ubiquitin-protein ligase RNF34	RNF34	Homo sapiens	41.64	1	1	19351	0
8965	1	Q969L2	Q969L2	Protein MAL2	MAL2	Homo sapiens	19.126	1	1	1448900	393753
8966	1	Q969Q0	Q969Q0	60S ribosomal protein L36a-like	RPL36AL	Homo sapiens	12.467	3	1	43235	0
8967	1	Q969R5	Q969R5	Lethal(3)malignant brain tumor-like protein 2	L3MBTL2	Homo sapiens	79.113	1	1	44989	0
8968	1	Q969S8	Q969S8	Polyamine deacetylase HDAC10	HDAC10	Homo sapiens	71.446	1	1	43689	0
8969	1	Q969Y2	Q969Y2	tRNA modification GTPase GTPBP3, mitochondrial	GTPBP3	Homo sapiens	52.058	1	1	109540	0
8970	1	Q96A25	Q96A25	Transmembrane protein 106A	TMEM106A	Homo sapiens	28.92	1	1	92184	94462
8971	1	Q96A58	Q96A58	Ras-related and estrogen-regulated growth inhibitor	RERG	Homo sapiens	22.609	1	1	154917	0
8972	1	Q96AH0	Q96AH0	SOSS complex subunit B2	NABP1	Homo sapiens	22.422	1	1	0	78552
8973	1	Q96AJ9	Q96AJ9	Vesicle transport through interaction with t-SNAREs homolog 1A	VTI1A	Homo sapiens	25.218	1	1	18374	0
8974	1	Q96B96	Q96B96	Lipid droplet assembly factor 1	LDAF1	Homo sapiens	17.522	1	1	162930	167327
8975	1	Q96BM9	Q96BM9	ADP-ribosylation factor-like protein 8A	ARL8A	Homo sapiens	21.417	1	1	767120	829507
8976	1	Q96CG3	Q96CG3	TRAF-interacting protein with FHA domain-containing protein A	TIFA	Homo sapiens	21.445	1	1	0	90182
8977	1	Q96CW6	Q96CW6	Probable RNA polymerase II nuclear localization protein SLC7A6OS	SLC7A6OS	Homo sapiens	35.029	1	1	113601	0
8978	1	Q96D09	Q96D09	G-protein coupled receptor-associated sorting protein 2	GPRASP2	Homo sapiens	93.775	1	1	100509	0
8979	1	Q96D31	Q96D31	Calcium release-activated calcium channel protein 1	ORAI1	Homo sapiens	32.67	1	1	70345	39887
8980	1	Q96DD7	Q96DD7	Protein shisa-4	SHISA4	Homo sapiens	21.522	1	1	48659	0
8981	1	Q96DX5	Q96DX5	Ankyrin repeat and SOCS box protein 9	ASB9	Homo sapiens	31.855	1	1	28334	0
8982	1	Q96DZ9	Q96DZ9	CKLF-like MARVEL transmembrane domain-containing protein 5	CMTM5	Homo sapiens	24.654	1	1	287914	199003
8983	1	Q96E09	Q96E09	PPP2R1A-PPP2R2A-interacting phosphatase regulator 1	PABIR1	Homo sapiens	30.529	1	1	87264	62506
8984	1	Q96E29	Q96E29	Transcription termination factor 3, mitochondrial	MTERF3	Homo sapiens	47.972	1	1	131976	0
8985	1	Q96EF0	Q96EF0	Myotubularin-related protein 8	MTMR8	Homo sapiens	78.922	1	1	49167	0
8986	1	Q96EK4	Q96EK4	THAP domain-containing protein 11	THAP11	Homo sapiens	34.455	1	1	88102	0
8987	1	Q96EP1	Q96EP1	E3 ubiquitin-protein ligase CHFR	CHFR	Homo sapiens	73.384	1	1	19960	0
8988	1	Q96EP9	Q96EP9	Sodium/bile acid cotransporter 4	SLC10A4	Homo sapiens	46.505	1	1	23896	0

8989	1	Q96ER3	Q96ER3	Protein SAAL1	SAAL1	Homo sapiens	53.558	1	1	33826	0
8990	1	Q96EU6	Q96EU6	Ribosomal RNA processing protein 36 homolog	RRP36	Homo sapiens	29.822	1	1	13497	0
8991	1	Q96F10	Q96F10	Thialysine N-epsilon-acetyltransferase	SAT2	Homo sapiens	19.155	1	1	105165	111490
8992	1	Q96FE7	Q96FE7	Phosphoinositide-3-kinase-interacting protein 1	PIK3IP1	Homo sapiens	28.246	1	1	78888	0
8993	1	Q96FJ2	Q96FJ2	Dynein light chain 2, cytoplasmic	DYNLL2	Homo sapiens	10.348	1	1	127493	0
8994	1	Q96FT9	Q96FT9	Intraflagellar transport protein 43 homolog	IFT43	Homo sapiens	23.53	1	1	45966	0
8995	1	Q96FX2	Q96FX2	Diphthamide biosynthesis protein 3	DPH3	Homo sapiens	9.238	1	1	0	13670
8996	1	Q96G01	Q96G01	Protein bicaudal D homolog 1	BICD1	Homo sapiens	110.751	1	1	32218	0
8997	1	Q96GC9	Q96GC9	Vacuole membrane protein 1	VMP1	Homo sapiens	46.237	1	1	246468	180029
8998	1	Q96GD4	Q96GD4	Aurora kinase B	AURKB	Homo sapiens	39.311	1	1	45608	0
8999	1	Q96GJ1	Q96GJ1	tRNA (uracil-5-)-methyltransferase homolog B	TRMT2B	Homo sapiens	56.477	1	1	24291	0
9000	1	Q96GL9	Q96GL9	Protein FAM163A	FAM163A	Homo sapiens	17.64	1	1	20702	0
9001	1	Q96GR4	Q96GR4	Palmitoyltransferase ZDHHC12	ZDHHC12	Homo sapiens	30.811	1	1	10747	0
9002	1	Q96GX2	Q96GX2	Ataxin-7-like protein 3B	ATXN7L3B	Homo sapiens	10.771	1	1	254692	502753
9003	1	Q96GZ6	Q96GZ6	Solute carrier family 41 member 3	SLC41A3	Homo sapiens	54.766	1	1	76374	0
9004	1	Q96H55	Q96H55	Unconventional myosin-XIX	MYO19	Homo sapiens	109.138	1	1	102745	0
9005	1	Q96HA7	Q96HA7	Tonsoku-like protein	TONSL	Homo sapiens	150.932	1	1	57007	59673
9006	1	Q96HH9	Q96HH9	GRAM domain-containing protein 2B	GRAMD2B	Homo sapiens	47.869	1	1	163663	39935
9007	1	Q96HJ5	Q96HJ5	Membrane-spanning 4-domains subfamily A member 3	MS4A3	Homo sapiens	22.934	1	1	0	297517
9008	1	Q96HJ9	Q96HJ9	Protein FMC1 homolog	FMC1	Homo sapiens	12.749	1	1	364547	127052
9009	1	Q96HP8	Q96HP8	Transmembrane protein 176A	TMEM176A	Homo sapiens	26.118	1	1	43538	0
9010	1	Q96HR9	Q96HR9	Receptor expression-enhancing protein 6	REEP6	Homo sapiens	23.417	1	1	24329	0
9011	1	Q96HU8	Q96HU8	GTP-binding protein Di-Ras2	DIRAS2	Homo sapiens	22.486	1	1	5903	0
9012	1	Q96I34	Q96I34	Protein phosphatase 1 regulatory subunit 16A	PPP1R16A	Homo sapiens	57.813	1	1	133569	0
9013	1	Q96IP4	Q96IP4	Terminal nucleotidyltransferase 5A	TENT5A	Homo sapiens	49.667	1	1	24890	0
9014	1	Q96J92	Q96J92	Serine/threonine-protein kinase WNK4	WNK4	Homo sapiens	134.74	1	1	94912	0
9015	1	Q96KF7	Q96KF7	Small integral membrane protein 8	SMIM8	Homo sapiens	11.058	1	1	55946	0
9016	1	Q96KJ9	Q96KJ9	Cytochrome c oxidase subunit 4 isoform 2, mitochondrial	COX4I2	Homo sapiens	20.009	1	1	159354	0
9017	1	Q96KR6	Q96KR6	Protein FAM210B, mitochondrial	FAM210B	Homo sapiens	20.422	1	1	30063	0
9018	1	Q96L34	Q96L34	MAP/microtubule affinity-regulating kinase 4	MARK4	Homo sapiens	82.519	1	1	55999	0
9019	1	Q96LT9	Q96LT9	RNA-binding region-containing protein 3	RNPC3	Homo sapiens	58.575	1	1	85691	0
9020	1	Q96MS3	Q96MS3	Glycosyltransferase 1 domain-containing protein 1	GLT1D1	Homo sapiens	38.507	1	1	0	35717
9021	1	Q96MY1	Q96MY1	Nucleolar protein 4-like	NOL4L	Homo sapiens	47.213	2	1	30290	0
9022	1	Q96N06	Q96N06	Spermatogenesis-associated protein 33	SPATA33	Homo sapiens	15.461	1	1	38264	0
9023	1	Q96N11	Q96N11	Integrator complex subunit 15	INTS15	Homo sapiens	50.046	1	1	26567	8675
9024	1	Q96NL6	Q96NL6	Sodium channel and clathrin linker 1	SCLT1	Homo sapiens	80.912	1	1	18874	0
9025	1	Q96NT1	Q96NT1	Nucleosome assembly protein 1-like 5	NAP1L5	Homo sapiens	19.592	1	1	299634	58125
9026	1	Q96NY8	Q96NY8	Nectin-4	NECTIN4	Homo sapiens	55.453	1	1	0	151118
9027	1	Q96NZ9	Q96NZ9	Proline-rich acidic protein 1	PRAP1	Homo sapiens	17.206	1	1	0	242308
9028	1	Q96PD2	Q96PD2	Discoidin, CUB and LCCL domain-containing protein 2	DCBLD2	Homo sapiens	85.033	1	1	114190	0
9029	1	Q96Q80	Q96Q80	Derlin-3	DERL3	Homo sapiens	26.677	1	1	135356	0
9030	1	Q96QD8	Q96QD8	Sodium-coupled neutral amino acid symporter 2	SLC38A2	Homo sapiens	56.024	1	1	0	45658
9031	1	Q96S06	Q96S06	Lipase maturation factor 1	LMF1	Homo sapiens	64.872	1	1	65969	0
9032	1	Q96SJ8	Q96SJ8	Tetraspanin-18	TSPAN18	Homo sapiens	27.711	1	1	138915	0
9033	1	Q96SL1	Q96SL1	Solute carrier family 49 member 4	SLC49A4	Homo sapiens	52.089	1	1	0	33462
9034	1	Q96T21	Q96T21	Selenocysteine insertion sequence-binding protein 2	SECISBP2	Homo sapiens	95.463	1	1	103955	0
9035	1	Q99259	Q99259	Glutamate decarboxylase 1	GAD1	Homo sapiens	66.897	1	1	0	36789
9036	1	Q99547	Q99547	M-phase phosphoprotein 6	MPHOSPH6	Homo sapiens	19.024	1	1	29550	0

9037	1	Q99574	Q99574	Neuroserpin	SERPINI1	Homo sapiens	46.427	1	1	48495	0
9038	1	Q99595	Q99595	Mitochondrial import inner membrane translocase subunit Tim17-A	TIMM17A	Homo sapiens	18.024	1	1	17895	0
9039	1	Q99612	Q99612	Krueppel-like factor 6	KLF6	Homo sapiens	31.865	1	1	204430	38494
9040	1	Q99613	Q99613	Eukaryotic translation initiation factor 3 subunit C	EIF3C	Homo sapiens	105.345	29	1	1061050	189796
9041	1	Q99643	Q99643	Succinate dehydrogenase cytochrome b560 subunit, mitochondrial	SDHC	Homo sapiens	18.61	1	1	44962	0
9042	1	Q99720	Q99720	Sigma non-opioid intracellular receptor 1	SIGMAR1	Homo sapiens	25.127	1	1	824908	488601
9043	1	Q99766	Q99766	ATP synthase subunit s, mitochondrial	DMAC2L	Homo sapiens	24.866	1	1	190189	39003
9044	1	Q99784	Q99784	Noelin	OLFM1	Homo sapiens	55.343	1	1	24638	28137
9045	1	Q99946	Q99946	Proline-rich transmembrane protein 1	PRRT1	Homo sapiens	31.427	1	1	16855	0
9046	1	Q99962	Q99962	Endophilin-A1	SH3GL2	Homo sapiens	39.96	1	1	31412	0
9047	1	Q9BQ15	Q9BQ15	SOSS complex subunit B1	NABP2	Homo sapiens	22.339	1	1	55371	0
9048	1	Q9BQ24	Q9BQ24	Zinc finger FYVE domain-containing protein 21	ZFYVE21	Homo sapiens	26.504	1	1	162399	0
9049	1	Q9BQ51	Q9BQ51	Programmed cell death 1 ligand 2	PDCD1LG2	Homo sapiens	30.957	1	1	0	111758
9050	1	Q9BQ75	Q9BQ75	Protein CMSS1	CMSS1	Homo sapiens	31.885	1	1	35333	0
9051	1	Q9BQC6	Q9BQC6	Ribosomal protein 63, mitochondrial	MRPL57	Homo sapiens	12.265	1	1	782010	191830
9052	1	Q9BQE3	Q9BQE3	Tubulin alpha-1C chain	TUBA1C	Homo sapiens	49.895	2	1	178808	200283
9053	1	Q9BQJ4	Q9BQJ4	Transmembrane protein 47	TMEM47	Homo sapiens	19.995	1	1	68588	0
9054	1	Q9BQQ3	Q9BQQ3	Golgi reassembly-stacking protein 1	GORASP1	Homo sapiens	46.483	1	1	109943	25616
9055	1	Q9BR01	Q9BR01	Sulfotransferase 4A1	SULT4A1	Homo sapiens	33.085	1	1	23640	0
9056	1	Q9BRL6	Q9BRL6	Serine/arginine-rich splicing factor 8	SRSF8	Homo sapiens	32.287	1	1	35134	0
9057	1	Q9BRP1	Q9BRP1	Programmed cell death protein 2-like	PDCD2L	Homo sapiens	39.417	1	1	65718	0
9058	1	Q9BRS8	Q9BRS8	La-related protein 6	LARP6	Homo sapiens	54.738	1	1	14260	0
9059	1	Q9BRT9	Q9BRT9	DNA replication complex GINS protein SLD5	GINS4	Homo sapiens	26.047	1	1	110946	0
9060	1	Q9BRU2	Q9BRU2	Transcription elongation factor A protein-like 7	TCEAL7	Homo sapiens	12.323	1	1	63730	0
9061	1	Q9BT17	Q9BT17	Mitochondrial ribosome-associated GTPase 1	MTG1	Homo sapiens	37.236	1	1	35785	0
9062	1	Q9BT25	Q9BT25	HAUS augmin-like complex subunit 8	HAUS8	Homo sapiens	44.857	1	1	24184	0
9063	1	Q9BT88	Q9BT88	Synaptotagmin-11	SYT11	Homo sapiens	48.297	1	1	36584	0
9064	1	Q9BTD8	Q9BTD8	RNA-binding protein 42	RBM42	Homo sapiens	50.414	1	1	174552	51879
9065	1	Q9BTL3	Q9BTL3	RNA guanine-N7 methyltransferase activating subunit	RAMAC	Homo sapiens	14.378	2	1	148486	0
9066	1	Q9BTT4	Q9BTT4	Mediator of RNA polymerase II transcription subunit 10	MED10	Homo sapiens	15.688	1	1	54592	22266
9067	1	Q9BTZ2	Q9BTZ2	Dehydrogenase/reductase SDR family member 4	DHRS4	Homo sapiens	29.537	3	1	118803	25707
9068	1	Q9BU68	Q9BU68	Proline-rich protein 15-like protein	PRR15L	Homo sapiens	11.704	1	1	164905	0
9069	1	Q9BUF7	Q9BUF7	Protein crumbs homolog 3	CRB3	Homo sapiens	12.854	1	1	0	19067
9070	1	Q9BUK0	Q9BUK0	Coiled-coil-helix-coiled-coil-helix domain-containing protein 7	CHCHD7	Homo sapiens	10.096	1	1	207754	32149
9071	1	Q9BUT9	Q9BUT9	MAPK regulated corepressor interacting protein 2	MCRIP2	Homo sapiens	17.828	1	1	51472	0
9072	1	Q9BUY5	Q9BUY5	Zinc finger protein 426	ZNF426	Homo sapiens	63.108	1	1	19463	0
9073	1	Q9BV68	Q9BV68	E3 ubiquitin-protein ligase RNF126	RNF126	Homo sapiens	33.861	1	1	197723	237577
9074	1	Q9BV81	Q9BV81	ER membrane protein complex subunit 6	EMC6	Homo sapiens	12.015	1	1	228800	265875
9075	1	Q9BVA1	Q9BVA1	Tubulin beta-2B chain	TUBB2B	Homo sapiens	49.952	3	1	8456140	30814
9076	1	Q9BVM4	Q9BVM4	Gamma-glutamylaminecyclotransferase	GGACT	Homo sapiens	17.326	1	1	215608	110429
9077	1	Q9BW19	Q9BW19	Kinesin-like protein KIFC1	KIFC1	Homo sapiens	73.746	1	1	76969	0
9078	1	Q9BW60	Q9BW60	Elongation of very long chain fatty acids protein 1	ELOVL1	Homo sapiens	32.661	1	1	48063	67881
9079	1	Q9BW66	Q9BW66	Cyclin-dependent kinase 2-interacting protein	CINP	Homo sapiens	24.322	1	1	16222	0
9080	1	Q9BW71	Q9BW71	HIRA-interacting protein 3	HIRIP3	Homo sapiens	61.956	1	1	123778	0
9081	1	Q9BWD3	Q9BWD3	Retrotransposon Gag-like protein 8A	RTL8A	Homo sapiens	13.189	2	1	30462	0
9082	1	Q9BWW4	Q9BWW4	Single-stranded DNA-binding protein 3	SSBP3	Homo sapiens	40.421	1	1	229785	0
9083	1	Q9BXJ4	Q9BXJ4	Complement C1q tumor necrosis factor-related protein 3	C1QTNF3	Homo sapiens	26.993	1	1	109713	0
9084	1	Q9BXJ8	Q9BXJ8	Ion channel TACAN	TMEM120A	Homo sapiens	40.61	1	1	200552	313183

9085	1 Q9BXV9	Q9BXV9	EKC/KEOPS complex subunit GON7	GON7	Homo sapiens	10.858	1	1	64482	18553
9086	1 Q9BXW9	Q9BXW9	Fanconi anemia group D2 protein	FANCD2	Homo sapiens	164.13	1	1	34796	0
9087	1 Q9BYX7	Q9BYX7	Putative beta-actin-like protein 3	POTEKP	Homo sapiens	42.016	3	1	116598	52569
9088	1 Q9BYZ2	Q9BYZ2	L-lactate dehydrogenase A-like 6B	LDHAL6B	Homo sapiens	41.943	1	1	0	18758
9089	1 Q9C010	Q9C010	cAMP-dependent protein kinase inhibitor beta	PKIB	Homo sapiens	8.467	1	1	118061	0
9090	1 Q9C0C7	Q9C0C7	Activating molecule in BECN1-regulated autophagy protein 1	AMBRA1	Homo sapiens	142.507	1	1	26339	0
9091	1 Q9C0D9	Q9C0D9	Ethanolaminephosphotransferase 1	SELENOI	Homo sapiens	45.201	1	1	123165	0
9092	1 Q9GZP9	Q9GZP9	Derlin-2	DERL2	Homo sapiens	27.567	1	1	372518	347725
9093	1 Q9GZX3	Q9GZX3	Carbohydrate sulfotransferase 6	CHST6	Homo sapiens	44.099	1	1	11467	29332
9094	1 Q9H013	Q9H013	Disintegrin and metalloproteinase domain-containing protein 19	ADAM19	Homo sapiens	104.999	1	1	0	53142
9095	1 Q9H015	Q9H015	Solute carrier family 22 member 4	SLC22A4	Homo sapiens	62.155	1	1	0	33282
9096	1 Q9H063	Q9H063	Repressor of RNA polymerase III transcription MAF1 homolog	MAF1	Homo sapiens	28.77	1	1	267415	59445
9097	1 Q9H079	Q9H079	KATNB1-like protein 1	KATNBL1	Homo sapiens	34.765	1	1	28131	0
9098	1 Q9H081	Q9H081	Protein MIS12 homolog	MIS12	Homo sapiens	24.139	1	1	175441	105417
9099	1 Q9H091	Q9H091	Zinc finger MYND domain-containing protein 15	ZMYND15	Homo sapiens	81.86	1	1	65564	0
9100	1 Q9H0R3	Q9H0R3	Transmembrane protein 222	TMEM222	Homo sapiens	23.228	1	1	91135	0
9101	1 Q9H0R8	Q9H0R8	Gamma-aminobutyric acid receptor-associated protein-like 1	GABARAPL1	Homo sapiens	14.04	1	1	196968	0
9102	1 Q9H0Z9	Q9H0Z9	RNA-binding protein 38	RBM38	Homo sapiens	25.499	5	1	127324	0
9103	1 Q9H1C7	Q9H1C7	Cysteine-rich and transmembrane domain-containing protein 1	CYSTM1	Homo sapiens	10.63	1	1	230592	328766
9104	1 Q9H1J7	Q9H1J7	Protein Wnt-5b	WNT5B	Homo sapiens	40.324	1	1	157099	0
9105	1 Q9H1U9	Q9H1U9	Mitochondrial nicotinamide adenine dinucleotide transporter SLC25A51	SLC25A51	Homo sapiens	33.673	1	1	139407	0
9106	1 Q9H239	Q9H239	Matrix metalloproteinase-28	MMP28	Homo sapiens	58.937	1	1	17421	0
9107	1 Q9H2D1	Q9H2D1	Mitochondrial folate transporter/carrier	SLC25A32	Homo sapiens	35.406	1	1	55947	21107
9108	1 Q9H2W1	Q9H2W1	Membrane-spanning 4-domains subfamily A member 6A	MS4A6A	Homo sapiens	26.944	1	1	30928	0
9109	1 Q9H2X8	Q9H2X8	Interferon alpha-inducible protein 27-like protein 2	IFI27L2	Homo sapiens	12.407	1	1	60935	0
9110	1 Q9H361	Q9H361	Polyadenylate-binding protein 3	PABPC3	Homo sapiens	70.031	1	1	186060	0
9111	1 Q9H3S4	Q9H3S4	Thiamin pyrophosphokinase 1	TPK1	Homo sapiens	27.265	1	1	65099	42833
9112	1 Q9H3S5	Q9H3S5	GPI mannosyltransferase 1	PIGM	Homo sapiens	49.46	1	1	54597	16947
9113	1 Q9H492	Q9H492	Microtubule-associated proteins 1A/1B light chain 3A	MAP1LC3A	Homo sapiens	14.27	1	1	626359	0
9114	1 Q9H4A9	Q9H4A9	Dipeptidase 2	DPEP2	Homo sapiens	53.367	1	1	7389	14747
9115	1 Q9H4B0	Q9H4B0	tRNA N6-adenosine threonylcarbamoyltransferase, mitochondrial	OSGEPL1	Homo sapiens	45.122	1	1	0	39505
9116	1 Q9H4E5	Q9H4E5	Rho-related GTP-binding protein RhoJ	RHOJ	Homo sapiens	23.819	1	1	45435	0
9117	1 Q9H4X1	Q9H4X1	Regulator of cell cycle RGCC	RGCC	Homo sapiens	14.557	1	1	36754	0
9118	1 Q9H5J4	Q9H5J4	Elongation of very long chain fatty acids protein 6	ELOVL6	Homo sapiens	31.375	1	1	8282	0
9119	1 Q9H694	Q9H694	Protein bicaudal C homolog 1	BICC1	Homo sapiens	104.846	1	1	91982	0
9120	1 Q9H6F2	Q9H6F2	Trimeric intracellular cation channel type A	TMEM38A	Homo sapiens	33.263	1	1	4254	14377
9121	1 Q9H6L2	Q9H6L2	Transmembrane protein 231	TMEM231	Homo sapiens	36.058	1	1	104377	0
9122	1 Q9H6L5	Q9H6L5	Reticulophagy regulator 1	RETREG1	Homo sapiens	54.679	1	1	60089	0
9123	1 Q9H6P5	Q9H6P5	Threonine aspartase 1	TASP1	Homo sapiens	44.457	1	1	37175	0
9124	1 Q9H6R0	Q9H6R0	ATP-dependent RNA helicase DHX33	DHX33	Homo sapiens	78.875	1	1	21131	0
9125	1 Q9H6X4	Q9H6X4	Transmembrane protein 134	TMEM134	Homo sapiens	21.585	1	1	0	75753
9126	1 Q9H6X5	Q9H6X5	Uncharacterized protein C19orf44	C19orf44	Homo sapiens	71.342	1	1	44404	0
9127	1 Q9H756	Q9H756	Leucine-rich repeat-containing protein 19	LRRC19	Homo sapiens	42.336	1	1	97221	0
9128	1 Q9H7S9	Q9H7S9	Zinc finger protein 703	ZNF703	Homo sapiens	58.224	1	1	71233	0
9129	1 Q9H841	Q9H841	NIPA-like protein 2	NIPAL2	Homo sapiens	42.158	1	1	72414	0
9130	1 Q9H892	Q9H892	Tetratricopeptide repeat protein 12	TTC12	Homo sapiens	78.757	1	1	15507	0
9131	1 Q9H920	Q9H920	E3 ubiquitin ligase RNF121	RNF121	Homo sapiens	37.883	1	1	44264	0
9132	1 Q9H930	Q9H930	Nuclear body protein SP140-like protein	SP140L	Homo sapiens	67.006	2	1	0	36536

9133	1	Q9H992	Q9H992	E3 ubiquitin-protein ligase MARCHF7	MARCHF7	Homo sapiens	78.052	1	1	16371	0
9134	1	Q9H9S3	Q9H9S3	Protein transport protein Sec61 subunit alpha isoform 2	SEC61A2	Homo sapiens	52.244	1	1	41917	0
9135	1	Q9H9S5	Q9H9S5	Ribitol 5-phosphate transferase FKRP	FKRP	Homo sapiens	54.569	1	1	71291	0
9136	1	Q9H9V9	Q9H9V9	2-oxoglutarate and iron-dependent oxygenase JMJD4	JMJD4	Homo sapiens	52.493	1	1	0	169646
9137	1	Q9HAC7	Q9HAC7	Succinate--hydroxymethylglutarate CoA-transferase	SUGCT	Homo sapiens	48.463	1	1	228516	0
9138	1	Q9HAF1	Q9HAF1	Chromatin modification-related protein MEAF6	MEAF6	Homo sapiens	21.634	1	1	141567	0
9139	1	Q9HAT8	Q9HAT8	E3 ubiquitin-protein ligase pellino homolog 2	PELI2	Homo sapiens	46.435	1	1	27628	0
9140	1	Q9HAW9	Q9HAW9	UDP-glucuronosyltransferase 1A8	UGT1A8	Homo sapiens	59.742	2	1	19423	0
9141	1	Q9HB20	Q9HB20	Pleckstrin homology domain-containing family A member 3	PLEKHA3	Homo sapiens	33.859	1	1	84302	30611
9142	1	Q9HB66	Q9HB66	Alternative protein MKKS	MKKS	Homo sapiens	7.26	1	1	219032	149660
9143	1	Q9HBE1	Q9HBE1	POZ-, AT hook-, and zinc finger-containing protein 1	PATZ1	Homo sapiens	74.059	1	1	29341	0
9144	1	Q9HBU6	Q9HBU6	Ethanolamine kinase 1	ETNK1	Homo sapiens	50.969	1	1	86085	0
9145	1	Q9HC44	Q9HC44	Vasculin-like protein 1	GPBP1L1	Homo sapiens	52.303	1	1	16557	0
9146	1	Q9HCE3	Q9HCE3	Zinc finger protein 532	ZNF532	Homo sapiens	141.699	1	1	12686	0
9147	1	Q9HCE7	Q9HCE7	E3 ubiquitin-protein ligase SMURF1	SMURF1	Homo sapiens	86.116	1	1	581140	0
9148	1	Q9HCL2	Q9HCL2	Glycerol-3-phosphate acyltransferase 1, mitochondrial	GPAM	Homo sapiens	93.795	1	1	63926	21440
9149	1	Q9NP90	Q9NP90	Ras-related protein Rab-9B	RAB9B	Homo sapiens	22.719	1	1	51006	0
9150	1	Q9NPD8	Q9NPD8	Ubiquitin-conjugating enzyme E2 T	UBE2T	Homo sapiens	22.52	1	1	231419	0
9151	1	Q9NQ84	Q9NQ84	G-protein coupled receptor family C group 5 member C	GPRC5C	Homo sapiens	48.195	1	1	0	52911
9152	1	Q9NQC1	Q9NQC1	E3 ubiquitin-protein ligase Jade-2	JADE2	Homo sapiens	87.468	1	1	95959	0
9153	1	Q9NQG1	Q9NQG1	Protein MANBAL	MANBAL	Homo sapiens	9.465	1	1	116091	21262
9154	1	Q9NQG6	Q9NQG6	Mitochondrial dynamics protein MID51	MIEF1	Homo sapiens	51.294	1	1	105485	50965
9155	1	Q9NR56	Q9NR56	Muscleblind-like protein 1	MBNL1	Homo sapiens	41.815	5	1	40400	0
9156	1	Q9NR77	Q9NR77	Peroxisomal membrane protein 2	PXMP2	Homo sapiens	22.251	1	1	24401	0
9157	1	Q9NRB3	Q9NRB3	Carbohydrate sulfotransferase 12	CHST12	Homo sapiens	48.415	1	1	25748	0
9158	1	Q9NRC8	Q9NRC8	NAD-dependent protein deacetylase sirtuin-7	SIRT7	Homo sapiens	44.899	1	1	7695	13683
9159	1	Q9NRJ3	Q9NRJ3	C-C motif chemokine 28	CCL28	Homo sapiens	14.277	1	1	25405	0
9160	1	Q9NRP0	Q9NRP0	Oligosaccharyltransferase complex subunit OSTC	OSTC	Homo sapiens	16.828	1	1	1781540	677111
9161	1	Q9NRQ2	Q9NRQ2	Phospholipid scramblase 4	PLSCR4	Homo sapiens	37.006	1	1	531232	0
9162	1	Q9NS91	Q9NS91	E3 ubiquitin-protein ligase RAD18	RAD18	Homo sapiens	56.224	1	1	27275	0
9163	1	Q9NSA3	Q9NSA3	Beta-catenin-interacting protein 1	CTNNBIP1	Homo sapiens	9.167	1	1	83244	0
9164	1	Q9NSI6	Q9NSI6	Bromodomain and WD repeat-containing protein 1	BRWD1	Homo sapiens	262.941	1	1	57600	0
9165	1	Q9NSK7	Q9NSK7	Protein C19orf12	C19orf12	Homo sapiens	16.284	1	1	146981	27201
9166	1	Q9NUG6	Q9NUG6	p53 and DNA damage-regulated protein 1	PDRG1	Homo sapiens	15.51	1	1	216552	83959
9167	1	Q9NUK0	Q9NUK0	Muscleblind-like protein 3	MBNL3	Homo sapiens	38.529	1	1	43806	0
9168	1	Q9NUM3	Q9NUM3	Zinc transporter ZIP9	SLC39A9	Homo sapiens	32.253	1	1	62815	11578
9169	1	Q9NUU6	Q9NUU6	Inactive ubiquitin thioesterase OTULINL	OTULINL	Homo sapiens	42.195	1	1	428288	831470
9170	1	Q9NV56	Q9NV56	MRG/MORF4L-binding protein	MRGBP	Homo sapiens	22.416	1	1	74666	42987
9171	1	Q9NV79	Q9NV79	Protein-L-isoaspartate O-methyltransferase domain-containing protein 2	PCMTD2	Homo sapiens	41.073	1	1	73370	0
9172	1	Q9NV92	Q9NV92	NEDD4 family-interacting protein 2	NDFIP2	Homo sapiens	36.388	1	1	71266	0
9173	1	Q9NVA4	Q9NVA4	Transmembrane protein 184C	TMEM184C	Homo sapiens	50.142	1	1	106596	0
9174	1	Q9NW61	Q9NW61	Pleckstrin homology domain-containing family J member 1	PLEKHJ1	Homo sapiens	17.549	1	1	97168	0
9175	1	Q9NWA0	Q9NWA0	Mediator of RNA polymerase II transcription subunit 9	MED9	Homo sapiens	16.401	1	1	73646	25694
9176	1	Q9NWK9	Q9NWK9	Box C/D snoRNA protein 1	ZNHIT6	Homo sapiens	53.919	1	1	35062	0
9177	1	Q9NWT8	Q9NWT8	Aurora kinase A-interacting protein	AURKAIP1	Homo sapiens	22.353	1	1	1001360	0
9178	1	Q9NWZ8	Q9NWZ8	Gem-associated protein 8	GEMIN8	Homo sapiens	28.637	1	1	147778	41271
9179	1	Q9NX08	Q9NX08	COMM domain-containing protein 8	COMMD8	Homo sapiens	21.09	1	1	0	83584
9180	1	Q9NX94	Q9NX94	WW domain binding protein 1-like	WBP1L	Homo sapiens	37.828	1	1	13188	0

9181	1	Q9NXF8	Q9NXF8	Palmitoyltransferase ZDHHC7	ZDHHC7	Homo sapiens	35.142	1	1	74427	0
9182	1	Q9NXL2	Q9NXL2	Rho guanine nucleotide exchange factor 38	ARHGEF38	Homo sapiens	89.077	1	1	102636	0
9183	1	Q9NXL6	Q9NXL6	SID1 transmembrane family member 1	SIDT1	Homo sapiens	93.841	1	1	66025	0
9184	1	Q9NXR5	Q9NXR5	Ankyrin repeat domain-containing protein 10	ANKRD10	Homo sapiens	44.768	1	1	32182	0
9185	1	Q9NXR8	Q9NXR8	Inhibitor of growth protein 3	ING3	Homo sapiens	46.745	1	1	115574	70353
9186	1	Q9NY28	Q9NY28	Probable polypeptide N-acetylgalactosaminyltransferase 8	GALNT8	Homo sapiens	72.851	1	1	41204	0
9187	1	Q9NYP7	Q9NYP7	Elongation of very long chain fatty acids protein 5	ELOVL5	Homo sapiens	35.293	1	1	85525	118321
9188	1	Q9NYZ1	Q9NYZ1	Golgi apparatus membrane protein TVP23 homolog B	TVP23B	Homo sapiens	23.577	2	1	218837	0
9189	1	Q9NZC4	Q9NZC4	ETS homologous factor	EHF	Homo sapiens	34.892	1	1	54304	0
9190	1	Q9NZE8	Q9NZE8	39S ribosomal protein L35, mitochondrial	MRPL35	Homo sapiens	21.514	1	1	191775	66571
9191	1	Q9NZM4	Q9NZM4	BRD4-interacting chromatin-remodeling complex-associated protein	BICRA	Homo sapiens	158.492	1	1	48138	0
9192	1	Q9NZV5	Q9NZV5	Selenoprotein N	SELENON	Homo sapiens	65.755	1	1	86370	29562
9193	1	Q9P0K1	Q9P0K1	Disintegrin and metalloproteinase domain-containing protein 22	ADAM22	Homo sapiens	100.435	1	1	23040	0
9194	1	Q9P0K8	Q9P0K8	Forkhead box protein J2	FOXJ2	Homo sapiens	62.395	1	1	48797	16423
9195	1	Q9P0P8	Q9P0P8	Mitochondrial transcription rescue factor 1	MTRES1	Homo sapiens	27.94	1	1	357699	108526
9196	1	Q9P0S9	Q9P0S9	Transmembrane protein 14C	TMEM14C	Homo sapiens	11.563	1	1	233459	0
9197	1	Q9P0X4	Q9P0X4	Voltage-dependent T-type calcium channel subunit alpha-1I	CACNA1I	Homo sapiens	245.108	1	1	11677	0
9198	1	Q9P1F3	Q9P1F3	Costars family protein ABRACL	ABRACL	Homo sapiens	9.054	1	1	629554	682500
9199	1	Q9P1T7	Q9P1T7	MyoD family inhibitor domain-containing protein	MDFIC	Homo sapiens	25.785	1	1	29932	0
9200	1	Q9P1W3	Q9P1W3	Calcium permeable stress-gated cation channel 1	TMEM63C	Homo sapiens	93.317	1	1	52964	0
9201	1	Q9P299	Q9P299	Coatomer subunit zeta-2	COPZ2	Homo sapiens	23.547	1	1	478854	55552
9202	1	Q9P2G1	Q9P2G1	Ankyrin repeat and IBR domain-containing protein 1	ANKIB1	Homo sapiens	122.004	1	1	70713	0
9203	1	Q9P2H3	Q9P2H3	Intraflagellar transport protein 80 homolog	IFT80	Homo sapiens	88.038	1	1	26576	0
9204	1	Q9P2K1	Q9P2K1	Coiled-coil and C2 domain-containing protein 2A	CC2D2A	Homo sapiens	186.19	1	1	6245	0
9205	1	Q9P2N7	Q9P2N7	Kelch-like protein 13	KLHL13	Homo sapiens	73.869	6	1	16637	0
9206	1	Q9P2R6	Q9P2R6	Arginine-glutamic acid dipeptide repeats protein	RERE	Homo sapiens	172.425	1	1	133123	67066
9207	1	Q9UBF6	Q9UBF6	RING-box protein 2	RNF7	Homo sapiens	12.682	1	1	110903	62540
9208	1	Q9UBI4	Q9UBI4	Stomatin-like protein 1	STOML1	Homo sapiens	42.97	1	1	10548	0
9209	1	Q9UBR5	Q9UBR5	Chemokine-like factor	CKLF	Homo sapiens	17.167	1	1	0	228900
9210	1	Q9UBS3	Q9UBS3	DnaJ homolog subfamily B member 9	DNAJB9	Homo sapiens	25.516	1	1	72448	69889
9211	1	Q9UBU8	Q9UBU8	Mortality factor 4-like protein 1	MORF4L1	Homo sapiens	41.471	2	1	0	66079
9212	1	Q9UDW1	Q9UDW1	Cytochrome b-c1 complex subunit 9	UQCR10	Homo sapiens	7.307	1	1	1664250	392251
9213	1	Q9UET6	Q9UET6	Putative tRNA (cytidine(32)/guanosine(34)-2'-O)-methyltransferase	FTSJ1	Homo sapiens	36.079	1	1	69014	64657
9214	1	Q9UF56	Q9UF56	F-box/LRR-repeat protein 17	FBXL17	Homo sapiens	75.699	1	1	72664	0
9215	1	Q9UGM3	Q9UGM3	Deleted in malignant brain tumors 1 protein	DMBT1	Homo sapiens	260.74	1	1	193141	0
9216	1	Q9UGU5	Q9UGU5	HMG domain-containing protein 4	HMGXB4	Homo sapiens	65.713	1	1	9706	0
9217	1	Q9UH73	Q9UH73	Transcription factor COE1	EBF1	Homo sapiens	64.466	2	1	21225	0
9218	1	Q9UH92	Q9UH92	Max-like protein X	MLX	Homo sapiens	33.303	1	1	108265	0
9219	1	Q9UHA3	Q9UHA3	Probable ribosome biogenesis protein RLP24	RSL24D1	Homo sapiens	19.62	1	1	67290	0
9220	1	Q9UHC7	Q9UHC7	E3 ubiquitin-protein ligase makorin-1	MKRN1	Homo sapiens	53.35	3	1	178871	109078
9221	1	Q9UHL0	Q9UHL0	ATP-dependent RNA helicase DDX25	DDX25	Homo sapiens	54.691	2	1	0	216889
9222	1	Q9UHW5	Q9UHW5	GPN-loop GTPase 3	GPN3	Homo sapiens	32.76	1	1	112931	0
9223	1	Q9UI17	Q9UI17	Dimethylglycine dehydrogenase, mitochondrial	DMGDH	Homo sapiens	96.812	1	1	9716	0
9224	1	Q9UI43	Q9UI43	rRNA methyltransferase 2, mitochondrial	MRM2	Homo sapiens	27.425	1	1	32449	0
9225	1	Q9UID6	Q9UID6	Zinc finger protein 639	ZNF639	Homo sapiens	56.055	1	1	23035	0
9226	1	Q9UIG8	Q9UIG8	Solute carrier organic anion transporter family member 3A1	SLCO3A1	Homo sapiens	76.554	1	1	48292	0
9227	1	Q9UII2	Q9UII2	ATPase inhibitor, mitochondrial	ATP5IF1	Homo sapiens	12.248	1	1	305449	199247
9228	1	Q9UII4	Q9UII4	E3 ISG15--protein ligase HERC5	HERC5	Homo sapiens	116.853	1	1	0	18819

9229	1	Q9UIL8	Q9UIL8	PHD finger protein 11	PHF11	Homo sapiens	37.581	1	1	55921	0
9230	1	Q9UIY3	Q9UIY3	RWD domain-containing protein 2A	RWDD2A	Homo sapiens	33.894	1	1	66424	0
9231	1	Q9UJ72	Q9UJ72	Annexin A10	ANXA10	Homo sapiens	37.277	1	1	6707	17154
9232	1	Q9UJK0	Q9UJK0	18S rRNA aminocarboxypropyltransferase	TSR3	Homo sapiens	33.597	1	1	153088	0
9233	1	Q9UJM8	Q9UJM8	2-Hydroxyacid oxidase 1	HAO1	Homo sapiens	40.924	1	1	0	30959
9234	1	Q9UJQ1	Q9UJQ1	Lysosome-associated membrane glycoprotein 5	LAMP5	Homo sapiens	31.473	1	1	33683	0
9235	1	Q9UK97	Q9UK97	F-box only protein 9	FBXO9	Homo sapiens	52.331	1	1	35762	0
9236	1	Q9UKF7	Q9UKF7	Cytoplasmic phosphatidylinositol transfer protein 1	PITPNC1	Homo sapiens	38.387	1	1	85546	70035
9237	1	Q9UKJ5	Q9UKJ5	Cysteine-rich hydrophobic domain-containing protein 2	CHIC2	Homo sapiens	19.252	1	1	0	104195
9238	1	Q9UKP3	Q9UKP3	Integrin beta-1-binding protein 2	ITGB1BP2	Homo sapiens	38.383	1	1	52411	0
9239	1	Q9UKX3	Q9UKX3	Myosin-13	MYH13	Homo sapiens	223.609	1	1	8567	53815
9240	1	Q9UL01	Q9UL01	Dermatan-sulfate epimerase	DSE	Homo sapiens	109.775	1	1	0	40420
9241	1	Q9ULF5	Q9ULF5	Zinc transporter ZIP10	SLC39A10	Homo sapiens	94.13	1	1	28373	0
9242	1	Q9ULI3	Q9ULI3	Protein HEG homolog 1	HEG1	Homo sapiens	147.463	1	1	54730	0
9243	1	Q9ULK5	Q9ULK5	Vang-like protein 2	VANGL2	Homo sapiens	59.714	1	1	76510	0
9244	1	Q9ULK6	Q9ULK6	RING finger protein 150	RNF150	Homo sapiens	48.071	1	1	23748	0
9245	1	Q9ULL1	Q9ULL1	Pleckstrin homology domain-containing family G member 1	PLEKHG1	Homo sapiens	155.44	1	1	9710	0
9246	1	Q9ULN7	Q9ULN7	Paraneoplastic antigen-like protein 8B	PNMA8B	Homo sapiens	68.614	1	1	25916	0
9247	1	Q9ULT0	Q9ULT0	Tetratricopeptide repeat protein 7A	TTC7A	Homo sapiens	96.187	1	1	0	82717
9248	1	Q9ULW8	Q9ULW8	Protein-arginine deiminase type-3	PADI3	Homo sapiens	74.744	1	1	0	45975
9249	1	Q9ULX7	Q9ULX7	Carbonic anhydrase 14	CA14	Homo sapiens	37.666	1	1	164135	0
9250	1	Q9ULX9	Q9ULX9	Transcription factor MafF	MAFF	Homo sapiens	17.76	1	1	79584	53271
9251	1	Q9UMR2	Q9UMR2	ATP-dependent RNA helicase DDX19B	DDX19B	Homo sapiens	53.928	14	1	31840	0
9252	1	Q9UMR5	Q9UMR5	Lysosomal thioesterase PPT2	PPT2	Homo sapiens	34.224	1	1	65477	0
9253	1	Q9UN81	Q9UN81	LINE-1 retrotransposable element ORF1 protein	L1RE1	Homo sapiens	40.055	1	1	58094	0
9254	1	Q9UNA0	Q9UNA0	A disintegrin and metalloproteinase with thrombospondin motifs 5	ADAMTS5	Homo sapiens	101.72	1	1	0	128667
9255	1	Q9UNK9	Q9UNK9	Protein angel homolog 1	ANGEL1	Homo sapiens	75.276	1	1	21389	0
9256	1	Q9UNL2	Q9UNL2	Translocon-associated protein subunit gamma	SSR3	Homo sapiens	21.079	1	1	1530813	685012
9257	1	Q9UNQ0	Q9UNQ0	Broad substrate specificity ATP-binding cassette transporter ABCG2	ABCG2	Homo sapiens	72.315	1	1	31023	9647
9258	1	Q9UNW9	Q9UNW9	RNA-binding protein Nova-2	NOVA2	Homo sapiens	49.006	3	1	53179	0
9259	1	Q9UPS6	Q9UPS6	Histone-lysine N-methyltransferase SETD1B	SETD1B	Homo sapiens	212.809	1	1	88111	0
9260	1	Q9UPT6	Q9UPT6	C-Jun-amino-terminal kinase-interacting protein 3	MAPK8IP3	Homo sapiens	147.458	1	1	36979	0
9261	1	Q9UPY6	Q9UPY6	Actin-binding protein WASF3	WASF3	Homo sapiens	55.295	1	1	55573	0
9262	1	Q9UPY8	Q9UPY8	Microtubule-associated protein RP/EB family member 3	MAPRE3	Homo sapiens	31.983	1	1	406197	0
9263	1	Q9UQ88	Q9UQ88	Cyclin-dependent kinase 11A	CDK11A	Homo sapiens	91.363	13	1	74515	0
9264	1	Q9Y234	Q9Y234	Lipoyltransferase 1, mitochondrial	LIPT1	Homo sapiens	42.48	1	1	11224	0
9265	1	Q9Y251	Q9Y251	Heparanase	HPSE	Homo sapiens	61.149	1	1	21634	43621
9266	1	Q9Y257	Q9Y257	Potassium channel subfamily K member 6	KCNK6	Homo sapiens	33.745	1	1	120386	54100
9267	1	Q9Y274	Q9Y274	Type 2 lactosamine alpha-2,3-sialyltransferase	ST3GAL6	Homo sapiens	38.214	1	1	0	57478
9268	1	Q9Y275	Q9Y275	Tumor necrosis factor ligand superfamily member 13B	TNFSF13B	Homo sapiens	31.224	1	1	0	29592
9269	1	Q9Y289	Q9Y289	Sodium-dependent multivitamin transporter	SLC5A6	Homo sapiens	68.642	1	1	66001	0
9270	1	Q9Y2I9	Q9Y2I9	TBC1 domain family member 30	TBC1D30	Homo sapiens	102.745	1	1	187423	275408
9271	1	Q9Y2X0	Q9Y2X0	Mediator of RNA polymerase II transcription subunit 16	MED16	Homo sapiens	96.794	1	1	52136	0
9272	1	Q9Y2Y1	Q9Y2Y1	DNA-directed RNA polymerase III subunit RPC10	POLR3K	Homo sapiens	12.321	1	1	119038	0
9273	1	Q9Y2Y8	Q9Y2Y8	Proteoglycan 3	PRG3	Homo sapiens	25.405	1	1	253418	143694
9274	1	Q9Y366	Q9Y366	Intraflagellar transport protein 52 homolog	IFT52	Homo sapiens	49.709	1	1	83446	0
9275	1	Q9Y3A2	Q9Y3A2	Probable U3 small nucleolar RNA-associated protein 11	UTP11	Homo sapiens	30.447	1	1	142907	0
9276	1	Q9Y3B6	Q9Y3B6	ER membrane protein complex subunit 9	EMC9	Homo sapiens	23.062	1	1	85529	72089

9277	1 Q9Y4C0	Q9Y4C0	Neurexin-3	NRXN3	Homo sapiens	180.602	1	1	59163	0
9278	1 Q9Y575	Q9Y575	Ankyrin repeat and SOCS box protein 3	ASB3	Homo sapiens	57.745	1	1	33360	16250
9279	1 Q9Y576	Q9Y576	Ankyrin repeat and SOCS box protein 1	ASB1	Homo sapiens	37.013	1	1	24382	0
9280	1 Q9Y584	Q9Y584	Mitochondrial import inner membrane translocase subunit Tim22	TIMM22	Homo sapiens	20.029	1	1	922459	244714
9281	1 Q9Y592	Q9Y592	Centrosomal protein of 83 kDa	CEP83	Homo sapiens	82.941	1	1	8497	0
9282	1 Q9Y5Q5	Q9Y5Q5	Atrial natriuretic peptide-converting enzyme	CORIN	Homo sapiens	116.488	1	1	35251	0
9283	1 Q9Y5V0	Q9Y5V0	Zinc finger protein 706	ZNF706	Homo sapiens	8.494	1	1	72041	0
9284	1 Q9Y5X0	Q9Y5X0	Sorting nexin-10	SNX10	Homo sapiens	23.598	1	1	0	43917
9285	1 Q9Y5Z0	Q9Y5Z0	Beta-secretase 2	BACE2	Homo sapiens	56.177	1	1	48523	0
9286	1 Q9Y623	Q9Y623	Myosin-4	MYH4	Homo sapiens	223.076	1	1	21147	0
9287	1 Q9Y653	Q9Y653	Adhesion G-protein coupled receptor G1	ADGRG1	Homo sapiens	77.738	1	1	40296	32083
9288	1 Q9Y6A9	Q9Y6A9	Signal peptidase complex subunit 1	SPCS1	Homo sapiens	18.297	1	1	4082880	1340370
9289	1 Q9Y6D0	Q9Y6D0	Selenoprotein K	SELENOK	Homo sapiens	10.617	1	1	219249	88349
9290	1 Q9Y6G3	Q9Y6G3	39S ribosomal protein L42, mitochondrial	MRPL42	Homo sapiens	16.66	1	1	434042	110833
9291	1 Q9Y6I8	Q9Y6I8	Peroxisomal membrane protein 4	PXMP4	Homo sapiens	24.263	1	1	105522	0
9292	1 Q9Y6J6	Q9Y6J6	Potassium voltage-gated channel subfamily E member 2	KCNE2	Homo sapiens	14.469	1	1	158639	0
9293	1 Q9Y6K0	Q9Y6K0	Choline/ethanolaminephosphotransferase 1	CEPT1	Homo sapiens	46.552	1	1	2041730	1225880
9294	1 Q9Y6L7	Q9Y6L7	Tolloid-like protein 2	TLL2	Homo sapiens	113.56	1	1	0	526603
