# Supplementary material for: Endometriosis-Associated Intestinal Tumor: A Case Report and Literature Review with Comprehensive Proteomic Data
Source: Surg Case Rep. 2026 Mar 11;12(1):25-0761. doi: 10.70352/scrj.cr.25-0761 (PMC12991934; doi:10.70352/scrj.cr.25-0761)
Supplement: Supplementary Methods — Detailed methods are described in the Supplementary Appendix. [file scr-12-01-25-0761-s002.pdf]

1. Protein Extraction and Digestion

Tissue samples were lysed in 200 µL of 100 mM Tris-HCl (pH 8.5) containing 4% SDS (Fujifilm Wako Pure Chemical Corp., Osaka, Japan) and sonicated with a Bioruptor II (SONIC Bio Co., Saitama, Japan) using 30 s on/off cycles for 30 min at high intensity. The lysates were then centrifuged at 20,000 × g for 15 min at 4°C, and the supernatants were collected. Protein concentrations were measured with a NanoDrop spectrophotometer (Thermo Fisher Scientific, Waltham, MA, USA) and adjusted to 0.5 µg/µL in 100 mM Tris-HCl (pH 8.5) with 4% SDS.

Proteins were reduced by adding 2 µL of 200 mM TCEP (Thermo Fisher Scientific) and incubating at 80°C for 10 min. Alkylation was performed with 2 µL of 375 mM iodoacetamide in 200 mM TEAB at room temperature for 30 min in the dark. Peptide preparation was carried out using a modified single-pot solid-phase-enhanced sample preparation (SP3) protocol [33,34]. Briefly, 20 µL of magnetic beads were added, followed by ethanol to a final concentration of 75% (v/v). After 10 min of gentle mixing, beads were washed twice with 80% ethanol and resuspended in 100 µL of 50 mM Tris-HCl (pH 8.0) containing 200 ng each of trypsin (Promega) and lysyl endopeptidase (Fujifilm Wako). Digestion was carried out at 37°C for 18 h.

The resulting peptides were acidified with 20 µL of 5% TFA, desalted using MonoSpin C18 (GL Science, Tokyo, Japan), eluted with 50% ACN/0.1% TFA, and lyophilized. Dried peptides were reconstituted in 20 µL of 3% ACN/0.1% formic acid, followed by vortexing and ultrasonication (30 s on/off) for 10 min at 4°C.

---

2. Liquid Chromatography–Mass Spectrometry (LC-MS/MS)

Peptides were loaded onto a 75 µm × 30 cm nanoLC column (CoAnn Technologies, Richland, WA, USA) maintained at 50°C and separated over a 100-min gradient with solvent A (0.1% formic acid in water) and solvent B (0.1% formic acid in 80% ACN): 0 min, 5% B; 86 min, 33% B; 92 min, 70% B; 100 min, 70% B. The flow rate was 150 nL/min on an UltiMate 3000 RSLCnano system (Thermo Fisher Scientific).

Peptides eluting from the column were analyzed on an Orbitrap Exploris 480 mass spectrometer (Thermo Fisher Scientific) coupled to the InSpIon system [35]. MS1 spectra were acquired at 15,000 resolution over m/z 495–865 with an AGC target of 3 × 10^6 and automatic injection time. MS2 spectra were collected at 45,000 resolution over m/z 200–1800 with AGC target 3 × 10^6, stepped collision energy of 26%, and isolation width of 6 Th. Optimized window arrangements for m/z 500–860 were applied. Each sample was analyzed once.

---

3. Protein Identification

Raw data were searched against an in silico human spectral library generated from the human proteome (UP000005640; 20,591 entries; downloaded March 2023) using DIA-NN (v1.8.1; https://github.com/vdemichev/DiaNN; accessed 1 February 2022) . The library was constructed assuming trypsin digestion with up to one missed cleavage, peptide lengths of 7–45 amino acids, precursor charges 2–4, precursor m/z 495–865, and fragment m/z 200–1800. N-terminal methionine excision and C-terminal carbamidomethylation were applied, and deep learning was used to predict spectra, retention times, and ion mobility.

DIA-NN searches employed MS1 and MS2 tolerances of 10 ppm, static cysteine carbamidomethylation, and options including “Remove likely interferences,” “Use isotopologues,” “Unrelated runs,” and “Match between runs.” Peak translation and relaxed-prof-inf commands were also applied. Peptide and protein identifications were accepted at <1% FDR.
